# Isolation of uranocenium, the *bis*-[8]annulene uranium cation

**DOI:** 10.1038/s41467-026-77066-2

**Published:** 2026-10-23

**Authors:** Cameron N. Deakin, Iulia Turcas, Benjamin L. L. Réant, Liam P. Griffin, John A. Seed, Adam Brookfield, David Collison, Eric J. L. McInnes, Andrew Kerridge, Conrad A. P. Goodwin

**Affiliations:** 1 Centre for Radiochemistry Research, The University of Manchester, Manchester, UK; 2 Department of Chemistry, The University of Manchester, Manchester, UK; 3 Photon Science Institute, The University of Manchester, Manchester, UK; 4 Department of Chemistry, Lancaster University, Lancaster, UK

**Keywords:** Synthetic chemistry methodology, Chemical bonding, Chemical bonding

## Abstract

Uranocene, [U^IV^(η^8^-C_8_H_8_)_2_], is the actinide equivalent to ferrocene. While ferrocene is readily oxidised to the ferrocenium cation, higher oxidation states of uranocene are unknown. Here we report the elusive uranocenium cation, [U^V^(η^8^-C_8_H_8_)_2_]^+^, and demonstrate using electron paramagnetic resonance spectroscopy and magnetometry that this formal U(V) complex has a 5 *f* ^1^ ground state. Enhanced uranium 5 *f* and 6 *d* mixing into filled δ-symmetry bonding orbitals in uranocenium, compared to uranocene, amplifies interorbital electron–electron repulsion, and counteracts bond length contraction, which minimises structural change. This manifests in solution where the uranocene/uranocenium redox couple exhibits near-ideal electrochemical reversibility, and in the solid-state where X-ray diffraction studies show minimal U–C bond length change upon oxidation. This demonstrates a case where actinide covalency governs molecular structure and physical properties across both solid and solution phases.

## Abstract (web summary)

While ferrocene is readily oxidized to the ferrocenium cation, higher oxidation states of uranocene are unknown. Here the authors report the elusive uranocenium cation and demonstrate that this formal U(V) complex has a 5 *f* ^1^ ground state and a molecular structure directed by significant uranium–ligand covalency.

## Introduction

Metallocenes, such as ferrocene, [Fe^II^(Cp)_2_], (Cp = {η^5^-C_5_H_5_}) are composed of a metal ion between two coplanar 6π Hückel aromatic cyclopentadienide anions^1–5^. While the metallocene motif finds use across the Periodic Table^6,7^, the large ionic radii and negative reduction potentials of actinide (An) elements essentially preclude the isolation of [An^II^(Cp)_2_] complexes^8^. Replacing Cp with the ring-expanded 10π Hückel aromatic *cyclo*-octatetraenide (COT, {η^8^-C_8_H_8_}^2–^) gives “actinocene” complexes – [An^IV^(COT)_2_] – such as uranocene^9–11^. Actinocenes serve as touchstones for evaluating 5*f*-derived bonding and actinide covalency, and understanding their electronic structure shaped the field of actinide chemistry in much the same way as ferrocene influenced *d*-block organometallic chemistry^12–22^.

Despite the centrality of uranocene, the one-electron-oxidized uranocenium cation, [U^V^(COT)_2_]^+^, the equivalent of the ferrocenium cation [Fe^III^(Cp)_2_]^+^, has not been isolated. Electrochemical oxidation of uranocene has been described as irreversible, yielding poorly defined products^23,24^, and reports of the [U(COT)_2_]^+^ cation in any oxidation state are limited to theory^25^, or as minor products in the gas phase^26,27^. We now report isolation of the parent uranocenium cation, [U^V^(COT)_2_]^+^, and a substituted analogue, [U^V^(COT^††^)_2_]^+^ (COT^††^ = {C_8_H_6_−1,4-(Si*i*Pr_3_)_2_}^2–^), as crystalline room-temperature stable borate salts and compare the electronic structure and bonding in both to anionic U(III) uranocenate complexes, and neutral U(IV) uranocene congeners.

## Results

### Synthesis & electrochemistry

**Fig. 1:**
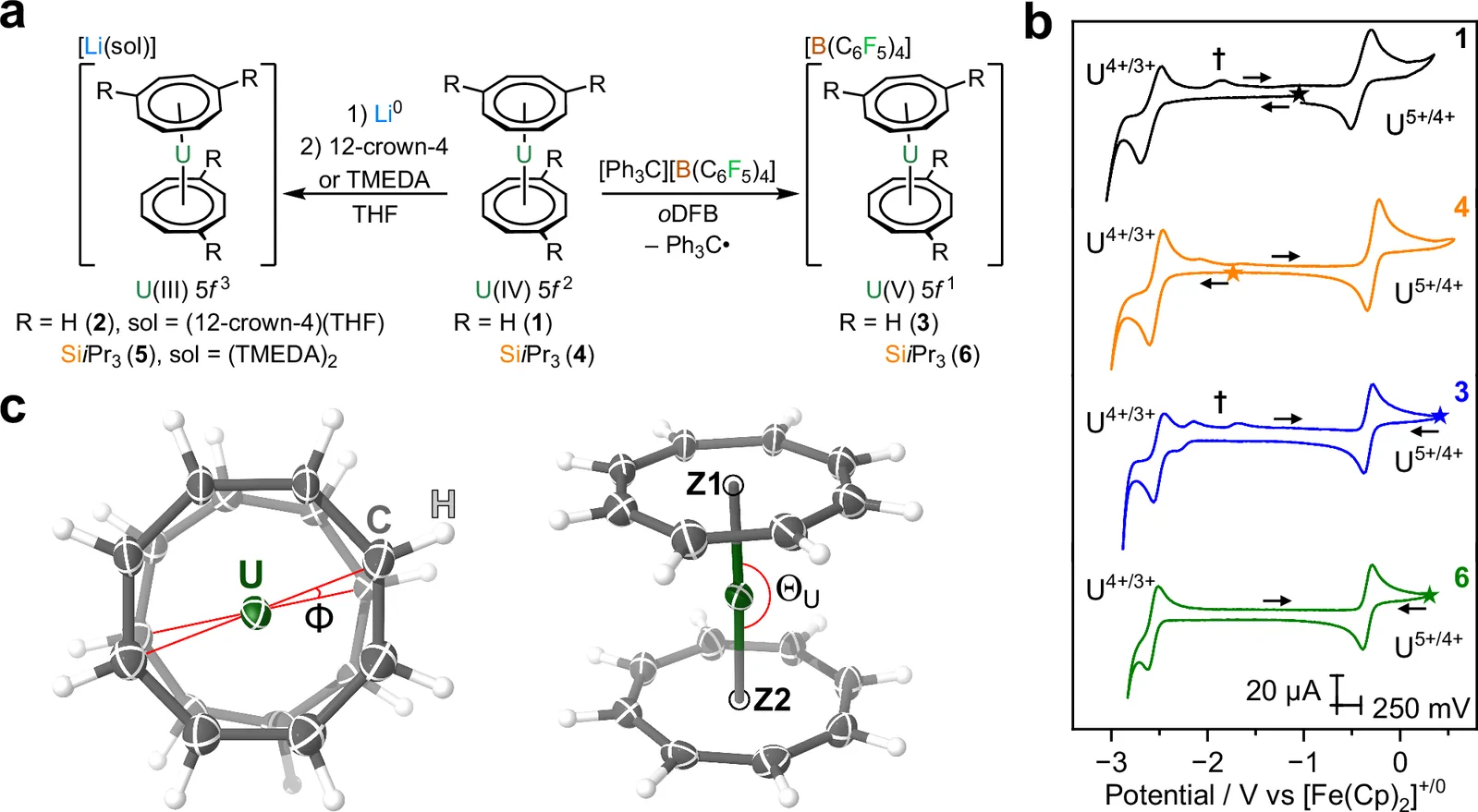
Synthesis, cyclic voltammetry, and molecular structure of uranocene complexes. **a** Synthesis of uranocenate anions and uranocenium cations as separated ion pairs from neutral uranocene complexes. Ph_3_C• refers to the by-product from the reduction of [Ph_3_C]^+^. **b** Cyclic voltammograms in THF (**1**, black; **4**, orange) or *ortho*-difluorobenzene (*o*DFB, **3**, blue; **6**, green), as 3 mM solutions supported by [*n*Bu_4_N][B(C_6_F_5_)_4_] (200 mM) at 200 mV s^–1^ at room temperature. * symbols indicate the starting point on each cycle, arrows show the scan direction, ^†^ denotes unidentified processes. **c** Molecular structure of the cation in **3** with thermal ellipsoids at 30% probability. Φ is the ring-twist angle, Z1 and Z2 are the COT ring centroids, and Θ_U_ is the angle between these and the U-atom. Dark green = U, grey = C, white = H.

Salt elimination between K_2_COT or K_2_COT^††^ and U^IV^Cl_4_, gave [U^IV^(COT)_2_], uranocene (**1**), and [U^IV^(COT^††^)_2_] (**4**) respectively. These were purified by modifying established routes (see *Supplementary Information* Section 1)^14,28–31^. Reduction of **1** or **4** with Li^0^ metal and catalytic naphthalene in tetrahydrofuran (THF) at room temperature under argon gave dark solutions over three days. Treatment with 12-crown-4 or *N*,*N*,*N*′,*N*′-tetramethyl-ethylenediamine (TMEDA) followed by recrystallisation from THF or hot mesitylene gave uranocenate complexes [Li(12-crown-4)(THF)][U^III^(COT)_2_] (**2**) and [Li(TMEDA)_2_][U^III^(COT^††^)_2_] (**5**) as phase-pure orange/brown crystals (Fig. 1a).

Parent or substituted actinocenes are known for thorium^28,29^, uranium^28^, neptunium^14,30^, plutonium^14,31^, and berkelium^32^, but only for the trivalent and tetravalent oxidation states. An irreversible anodic wave (*E*_pa_ value only) for **1** has been reported at 0.33 V *vs* SCE in dichloromethane^23,24^, or –0.13 V when referenced to [Fc]^+/0^ (ref. ^33^). This suggests mild non-aqueous oxidants might give [U^V^(COT)_2_]^+^ (ref. ^33^). The organic oxidant [Ph_3_C][B(C_6_F_5_)_4_] was explored to avoid electrophilic transition-metal cations and nucleophiles that might displace COT rings. Both **1** and **4** react rapidly with [Ph_3_C][B(C_6_F_5_)_4_] in *ortho*-difluorobenzene (*o*DFB) at room temperature to give [U^V^(COT)_2_][B(C_6_F_5_)_4_] (**3**) and [U^V^(COT^††^)_2_][B(C_6_F_5_)_4_] (**6**), respectively (Fig. 1a). Complexes **3** and **6** are stable indefinitely at room temperature in *o*DFB, bromobenzene, Et_2_O, and in the solid-state. When exposed to THF in *o*DFB solution (1:5 w/w), both complex **3** and **6** initiate the living-chain polymerisation of THF following 1^st^ order reaction kinetics (*t*_1/﻿2_ = 21.4 and 24.8 minutes, respectively). In both cases we find direct conversion of the U(V) complexes into their respective U(IV) precursors (**1** and **4**) without intermediates observed by UV-Vis-NIR spectroscopy, evidenced by the presence of isosbestic points in both. The lifetime of complexes **3** and **6** in neat THF was too short for analysis, but kinetic data gathered in 2-MeTHF revealed longer lifetimes (*t*_1/2_ = 115.5 and 187.3 minutes, respectively) than in the dilute THF/*o*DFB solutions (see Supplementary Figs. S129–S136). The oxidation of **1** with [Ph_3_C][B(C_6_F_5_)_4_] was also explored in fluorobenzene and *d*_5_-bromobenzene, which gave **3** as the sole uranium-containing product on both occasions, suggesting *o*DFB is not unique as a suitable solvent and others may be appropriate provided they are unreactive towards the formal U(V) centre.

Cyclic voltammograms of **3** and **6** in *o*DFB with [*n*Bu_4_N][B(C_6_F_5_)_4_] electrolyte (Fig. 1c and Table 1) showed diffusion-limited U(V/IV) redox couples with half-wave potentials (*E*_1/2_) at –0.33 V (**3**) and –0.34 V (**6**). At a scan rate of 25 mV s^–1^, the peak-to-peak separation (Δ*E*_p_) for the oxidation and reduction waves in **3** is 80 mV, for **6** it is 100 mV, and for ferrocene it is 100 mV. At 1,000 mV s^–1^, Δ*E*_p_ for ferrocene increases to 280 mV, while **3** (100 mV) and **6** (160 mV) both show smaller values. The U(V/IV) Δ*E*_p_ values are comparable to or smaller than those of ferrocene at all scan rates – which, therefore, defines the uranocene/uranocenium couple as fully reversible by analogy to the ferrocene/ferrocenium couple.

**Table 1:** Summary of electrochemical data for the U(IV/III) and U(V/IV) redox couples in 1, 3, 4, and 6

200 mV s^–1^	U(IV/III)				U(V/IV)			
	1 ^A^	4 ^A^	3 ^B^	6 ^B^	1 ^A^	4 ^A^	3 ^B^	6 ^B^
***E***_**1/2**_	–2.58	–2.53	–2.50	–2.57	–0.41	–0.28	–0.33	–0.34
***E***_**pc**_ **/ V**	–2.65	–2.62	–2.57	–2.64	–0.53	–0.35	–0.38	–0.39
***E***_**pa**_ **/ V**	–2.51	–2.44	–2.43	–2.50	–0.29	–0.21	–0.28	–0.29
**Δ*E***_**p**_ **/ mV**	140	180	140	140	240	140	100	100
***i***_**pa**_**/*i***_**pc**_	0.63	1.00	0.86	0.97	1.05	1.05	0.99	1.02

^A^ 3.0 mM analyte in THF supported by [*n*Bu_4_N][B(C_6_F_5_)_4_] (0.2 M). ^B^ 3.0 mM analyte in *o*DFB supported by [*n*Bu_4_N][B(C_6_F_5_)_4_] (0.2 M).

Despite the reaction between **3** or **6** with THF, cyclic voltammetry of neutral **1** and **4** in THF with [*n*Bu_4_N][B(C_6_F_5_)_4_] electrolyte showed the U(V/IV) couples to be essentially identical to those of **3** and **6** (in *o*DFB), suggesting that ion-pairing effects are minimal, and that on the electrochemical timescale reaction with solvents does not account for previous reports stating the [U^V^(COT)_2_]^+^ cation was unstable^23,24^. When [*n*Bu_4_N][PF_6_] was explored as the supporting electrolyte, as in previous works^23,24^, only irreversible oxidation waves were observed for both **1** and **4**. The electrochemical data suggest that other oxidants may be suitable for the oxidation of **1**, provided the anionic component is unreactive towards the [U^V^(COT)_2_]^+^ cation. The oxidants [Fc][B(C_6_F_5_)_4_] and Ag^I^[B(C_6_F_5_)_4_] both feature the same anion as in **3**, and so the nature of the oxidising component can be examined in isolation. While **1** is smoothly oxidised by [Fc][B(C_6_F_5_)_4_] to give **3**, and indeed **3** is unreactive to Fc as shown in a separate experiment, the reaction with Ag^I^[B(C_6_F_5_)_4_] gave neutral COT as the sole identifiable product. Alternative Ag(I) oxidants such as Ag^I^[B{C_6_H_3_−3,5-(CF_3_)_2_}_4_] and Ag^I^[SbF_6_] gave similar results (see *Supplementary Information* Section 4).

**Fig. 2:**
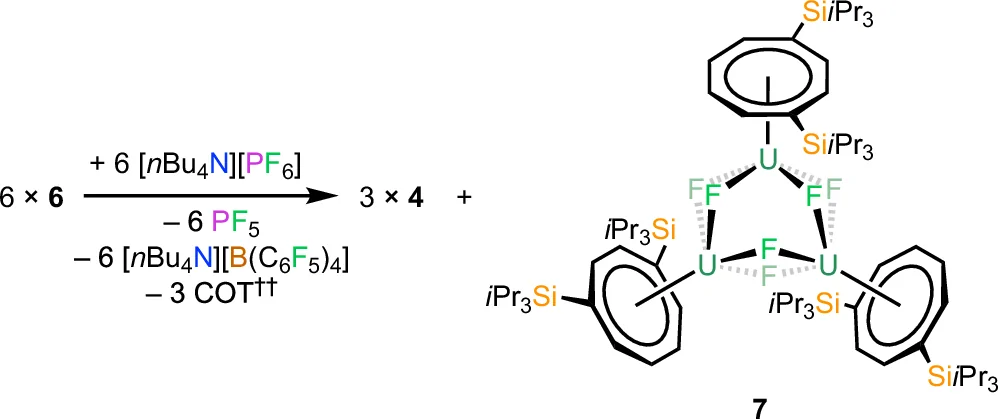
Fluoride abstraction by a uranocenium cation. The stoichiometric reaction between complex **6** and [*n*Bu_4_N][PF_6_] produces multiple species identified by multinuclear NMR spectroscopies, and the fluoride-bridged U(IV) trimer complex **7**.

The stoichiometric reaction between six equivalents of isolated **6** and [*n*Bu_4_N][PF_6_] in *o*DFB or *d*_5_-bromobenzene gave a 1:3:3:6 mixture of [{U^IV^(COT^††^)(μ-F)_2_}_3_] (**7**), complex **4**, neutral COT^††^, and [*n*Bu_4_N][B(C_6_F_5_)_4_], by nuclear magnetic resonance (NMR) spectroscopy, and the structure of **7** was determined by single-crystal X-ray diffraction (SCXRD) (see Fig. 2 and *Supplementary Information*). The formation of two U(IV) products (**4** and **7**) in a 1:3 ratio from one U(V) precursor (**6**) requires a reducing equivalent, which is satisfied by the elimination of neutral COT^††^. Complex **7** features new U–F bonds requiring a fluoride source. No [B(C_6_F_5_)_4_]^–^ degradation products were found by ^11^B NMR spectroscopy of the reaction mixture, and complexes **3** and **6** are stable indefinitely in *o*DFB and bromobenzene. Defluorination of [PF_6_]^–^, producing PF_5_, is the most likely fluoride source, but PF_5_ is a gas (boiling point, b.p. = –84.6 °C) and reacts with the borosilicate glass surface of the reaction tube giving POF_3_, which is also a gas (b.p. = –39.7 °C) – hindering detection by NMR spectroscopy. To confirm the formation of PF_5_, THF was added to the reaction mixture to form the PF_5_–THF solvate^34^. This was performed after the reaction was complete by ^1^H NMR spectroscopy to circumvent the reaction between **6** and THF. NMR spectroscopy showed POF_3_ (δ_F_ = –89.91, δ_P_ = –33.94; where δ refers to the chemical shift in ppm between the observed peak and a reference standard), the PF_5_–THF solvate (δ_F_ = –73.63, δ_P_ = –146.80), and free PF_5_ (δ_F_ = –67.76, δ_P_ = –134.85). The analogous reaction with **3** yielded insoluble products, precluding analysis by NMR spectroscopy. However, multinuclear NMR spectroscopy of the solution above the insoluble products over the course of 144 hours showed the growth of signals for PF_3_, PF_5_, and POF_3_ (Supplementary Figs. S88 and S89) as seen with **6**. These results rationalise electrochemical data gathered using [PF_6_]^–^-based electrolytes^23,24^, and show that the apparent irreversibility of uranocene oxidation under these conditions is from prompt reaction of the uranocenium cation with the electrolyte.

Defluorination of [PF_6_]^–^ is a known degradation pathway for this anion in the presence of Lewis-acids or redox active metal centres^35–38^. Given the propensity for fluoride abstraction from [PF_6_]^–^ by both **3** and **6**, we explored the anion-exchange reaction of **6** using [*n*Bu_4_N][SbF_6_] as Sb(V) is more Lewis-acidic than P(V), and therefore perhaps more resistant to defluorination. Complex **6** was chosen due to the greater solubility imparted by the COT^††^ ligands to aid in the isolation of any crystalline products, as seen with **7**. When *d*_5_-bromobenzene was added to a mixture of [*n*Bu_4_N][SbF_6_] and **6**, a dark coloured insoluble material quickly formed. ^1^H NMR spectroscopy of the solution revealed the presence of neutral COT^††^, indicating a redox process had occurred, and none of the resonances associated with **6** remained. After 24 hours, the relative integrals of neutral COT^††^ to the [*n*Bu_4_N] cation reached approximately 1:1. The ^19^F{^1^H} NMR spectrum showed a weak signal corresponding to [SbF_6_]^–^, but quadrupolar broadening from ^121^Sb (*I* = ^5^/_2_) and ^123^Sb (*I* = ^7^/_2_) precluded the identification of any other Sb-containing products. Given that **6** is stable indefinitely in the solvent the reaction was performed in, these data suggest that the [U^V^(COT^††^)_2_]^+^ cation is unstable in the presence of [SbF_6_],^–^ but the reaction pathway is unknown at this stage.

### Molecular structures

Complexes **1** and **4** crystallise with half a molecule in the asymmetric unit and show eclipsed configurations where **1** approximates to *D*_8h_ symmetry, and **4** has *C*_2_ symmetry with the molecular COT···U···COT axis lying perpendicular to the symmetry axis. Complexes **2** and **5** show a decrease in symmetry where both crystallise with one molecule in the asymmetric unit as separated ion pairs with long C_COT_···Li distances (**2**, 5.343(10) Å; **5**, 4.741(6) Å). Lastly, both **3** (cation shown in Fig. 1c) and **6** have two independent molecules in their asymmetric units, but in each case the ion pairs are well separated with nearest U···F contacts >4 Å throughout, and no uranium–solvent contacts. In **3**, the [B(C_6_F_5_)_4_]^–^ anions and lattice *o*DFB provide a fluorinated cavity in which the cation resides, whereas the Si*i*Pr_3_ groups in **6** cause neighbouring cations to pack closely, and *n*-hexane molecules occupy void spaces. Table 2 gives key structural parameters for complexes **1**–**6**.

**Table 2:** Select metrical parameters for the molecular structures of complexes **1**–**6**, ordered by the formal oxidation state of the uranium ion in each series

Å / ° ^A^	2 U(III)	1 U(IV) ^B^	3 U(V)	5 U(III)	4 U(IV)	6 U(V)
U–C_range_ U(1) ^C^ U(2)	2.716(5)–2.756(5) –	2.639(13)–2.651(14) –	2.559(14)–2.651(12) 2.55(3)–2.653(10)	2.702(3)–2.824(2) –	2.632(3)–2.748(2) –	2.619(3)–2.769(3) 2.619(3)–2.735(3)
U–C_mean_^D^	2.732(5)	2.646(11)	2.628(11)	2.752(3)	2.684(3)	2.661(2)
U···Z1/Z2 U(1) U(2)	2.011(2) 2.024(2) –	1.912(4) –	1.850(5) 1.878(3) 1.855(5) 1.869(3)	2.0343(9) 2.0419(9) –	1.9451(10) –	1.9075(10) 1.9244(10) 1.9115(10) 1.9125(10)
Θ_U_ U(1) ^E^ U(2)	173.87(8) –	180 –	176.64(18) 177.64(19)	173.39(4) –	177.34(5) –	171.99(5) 171.33(4)
ϕ ^F^	3.6(3)	0	10.1(6) 9.0(5)	0.11(11)	1.05(12)	1.55(13) 3.48(12)

^A^ Unless stated otherwise, numbers in parentheses are the standard uncertainty determined from the X-ray diffraction measurement. ^B^ Complex **1** has been reported previously, but we provide new data collected at 100 K for comparison with **2**–**6** (refs. ^10,28^). ^C^ Only values from the largest occupied disordered component were used. ^D^ Numbers in parentheses give the weighted standard deviation^82^. ^E^ Θ_U_ refers to the angle defined by both COT_centroid_ positions (Z1 and Z2), and the uranium atom. ^F^ ϕ refers to the twist angle between COT rings.

The ionic radius of uranium decreases from U(III) (1.025 Å) to U(IV) (0.89 Å) and U(V) (0.76 Å) in six-coordinate systems, the only comparison point available for U(III)^39^. Table 2 shows the mean U–C distance decreases by 0.086(12) Å (where the number in parentheses is the root sum square uncertainty between the two means) from **2** to **1** (and by 0.068(4) Å from **5** to **4**) – somewhat less than the ionic radius difference between U(III) and U(IV) (0.135 Å). Oxidation to U(V) in **3** and **6** causes further contraction in the mean U–C distances by 0.018(16) Å and 0.023(4) Å, respectively, which either do not, or barely, reach statistical significance. A comparison of the U···COT_centroid_ distances across the series shows the same trend. Together, these data indicate that the bond length contraction between the uranocene and uranocenium compounds is two to three times smaller than the ionic radius difference between U(IV) and U(V) (0.13 Å)^39^. Several lanthanide {M^III^(COT)_2_}^n–^ complexes exist for metals with larger ionic radii but shorter M–C distances than in **3** or **6** (refs. ^40,41^), which suggests this discrepancy is not steric in nature.

### EPR spectroscopy and SQUID magnetometry

**Fig. 3:**
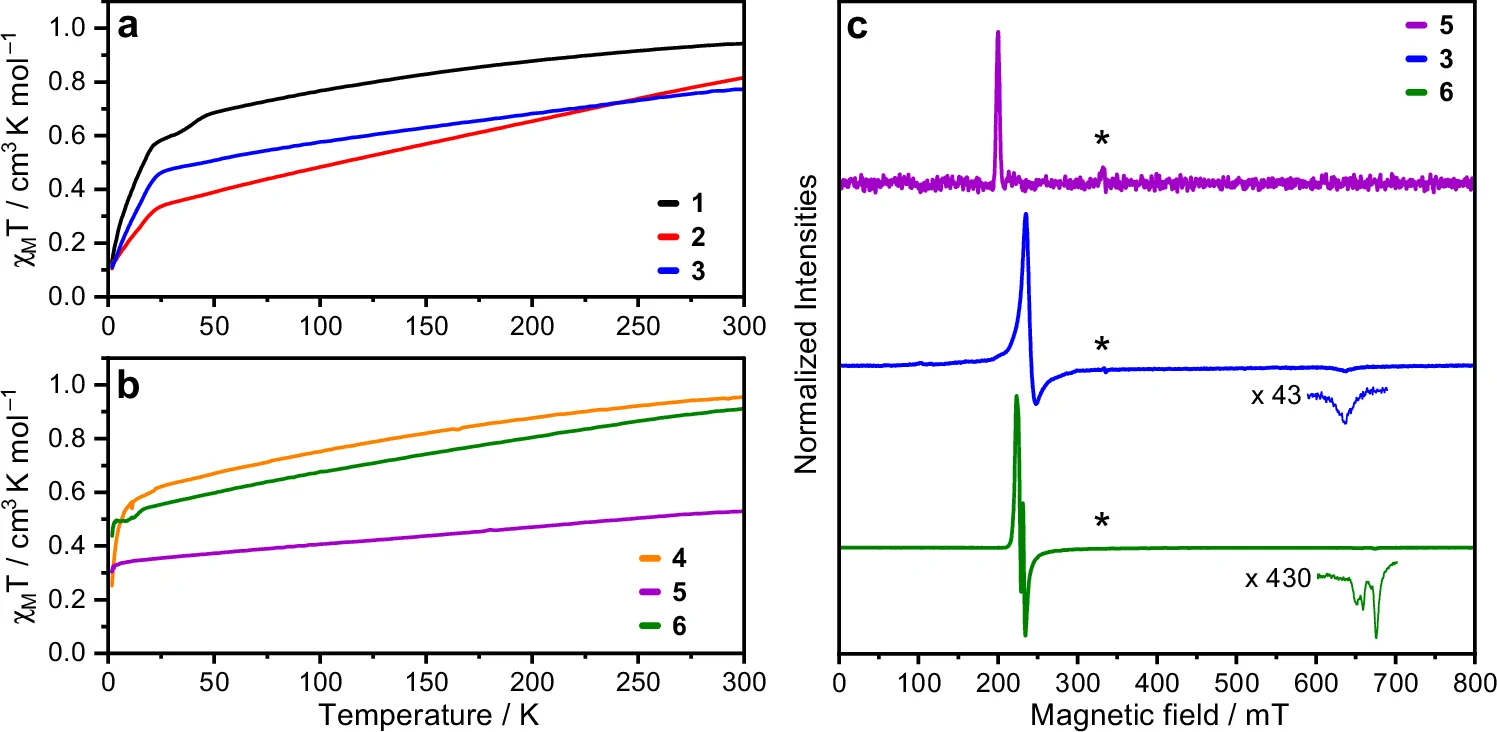
Electronic ground state characterisation. **a**,**b** Temperature dependence of ${\chi }_{M}T$ for U(IV) **1** (black) and **4** (orange); U(III) **2** (red) and **5** (purple); and U(V) **3** (blue) and **6** (green), measured in the solid state by field-cooled SQUID magnetometry (1.8–300 K). **c** Experimental X-band powder EPR spectra of **5** (purple), **3** (blue), and **6** (green) at 5 K. * indicates a weak signal in the resonator cavity.

Continuous-wave X-band electron paramagnetic resonance (EPR) spectroscopy and variable-temperature SQUID magnetometry measurements were carried out to characterise the nature of the electronic ground states of **1**–**6**. Magnetic data for all complexes support well-isolated ground doublets (or pseudo doublets), showing mostly weak near-linear variable-temperature dependence down to *ca*. 20 K (Fig. 3a, b). Lower-temperature field-cooled and zero-field-cooled magnetic susceptibility data diverged due to the onset of slow magnetisation dynamics, so low-temperature limiting molar magnetic susceptibility temperature product values (${\chi }_{M}T$; where *T* is temperature) were estimated by extrapolation and are consistent with the EPR-assigned ground states and effective *g*-values.

The EPR spectrum of a powder sample of **3** at 5 K (Fig. 3c) takes the form of an effective spin *S* = ½ with axial effective *g*-values, *g*_*x,y*_ = 2.83 and *g*_z_ = 1.06. Spectra of **6** have features at similar fields but with more structure. The spectra of **6** can be simulated as arising from two effective *S* = ½ species with *g*_*x,y,z*_ = 3.01, 2.95, 1.00 and *g*_*x,y,z*_ = 2.96, 2.89, 1.03, respectively (see *Supplementary Information* Section 8). The slight rhombicity in the effective *g*-values is consistent with symmetry-breaking due to the Si*i*Pr_3_ groups, and the two similar species is consistent with the two molecules found in the asymmetric unit of the crystal structure.

The spectra of **3** and **6** are consistent with well-isolated Kramers doublet (KD) ground states. Under the assumption of Russell-Saunders (RS) coupling, the U(V) ion gives a ^2^F_5/2_ ground multiplet, which is split into a series of KDs by the ligand field. In *D*_8h_ symmetry, these KDs are pure $\left|{m}_{J}\right|$ states (although mixing with the ^2^F_7/2_ excited state is allowed). The experimental effective *g*-values for **3** and **6** are reasonably close to those expected for the pure $|J,{m}_{J}\rangle=|5/2,\pm 1/2\rangle$ KD (*g*_*x,y*_ = 2.57 and *g*_z_ = 0.86) (see Supplementary Tables S26 and S27). Similar *g*-values, but with lower *g*_x,y_, have been reported by Gourier and co-workers for the related *bis*-(cycloheptatrienyl)-uranium anion [U^V^(η^7^-C_7_H_7_)_2_]^–^ (*g*_*x,y*_ = 2.37 and *g*_z_ = 1.24) which they also assign as dominated by the $|J,{m}_{J}\rangle=|5/2,\pm 1/2\rangle$ KD^42^. They concluded that deviations were primarily due to mixing with $|J,{m}_{J}\rangle=|5/2,\pm 5/2\rangle$. They argued that the effect of the δ-bonding is to push the |*m*_l_ | = 2 (5 *f*_δ_) orbitals to much higher energy and hence these states have little effect on the ground state KD. This leaves mixing with states deriving from |*m*_l_ | = 0, 1, 3 to dominate; this can also be described in terms of mixing of |*J*,*m*_*J*_ > = |5/2, ±1/2> with |*J*,*m*_*J*_ > = |5/2, ±5/2> dominating deviations from the pure |5/2, ±1/2> *g*-values, with |*J*,*m*_*J*_ > = |5/2, ±3/2> at much higher energy. Either way, modelling shows that this leads to a decrease of |*g*_x,y_| from 18/7 or to highly rhombic *g*-values (depending on admixtures allowed under the assumed symmetry)^25,42^. Interestingly, similar *g*-values are found for the valence isoelectronic 4 *f* ^1^ complexes [Li(TMEDA)_2_][Ce^III^(COT^††^)_2_] (see Supplementary Table S26; *g*_x_ = 2.35, *g*_y_ = 2.25, *g*_z_ = 1.09) and [Li(THF)_4_][Ce^III^(COT)_2_] (*g*_x_ = 2.270, *g*_y_ = 2.274, *g*_z_ = 1.123)^43^. This can be explained by a second-order admixture with the $|J,{m}_{J}\rangle=|7/2,\pm 1/2\rangle$ KD of the ^2^${{\rm{F}}}_{7/2}$ manifold (see *Supplementary Information* Section 8 – Supplementary Figs. S217 and S218). Hence, there are potential competing effects in the observed *g*-values. The models above do not rationalise our finding of |*g*_x,y_ | > 18/7; however, we note that our active space calculations are in quantitative agreement, giving |*g*_*x,y*_ | = 2.83 (2.64) and |*g*_z_ | = 1.15 (1.12) at the complete active space (restricted active space) self-consistent field [CAS(RAS)SCF] level. Previous calculations on [U^V^(COT)_2_]^+^ in *D*_8h_ symmetry gave lower values of |*g*_*x,y*_ | = 2.43 and |*g*_z_ | = 1.11, albeit with a smaller active space that captured the metal–ligand interaction less precisely^25^.

Complex **2** is EPR-silent, consistent with a KD with pure $|{m}_{J}| > 1/2$, where the U(III) ion has a ^4^I_9/2_ ground Russell-Saunders term. The spectrum of **5** (Fig. 3c) has a weak feature at *g*_z_ = 3.34; the absorption-like shape assigns this to the unique axis of an axial spectrum. We do not observe the *g*_x,y_ features, but the upper limit is *g*_x,y_ < 0.4 (based on the field range of our electromagnet). The effective *g*_z_ is close to that expected for the $|J,{m}_{J}\rangle=|9/2,\pm 5/2\rangle$ KD (*g*_z_ = 3.64); this is confirmed by RASSCF calculations (*g*_z_ = 3.42 and *g*_x,y_ = 0.02) and previous calculations for both [U^III^(COT)_2_]^–^ (calculated *g*_z_ = 3.38 and *g*_x,y_ = 0.07)^44^ and valence isoelectronic [Nd^III^(COT)_2_]^–^ (calculated *g*_z_ = 3.485 and *g*_x,y_ = 0.003)^45^. Transitions within the pure $|{m}_{J}|=5/2$ KD would be EPR forbidden (*g*_x,y_ = 0), and the low signal-to-noise ratio of the EPR spectrum of **5** is consistent with a weak transition probability. The latter is only non-zero due to symmetry breaking by the Si*i*Pr_3_ groups, enabling mixing of the predominantly ${m}_{J}\approx \pm 5/2$ ground KD with higher lying $|{m}_{J}|=3/2,\,7/2$ KDs. The EPR silence of **2** is then consistent with the higher symmetry and purer ground state.

Taken together, these experimental data describe complexes **3** and **6** as containing formal U(V) 5 *f* ^1^ metal centres, despite the presence of two strongly reducing dianions around a highly oxidising U(V) centre. Some authors have argued the U-centre in [U(η^7^-C_7_H_7_)_2_]^–^ is best described as U(III) based on computational studies and partitioning pairs of bonding electrons in mixed ligand and $5{f}_{\delta }$ orbitals^46^, whereas EPR spectroscopy and magnetic studies have been interpreted in terms of a formal U(V) oxidation state in [U^V^(η^7^-C_7_H_7_)_2_]^–^ with extensive $5{f}_{\delta }$ mixing^42^. Others highlight the nuance between assigning formal oxidation state and descriptions of electron density or metal charge derived from spectroscopic and theoretical analyses^47,48^. The same criteria used to assign [U^V^(η^7^-C_7_H_7_)_2_]^–^ as containing a U(III) centre also apply to complex **3**, which has very high uranium content in the bonding δ-orbitals. However, magnetic, spectroscopic, and various computational analyses comparing the unambiguous U(III) complex **2** to formal U(V) complex **3** show they are entirely distinct. It is for this reason that we describe the formal oxidation state of the metal in all cases herein, and defer to further spectroscopic investigations into the new actinocenium class of molecules.

### Electronic structure calculations

Prior calculations on uranocene at the density functional theory (DFT) level describe the two unpaired valence 5 *f* electrons as residing in metal-localised 5 *f*_ϕ_-symmetry (*i.e*. ${{5f}_{\phi }}^{2}$, ${e}_{3u}$ under *D*_8h_ symmetry) non-bonding molecular orbitals (MOs)^12,13^. This contrasts with calculations performed at higher levels of theory, which give a ${{5f}_{\pi }}^{1}{{5f}_{\phi }}^{1}$ configuration^49–51^. Nevertheless, topological analyses of the electron density via the quantum theory of atoms in molecules (QTAIM) approach in complex **1** reveals qualitatively similar results using wavefunctions derived from DFT calculations^52^, and from CASSCF calculations^49^. As *f*-element bonding is often highly ionic in character, oxidation and reduction typically drive large bond-length changes and structural reorganisation that trend with their ionic radii and coordination numbers^39^. This is not the case when comparing uranocene (**1** or **4**) to the uranocenium cations (**3** or **6**) in both the solid-state and solution; therefore, an explanation was sought in their electronic structures.

Active space calculations were performed on complexes **1**–**3** using coordinates from the SCXRD studies with optimised H-atom positions (see Methods). These calculations showed no substantial multiconfigurational character, while *f*-orbital occupation and electron density analyses agree with those obtained using unrestricted DFT calculations, justifying the use of the latter for subsequent analysis. Calculations were performed on complexes **1**–**6** at the PBE0 level of theory^53,54^, using structures as detailed above. Additional DFT functionals were explored with complexes **1** and **3** spanning GGA, meta-GGA, and hybrid(meta)-GGA functionals and a range of included Hartree-Fock exchange (HFX) – see (Supplementary Figs. S242 and S243). Analysis of the MO compositions and energies in **1** and **3** showed that separation in energy between the 5 *f* and 6*d*-derived δ-bonding MOs was similar across all functionals except when very large values of HFX were included. The most pertinent difference in all cases is how the energy of the non-bonding 5 *f* electron(s) in **1** and **3** changed relative to the δ-bonding MOs. Functionals with greater (or any) HFX included generally reported the MO which comprises the non-bonding 5 *f* electron(s) as close to, or below, the energy of the occupied δ-bonding orbitals, whereas those less, or no HFX included gave this MO as above the δ-bonding orbitals. We proceed with analysis based on orbitals derived from calculations at the PBE0 level, which has been successful with other actinide systems, and note that while orbital energies have a functional dependence, orbital compositions are similar across all methods.

The U(III) complexes have natural spin densities at uranium of 3.02 (**2**) and 3.01 (**5**), consistent with 5 *f* ^3^ configurations in an ionic bonding environment. Conversely, the U(IV) complexes are 10% above that expected for 5 *f* ^2^ ions (**1**, 2.23; **4**, 2.22), and both U(V) complexes are 40-50% higher (**3**, 1.39; **6**, 1.55) than expected for 5 *f* ^1^ ions.

**Fig. 4:**
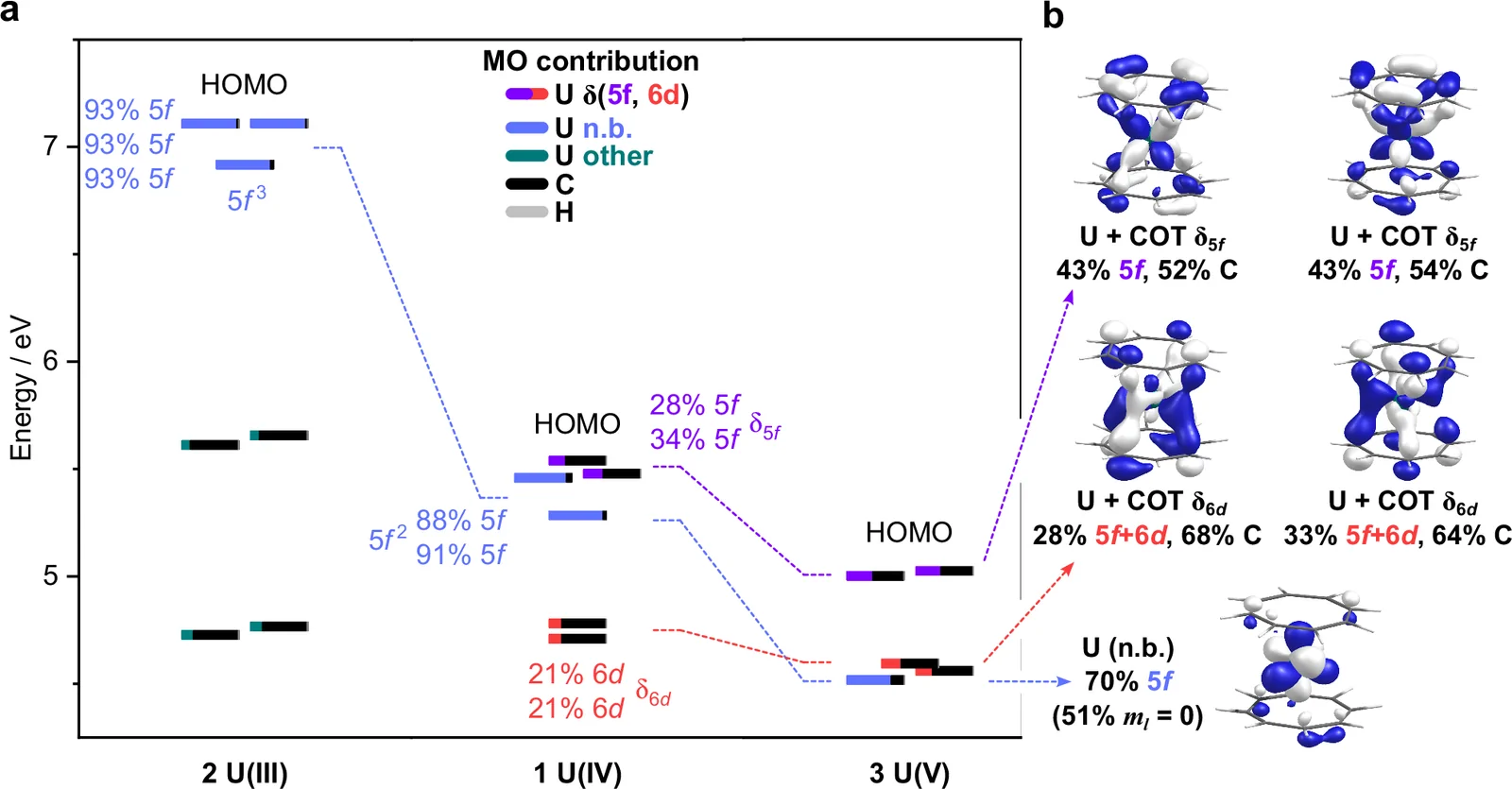
Frontier α-spin molecular orbital compositions and visualisations. **a** A partial molecular orbital diagram for complexes **1**–**3**, in order of increasing formal uranium oxidation state, showing the evolution of orbital composition and energy from U(III) **2**, to U(IV) **1**, and U(V) **3**. MO energies scaled with the totally symmetric σ-bonding orbital (${a}_{1g}$ under *D*_8h_ symmetry) at 0 eV (n.b. = non-bonding). **b** Frontier Kohn-Sham molecular orbital compositions for complex **3** with isosurface values set to 0.05 e Å^–3^ (see *Supplementary Information* for all others).

In actinocenes, δ-bonding arises from mixing between the {COT_2_} ${e}_{2g}$ and ${e}_{2u}$ sets and metal ${d}_{{x}^{2}-{y}^{2}/{xy}}$ and ${f}_{{z({x}^{2}-{y}^{2})}/{xyz}}$ orbitals (using *D*_8h_ symmetry terms)^13,15–22^. The frontier molecular orbitals (MOs) in U(IV) **1** reveal two δ-bonding manifolds with significant 5 *f* (28.1 and 33.7%) or 6 *d* (21.3 and 21.2%) character, as in prior works^20–22^. In U(III) **2**, these bonding orbitals have lower metal 5 *f* (14.5 and 14.4%) and 6 *d* (19.8 and 19.6%) involvement due to the decreased effective nuclear charge and longer metal–carbon bond lengths. Upon oxidation to U(V) in **3**, the 5 *f* (42.7 and 43.0%) and 6 *d* (32.8 and 28.0%) involvement in δ-bonding increases significantly, which is shown graphically in Fig. 4. In substituted **4,** **5**, and **6**, 6 *d* involvement in δ-bonding is similar across all three oxidation states (17.0–18.9%) but, as with **1**–**3**, the 5*f*-based δ-bonding contributions increase from U(III) **5** (13.3 and 14.0%), to U(IV) **4** (25.2 and 33.5%), then U(V) **6** (41.3 and 42.9%).

Analysis of the MO compositions within the natural localised molecular orbital (NLMO) framework revealed qualitatively similar results to the Kohn-Sham orbital analysis above. The uranium contribution to the 5 *f* and 6*d*-derived δ-symmetry orbitals increases markedly from U(III) **2**, where no bonding interaction is recovered, to U(IV) **1** [35% U (100% 5 *f*) and 18% U (99% 6 *d*, 1% 5 *f*)], and U(V) **3** [46% U (89% 5 *f*, 11% 6 *d*) and 41% U (81% 5 *f*, 19% 6 *d*)]. The results for **4**–**6** are similar (see Supplementary Tables S52–S56).

Topological analysis of the electron density at the U–C bond critical points (BCPs) in complexes **1**–**6** characterises the bonding in **3** (and **6**) as more covalent than in **1** or **2** (and **4** or **5**). The uranocenium complexes show significant electron and energy density increases at the U–C BCPs over their neutral and anionic counterparts, and the delocalisation indices [δ(U,C)] are approximately 20% greater in **3** and **6** than in **1** and **4**. The ratio of kinetic (G) and potential ( | V | ) energy density is <1 for all complexes, indicating polar-covalent bonding, but **3** and **6** show the largest deviation and have a greater proportion of covalent character than the others.

An energy decomposition analysis was performed to gain insight into the nature of the metal−ligand interactions in **1**–**6**. The total interaction energy ($\Delta {E}_{{{\mathrm{int}}}}$) and the individual contributions to the total are given by (**1**) & (**2**), where COT^R^ is either COT, or COT^††^, and *n* is the charge on the fragment required to balance the formal uranium oxidation state.

1 $$\Delta E_{{\mathrm{int}}}=E_{{\mathrm{complex}}} - E\!\left(\left\{{\mathrm{U}}^{{\mathrm{III/IV/V}}}\left({\mathrm{COT}}^{{\mathrm{R}}}\right)^{n+}\right\}+\left\{{\mathrm{COT}}^{{\mathrm{R}}}\right\}^{2-}\right)$$

2 $$\Delta {E}_{{{\mathrm{int}}}}=\Delta {E}_{{Pauli}}+\Delta {E}_{{Orb}}+\Delta {E}_{{elstat}}$$

These contributions reflect the stabilising effect of bringing ligand anions into proximity with metal cations ($\Delta {E}_{{elstat}}$), the repulsive penalty paid upon bringing ligand and metal fragment orbitals into proximity prior to allowing them to mix ($\Delta {E}_{{Pauli}}$), and finally the stabilisation gained upon orbital mixing ($\Delta {E}_{{Orb}}$) which reflects the covalent part of the interaction. The total interaction in complex **3** is stabilised versus **1** ($\Delta \Delta {E}_{{{\mathrm{int}}}}$ = –1,068 kJ mol^–1^), mainly due to a larger $\Delta {E}_{{elstat}}$ contribution from the larger charge at the U(V) centre in **3** versus U(IV) in **1** ($\Delta \Delta {E}_{{Elstat}}\,$= –697 kJ mol^–1^), and also larger total orbital contributions in **3** ($\Delta \Delta {E}_{{Orb}}$ = –452 kJ mol^–1^) from increased mixing between metal and ligand orbitals. Conversely, **3** shows a slight increase in Pauli repulsion ($\Delta \Delta {E}_{{Pauli}}\,$= +84 kJ mol^–1^). Relaxation of the fragment orbitals into the δ-bonding complex orbitals requires minimising electron–electron repulsion, thereby increasing their spatial extent; nevertheless, expelling electron density from the internuclear region is more costly in **3** due to the higher metal charge than in **1**.

Oxidation to U(V) in **3** enhances uranium–COT orbital mixing, while the increased metal charge would be expected to cause a larger decrease in the U–C distances than is seen experimentally. However, the strongly mixed and highly angular nature of the δ-bonding orbitals in **3** means that further bond length contraction comes at the cost of moving electron density out of the bonding region. Extended transition state - natural orbitals for chemical valence (ETS-NOCV) data for **3** confirms the energetic results above do not arise simply from internal polarisation within the fragments (Supplementary Fig. S247 and Supplementary Table S70). In the case of the U(III) complex **2**, polarisation of the non-bonding 5 *f* electrons is clearly visible in the NOCV α-channel. Conversely, with U(IV) **1**, and U(V) **3**, there is substantial rearrangement from the ligand fragment into δ-symmetry bonding orbitals, which reflects the stronger covalent contribution to bonding in these complexes.

As with multiply-bonded actinide complexes^55^, the increase in uranium *f*-orbital participation directs the structural change, as *d*-orbital contributions do not change substantially between **4** and **6**, but nevertheless display the same solution and solid-state structural behaviour as **1** and **3**. Similar repulsive effects govern carbon–halogen bonding in methyl halides^56^, have been invoked to explain bonding in F_2_ (ref. ^57^), but do not apply to the ionic bonding in lanthanide {M^III^(COT)_2_}^n–^ complexes.

## Discussion

Isolation of the parent uranocenium cation, [U^V^(COT)_2_]^+^, alongside a substituted analogue [U^V^(COT^††^)_2_]^+^, establishes two structurally authenticated U(III)/U(IV)/U(V) actinocene series where electronic-structure changes can be tracked in nearly invariant ligand environments across multiple oxidation states. Despite increased metal–ligand covalency, structural changes between U(IV) uranocene and U(V) uranocenium complexes are minimal due to counterbalancing energetic components to the bonding. The minimised structural change grants the uranocene/uranocenium redox couple electrochemical reversibility comparable to that of the canonical outer-sphere redox pair ferrocene/ferrocenium. Uranocene was first named for its resemblance to an *f*-block analogue of ferrocene. Now, more than fifty years later, this analogy is cemented by the parallel between their one-electron oxidised counterparts, the ferrocenium and uranocenium cations. These results open the way to well-defined actinocene/actinocenium redox chemistry and provide a platform for interrogating 5 *f*/6 *d* covalency across multiple oxidation states with minimal geometric distortion.

## Methods

### General details and synthesis

***Caution!*** Natural-abundance uranium (assumed standard composition: 0.7204% ^235^U, *t*_1/2_ = 7.04×10^8^ years; 99.2742% ^238^U, *t*_1/2_ = 4.468×10^9^ years) is a weak α-emitter (4.198 and 4.151 MeV from ^238^U). Along with α-, β-, and γ-emitting decay products, this material represents both radiotoxicity (α-particles) and heavy-metal health hazards. All manipulations were performed in continuous extraction fume cupboards or positive pressure inert atmosphere gloveboxes located in a dedicated laboratory with contamination monitoring protocols, along with training and materials for decontamination equipment.

Unless otherwise described, all syntheses and manipulations were conducted under BOC PureShield argon (99.995%) with rigorous exclusion of oxygen and water using Schlenk line and glove box techniques in a Glovebox Systemtechnik MEGA. See the *Supplementary Information* for the purification and drying of solvents, precursor synthesis, and synthesis of **1** and **4**.

### Synthesis of [Li(12-crown-4)(THF)][U^III^(COT)_2_] (2)

THF (15 mL) was added to a pre-cooled (–98 °C) Schlenk vessel containing a mixture of **1** (0.446 g, 1.0 mmol, 1 equiv.), Li^0^ metal (0.008 g, 1.2 mmol, 1.2 equiv.) and naphthalene (0.002 g, 0.016 mmol) and a glass-coated stirrer bar. The mixture was stirred for two days to give a dark brown solution. The supernatant was filtered through a glass microfiber filter disc into a new Schlenk vessel, and the volatiles were removed under vacuum (10^–3^ mbar), which gave putative [Li(THF)_4_][U^III^(COT)_2_] (0.153 g, crude yield), which was used without further characterisation. THF (10 mL) was added to a glass vial containing putative [Li(THF)_4_][U^III^(COT)_2_] (0.153 g, 0.21 mmol, 1 equiv.) and a PTFE-coated stirrer bar. 12-crown-4 (0.036 g, 0.21 mmol, 1 equiv.) was added to the brown solution and stirred briefly. Storage at –35 °C gave a single crystalline crop of **2**. The product was isolated by decanting the supernatant while cold, followed by drying the crystals under vacuum (10^–3^ mbar) for 4 h to give **2** in low overall isolated crystalline yield (0.140 g, 20%).

Elemental analysis on C_28_H_40_LiO_5_U calc. (%): C = 47.93, H = 5.75, *N* = 0.00; found (%): C = 48.26, H = 5.56, *N* = 0.00.

^1^H NMR (*d*_8_-THF, 400.07 MHz, 298 K): δ = 6.71 (br. s, $\bar{\nu }$ = 191.2 Hz, 16H, 12-crown-4 C*H*_2_), −31.18 (br. s, $\bar{\nu }$ = 194.3 Hz, 16H, COT C*H*) ppm.

^13^C{^1^H} NMR (*d*_8_-THF, 100.61 MHz, 298 K): δ = no resonances observed.

UV-Vis-NIR (THF, 0.55 mM): λ_max_ (ν/cm^–1^; ε/M^–1^cm^–1^) = 1,456 (6,868; 280), 1,386 (7,215; 295), 1,318 (7,587; 248), 389 (25,707; 1,591).

FT-IR (ATR, microcrystalline): cm^–1^ = 477 (s), 508 (m), 543 (w), 559 (s), 592 (vw), 604 (vw), 678 (vs), 701 (m), 738 (m), 756 (vw), 859 (s), 890 (vs), 927 (s), 1,017 (s), 1,036 (s), 1,079 (vs), 1,130 (m), 1,241 (w), 1,282 (w), 1,297 (vw), 1,447 (w), 2,877 (w), 2,929 (vw), 2,953 (vw), 3,005 (m).

Repeat synthesis gave crystals of **2**^**β**^, which differs in the unit cell but not structure or composition (see *Supplementary Information*).

### Synthesis of [U^V^(COT)_2_][B(C_6_F_5_)_4_] (3)

*o*DFB (10 mL) was added to a pre-cooled (–35 °C) Schlenk vessel containing a mixture of **1** (0.260 g, 0.58 mmol, 1 equiv.) and [Ph_3_C][B(C_6_F_5_)_4_] (0.535 g, 0.58 mmol, 1 equiv.) and a PTFE-coated stirrer bar. The mixture was allowed to warm to room temperature and stirred overnight (18 hours) to give a dark brown solution. The supernatant was filtered through a glass microfiber filter disc into a new Schlenk vessel, and the volatiles were removed under vacuum (10^–3^ mbar) which gave a dark brown solid. The solids were washed with *n*-hexane (10 mL), and the volatiles were removed under vacuum (10^–3^ mbar). *o*DFB (2 mL) was added and the dark brown solution and layered with *n*-hexane (10 mL). Storage at room temperature gave a single crystalline crop of **3**. The product was isolated by decanting the supernatant while cold, and the crystals were dried under vacuum (10^–3^ mbar) for 4 h to give **3** in fair isolated crystalline yield (0.315 g, 48%).

Elemental analysis on C_40_H_16_BF_20_U·(C_6_H_4_F_2_)_n_ for *n* = 1 calc. (%): C = 44.57, H = 1.63, *N* = 0.00; for *n* = 0 calc. (%): C = 42.69, H = 1.43, *N* = 0.00; found (%): C = 43.65, H = 1.54, *N* = 0.00. *Note*, the *o*DFB is present in the crystal structure of **3**, and elemental analysis results show incomplete desolvation under vacuum.

^1^H NMR (*d*_5_-bromobenzene, 400.07 MHz, 298 K): δ = 6.7 (m, *o*DFB C*H*), −21.46 (br. s, $\bar{\nu }$ = 112.6 Hz, COT C*H*) ppm.

^11^B{^1^H} NMR (*d*_5_-bromobenzene, 128.36 MHz, 298 K): δ = −15.92 (s, B(C_6_F_5_)_4_ *B*) ppm.

^13^C{^1^H} NMR (*d*_5_-bromobenzene, 100.61 MHz, 298 K): δ = no resonances observed.

^19^F{^1^H} NMR (*d*_5_-bromobenzene, 376.50 MHz, 298 K): δ = −129.70 (s, B(C_6_F_5_)_4_ *F*), −137.25 (s, *o*DFB), −160.71 (m, B(C_6_F_5_)_4_ *F*), −164.10 (m, B(C_6_F_5_)_4_ *F*) ppm.

UV-Vis-NIR (THF, 0.35 mM): λ_max_ (ν/cm^–1^; ε/M^–1^cm^–1^) = 700 (14,285; 1,039), 492 (20,325; 2,436), 448 (22,321; 2,717), 416 (24,038; 2,096), 376 (26,596; 2,972), 336 (29,762; 8,130), 326 (30,675; 7,601).

FT-IR (ATR, microcrystalline): cm^–1^ = 413 (m), 438 (w), 450 (m), 475 (m), 491 (w), 501 (w), 516 (w), 524 (vw), 534 (w), 547 (m), 573 (s), 602 (m), 610 (m), 625 (w), 660 (vs), 682 (s), 717 (vs), 732 (s), 756 (vs), 775 (s), 806 (w), 843 (w), 857 (vw), 904 (s), 976 (vs), 1,030 (vw), 1,081 (vs), 1,192 (vw), 1,266 (s), 1,324 (vw), 1,338 (vw), 1,375 (m), 1,412 (s), 1,455 (vs), 1,509 (s), 1,640 (m), 2,945 (vw), 3,062 (vw), 3,619 (vw), 3,627 (vw).

### Synthesis of [Li(TMEDA)_2_][U^III^(COT^††^)_2_] (5)

THF (15 mL) was added to a Schlenk vessel containing a mixture of **4** (0.918 g, 0.86 mmol, 1 equiv.) and Li^0^ metal (0.007 g, 1.0 mmol, 1.2 equiv.) and a glass-coated stirrer bar. The mixture was stirred for five days to give a dark brown solution. The supernatant was filtered through a glass microfiber filter disc into a new Schlenk vessel, and the volatiles were removed under vacuum (10^–3^ mbar), which gave a dark brown solid. TMEDA (2 mL) was added, and the mixture was stored at –35 °C, yielding large brown crystals. The crude product was isolated by decanting the supernatant while cold, the solid was washed with *n*-hexane (10 mL) and the crystals were dried under vacuum (10^–3^ mbar) for 4 h. Mesitylene (2 mL) was added, briefly heated and storage at –35 °C gave a single crystalline crop of **5**. The product was isolated by decanting the supernatant while cold, and the crystals were washed with *n*-hexane (5 mL) and dried under vacuum (10^–3^ mbar) for 4 h to give **5** in low isolated crystalline yield (0.323 g, 26%).

Elemental analysis on C_64_H_128_Si_4_N_4_LiU·(C_9_H_12_) calc. (%): C = 61.26, H = 9.86, *N* = 3.91; found (%): C = 61.24, H = 10.00, *N* = 3.62. *Note*, the mesitylene is present in the crystal structure of **5**, and elemental analysis results show incomplete desolvation under vacuum.

^1^H NMR (d_8_-THF, 400.07 MHz, 298 K): δ = 6.73 (s, 5H, mesitylene C*H*), 2.30 (s, 10H, TMEDA C*H*_2_), 2.21 (s, 15H, mesitylene C*H*_3_), 2.15 (s, 30H, TMEDA C*H*_3_), −0.24 (br. s, 42H, TIPS C*H*/C*H*_3_), −0.45 (br. s, 42H, TIPS C*H*/C*H*_3_), −1.22 (br. s, 12H, $\bar{\nu }$ = 64.4 Hz, COT C*H*) ppm.

^13^C{^1^H} NMR (d_8_-THF, 100.61 MHz, 298 K): δ = 138.23 (s, mesitylene *C*(CH_3_)), 127.73 (s, mesitylene *C*H), 59.08 (s, TMEDA *C*H_2_), 46.36 (s, TMEDA *C*H_3_), 21.46 (s, mesitylene *C*H_3_) ppm.

^29^Si{^1^H} NMR (d_8_-THF, 79.47 MHz, 298 K): δ = no resonances observed.

UV-Vis-NIR (THF, 0.25 mM): λ_max_ (ν/cm^–1^; ε/M^–1^cm^–1^) = 1,740 (5,747; 225), 1,563 (6,398; 274), 1,467 (6,815; 257), 1,428 (7,001; 241), 1,265 (7,908; 333), 1,234 (8,103; 381), 1,189 (8,413; 337), 637 (15,701; 91), 617 (16,213; 208), 592 (16,878; 630), 402 (24,894; 2,397).

FT-IR (ATR, microcrystalline): cm^–1^ = 419 (m), 438 (w), 446 (m), 469 (w), 491 (w), 508 (w), 518 (vw), 575 (vw), 604 (w), 637 (vw), 658 (vw), 688 (m), 721 (vw), 750 (s), 773 (s), 787 (m), 836 (w), 882 (vw), 929 (vw), 945 (m), 962 (m), 989 (m), 1,013 (vw), 1,034 (vw), 1,071 (s), 1,097 (vs), 1,128 (s), 1,159 (s), 1,184 (vs), 1,208 (s), 1,248 (m), 1,289 (m), 1,361 (s), 1,379 (s), 1,412 (vs), 1,459 (vw), 1,607 (s), 2,709 (vs), 2,717 (vs), 2,797 (s), 2,857 (vw), 2,886 (w), 2,916 (w), 2,937 (w), 3,025 (vs).

### Synthesis of [U^V^(COT^††^)_2_][B(C_6_F_5_)_4_] (6)

*o*DFB (10 mL) was added to a pre-cooled (–35 °C) Schlenk vessel containing a mixture of **4** (0.921 g, 0.86 mmol, 1 equiv.) and [Ph_3_C][B(C_6_F_5_)_4_] (0.799 g, 0.86 mmol, 1 equiv.) and a PTFE-coated stirrer bar. The mixture was allowed to warm to room temperature and stirred overnight (18 h) to give a dark red solution. The supernatant was filtered through a glass microfiber filter disc into a new Schlenk vessel, and the volatiles were removed under vacuum (10^–3^ mbar), which gave a dark red solid. The solids were washed with *n*-hexane (10 mL), and the volatiles were removed under vacuum (10^–3^ mbar). *o*DFB (2 mL) was added, and the dark brown solution was layered with *n*-hexane (10 mL). Storage at room temperature gave a single crystalline crop of **6**. The product was isolated by decanting the supernatant while cold, and the crystals were dried under vacuum (10^–3^ mbar) for 4 h to give **6** in low isolated crystalline yield (0.578 g, 38%).

Elemental analysis on C_76_H_96_Si_4_F_20_BU calc. (%): C = 52.14, H = 5.53, *N* = 0.00; found (%): C = 51.26, H = 5.54, *N* = 0.00.

^1^H NMR (*d*_5_-bromobenzene, 400.07 MHz, 298 K): δ = 6.16 (br. s, $\bar{\nu }$ = 87.6 Hz, 12H, COT C*H*), 3.12 (m, 84H, TIPS C*H*/C*H*_3_) ppm.

^11^B{^1^H} NMR (*d*_5_-bromobenzene, 128.36 MHz, 298 K): δ = −15.50 (s, B(C_6_F_5_)_4_ *B*) ppm.

^13^C{^1^H} NMR (*d*_5_-bromobenzene, 100.61 MHz, 298 K): δ = no resonances observed.

^19^F{^1^H} NMR (*d*_5_-bromobenzene, 376.50 MHz, 298 K): δ = −130.96 (s, B(C_6_F_5_)_4_ *F*), −161.67 (t, *J*_FF_ = 21.0 Hz, B(C_6_F_5_) *F*), −165.32 (m, B(C_6_F_5_)_4_ *F*) ppm.

^29^Si{^1^H} NMR (*d*_5_-bromobenzene, 79.47 MHz, 298 K): δ = −97.73 (s, TIPS *Si*) ppm.

UV-Vis-NIR (*o*DFB, 0.28 mM): λ_max_ (ν/cm^–1^; ε/M^–1^cm^–1^) = 784 (12,755; 793), 496 (20,161; 2,715), 396 (25,253; 5,163), 324 (30,864; 4,231).

FT-IR (ATR, microcrystalline): cm^–1^ = 411 (s), 421 (m), 438 (m), 448 (m), 475 (w), 491 (s), 522 (w), 543 (m), 573 (vw), 600 (w), 610 (w), 639 (vw), 660 (vw), 678 (vw), 707 (vs), 725 (vs), 756 (vw), 769 (w), 816 (s), 880 (vw), 921 (m), 937 (m), 978 (vw), 1013 (w), 1083 (vw), 1192 (vs), 1225 (s), 1274 (m), 1315 (vs), 1340 (vs), 1373 (s), 1383 (m), 1412 (s), 1459 (vw), 1513 (vw), 1642 (s), 2721 (vs), 2869 (w), 2890 (s), 2947 (w).

### Cyclic voltammetry

Electrochemical data were measured using an Ivium CompactStat.h20250. Data were collected using IviumSoft 4.1170 software in Advanced Mode. Measurements were performed using a three-electrode cell in an inert atmosphere glovebox under an atmosphere of argon with a glassy carbon working electrode (3 mm diameter, BASi MF-2012), a platinum wire counter electrode (7.5 cm length, BASi MW-1032), and a platinum wire auxiliary reference electrode (7.5 cm length, BASi MW-1032) at ambient temperature.

The working electrode surface was polished prior to each set of experiments and cleaned between each scan. This was done to prevent the buildup of oxidised product on the electrode surfaces. All data were collected in a positive-feedback IR compensation mode. Prior to each scan, the open-circuit potential (OCP) and internal resistance (*i*R) were each recorded for 10 seconds, and the mean was used to calibrate each experiment. Measurements were performed using 0.2 M electrolyte concentration and 3.0 mM analyte concentration. All data are internally referenced to the [Fc]^+/0^ couple by adding *ca*. 1 mg of Fc (*ca*. 3 mM) to solutions at the end of the experiment collection, unless otherwise stated. Small deviations in reported *E*_1/2_ values between scans arise from the 20 mV step used in the experiment. While internal referencing to the [Fc*]^+/0^ couple is preferred due to the somewhat improved electrochemical characteristics compared to that of Fc^58,59^, the [Fc*]^+/0^ couple overlaps with data for some of the complexes herein. Furthermore, bulk samples of **3** and **6** react with Fc*, to give an intractable mixture of products, and so its use as an internal reference would be inappropriate.

### Electronic structure calculations

Unrestricted Kohn-Sham density functional theory (DFT) calculations were performed at the scalar-relativistic (SR) DFT level. Where appropriate, only the anion or cation was considered due to the separated nature of the anion and cation parts of the relevant complexes. Coordinate sets were calculated in the gas phase where only H-atom positions were optimised, all others atom positions were held at the coordinates determined by SCXRD. These calculations were performed with the ORCA 6.1.0 quantum chemistry program suite^60^. The PBE0^53,54^ hybrid functional was used with the D3-BJ dispersion correction^61^, a tight integration grid (DefGrid3), and the resolution of the identity ‘chain of spheres’ (RIJCOSX) approximation^62,63^. Scalar relativistic effects were accounted for using the exact two-component (X2C) Hamiltonian, along with the diagonal local approximation to the unitary transformation matrix (DLU-X2C) to decrease computational cost^64^. Segmented contracted all-electron error-consistent basis sets of triple‑ζ valence quality were used for all atoms as follows: H, C, Si (X2C-SVPall for optimisations, X2C-TZVPall for properties)^65,66^. For U, the cc-pVDZ-X2C basis set was used throughout^67–69^, with automatic generation of auxiliary basis sets^70^. Picture-change effects were accounted for, and the full relativistic Hamiltonian derivative was calculated (PictureChange 2). Where appropriate, the optimised structures were confirmed to be true minima by analytical vibrational frequency calculations.

Further gas-phase single-point calculations were performed in ADF2025.102 (ref. ^71^) to perform energy decomposition (EDA) and extended transition state natural orbitals for chemical valence (ETS-NOCV) analyses for the M + COT interactions as {M(COT^R^)}^n+^ + {(COT^R^)} (refs. ^72–75^). These calculations employed the PBE0 functional, with D3-BJ dispersion correction^61,76^ and a Slater-type doubly-polarised triple-ζ basis set (TZ2P) on U atoms, and a triple-ζ basis set (TZP) on H, C, Si atoms – all electrons were explicitly treated^77^. The ZORA (Zeroth Order Regular Approximation) scalar relativistic Hamiltonian was used throughout^78,79^. The VeryGood BeckeGrid was employed with a RadialGridBoost (level 3) applied to avoid linear dependencies.

Electron density-based analysis of metal–ligand bonding used the AIMAll software package^80^ using WFX files generated from the DFT calculations (in ORCA 6.1.0 using the orca_2aim module). The default settings were used to complete the electron density topology and integration of atomic basins. The NBO (7.0) software package was used to compute NLMOs to analyse bonding orbitals^81^.

## Supplementary information

Supplementary Information

Transparent Peer Review file

## Data Availability

Crystallographic data are available free of charge from the Cambridge Crystallographic Data Centre under CCDC 2481660, 2481662–2481664, 2481666, 2503922–2503925, 2521131, and 2522902. Raw instrument and calculation outputs generated in this study are available at FigShare (DOI:10.48420/31025713). All other data are available in the *Supplementary Information*, and all data are available from the corresponding authors upon request.

## References

[1] Miller, S. A., Tebboth, J. A. & Tremaine, J. F. Dicyclopentadienyliron. *J. Chem. Soc.* **114**, 632–635 (1952).

[2] Wilkinson, G., Rosenblum, M., Whiting, M. C. & Woodward, R. B. The structure of iron bis-cyclopentadienyl. *J. Am. Chem. Soc.* **74**, 2125–2126 (1952).

[3] Woodward, R. B., Rosenblum, M. & Whiting, M. C. A new aromatic system. *J. Am. Chem. Soc.* **74**, 3458–3459 (1952).

[4] Dunitz, J. D. & Orgel, L. E. Bis-cyclopentadienyl Iron: a molecular sandwich. *Nature* **171**, 121–122 (1953).

[5] Dunitz, J. D., Orgel, L. E. & Rich, A. The crystal structure of ferrocene. *Acta Crystallogr.* **9**, 373–375 (1956).

[6] Togni, A. & Halterman, R. L. *Metallocenes*: *Synthesis Reactivity Applications* (Wiley, 1998).

[7] Long, N. J. *Metallocenes*: *An Introduction to Sandwich Complexes* (Blackwell Science, 1998).

[8] Guo, F. S. et al. Isolation of a perfectly linear uranium(II) metallocene. *Angew. Chem., Int. Ed.* **59**, 2299–2303 (2020). 10.1002/anie.201912663 31710765

[9] Streitwieser, A. Jr. & Mueller-Westerhoff, U. Bis(cyclooctatetraenyl)uranium (Uranocene). A new class of sandwich complexes that utilize atomic f orbitals. *J. Am. Chem. Soc.* **90**, 7364–7364 (1968).

[10] Zalkin, A. & Raymond, K. N. Structure of Di-π-cyclooctatetraeneuranium (Uranocene). *J. Am. Chem. Soc.* **91**, 5667–5668 (1969).

[11] Streitwieser, A. Jr. et al. Preparation and properties of uranocene, Di-π-cyclooctatetraeneuranium(IV). *J. Am. Chem. Soc.* **95**, 8644–8649 (1973).

[12] Smiles, D. E. et al. The duality of electron localization and covalency in lanthanide and actinide metallocenes. *Chem. Sci.* **11**, 2796–2809 (2020). 10.1039/c9sc06114b PMC8157540 34084340

[13] Minasian, S. G. et al. New evidence for 5f covalency in actinocenes determined from carbon K-edge XAS and electronic structure theory. *Chem. Sci.* **5**, 351–359 (2014).

[14] Karraker, D. G., Stone, J. A., Jones, E. R. & Edelstein, N. Bis(cyclooctatetraenyl)neptunium(IV) and Bis(cyclooctatetraenyl)plutonium(IV). *J. Am. Chem. Soc.* **92**, 4841–4845 (1970).

[15] Chang, A. H. H. & Pitzer, R. M. Electronic structure and spectra of uranocene. *J. Am. Chem. Soc.* **111**, 2500–2507 (1989).

[16] Brennan, J. G., Green, J. C. & Redfern, C. M. Covalency in bis([8]annulene)uranium from photoelectron spectroscopy with variable photon energy. *J. Am. Chem. Soc.* **111**, 2373–2377 (1989).

[17] Clark, J. P. & Green, J. C. An investigation of the electronic structure of bis(*η*-cyclo-octatetraene)-actinoids by helium-(I) and -(II) photoelectron spectroscopy. *J. Chem. Soc. Dalton Trans*. **6**, 505–508 (1977).

[18] P Clark, J. & C Green, J. The He-I photoelectron spectra of uranocene and thorocene. *J. Organomet. Chem.* **112**, C14–C16 (1976).

[19] Raymond, K. N. & Eigenbrot, C. W. Jr. Structural criteria for the mode of bonding of organoactinides and -lanthanides and related compounds. *Acc. Chem. Res.* **13**, 276–283 (1980).

[20] Fragala, I., Condorelli, G., Zanella, P. & Tondello, E. Photoelectron spectroscopy of actinide organometallic compounds I. Bis(cyclooctatetraene)actinide(IV) complexes. *J. Organomet. Chem.* **122**, 357–363 (1976).

[21] Roesch, N. & Streitwieser, A. Jr. Quasirelativistic SCF-Xα scattered-wave study of uranocene, thorocene, and cerocene. *J. Am. Chem. Soc.* **105**, 7237–7240 (1983).

[22] Hayes, R. G. & Edelstein, N. Elementary molecular orbital calculation on U(C_8_H_8_)_2_ and its application to the electronic structures of U(C_8_H_8_)_2_, Np(C_8_H_8_)_2_, and Pu(C_8_H_8_)_2_. *J. Am. Chem. Soc.* **94**, 8688–8691 (1972).

[23] Butcher, J. A. Jr., Chambers, J. Q. & Pagni, R. M. Anodic oxidation of uranocene. Evidence for new organouranium cations in solution. *J. Am. Chem. Soc.* **100**, 1012–1013 (1978).

[24] Butcher, J. A., Pagni, R. M. & Chambers, J. Q. The anodic oxidation of uranocene. Evidence for a reactive dication. *J. Organomet. Chem.* **199**, 223–227 (1980).

[25] Solis-Céspedes, E. & Páez-Hernández, D. Modeling the electronic states and magnetic properties derived from the f^1^ configuration in lanthanocene and actinocene compounds. *Dalton Trans.* **46**, 4834–4843 (2017). 10.1039/c7dt00111h 28349155

[26] Bedford, R. G. Sublimation of bis(η^8^−1,3,5,7-cyclooctatetraene)uranium. *J. Phys. Chem.* **81**, 1284–1289 (1977).

[27] Marks, J. H., Batchelor, A. G., Blais, J. R. C. & Duncan, M. A. Cation complexes of uranium and thorium with cyclooctatetraene: photochemistry and decomposition products. *J. Phys. Chem. A* **126**, 4230–4240 (2022). 10.1021/acs.jpca.2c03035 35749286

[28] Avdeef, A., Raymond, K. N., Hodgson, K. O. & Zalkin, A. Two isostructural actinide π Complexes. Crystal and molecular structure of Bis(cyclooctatetraenyl)uranium(IV), U(C_8_H_8_)_2_, and Bis(cyclooctatetraenyl)thorium(IV), Th(C_8_H_8_)_2_. *Inorg. Che*. *m* **11**, 1083–1088 (1972).

[29] Streitwieser, A. Jr. & Yoshida, N. Di-π-cyclooctatetraenethorium. *J. Am. Chem. Soc.* **91**, 7528–7528 (1969).

[30] De Ridder, D. J. A., Rebizant, J., Apostolidis, C., Kanellakopulos, B. & Dornberger, E. Bis(cyclooctatetraenyl)neptunium(IV). *Acta Crystallogr., Sect. C.* **52**, 597–600 (1996).

[31] Windorff, C. J. et al. A single small-scale plutonium redox reaction system yields three crystallographically-characterizable organoplutonium complexes. *Inorg. Chem.* **59**, 13301–13314 (2020). 10.1021/acs.inorgchem.0c01671 32910649

[32] Russo, D. R. et al. Berkelium–carbon bonding in a tetravalent berkelocene. *Science* **387**, 974–978 (2025). 10.1126/science.adr3346 40014698

[33] Connelly, N. G. & Geiger, W. E. Chemical Redox Agents for Organometallic Chemistry. *Chem. Rev.* **96**, 877–910 (1996). 10.1021/cr940053x 11848774

[34] Kuppuswamy, S. et al. One-electron oxidation chemistry and subsequent reactivity of diiron imido complexes. *Inorg. Chem.* **53**, 5429–5437 (2014). 10.1021/ic403039x 24833117

[35] Jordan, R. F., Dasher, W. E. & Echols, S. F. Reactive cationic dicyclopentadienyl zirconium(IV) complexes. *J. Am. Chem. Soc.* **108**, 1718–1719 (1986).

[36] Tarifa, L., Cano-Asensio, J., López, J. A., Tejel, C. & Geer, A. M. Cooperative anion activation at a cobalt center through ion pairing and ligand design. *Nat. Commun.* **17**, 2469 (2026). 10.1038/s41467-026-69257-8 PMC12992617 41654490

[37] Thomas, B. J., Mitchell, J. F., Theopold, K. H. & Leafy, J. A. Synthesis and structure determination of [Cp*_4_Cr_4_(μ-F)_5_Cl_2_]PF_6_. A tetranuclear chromium complex formed by fluoride abstraction from hexafluorophosphate. *J. Organomet. Chem.* **348**, 333–342 (1988).

[38] Blackmore, K. J., Sly, M. B., Haneline, M. R., Ziller, J. W. & Heyduk, A. F. Group IV imino-semiquinone complexes obtained by oxidative addition of halogens. *Inorg. Chem.* **47**, 10522–10532 (2008). 10.1021/ic801127r 18937444

[39] Shannon, R. D. Revised effective ionic radii and systematic studies of interatomic distances in halides and chalcogenides. *Acta Crystallogr., Sect. A* **32**, 751–767 (1976).

[40] Orlova, A. P. et al. Molecular network approach to anisotropic Ising lattices: parsing magnetization dynamics in Er^3+^ systems with 0–3-dimensional spin interactivity. *J. Am. Chem. Soc.* **145**, 22265–22275 (2023). 10.1021/jacs.3c08946 PMC10571078 37774116

[41] Harriman, K. L. M., Korobkov, I. & Murugesu, M. From a piano stool to a sandwich: a stepwise route for improving the slow magnetic relaxation properties of thulium. *Organometallics* **36**, 4515–4518 (2017).

[42] Gourier, D., Caurant, D., Arliguie, T. & Ephritikhine, M. EPR and angle-selected ENDOR study of 5f-ligand interactions in the [U(η^7^-C_7_H_7_)_2_]^–^ Anion, an f^1^ analogue of uranocene. *J. Am. Chem. Soc.* **120**, 6084–6092 (1998).

[43] Walter, M. D., Booth, C. H., Lukens, W. W. & Andersen, R. A. Cerocene revisited: the electronic structure of and interconversion between Ce_2_(C_8_H_8_)_3_ and Ce(C_8_H_8_)_2_. *Organometallics* **28**, 698–707 (2009).

[44] Singh, S. K., Cramer, C. J. & Gagliardi, L. Correlating electronic structure and magnetic anisotropy in actinide complexes [An(COT)_2_], An^III/IV^ = U, Np, and Pu. *Inorg. Chem.* **59**, 6815–6825 (2020). 10.1021/acs.inorgchem.0c00105 32368906

[45] Gendron, F., Pritchard, B., Bolvin, H. & Autschbach, J. Single-ion 4f element magnetism: an ab-initio look at Ln(COT)_2_^−^. *Dalton Trans.* **44**, 19886–19900 (2015). 10.1039/c5dt02858b 26510902

[46] Li, J. & Bursten, B. E. Electronic structure of cycloheptatrienyl sandwich compounds of actinides: An(η^7^-C_7_H_7_)_2_ (An=Th, Pa, U, Np, Pu, Am). *J. Am. Chem. Soc.* **119**, 9021–9032 (1997).

[47] Sergentu, D.-C., Gendron, F. & Autschbach, J. Similar ligand–metal bonding for transition metals and actinides? 5f^1^ U(C_7_H_7_)^2−^*versus* 3d^n^ metallocenes. *Chem. Sci.* **9**, 6292–6306 (2018). 10.1039/c7sc05373h PMC6063092 30123484

[48] Qiao, Y. et al. Enhanced 5f-δ bonding in [U(C_7_H_7_)_2_]^−^: C K-edge XAS, magnetism, and ab initio calculations. *Chem. Commun.* **57**, 9562–9565 (2021). 10.1039/d1cc03414f 34546232

[49] Kerridge, A. f-Orbital covalency in the actinocenes (An=Th–Cm): multiconfigurational studies and topological analysis. *RSC Adv.* **4**, 12078–12086 (2014).

[50] Kerridge, A. & Kaltsoyannis, N. Are the ground states of the later actinocenes multiconfigurational? All-electron spin−orbit coupled CASPT2 calculations on An(η^8^-C_8_H_8_)_2_ (An=Th, U, Pu, Cm). *J. Phys. Chem. A* **113**, 8737–8745 (2009). 10.1021/jp903912q 19719318

[51] Liu, W., Dolg, M. & Fulde, P. Low-lying electronic states of lanthanocenes and actinocenes M(C_8_H_8_)_2_ (M=Nd, Tb, Yb, U). *J. Chem. Phys.* **107**, 3584–3591 (1997).

[52] Chowdhury, S. R., Goodwin, C. A. P. & Vlaisavljevich, B. What is the nature of the uranium(III)–arene bond? *Chem. Sci.* **15**, 1810–1819 (2024). 10.1039/d3sc04715f PMC10829017 38303954

[53] Perdew, J. P., Ernzerhof, M. & Burke, K. Rationale for mixing exact exchange with density functional approximations. *J. Chem. Phys.* **105**, 9982–9985 (1996).

[54] Adamo, C. & Barone, V. Toward reliable density functional methods without adjustable parameters: the PBE0 model. *J. Chem. Phys.* **110**, 6158–6170 (1999).

[55] Lu, E. et al. Emergence of the structure-directing role of f-orbital overlap-driven covalency. *Nat. Commun.* **10**, 634 (2019). 10.1038/s41467-019-08553-y PMC6367379 30733443

[56] Blokker, E. et al. The chemical bond: when atom size instead of electronegativity difference determines trend in bond strength. *Chem. - Eur. J.* **27**, 15616–15622 (2021). 10.1002/chem.202103544 PMC9298008 34609774

[57] Forslund, L. E. & Kaltsoyannis, N. Why is the F_2_ bond so weak? A bond energy decomposition analysis. *N. J. Chem.* **27**, 1108–1114 (2003).

[58] Aranzaes, J. R., Daniel, M.-C. & Astruc, D. Metallocenes as references for the determination of redox potentials by cyclic voltammetry — Permethylated iron and cobalt sandwich complexes, inhibition by polyamine dendrimers, and the role of hydroxy-containing ferrocenes. *Can. J. Chem.* **84**, 288–299 (2006).

[59] Goodwin, C. A. P. et al. Isolation and electronic structures of derivatized manganocene, ferrocene and cobaltocene anions. *Nat. Chem.* **13**, 243–248 (2021). 10.1038/s41557-020-00595-w PMC7610420 33318673

[60] Neese, F. Software Update: The ORCA Program System—Version 6.0. *WIREs Comput. Mol. Sci.* **15**, e70019 (2025).

[61] Grimme, S., Ehrlich, S. & Goerigk, L. Effect of the damping function in dispersion corrected density functional theory. *J. Comput. Chem.* **32**, 1456–1465 (2011). 10.1002/jcc.21759 21370243

[62] Neese, F., Wennmohs, F., Hansen, A. & Becker, U. Efficient, approximate and parallel Hartree–Fock and hybrid DFT calculations. A ‘chain-of-spheres’ algorithm for the Hartree–Fock exchange. *Chem. Phys.* **356**, 98–109 (2009).

[63] Izsak, R. & Neese, F. An overlap fitted chain of spheres exchange method. *J. Chem. Phys.* **135**, 144105 (2011). 10.1063/1.3646921 22010696

[64] Peng, D. & Reiher, M. Local relativistic exact decoupling. *J. Chem. Phys.* **136**, 244108 (2012). 10.1063/1.4729788 22755566

[65] Pollak, P. & Weigend, F. Segmented contracted error-consistent basis sets of double- and triple-ζ valence quality for one- and two-component relativistic all-electron calculations. *J. Chem. Theory Comput.* **13**, 3696–3705 (2017). 10.1021/acs.jctc.7b00593 28679044

[66] Franzke, Y. J., Treß, R., Pazdera, T. M. & Weigend, F. Error-consistent segmented contracted all-electron relativistic basis sets of double- and triple-zeta quality for NMR shielding constants. *Phys. Chem. Chem. Phys.* **21**, 16658–16664 (2019). 10.1039/c9cp02382h 31317138

[67] Pritchard, B. P., Altarawy, D., Didier, B., Gibson, T. D. & Windus, T. L. New basis set exchange: an open, up-to-date resource for the molecular sciences community. *J. Chem. Inf. Model.* **59**, 4814–4820 (2019). 10.1021/acs.jcim.9b00725 31600445

[68] Schuchardt, K. L. et al. Basis set exchange: a community database for computational sciences. *J. Chem. Inf. Model.* **47**, 1045–1052 (2007). 10.1021/ci600510j 17428029

[69] Feng, R. & Peterson, K. A. Correlation consistent basis sets for actinides. II. The atoms Ac and Np-Lr. *J. Chem. Phys.* **147**, 084108 (2017). 10.1063/1.4994725 28863538

[70] Stoychev, G. L., Auer, A. A. & Neese, F. Automatic generation of auxiliary basis sets. *J. Chem. Theory Comput.* **13**, 554–562 (2017). 10.1021/acs.jctc.6b01041 28005364

[71] Baerends, E. J. et al. The Amsterdam modeling suite. *J. Chem. Phys.* **162**, 162501 (2025). 10.1063/5.0258496 40260801

[72] Michalak, A., DeKock, R. L. & Ziegler, T. Bond multiplicity in transition-metal complexes: applications of two-electron valence indices. * J. Phys. Chem. A* **112**, 7256–7263 (2008). 10.1021/jp800139g 18627137

[73] Nalewajski, R. F., Mrozek, J. & Michalak, A. Two-electron valence indices from the Kohn-Sham orbitals. *Int. J. Quantum Chem.* **61**, 589–601 (1997).

[74] Mitoraj, M. P., Michalak, A. & Ziegler, T. A combined charge and energy decomposition scheme for bond analysis. *J. Chem. Theory Comput.* **5**, 962–975 (2009). 10.1021/ct800503d 26609605

[75] Sagan, F. & Mitoraj, M. P. Kinetic and potential energy contributions to a chemical bond from the charge and energy decomposition scheme of extended transition state natural orbitals for chemical valence. *J. Phys. Chem. A* **123**, 4616–4622 (2019). 10.1021/acs.jpca.9b01420 31058501

[76] Caldeweyher, E. et al. A generally applicable atomic-charge dependent London dispersion correction. *J. Chem. Phys.* **150**, 154122 (2019). 10.1063/1.5090222 31005066

[77] van Lenthe, E. & Baerends, E. J. Optimized Slater-type basis sets for the elements 1-118. *J. Comput. Chem.* **24**, 1142–1156 (2003). 10.1002/jcc.10255 12759913

[78] van Lenthe, E., Baerends, E. J. & Snijders, J. G. Relativistic regular two-component Hamiltonians. *J. Chem. Phys.* **99**, 4597–4610 (1993).

[79] van Lenthe, E., Baerends, E. J. & Snijders, J. G. Relativistic total energy using regular approximations. *J. Chem. Phys.* **101**, 9783–9792 (1994).

[80] AIMAll v. Version 19.10.12 (TK Gristmill Software, 2019).

[81] Glendening, E. D., Landis, C. R. & Weinhold, F. NBO 7.0: New vistas in localized and delocalized chemical bonding theory. *J. Comput. Chem.* **40**, 2234–2241 (2019). 10.1002/jcc.25873 31172571

[82] Clegg, W. et al. *Crystal Structure Analysis*. 2nd edn, (Oxford University Press, 2009).

