# Supplementary material for: Isolation of uranocenium, the bis-[8]annulene uranium cation
Source: Nat Commun. 2026 Oct 23;17:10914. doi: 10.1038/s41467-026-77066-2 (PMC13694272; doi:10.1038/s41467-026-77066-2)
Supplement: Supplementary file 1 [file 41467_2026_77066_MOESM1_ESM.pdf]

Supplementary Information for:

Isolation of Uranocenium, the *bis*-[8]annulene Uranium Cation

Cameron N. Deakin[1,2], Iulia Turcas[2,3], Benjamin L. L. Réant[1,2,†], Liam P. Griffin[1,2†], John A. Seed[1,2], Adam Brookfield[3], David Collison[2,3], Eric J. L. McInnes[2,3*], Andrew Kerridge[4*], Conrad A. P. Goodwin[1,2*]

[1] Centre for Radiochemistry Research, The University of Manchester, Manchester, M13 9PL, UK.

[2] Department of Chemistry, The University of Manchester, Manchester, M13 9PL, UK.

[3] Photon Science Institute, The University of Manchester, Manchester, M13 9PL, UK.

[4] Department of Chemistry, Lancaster University, Lancaster, LA1 4YB, UK.

* Correspondence: eric.mcinnes@manchester.ac.uk
a.kerridge@lancaster.ac.uk
conrad.goodwin@manchester.ac.uk

† These authors contributed equally.

Table of contents

S1. Experimental details 3
Equipment, materials, and solvents 3
Synthesis of precursors and materials 5
Synthesis of complexes **1**–**10** 10
S2. ATR-IR / FTIR spectroscopy 19
S3. X-ray diffraction 23
General considerations – single crystal X-ray diffraction (SCXRD) 23
Molecular structures 29
General considerations – powder X-ray diffraction (PXRD) 36
S4. NMR spectroscopy 45
Precursors and materials 45
Complexes **1**–**6**, **9**, and **10** 52
Additional discussion of NMR data 65
Small-scale oxidation and reactivity studies 65
S5. Solution phase UV-Vis-NIR spectroscopy 95
Decomposition studies of complexes **3** and **6** 107
Solid-state UV-vis-NIR spectroscopy 114
S6. Electrochemistry 117
Materials and general considerations 117
Data for **3**, **6**, and $[Fe(Cp)_2]$ in oDFB supported by $[nBu_4N][B(C_6F_5)_4]$ 119
Data for **1**, **4**, and $[Fe(Cp)_2]$ in THF supported by $[nBu_4N][B(C_6F_5)_4]$ 131
Data for **1** and **4** in THF supported by $[nBu_4N][PF_6]$ 143
S7. SQUID Magnetometry 154
General details 154
Variable-temperature SQUID magnetometry 155
Analysis of magnetometry data 166
S8. EPR Spectroscopy 168
General details 168
Experimental parameters 169
Summary of experimental and simulated EPR parameters 169
Experimental and simulated EPR spectra 171
Admixtures between $|J, \pm m_J\rangle$ states accounting for experimental Ce(III) g-values 174
S9. Computational methodology – DFT 178
General considerations 178
Metal spin and charge analysis for **1**–**6** 180
Frontier molecular orbital compositions and visualizations and compositions 181
Natural bonding molecular orbital compositions and visualizations 200
Summary of δ-bonding MO and NLMO compositions for U(IV/V) complexes 210
Energy decomposition analysis for $\{M(COT^R)\}^{n+}$ + $\{COT^R\}^{2-}$ with **1**–**6** 216
Topological analysis of the electron density 230
S10. Computational methodology – CASSCF & RASSCF 233
General considerations 233
Active spaces 233
State averaging 234
Electronic structure 234
EPR g-values 236
Active space orbitals 237
S11. References 244

S1. Experimental details

Equipment, materials, and solvents

Caution! Natural-abundance uranium (assumed standard composition: 0.7204% ^{235}U, $t_{1/2}$ = 7.04×10^8 years; 99.2742% ^{238}U, $t_{1/2} = 4.468\times10^9$ years) is a weak α-emitter (4.198 and 4.151 MeV from ^{238}U). Along with α-, β-, and γ-emitting decay products, this material represents both radiotoxicity (α-particles) and heavy-metal health hazards. Hence, all manipulations were performed in continuous extraction fume cupboards or positive pressure inert atmosphere gloveboxes located in a dedicated laboratory with contamination monitoring protocols, along with training and materials for decontamination equipment. Additional safeguards include the use of hand-held radiation monitoring equipment and dosimetry surveys for all workers.

Unless otherwise described, all syntheses and manipulations were conducted under BOC PureShield argon (99.995%) with rigorous exclusion of oxygen and water using Schlenk line and glove box techniques in a Glovebox Systemtechnik MEGA. 3 Å molecular sieves were activated by heating for 8 hours at 300°C, 10^{-3} mbar. *n*-hexane, Et_2O, THF and toluene were degassed by sparging (N_2) and dried by passage through neutral alumina columns (INERT Corp.), then degassed under vacuum, stored over a K mirror and used immediately (*n*-hexane, Et_2O, toluene), or stored over 3 Å molecular sieves for >4 days before use (THF). *ortho*-difluorobenzene (*o*DFB) and *N*,*N*,*N*',*N*'-tetramethylethylenediamine (TMEDA) were dried by refluxing over CaH_2. 1,4-dioxane and mesitylene were dried by refluxing over K metal. After 4-5 days, the solvents were distilled into J. Young's valve appended vessels. d_6-benzene (Merck) and d_8-THF were dried by refluxing over K metal. After 4-5 days, the solvents were vacuum transferred to J. Young's valve-appended vessels. 12-crown-4 and d_5-bromobenzene were dried by storage on 3 Å molecular sieves for one week. Naphthalene was purified by sublimation before use, and oil-free Li^0 metal granules (Aldrich, 99% trace metal basis) were used without further purification. $Ag^I[SbF_6]$ (Merck) was dried under vacuum (10^{-3} mbar) in the dark for 24 hours before use. $[Ce^{III}I_3(THF)_4]$[1], $U^{IV}Cl_4$ (ref.[2]), $[Ph_3C][B(C_6F_5)_4]$[3], and $K_2COT^{††}$ (refs.[4,5]) were prepared as described previously, and $[U^{IV}(COT)_2]$ (**1**) was synthesized via modification of a previously reported route[6]. See below for details on the synthesis of all these materials as well as $[nBu_4N][PF_6]$ and $[nBu_4N][SbF_6]$. $[Fe(Cp^*)_2]$ (Fc*; Cp* = $\{C_5Me_5\}$; Merck), and $[Fe(Cp)_2]$ (Fc; Cp = $\{C_5H_5\}$; Aldrich) were sublimed prior to use. $[nBu_4N]Cl$ (Fluorochem), $Na[PF_6]$ (Fluorochem), $Na[SbF_6]$ (ThermoScientific), and $K[B(C_6F_5)_4]$ (Ambeed) were used as received.

Schlenk glassware was heated strongly using a butane flame under vacuum (10^{-3} mbar) and then allowed to cool before use. Borosilicate glass vials (4 mL and 20 mL) used for inert atmosphere glovebox synthesis techniques were stored in a vacuum oven (160°C, 10^{-1} mbar) for at least 12 hours and then under vacuum (10^{-3} mbar) in the glovebox antechamber for at least 16 hours before use. All glassware used with solutions containing actinide materials was silanized using Me_3SiCl before use. Glass-fibre filter discs, PTFE-coated stirrer bars (25 × 8 mm and 12 × 3 mm) and glass-coated stirrer bars (25 × 6 mm) were stored in an oven (150°C) for at least 12 hours before use. NMR spectroscopic data collection was performed on either a Bruker Avance III (400 MHz), a Bruker Advance III HD (400 MHz), or a JEOL JNM-ECZ 400 MHz spectrometer between 295 K to 299 K. Solution and solid phase UV-Vis-NIR spectra were collected at ambient temperature using a PerkinElmer Lambda 1050 UV-Vis-NIR spectrometer. Solutions were contained either in a Hellma low volume (1 mL) screw-capped quartz cuvette (10 × 4 mm path length fluorescence cell), a Hellma 1 mm path length absorbance cell, or, in a quartz J. Youngs tap appended 10 mm pathlength fluorescence cell. Solids were contained in the Praying Mantis attachment, and Spectralon® was used as the reference. ATR FT-IR spectra of microcrystalline samples were collected using a Bruker ALPHA II FT-IR spectrometer equipped with a Platinum ATR module with a diamond window. Elemental microanalyses (C/H/N) were carried out by Martin Jennings and Anne Davies at the University of Manchester. Customized glassware was produced by David Marshall and Steven Green at the University of Manchester.

A note on NMR spectroscopy of paramagnetic samples

All spectra were referenced to internal solvent residuals (^{1}H and ^{13}C) or externally to 10% TMS in d_1-chloroform *via* Eq. **S1**, which is the IUPAC recommended convention.

$$\Delta\ (Hz) = \frac{SR^{1H}}{SF^{1H}} \times SF^{NUC}$$

Eq. S1.

Where SR^{1H} is the spectrum reference frequency (in Hz) of a reference ^{1}H NMR spectrum collected with TMS set to 0 ppm collected under the same experimental conditions; SF^{1H} is the spectrometer frequency (in MHz) for the ^{1}H nucleus; SF^{NUC} is the spectrometer frequency (in MHz) of the nucleus in question. The answer is given in Hz.

Table S1. Compound numbering, formula, reaction scale (by metal quantity or limiting reagent used), yield, and then % yield where appropriate.

	Scale (mmol metal)	Formula	Yield (g)	% yield
**1**	10.00	$[U^{IV}(COT)_2]$	3.114	70
**2**	1.00	$[Li(12\text{-crown-}4)(THF)][U^{III}(COT)_2]$	0.140	20
**3**	0.58	$[U^{V}(COT)_2][B(C_6F_5)_4]$	0.315	48
**4**	5.00	$[U^{IV}(COT^{\dagger\dagger})_2]$	3.119	58
**5**	0.86	$[Li(TMEDA)_2][U^{III}(COT^{\dagger\dagger})_2]\cdot(C_9H_{12})$	0.323	26
**6**	0.86	$[U^{V}(COT^{\dagger\dagger})_2][B(C_6F_5)_4]$	0.578	38
**7** [A]	–	$[\{U^{IV}(COT^{\dagger\dagger})(\mu\text{-}F)_2\}_3]$	–	–
**8** [A]	–	$[Li(12\text{-crown-}4)_2][U^{III}(COT)_2]$	–	–
**9**	1.00	$[Ce^{IV}(COT^{\dagger\dagger})_2]$	0.441	45
**10**	1.09	$[Li(TMEDA)_2][Ce^{III}(COT^{\dagger\dagger})_2]\cdot(C_9H_{12})$	0.444	31

Note: Yields are representative of a single iteration only and are intended only to indicate what might be expected from these unoptimized reactions. In each case, the yield is given with respect to a limiting reagent such as the quantity of metal halide used. [A] Obtained as part of a mixture.

Synthesis of precursors and materials

Synthesis of $U^{IV}Cl_4$

Hexachloropropene (200 mL) was added to a 1 L J. Youngs valve appended round-bottomed flask containing U_3O_8 (21.970 g, 26.1 mmol) and a PTFE-coated stirrer bar. The Schlenk flask was equipped with a CondenSyn® water-free condenser and the flask placed under an argon gas supply. The mixture was heated carefully to reflux, which was accompanied by a violent exotherm and the liberation of a dark brown gas. The flask was lifted away from the heating mantle to allow the exotherm to subside before heating was resumed. The reaction mixture was then left to gently reflux overnight (18 hours), after which the green suspension was allowed to cool to room temperature. The green solids were isolated by filtration and washed three times with DCM (3 × 150 mL). The solids were dried under vacuum (10^{-3} mbar) for 4 hours to give UCl_4 in excellent yield (29.730 g, 92%) and was used without further purification.

Synthesis of $[Ce^{III}I_3(THF)_4]$

THF (125 mL) was added to a pre-cooled (0°C) Schlenk vessel containing a Ce^0 metal filings (4.98 g, 35.5 mmol, 1 equiv.) and a PTFE-coated stirrer bar followed by the portion wise addition of solid iodine (13.515 g, 53.3 mmol, 1.5 equiv.). The mixture was allowed to warm to room temperature and stirred for three days to give a pale brown suspension. The

suspension was concentrated by 50% followed by the addition of Et_2O (50 mL) and pentane (50 mL) and stirred for 30 minutes. The solids were allowed to settle briefly and the supernatant filtered through a glass microfibre filter. The solids were washed once with Et_2O (50 mL) and pentane (50 mL). The solids were dried under vacuum (10^{-3} mbar) at 120°C for 6 hours to give a beige solid. The solids were purified by Soxhlet extraction in THF at 100°C. The beige suspension was allowed to cool and concentrated by 30% followed by the addition of Et_2O (50 mL) and pentane (50 mL). The supernatant was filtered through a glass microfibre filter, and the solids were dried under vacuum (10^{-3} mbar) for 4 hours to give [$Ce^{III}I_3(THF)_4$] in excellent yield (26.430 g, 92%).

K_2COT

THF (30 mL) was added to a Schlenk vessel containing K^0 metal (6.950 g, 177.8 mmol, 2 equiv.) and a PTFE-coated stirrer bar. Separately, THF (10 mL) was added to a Schlenk vessel containing COT (9.250 g, 88.8 mmol, 1 equiv.) and a PTFE-coated stirrer bar. The COT solution was added dropwise to the pre-cooled (–98°C) K^0 metal suspension. The mixture was allowed to warm slowly to room temperature and left to stir for 48 hours to give a dark red suspension. The suspension was filtered through a glass-fritted filter packed with Celite® into a new Schlenk vessel. The volatiles were removed under vacuum (10^{-3} mbar) to give K_2COT as a beige solid in fair yield (10.840 g, 68%), which was used without further purification.

^{1}H NMR (d_8-THF, 400.07 MHz, 298 K): δ = 5.75 (s, COT C<u>*H*</u>) ppm.

Synthesis of $H_2COT^{\dagger\dagger}$

Pentane (150 mL) was added to a 500 mL J. Youngs appended round-bottomed flask containing a mixture of COD (4.91 mL, 40.0 mmol, 1 equiv.), TMEDA (18.34 mL, 120.0 mmol, 3 equiv.) and a PTFE-coated stirrer bar. The colourless solution was cooled to 0°C and *n*-BuLi (49.6 mL, 124.0 mmol, 3.1 equiv.) was added dropwise to the colourless mixture. The mixture was allowed to warm to room temperature and stirred overnight (18 hours) to give an orange suspension. The Schlenk flask was equipped with a CondenSyn® water-free condenser and refluxed overnight (18 hours) to give an orange suspension. The volatiles were removed under vacuum (10^{-3} mbar) to give an orange solid. DME (100 mL) was added, and the mixture filtered through a fritted glass filter packed with Celite® into a new Schlenk vessel. TIPS-triflate (21.5 mL, 80.0 mmol, 2 equiv.) was added dropwise to the pre-cooled

(–35°C) orange solution and the mixture was allowed to warm to room temperature and stirred overnight (18 hours) which gave a colourless solution. The volatiles were removed under vacuum (10^{-3} mbar) to give a colourless solid. Petroleum ether (150 mL) was added, and the supernatant filtered through a fritted glass filter. The volatiles removed under vacuum (10^{-3} mbar) to give a colourless oil which was crystallised from acetone after storage at –35°C to give a single crystalline crop of $H_2COT^{\dagger\dagger}$ in fair yield (9.325 g, 56%).

^{1}H NMR (d_1-chloroform, 400.07 MHz, 298 K): δ = 6.10 (d, J_{HH} = 9.0 Hz, 2H, COT C*H*), 5.65 (s, 2H, COT C*H*), 5.45 (d, J_{HH} = 8.1 Hz, 2H, COT C*H*), 2.79 (d, J_{HH} = 7.7 Hz, 2H, COT C*H*), 1.20 (sept, J_{HH} = 7.1 Hz, 6H, TIPS C*H*)., 1.07 (dd, J_{HH} = 7.5 Hz and 2.6 Hz, 36H, TIPS CH_3) ppm.

Synthesis of $K_2COT^{\dagger\dagger}$

Hexane (100 mL) was added to a pre-cooled (0°C) Schlenk vessel containing a mixture of $H_2COT^{\dagger\dagger}$ (6.446 g, 15.47 mmol, 1 equiv.), KO*t*Bu (3.47 g, 30.94 mmol, 2 eq.) and a PTFE-coated stirrer bar followed by the dropwise addition of *n*-BuLi (13.0 mL, 32.48 mmol, 2.1 equiv.). The mixture was allowed to warm to room temperature at stirred overnight (18 hours) to give a colourless suspension. The colourless solids were allowed to settle briefly, the supernatant filtered through a glass microfibre filter and washed with hexane (50 mL). The volatiles were removed under vacuum (10^{-3} mbar) to give a colourless solid and were dried under vacuum (10^{-3} mbar) for 4 hours to give $K_2COT^{\dagger\dagger}$ in excellent yield (6.02 g, 92%).

^{1}H NMR (d_6-benzene, 400.07 MHz, 298 K): δ = 6.49 (m, 6H, COT C*H*), 1.84 (sept, J_{HH} = 7.5 Hz, 6H, TIPS C*H*), 1.37 (d, J_{HH} = 7.5 Hz, 36H, TIPS C*H*3) ppm.

^{29}Si{^{1}H} NMR (d_6-benzene, 79.47 MHz, 298 K): δ = 5.50 (s, TIPS Si) ppm.

Synthesis of $[Ph_3C][B(C_6F_5)_4]$

Method 1. A solution of bromopentafluorobenzene (11.0 mL, 88.2 mmol) in hexane (250 mL) was cooled to –78°C in a dry ice–acetone bath. *n*-BuLi (32.0 mL, 80 mmol) was added to the solution dropwise, and the mixture was stirred at –78°C for 2.5 hours. BCl_3 (1.0 M in hexanes, 16.0 mL, 16.0 mmol) was added to the mixture dropwise at –78°C, and the resulting viscous suspension was stirred and warmed to room temperature gradually overnight (18 hours). The volatiles were removed from the mixture under vacuum, and the remaining solid was brought into a glovebox and ground into a fine powder. The powder, putatively "$Li[B(C_6F_5)_4]$" was suspended in hexane (200 mL), and solid Ph_3CCl (9.80 g, 35.2

mmol) was added using a solid-addition Schlenk side-arm. A reaction occurred immediately, and a yellow powder formed. The mixture was stirred at room temperature overnight and filtered through a glass frit. The collected powder was washed with hexane (3 × 50 mL) and dried under vacuum for 3 hours. The yellow solid was taken up in dichloromethane (150 mL) and filtered to remove LiCl. The filtrate was concentrated to *ca*. 15 mL, and pentane (200 mL) was added. Phase separation occurred initially, but as the mixture was stirred, the oily lower layer gradually solidified. The supernatant was removed by a cannula, and the remaining powder was dried under vacuum to afford $[Ph_3C][B(C_6F_5)_4]$ as a yellow powder (13.50 g, 91%), which was used without further purification.

Method 2. The reaction described in **Method 1** was performed using commercial $K[B(C_6F_5)_4]$ instead of synthesising $Li[B(C_6F_5)_4]$ in the first step. DCM (100 mL) was added to a Schlenk vessel containing a mixture of $K[B(C_6F_5)_4]$ (25.000 g, 34.8 mmol, 1 equiv.), Ph_3CCl (9.701 g, 34.8 mmol, 1 equiv.) and a PTFE-coated stirrer bar. The mixture was stirred overnight (18 hours) to give a bright yellow solution over pale solids presumed to be KCl. The supernatant was filtered through a glass microfiber filter disc into a new Schlenk vessel, and the volatiles were removed under vacuum (10^{-3} mbar), to give a bright yellow oil. Hexane (100 mL) was added, and the suspension sonicated until a yellow solid precipitated. The solids were isolated by filtration, washed with hexane (3 x 50 mL) and dried under vacuum (10^{-3} mbar) to afford $[Ph_3C][B(C_6F_5)_4]$ as a yellow powder (27.283 g, 85%), which was used without further purification.

$[nBu_4N][B(C_6F_5)_4]$

Deionized H_2O (350 mL) was added at room temperature to a 1 L RBF charged with $[n\text{Bu}_4N]Cl$ (4.440 g, 15 mmol), $K[B(C_6F_5)_4]$ (10.772 g, 15 mmol), and a PTFE-coated stirrer bar. A white precipitate quickly formed, and the suspension was stirred for 72 hours. The suspension was extracted with DCM (5 x 150 mL), and the combined organics were washed with deionized H_2O (2 x 150 mL). After drying with $MgSO_4$ and filtering, the DCM was removed under vacuum (100 mbar) to afford the electrolyte $[n\text{Bu}_4N][B(C_6F_5)_4]$ as a white solid. The electrolyte was dissolved in the minimum amount of hot methanol (<40 mL), left to slowly cool to room temperature, followed by further storage at –35°C overnight. The crystals were isolated by filtration and air-dried. A further two crystallizations were carried out under the same conditions. The triply recrystallized material was transferred to a Schlenk

flask and dried under vacuum (4 x 10^{-3} mbar) at 100°C for 16 hours. Yield of [nBu$_4$N][B(C_6F_5)$_4$] = 9.843 g, 10.7 mmol, 76%.

^{1}H NMR (d-chloroform, 400.07 MHz, 298 K): δ = 2.97–3.04 (m, 8H, NC$\underline{H}_2$CH$_2$CH$_2$CH$_3$), 1.50–1.59 (m, 8H, NCH$_2$C$\underline{H}_2$CH$_2$CH$_3$), 1.33 (qt, $^{1}J_{HH}$ = 7.4 Hz, 7.4 Hz, 8H, NCH$_2$CH$_2$C$\underline{H}_2$CH$_3$), 0.95 (t, $^{1}J_{HH}$ = 7.4 Hz, 12H, NCH$_2$CH$_2$CH$_2$C$\underline{H}_3$) ppm.

^{11}B NMR (d-chloroform, 128.36 MHz, 298 K): δ = –16.65 (s, $\underline{B}$(C_6F_5)$_4$) ppm.

^{13}C{^{1}H} NMR (d-chloroform, 100.61 MHz, 298 K): δ = 148.28 (d, J = 241.1 Hz, o-$\underline{C}_6F_5$), 138.31 (br, d, J = 245.0 Hz, p-$\underline{C}_6F_5$), 136.30 (br, d, J = 245.4 Hz, m-$\underline{C}_6F_5$), 123.04–125.29 (br, m, i-$\underline{C}_6F_5$), 58.90 (s, N$\underline{C}$H$_2$CH$_2$CH$_2$CH$_3$), 23.69 (s, NCH$_2\underline{C}$H$_2$CH$_2$CH$_3$), 19.57 (s, NCH$_2$CH$_2\underline{C}$H$_2$CH$_3$), 13.30 (s, NCH$_2$CH$_2$CH$_2\underline{C}$H$_3$) ppm.

^{19}F NMR (d-chloroform, 376.40 MHz, 298 K): δ = –132.51 (br, d, o-$C_6\underline{F}_5$), –162.72 (t, J = 20.4 Hz, p-$C_6\underline{F}_5$), –166.70 (br, d, m-$C_6\underline{F}_5$) ppm.

[nBu$_4$N][PF$_6$]

Deionized H_2O (300 mL) was added at room temperature to a 500 mL RBF charged with [nBu$_4$N]Cl (8.120 g, 29 mmol), Na[PF$_6$] (4.872 g, 29 mmol), and a PTFE-coated stirrer bar. A white precipitate quickly formed, and the suspension was stirred for 72 hours. The precipitate was dissolved with DCM (200 mL) and separated from the aqueous layer. The aqueous layer was further washed with DCM (3 x 100 mL), and the combined organics were washed with deionized H_2O (2 x 150 mL). After drying with $MgSO_4$ and filtering, the DCM was removed under vacuum (100 mbar) to afford the electrolyte [nBu$_4$N][PF$_6$] as a white solid. The electrolyte was dissolved in the minimum amount of hot ethanol (<70 mL), left to slowly cool to room temperature, followed by further storage at –35°C overnight. The crystals were isolated by filtration and air-dried. A further two crystallizations were carried out under the same conditions. The triply recrystallized material was transferred to a Schlenk flask and dried under vacuum (4 x 10^{-3} mbar) at 60°C for 16 hours. Yield of [nBu$_4$N][PF$_6$] = 9.270 g, 23.9 mmol, 83%.

^{1}H NMR (d-chloroform, 400.07 MHz, 298 K): δ = 3.11–3.17 (m, 8H, NC$\underline{H}_2$CH$_2$CH$_2$CH$_3$), 1.54–1.62 (m, 8H, NCH$_2$C$\underline{H}_2$CH$_2$CH$_3$), 1.40 (qt, $^{1}J_{HH}$ = 7.3 Hz, 7.3 Hz, 8H, NCH$_2$CH$_2$C$\underline{H}_2$CH$_3$), 0.98 (t, $^{1}J_{HH}$ = 7.3 Hz, 12H, NCH$_2$CH$_2$CH$_2$C$\underline{H}_3$) ppm.

^{13}C{^{1}H} NMR (d-chloroform, 100.61 MHz, 298 K): δ = 58.58 (s, N$\underline{C}$H$_2$CH$_2$CH$_2$CH$_3$), 23.79 (s, NCH$_2\underline{C}$H$_2$CH$_2$CH$_3$), 19.64 (s, NCH$_2$CH$_2\underline{C}$H$_2$CH$_3$), 13.61 (s, NCH$_2$CH$_2$CH$_2\underline{C}$H$_3$) ppm.

^{19}F NMR (d-chloroform, 376.40 MHz, 298 K): δ = –72.30 (d, $^{1}J_{PF}$ = 712.8 Hz, P$\underline{F}_6$) ppm.

^{31}P NMR (d-chloroform, 161.95 MHz, 298 K): δ = –144.40 (sept, $^{1}J_{PF}$ = 712.8 Hz, $\underline{P}$F$_6$) ppm.

Synthesis of [nBu$_4$N][SbF$_6$]

Deionised H_2O (300 mL) was added at room temperature to a 500 mL RBF charged with [*n*Bu$_4$N]Cl (5.370 g, 19.3 mmol), Na[SbF$_6$] (5.000 g, 19.32 mmol), and a PTFE-coated stirrer bar. A white precipitate quickly formed, and the suspension was stirred overnight (16 hours). The precipitate was dissolved with DCM (200 mL) and separated from the aqueous layer. The aqueous layer was further washed with DCM (3 × 100 mL), and the combined organic layers were washed with deionised H_2O (2 x 150 mL). After drying over $MgSO_4$ followed by filtration, the volatiles were removed under vacuum (100 mbar) to afford crude [*n*Bu$_4$N][SbF$_6$] as a white solid. The material was dissolved in the minimum amount of hot ethanol (<70 mL), left to slowly cool to room temperature, followed by storage at –35°C overnight. Crystals were isolated by filtration, then transferred to a Schlenk flask and dried under vacuum (4 × 10^{-3} mbar) at 50°C for 8 hours to give [*n*Bu$_4$N][SbF$_6$] in moderate isolated crystalline yield (4.548 g, 49%).

^{1}H NMR (*d*-chloroform, 400.07 MHz, 298 K): δ = 3.12 (m, 8H, NC$\underline{H}_2$), 1.59 (m, 8H, NCH$_2$C$\underline{H}_2$), 1.41 (hxt, J_{HH} = 7.3 Hz, 8H, C$\underline{H}_2$CH$_3$), 0.99 (t, J_{HH} = 7.3 Hz, 12H, CH$_2$C$\underline{H}_3$) ppm.

^{19}F{^{1}H} NMR (*d*-chloroform, 376.40 MHz, 298 K): δ = –122.44 (m, SbF$_6$) ppm.

*Synthesis of complexes **1–10***

*Synthesis of [U^{IV}(COT)$_2$] (**1**)*

THF (40 mL) was added to a pre-cooled (–98°C) Schlenk vessel containing a mixture of U^{IV}Cl$_4$ (3.798 g, 10.0 mmol, 1 equiv.), and K$_2$COT (3.647 g, 20.0 mmol, 2 equiv.) and a PTFE-coated stirrer bar. The mixture was allowed to warm to room temperature and stirred overnight (18 hours) to give a dark green solution over pale solids, presumed to be KCl. Volatiles were removed under vacuum (10^{-3} mbar), which gave a dark green solid. The solids were purified by Soxhlet extraction into toluene at 160°C. The dark green suspension was allowed to cool, and the volatiles were removed under vacuum (10^{-3} mbar), which gave dark green crystals. The crystals were dried under vacuum (10^{-3} mbar) for 4 hours to give **1** in good overall isolated crystalline yield (3.114 g, 70%).

^{1}H NMR (d_8-THF, 400.07 MHz, 298 K): δ = −36.99 (br. s, $\bar{\nu}$ = 82.9 Hz, COT C$\underline{H}$) ppm.

^{13}C{^{1}H} NMR (d_8-THF, 100.61 MHz, 298 K): δ = no resonances observed.

FT-IR (ATR, microcrystalline): cm^{-1} = 471 (vw), 504 (w), 520 (vw), 549 (w), 575 (w), 693 (vs), 744 (s), 779 (m), 793 (vs), 898 (s), 1,015 (s), 1,089 (m), 1,260 (m), 1,319 (vw).

*Synthesis of [Li(12-crown-4)(THF)][U^{III}(COT)$_2$] (**2**)*

THF (15 mL) was added to a pre-cooled (–98°C) Schlenk vessel containing a mixture of [U^{IV}(COT)$_2$] (0.446 g, 1.0 mmol, 1 equiv.), Li^0 metal (0.008 g, 1.2 mmol, 1.2 equiv.) and naphthalene (0.002 g, 0.016 mmol) and a glass-coated stirrer bar. The mixture was stirred for two days to give a dark brown solution. The supernatant was filtered through a glass microfiber filter disc into a new Schlenk vessel, and the volatiles were removed under vacuum (10^{-3} mbar), which gave putative [Li(THF)$_4$][U^{III}(COT)$_2$] (0.153 g, crude yield), which was used without further characterization.

THF (10 mL) was added to a glass vial containing putative [Li(THF)$_4$][U^{III}(COT)$_2$] (0.153 g, 0.21 mmol, 1 equiv.) and a PTFE-coated stirrer bar. 12-crown-4 (0.036 g, 0.21 mmol, 1 equiv.) was added to the brown solution and stirred briefly. Storage at –35°C gave a single crystalline crop of **2**. The product was isolated by decanting the supernatant while cold, followed by drying the crystals under vacuum (10^{-3} mbar) for 4 hours to give **2** in low overall isolated crystalline yield (0.140 g, 20%).

Elemental analysis on $C_{28}H_{40}LiO_5U$ calc. (%): C = 47.93, H = 5.75, N = 0.00; found (%): C = 48.26, H = 5.56, N = 0.00.

^{1}H NMR (d_8-THF, 400.07 MHz, 298 K): δ = 6.71 (br. s, $\bar{\nu}$ = 191.2 Hz, 16H, 12-crown-4 C$\underline{H}_2$), −31.18 (br. s, $\bar{\nu}$ = 194.3 Hz, 16H, COT C$\underline{H}$) ppm.

^{13}C{^{1}H} NMR (d_8-THF, 100.61 MHz, 298 K): δ = no resonances observed.

UV-Vis-NIR (THF, 0.55 mM): λ_{max} (ν/cm^{-1}; ε/M^{-1}cm^{-1}) = 1,456 (6,868; 280), 1,386 (7,215; 295), 1,318 (7,587; 248), 389 (25,707; 1,591).

FT-IR (ATR, microcrystalline): cm^{-1} = 477 (s), 508 (m), 543 (w), 559 (s), 592 (vw), 604 (vw), 678 (vs), 701 (m), 738 (m), 756 (vw), 859 (s), 890 (vs), 927 (s), 1,017 (s), 1,036 (s), 1,079 (vs), 1,130 (m), 1,241 (w), 1,282 (w), 1,297 (vw), 1,447 (w), 2,877 (w), 2,929 (vw), 2,953 (vw), 3,005 (m).

Repeat synthesis gave crystals of **2**$^{\beta}$, which differs in the unit cell but not structure or composition.

*Synthesis of [U^V(COT)$_2$][B(C_6F_5)$_4$] (**3**)*

*o*DFB (10 mL) was added to a pre-cooled (–35°C) Schlenk vessel containing a mixture of [U^{IV}(COT)$_2$] (0.260 g, 0.58 mmol, 1 equiv.) and [Ph_3C][B(C_6F_5)$_4$] (0.535 g, 0.58 mmol, 1

equiv.) and a PTFE-coated stirrer bar. The mixture was allowed to warm to room temperature and stirred overnight (18 hours) to give a dark brown solution. The supernatant was filtered through a glass microfiber filter disc into a new Schlenk vessel, and the volatiles were removed under vacuum (10^{-3} mbar) which gave a dark brown solid. The solids were washed with *n*-hexane (10 mL), and the volatiles were removed under vacuum (10^{-3} mbar). *o*DFB (2 mL) was added and the dark brown solution and layered with *n*-hexane (10 mL) Storage at room temperature gave a single crystalline crop of **3**. The product was isolated by decanting the supernatant while cold, and the crystals were dried under vacuum (10^{-3} mbar) for 4 hours to give **3** in fair isolated crystalline yield (0.315 g, 48%).

Elemental analysis on $C_{40}H_{16}BF_{20}U\cdot(C_6H_4F_2)_n$ for n = 1 calc. (%): C = 44.57, H = 1.63, N = 0.00; for n = 0 calc. (%): C = 42.69, H = 1.43, N = 0.00; found (%): C = 43.65, H = 1.54, N = 0.00. *Note*, the *o*DFB is present in the crystal structure of **3**, and elemental analysis results show incomplete desolvation under vacuum.

^{1}H NMR (d_5-bromobenzene, 400.07 MHz, 298 K): δ = 6.7 (m, *o*DFB C<u>*H*</u>), −21.46 (br. s, $\bar{\nu}$ = 112.6 Hz, COT C<u>*H*</u>) ppm.

^{11}B{^{1}H} NMR (d_5-bromobenzene, 128.36 MHz, 298 K): δ = −15.92 (s, $B(C_6F_5)_4$ <u>*B*</u>) ppm.

^{13}C{^{1}H} NMR (d_5-bromobenzene, 100.61 MHz, 298 K): δ = no resonances observed.

^{19}F{^{1}H} NMR (d_5-bromobenzene, 376.50 MHz, 298 K): δ = −129.70 (s, $B(C_6F_5)_4$ <u>*F*</u>), −137.25 (s, *o*DFB), −160.71 (m, $B(C_6F_5)_4$ <u>*F*</u>), −164.10 (m , $B(C_6F_5)_4$ <u>*F*</u>) ppm.

UV-Vis-NIR (THF, 0.35 mM): λ_{max} (ν/cm^{-1}; ε/M^{-1}cm^{-1}) = 700 (14,285; 1,039), 492 (20,325; 2,436), 448 (22,321; 2,717), 416 (24,038; 2,096), 376 (26,596; 2,972), 336 (29,762; 8,130), 326 (30,675; 7,601).

FT-IR (ATR, microcrystalline): cm^{-1} = 413 (m), 438 (w), 450 (m), 475 (m), 491 (w), 501 (w), 516 (w), 524 (vw), 534 (w), 547 (m), 573 (s), 602 (m), 610 (m), 625 (w), 660 (vs), 682 (s), 717 (vs), 732 (s), 756 (vs), 775 (s), 806 (w), 843 (w), 857 (vw), 904 (s), 976 (vs), 1,030 (vw), 1,081 (vs), 1,192 (vw), 1,266 (s), 1,324 (vw), 1,338 (vw), 1,375 (m), 1,412 (s), 1,455 (vs), 1,509 (s), 1,640 (m), 2,945 (vw), 3,062 (vw), 3,619 (vw), 3,627 (vw).

*Synthesis of [$U^{IV}(COT^{\dagger\dagger})_2$] (**4**)*

THF (40 mL) was added to a pre-cooled (–98°C) Schlenk vessel containing a mixture of $U^{IV}Cl_4$ (1.899 g, 5.0 mmol, 1 equiv.) and $K_2COT^{\dagger\dagger}$ (4.950 g, 10.0 mmol, 2 equiv.) and a PTFE-coated stirrer bar. The mixture was allowed to warm to room temperature and stirred overnight (18 hours) to give a dark green solution over pale solids, presumed to be KCl. Volatiles were removed under vacuum (10^{-3} mbar), which gave a dark green solid. Pentane

(25 mL) was added, the dark green mixture was briefly (<1 min.) heated to boiling, and the pale solids were manually freed from the vessel sidewalls. The solids were allowed to settle while the mixture cooled to room temperature, and the supernatant was filtered through a glass microfiber filter disc into a new Schlenk vessel. The clear, dark-green solution was concentrated to *ca*. 10 mL. Storage at –35°C to give two crystalline crops of **4**. The product was isolated by decanting the supernatant while cold, and the crystals were dried under vacuum (10^{-3} mbar) for 4 hours to give **4** in good isolated crystalline yield (3.119 g, 58%).
Elemental analysis on $C_{52}H_{96}Si_4U$ calc. (%): C = 58.28, H = 9.03, N = 0.00; found (%): C = 58.29, H = 9.12, N = 0.00.
^{1}H NMR (d_6-benzene, 400.07 MHz, 298 K): δ = −2.25 (m, 84H, TIPS C*H*/C*H*$_3$), −4.46 (s, 12H, $\bar{\nu}$ = 135.2 Hz, COT C*H*) ppm.
^{13}C{^{1}H} NMR (d_6-benzene, 100.61 MHz, 298 K): δ = no resonances observed.
^{29}Si{^{1}H} NMR (d_6-benzene, 79.47 MHz, 298 K): δ = no resonances observed.
UV-Vis-NIR (THF, 0.35 mM): λ_{max} (ν/cm^{-1}; ε/M^{-1}cm^{-1}) = 634 (15,773; 95), 616 (16,234; 226), 592 (16,892; 711), 404 (24,752; 1,005), 322 (31,056; 3,229), 304 (32,894; 3,628).
FT-IR (ATR, microcrystalline): cm^{-1} = 417 (m), 434 (m), 444 (m), 469 (w), 489 (w), 514 (vw), 551 (w), 571 (vw), 584 (w), 610 (w), 635 (vw), 664 (vw), 693 (vs), 742 (vw), 789 (m), 822 (s), 880 (vw), 921 (m), 939 (vw), 964 (m), 995 (m), 1,013 (vw), 1,023 (vw), 1,069 (s), 1,077 (s), 1,217 (s), 1,250 (s), 1,297 (s), 1,336 (vs), 1,365 (s), 1,383 (s), 1,404 (vs), 1,459 (w), 2,715 (vs), 2,758 (vs), 2,768 (vs), 2,861 (vw), 2,888 (m), 2,920 (w), 2,941 (w).

*Synthesis of [Li(TMEDA)$_2$][U^{III}(COT††)$_2$] (**5**)*

THF (15 mL) was added to a Schlenk vessel containing a mixture of [U^{IV}(COT††)$_2$] (0.918 g, 0.86 mmol, 1 equiv.) and Li^0 metal (0.007 g, 1.0 mmol, 1.2 equiv.) and a glass-coated stirrer bar. The mixture was stirred for five days to give a dark brown solution. The supernatant was filtered through a glass microfiber filter disc into a new Schlenk vessel, and the volatiles were removed under vacuum (10^{-3} mbar), which gave a dark brown solid. TMEDA (2 mL) was added, and the mixture was stored at –35°C, yielding large brown crystals. The crude product was isolated by decanting the supernatant while cold, the solid was washed with *n*-hexane (10 mL) and the crystals were dried under vacuum (10^{-3} mbar) for 4 hours. Mesitylene (2 mL) was added, briefly heated and storage at –35°C gave a single crystalline crop of **5**. The product was isolated by decanting the supernatant while cold, and the crystals were washed with *n*-hexane (5 mL) and dried under vacuum (10^{-3} mbar) for 4 hours to give **5** in low isolated crystalline yield (0.323 g, 26%).

Elemental analysis on $C_{64}H_{128}Si_4N_4LiU\cdot(C_9H_{12})$ calc. (%): C = 61.26, H = 9.86, N = 3.91; found (%): C = 61.24, H = 10.00, N = 3.62. *Note*, the mesitylene is present in the crystal structure of **5**, and elemental analysis results show incomplete desolvation under vacuum.
^{1}H NMR (d_8-THF, 400.07 MHz, 298 K): δ = 6.73 (s, 5H, mesitylene C$\underline{H}$), 2.30 (s, 10H, TMEDA C$\underline{H}_2$), 2.21 (s, 15H, mesitylene C$\underline{H}_3$), 2.15 (s, 30H, TMEDA C$\underline{H}_3$), −0.24 (br. s, 42H, TIPS C$\underline{H}$/C$\underline{H}_3$), −0.45 (br. s, 42H, TIPS C$\underline{H}$/C$\underline{H}_3$), −1.22 (br. s ,12H, $\bar{\nu}$ = 64.4 Hz, COT C$\underline{H}$).
^{13}C{^{1}H} NMR (d_8-THF, 100.61 MHz, 298 K): δ = 138.23 (s, mesitylene $\underline{C}$(CH$_3$)), 127.73 (s, mesitylene $\underline{C}$H), 59.08 (s, TMEDA $\underline{C}$H$_2$), 46.36 (s, TMEDA $\underline{C}$H$_3$), 21.46 (s, mesitylene $\underline{C}$H$_3$) ppm.
^{29}Si{^{1}H} NMR (d_8-THF, 79.47 MHz, 298 K): δ = no resonances observed.
UV-Vis-NIR (THF, 0.25 mM): λ_{max} (ν/cm^{-1}; ε/M^{-1}cm^{-1}) = 1,740 (5,747; 225), 1,563 (6,398; 274), 1,467 (6,815; 257), 1,428 (7,001; 241), 1,265 (7,908; 333), 1,234 (8,103; 381), 1,189 (8,413; 337), 637 (15,701; 91), 617 (16,213; 208), 592 (16,878; 630), 402 (24,894; 2,397).
FT-IR (ATR, microcrystalline): cm^{-1} = 419 (m), 438 (w), 446 (m), 469 (w), 491 (w), 508 (w), 518 (vw), 575 (vw), 604 (w), 637 (vw), 658 (vw), 688 (m), 721 (vw), 750 (s), 773 (s), 787 (m), 836 (w), 882 (vw), 929 (vw), 945 (m), 962 (m), 989 (m), 1,013 (vw), 1,034 (vw), 1,071 (s), 1,097 (vs), 1,128 (s), 1,159 (s), 1,184 (vs), 1,208 (s), 1,248 (m), 1,289 (m), 1,361 (s), 1,379 (s), 1,412 (vs), 1,459 (vw), 1,607 (s), 2,709 (vs), 2,717 (vs), 2,797 (s), 2,857 (vw), 2,886 (w), 2,916 (w), 2,937 (w), 3,025 (vs).

*Synthesis of [U^V(COT††)$_2$][B(C$_6$F$_5$)$_4$] (**6**)*

*o*DFB (10 mL) was added to a pre-cooled (–35°C) Schlenk vessel containing a mixture of [U^{IV}(COT††)$_2$] (0.921 g, 0.86 mmol, 1 equiv.) and [Ph$_3$C][B(C$_6$F$_5$)$_4$] (0.799 g, 0.86 mmol, 1 equiv.) and a PTFE-coated stirrer bar. The mixture was allowed to warm to room temperature and stirred overnight (18 hours) to give a dark red solution. The supernatant was filtered through a glass microfiber filter disc into a new Schlenk vessel, and the volatiles were removed under vacuum (10^{-3} mbar), which gave a dark red solid. The solids were washed with *n*-hexane (10mL), and the volatiles were removed under vacuum (10^{-3} mbar). *o*DFB (2 mL) was added, and the dark brown solution was layered with *n*-hexane (10 mL). Storage at room temperature gave a single crystalline crop of **6**. The product was isolated by decanting the supernatant while cold, and the crystals were dried under vacuum (10^{-3} mbar) for 4 hours to give **6** in low isolated crystalline yield (0.578 g, 38%).
Elemental analysis on $C_{76}H_{96}Si_4F_{20}BU$ calc. (%): C = 52.14, H = 5.53, N = 0.00; found (%): C = 51.26, H = 5.54, N = 0.00.

^{1}H NMR (d_5-bromobenzene, 400.07 MHz, 298 K): δ = 6.16 (br. s, $\bar{\nu}$ = 87.6 Hz, 12H, COT C*H*), 3.12 (m, 84H, TIPS C*H*/CH_3) ppm.

^{11}B{1H} NMR (d_5-bromobenzene, 128.36 MHz, 298 K): δ = −15.50 (s, $B(C_6F_5)_4$ *B*) ppm.

^{13}C{^{1}H} NMR (d_5-bromobenzene, 100.61 MHz, 298 K): δ = no resonances observed.

^{19}F{^{1}H} NMR (d_5-bromobenzene, 376.50 MHz, 298 K): δ = −130.96 (s, $B(C_6F_5)_4$ *F*), −161.67 (t, J_{FF} = 21.0 Hz, $B(C_6F_5)$ *F*), −165.32 (m , $B(C_6F_5)_4$ *F*) ppm.

^{29}Si{^{1}H} NMR (d_5-bromobenzene, 79.47 MHz, 298 K): δ = −97.73 (s, TIPS *Si*) ppm.

UV-Vis-NIR (*o*DFB, 0.28 mM): λ_{max} (ν/cm^{-1}; ε/M^{-1}cm^{-1}) = 784 (12,755; 793), 496 (20,161; 2,715), 396 (25,253; 5,163), 324 (30,864; 4,231).

FT-IR (ATR, microcrystalline): cm^{-1} = 411 (s), 421 (m), 438 (m), 448 (m), 475 (w), 491 (s), 522 (w), 543 (m), 573 (vw), 600 (w), 610 (w), 639 (vw), 660 (vw), 678 (vw), 707 (vs), 725 (vs), 756 (vw), 769 (w), 816 (s), 880 (vw), 921 (m), 937 (m), 978 (vw), 1,013 (w), 1,083 (vw), 1,192 (vs), 1,225 (s), 1,274 (m), 1,315 (vs), 1,340 (vs), 1,373 (s), 1,383 (m), 1,412 (s), 1,459 (vw), 1,513 (vw), 1,642 (s), 2,721 (vs), 2,869 (w), 2,890 (s), 2,947 (w).

*Synthesis of [{U^{IV}(COT††)(μ-F)$_2$}$_3$] (**7**)*

Small scale: *o*DFB (2 mL) was added to a 4 mL borosilicate glass vial containing a mixture of **6** (0.027 g, 0.015 mmol, 1 equiv.) and [*n*Bu_4N][PF_6] (0.006 g, 0.015 mmol, 1 equiv.). The mixture was shaken briefly to give a dark-brown solution. The dark-brown solution was concentrated to *ca*. 1 mL. Storage at –35°C gave a mixture of red crystals of **7** and green crystals of **4**, which were identified by SCXRD.

^{1}H NMR (d_5-bromobenzene, 400.07 MHz, 298 K): δ = 6.63 (s, 1H, COT C*H*), 6.35 (d, J_{HH} = 10.9 Hz, 1H, COT C*H*), 6.07 (d, J_{HH} = 10.7 Hz, 1H, COT C*H*), 2.79 (m ,8H, [*n*Bu_4N] NCH_2), 1.39 (m, [*n*Bu_4N] CH_2 and TIPS C*H*/CH_2), 1.07 (m, 8H, [*n*Bu_4N][$B(C_6F_5)_4$] CH_3), −0.75 (br. m, [U^{IV}(COT††)$_2$] and [{U^{IV}(COT††)(μ-F)$_2$}$_3$] COT C*H* and TIPS C*H*/CH_3) ppm.

^{11}B{^{1}H} NMR (d_5-bromobenzene, 128.27 MHz, 298 K): δ = −17.29 (s, [$B(C_6F_5)_4$] *B*) ppm.

^{13}C{^{1}H} NMR (d_5-bromobenzene, 100.61 MHz, 298 K): δ = no resonances observed.

^{19}F{^{1}H} NMR (d_5-bromobenzene, 376.50 MHz, 298 K): δ = −131.39 (s, [$B(C_6F_5)_4$] *F*), −161.58 (t, J_{FF} = 20.9 Hz, [$B(C_6F_5)_4$] *F*), −165.54 (m, [$B(C_6F_5)_4$] *F*) ppm.

^{29}Si{^{1}H} NMR (d_5-bromobenzene, 79.47 MHz, 298 K): δ = no resonances observed.

^{31}P{^{1}H} NMR (d_5-bromobenzene, 161.95 MHz, 298 K): δ = no resonances observed.

Larger scale: Observation of all ^{19}F and ^{31}P-containing species required higher sample concentrations than possible using d_5-bromobenzene, so the reaction was repeated on a larger scale using *o*DFB as the solvent. *o*DFB (0.5 mL) was added to a silanized J. Young valve-appended NMR tube containing a mixture of **6** (0.100 g, 0.057 mmol, 1 equiv.) and [nBu$_4$N][PF$_6$] (0.022 g, 0.057 mmol, 1 equiv.). The reaction was allowed to reach completion as determined by ^1H NMR spectroscopy, after which a few drops of H_8-THF were added to the NMR tube to form the THF–PF_5 complex which could be observed definitively. Note the presence of POF_3 is due to the reaction of PF_5 with the surface of the borosilicate glass tube despite pre-silanization.

$^{19}F\{^1H\}$ NMR (*o*DFB, 376.50 MHz, 298 K): δ = −33.83 (d, J_{FP} = 1399.30 Hz, PF_3 $\underline{F}$), −67.76 (br. s, PF_5 $\underline{F}$), −73.63 (d, J_{FP} = 707.60 Hz, PF_5·THF $\underline{F}$), −89.91 (d, J_{FP} = 1070.50 Hz, POF_3 $\underline{F}$) ppm.

$^{31}P\{^1H\}$ NMR (*o*DFB, 161.95 MHz, 298 K): δ = 105.12 (q, J_{PF} = 1399.40 Hz, PF_3, $\underline{P}$), −33.94 (q, J_{PF} = 1066.72 Hz, POF_3 $\underline{P}$), −134.85 (sext, J_{PF} = 805.00 Hz, PF_5, $\underline{P}$), −146.80 (p, J_{PF} = 696.26 Hz, PF_5·THF $\underline{P}$) ppm.

*Synthesis of [Li(12-crown-4)$_2$][U^{III}(COT)$_2$] (**8**)*

When the synthesis of **2** was performed using 2 equivalents of 12-crown-4, several crystals of **8** were isolated alongside crystals of **2**. Complex **8** could not be isolated in pure form using this method, and so complex **2** was used for all further studies.

*Synthesis of [Ce^{IV}(COT††)$_2$] (**9**)*

THF (15 mL) was added to a pre-cooled (–98°C) Schlenk vessel containing a mixture of [Ce^{III}I$_3$(THF)$_4$] (0.809 g, 1.0 mmol, 1 equiv.) and K$_2$COT†† (0.990 g, 2.0 mmol, 2 equiv.) and a PTFE-coated stirrer bar. The mixture was allowed to warm to room temperature and stirred overnight (18 hours) to give a green solution over pale solids, presumed to be KI. The solids were allowed to settle, and the supernatant was filtered through a glass microfiber filter disc into a new Schlenk vessel. Volatiles were removed under vacuum (10^{-3} mbar), which gave a green solid of putative [Ce^{III}(COT††)$_2$K], which was used for subsequent steps without further purification.

THF (15 mL) was added to a pre-cooled (–98°C) Schlenk vessel containing a mixture of putative [Ce^{III}(COT††)$_2$K] and Ag^{I}I (0.258 g, 1.1 mmol, 1.1 equiv.). The mixture was allowed

to warm to room temperature and stirred overnight (18 hours) to give a dark purple solution over pale solids, presumed to be Ag^0. Volatiles were removed under vacuum (10^{-3} mbar), which gave a green solid. *n*-Hexane (15 mL) was added, the purple mixture was briefly (<1 min.) heated to boiling, and the pale solids were manually freed from the vessel sidewalls. The solids were allowed to settle while the mixture cooled to room temperature, and the supernatant was filtered through a glass microfiber filter disc into a new Schlenk vessel. The clear purple solution was concentrated to *ca.* 1 mL. Storage at –35°C gave a single crystalline crop of **9**. The product was isolated by decanting the supernatant while cold, and the crystals were dried under vacuum (10^{-3} mbar) for 4 hours to give **9** in low isolated crystalline yield (0.441 g, 45%).

Elemental analysis on $C_{52}H_{96}Si_4Ce$ calc. (%): C = 64.14, H = 9.94, N = 0.00; found (%): C = 63.19, H = 10.13, N = 0.00.

^{1}H NMR (d_6-benzene, 400.07 MHz, 298 K): δ = 6.32 (m, 4H, COT C*H*), 5.96 (s, 4H, COT C*H*), 5.71 (s, 4H, COT C*H*), 1.45 (d, J_{HH} = 3.5 Hz, 36H, TIPS CH_3), 1.43 (d, J_{HH} = 3.5 Hz, 36H, TIPS CH_3), 2.01 (sept, J_{HH} = 7.5 Hz, 12H, TIPS C*H*) ppm.

^{13}C{^{1}H} NMR (d_6-benzene, 100.61 MHz, 298 K): δ = 121.99 (s, COT *C*H), 120.08 (s, COT *C*H), 118.97 (s, COT *C*H), 19.87 (s ,TIPS *C*H_3), 19.84 (TIPS *C*H_3), 14.85 (s, TIPS *C*H).

^{29}Si{^{1}H} NMR (d_6-benzene, 79.47 MHz, 298 K): δ = 0.27 (s, TIPS *Si*) ppm.

UV-Vis-NIR (THF, 0.20 mM): λ_{max} (ν/cm^{-1}; ε/M^{-1}cm^{-1}) = 659 (15,175; 1,733), 525 (19,048; 8,195), 307 (32,573; 3,263).

FT-IR (ATR, microcrystalline): cm^{-1} = 419 (w), 436 (w), 448 (w), 462 (w), 471 (vw), 491 (vw), 512 (vw), 573 (vw), 635 (vw), 664 (vw), 736 (vw), 754 (s), 783 (m), 818 (s), 857 (m), 880 (vw), 902 (s), 923 (m), 937 (w), 962 (m), 995 (m), 1,013 (vw), 1,026 (w), 1,075 (s), 1,180 (s), 1,217 (s), 1,250 (s), 1,297 (vs), 1,338 (vs), 1,365 (s), 1,383 (m), 1,406 (vs), 1,459 (m), 2,715 (vs), 2,721 (vs), 2,754 (vs), 2,861 (vw), 2,888 (m), 2,920 (w), 2,939 (w).

*Synthesis of [Li(TMEDA)$_2$][Ce^{III}(COT††)$_2$] (**10**)*

THF (15 mL) was added to a Schlenk vessel containing a mixture of [Ce^{IV}(COT††)$_2$] (1.061 g, 1.09 mmol, 1 equiv.) and Li^0 metal (0.008 g, 1.2 mmol, 1.1 equiv.) and a glass-coated stirrer bar. The mixture was stirred for five days to give a green solution. The supernatant was filtered through a glass microfiber filter disc into a new Schlenk vessel, and the volatiles were removed under vacuum (10^{-3} mbar), which gave a green solid. TMEDA (2 mL) was added, and the mixture was stored at –35°C, yielding large brown crystals. The crude product was isolated by decanting the supernatant while cold, the solid was washed with *n*-

hexane (10 mL) and the crystals were dried under vacuum (10^{-3} mbar) for 4 hours. Mesitylene (2 mL) was added, briefly heated and storage at –35°C gave a single crystalline crop of **10**. The product was isolated by decanting the supernatant while cold, and the crystals were washed with *n*-hexane (5 mL) and dried under vacuum (10^{-3} mbar) for 4 hours to give **10** in low isolated crystalline yield (0.444 g, 31%).

Elemental analysis on $C_{64}H_{128}Si_4N_4LiCe{\cdot}(C_9H_{12})$ calc. (%): C = 65.76, H = 10.58, N = 4.20; found (%): C = 65.78, H = 10.81, N = 4.12. *Note*, the mesitylene is present in the crystal structure of **10**, and elemental analysis results show incomplete desolvation under vacuum.

^{1}H NMR (d_8-THF, 400.07 MHz, 298 K): δ = 6.74 (s, 4H, mesitylene C*H*), 2.79 (s, 12H, $\bar{\nu}$ = 33.4 Hz, COT CH), 2.31 (s, TMEDA CH_2), 2.22 (s, mesitylene CH_3), 2.22 (br. s, TIPS C*H*/CH_3), 2.15 (s, TMEDA CH_3), 2.08 (br. s, TIPS C*H*/CH_3) ppm.

^{13}C{^{1}H} NMR (d_8-THF, 100.61 MHz, 298 K): δ = 138.24 (s, mesitylene *C*(CH_3)), 127.74 (s, mesitylene *C*H), 59.09 (s, TMEDA *C*H_2), 46.37 (s, TMEDA *C*H_3), 21.51 (s, mesitylene *C*H_3).

^{29}Si{^{1}H} NMR (d_8-THF, 79.47 MHz, 298 K): δ = −4.43 (s, TIPS *Si*) ppm.

UV-Vis-NIR (THF, 0.25 mM): λ_{max} (ν/cm^{-1}; ε/M^{-1}cm^{-1}) = 666 (15,015; 1,007), 340 (29,411; 3,891), 308 (32,468; 6,649).

FT-IR (ATR, microcrystalline): cm^{-1} = 417 (w), 438 (w), 471 (w), 491 (w), 514 (vw), 580 (vw), 612 (w), 637 (vw), 658 (vw), 688 (m), 721 (vw), 752 (s), 773 (m), 787 (m), 836 (w), 855 (s), 882 (vw), 931 (vw), 945 (m), 964 (m), 989 (m), 1,013 (vw), 1,038 (vw), 1,071 (m), 1,097 (s), 1,128 (s), 1,159 (s), 1,184 (vs), 1,213 (s), 1,248 (m), 1,289 (m), 1,361 (s), 1,379 (s), 1,412 (vs), 1,459 (vw), 607 (s), 2,709 (vs), 2,717 (vs), 2,750 (vs), 2,797 (s), 2,857 (vw), 2,886 (w), 2,916 (w), 2,937 (w), 3,029 (vs).

S2. ATR-IR / FTIR spectroscopy

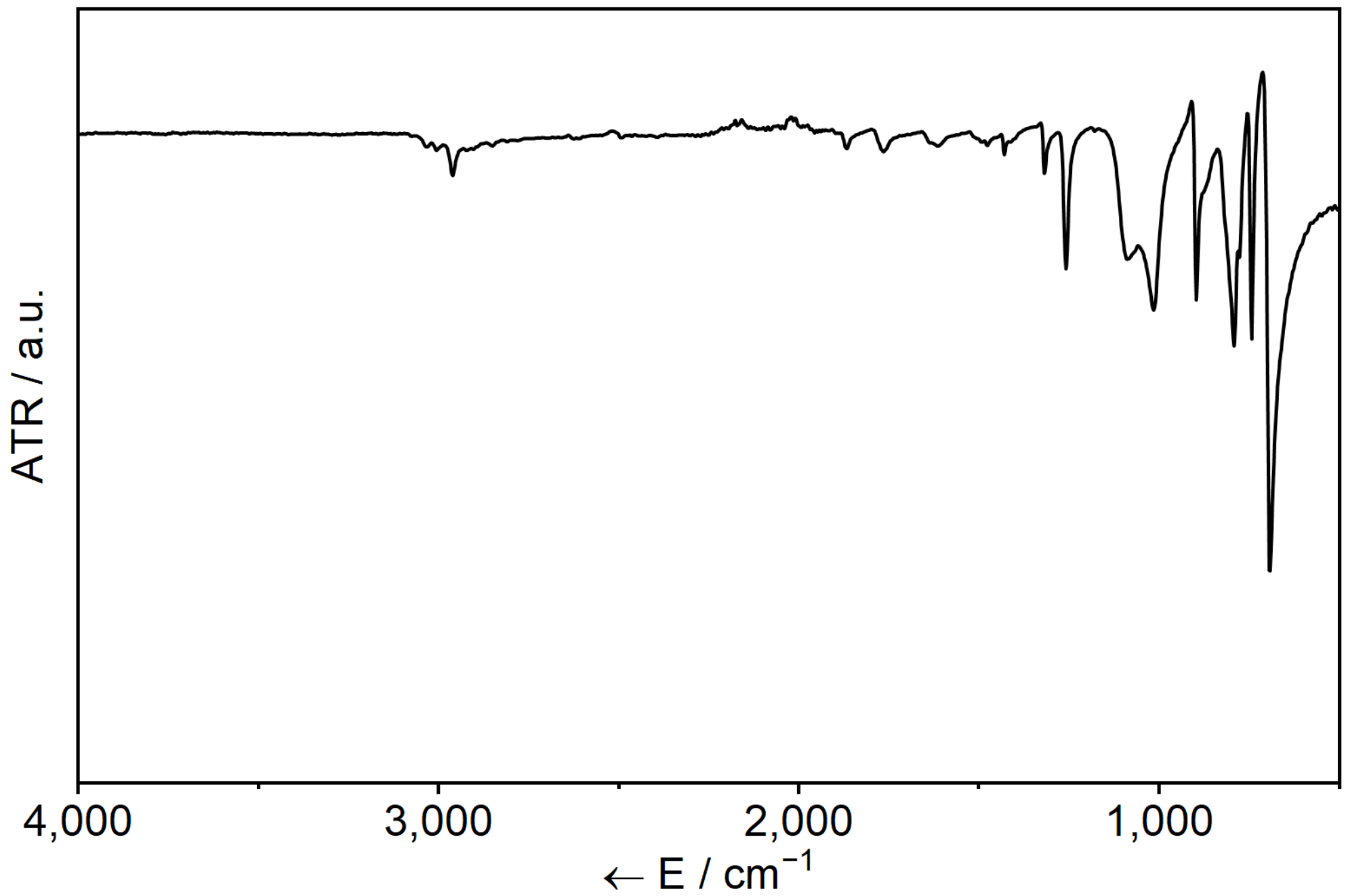

Figure S1. ATR-IR spectrum of microcrystalline **1**.

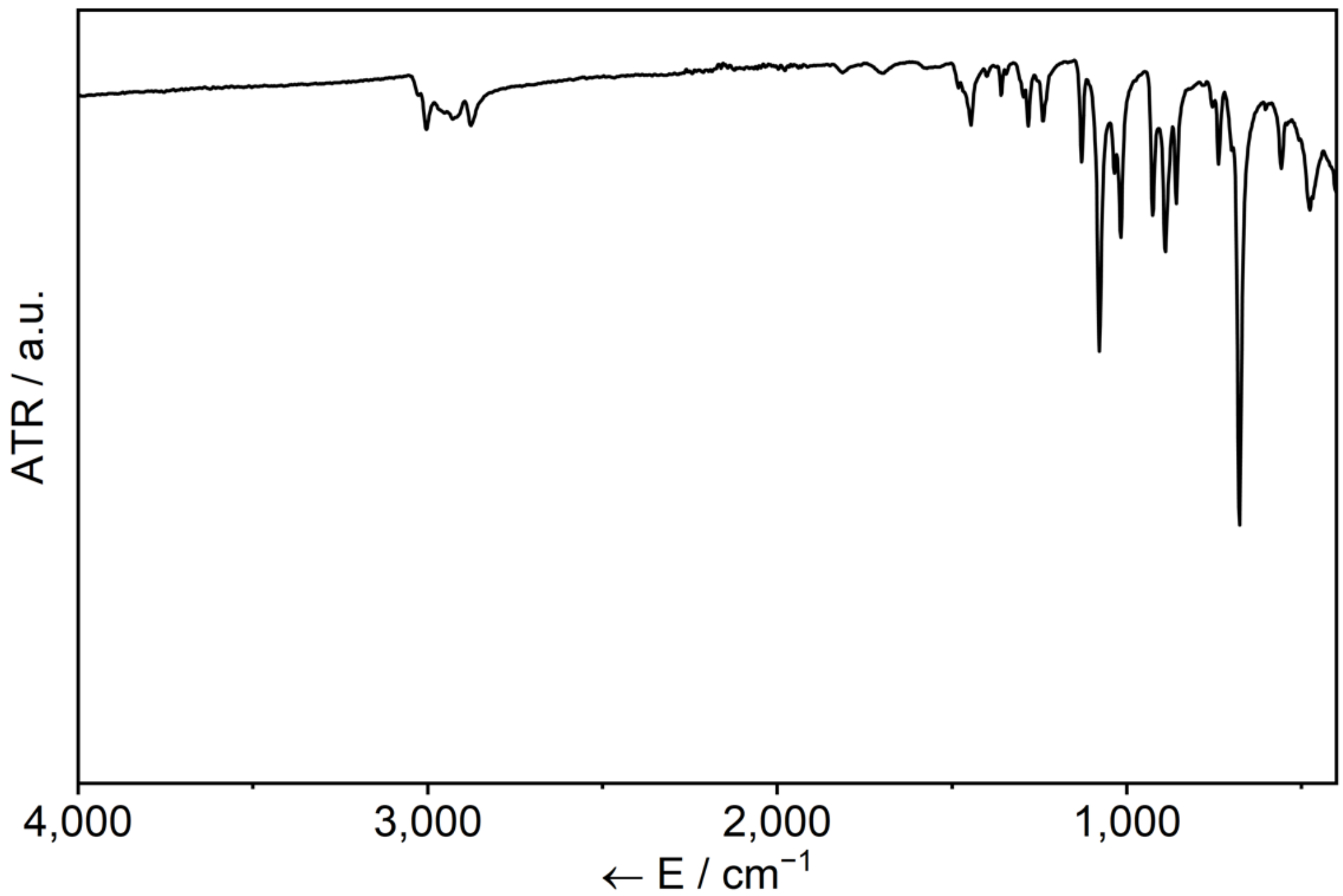

Figure S2. ATR-IR spectrum of microcrystalline **2**.

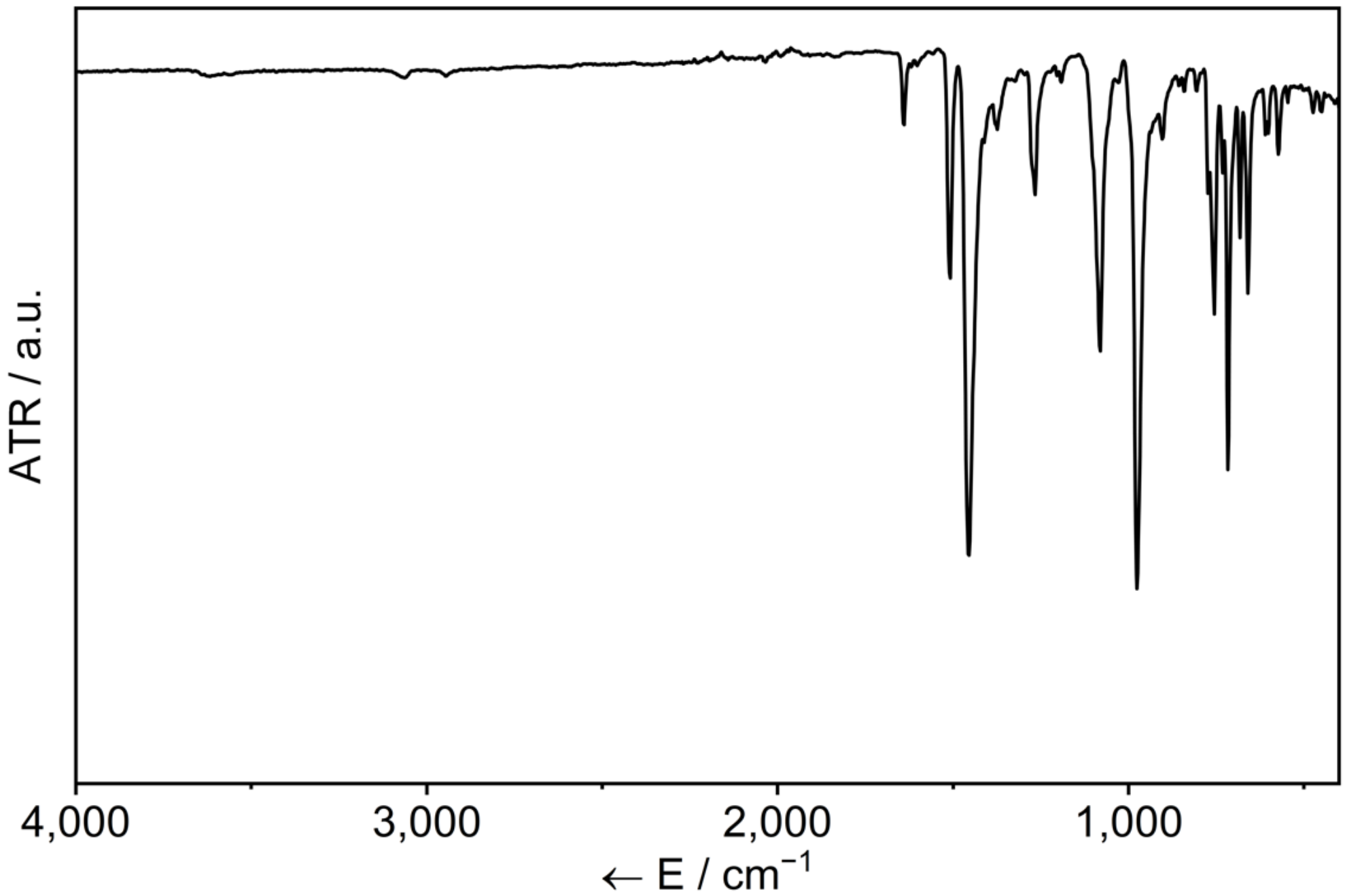

Figure S3. ATR-IR spectrum of microcrystalline **3**.

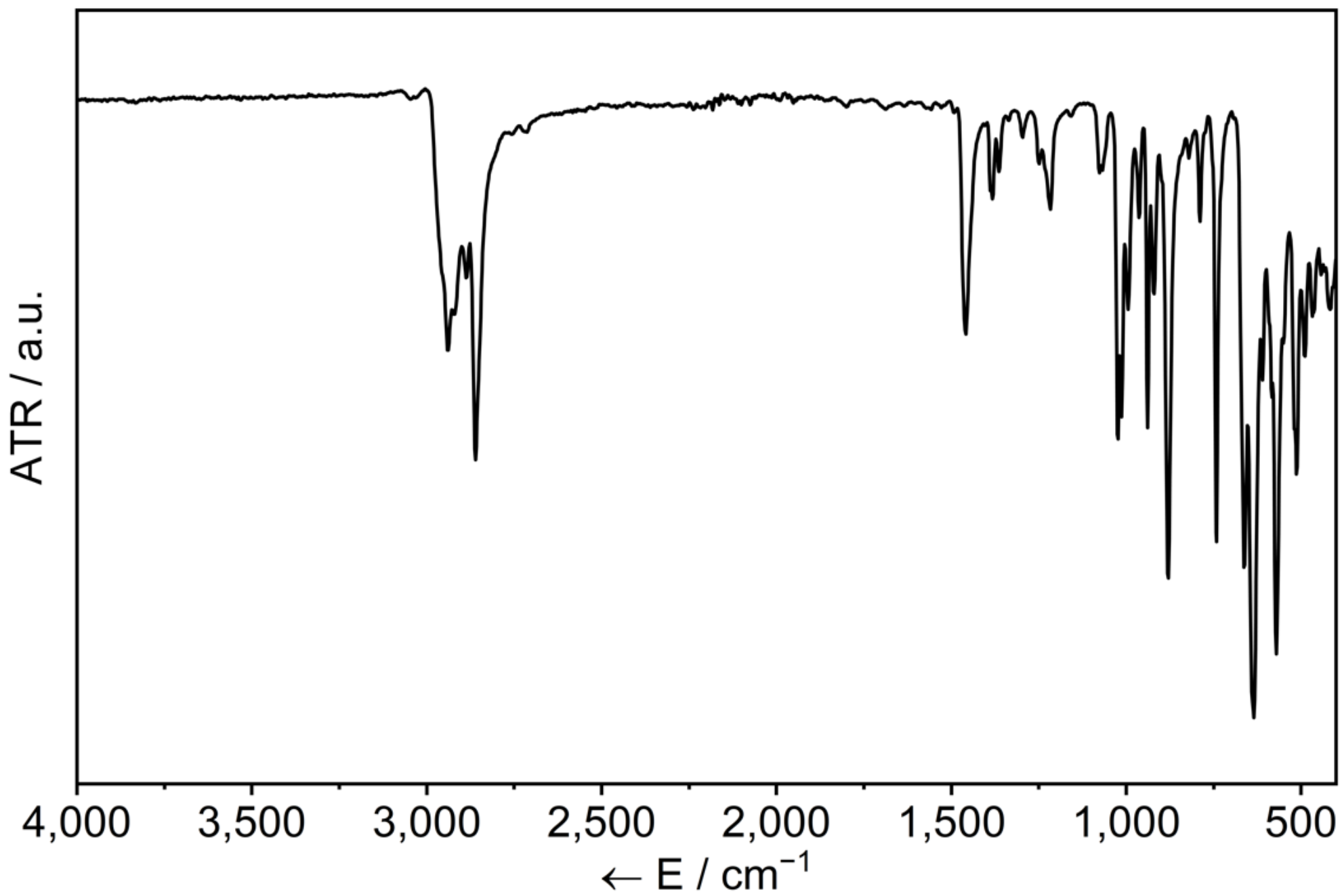

Figure S4. ATR-IR spectrum of microcrystalline **4**.

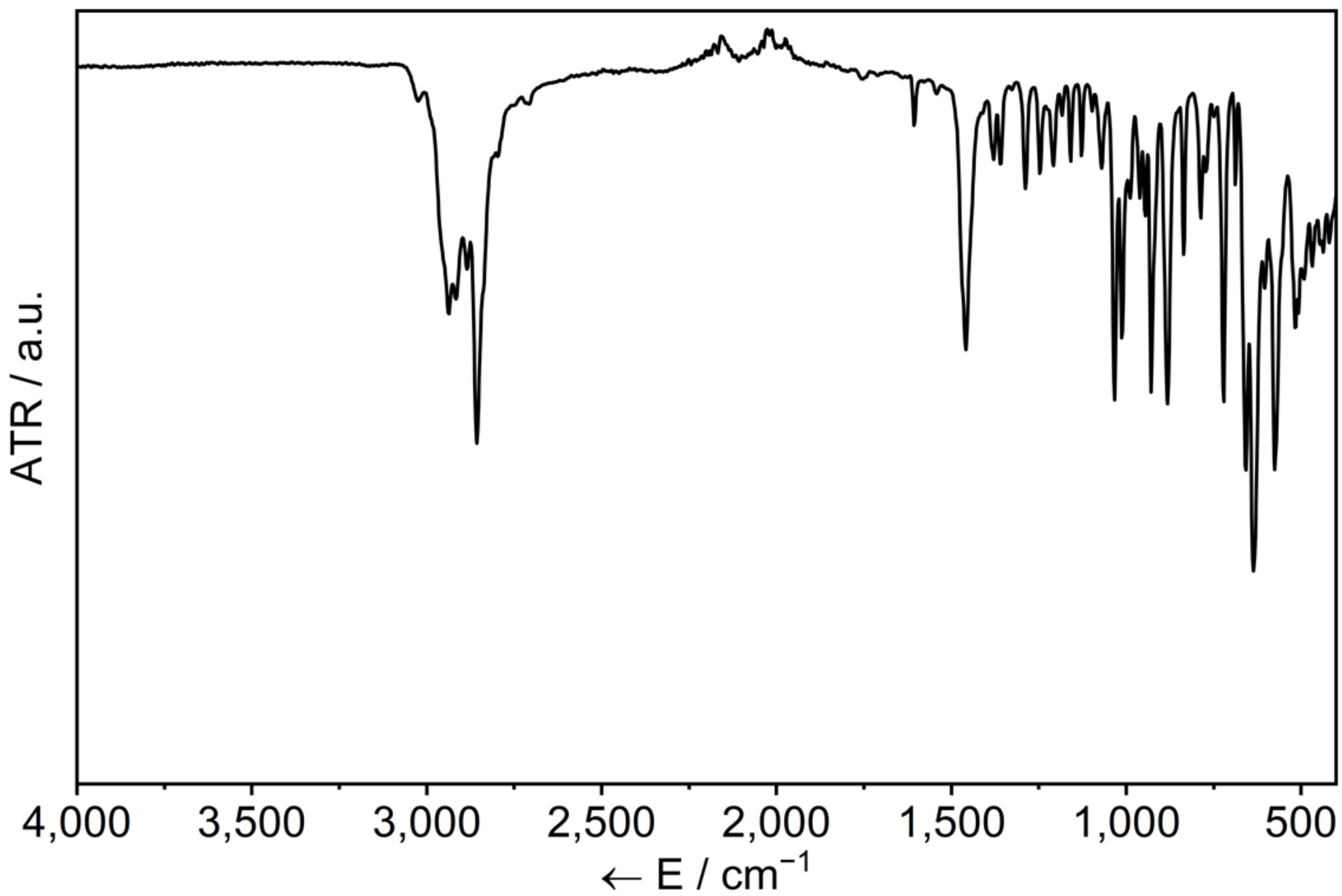

Figure S5. ATR-IR spectrum of microcrystalline **5**.

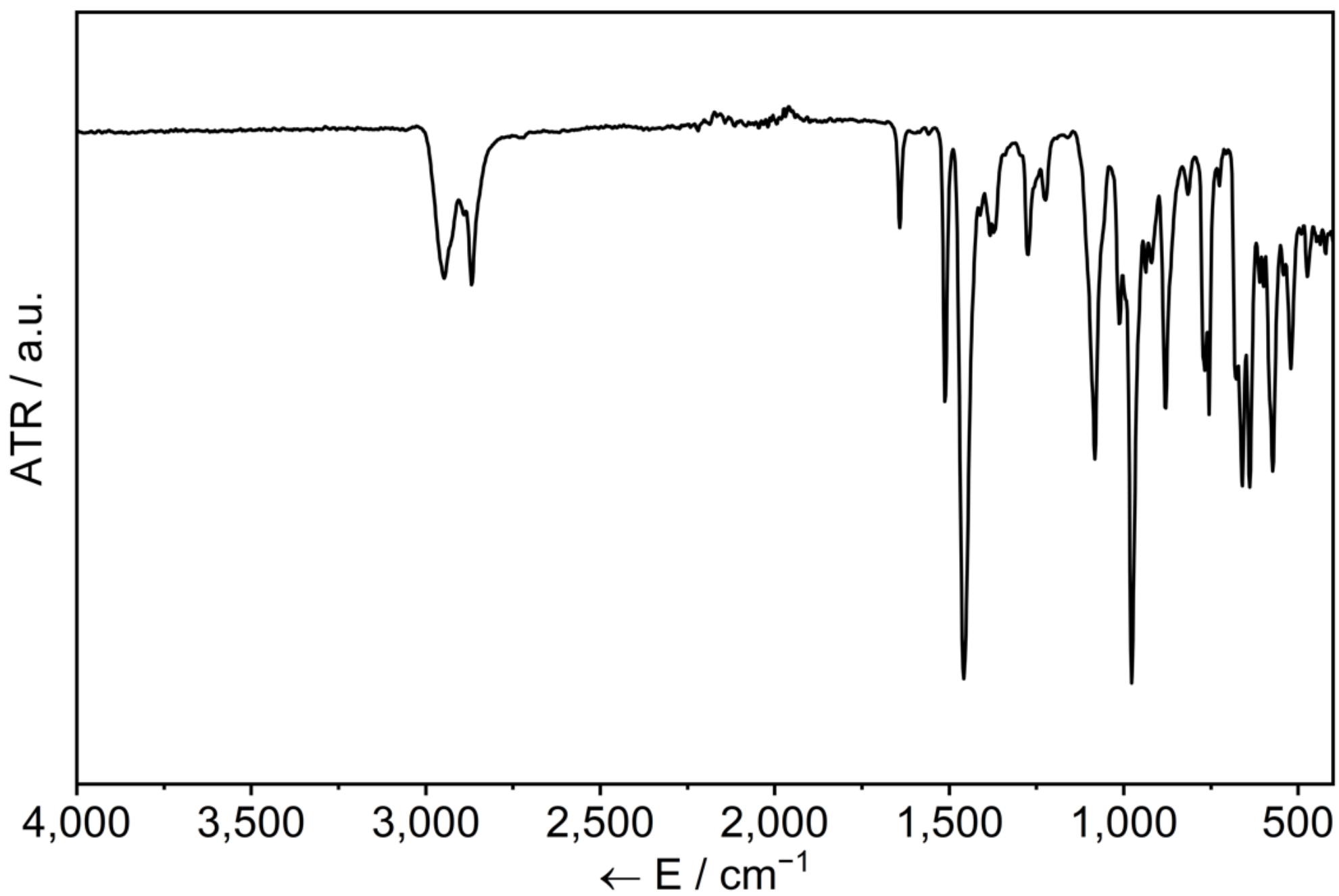

Figure S6. ATR-IR spectrum of microcrystalline **6**.

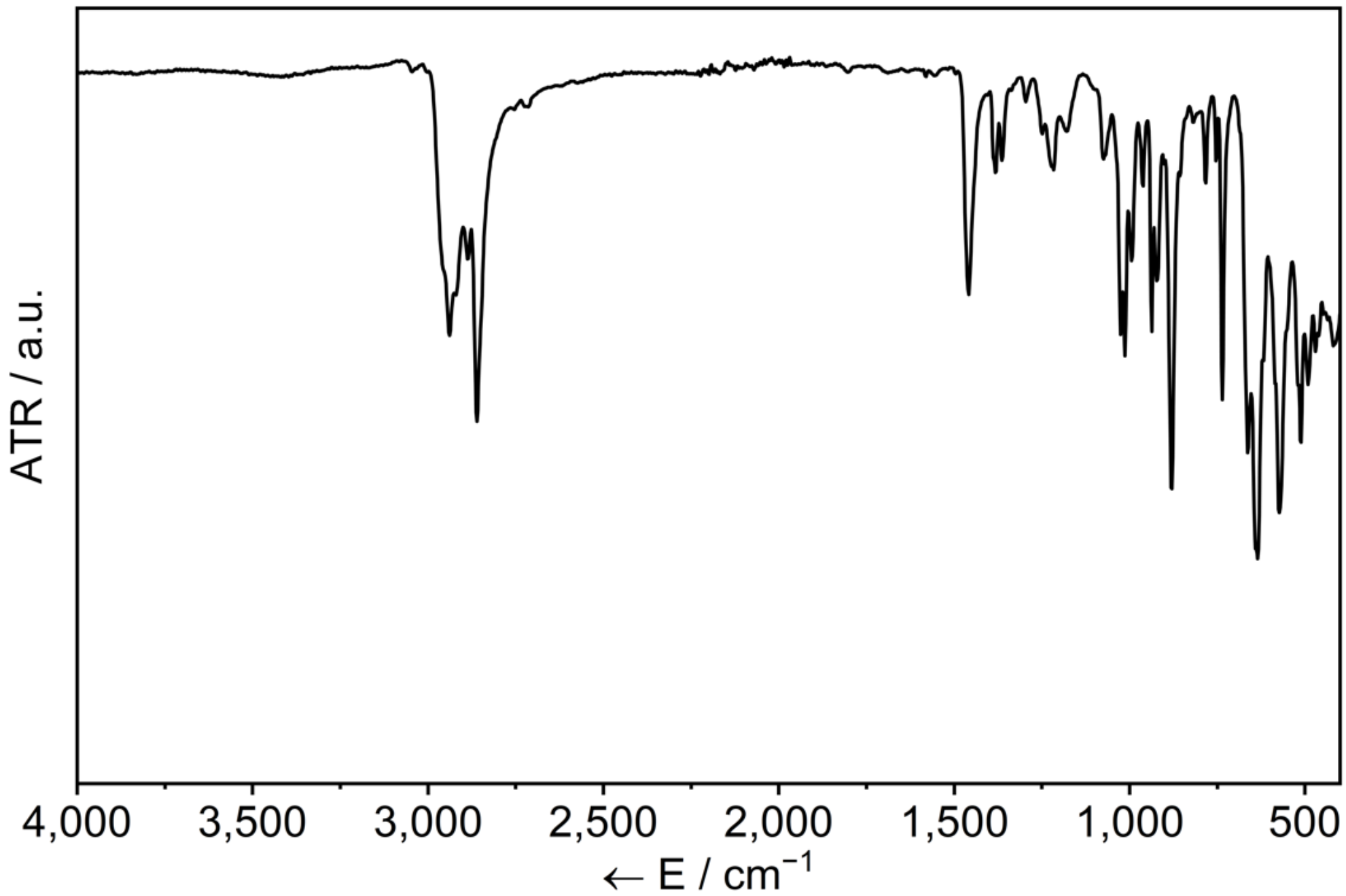

Figure S7. ATR-IR spectrum of microcrystalline **9**.

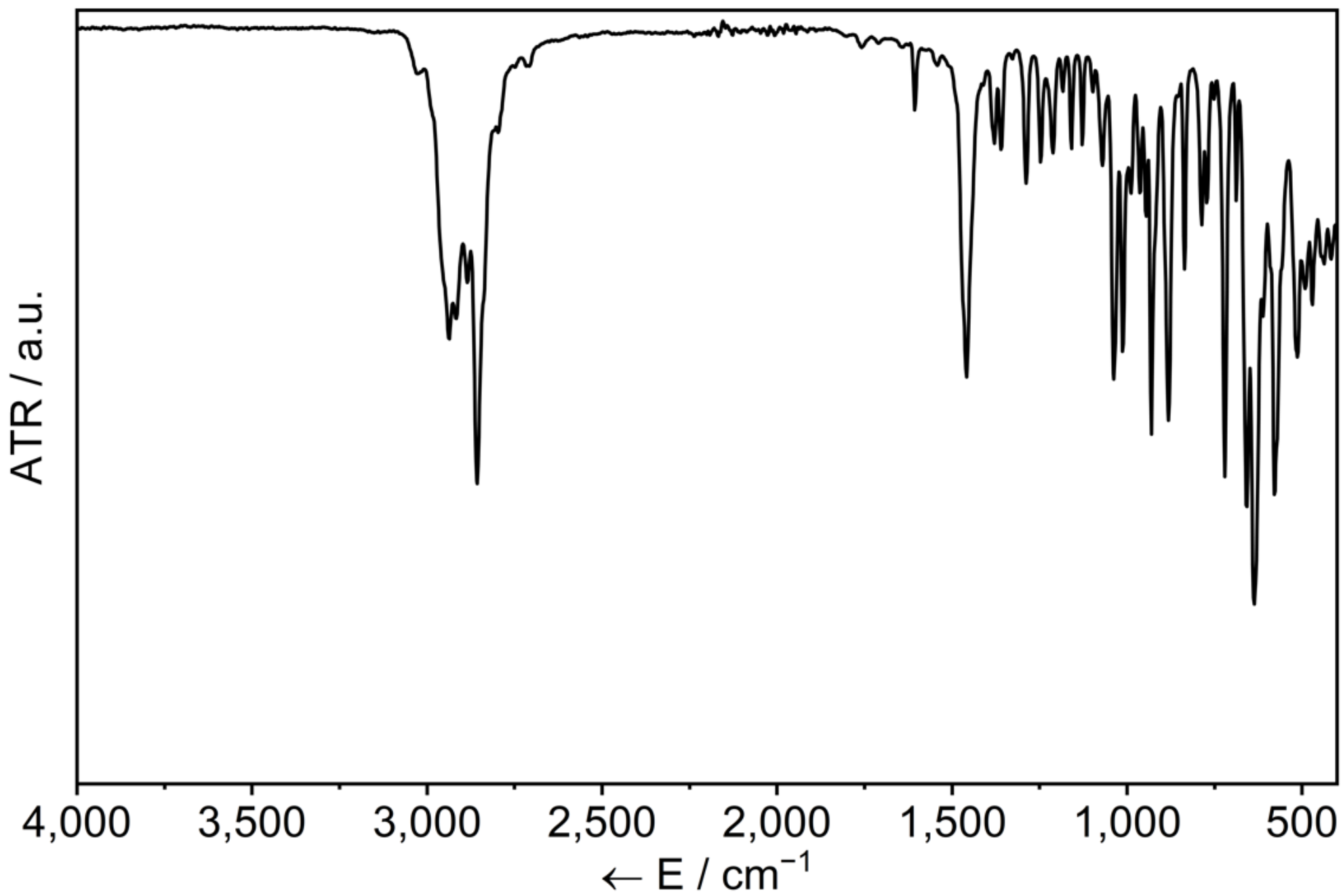

Figure S8. ATR-IR spectrum of microcrystalline **10**.

S3. X-ray diffraction

General considerations – single crystal X-ray diffraction (SCXRD)

For details on how crystals were grown, please consult Section 1. Crystals coated in Cargille NVH immersion oil were mounted on a MiTeGen micromount. Data for **1**, **2**$^{\beta}$, **3**, **4**, **5**, **7**, **8**, and **9** were collected using a Rigaku XtaLAB synergy DW diffractometer, equipped with a PhotonJet Cu Kα (λ = 1.54184 Å) or a Mo Kα (λ = 0.71073 Å) radiation source, using a 4-circle κ goniometer, a HyPix-6000HE hybrid pixel array detector operating in shutterless mode and an Oxford Cryosystems Cryostream 800 nitrogen flow gas system at a temperature of 100K. Data for **2** and **6** were collected using a Rigaku FR-X DW diffractometer, equipped with an FR-X high-intensity rotating anode Cu Kα radiation source (λ = 1.54184 Å) and VariMAXTM microfocus optics, using an AFC-11 4-circle κ goniometer, a HyPix-6000HE hybrid pixel array detector operating in shutterless mode and an Oxford Cryosystems Cryostream 800 nitrogen flow gas system at a temperature of 100 K. Intensities were integrated from data recorded from ω, or ω and φ rotation, at the frame widths and exposure times outlined in **Table S2**. The crystal data for all complexes are outlined in **Table S3** to **Table S5**.

Table S2. Data collection parameters and CCDC reference codes for all structures herein.

	Formula	Frame width (°)	Exposure time (s)	CCDC ref.
**1**	$[U^{IV}(COT)_2]$	0.50	0.05	2503922
**2**	$[Li(12\text{-crown-}4)(THF)][U^{III}(COT)_2]$	0.50	0.25 / 0.50	2503923
**3**	$[U^{V}(COT)_2][B(C_6F_5)_4]\cdot 1.5(C_6H_4F_2)$	0.50	3.00 / 10.00	2503924
**4**	$[U^{IV}(COT^{\dagger\dagger})_2]$	0.50	0.05 / 0.12	2481662
**5**	$[Li(TMEDA)_2][U^{III}(COT^{\dagger\dagger})_2]\cdot(C_9H_{12})$	0.50	35.00	2481664
**6**	$[U^{V}(COT^{\dagger\dagger})_2][B(C_6F_5)_4]\cdot(C_6H_{14})$	0.50	2.00 / 8.00	2481663
**7**	$[\{U^{IV}(COT^{\dagger\dagger})(\mu\text{-F})_2\}_3]$	0.50	0.10 / 0.40	2522902
**2**$^{\beta}$	$[Li(12\text{-crown-}4)(THF)][U^{III}(COT)_2]$	0.50	2.00 / 7.00	2521131
**8**	$[Li(12\text{-crown-}4)_2[U^{III}(COT)_2]$	0.50	1.50 / 5.00	2503925
**9**	$[Ce^{IV}(COT^{\dagger\dagger})_2]$	0.50	0.27 / 1.06	2481660
**10**	$[Li(TMEDA)_2][Ce^{III}(COT^{\dagger\dagger})_2]\cdot(C_9H_{12})$	0.50	25.00	2481666
	$[nNBu_4][SbF_6]$	0.50	0.30 / 0.08	2556163

CrysAlisPro[7] was used for final unit cell determination and parameters were refined from the observed positions of all strong reflections in each data set. An analytical absorption correction was applied[7]. The Olex2 (ref.[8]) GUI was used for structure solution and refinement along with the ShelX software packages[9,10]. The structures were solved using

ShelXT[9]; the datasets were refined by ShelXL[10] using full-matrix least-squares on all unique F^2 values, with anisotropic displacement parameters for all non-hydrogen atoms, and with constrained riding hydrogen geometries; U_{iso}(H) was set at 1.2 (1.5 for methyl groups if applicable) times U_{eq} of the parent atom. The largest features in final difference syntheses were close to heavy atoms and were of no chemical significance. Olex2 (ref.[8]) combined with POV-Ray[11], Gimp[12], and InkScape[13], were employed for molecular graphics. The following CCDC references contain the supplementary crystal data for this article: **1** (2503922), **2** (2503923), **2**$^{\beta}$ (2521131), **3** (2503924), **4** (2481662), **5** (2481664), **6** (2481663), **7** (2522902), **8** (2503925), **9** (2481660), **10** (2481666), and [nBu$_4$N][SbF$_6$] (2556163). These data can be obtained free of charge from the Cambridge Crystallographic Data Centre via www.ccdc.cam.ac.uk/data_request/cif.

The combined error from two individual metrics that have their own associated errors (estimated standard deviation, or standard uncertainty used interchangeably here) can be calculated as the root of the sum of the square of each error (**Eq. S2**). This is not strictly appropriate for combining more than two individual errors[14].

$$\sigma_{tot} = \sqrt{{\sigma_1}^2 + {\sigma_2}^2}$$

Eq. S2.

The combined error for the numerical average for multiple (independent) bond lengths, such as the five independent M–C bonds in an M–($\eta^5C_5H_5$) complex, is calculated using the alternate weighted standard deviation from Parsons and Clegg (**Eq. S3**)[14].

$$\sigma_{tot} = \frac{1}{\sqrt{\sum_{1\to n} W_n}} \qquad W_n = 1/{\sigma_n}^2$$

Eq. S3.

Table S3. Crystallographic data for **1**, **2**, and **3**.

	**1**	**2**	**3**
Identification code	ccapg458	lcapg151	ccapg423
Formula	$C_{16}H_{16}U$	$C_{28}H_{40}LiO_5U$	$C_{49}H_{22}BF_{23}U$
Fw	446.32	701.57	1296.50
Temperature / K	99.99(10)	100.00(10)	100.00(10)
Crystal system	monoclinic	monoclinic	triclinic
Space group	$P2_1/c$	$P2_1/c$	$P\overline{1}$
a / Å	7.01476(15)	8.77010(10)	13.6552(2)
b / Å	8.6759(2)	25.4110(2)	16.8748(3)
c / Å	10.4784(2)	12.19680(10)	20.3859(3)
α / °	90	90	77.2700(10)
β / °	98.753(2)	94.1790(10)	89.1180(10)
γ / °	90	90	84.3150(10)
Volume / $Å^3$	630.28(2)	2710.92(4)	4559.39(13)
Z	2	4	4
$\rho_{calc}g$ / cm^3	2.352	1.719	1.889
μ / mm^{-1}	35.999	17.126	11.222
F(000)	408	1372	2480
Crystal size / mm^3	0.04 × 0.03 × 0.02	0.193 × 0.069 × 0.062	0.14 × 0.05 × 0.03
Radiation	Cu Kα (λ = 1.54184)	Cu Kα (λ = 1.54184)	Cu Kα (λ = 1.54184)
2Θ range / °	13.31 to 140.14	6.96 to 140.12	4.44 to 140.14
Index ranges	−7 ≤ h ≤ 8, −10 ≤ k ≤ 9, −12 ≤ l ≤ 12	−10 ≤ h ≤ 9, −30 ≤ k ≤ 30, −13 ≤ l ≤ 14	−16 ≤ h ≤ 16, −20 ≤ k ≤ 20, −24 ≤ l ≤ 24
No. reflections	4293	14761	81008
Unique reflections	1203 [R_{int} = 0.0718, R_{sigma} = 0.0729]	5143 [R_{int} = 0.0383, R_{sigma} = 0.0373]	17270 [R_{int} = 0.0682, R_{sigma} = 0.0464]
Data / restraints / parameters	1203/154/79	5143/1221/389	17270/3731/1453
GOOF on F^2	1.010	1.072	1.043
Final R indexes [I ≥ 2σ (I)] [A]	R_1 = 0.0328, wR_2 = 0.0759	R_1 = 0.0305, wR_2 = 0.0820	R_1 = 0.0475, wR_2 = 0.1218
Final R indexes [all data]	R_1 = 0.0533, wR_2 = 0.0874	R_1 = 0.0319, wR_2 = 0.0832	R_1 = 0.0565, wR_2 = 0.1264
Largest diff. (peak / hole) / e $Å^{-3}$	2.075/−1.536	1.726/−1.494	2.331/−2.246

[A] $R = \sum||F_O| - |F_C||/\sum|F_O|$; $R_W = [\sum w(F_O^2 - F_C^2)^2/\sum w(F_O^2)^2]^{0.5}$; $S = [\sum w(F_O^2 - F_C^2)^2/(\text{no. data} - \text{no. params})]^{0.5}$ for all data.

Table S4. Crystallographic data for **4**, **5**, and **6**.

	**4**	**5**	**6**
Identification code	ccapg238	ccapg269	rcapg183
Formula	$C_{52}H_{96}Si_4U$	$C_{73}H_{140}LiN_4Si_4U$	$C_{79}H_{103}BF_{20}Si_4U$
Fw	1071.67	1431.21	1793.81
Temperature / K	100.00(10)	99.97(15)	99.98(12)
Crystal system	monoclinic	triclinic	monoclinic
Space group	$C2/c$	$P\overline{1}$	$P2_1/c$
a / Å	22.4483(2)	13.9014(2)	29.96026(18)
b / Å	11.59460(10)	14.8767(3)	18.94501(10)
c / Å	22.7177(2)	21.5551(4)	28.56509(18)
α / °	90	88.8714(15)	90
β / °	114.1000(10)	76.2858(15)	98.4083(6)
γ / °	90	79.5075(16)	90
Volume / Å^3	5397.53(9)	4256.96(14)	16039.20(17)
Z	4	2	8
ρ_{calc}g / cm^3	1.319	1.117	1.486
μ / mm^{-1}	9.532	1.999	7.035
F(000)	2224	1514	7280
Crystal size / mm^3	0.189 × 0.165 × 0.148	0.256 × 0.216 × 0.178	0.12 × 0.06 × 0.05
Radiation	Cu Kα (λ = 1.54184)	Mo Kα (λ = 0.71073)	Cu Kα (λ = 1.54184)
2Θ range / °	8.53 to 140.14	4.00 to 52.04	5.54 to 140.14
Index ranges	−27 ≤ h ≤ 27, −10 ≤ k ≤ 14, −27 ≤ l ≤ 27	−17 ≤ h ≤ 17, −18 ≤ k ≤ 18, −26 ≤ l ≤ 26	−36 ≤ h ≤ 36, −18 ≤ k ≤ 23, −34 ≤ l ≤ 34
No. reflections	40660	67677	114344
Unique reflections	5131 [R_{int} = 0.0557, R_{sigma} = 0.0317]	16754 [R_{int} = 0.0449, R_{sigma} = 0.0417]	30347 [R_{int} = 0.0315, R_{sigma} = 0.0287]
Data / restraints / parameters	5131/0/270	16754/240/783	30347/0/1941
GOOF on F^2	1.013	1.025	1.024
Final R indexes [I ≥ 2σ (I)] [A]	R_1 = 0.0238, wR_2 = 0.0594	R_1 = 0.0290, wR_2 = 0.0595	R_1 = 0.0280, wR_2 = 0.0667
Final R indexes [all data]	R_1 = 0.0242, wR_2 = 0.0596	R_1 = 0.0373, wR_2 = 0.0615	R_1 = 0.0313, wR_2 = 0.0680
Largest diff. (peak / hole) / e Å^{-3}	1.541/−0.887	0.882/−0.433	4.426/−1.657

[A] $R = \sum||F_O| - |F_C||/\sum|F_O|$; $R_W = [\sum w(F_O^2 - F_C^2)^2/\sum w(F_O^2)^2]^{0.5}$; $S = [\sum w(F_O^2 - F_C^2)^2/(\text{no. data} - \text{no. params})]^{0.5}$ for all data.

Table S5. Crystallographic data for **7**, **8**, **9**, and **10**.

	**7**	**8**	**9**	**10**
Identification code	ccapg491	ccapg439	ccapg250	ccapg263
Formula	$C_{78}H_{144}F_6Si_6U_3$	$C_{32}H_{48}LiO_8U$	$C_{52}H_{96}CeSi_4$	$C_{73}H_{140}CeLiN_4Si_4$
Fw	2078.55	805.67	973.76	1333.30
Temperature / K	100.0(2)	100.00(10)	100.00(10)	99.97(11)
Crystal system	hexagonal	triclinic	monoclinic	triclinic
Space group	$P6_3/m$	$P\overline{1}$	$C2/c$	$P\overline{1}$
a / Å	18.0114(3)	7.9999(3)	22.5858(2)	13.9311(2)
b / Å	18.0114(3)	13.5566(5)	11.60360(10)	14.8365(2)
c / Å	21.4116(3)	15.3361(6)	22.5648(3)	21.5626(4)
α / °	90	73.862(3)	90	89.1378(14)
β / °	90	89.289(3)	113.7915(13)	76.5517(15)
γ / °	120	88.552(3)	90	79.4896(14)
Volume / Å^3	6015.5(2)	1597.16(10)	5411.15(11)	4260.08(13)
Z	2	2	4	2
ρ_{calcg} / cm^3	1.148	1.675	1.195	1.039
μ / mm^{-1}	12.099	14.694	7.563	0.627
F(000)	2052	798	2088	1446
Crystal size / mm^3	0.25 x 0.2 x 0.11	0.19 × 0.09 × 0.07	0.139 × 0.118 × 0.083	0.256 × 0.216 × 0.178
Radiation	Cu Kα (λ = 1.54184)	Cu Kα (λ = 1.54184)	Cu Kα (λ = 1.54184)	Mo Kα (λ = 0.71073)
2Θ range / °	5.67 to 140.11	6.00 to 136.49	8.57 to 140.15	4.35 to 52.04
Index ranges	−19 ≤ h ≤ 21, −21 ≤ k ≤ 21, −26 ≤ l ≤ 23	−9 ≤ h ≤ 9, −16 ≤ k ≤ 16, −18 ≤ l ≤ 18	−27 ≤ h ≤ 24, −14 ≤ k ≤ 13, −25 ≤ l ≤ 27	−17 ≤ h ≤ 17, −18 ≤ k ≤ 18, −26 ≤ l ≤ 25
No. reflections	50174	11057	19261	62640
Unique reflections	3912 [R_{int} = 0.0818, R_{sigma} = 0.0326]	–	5113 [R_{int} = 0.0370, R_{sigma} = 0.0352]	16759 [R_{int} = 0.0308, R_{sigma} = 0.0282]
Data / restraints / parameters	3912/0/148	11057/932/380	5113/0/270	16759/3/783
GOOF on F^2	1.197	1.069	1.057	1.045
Final R indexes [I ≥ 2σ (I)] [A]	R_1 = 0.0539, wR_2 = 0.1351	R_1 = 0.0796, wR_2 = 0.2136	R_1 = 0.0223, wR_2 = 0.0559	R_1 = 0.0250, wR_2 = 0.0620
Final R indexes [all data]	R_1 = 0.0553, wR_2 = 0.1358	R_1 = 0.0920, wR_2 = 0.2291	R_1 = 0.0231, wR_2 = 0.0563	R_1 = 0.0283, wR_2 = 0.0630
Largest diff. (peak / hole) / e Å^{-3}	1.826/−2.018	6.231/−5.518	0.612/−0.332	0.649/−0.624

[A] $R = \sum||F_O| - |F_C||/\sum|F_O|$; $R_W = [\sum w(F_O^2 - F_C^2)^2/\sum w(F_O^2)^2]^{0.5}$; $S = [\sum w(F_O^2 - F_C^2)^2/(\text{no. data} - \text{no. params})]^{0.5}$ for all data.

Table S6. Crystallographic data for [nBu$_4$N][SbF$_6$].

	**7**
Identification code	ccapg591
Formula	$C_{16}H_{36}NF_6Sb$
Fw	478.21
Temperature / K	100.00(10)
Crystal system	monoclinic
Space group	$P2_1/c$
a / Å	9.81929(16)
b / Å	14.1989(2)
c / Å	15.2735(3)
α / °	90
β / °	91.4985(15)
γ / °	90
Volume / Å^3	2128.75(6)
Z	4
ρ_{calcg} / cm^3	1.492
μ / mm^{-1}	10.734
F(000)	976.0
Crystal size / mm^3	0.23 × 0.16 × 0.07
Radiation	Cu Kα (λ = 1.54184)
2Θ range / °	8.504 to 140.15
Index ranges	$-11 \le h \le 11$, $-17 \le k \le 17$, $-18 \le l \le 17$
No. reflections	6912
Unique reflections	6912 [R_{sigma} = 0.0234]
Data / restraints / parameters	6912/0/222
GOOF on F^2	1.053
Final R indexes [I ≥ 2σ (I)] [A]	R_1 = 0.0503, wR_2 = 0.1409
Final R indexes [all data]	R_1 = 0.0529, wR_2 = 0.1438
Largest diff. (peak / hole) / e Å^{-3}	2.99/–1.13

[A] $R = \sum||F_O| - |F_C||/\sum|F_O|$; $R_W = [\sum w(F_O^2 - F_C^2)^2/\sum w(F_O^2)^2]^{0.5}$; $S = [\sum w(F_O^2 - F_C^2)^2/(\text{no. data} - \text{no. params})]^{0.5}$ for all data.

Molecular structures

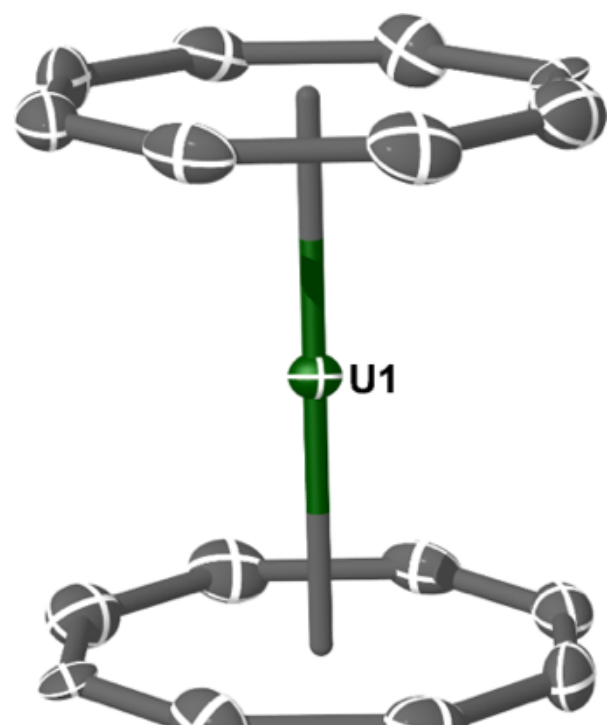

Figure S9. Molecular structure of **1**. Ellipsoids set at 50% probability and H-atoms removed for clarity (operations: X, Y, Z). Selected bond lengths and angles: U1–C_{range} = 2.639(13)–2.651(12) Å, U1···$C_{8\text{-centroid}}$(1) = 1.912(4) Å, $C_{8\text{-centroid}}$(1)···U1···$C_{8\text{-centroid}}$(2) = 180°.

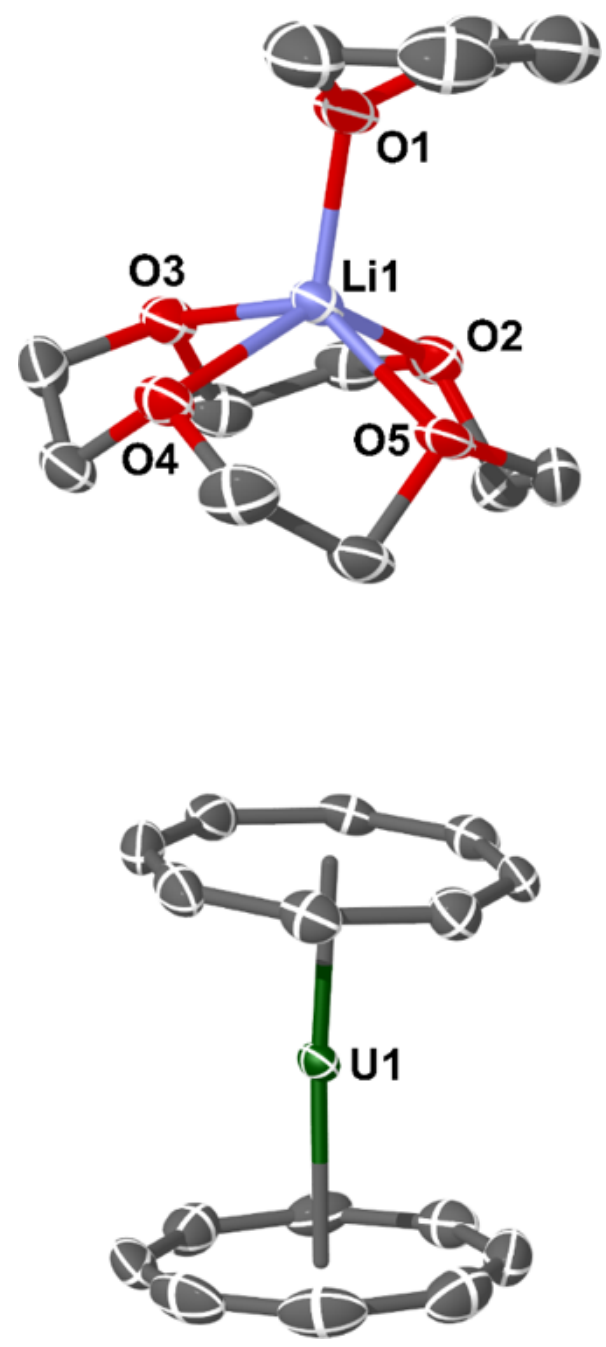

Figure S10. Molecular structure of **2**. Ellipsoids set at 50% probability and H-atoms removed for clarity (operations: X, Y, Z). Selected bond lengths and angles: U1–C_{range} = 2.716(5)–2.756(5) Å, U1···$C_{8\text{-centroid}}$(1) = 2.011(2) Å, U1···$C_{8\text{-centroid}}$(2) = 2.024(2) Å, $C_{8\text{-centroid}}$(1)···U1···$C_{8\text{-centroid}}$(2) = 173.87(6)°.

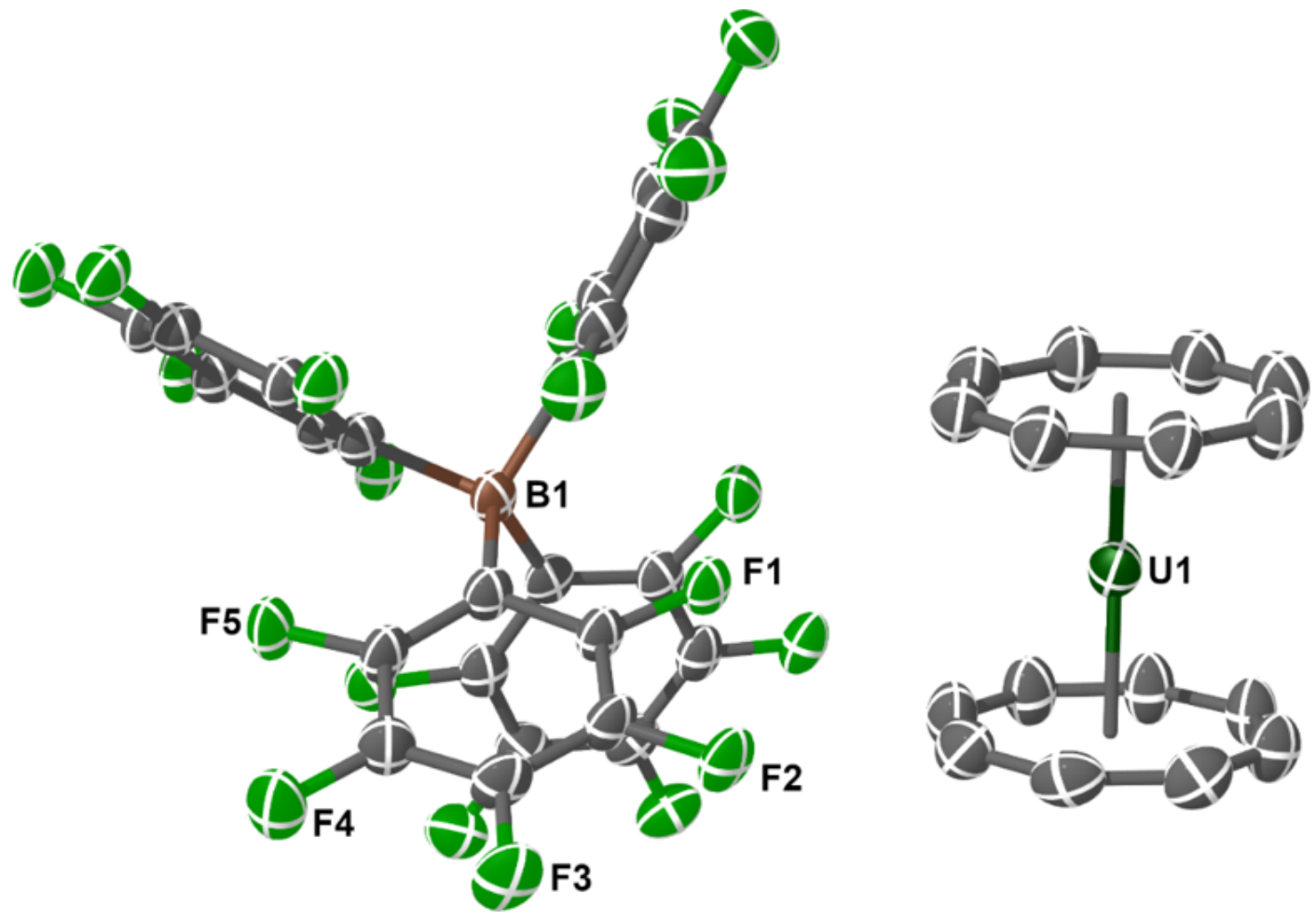

Figure S11. Molecular structure of **3**. Molecular structure of **3**. Ellipsoids set at 50% probability and H-atoms removed for clarity (operations: X, Y, Z). Selected bond lengths and angles: U1–C_{range} = 2.559(14)–2.70(2) Å, U1⋯$C_{8\text{-centroid}}$(1) = 1.850(5) Å, U1⋯$C_{8\text{-centroid}}$(2) = 1.878(3) Å, $C_{8\text{-centroid}}$(1)⋯U1⋯$C_{8\text{-centroid}}$(2) = 175.86(16)°.

U2–C_{range} = 2.560(12)–2.75(3) Å, U2⋯$C_{8\text{-centroid}}$(1) = 1.855(5) Å, U2⋯$C_{8\text{-centroid}}$(2) = 1.869(3) Å, $C_{8\text{-centroid}}$(1)⋯U2⋯$C_{8\text{-centroid}}$(2) = 177.64(17)°.

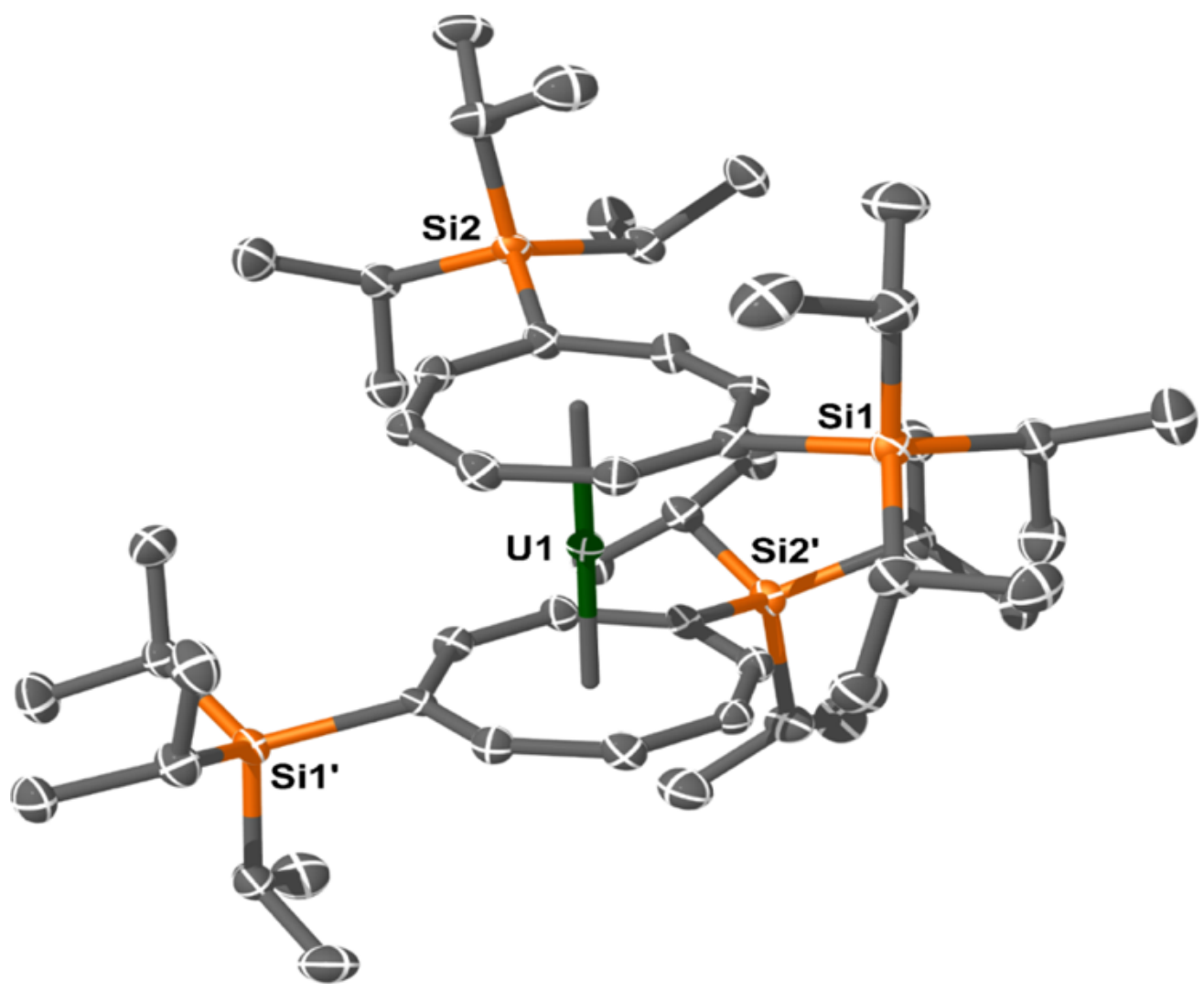

Figure S12. Molecular structure of **4**. Ellipsoids set at 50% probability and H-atoms removed for clarity (operations: X, Y, Z; ½–X, Y, 1–Z). Selected bond lengths and angles: U1–C_{range} = 2.632(3)–2.748(2) Å, U1⋯$C_{8\text{-centroid}}$ = 1.945(1) Å, $C_{8\text{-centroid}}$⋯U1⋯$C_{8\text{-centroid}}$' = 177.34(5)°.

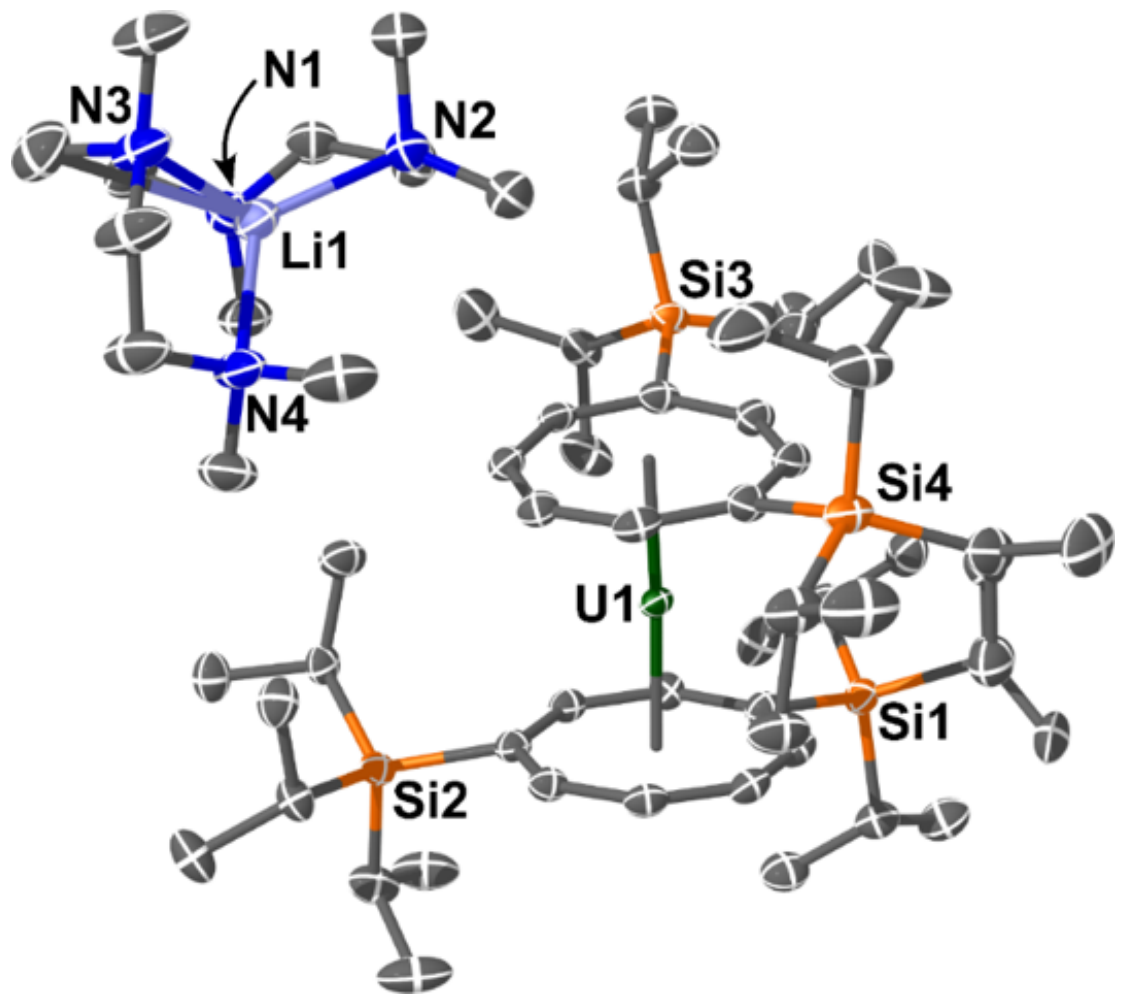

Figure S13. Molecular structure of **5**. Ellipsoids set at 50% probability and H-atoms removed for clarity (operations: X, Y, Z). Selected bond lengths and angles: U1–C_{range} = 2.702(3)–2.824(2) Å, U1···$C_{8\text{-centroid}}$(1) = 2.034(1) Å, U1···$C_{8\text{-centroid}}$(2) = 2.042(1) Å, $C_{8\text{-centroid}}$(1)···U1···$C_{8\text{-centroid}}$(2) = 173.39(4)°.

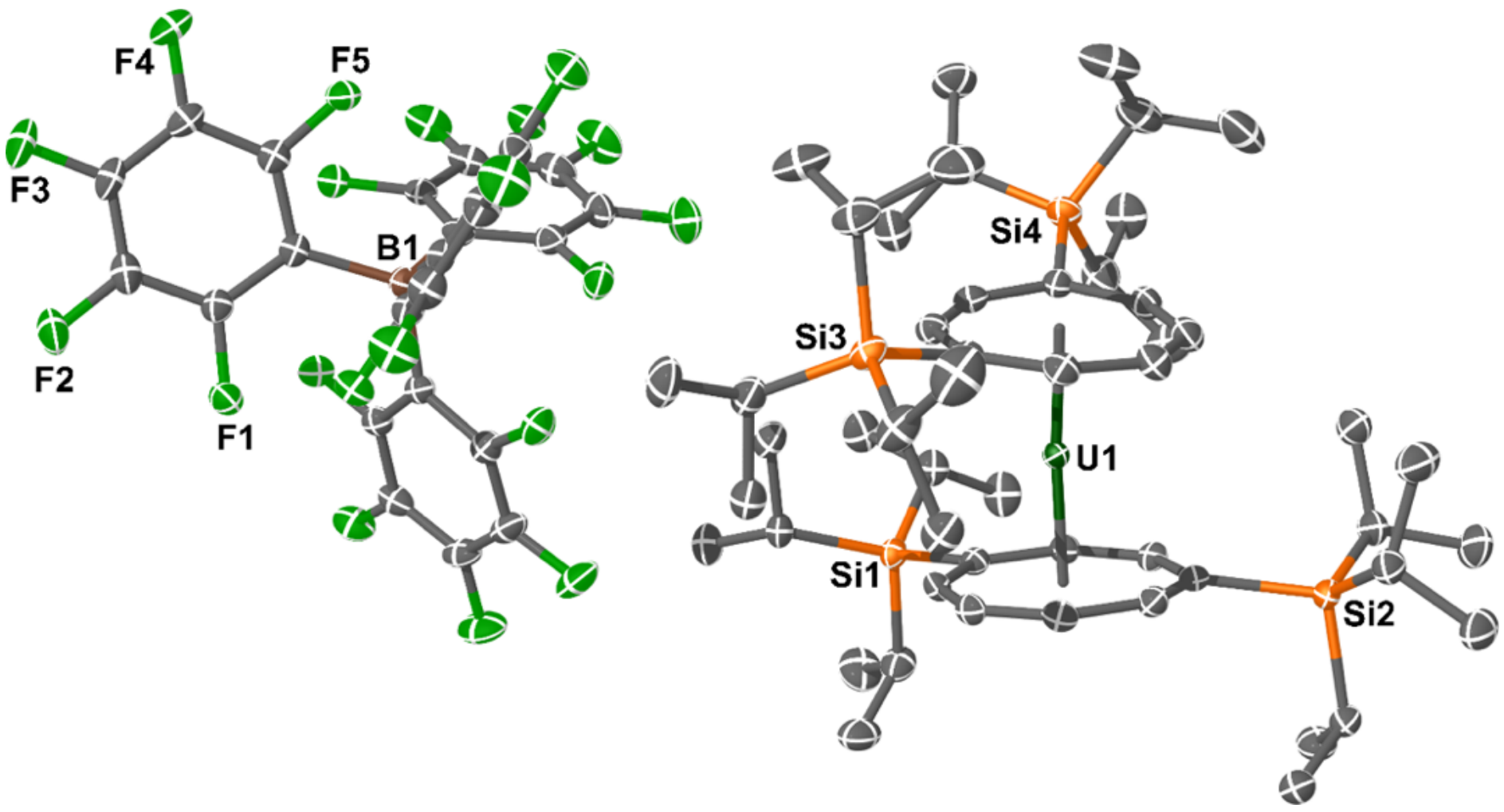

Figure S14. Molecular structure of **6**. Ellipsoids set at 50% probability. Disordered components and H-atoms removed for clarity (operations: X, Y, Z). Selected bond lengths and angles: U1–C_{range} = 2.619(3)–2.769(3) Å, U1···$C_{8\text{-centroid}}$(1) = 1.907(1) Å, U1···$C_{8\text{-centroid}}$(2) = 1.924(1) Å, $C_{8\text{-centroid}}$(1)···U1···$C_{8\text{-centroid}}$(2) = 171.99(5)°. U2–C_{range} = 2.619(3)–2.735(3) Å, U2···$C_{8\text{-centroid}}$(1) = 1.912(1) Å, U2···$C_{8\text{-centroid}}$(2) = 1.913(1) Å, $C_{8\text{-centroid}}$(1)···U2···$C_{8\text{-centroid}}$(2) = 171.33(6)°.

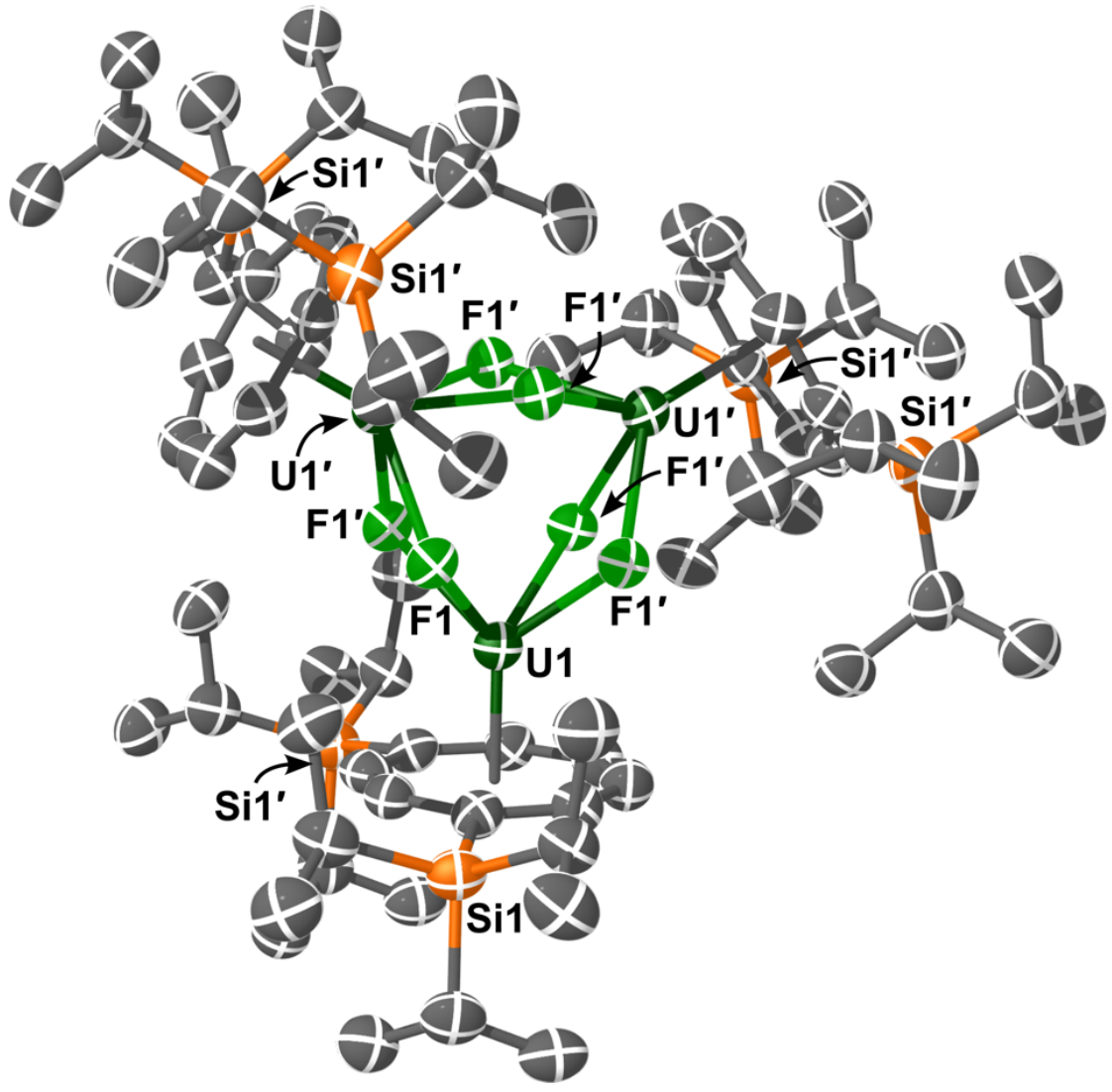

Figure S15. Molecular structure of **7**. Ellipsoids set at 50% probability, and H-atoms removed for clarity (operations: X, Y, Z; X, Y, 3/2–Z; 1–Y, 1+X, 3/2–Z; 1–Y, 1+X–Y, Z; Y–X, 1–X; Z; Y–X, 1–X, 3/2–Z). Selected bond lengths and angles: U1–C_{range} = 2.601(8)–2.675(7) Å, U1···$C_{8\text{-centroid}}$ = 1.890(2) Å, U–F = 2.316(4) Å, U1–F1–U1′ = 111.23(17)°.

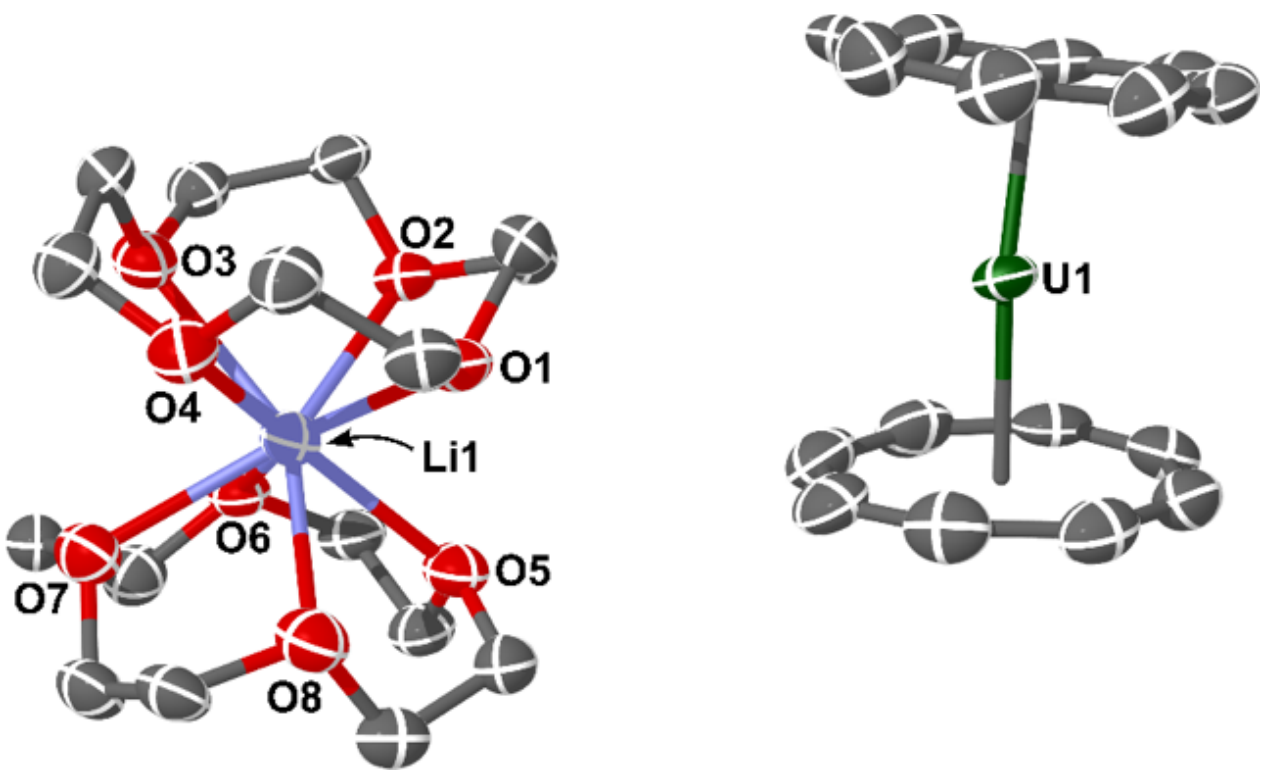

Figure S16. Molecular structure of **8**. Ellipsoids set at 50% probability and H-atoms removed for clarity (operations: X, Y, Z). Selected bond lengths and angles: U1–C_{range} = 2.639(17)–2.761(19) Å, U1···$C_{8\text{-centroid}}$(1) = 1.999(7) Å, U1···$C_{8\text{-centroid}}$(2) = 2.000(8) Å, $C_{8\text{-centroid}}$(1)···U1···$C_{8\text{-centroid}}$(2) = 171.62(21)°.

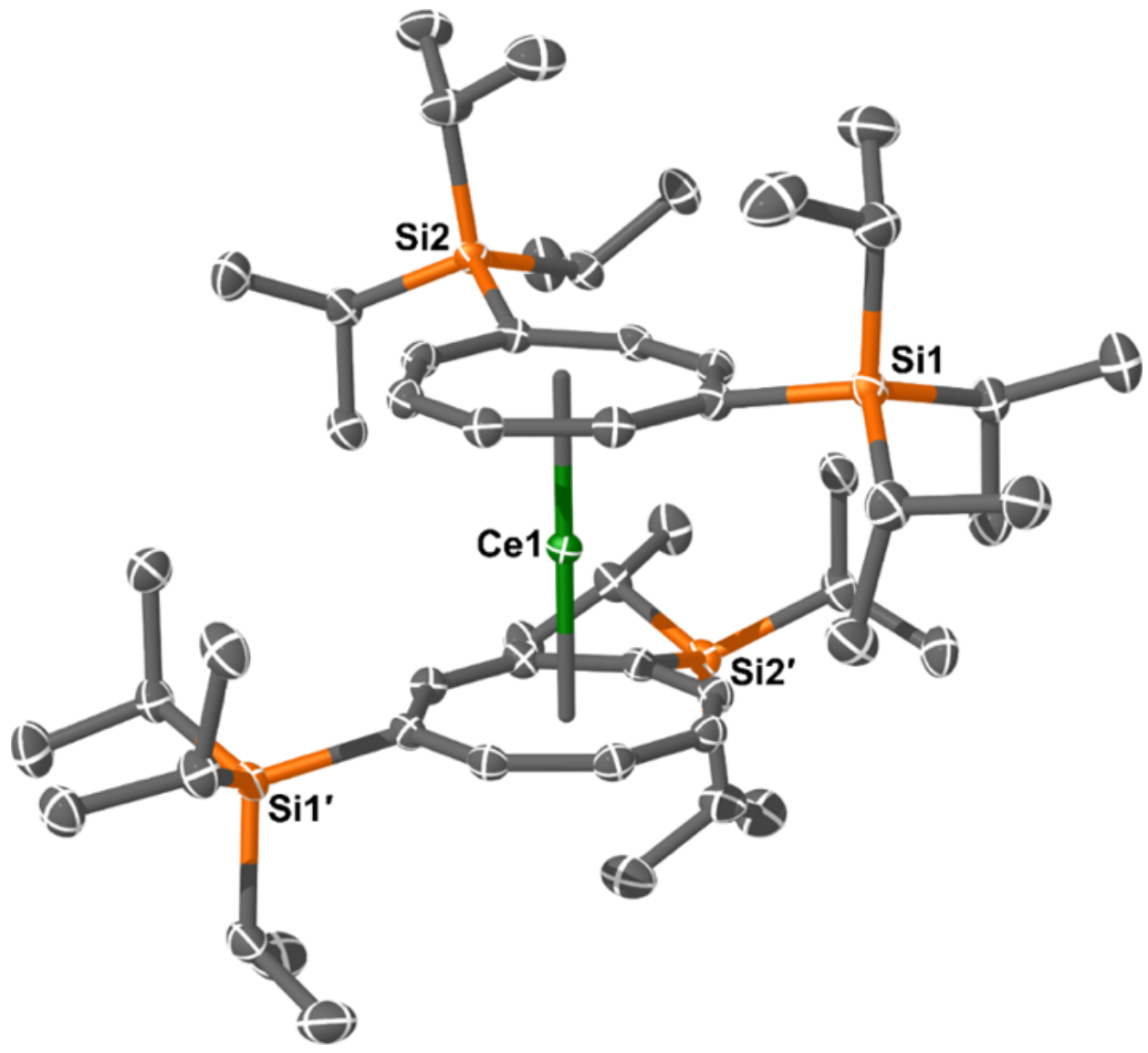

Figure S17. Molecular structure of **9**. Ellipsoids set at 50% probability, and H-atoms removed for clarity (operations: X, Y, Z; ½–X, Y, 1–Z). Selected bond lengths and angles: Ce1–C_{range} = 2.6670(18)–2.7758(17) Å, Ce1···$C_{8\text{-centroid}}$ = 1.992(1) Å, $C_{8\text{-centroid}}$···Ce1···$C_{8\text{-centroid}}$' = 177.54(4)°.

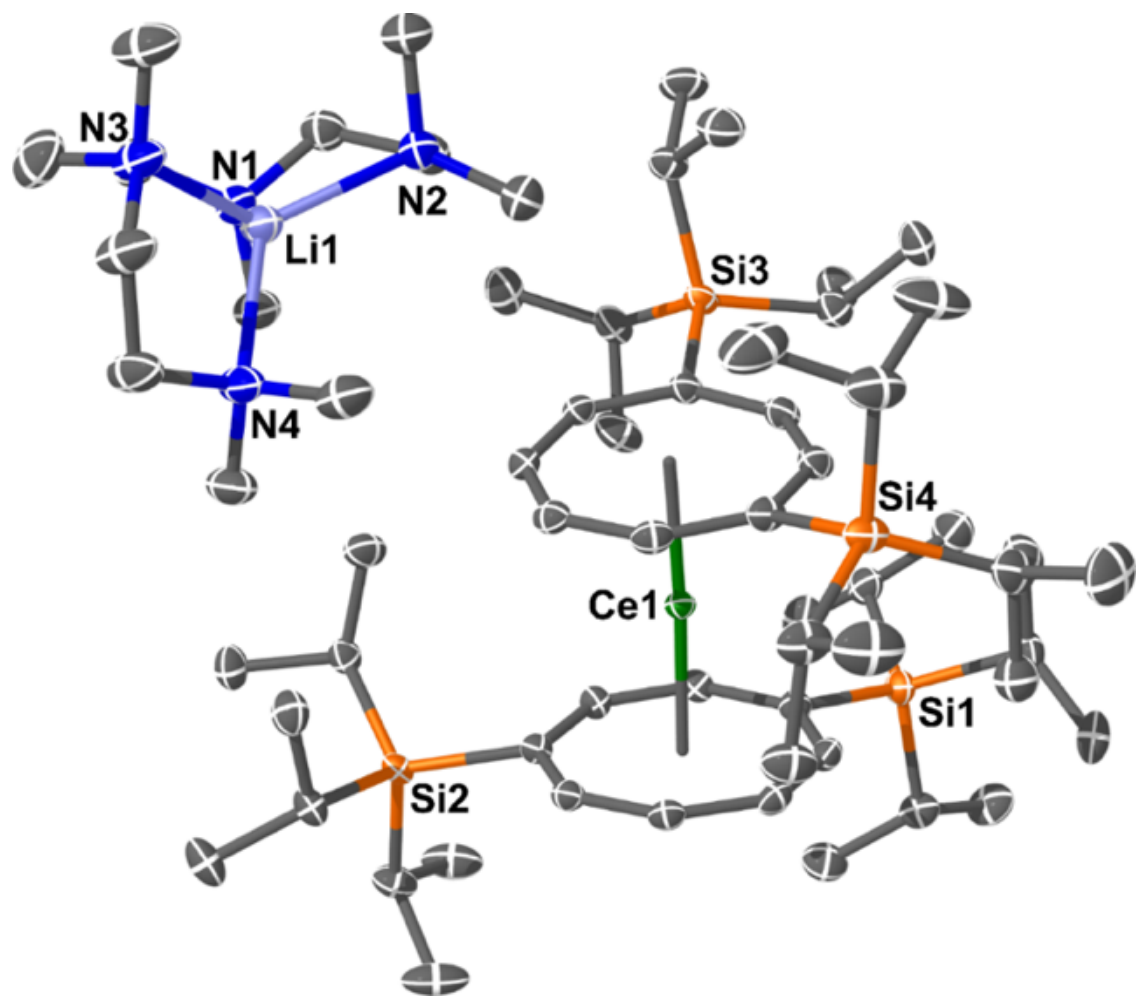

Figure S18. Molecular structure of **10**. Ellipsoids set at 50% probability and H-atoms removed for clarity (operations: X, Y, Z). Selected bond lengths and angles: Ce1–C_{range} = 2.7321(16)–2.8386(15) Å, Ce1···$C_{8\text{-centroid}}$(1) = 2.062(1) Å, Ce1···$C_{8\text{-centroid}}$(2) = 2.071(1) Å, $C_{8\text{-centroid}}$(1)···Ce1···$C_{8\text{-centroid}}$(2) = 173.43(2)°.

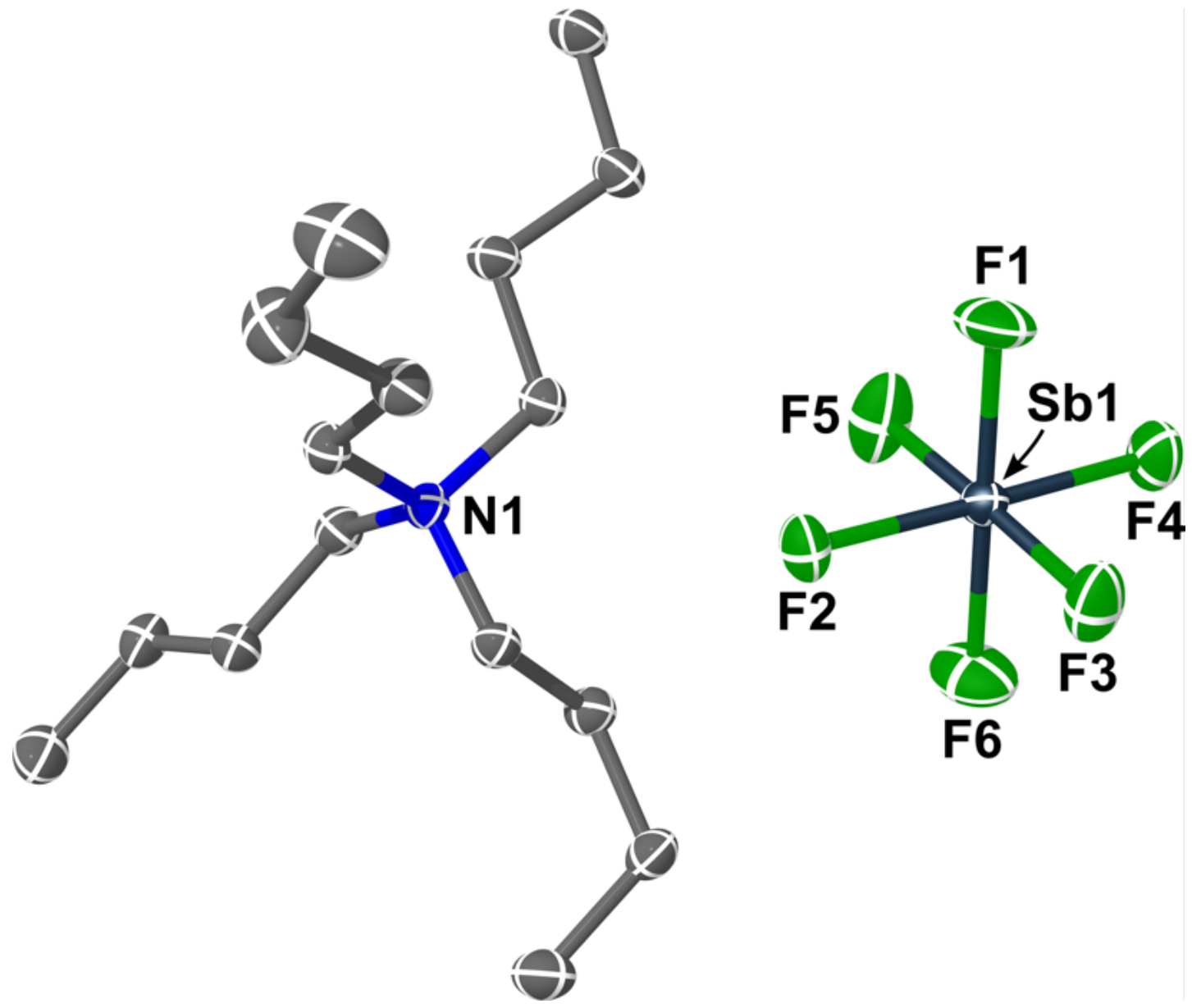

Figure S19. Molecular structure of [nBu$_4$N][SbF$_6$]. Ellipsoids set at 50% probability and H-atoms removed for clarity (operations: X, Y, Z). Selected bond lengths: Sb1–F1 = 1.865(3) Å, Sb1–F2 = 1.877(3) Å, Sb1–F3 = 1.868(3) Å, Sb1–F4 = 1.875(3) Å, Sb1–F5 = 1.873(4) Å, Sb1–F6 = 1.879(3) Å.

Table S7. Selected bond lengths (Å) and angles (°) for complexes **1**, **2**, **3** and **8**.

(Å or °)	1	2	8	3 (U1)	3 (U2)
U1–C_{range}	2.639(13)–2.651(12)	2.716(5)–2.756(5)	2.639(17)–2.761(19)	2.559(14)–2.70(2)	2.560(15)–2.652(10)
U1⋯$C_{8\text{-centroid}}$(1)	1.912(4)	2.011(2)	1.999(7)	1.850(5)	1.855(3)
U1⋯$C_{8\text{-centroid}}$(2)	–	2.024(2)	2.000(8)	1.878(3)	1.869(3)
$C_{8\text{-centroid}}$(1)⋯U1⋯$C_{8\text{-centroid}}$(2)	180	173.87(6)	171.62(21)	175.86(16)	177.64(17)

Table S8. Selected bond lengths (Å) and angles (°) for complexes **4**, **5** and **6**.

(Å or °)	4	5	6 (U1)	6 (U2)
U1–C_{range}	2.632(3)–2.748(2)	2.702(3)–2.824(2)	2.619(3)–2.769(3)	2.619(3)–2.735(3)
U1⋯$C_{8\text{-centroid}}$(1)	1.945(1)	2.034(1)	1.907(1)	1.912(11)
U1⋯$C_{8\text{-centroid}}$(2)	–	2.042(1)	1.924(1)	1.913(11)
$C_{8\text{-centroid}}$(1)⋯U1⋯$C_{8\text{-centroid}}$(2)	177.34(5)	173.39(4)	171.99(5)	171.33(6)

Table S9. Selected bond lengths (Å) and angles (°) for complexes **9** and **10**.

(Å or °)	9	10
M1–C_{range}	2.6670(18)–2.7758(17)	2.7321(16)–2.8386(15)
M1⋯$C_{8\text{-centroid}}$(1)	1.992(1)	2.062(1)
M1⋯$C_{8\text{-centroid}}$(2)	–	2.071(1)
$C_{8\text{-centroid}}$(1)⋯M1⋯$C_{8\text{-centroid}}$(2)	177.54(4)	173.43(2)

General considerations – powder X-ray diffraction (PXRD)

Powder X-ray diffraction (PXRD) was used to determine the bulk purity of **2**–**6**, **9**, and **10**.

Finely ground samples were mounted on the tip of a micromount as a thick paste in Fomblin YR-1800. Datasets were collected using a Rigaku FR-X DW diffractometer, equipped with an FR-X high-intensity rotating anode Cu Kα radiation source (λ = 1.54184 Å) and VariMAXTM microfocus optics, using an AFC-11 4-circle κ goniometer, a HyPix-6000HE hybrid pixel array detector operating in shutterless mode and an Oxford Cryosystems Cryostream 800 nitrogen flow gas system at 100 K. Data were collected between 3–50°θ, with a detector distance of 150 mm and a beam divergence of 0.5 mRad[15]. X-ray data were collected using CrysAlisPro software[7].

The instrument was calibrated using the collected data, with the instrument model refined using diffraction peak positions measured at multiple detector angles. X-ray data were reduced and integrated using CrysAlisPro software[7]. Peak hunting and unit cell indexing were performed using TOPAS software[16]. JANA2020[17] was used for Le Bail profile analysis.

Table S10. Comparison of the unit cells determined from PXRD and SC-XRD data for **2**–**6**, **9**, and **10**.

	PXRD for **2**	SC-XRD for **2**	PXRD for **3**	SC-XRD for **3**
a / Å	8.6976(3)	8.70910(16)	13.6496(20)	13.6552(2)
b / Å	11.6070(3)	11.5974(2)	16.9042(16)	16.8748(3)
c / Å	13.3112(5)	13.3483(2)	20.2096(13)	20.3859(3)
α / °	86.404(3)	86.6715(16)	76.472(8)	77.2700(10)
β / °	88.112(3)	88.1080(15)	88.252(7)	89.1180(10)
γ / °	88.124(3)	88.1424(16)	85.301(11)	84.3150(10)
Volume / Å^3	1339.81(7)	1344.58(4)	4518.1(8)	4559.39(13)
Temperature / K	100	100	100	100

	PXRD for **4**	SC-XRD for **4**	PXRD for **9**	SC-XRD for **9**
a / Å	22.7207(9)	22.4483(2)	22.610(2)	22.5858(2)
b / Å	11.6029(3)	11.59460(10)	11.5947(3)	11.60360(10)
c / Å	22.4514(7)	22.7177(2)	22.5469(17)	22.5648(3)
α / °	90	90	90	90
β / °	114.149(3)	114.1000(10)	113.841(3)	113.7915(13)
γ / °	90	90	90	90
Volume / Å^3	5400.8(3)	5397.53(9)	5406.4(7)	5411.15(11)
Temperature / K	100	100	100	100

	PXRD for **5**	SC-XRD for **5**	PXRD for **10**	SC-XRD for **10**
a / Å	14.120(4)	13.9014(2)	13.9476(13)	13.9311(2)
b / Å	14.565(4)	14.8767(3)	14.8820(13)	14.8365(2)
c / Å	21.447(6)	21.5551(4)	21.506(2)	21.5626(4)
α / °	91.93(2)	88.8714(15)	89.164(9)	89.1378(14)
β / °	75.70(3)	76.2858(15)	76.241(12)	76.5517(15)
γ / °	79.74(2)	79.5075(16)	79.315(8)	79.4896(14)
Volume / Å^3	4192(2)	4256.96(14)	4258.7(8)	4260.08(13)
Temperature / K	100	100	100	100

	PXRD for **6**	SC-XRD for **6**
a / Å	29.8927(7)	29.96026(18)
b / Å	18.9392(3)	18.94501(10)
c / Å	28.5459(8)	28.56509(18)
α / °	90	90
β / °	98.396(2)	98.4083(6)
γ / °	90	90
Volume / Å^3	15987.9(7)	16039.20(17)
Temperature/ K	100	100

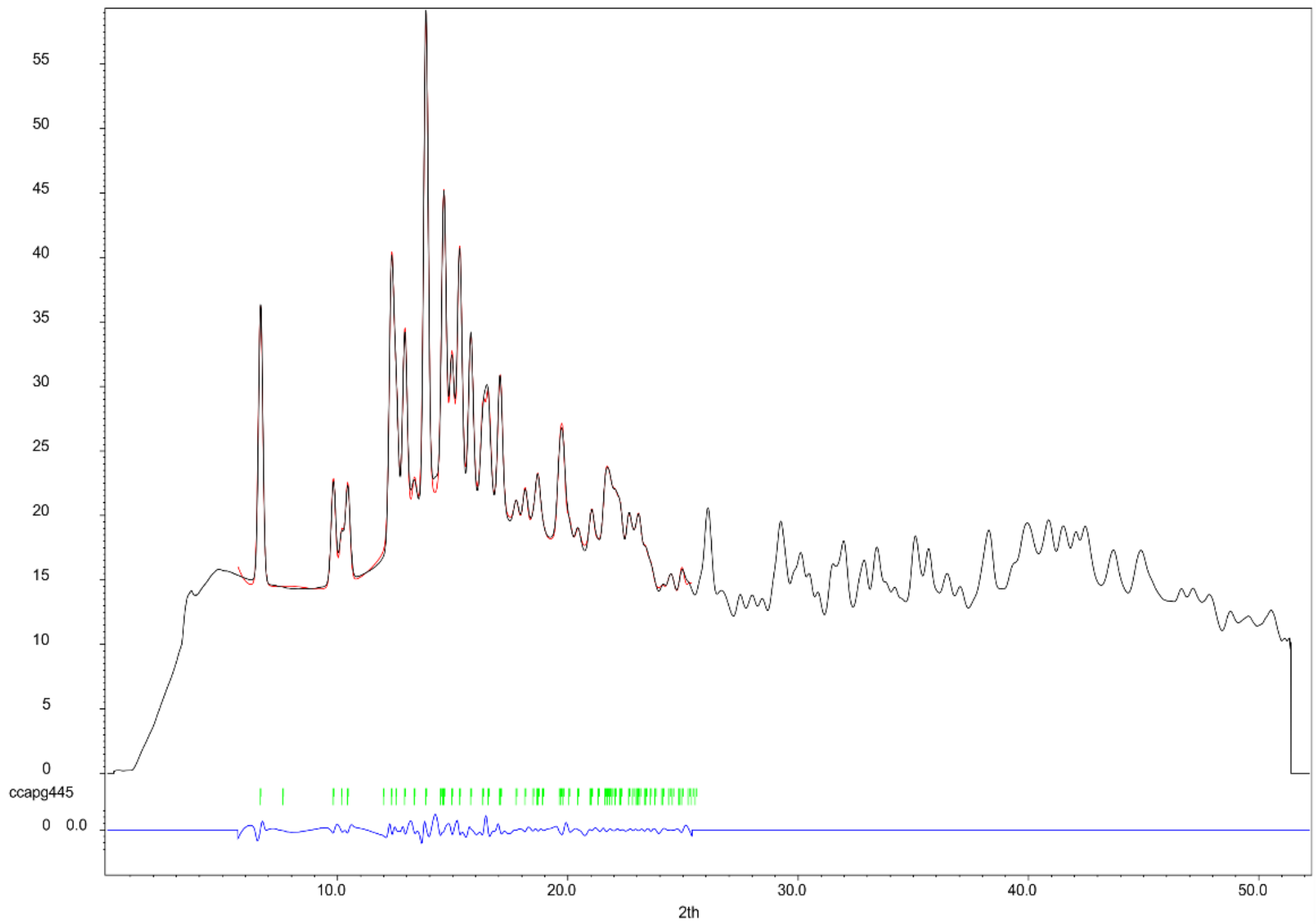

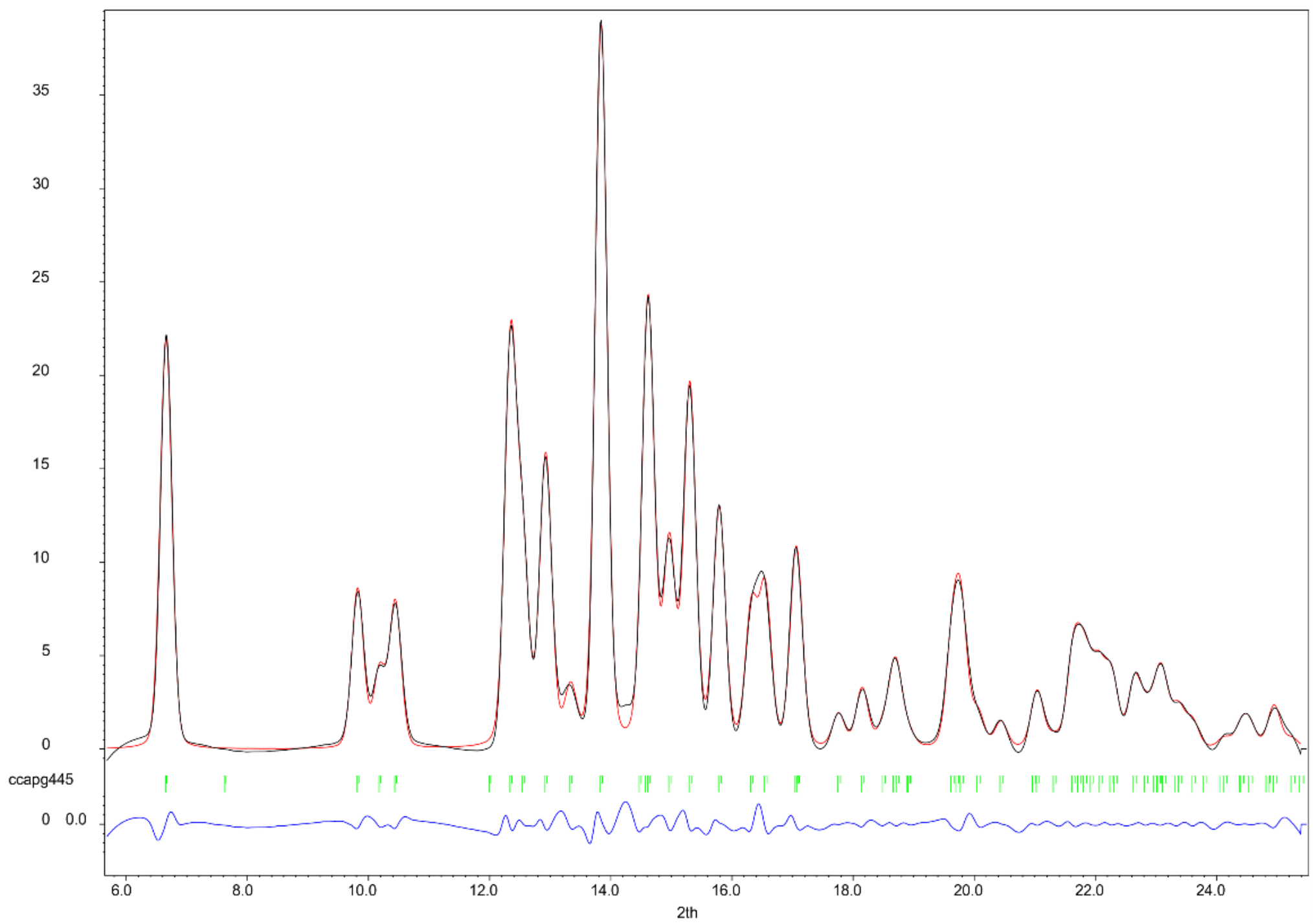

Figure S20. (**Top**) Full PXRD pattern for a sample of **2**; (**Bottom**) Selected range for Le Bail profile fitting (black = observed data, red = calculated profile, green = reflection positions, blue = obs-cal difference, R_p = 0.90%, wR_p = 1.24%).

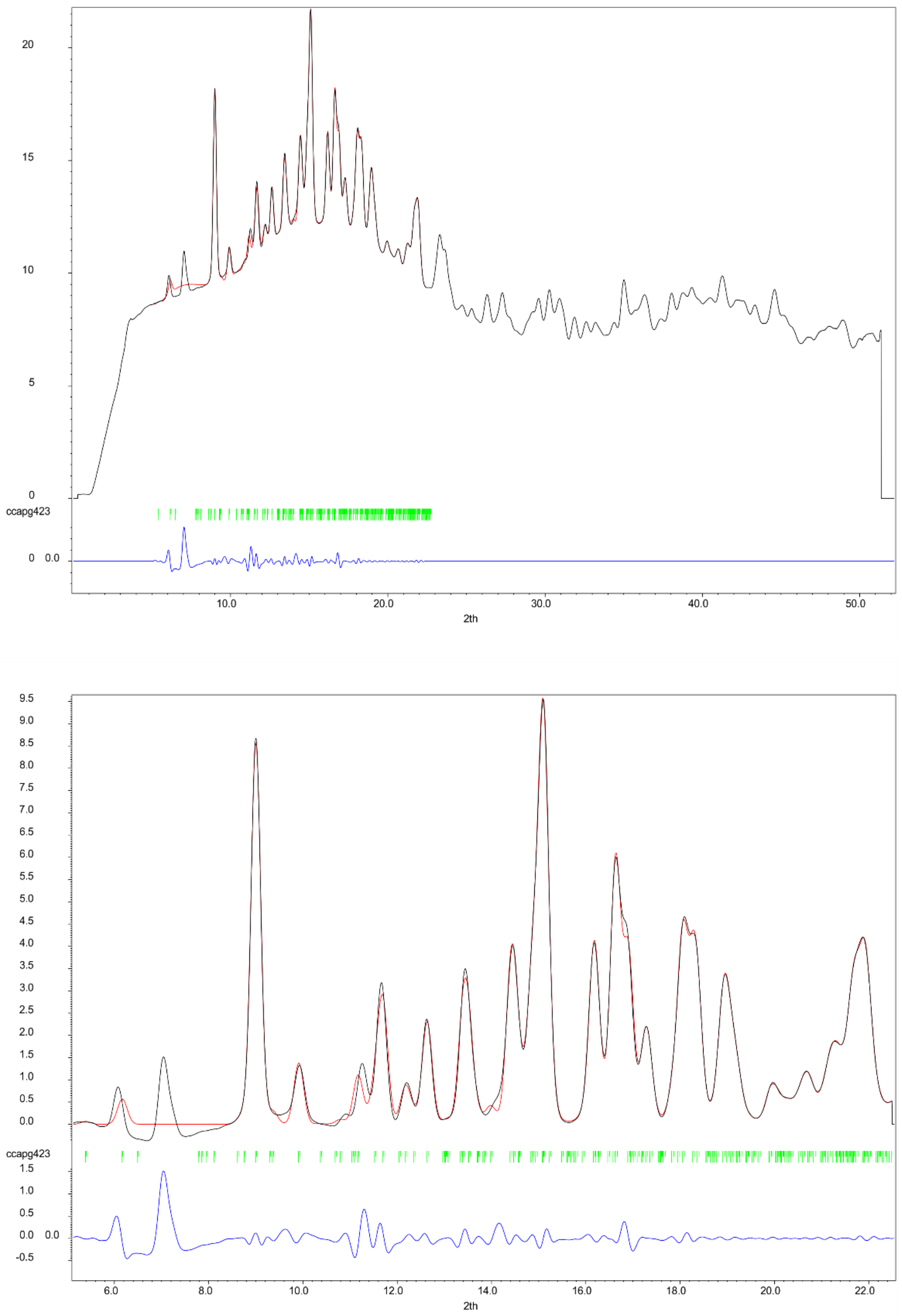

Figure S21. (**Top**) Full PXRD pattern for a sample of **3**; (**Bottom**) Selected range for Le Bail profile fitting (black = observed data, red = calculated profile, green = reflection positions, blue = obs-cal difference, R_p = 0.87%, wR_p = 1.56%).

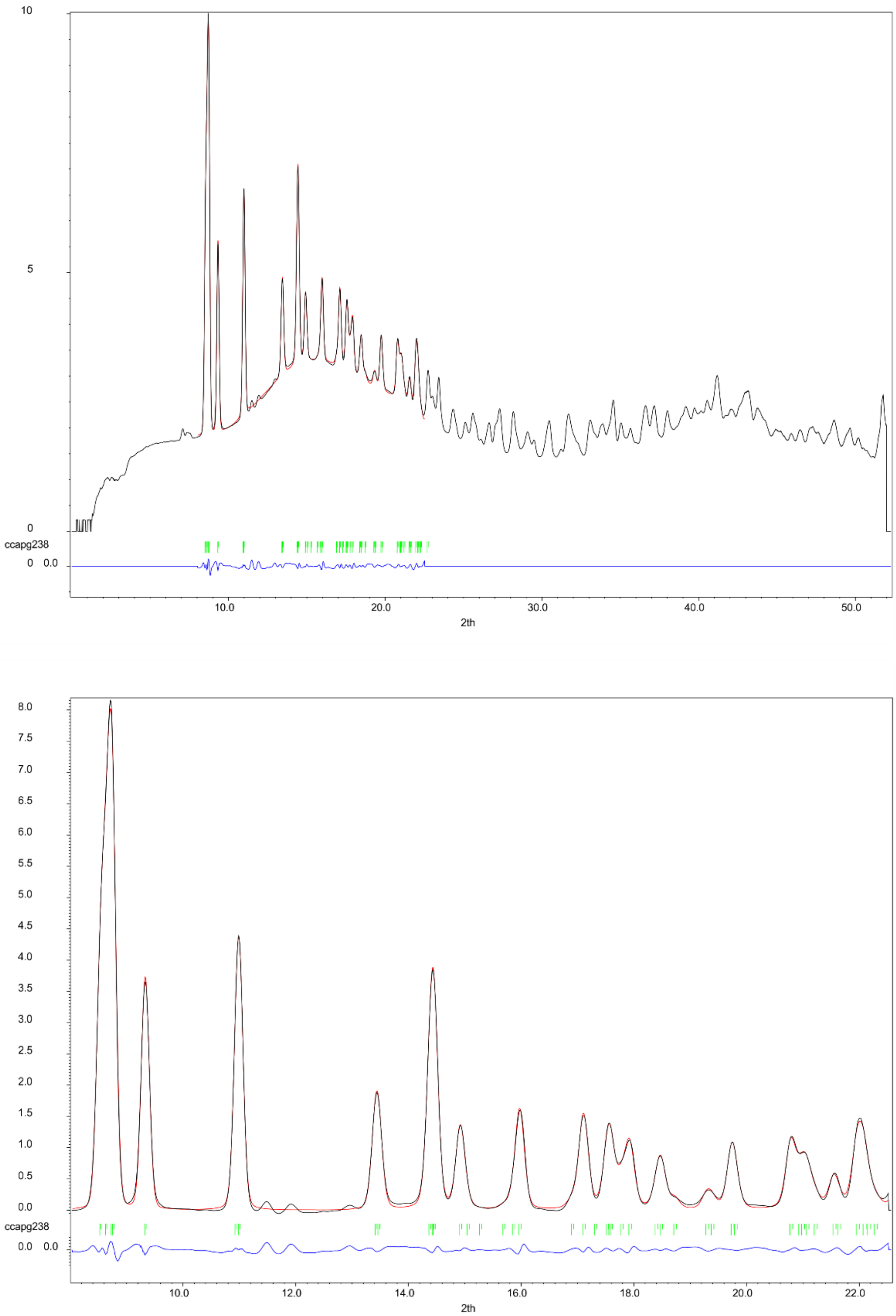

Figure S22. (**Top**) Full PXRD pattern for a sample of **4**; (**Bottom**) Selected range for Le Bail profile fitting (black = observed data, red = calculated profile, green = reflection positions, blue = obs-cal difference, R_p = 0.86%, wR_p = 1.12%).

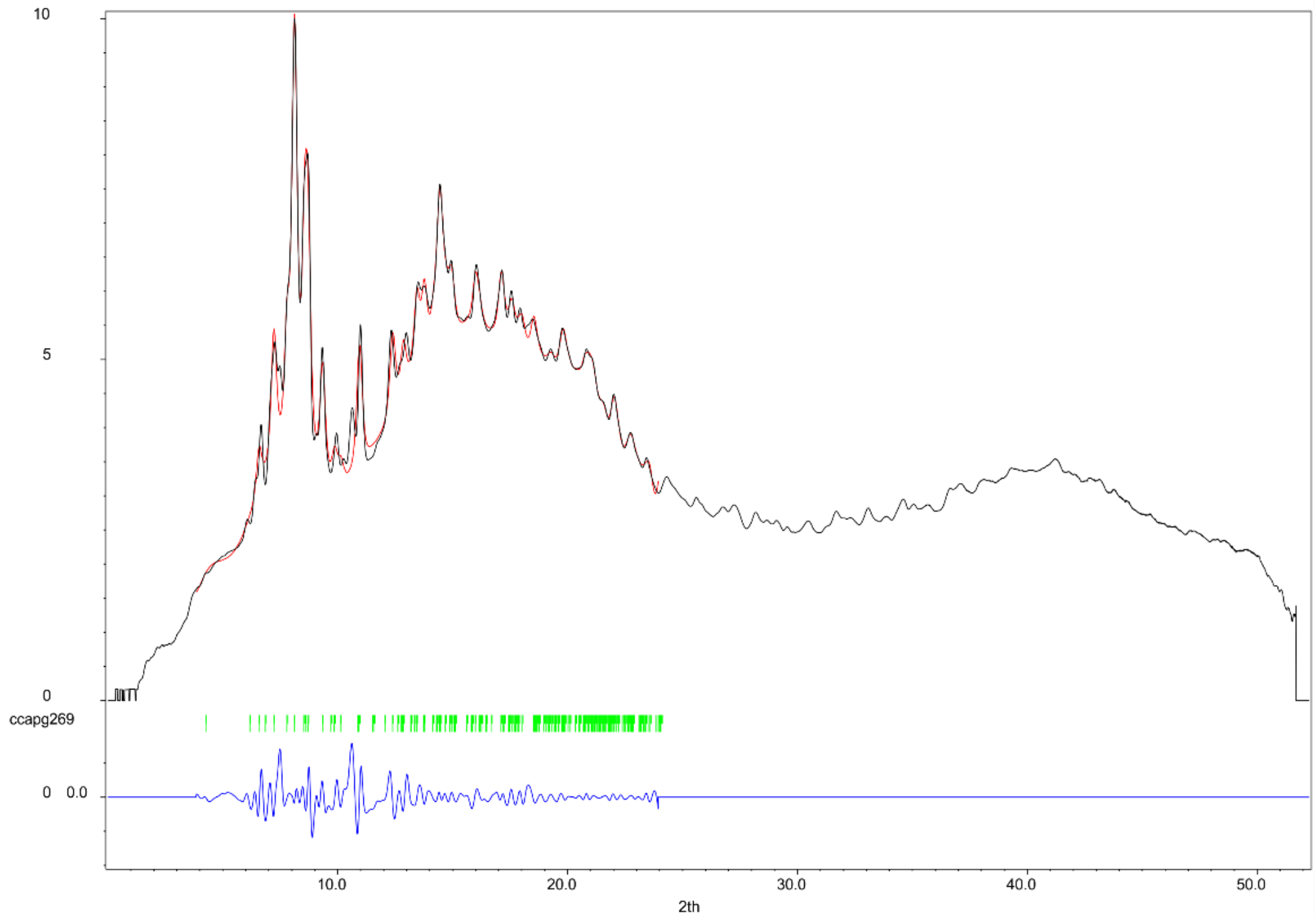

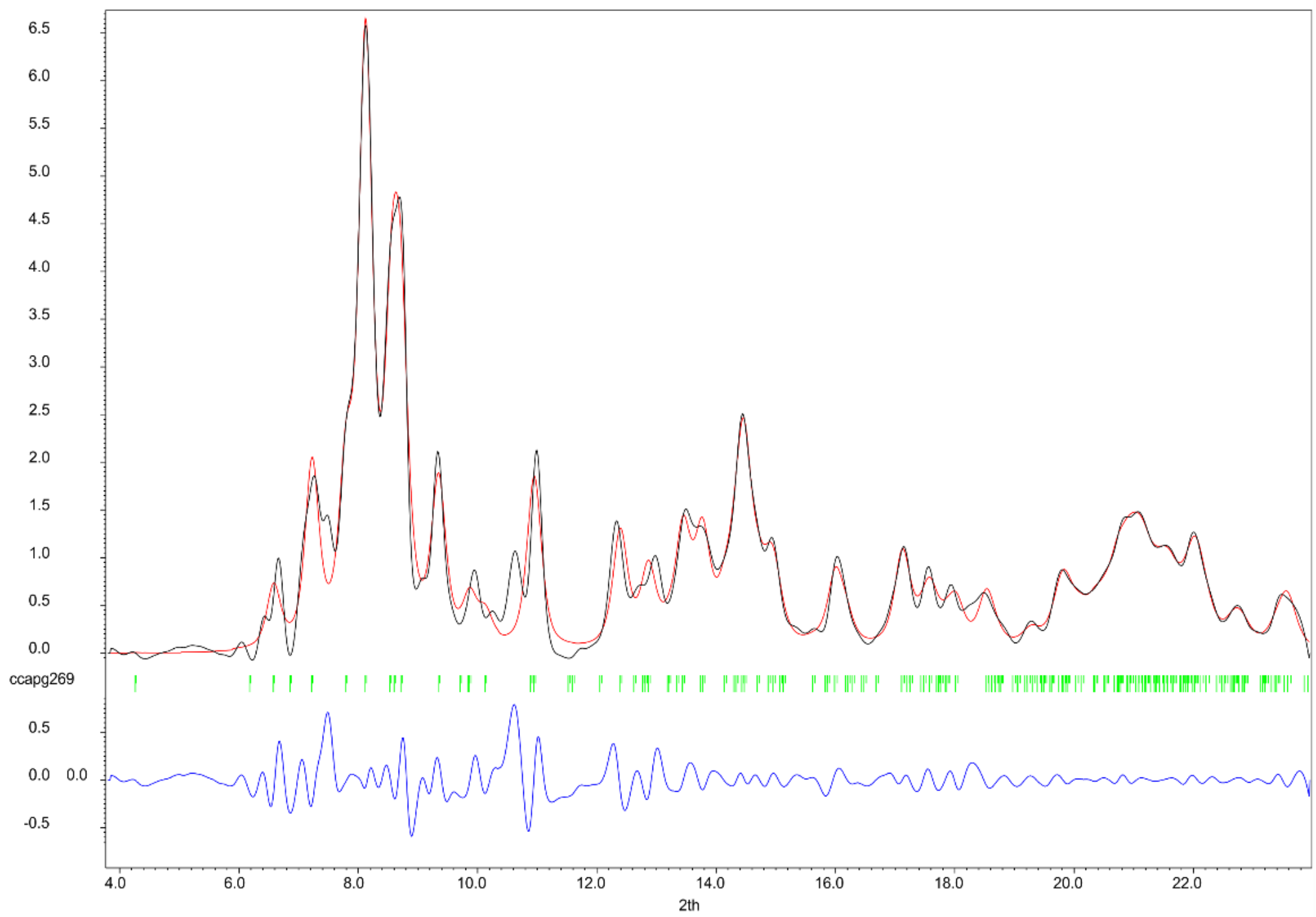

Figure S23. (**Top**) Full PXRD pattern for a sample of **5**; (**Bottom**) Selected range for Le Bail profile fitting (black = observed data, red = calculated profile, green = reflection positions, blue = obs-cal difference, R_p = 1.97%, wR_p = 3.18%).

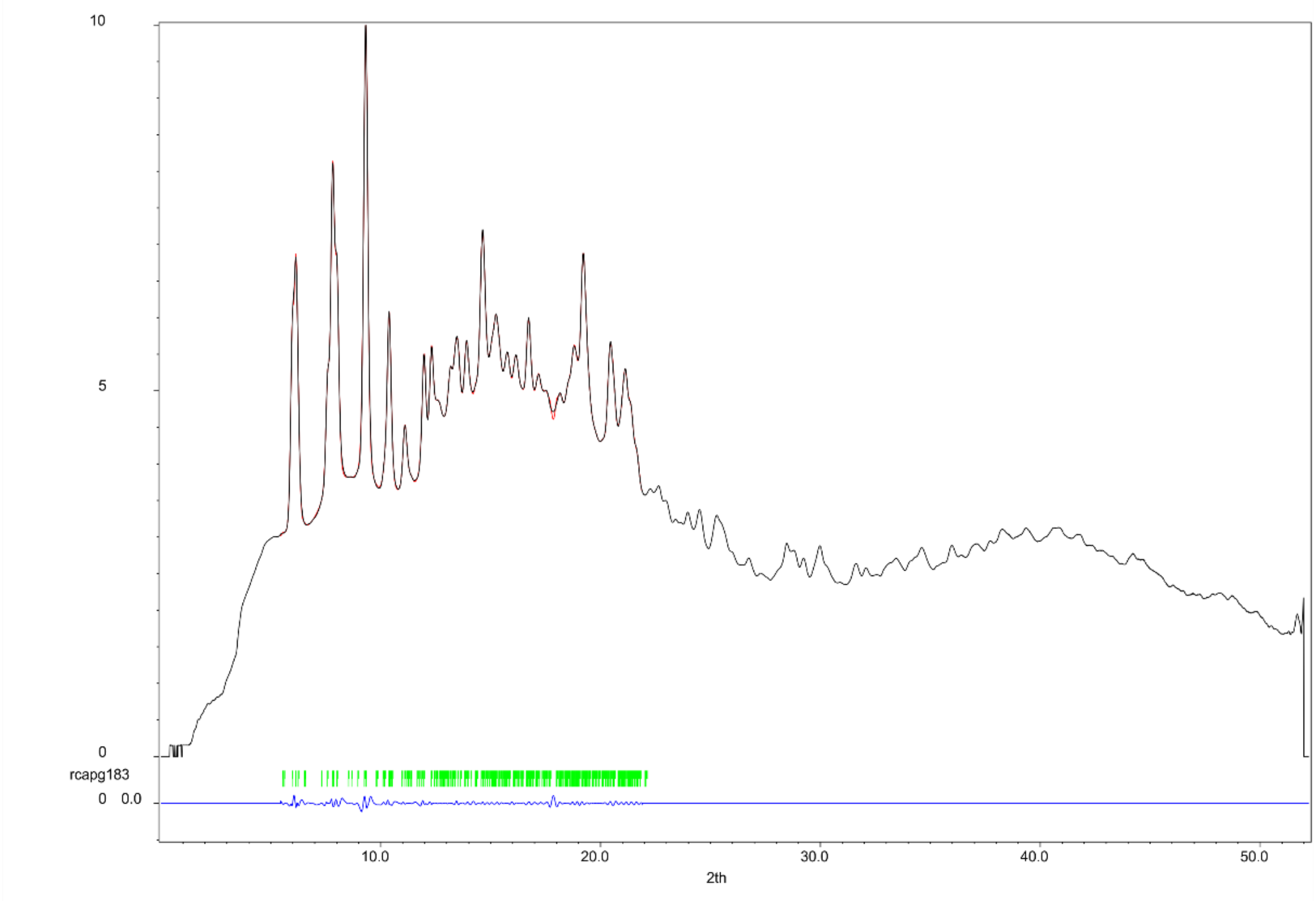

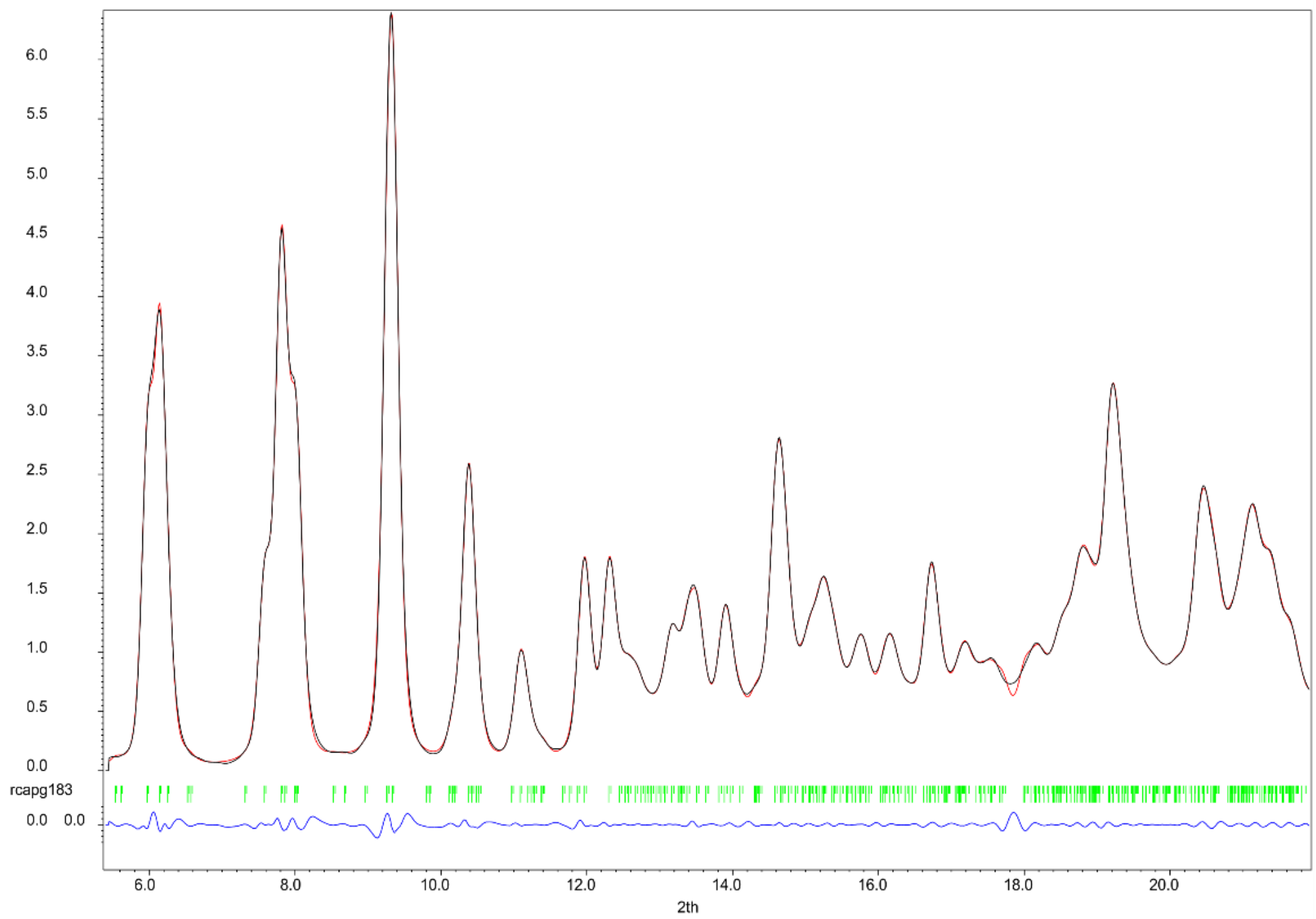

Figure S24. (**Top**) Full PXRD pattern for a sample of **6**; (**Bottom**) Selected range for Le Bail profile fitting (black = observed data, red = calculated profile, green = reflection positions, blue = obs-cal difference, R_p = 0.29%, wR_p = 0.43%).

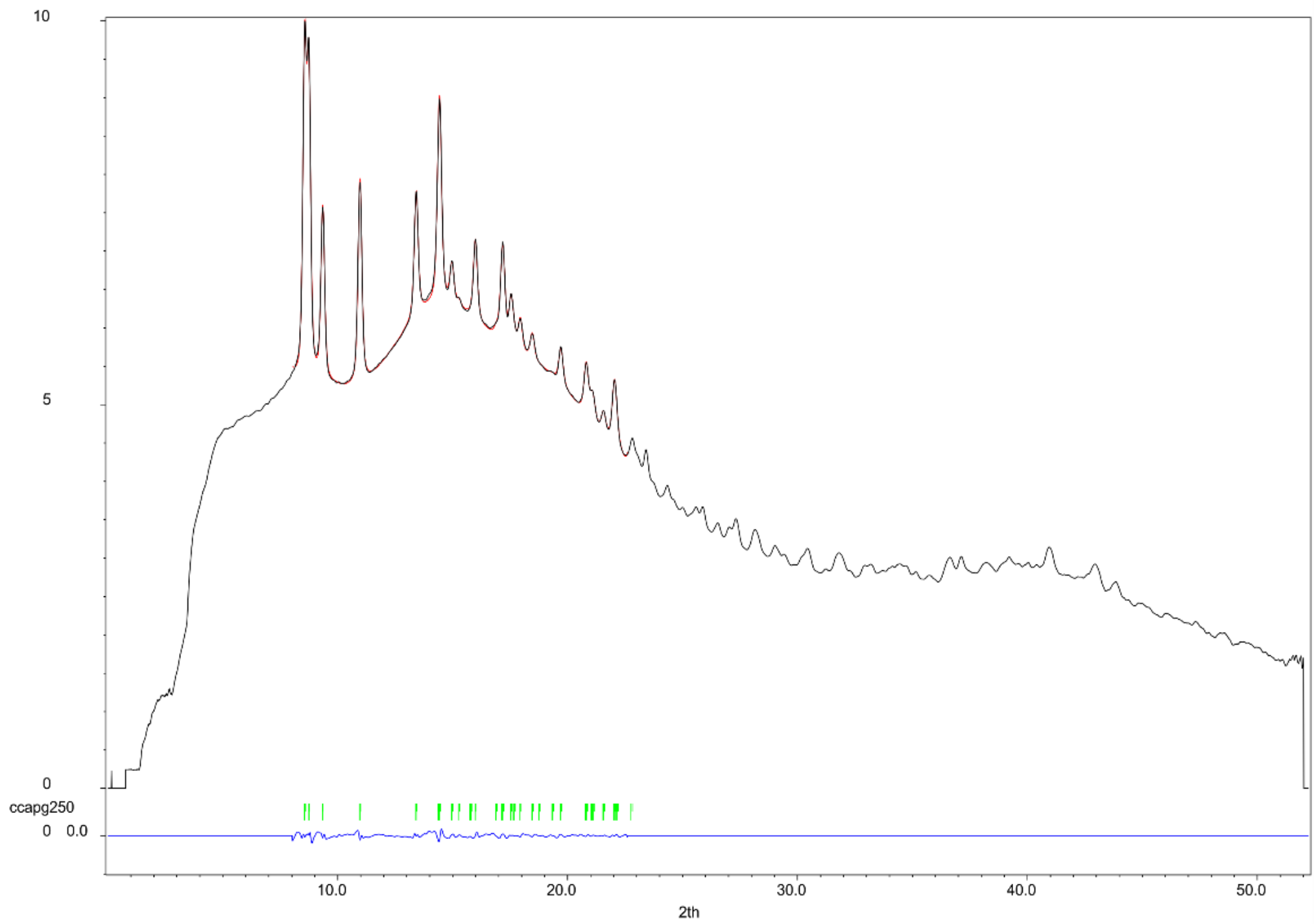

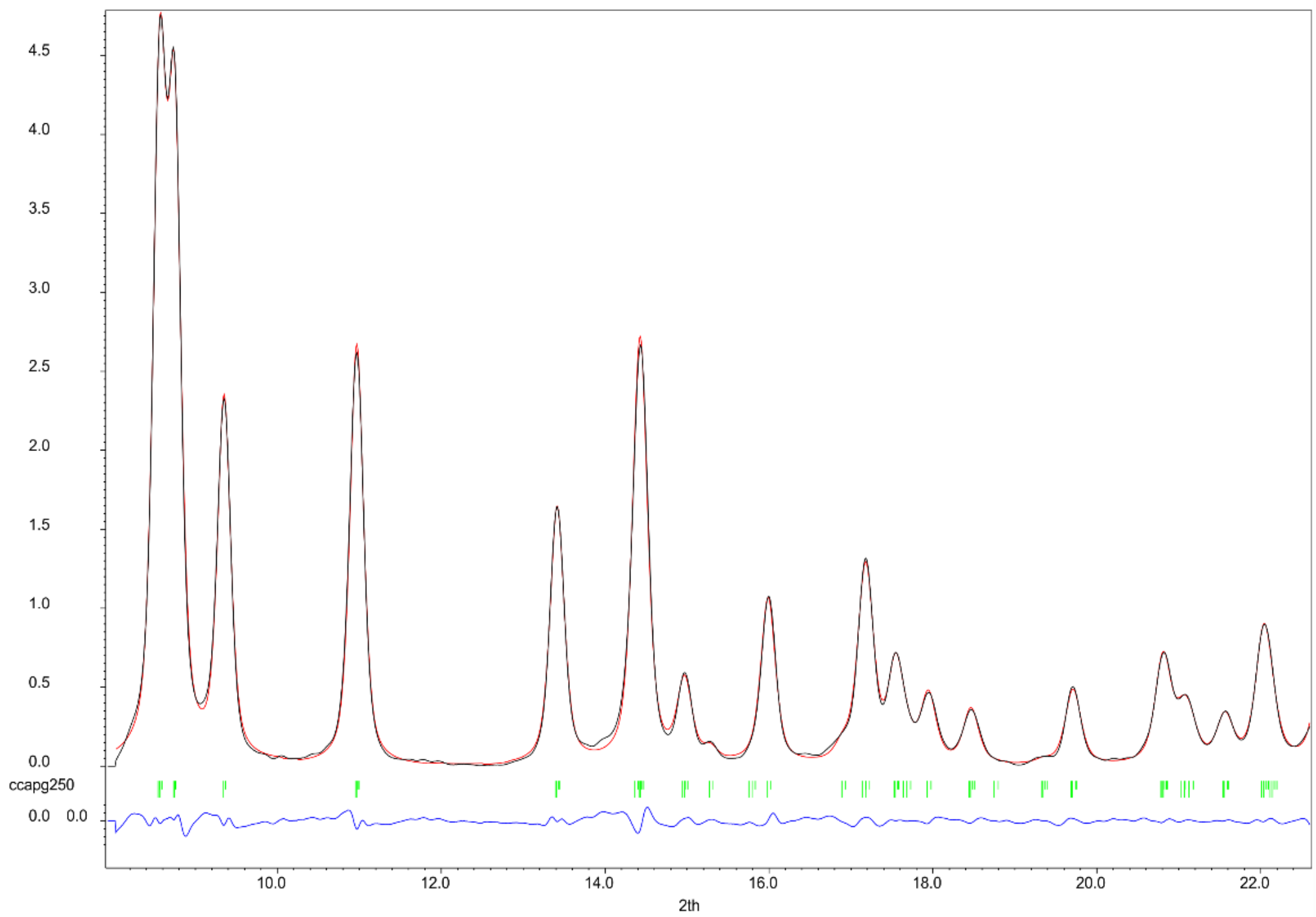

Figure S25. (**Top**) Full PXRD pattern for a sample of **9**; (**Bottom**) Selected range for Le Bail profile fitting (black = observed data, red = calculated profile, green = reflection positions, blue = obs-cal difference, R_p = 0.27%, wR_p = 0.36%).

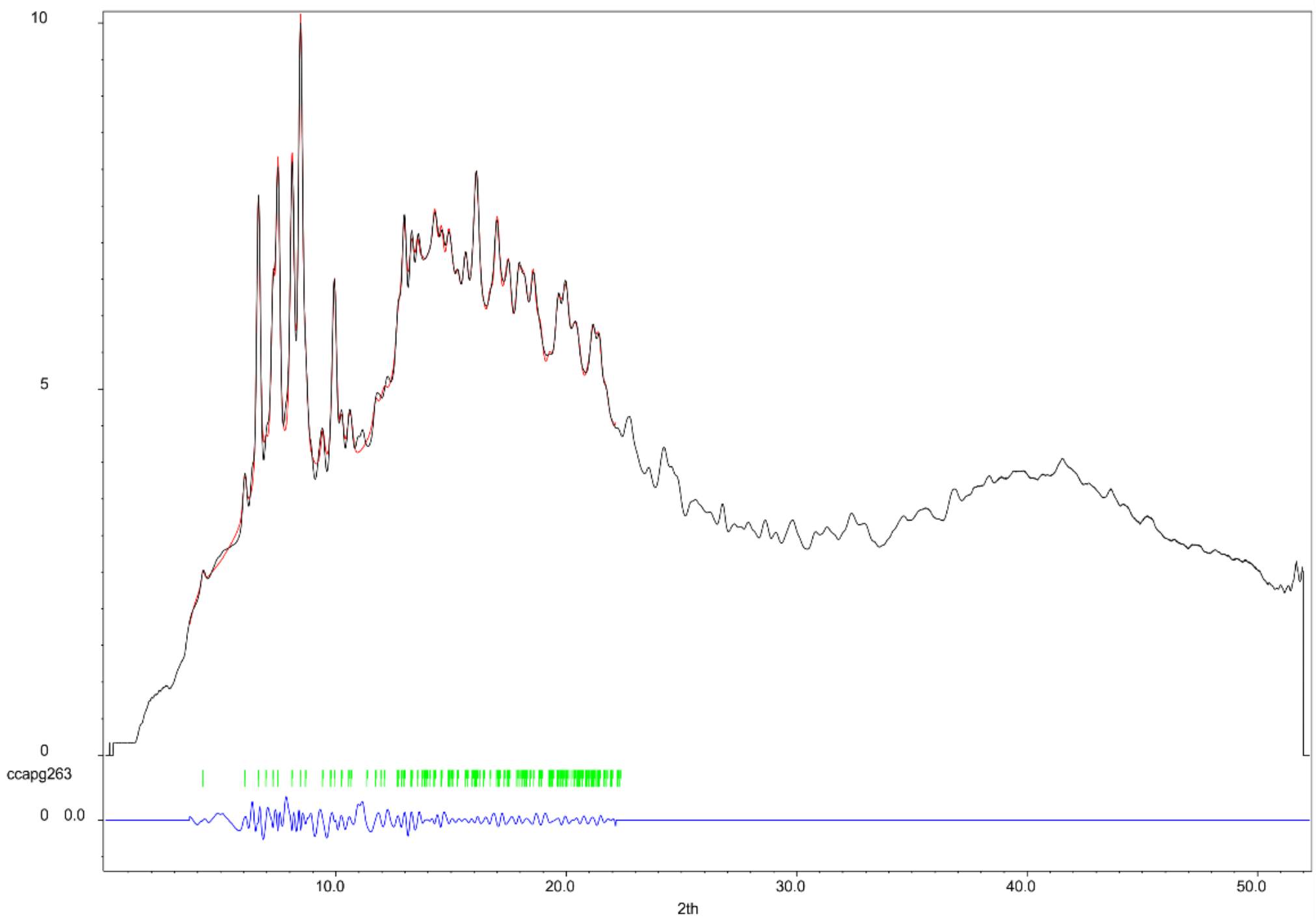

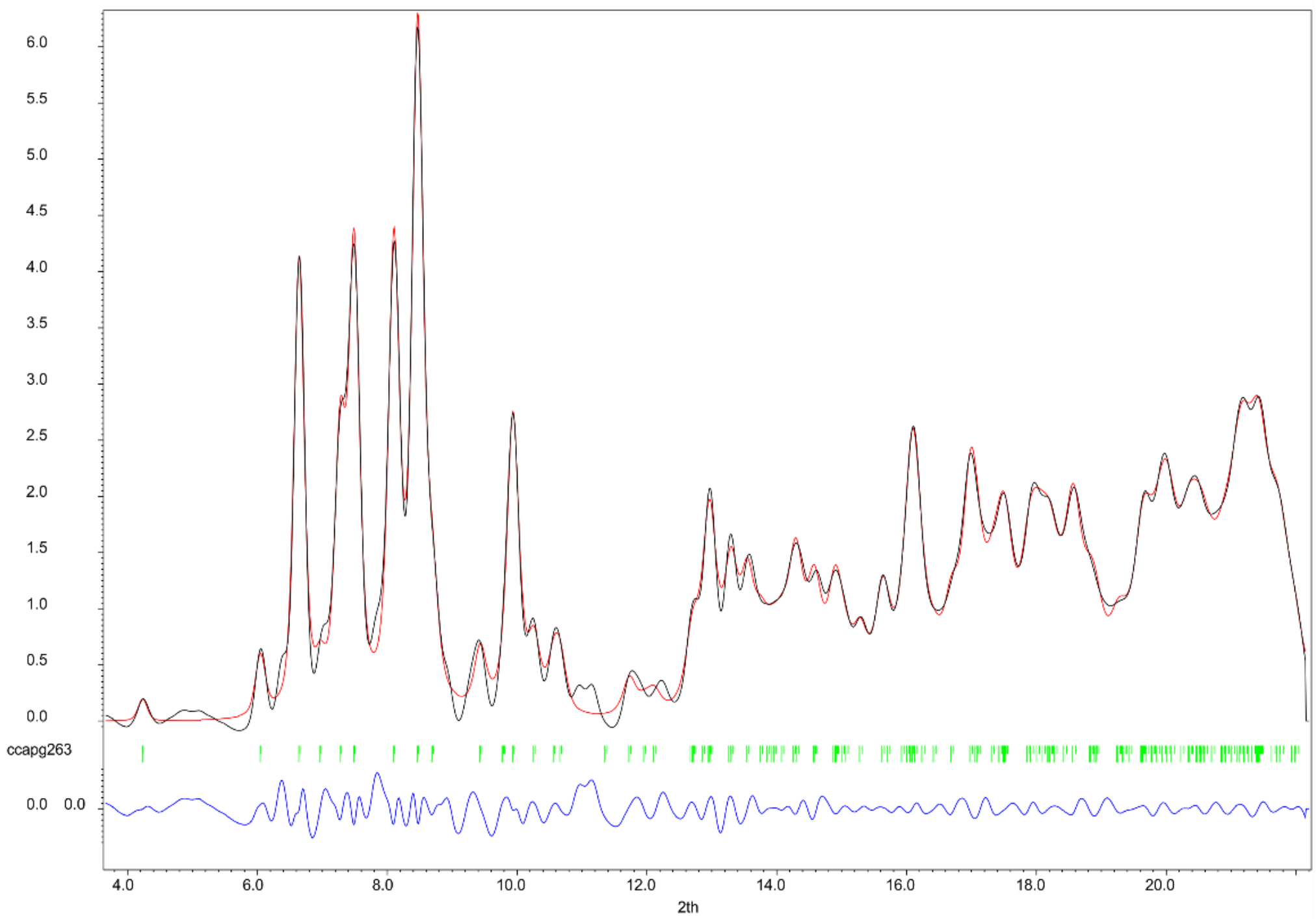

Figure S26. (**Top**) Full PXRD pattern for a sample of **10**; (**Bottom**) Selected range for Le Bail profile fitting (black = observed data, red = calculated profile, green = reflection positions, blue = obs-cal difference, R_p = 1.10%, wR_p = 1.50%).

S4. NMR spectroscopy

Precursors and materials

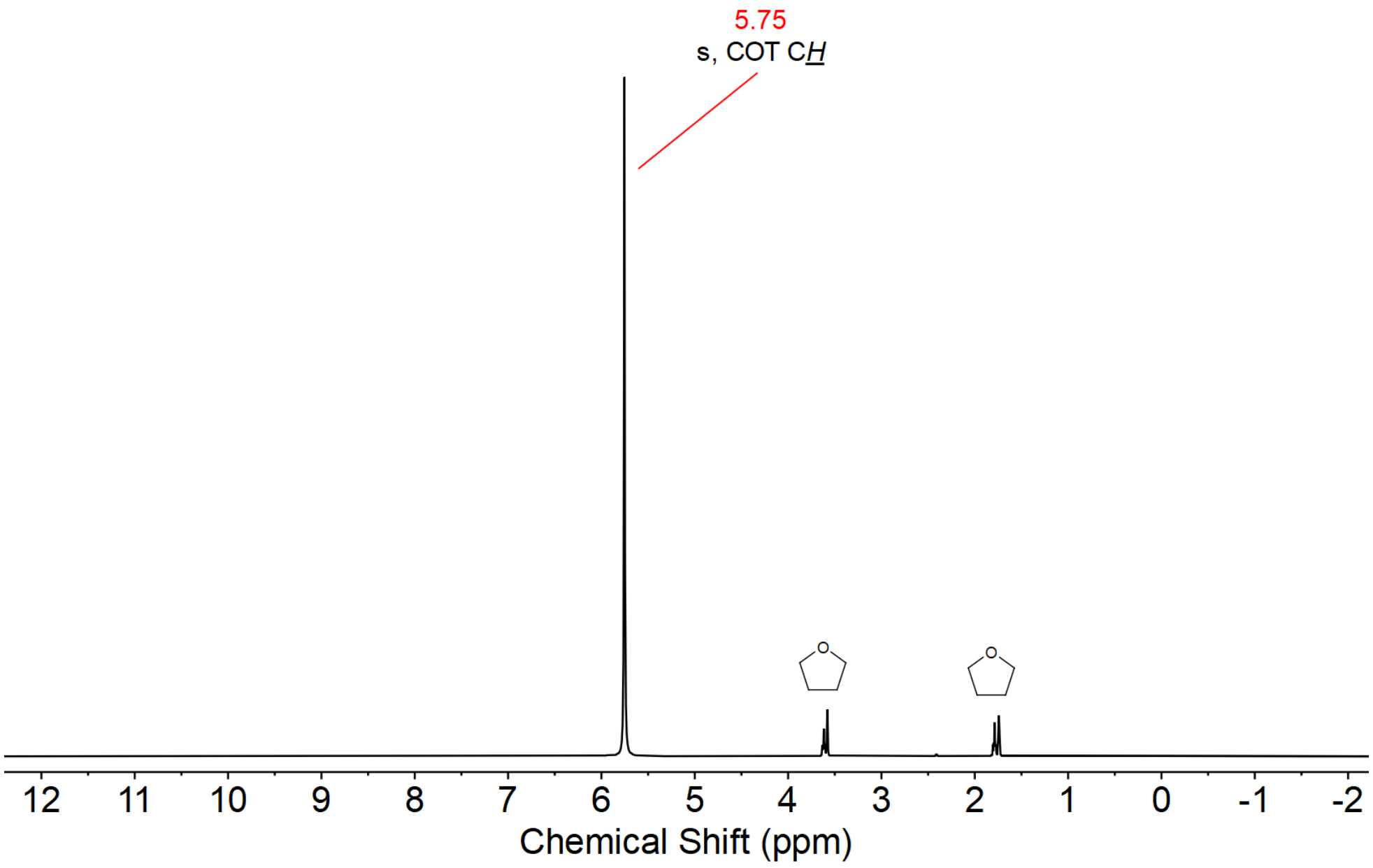

Figure S27. ^{1}H NMR spectrum of K_2COT in d_8-THF showing from +12 to –2 ppm.

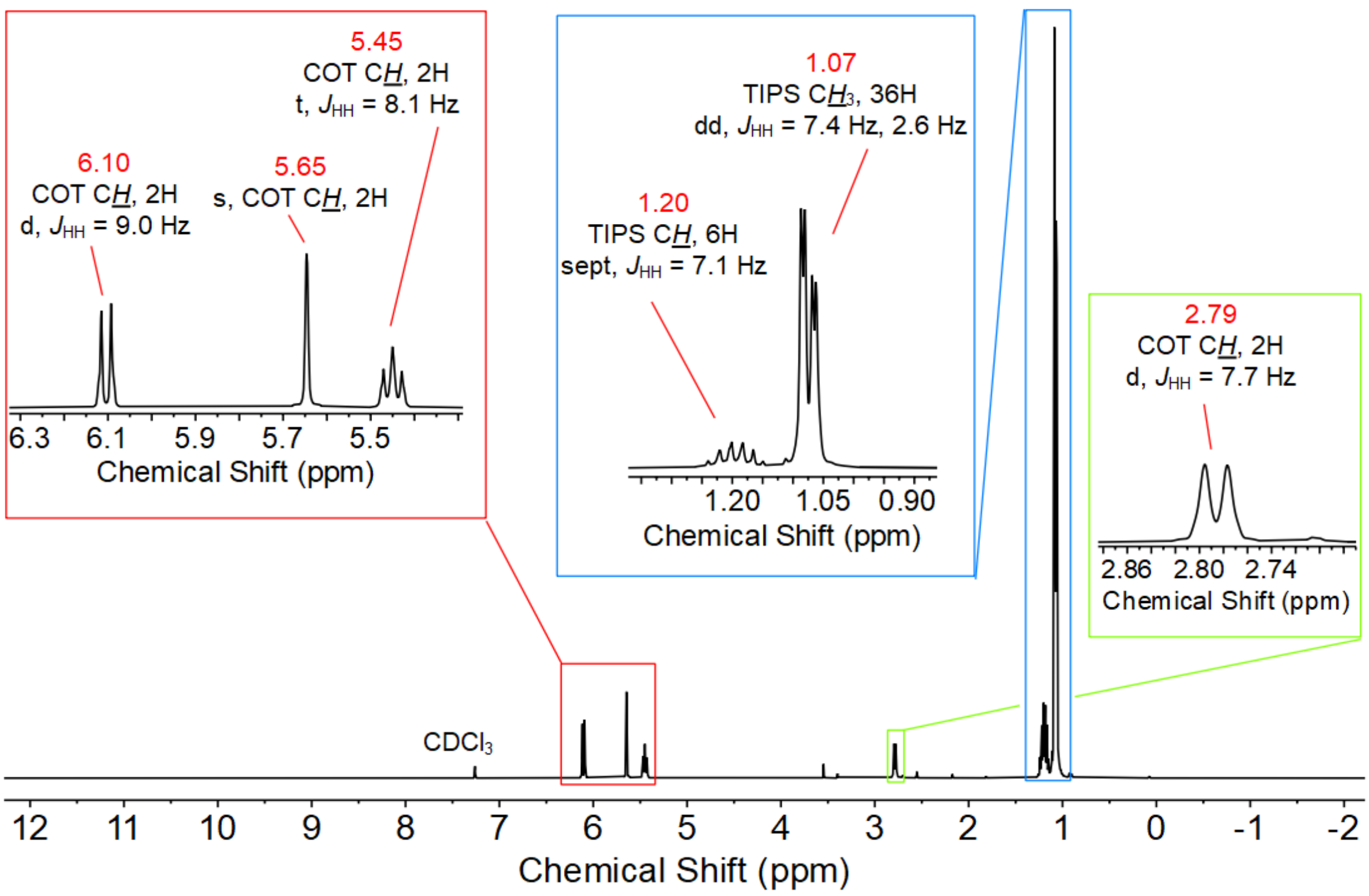

Figure S28. ^{1}H NMR spectrum of $H_2COT^{\dagger\dagger}$ in d_1-chloroform showing from +12 to –2 ppm.

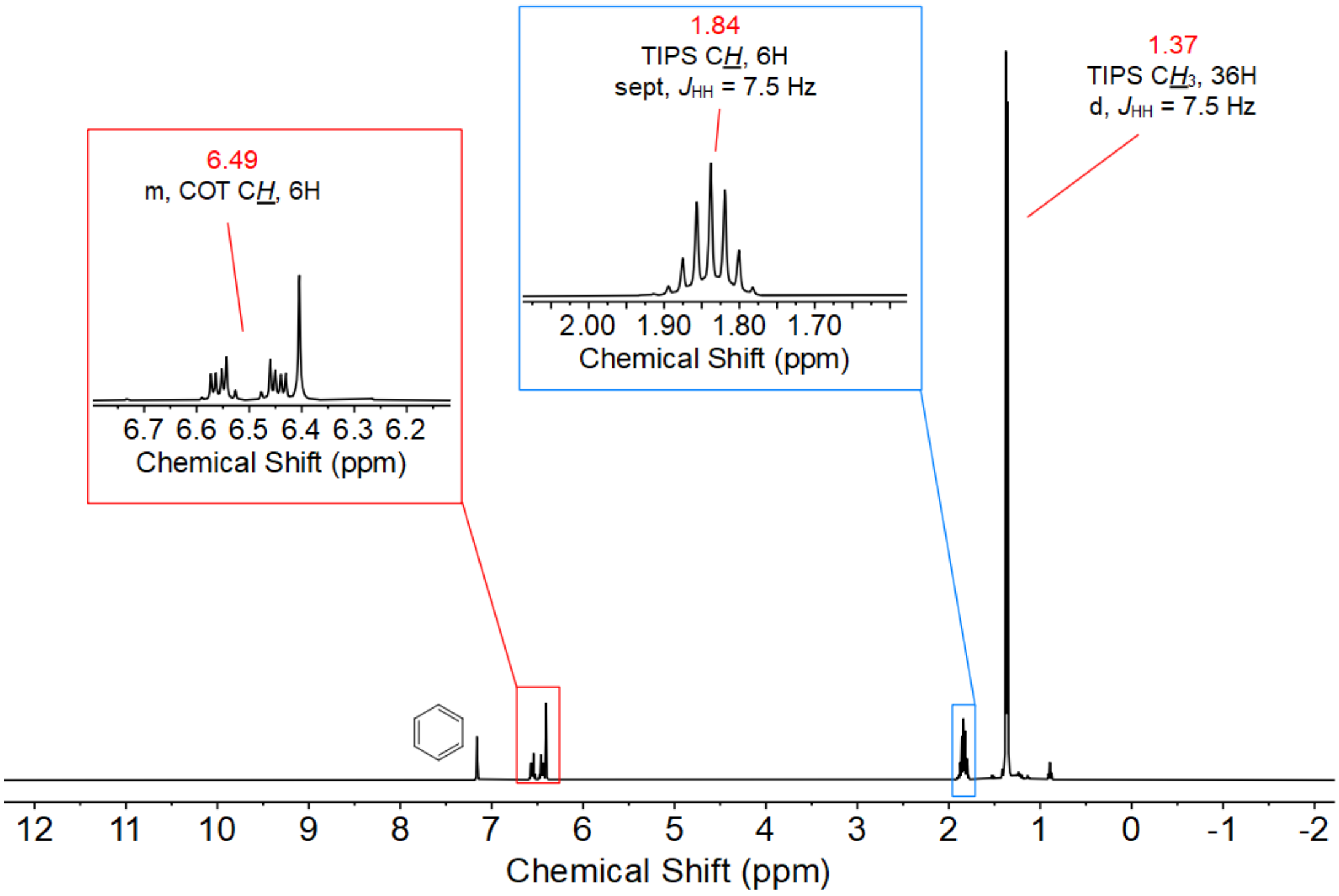

Figure S29. ^{1}H NMR spectrum of $K_2COT^{\dagger\dagger}$ in d_6-benzene showing from +12 to –2 ppm.

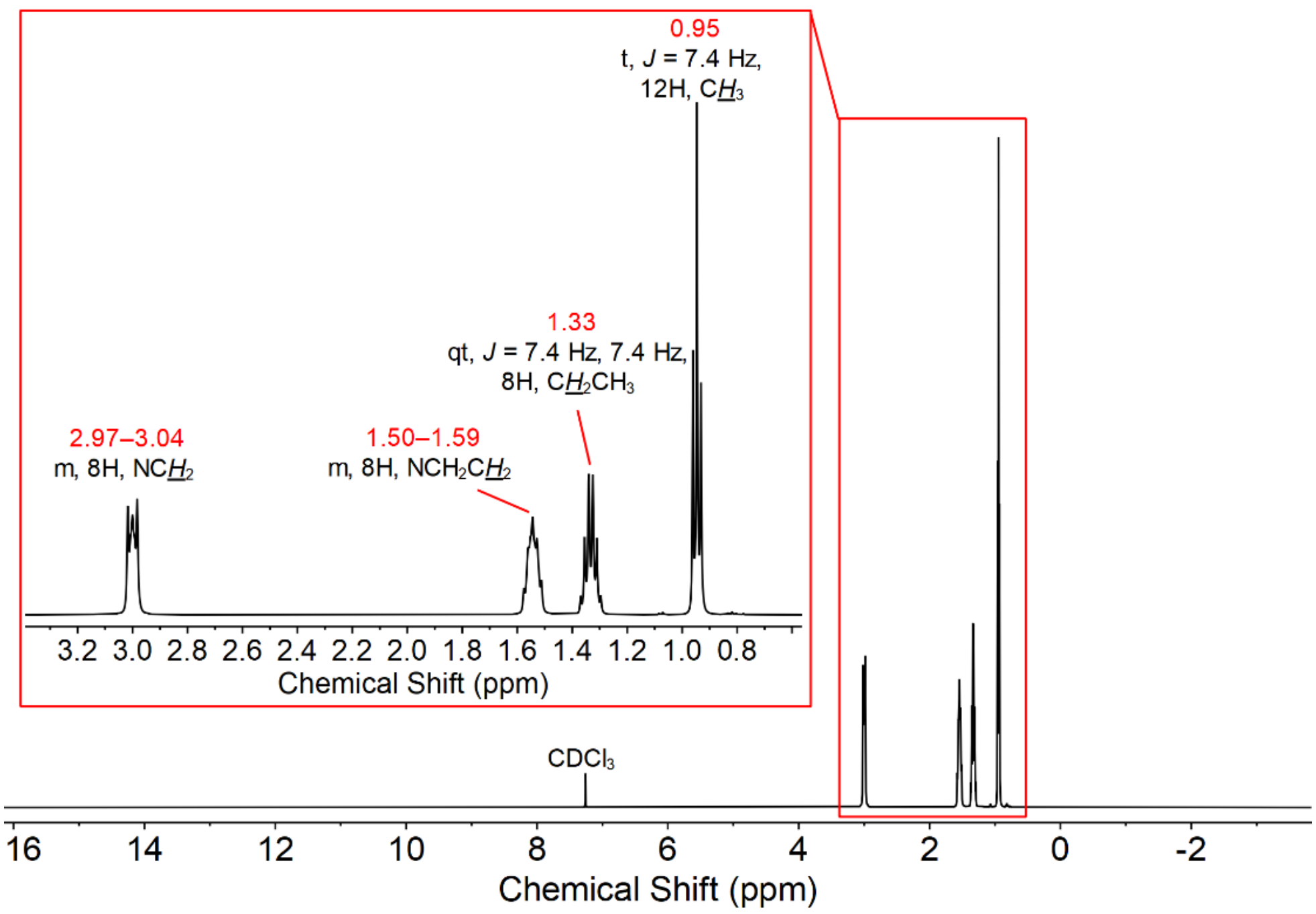

Figure S30. ^{1}H NMR spectrum of [nBu$_4$N][B(C$_6$F$_5$)$_4$] in d-chloroform showing from +16 to –4 ppm.

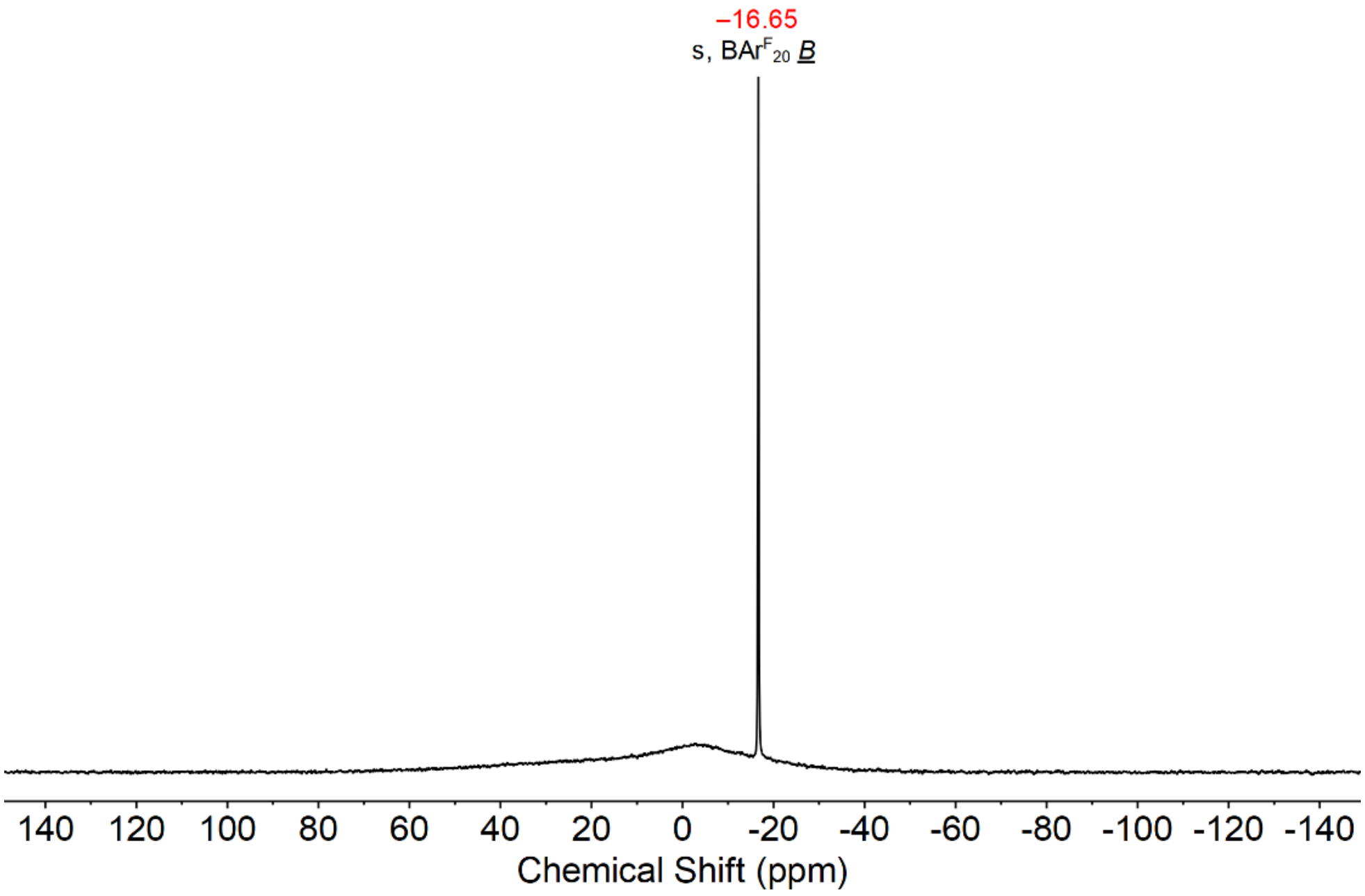

Figure S31. ^{11}B NMR spectrum of [nBu$_4$N][B(C$_6$F$_5$)$_4$] in *d*-chloroform showing from +150 to –150 ppm.

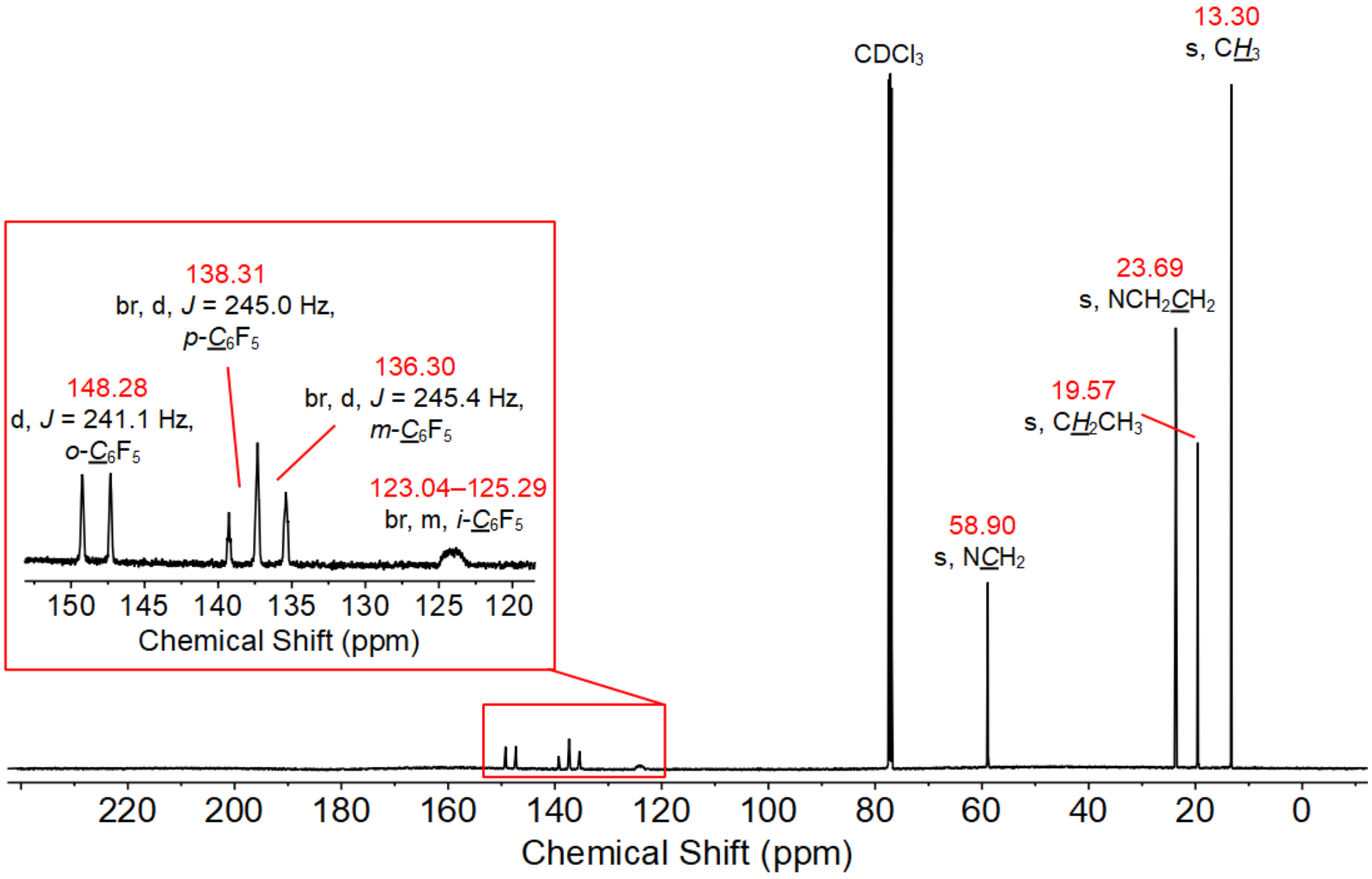

Figure S32. ^{13}C{^{1}H} NMR spectrum of [nBu$_4$N][B(C$_6$F$_5$)$_4$] in *d*-chloroform showing from +240 to –10 ppm.

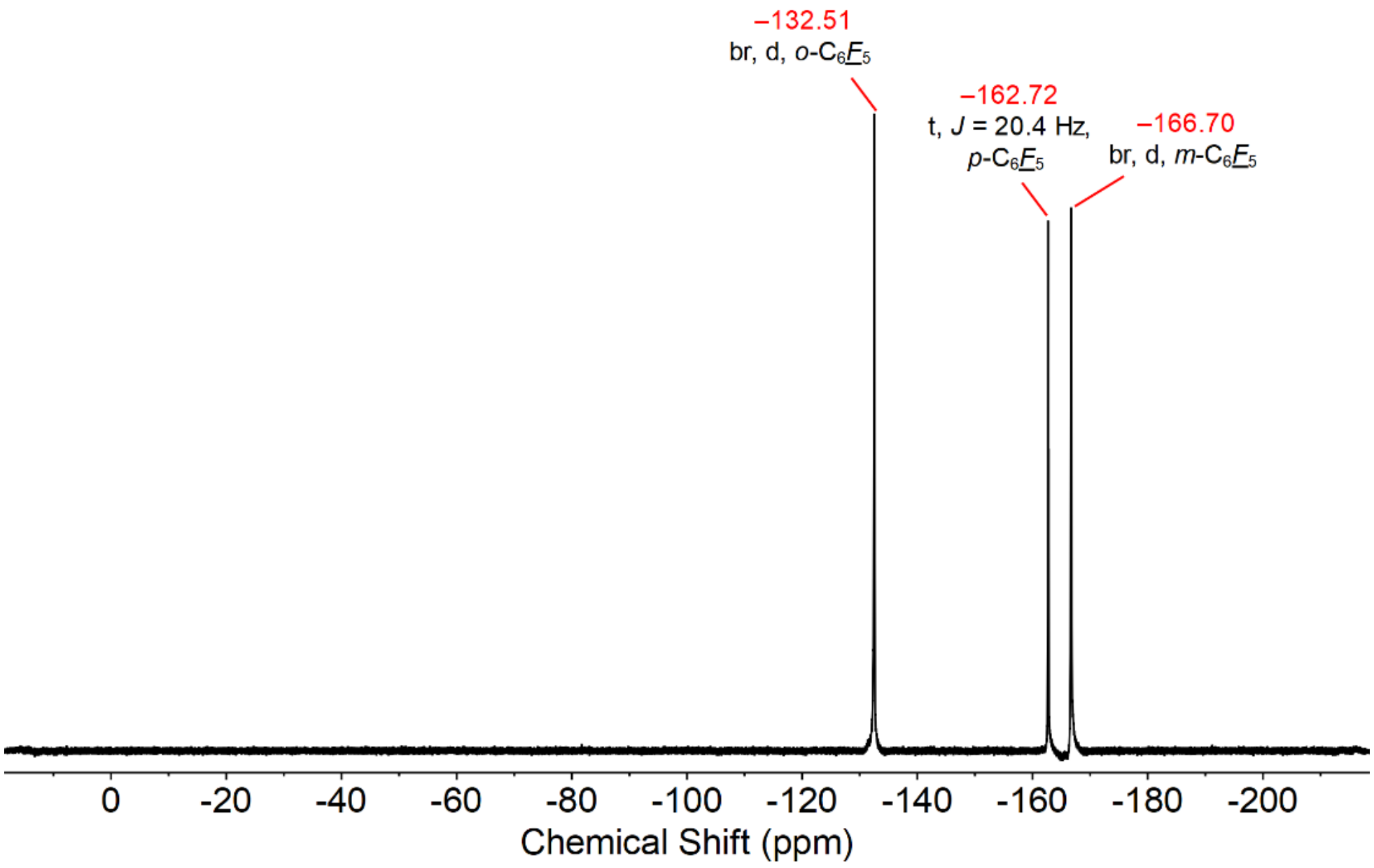

Figure S33. ^{19}F NMR spectrum of [nBu$_4$N][B(C_6F_5)$_4$] in *d*-chloroform showing from +20 to –220 ppm.

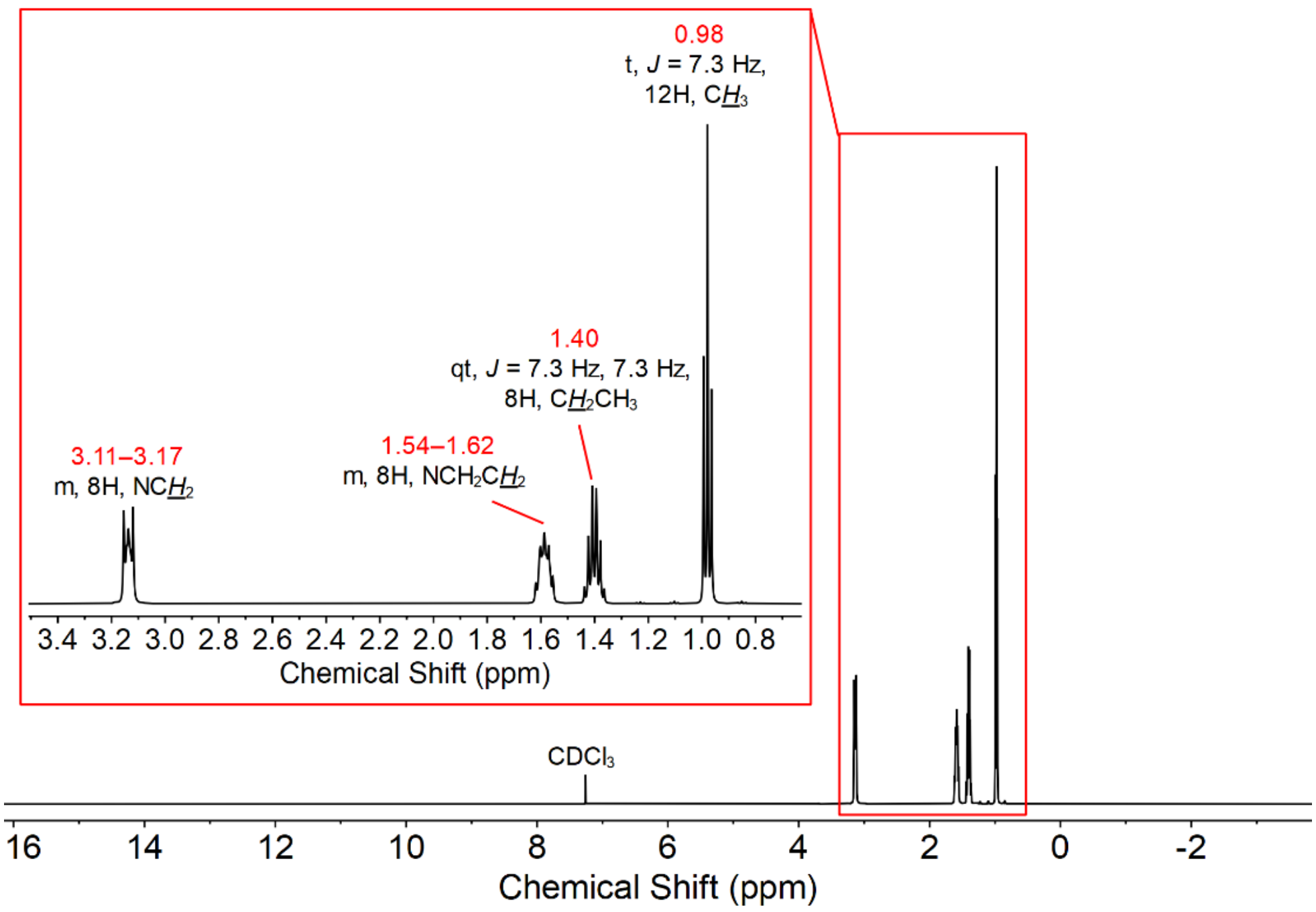

Figure S34. ^{1}H NMR spectrum of [nBu$_4$N][PF_6] in *d*-chloroform showing from +16 to –4 ppm.

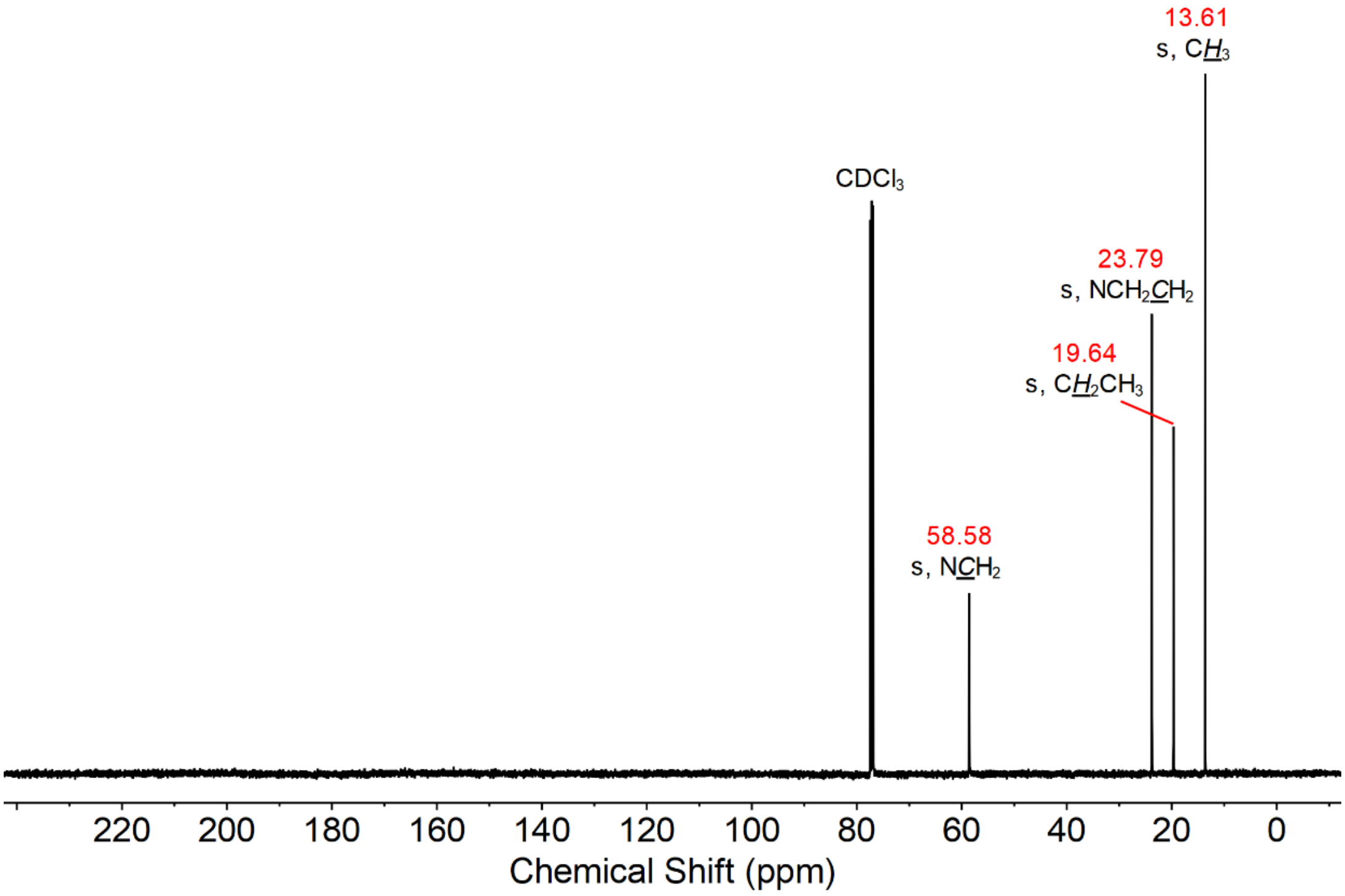

Figure S35. $^{13}C\{^{1}H\}$ NMR spectrum of [nBu$_4$N][PF$_6$] in *d*-chloroform showing from +240 to –10 ppm.

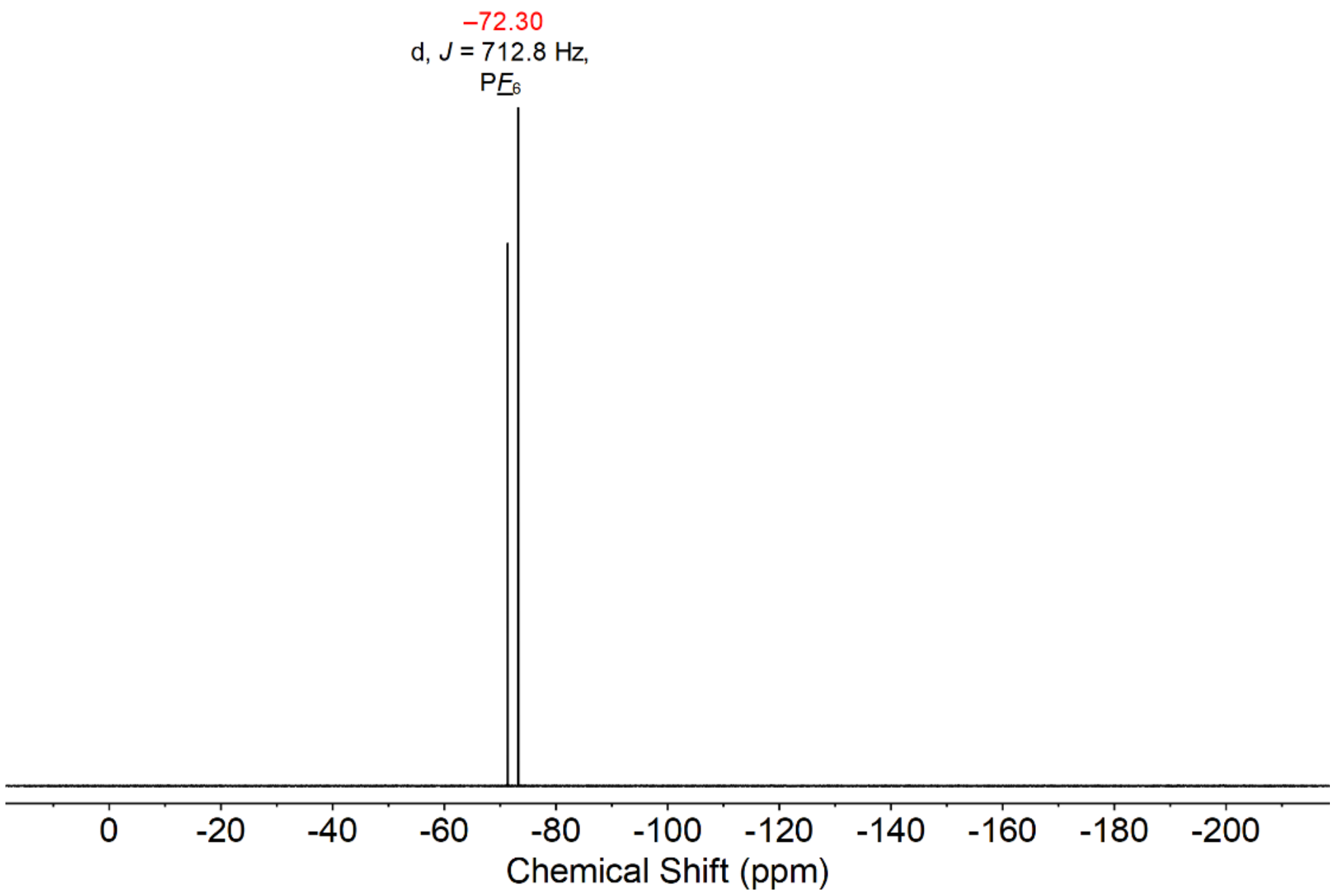

Figure S36. ^{19}F NMR spectrum of [nBu$_4$N][PF$_6$] in *d*-chloroform showing from +20 to –220 ppm.

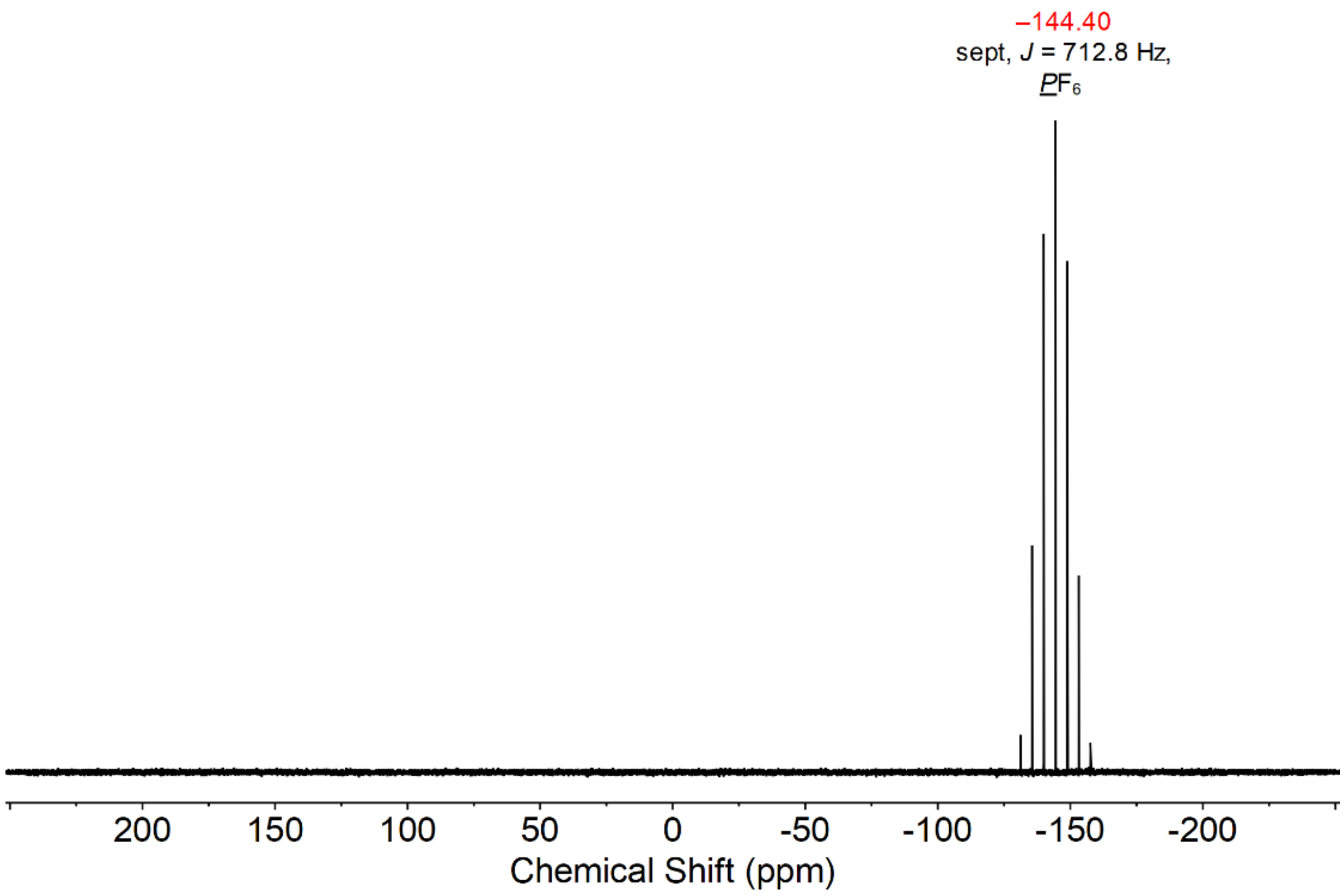

Figure S37. ^{31}P NMR spectrum of [nBu$_4$N][PF$_6$] in d-chloroform showing from +250 to –250 ppm.

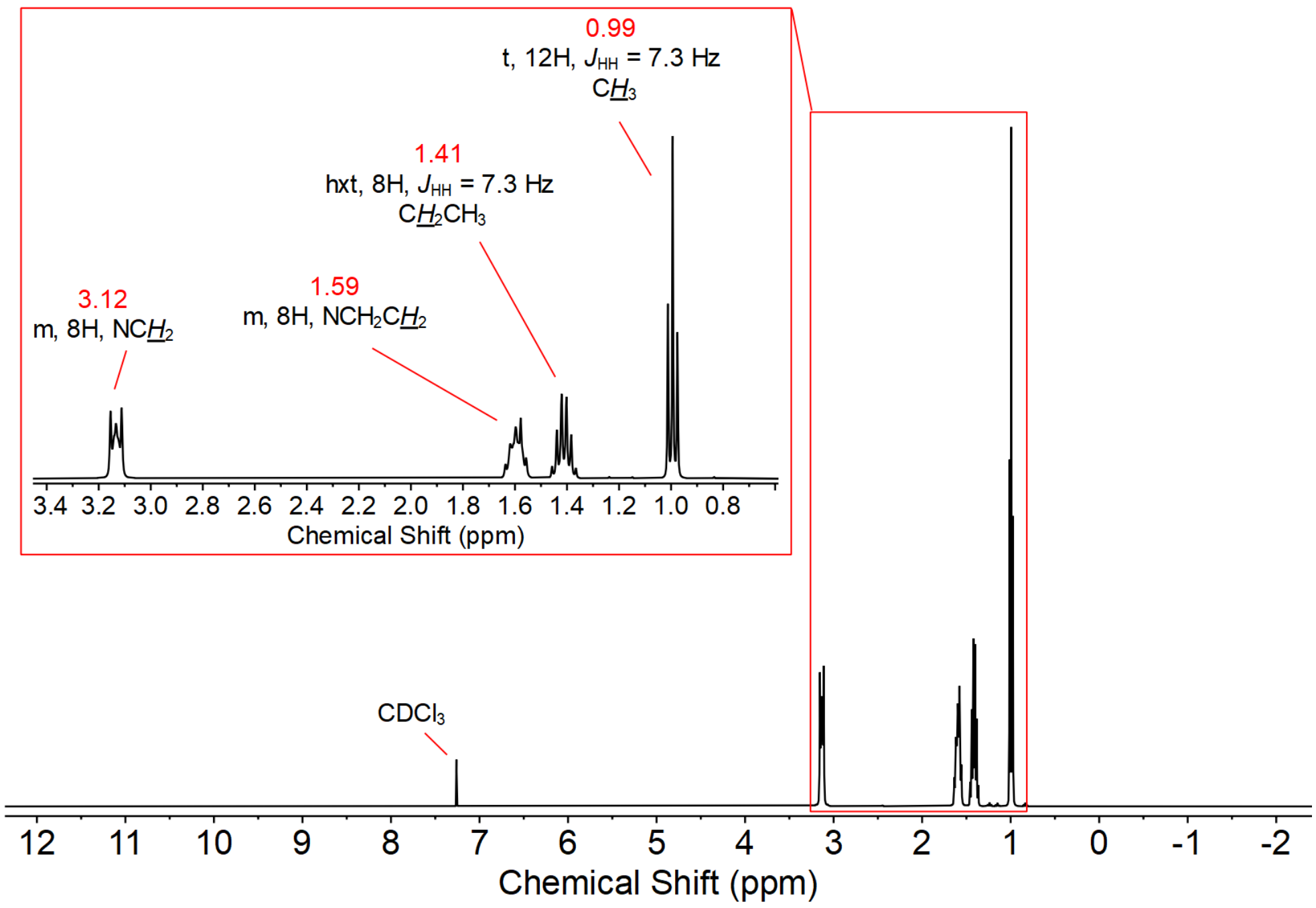

Figure S38. ^{1}H NMR spectrum of [nBu$_4$N][SbF$_6$] in d-chloroform showing from +12 to –2 ppm.

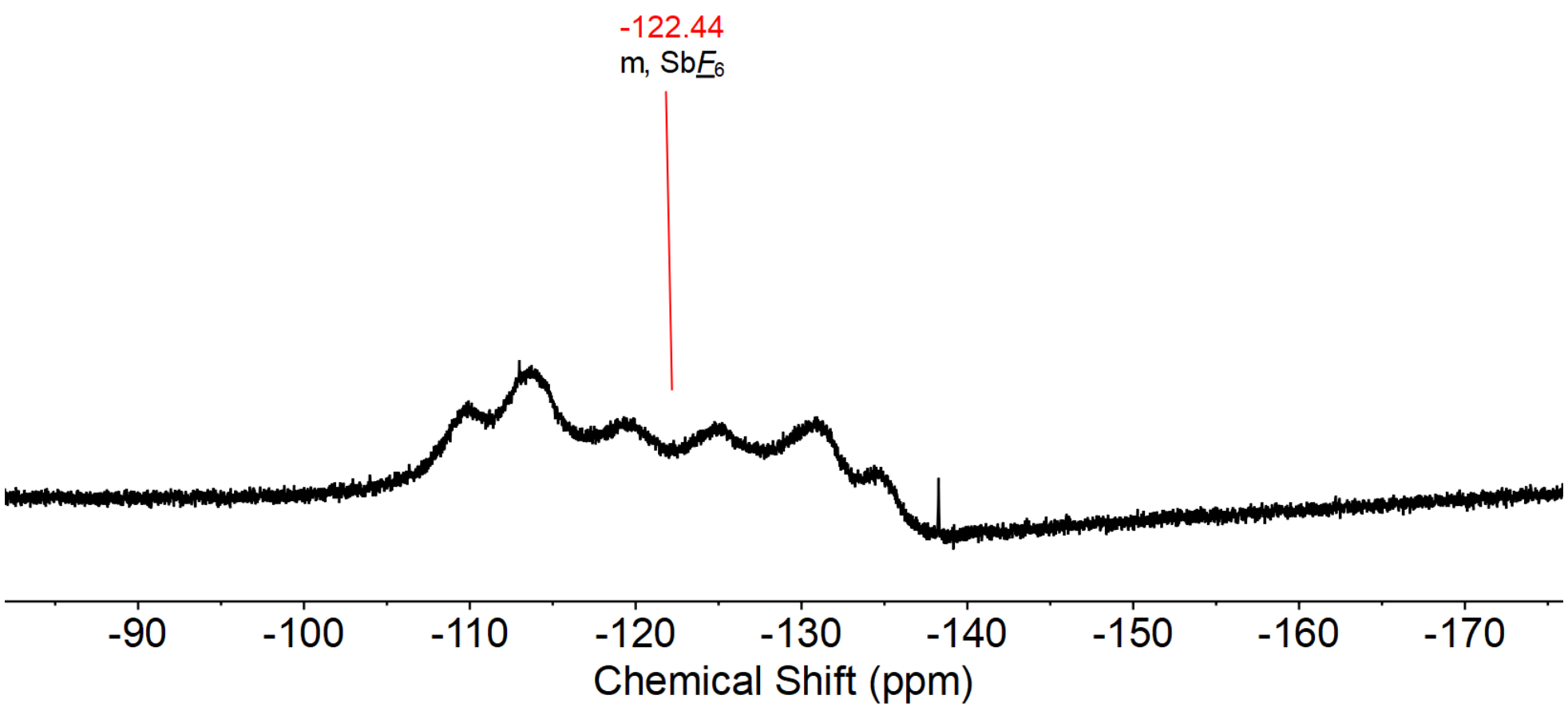

Figure S39. $^{19}F\{^{1}H\}$ NMR spectrum of [nBu$_4$N][SbF$_6$] in *d*-chloroform. The multiplet structure arises from coupling with ^{121}Sb (I = 5/2) and ^{123}Sb (I = 7/2) nuclei.

*Complexes **1–6**, **9**, and **10***

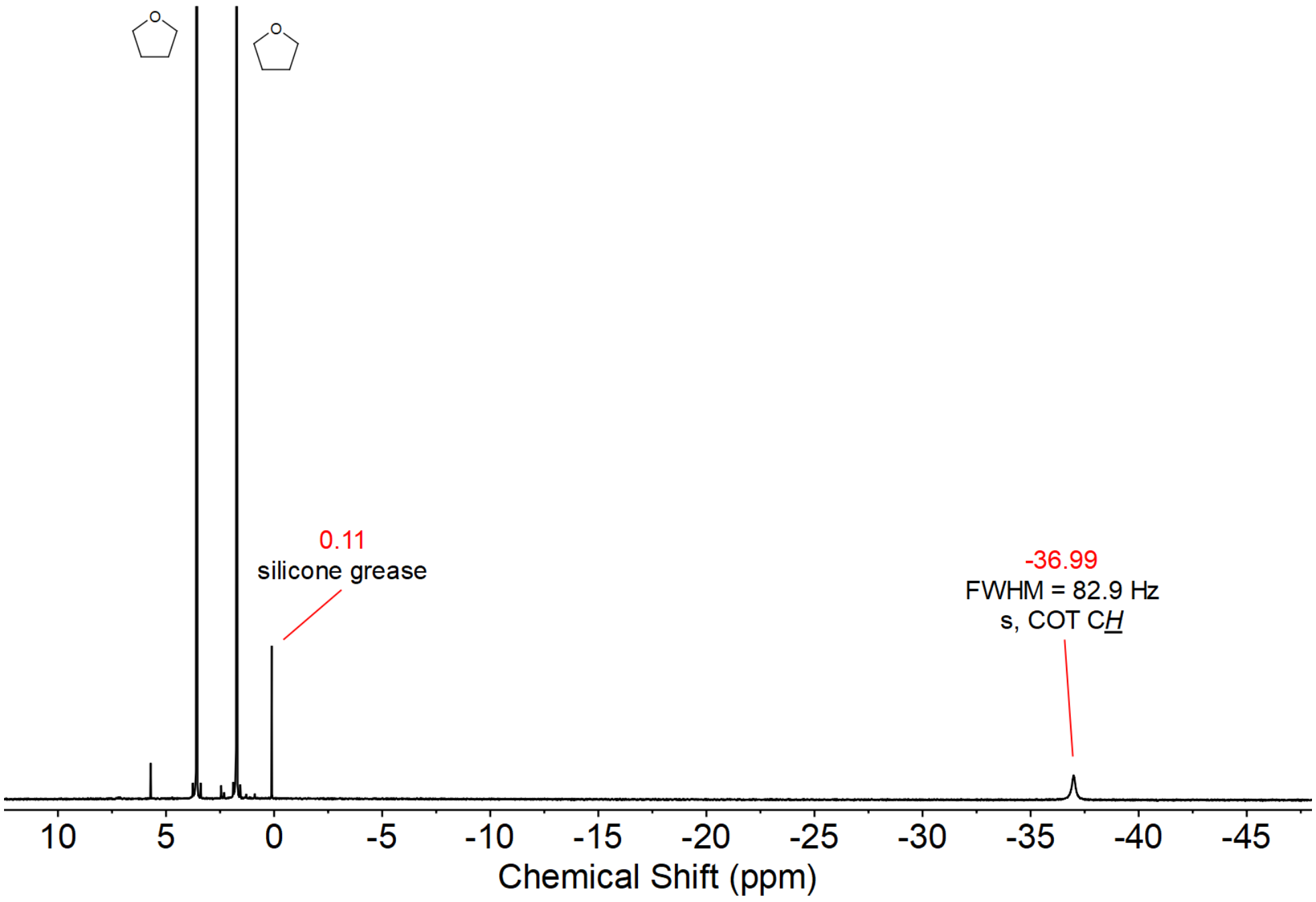

Figure S40. ^{1}H NMR spectrum of **1** in d_8-THF showing from +12 to –48 ppm.

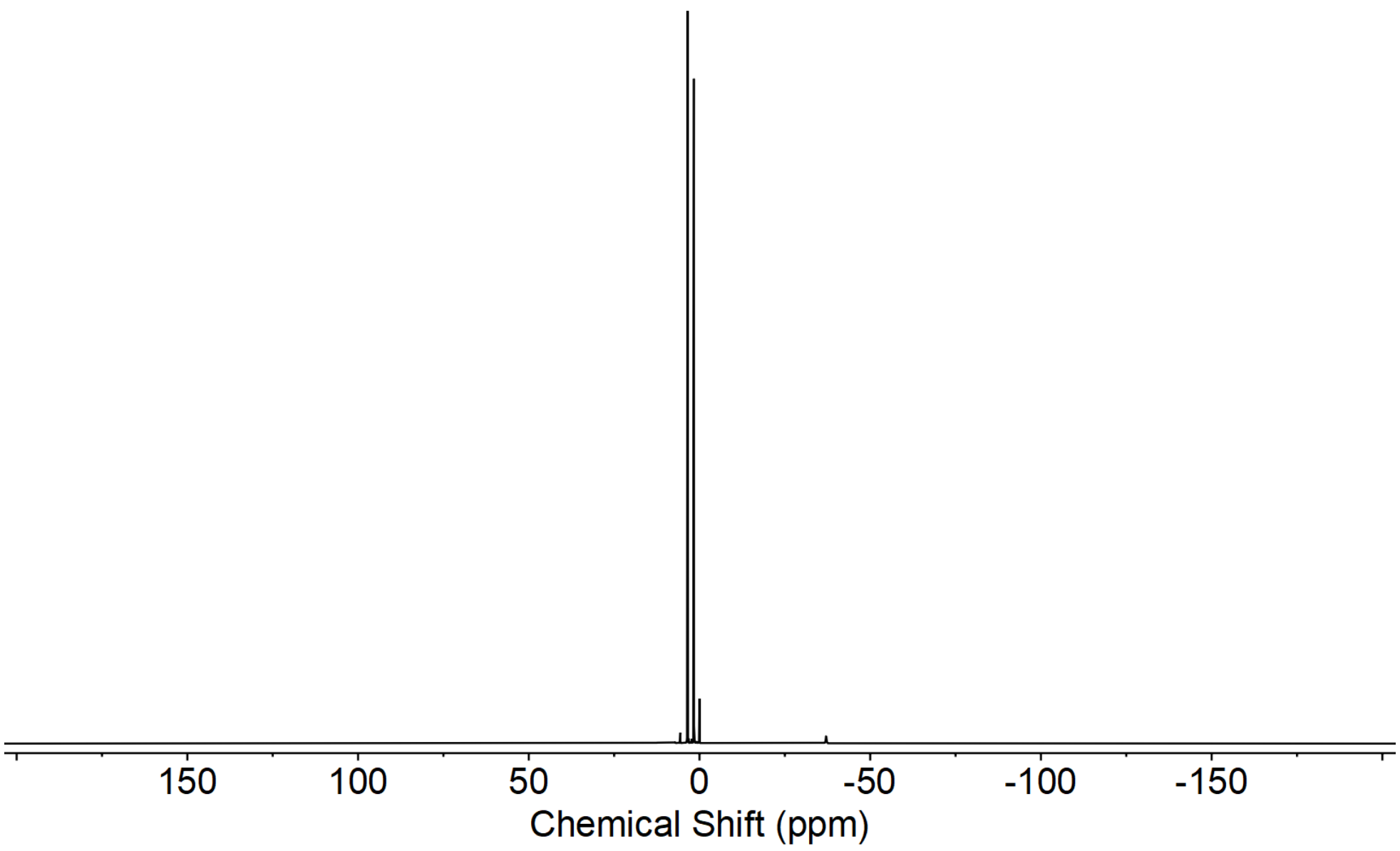

Figure S41. ^{1}H NMR spectrum of **1** in d_8-THF showing from +200 to –200 ppm.

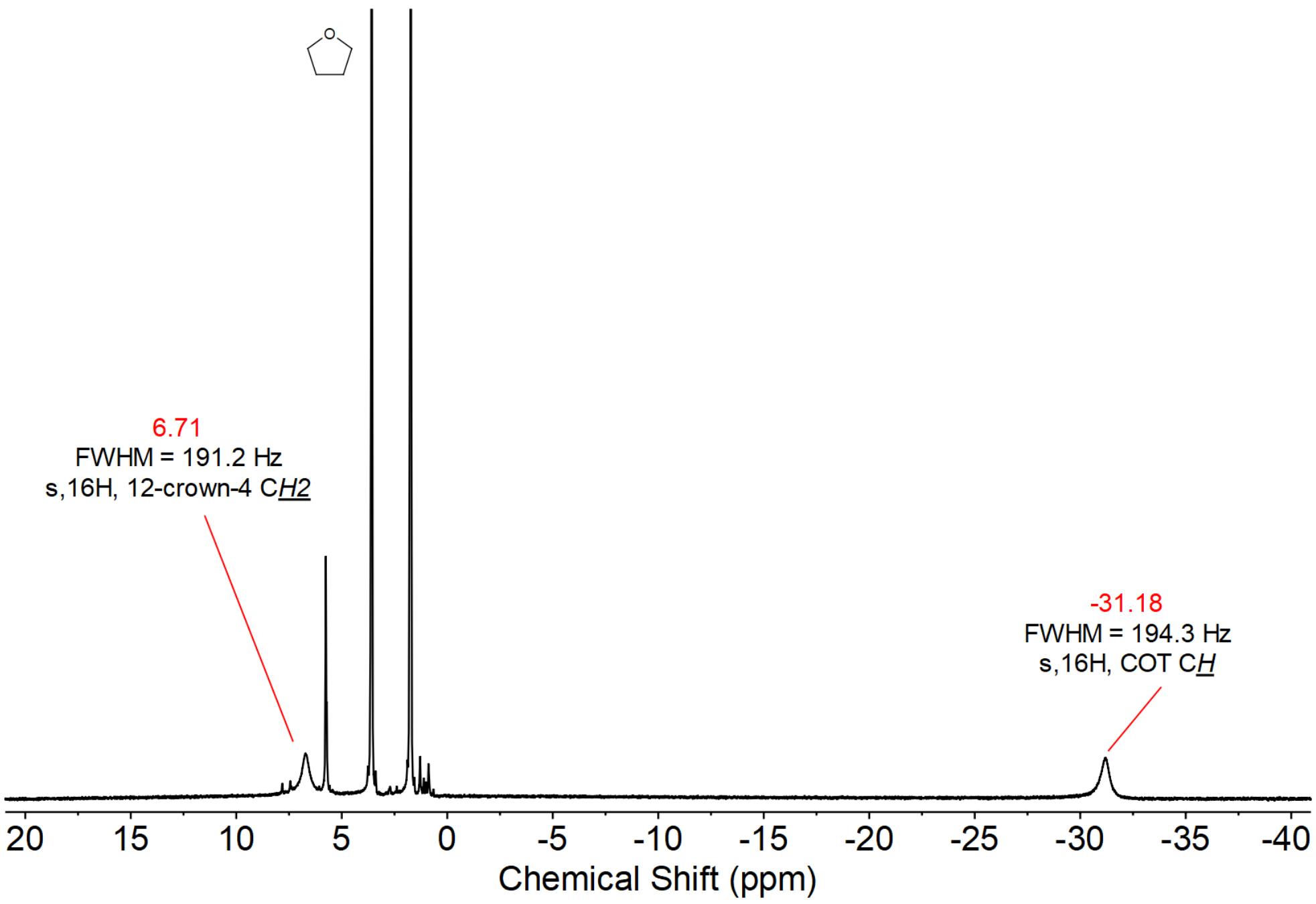

Figure S42. ^{1}H NMR spectrum of **2** in d_8-THF showing from +20 to –40 ppm.

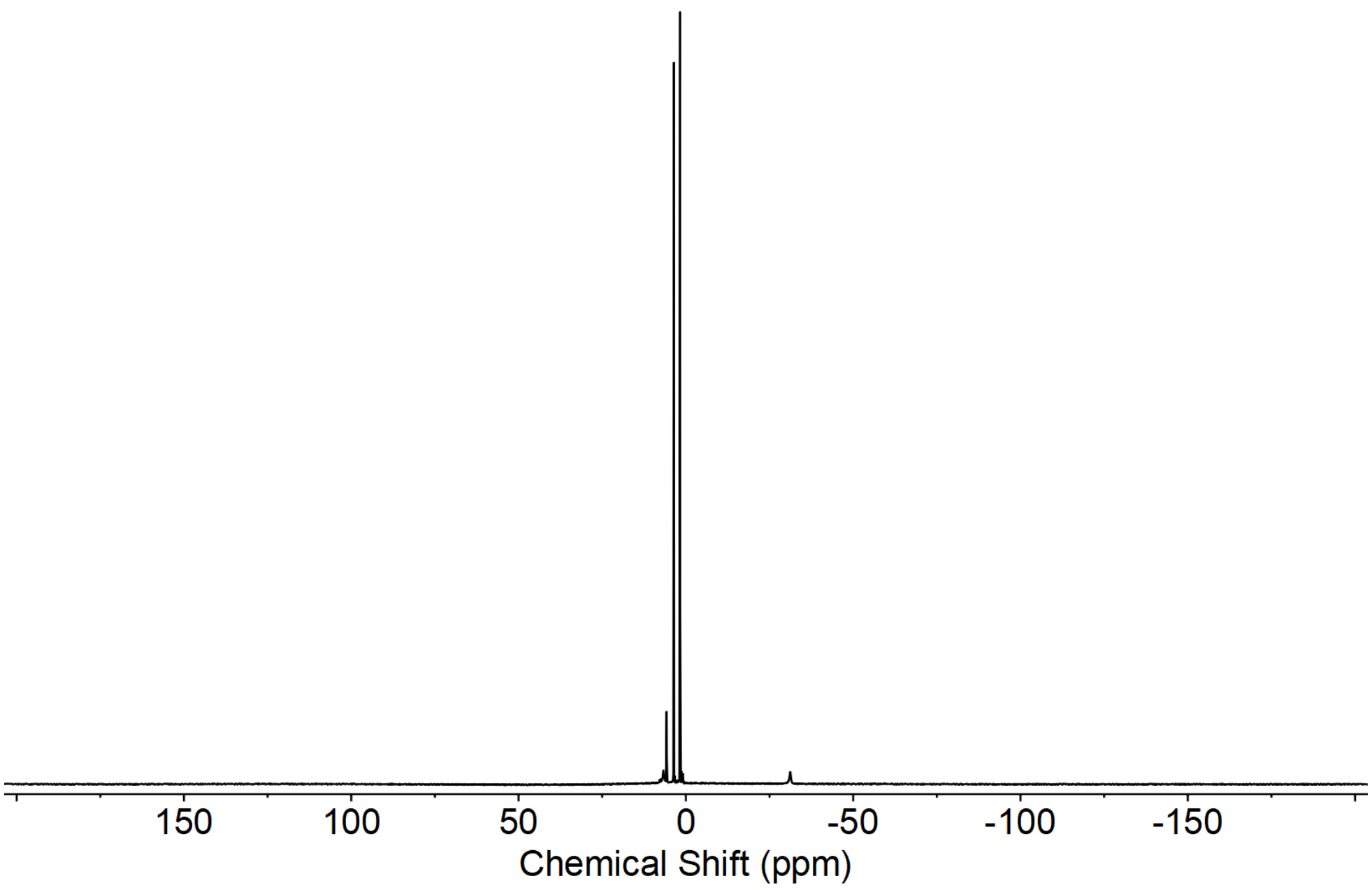

Figure S43. ^{1}H NMR spectrum of **2** in d_8-THF showing from +200 to –200 ppm.

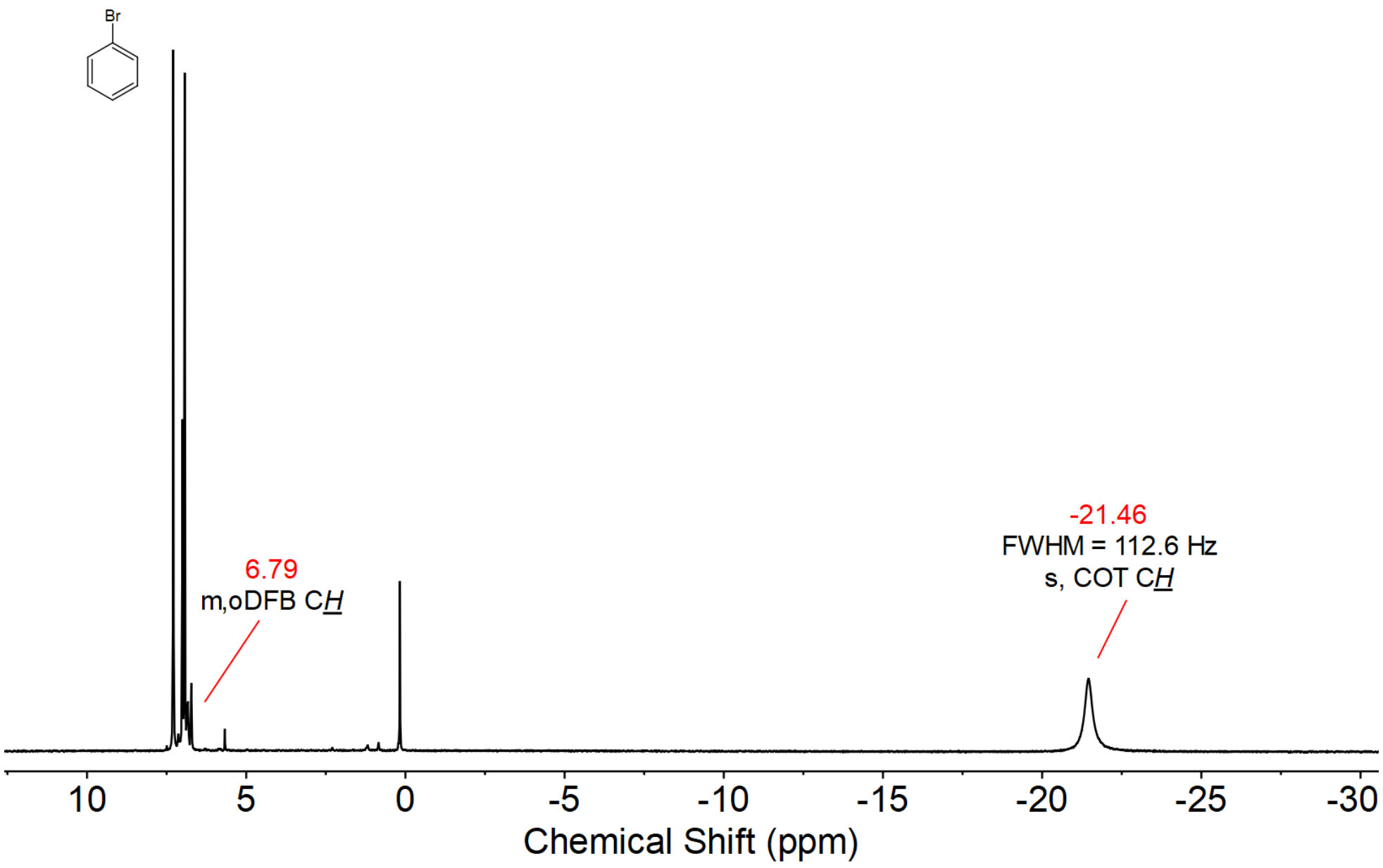

Figure S44. ^{1}H NMR spectrum of **3** in d_5-bromobenzene showing from +12 to –30 ppm.

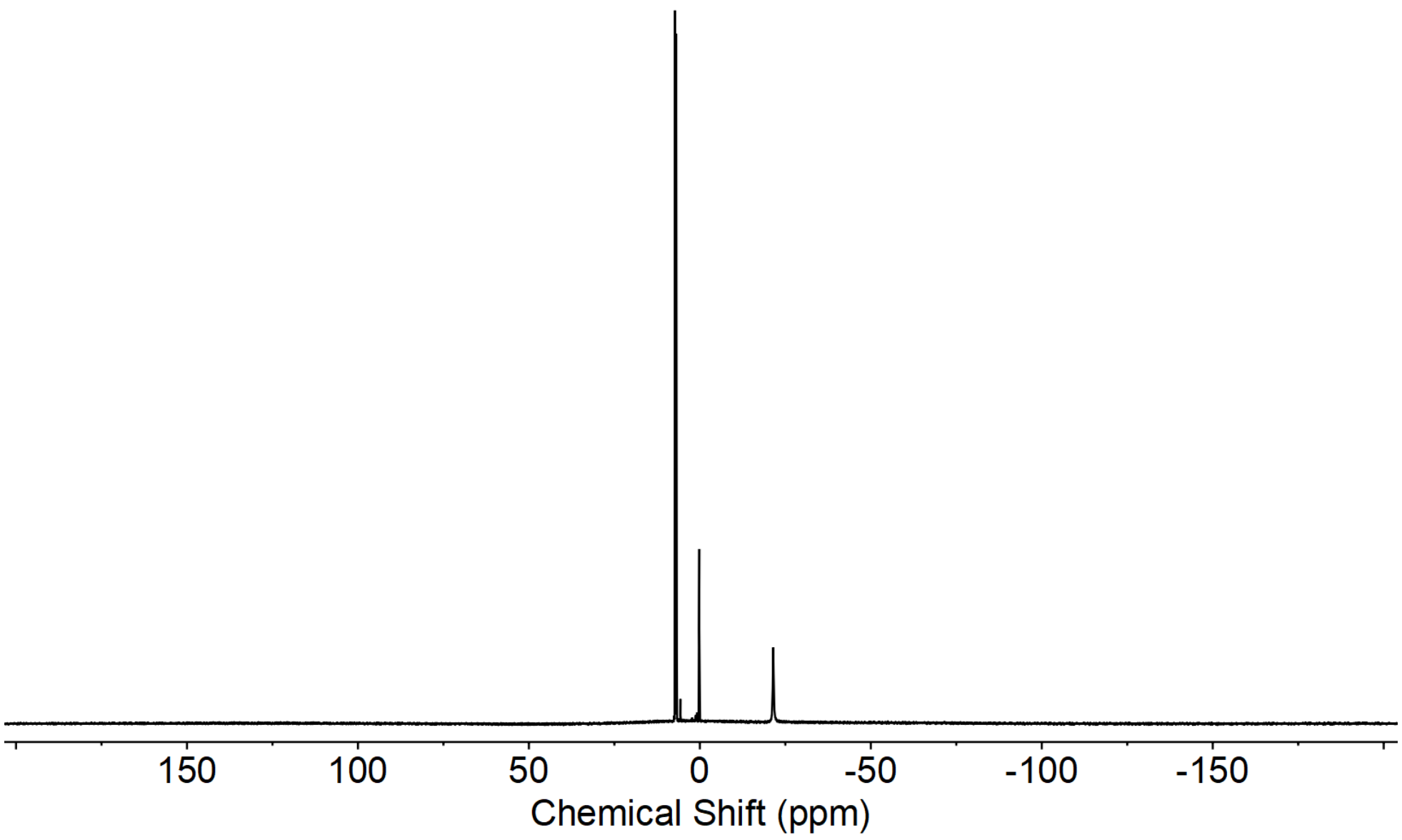

Figure S45. ^{1}H NMR spectrum of **3** in d_5-bromobenzene showing from +200 to –200 ppm.

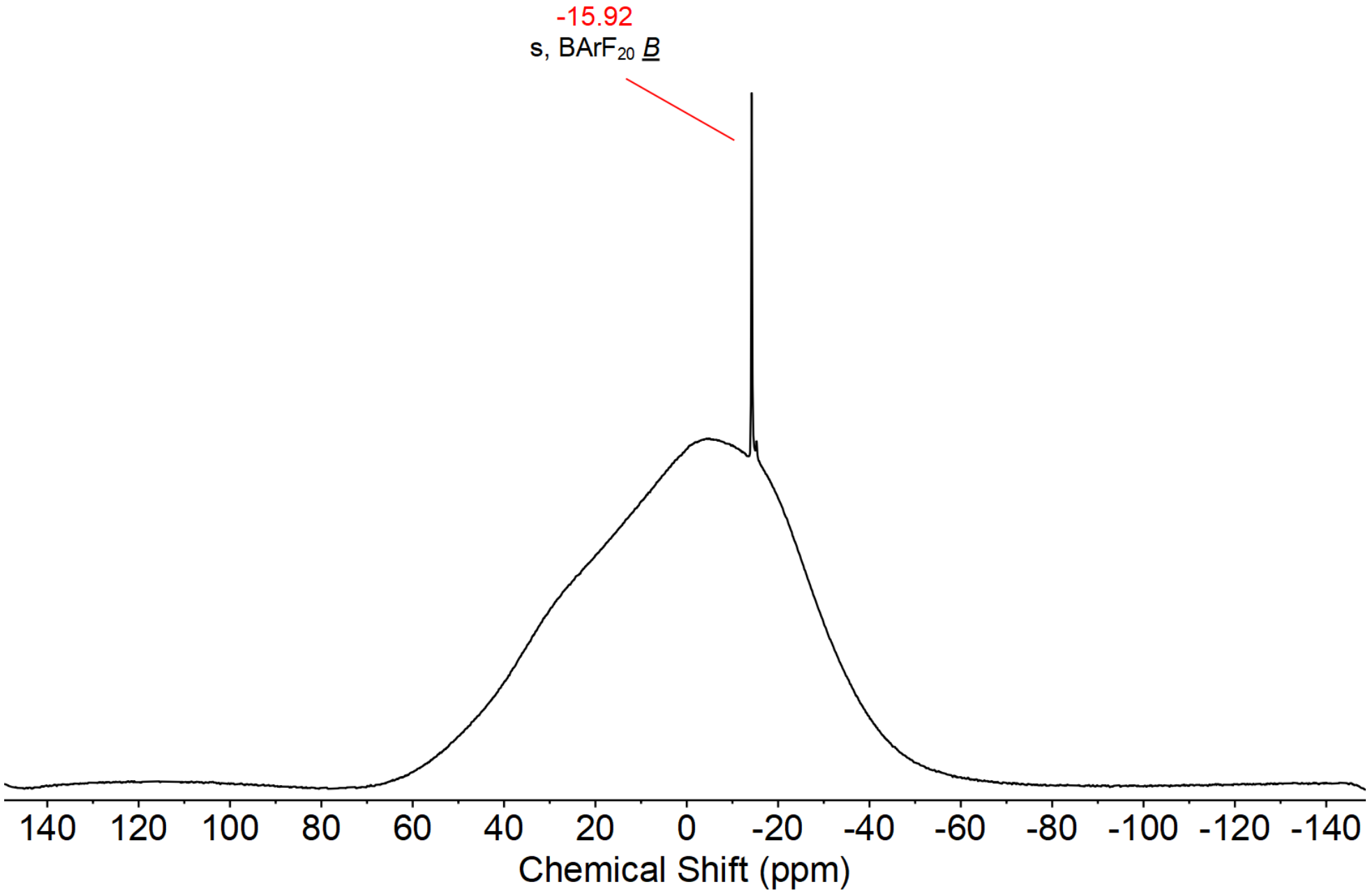

Figure S46. $^{11}B\{^{1}H\}$ NMR spectrum of **3** in d_5-bromobenzene.

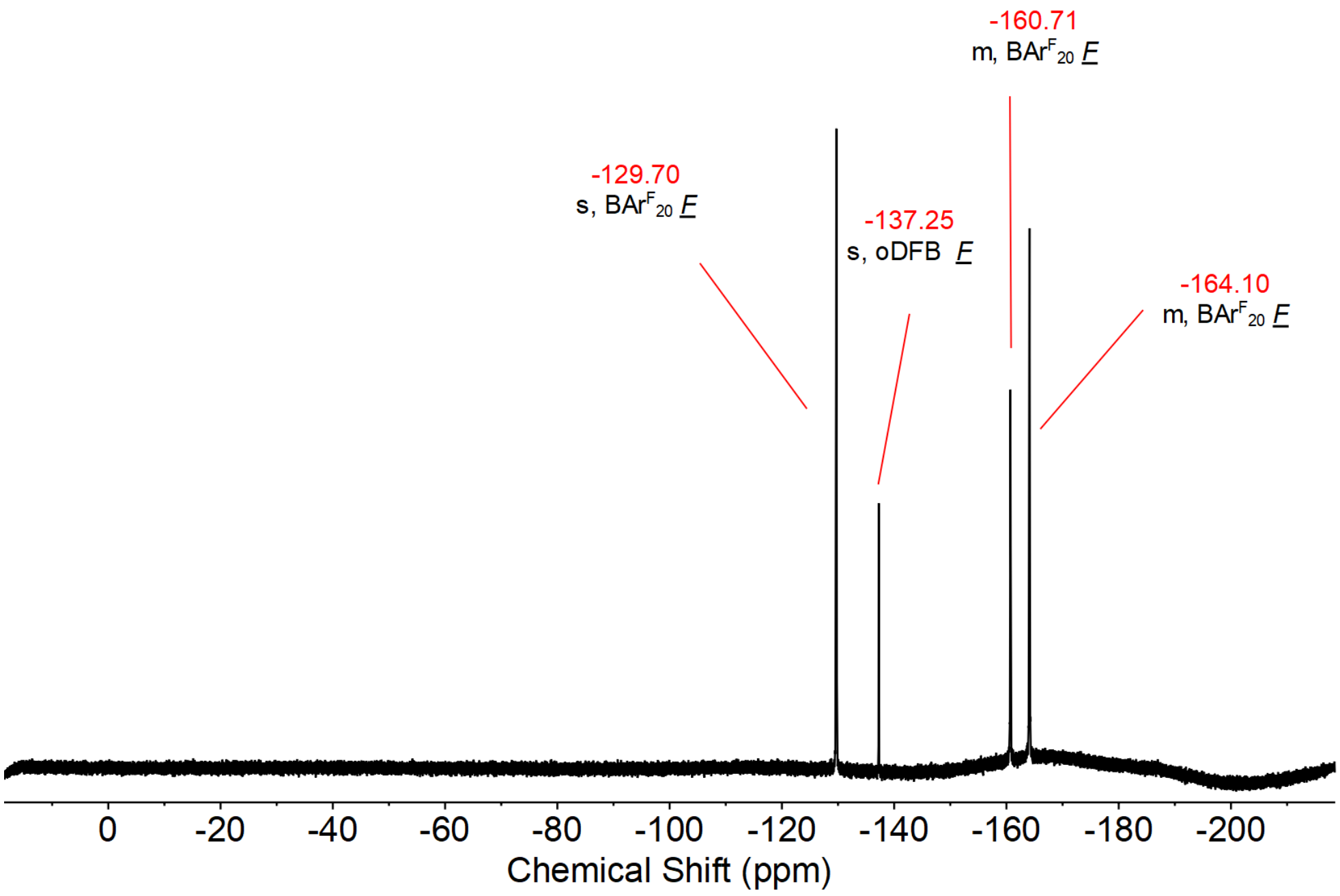

Figure S47. $^{19}F\{^{1}H\}$ NMR spectrum of **3** in d_5-bromobenzene.

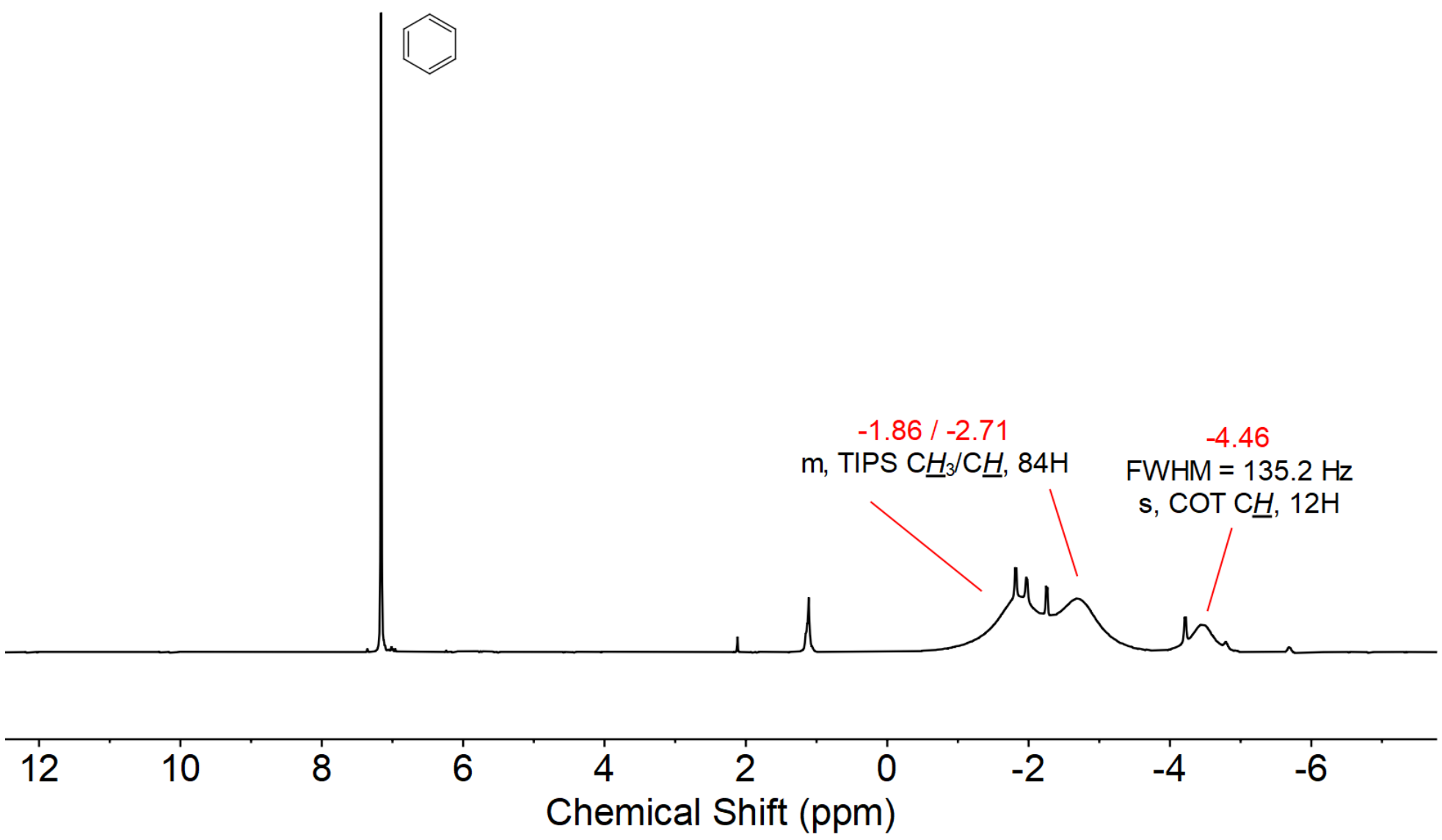

Figure S48. ^{1}H NMR spectrum of **4** in d_6-benzene showing from +12 to –7 ppm.

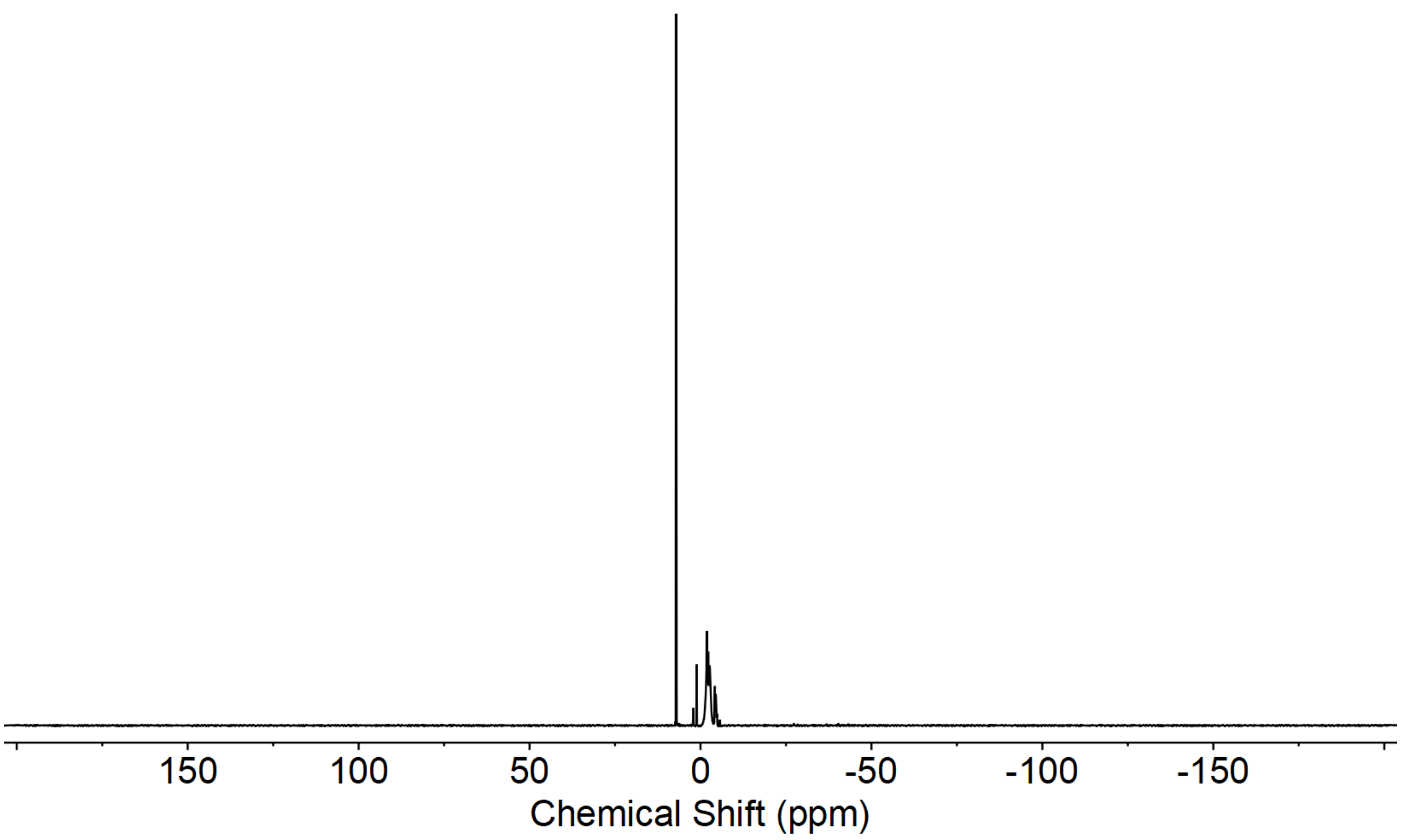

Figure S49. ^{1}H NMR spectrum of **4** in d_6-benzene showing from +200 to –200 ppm.

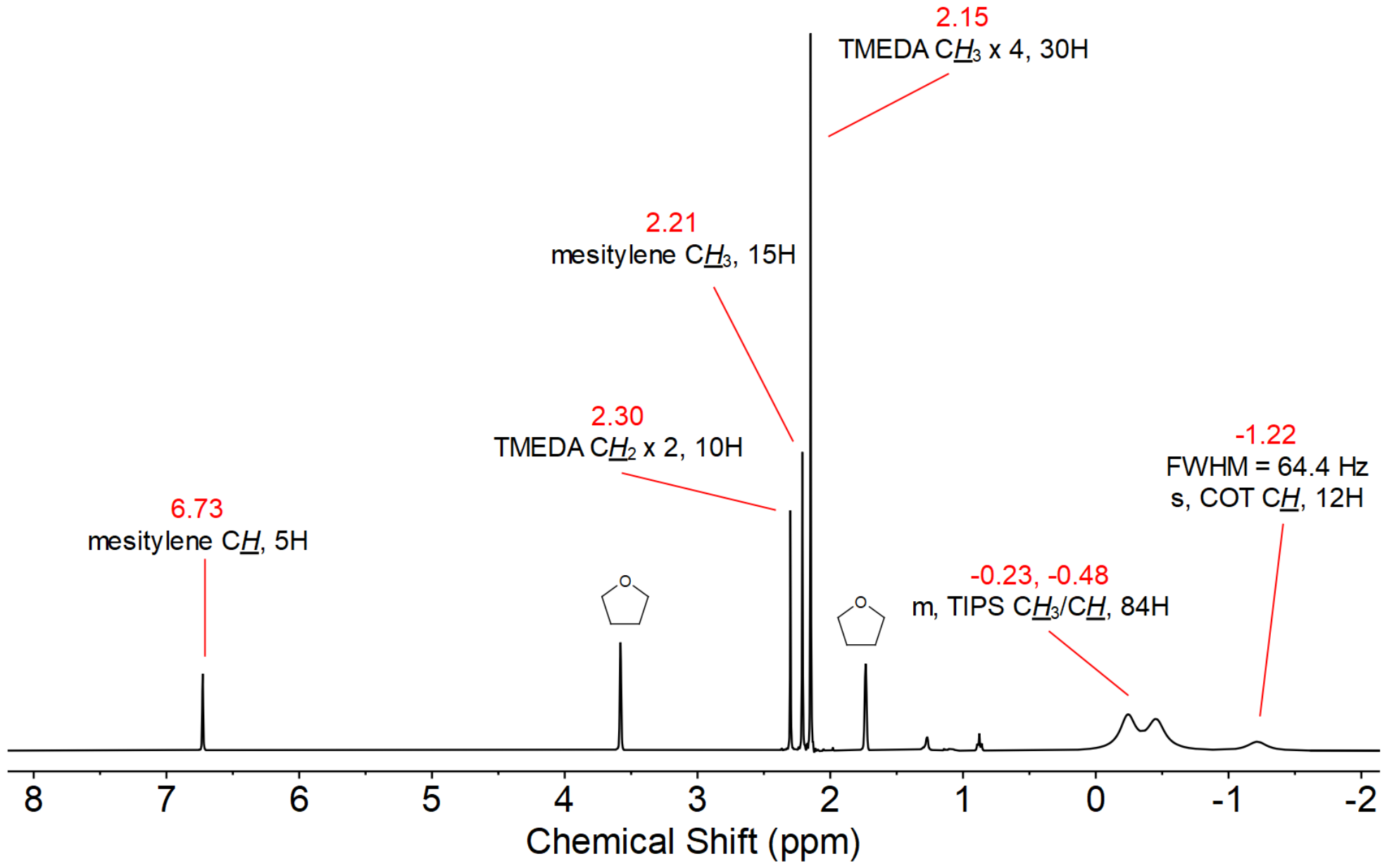

Figure S50. ^{1}H NMR spectrum of **5** in d_8-THF showing from +8 to –2 ppm.

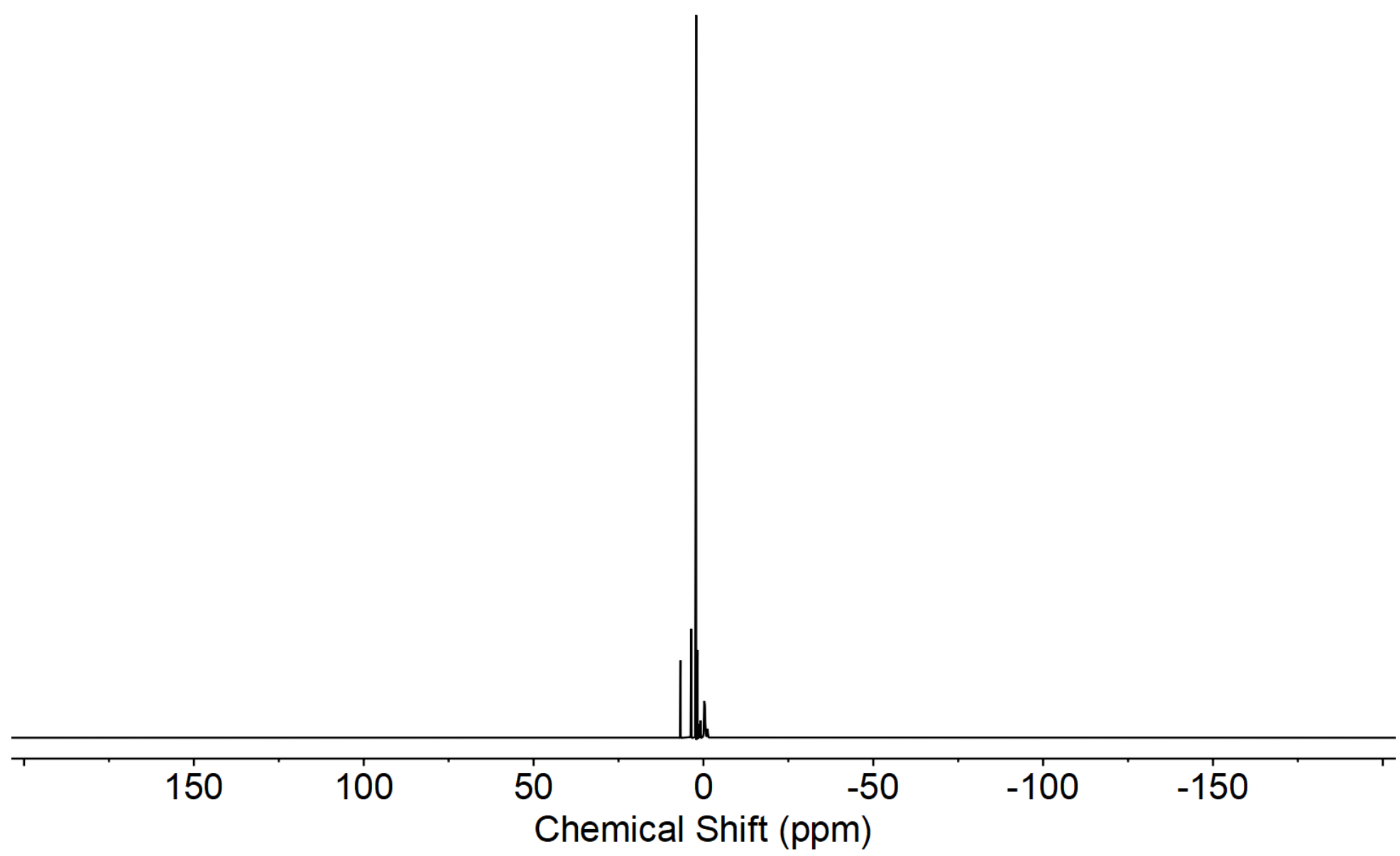

Figure S51. ^{1}H NMR spectrum of **5** in d_8-THF showing from +200 to –200 ppm.

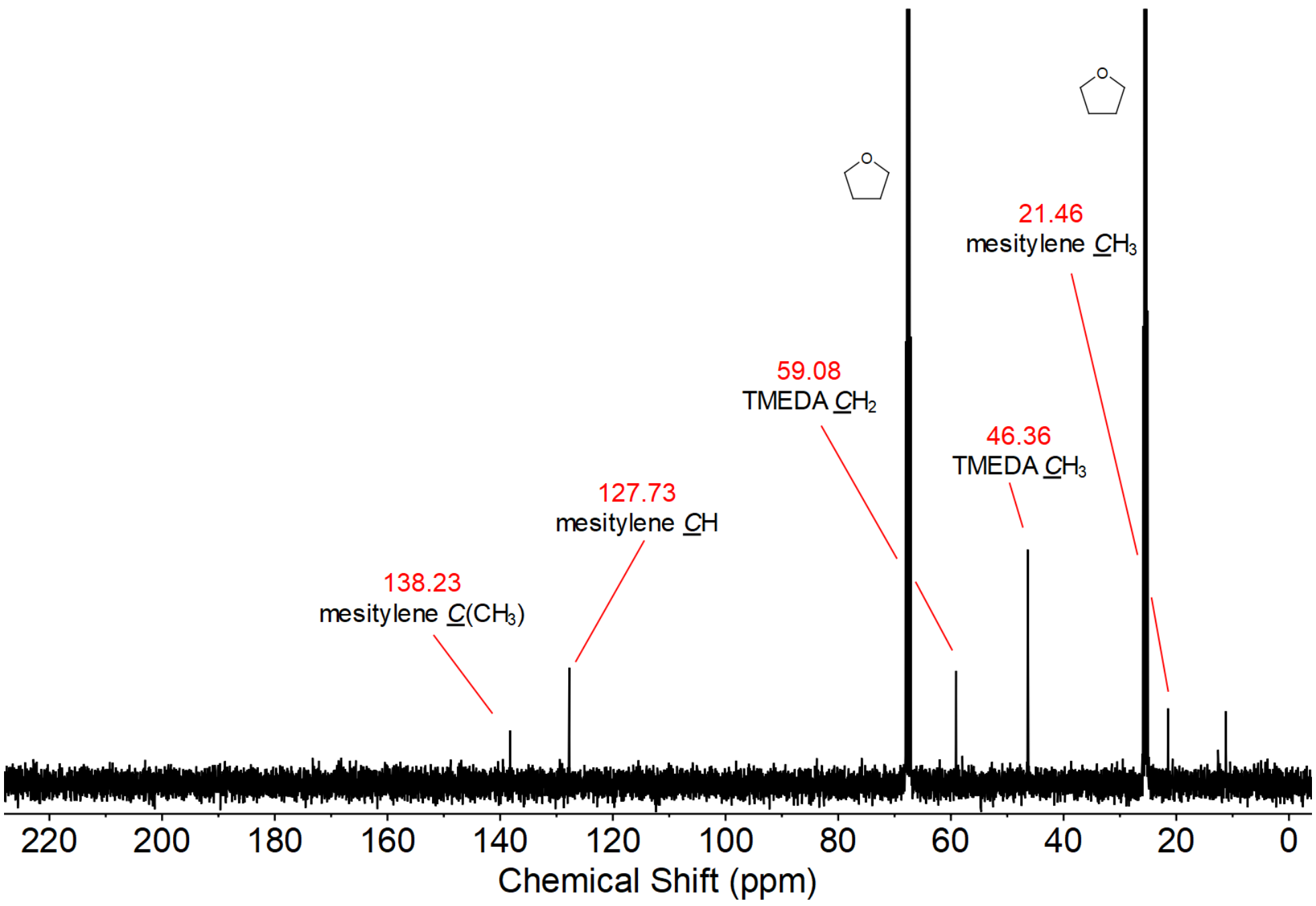

Figure S52. $^{13}C\{^1H\}$ NMR spectrum of **5** in *d*$_8$-THF.

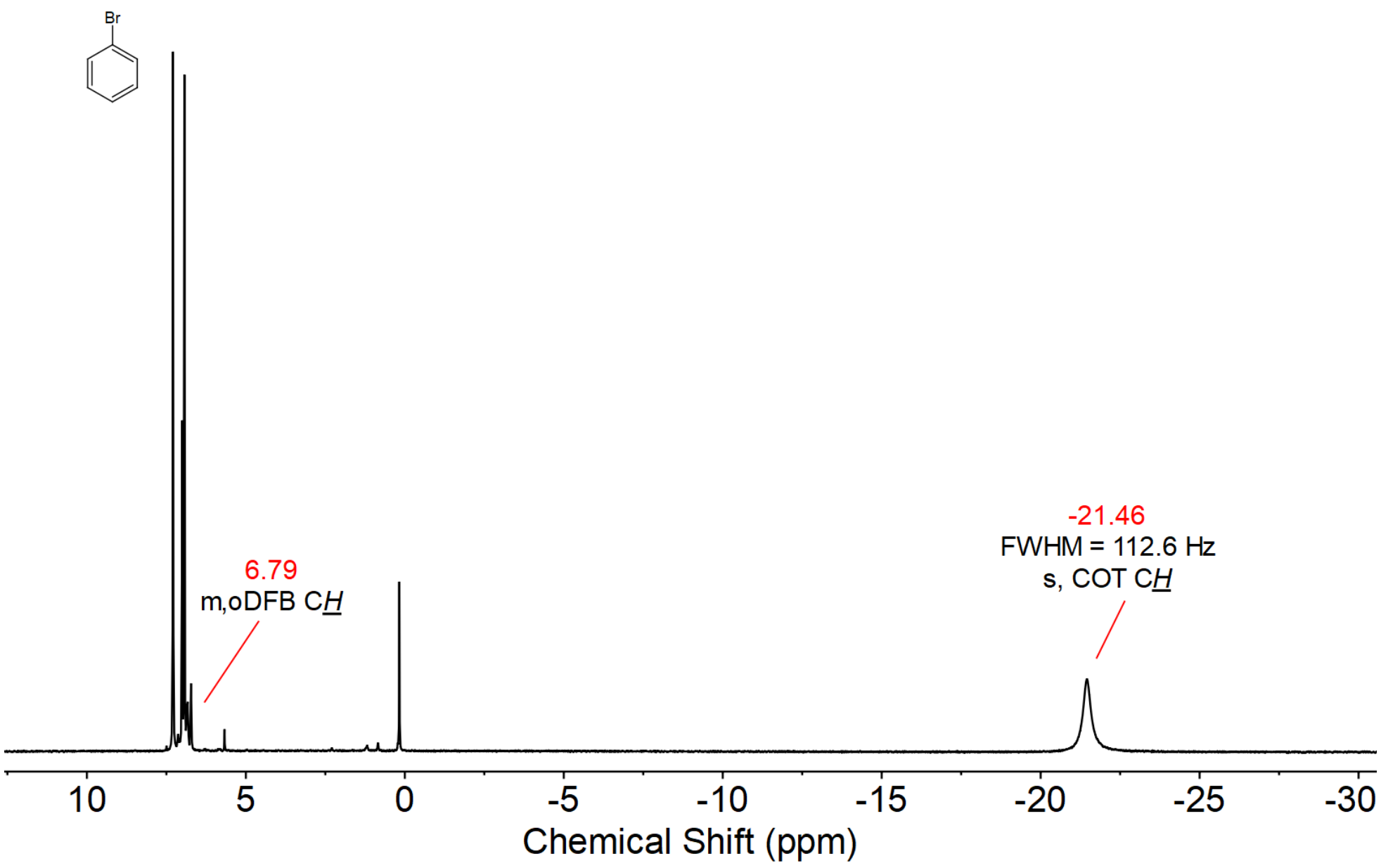

Figure S53. ^1H NMR spectrum of **6** in *d*$_5$-bromobenzene showing from +12 to –5 ppm.

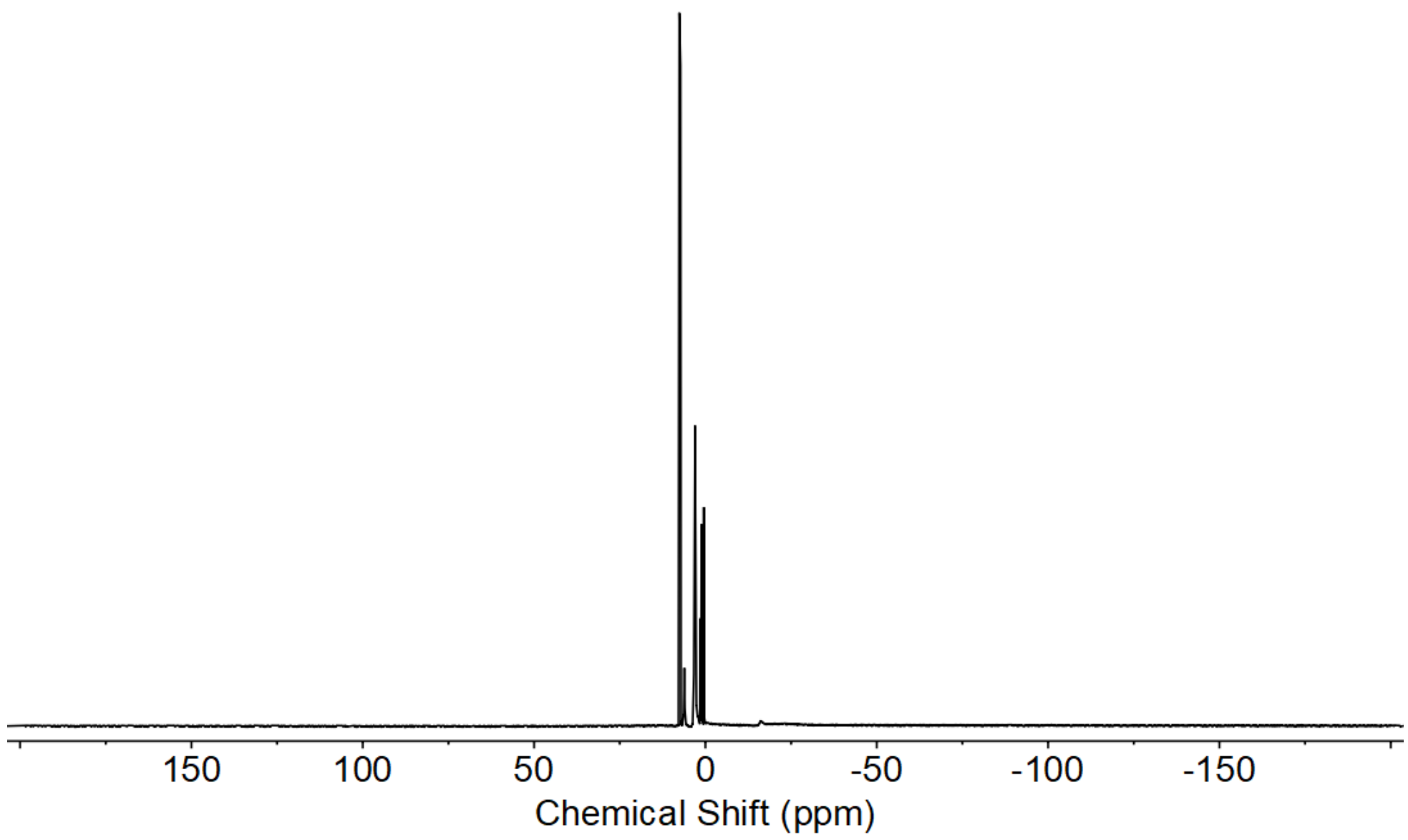

Figure S54. ^{1}H NMR spectrum of **6** in d_5-bromobenzene showing from +200 to –200 ppm.

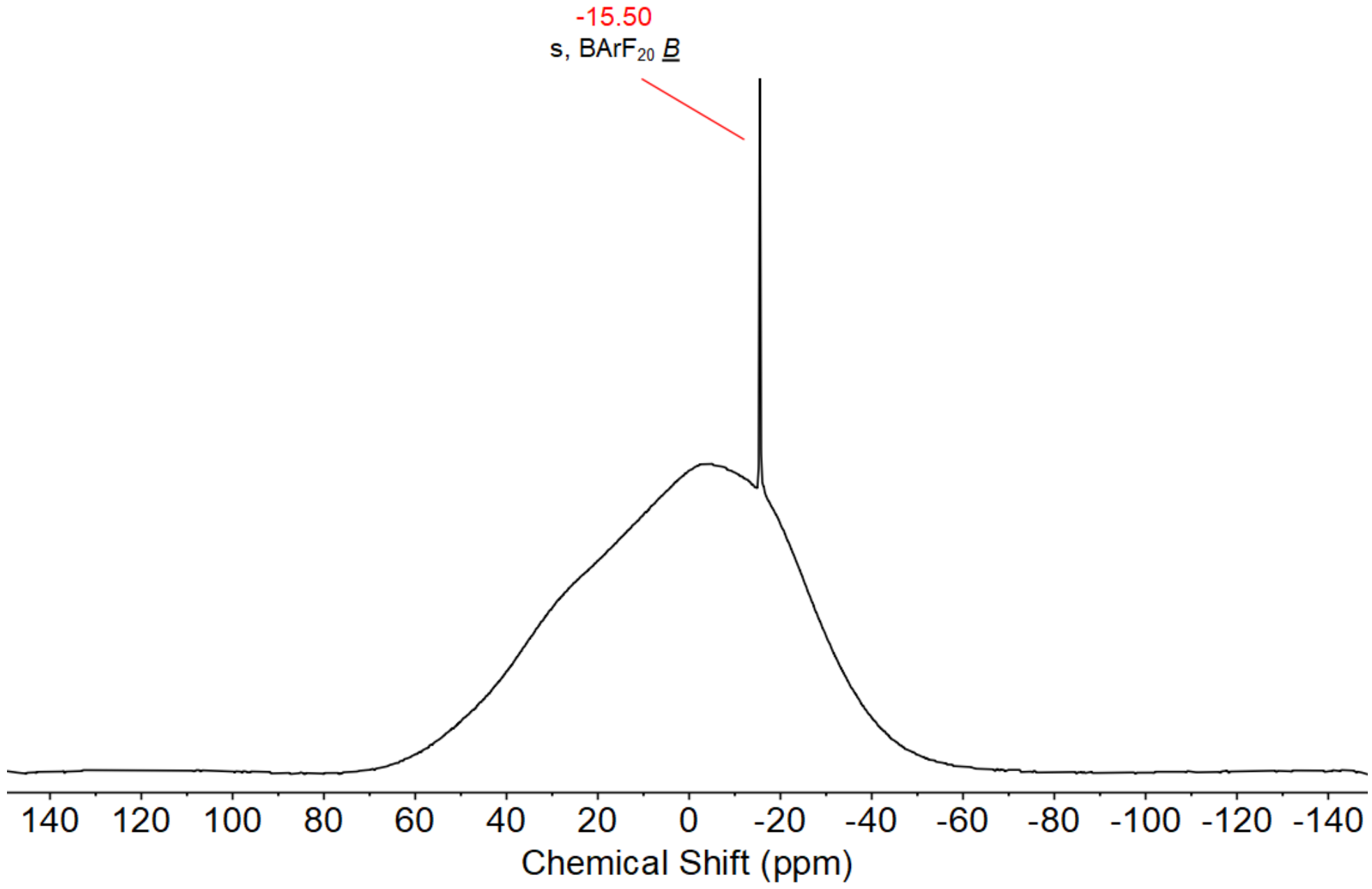

Figure S55. ^{11}B{^{1}H} NMR spectrum of **6** in d_5-bromobenzene.

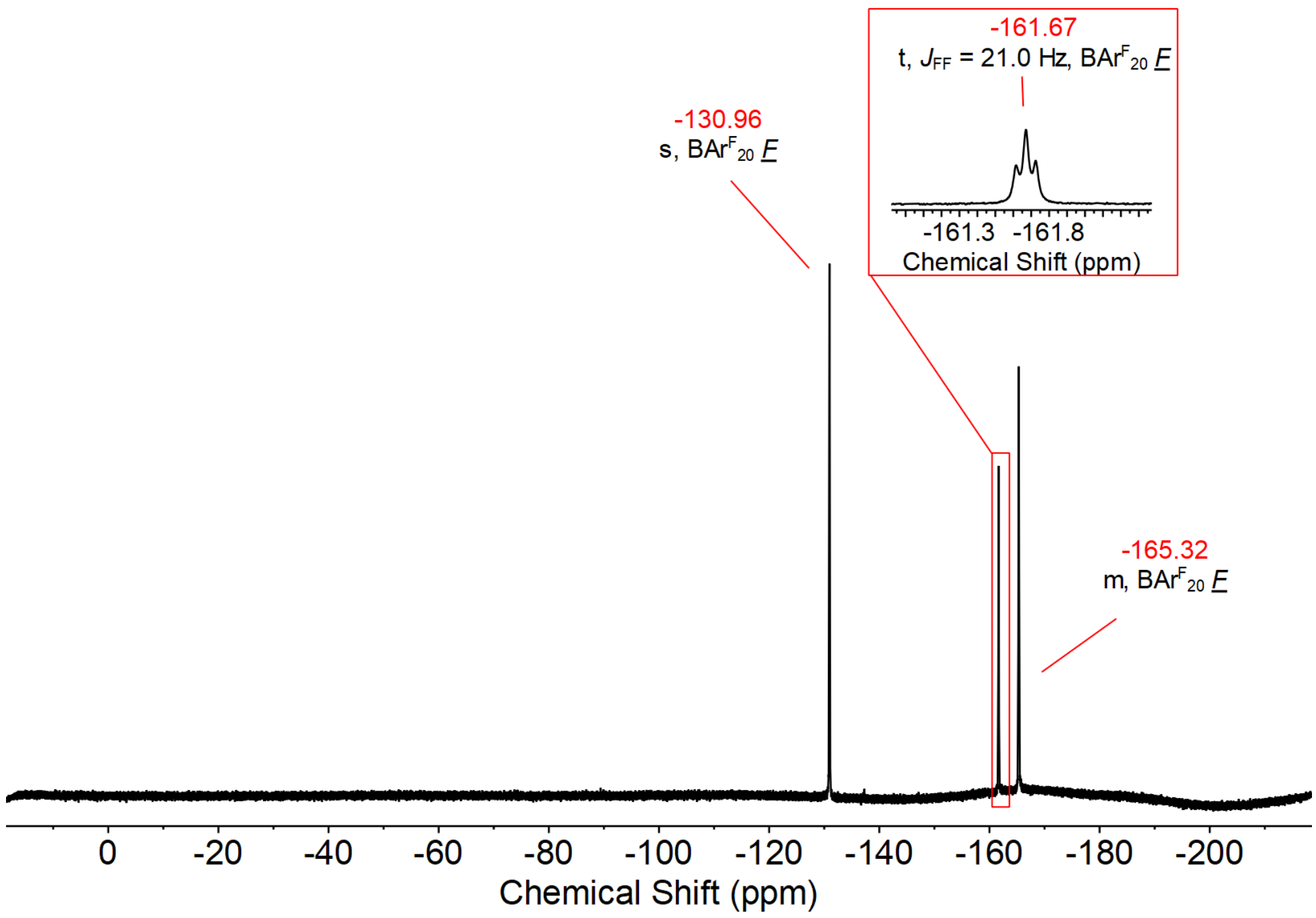

Figure S56. $^{19}F\{^1H\}$ NMR spectrum of **6** in d_5-bromobenzene.

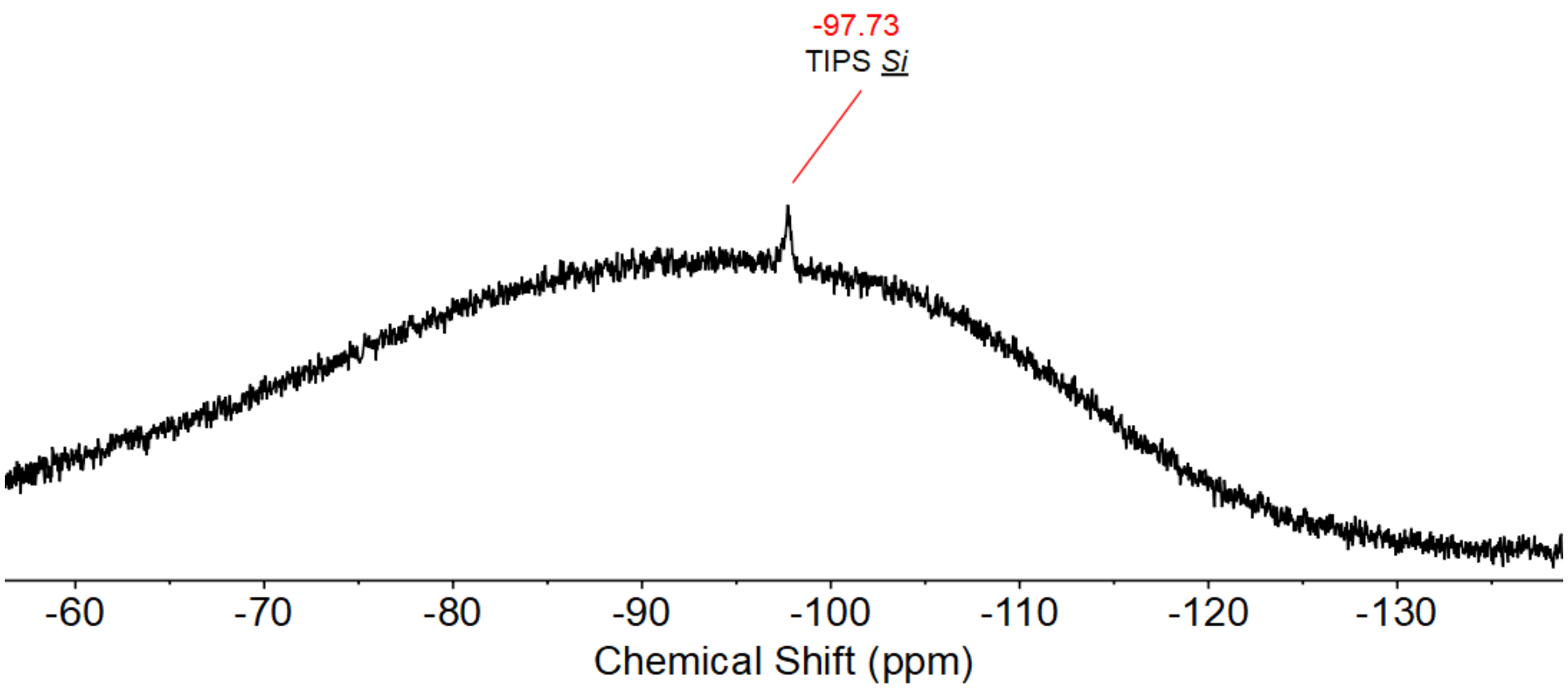

Figure S57. $^{29}Si\{^1H\}$ NMR spectrum of **6** in d_5-bromobenzene.

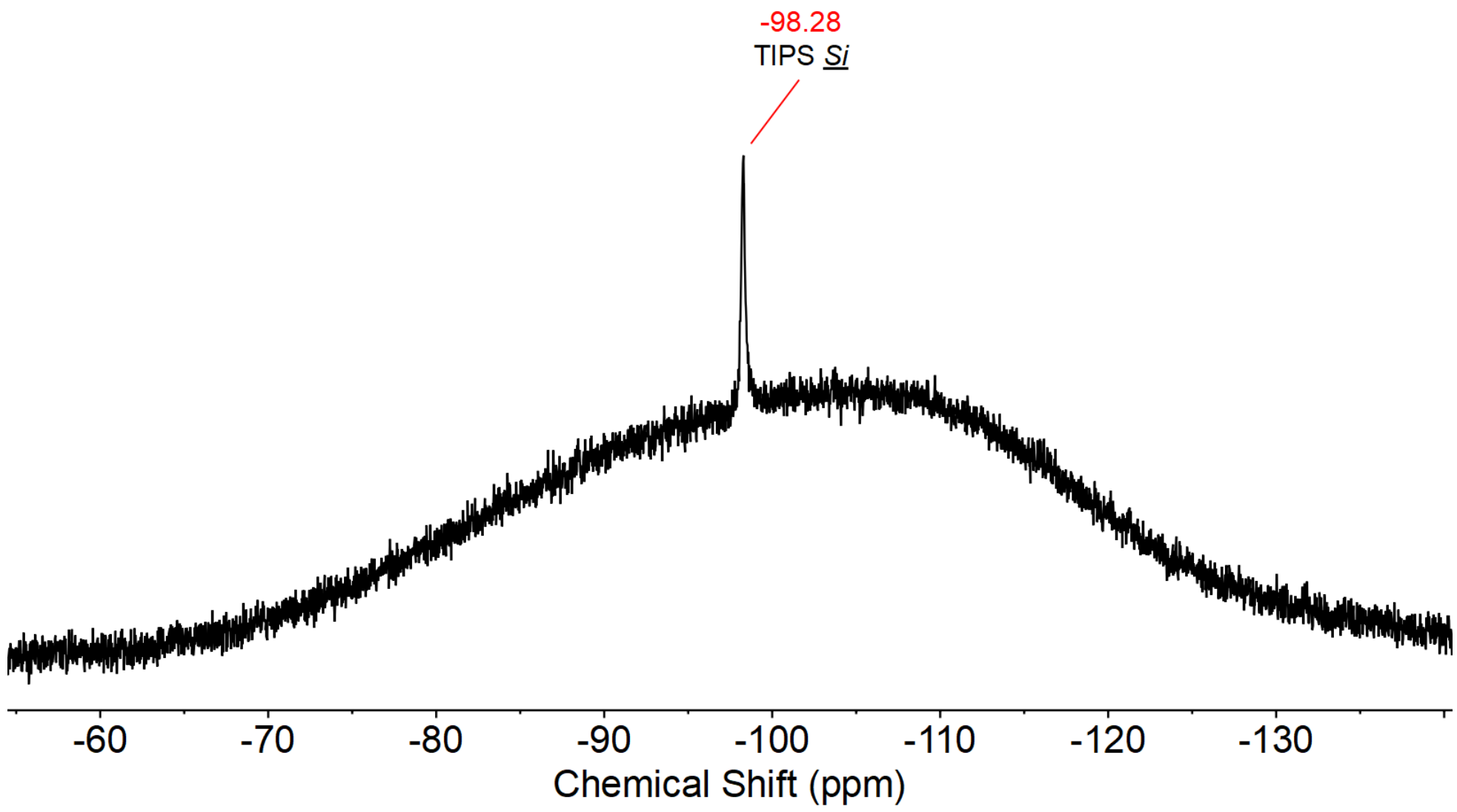

Figure S58. ^{29}Si{^{1}H} NMR spectrum of **6** in *o*DFB.

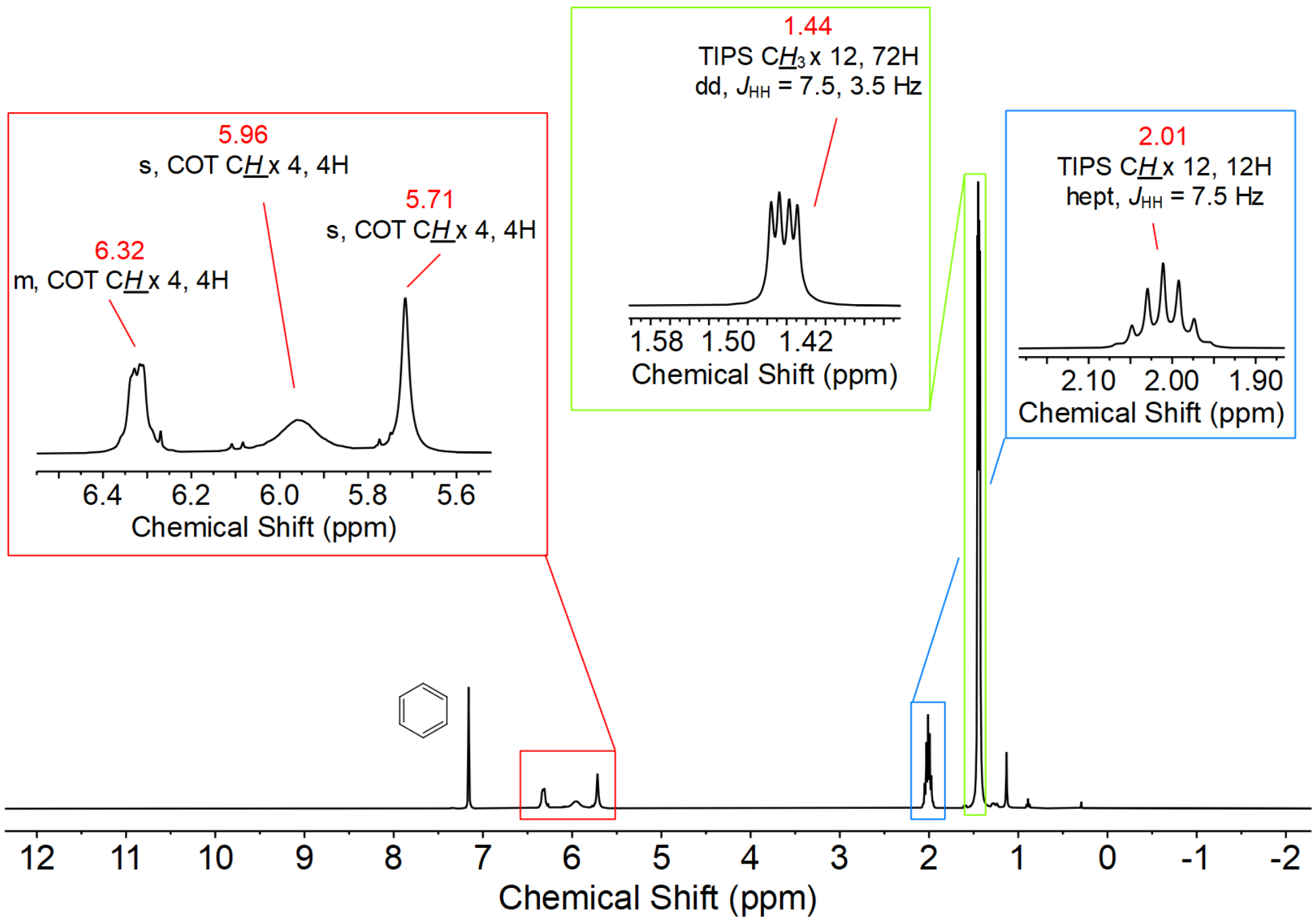

Figure S59. ^{1}H NMR spectrum of **9** in d_6-benzene from +12 to –2 ppm.

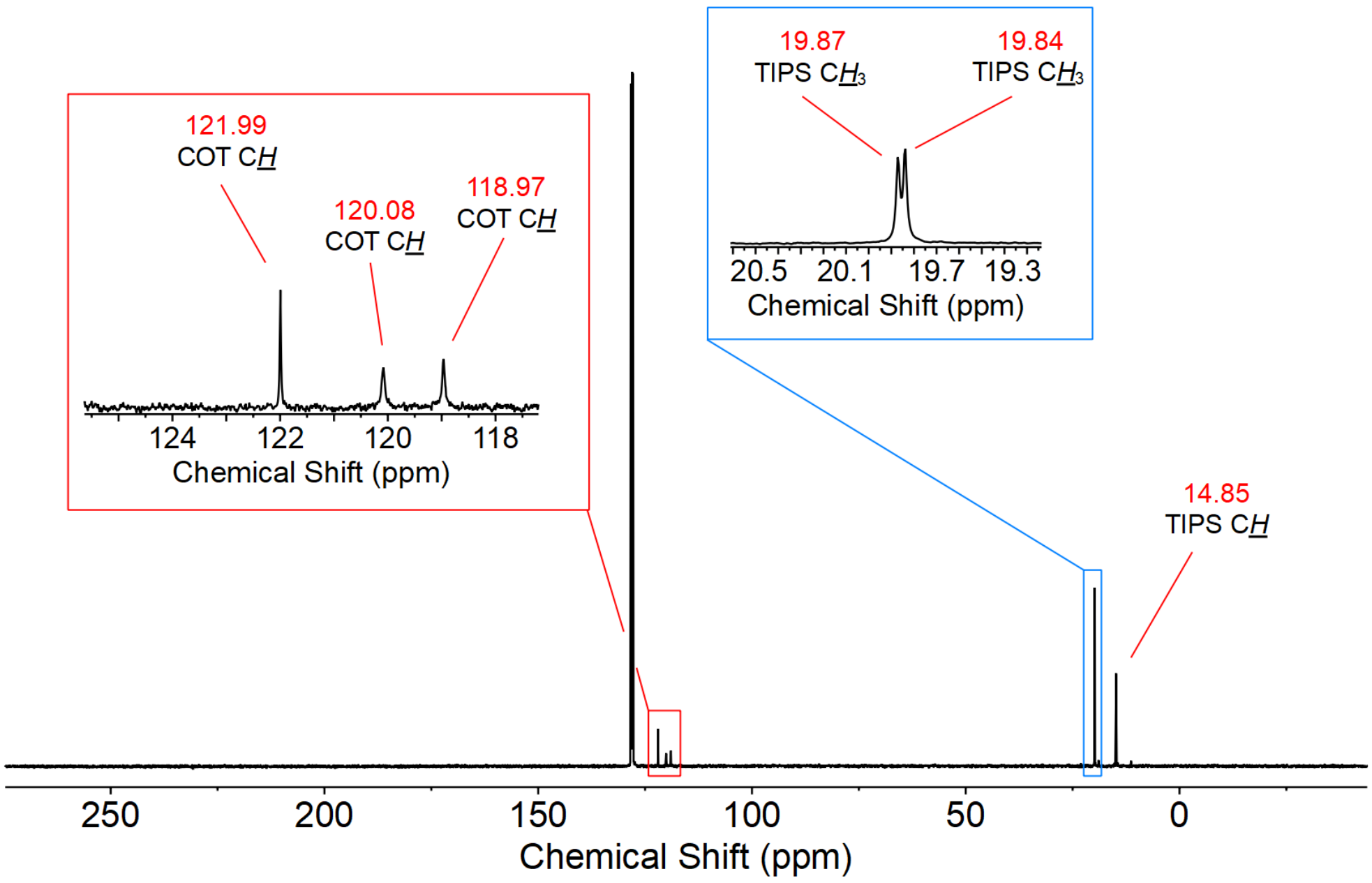

Figure S60. $^{13}C\{^{1}H\}$ NMR spectrum of **9** in d_6-benzene.

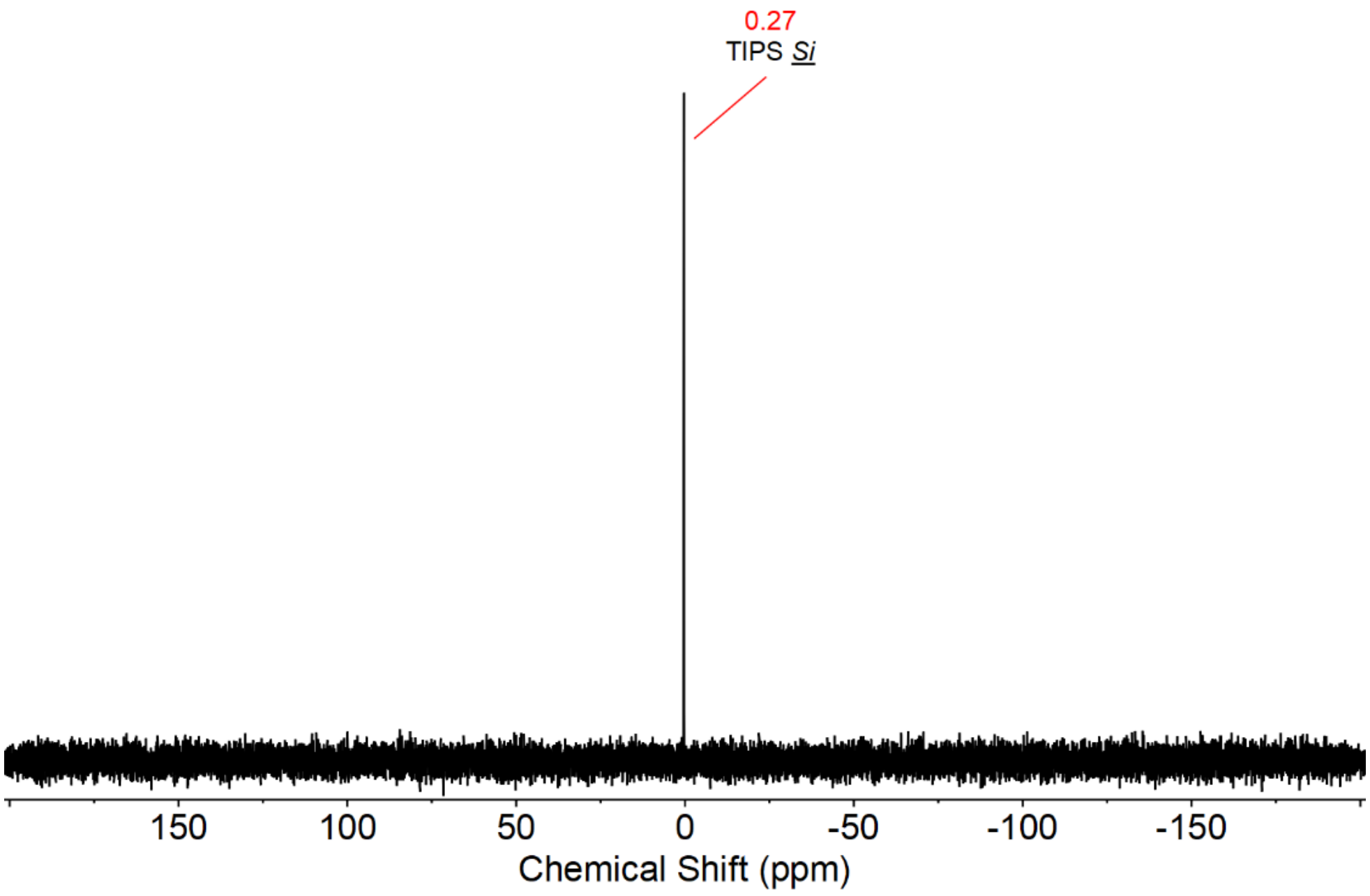

Figure S61. ^{29}Si DEPT90 NMR spectrum of **9** in d_6-benzene.

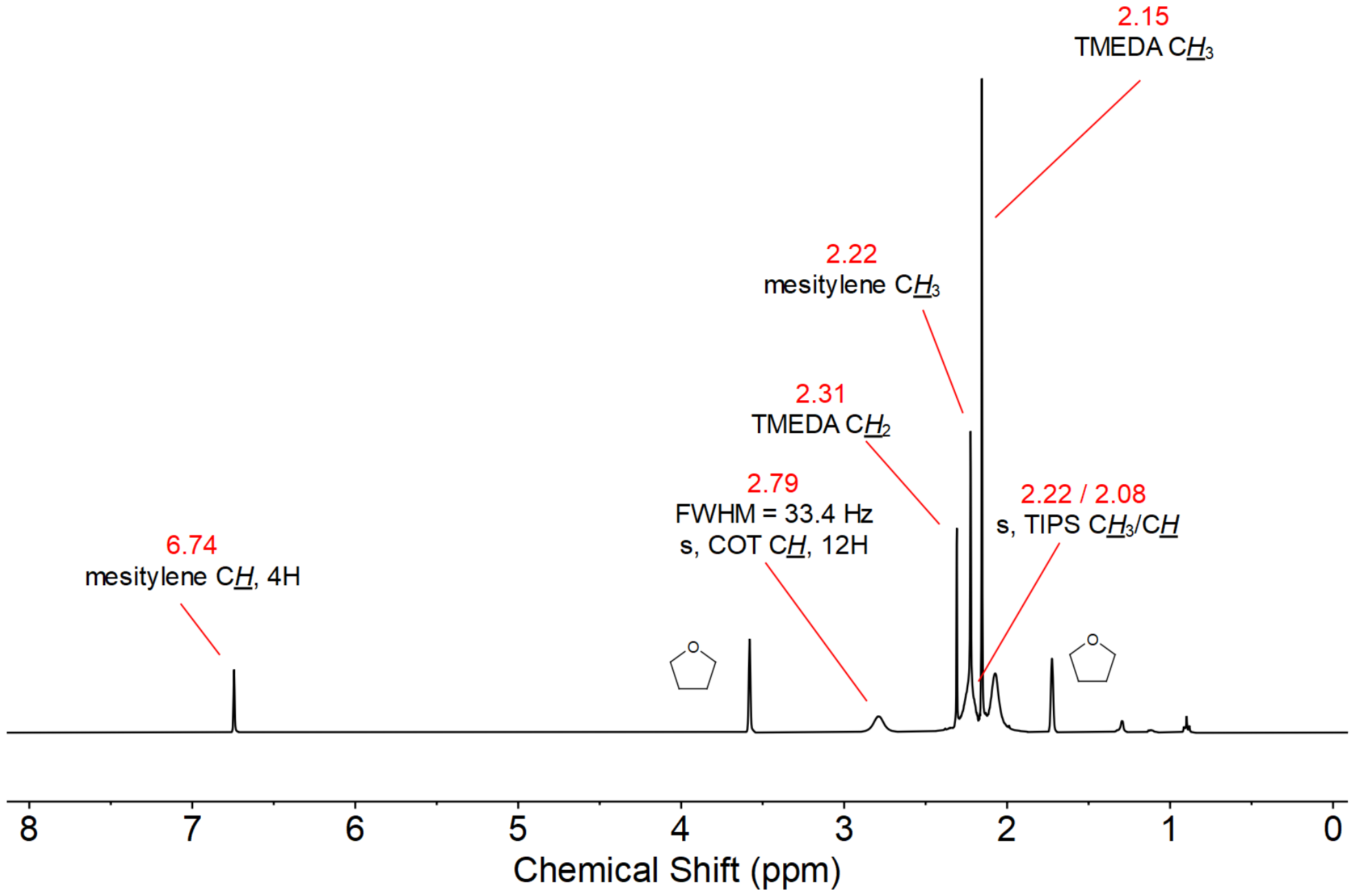

Figure S62. ^{1}H NMR spectrum of **10** in d_8-THF showing from +8 to 0 ppm.

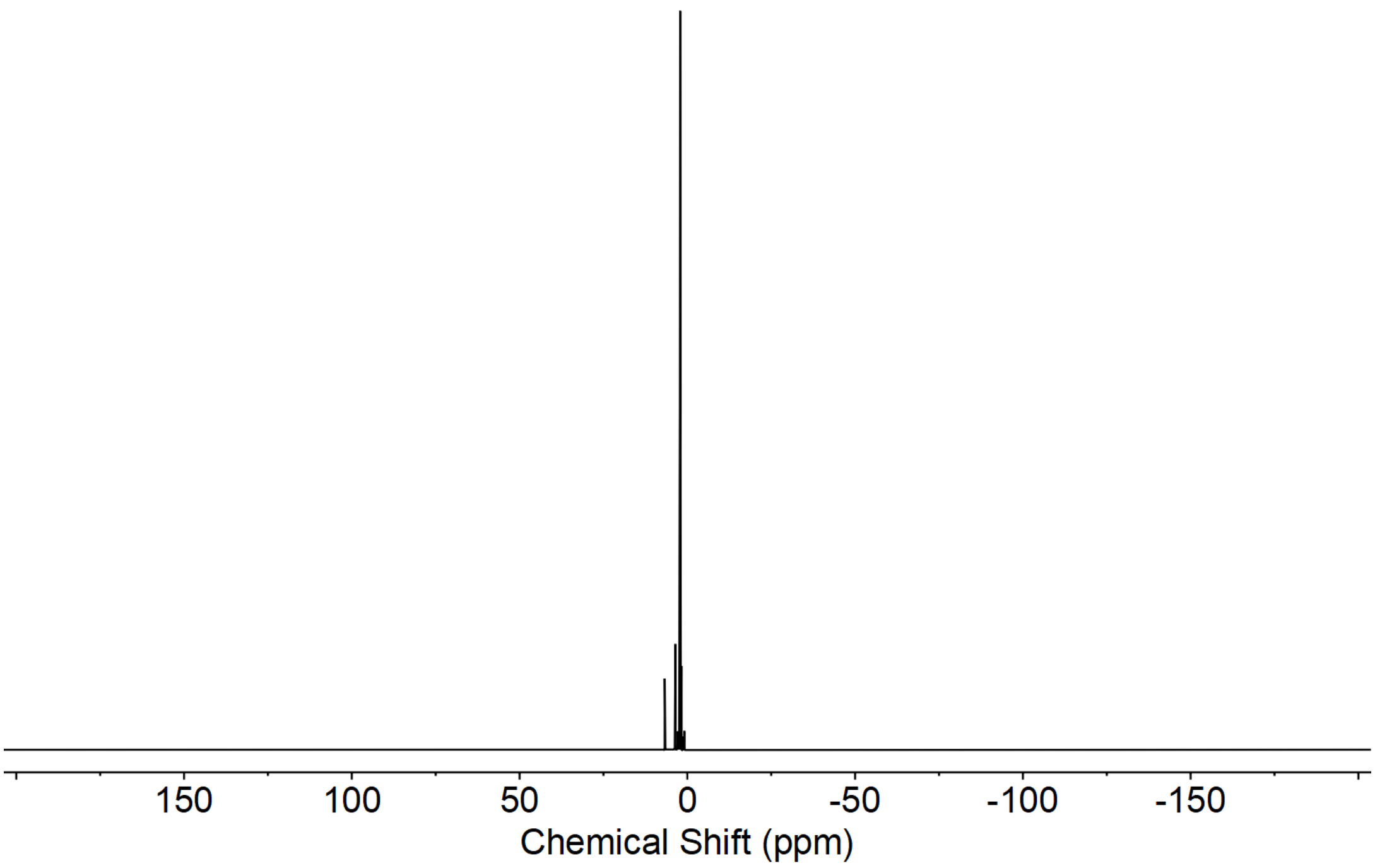

Figure S63. ^{1}H NMR spectrum of **10** in d_8-THF showing from +200 to –200 ppm.

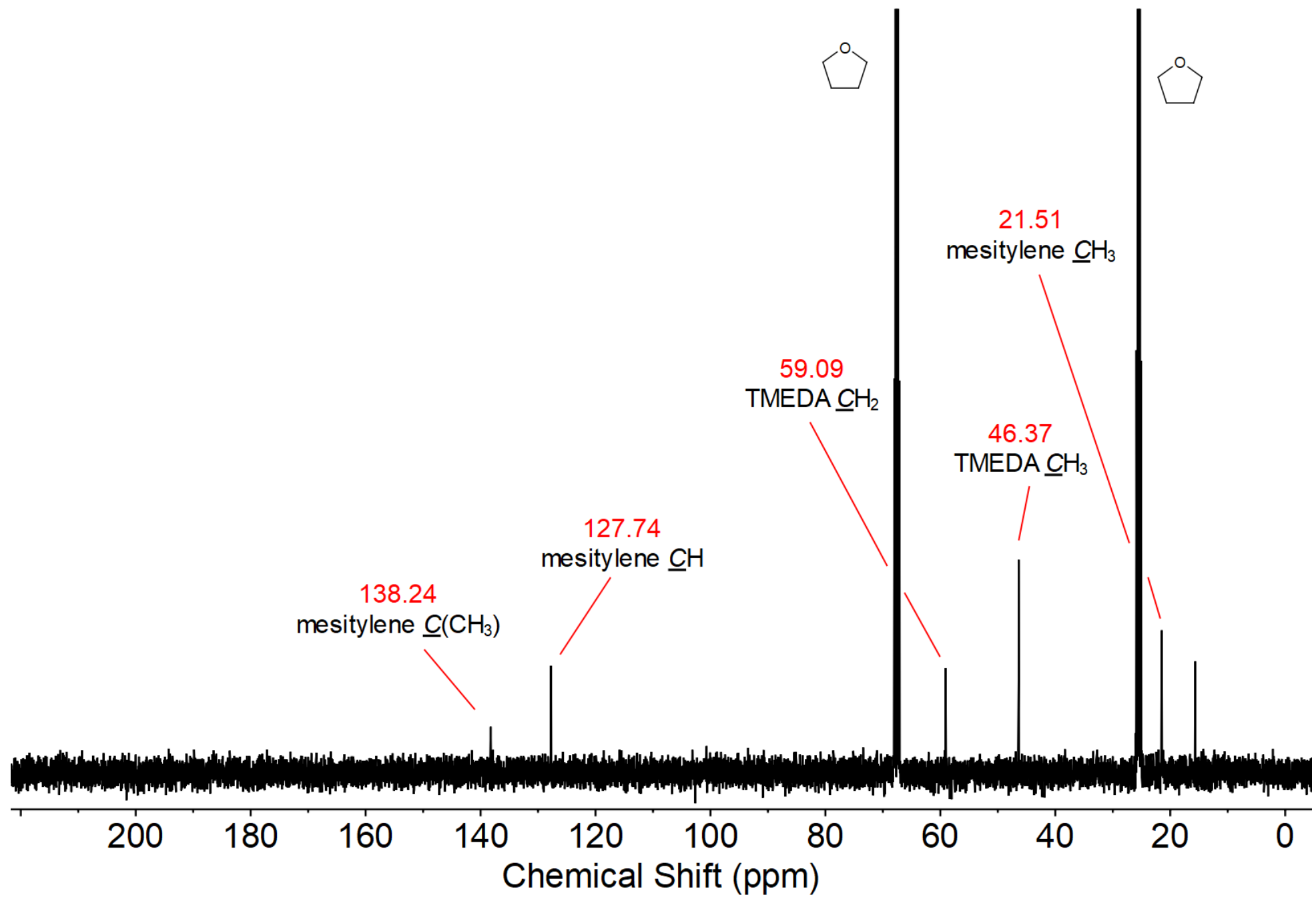

Figure S64. $^{13}C\{^1H\}$ NMR spectrum of **10** in d_8-THF.

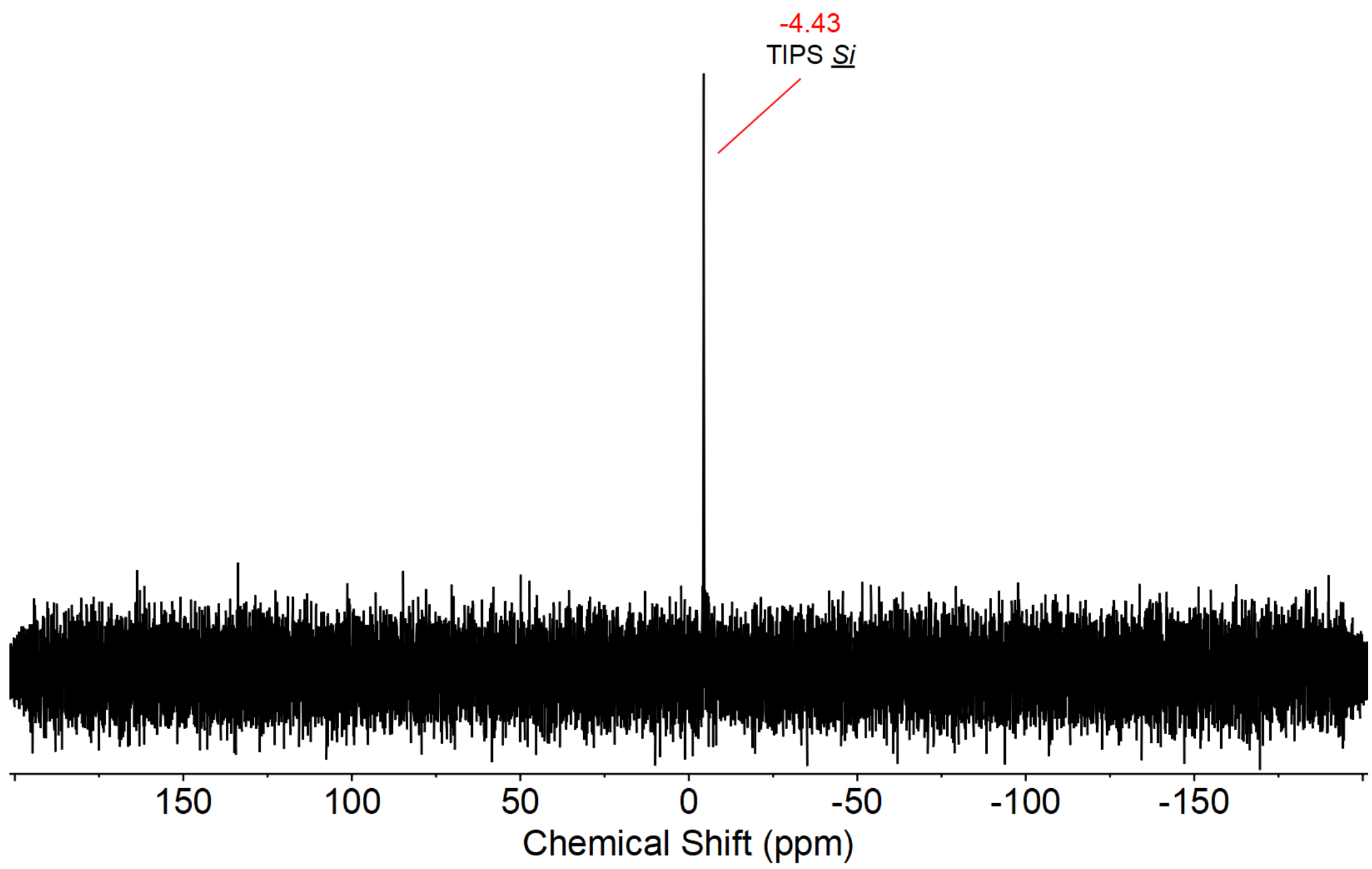

Figure S65. $^{29}Si\{^1H\}$ NMR spectrum of **10** in d_8-THF.

Additional discussion of NMR data

The ^{1}H nuclear magnetic resonance (NMR) spectra of all uranium complexes show paramagnetic broadening and wide chemical shift ranges due to their U(III) $5f^3$ ($^4I_{9/2}$), U(IV) $5f^2$ ($^3H_4$) and U(V) $5f^1$ ($^2F_{5/2}$) centres. In complexes **1**–**3**, the COT ring CH resonances (δ_H = –37.7, **1**; –31.19, **2**; –21.46, **3**) show that both the uranocenate (**2**) and uranocenium (**3**) complexes show a downfield shift relative to neutral **1** [18,19]. In **4**, **5**, and **6**, the ^{1}H resonances of the Si*i*Pr$_3$ groups reveal restricted rotation about the U···COT†† axis.

Magnetic moments determined by NMR spectroscopy (Evans method)

Table S11. Data for the determination of the magnetic moments of complexes **2**–**6**, and **10**.

	μ_{eff} / B.M mol^{-1}	Sample mass / g ^{A}	Solvent + sample mass / g ^{A}	M$_r$ / g mol^{-1}	Δ peak / Hz ^{B}
**2**	3.87(7)	0.0036	0.7716	701.55	65.30
**3** ^{C}	2.07(1)	0.0103	0.9653	1125.31	23.05
**4**	2.77(1)	0.0240	0.6120	1071.69	162.76
**5**	3.16(1)	0.0288	0.6918	1431.12	174.23
**6** ^{C}	2.59(1)	0.0225	0.7245	1750.60	68.79
**10**	2.74(1)	0.0176	0.5096	1333.20	109.69

^{A} The small masses engender large errors in this methodology; the results should be interpreted cautiously along with other data. ^{B} Spectrometer frequency 400.130 MHz. ^{C} Spiked with two drops of toluene to use as the reference peak. Simple diamagnetic correction of M_r / –2,000,000 applied. ρd_6-benzene = 0.950 g mL^{-1}, ρd_6-THF = 0.985 g mL^{-1}, ρ*o*DFB = 1.16 g mL^{-1}, ρtoluene = 0.867 g mL^{-1}.

Small-scale oxidation and reactivity studies

The following section details exploratory reactions which were performed to better understand the redox chemistry of **1**, and the conditions which permit the formation of **3** and **6** and their stability in solution. These reactions were monitored by multinuclear NMR spectroscopy.

In the case of **1** we explored the use of alternative solvents during the oxidation step (fluorobenzene, d_5-bromobenzene); and alternative oxidants and anions ([Fc][B(C_6F_5)$_4$], Ag^{I}[B(C_6F_5)$_4$], Ag^{I}[B{C_6H_3-3,5-(CF_3)$_2$}$_4$], Ag^{I}[SbF_6]). With complexes **3** and **6**, we used anion-exchange reactions with [*n*Bu$_4$N][PF_6] to determine if the uranocenium cations were stable in the presence of the [PF_6] anion. The reactivity between **6** and [*n*Bu$_4$N][SbF_6] was also examined due to the greater fluorophillicity of Sb(V) compared to P(V). Lastly, the reaction between **3** both [Fc] and [Fc*] was examined.

*Oxidation of **1** with [Ph_3C][$B(C_6F_5)_4$] in fluorobenzene*

Fluorobenzene was added to a mixture of **1** (20 mg) and [Ph_3C][$B(C_6F_5)_4$] (41 mg) in a J. Youngs appended NMR tube, resulting in an immediate colour change to dark brown, and the ^1H NMR spectrum (below) showed conversion to **3**. The resonances corresponding to Gomberg's dimer, which is a byproduct of the oxidation, are obscured by the fluorobenzene resonance.

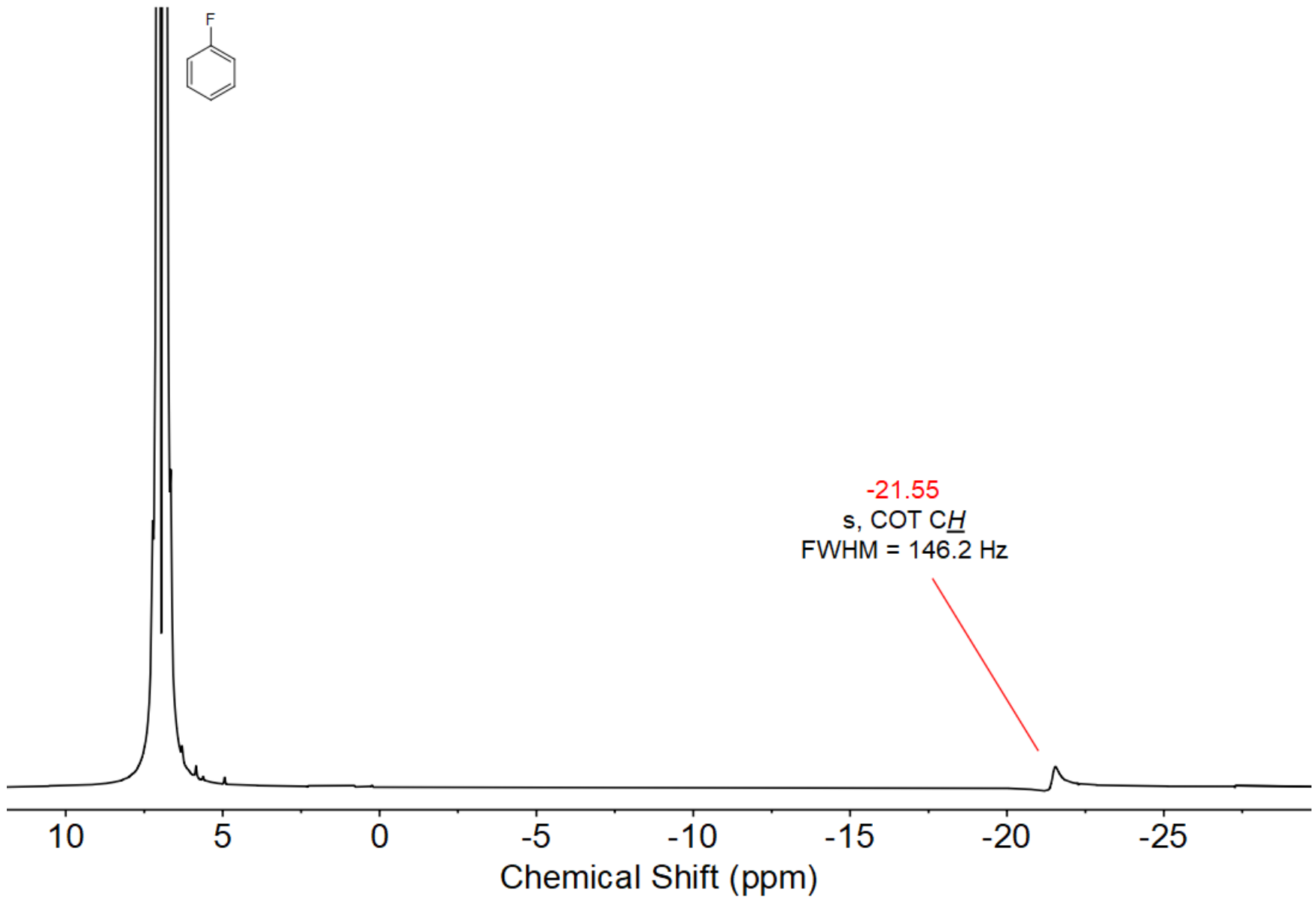

Figure S66. ^1H NMR spectrum of the crude reaction mixture between **1** and [Ph_3C][$B(C_6F_5)_4$] in fluorobenzene showing from +12 to –30 ppm.

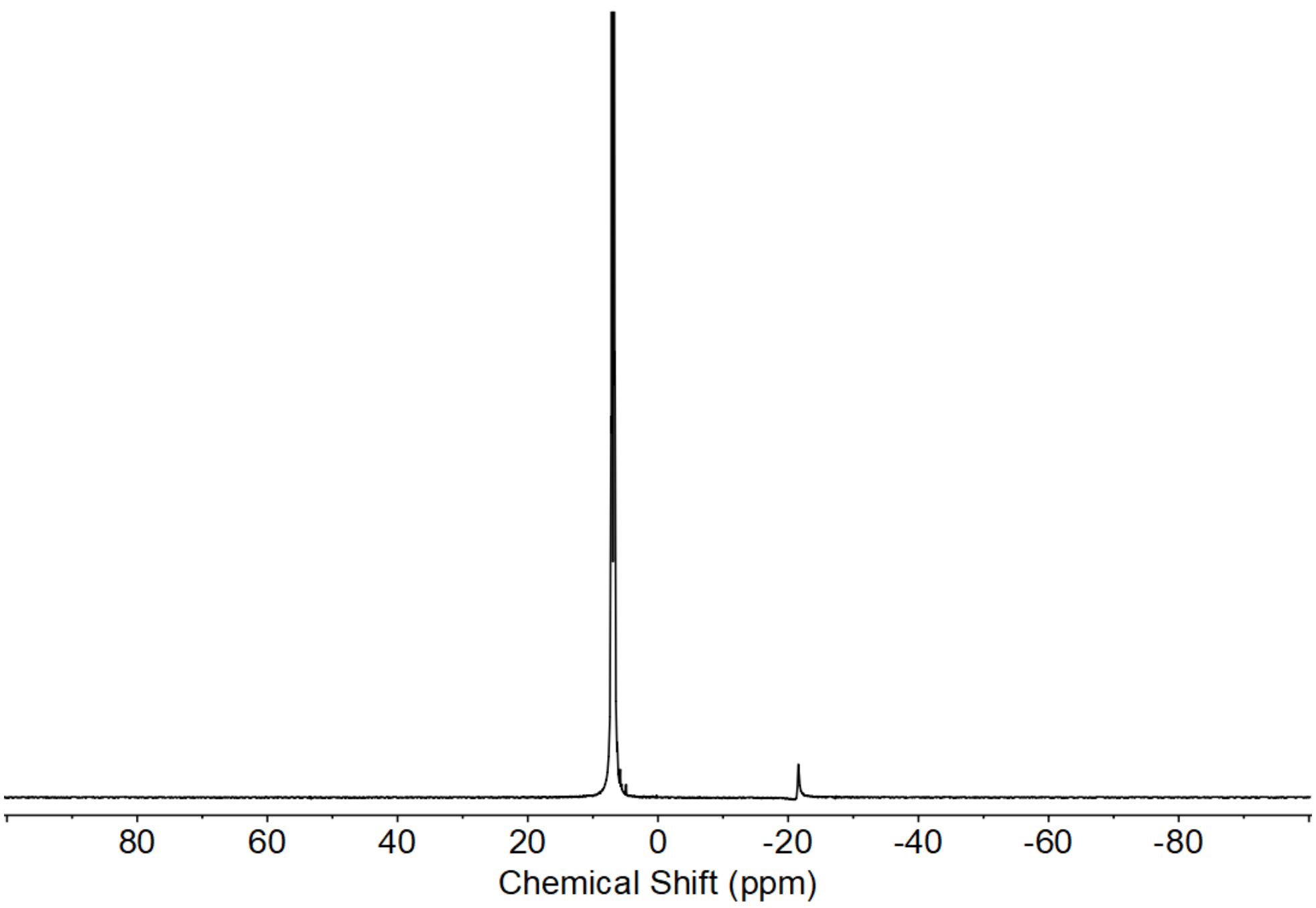

Figure S67. ^1H NMR spectrum of the crude reaction mixture between **1** and [Ph_3C][$B(C_6F_5)_4$] in fluorobenzene showing from +100 to –100 ppm.

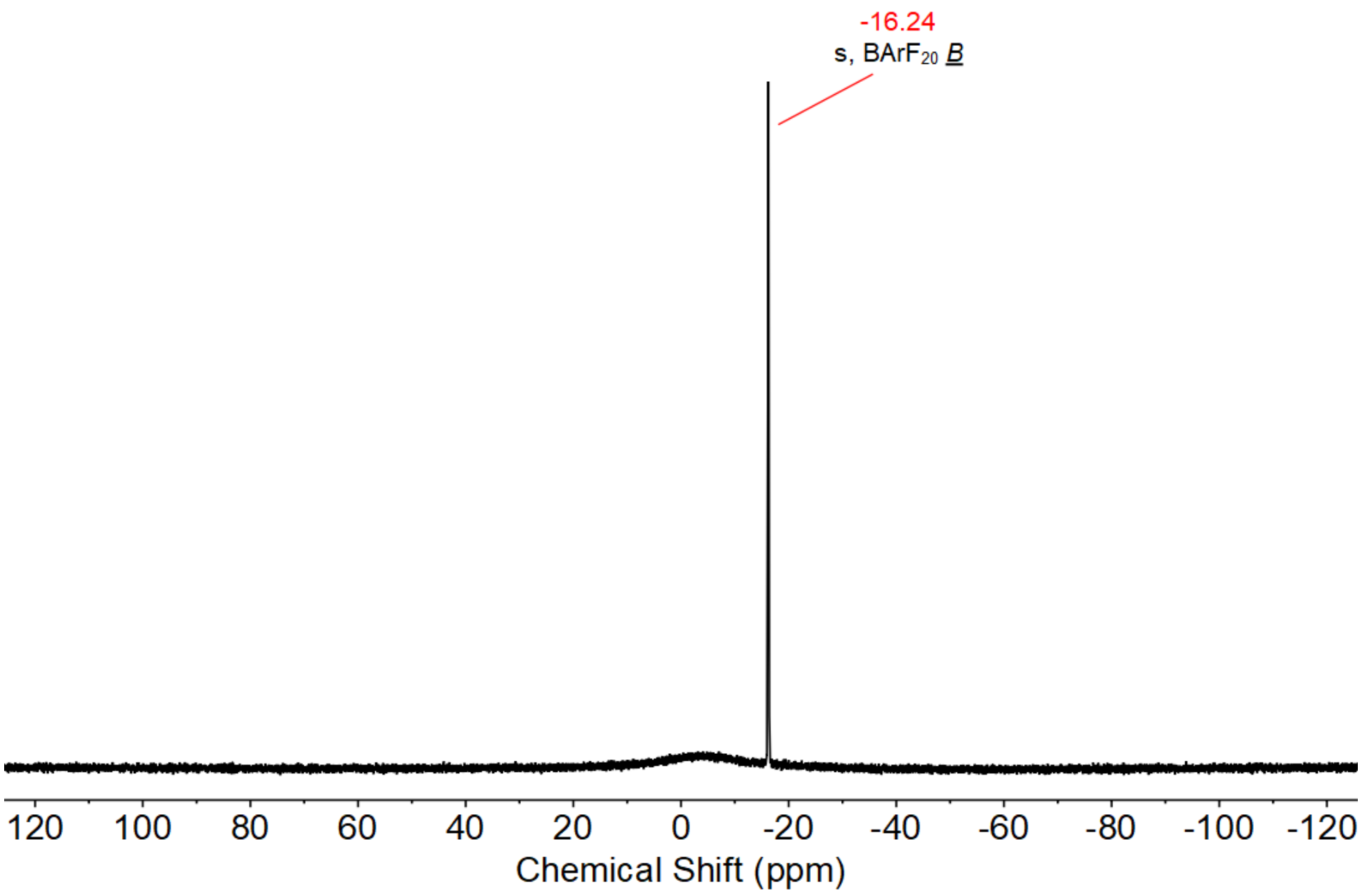

Figure S68. $^{11}B\{^{1}H\}$ NMR spectrum of the crude reaction mixture between **1** and $[Ph_3C][B(C_6F_5)_4]$ in fluorobenzene.

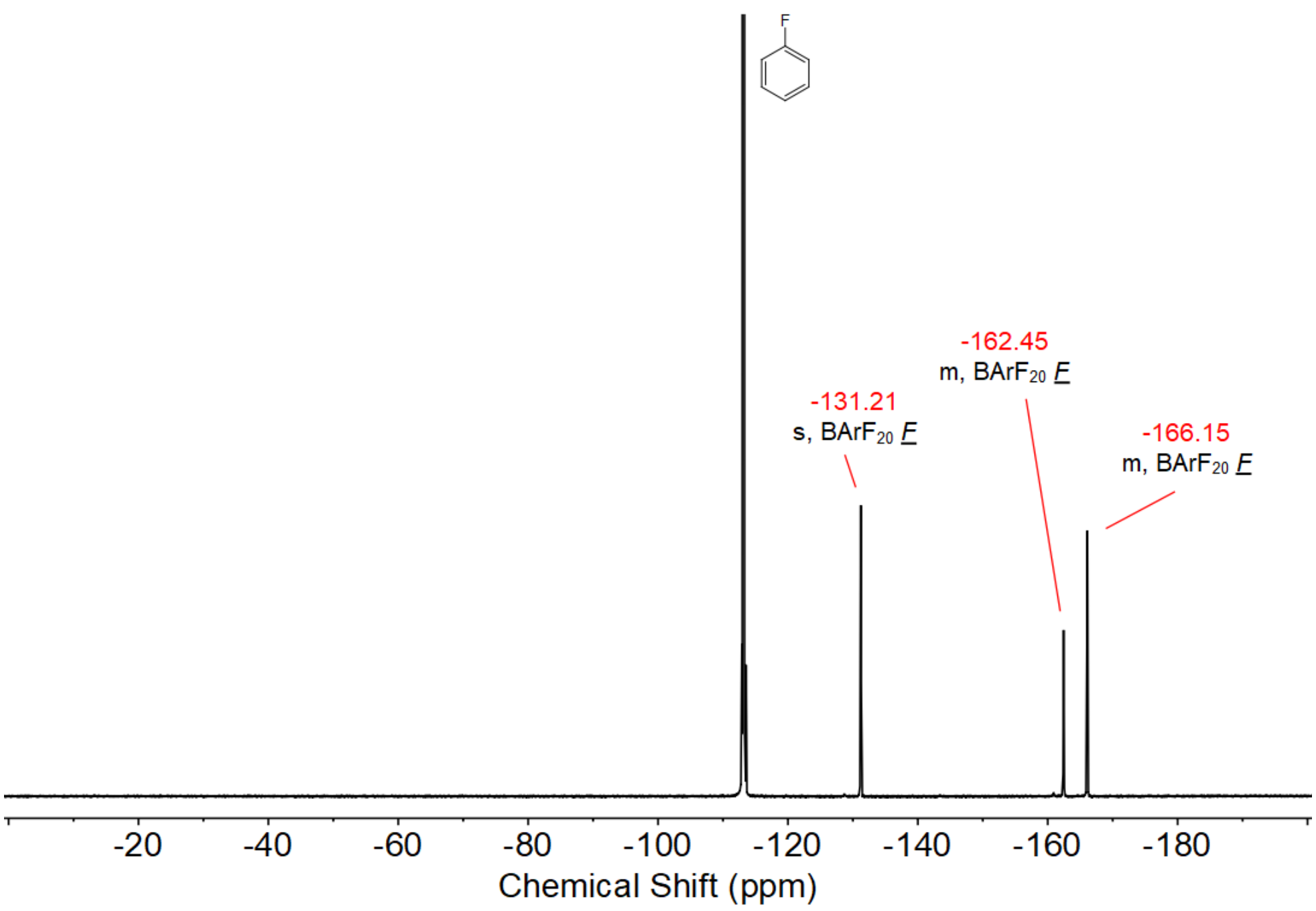

Figure S69. $^{19}F\{^{1}H\}$ NMR spectrum of the crude reaction mixture between **1** and $[Ph_3C][B(C_6F_5)_4]$ in fluorobenzene.

*Oxidation of **1** with [Ph_3C][$B(C_6F_5)_4$] in d_5-bromobenzene*

d_5-bromobenzene was added to a mixture of **1** (20 mg) and [Ph_3C][$B(C_6F_5)_4$] (41 mg) in a J. Youngs appended NMR tube which resulted in an immediate colour change to dark brown. The ^1H NMR spectrum (below) showed the formation of **3** and Gomberg's dimer.

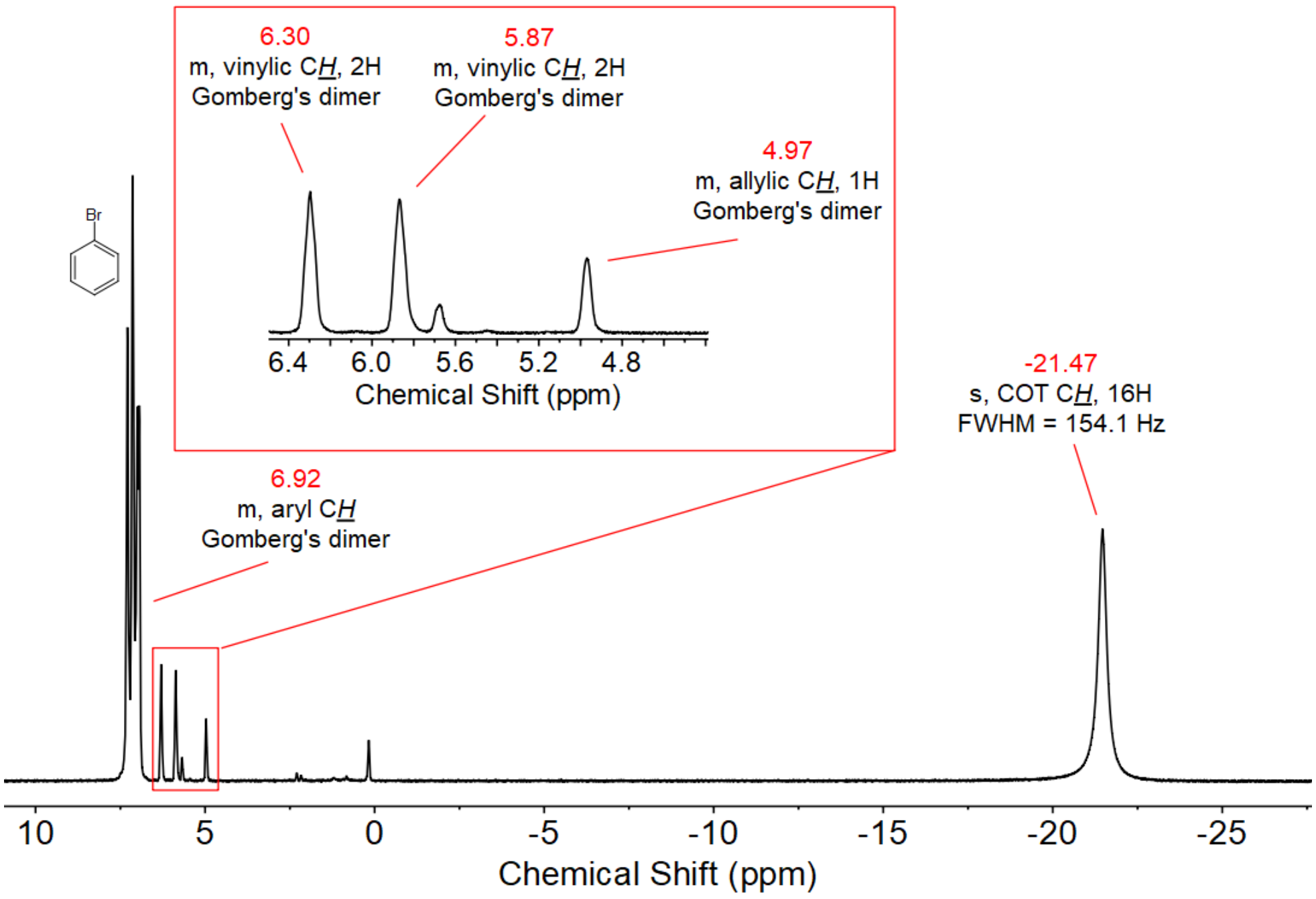

Figure S70. ^1H NMR spectrum of the crude reaction mixture between **1** and [Ph_3C][$B(C_6F_5)_4$] in d_5-bromobenzene showing from +10 to –27 ppm.

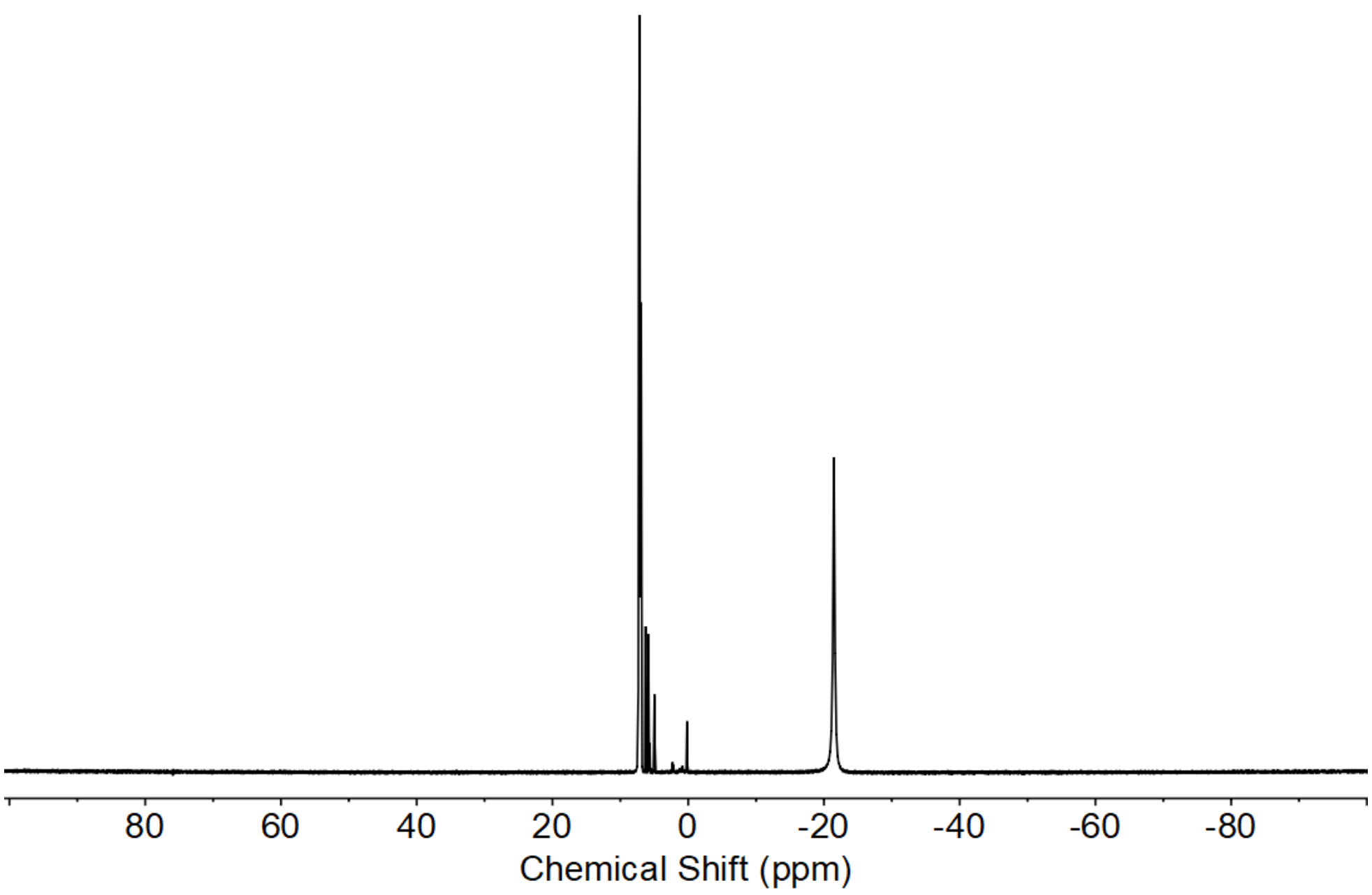

Figure S71. ^1H NMR spectrum of the crude reaction mixture between **1** and [Ph_3C][$B(C_6F_5)_4$] in d_5-bromobenzene showing from +100 to –100 ppm.

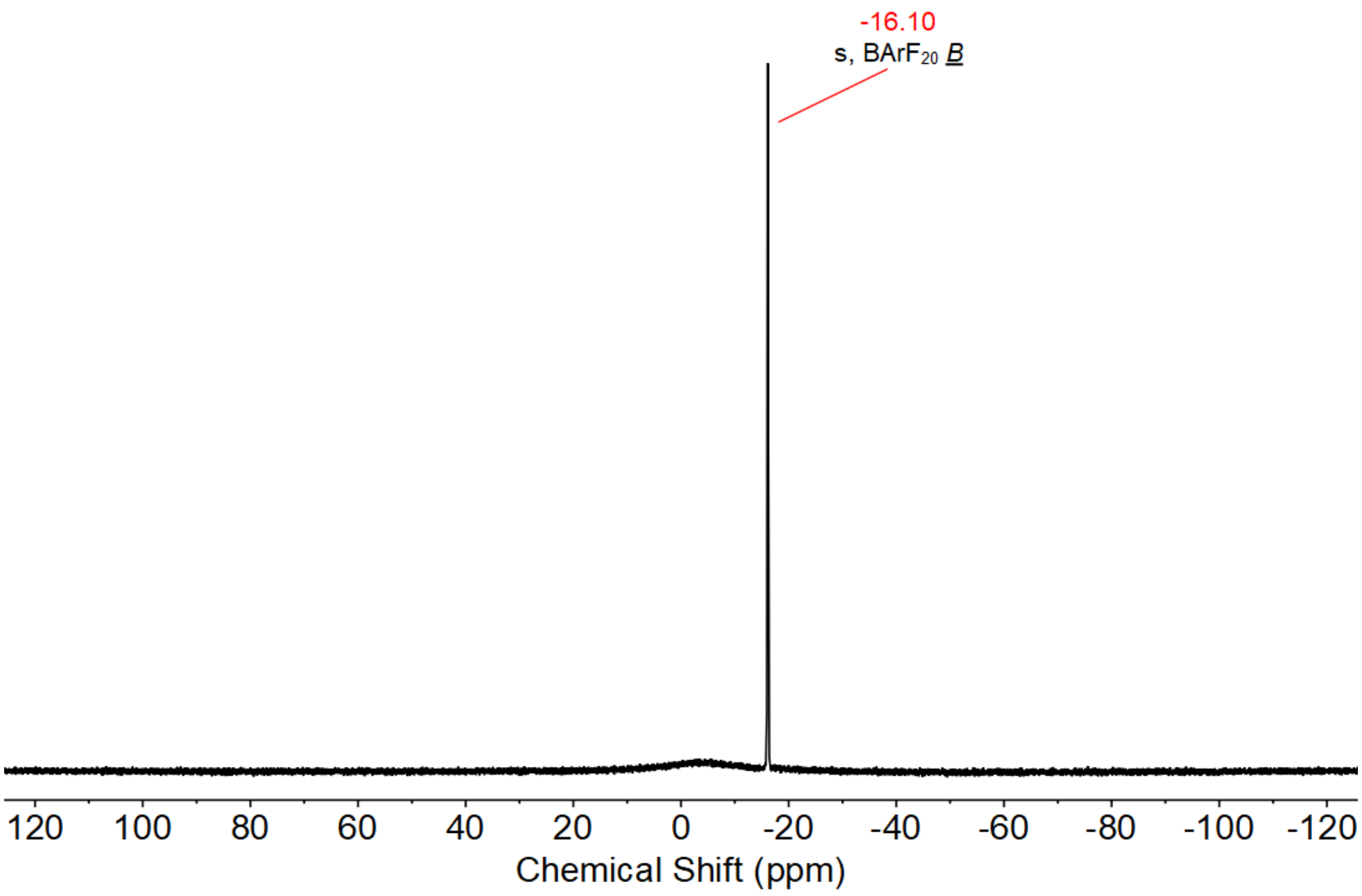

Figure S72. $^{11}B\{^{1}H\}$ NMR spectrum of the crude reaction mixture between **1** and $[Ph_3C][B(C_6F_5)_4]$ in *d*$_5$-bromobenzene.

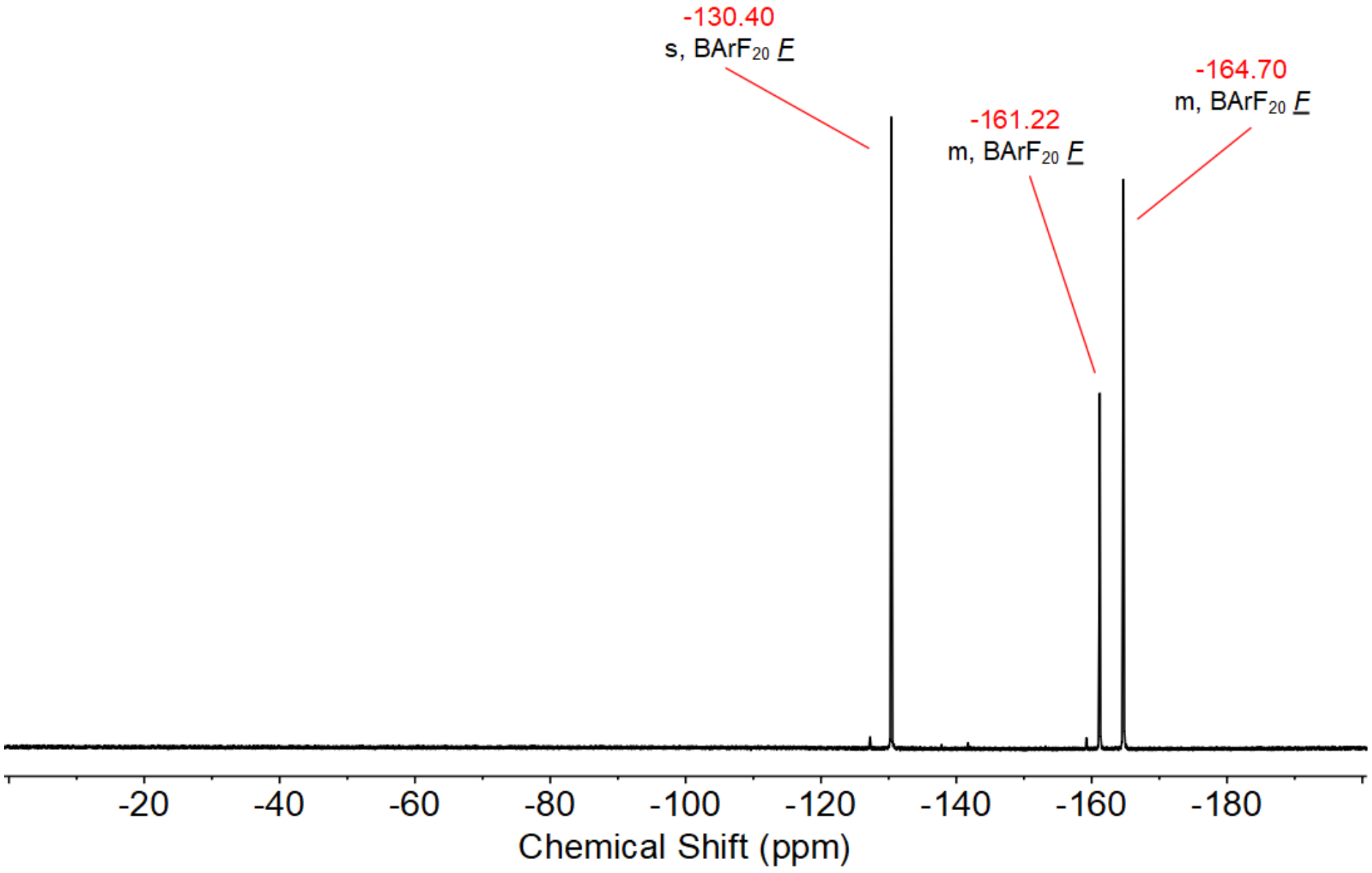

Figure S73. $^{19}F\{^{1}H\}$ NMR spectrum of the crude reaction mixture between **1** and $[Ph_3C][B(C_6F_5)_4]$ in *d*$_5$-bromobenzene.

*Oxidation of **1** with [Fc][B(C_6F_5)$_4$] in oDFB*

*o*DFB was added to a mixture of **1** (20 mg) and [Fc][B(C_6F_5)$_4$] (39 mg) in a J. Youngs appended NMR tube which resulted in an immediate colour change to dark brown, yielding **3**. Inspection of the ^{1}H NMR spectrum (below) showed resonances corresponding to **3**, while those for [Fc] are obscured by the *o*DFB resonances.

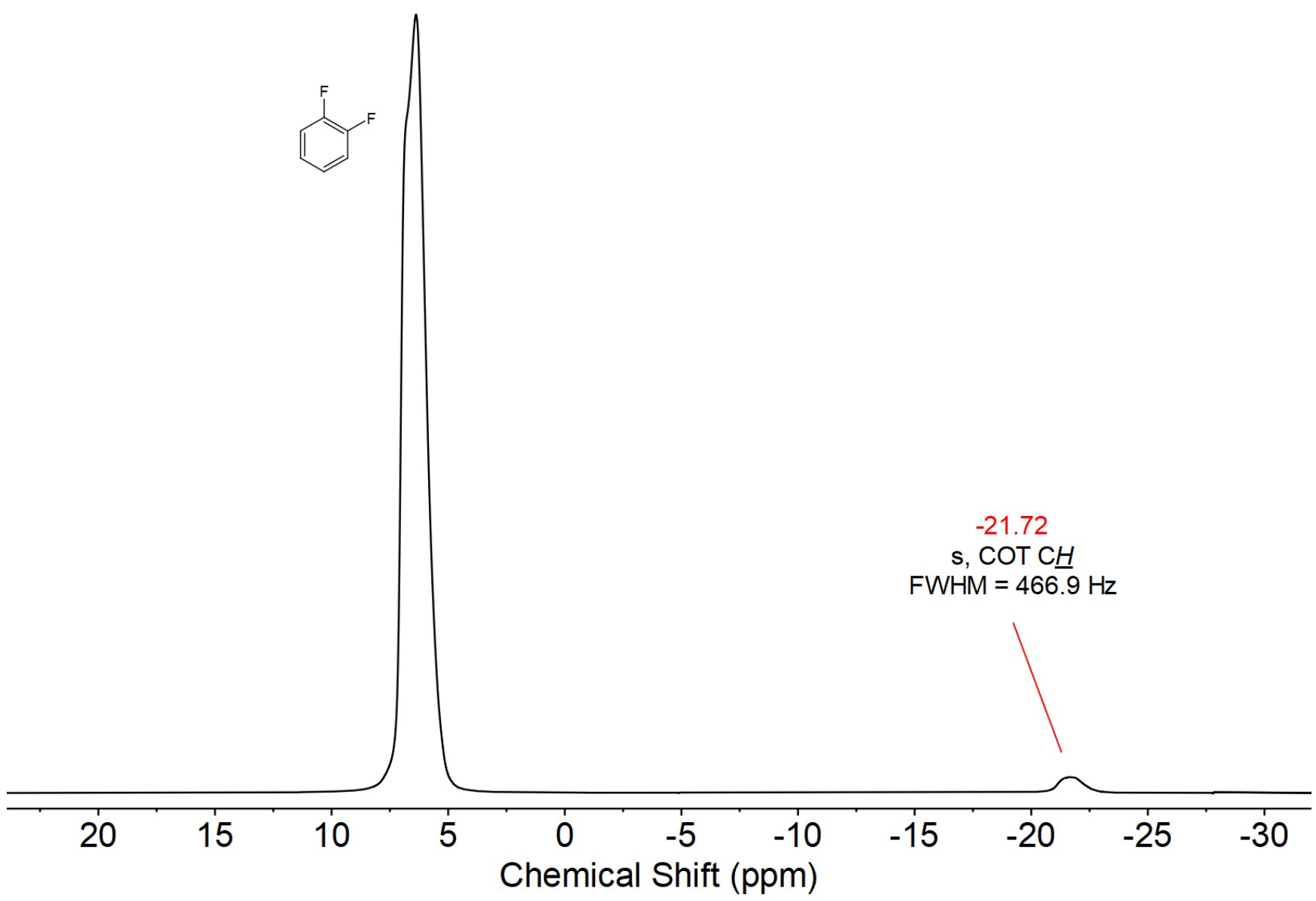

Figure S74. ^{1}H NMR spectrum of the crude reaction mixture between **1** and [Fc][B(C_6F_5)$_4$] in *o*DFB yielding **3** showing from +25 to –35 ppm.

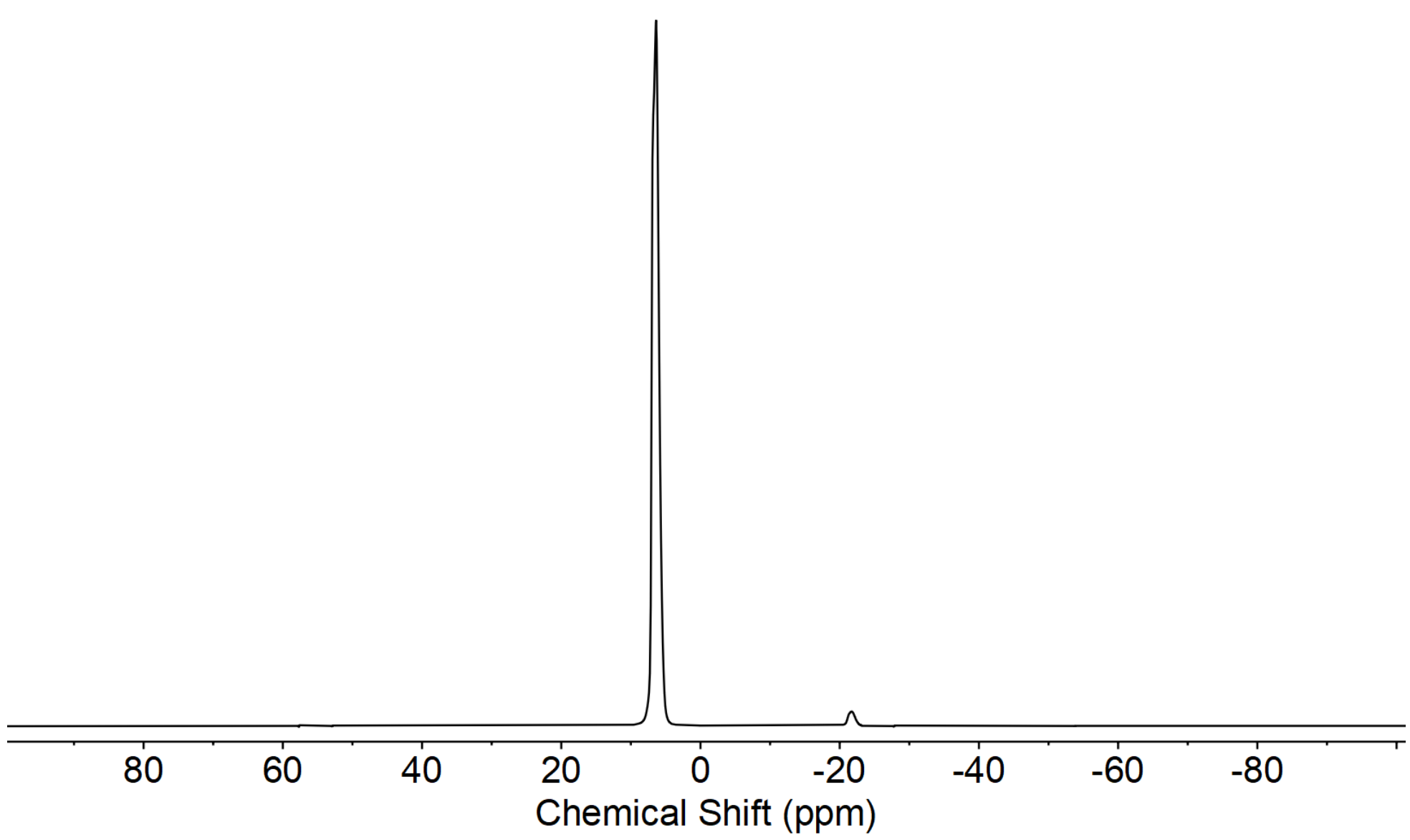

Figure S75. ^{1}H NMR spectrum of the crude reaction mixture between **1** and [Fc][B(C_6F_5)$_4$] in *o*DFB yielding **3** showing from +100 to –100 ppm.

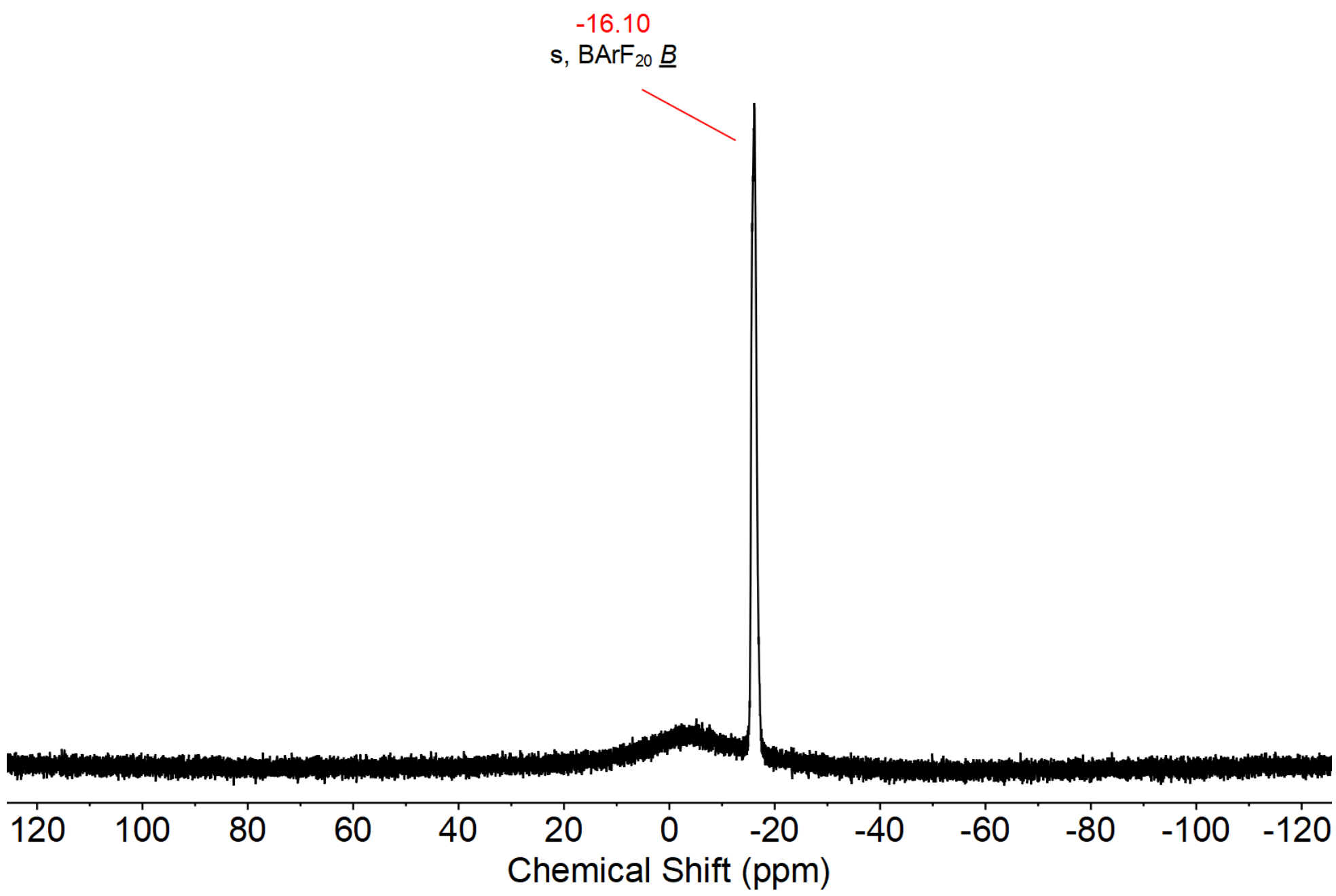

Figure S76. $^{11}B\{^1H\}$ NMR spectrum of the crude reaction mixture between **1** and $[Fc][B(C_6F_5)_4]$ in *o*DFB yielding **3**.

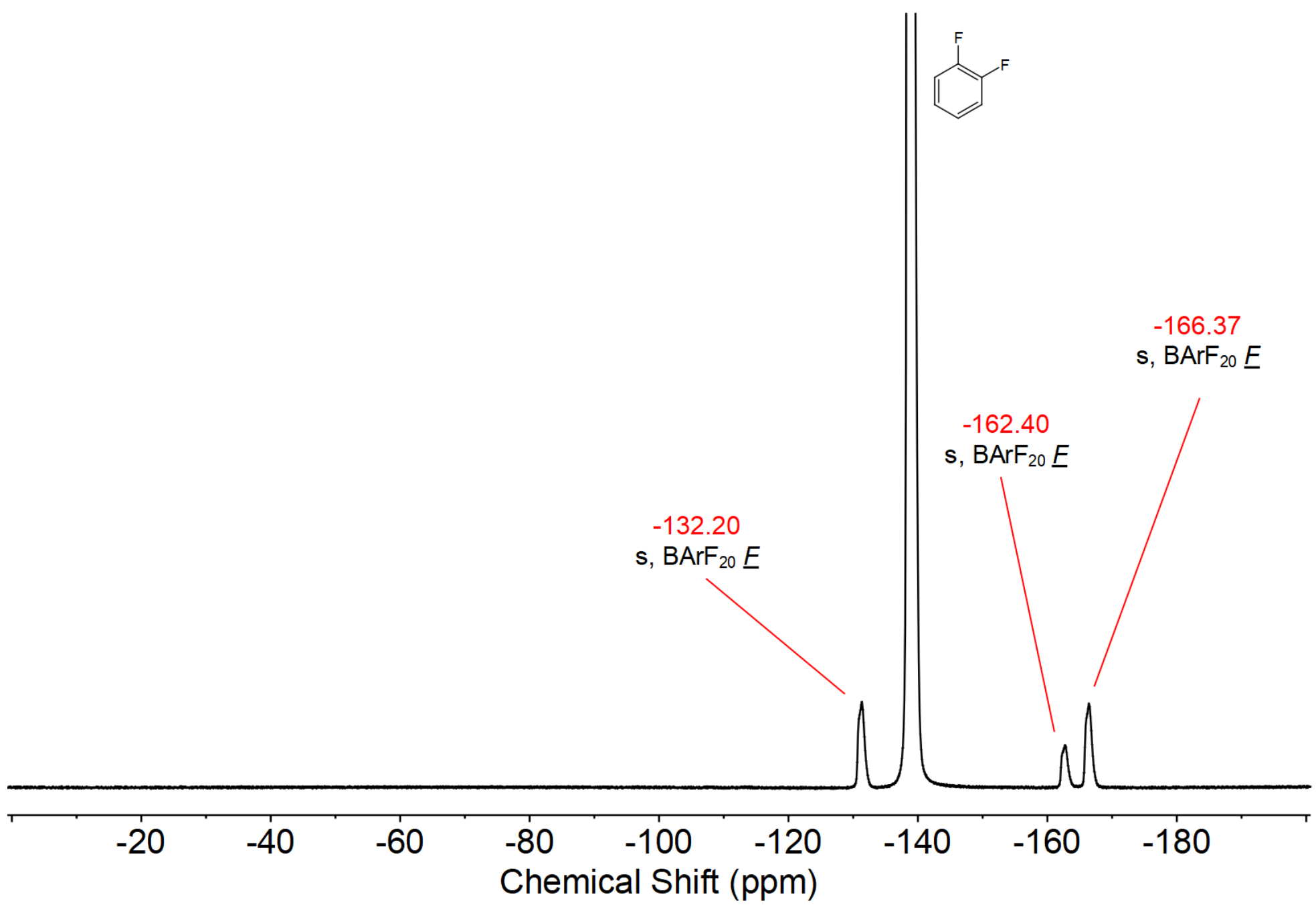

Figure S77. $^{19}F\{^1H\}$ NMR spectrum of the crude reaction mixture between **1** and $[Fc][B(C_6F_5)_4]$ in *o*DFB yielding **3**.

*The reaction between **1** and $Ag^{I}[B(C_6F_5)_4]$ in oDFB*

*o*DFB was added to a mixture of **1** (50 mg) and $Ag^{I}[B(C_6F_5)_4]$ (88 mg) in a borosilicate glass vial containing a PTFE-coated stirrer bar and resulted in an immediate colour change to dark brown and the precipitation of a dark precipitate. The suspension was stirred overnight and the supernatant filtered through a glass microfibre filter. Attempts to crystallise products from the reaction mixture were unsuccessful. Volatiles were removed under vacuum (10^{-3} mbar), and multinuclear NMR spectra were collected of the dried reaction mixture in *o*DFB which revealed resonances corresponding to **1** and an unknown COT containing compound.

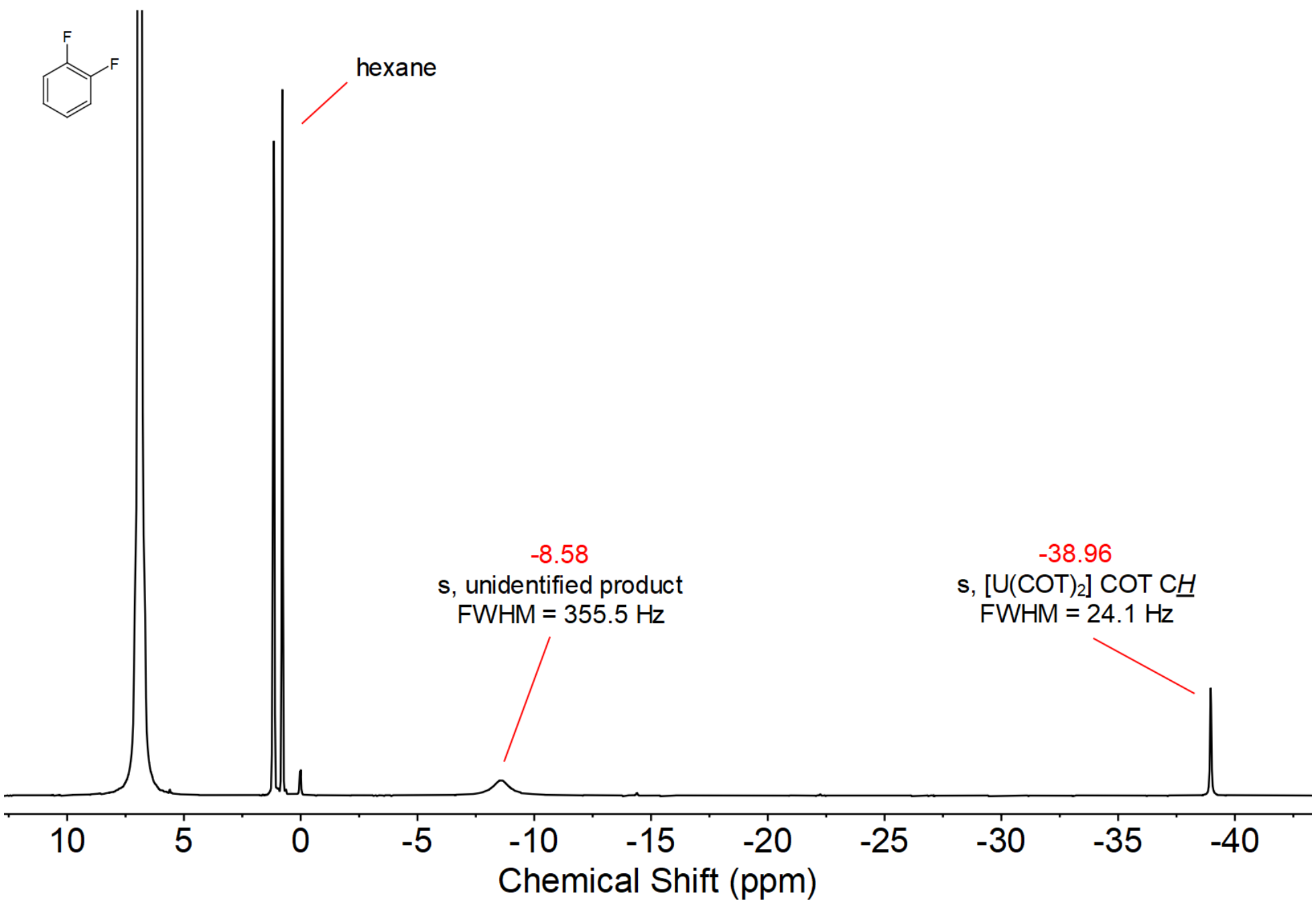

Figure S78. ^{1}H NMR spectrum of the reaction mixture between **1** and one equivalent of $Ag^{I}[B(C_6F_5)_4]$ in *o*DFB after stirring overnight at room temperature showing from +12 to –42 ppm.

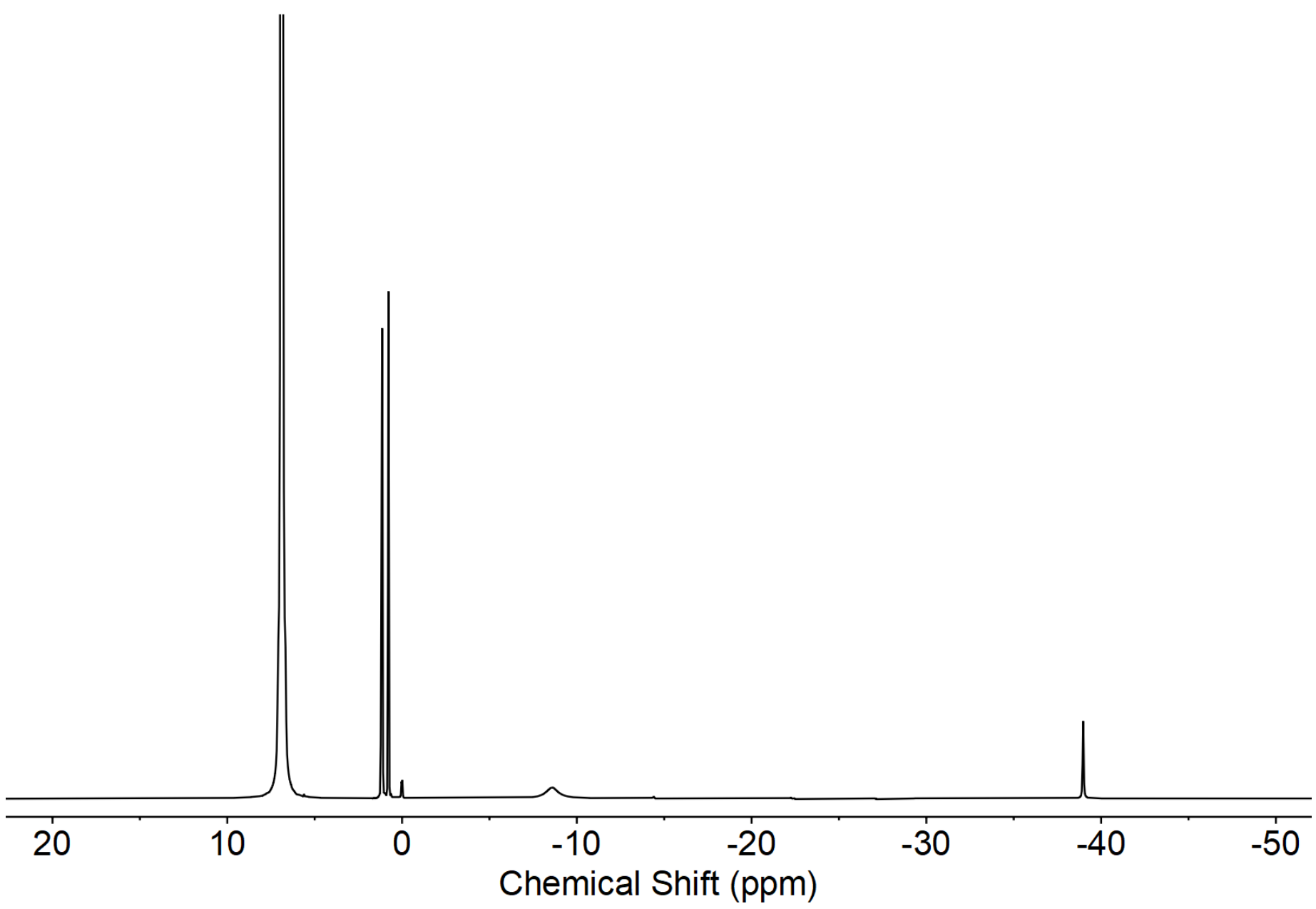

Figure S79. ^{1}H NMR spectrum of the reaction mixture between **1** and $Ag^{I}[B(C_6F_5)_4]$ in *o*DFB after stirring overnight at room temperature showing from +100 to –100 ppm.

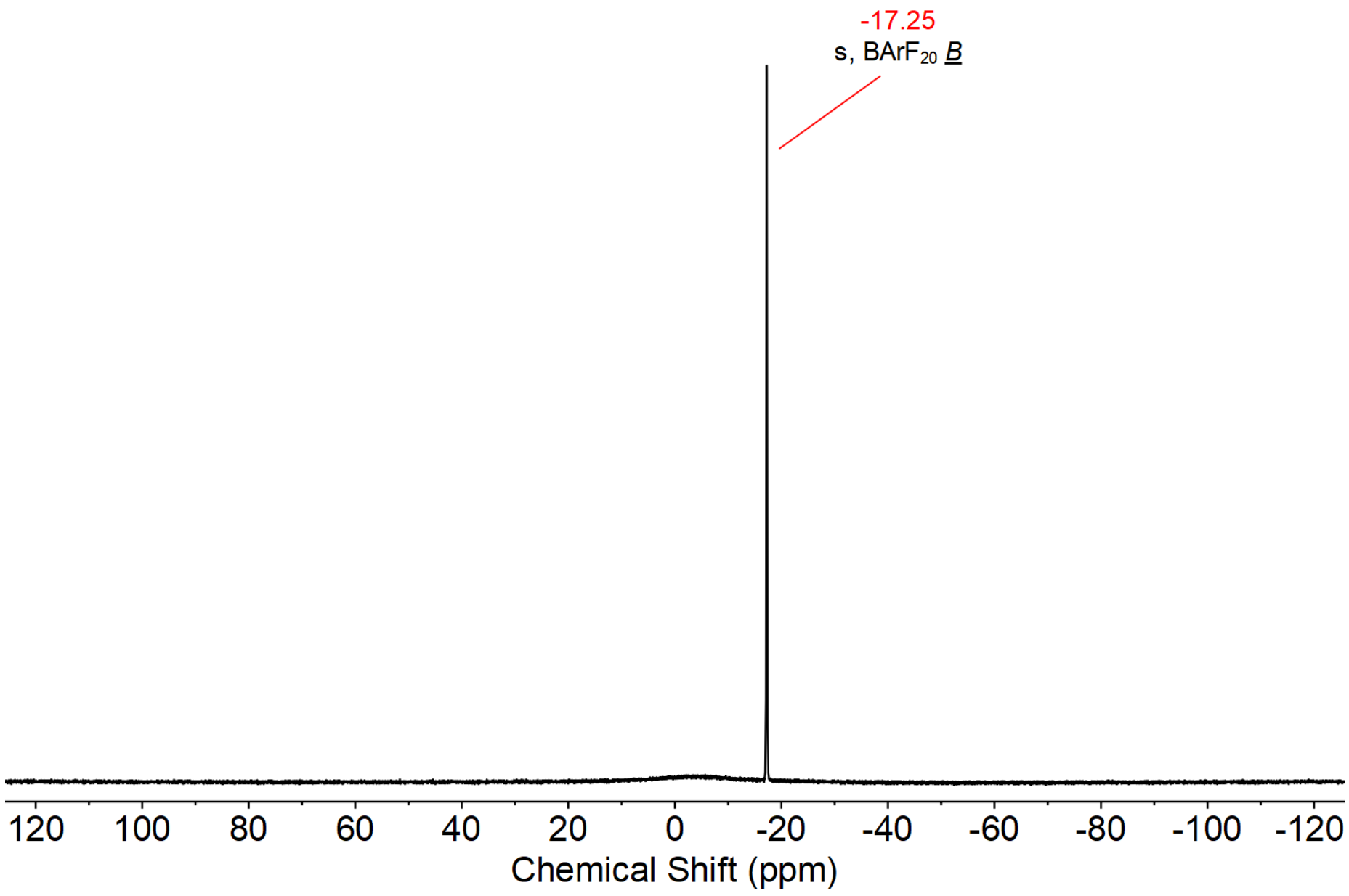

Figure S80. $^{11}B\{^{1}H\}$ NMR spectrum of the reaction mixture between **1** and one equivalent of $Ag^{I}[B(C_6F_5)_4]$ in *o*DFB after stirring overnight at room temperature.

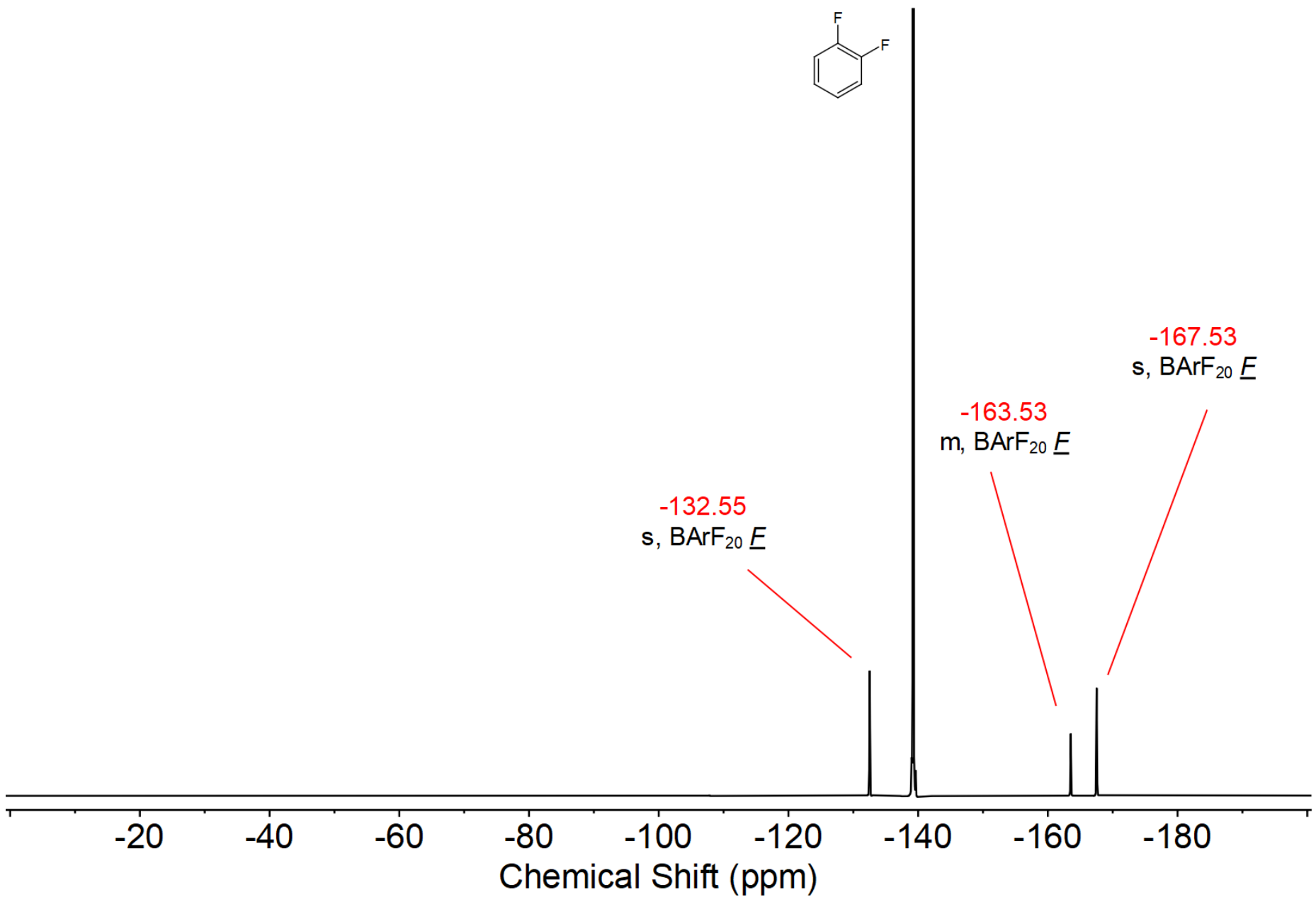

Figure S81. $^{19}F\{^{1}H\}$ NMR spectrum of the reaction mixture between **1** and one equivalent of $Ag^{I}[B(C_6F_5)_4]$ in *o*DFB after stirring overnight at room temperature.

*The reaction between **1** and $Ag^I[B\{C_6H_3\text{-}3,5\text{-}(CF_3)_2\}_4]$ in oDFB*

*o*DFB was added to a mixture of **1** (50 mg) and $Ag^I[B\{C_6H_3\text{-}3,5\text{-}(CF_3)_2\}_4]$ (109 mg) in a borosilicate glass vial containing a PTFE-coated stirrer bar, which resulted in an immediate colour change to dark brown and the precipitation of a dark-coloured precipitate. The suspension was stirred overnight and the supernatant filtered through a glass microfibre filter. Attempts to crystallise products from the reaction mixture were unsuccessful. Volatiles were removed under vacuum (10^{-3} mbar), and multinuclear NMR spectra were collected of the dried reaction mixture in *o*DFB, which revealed resonances corresponding to an unknown COT containing compound.

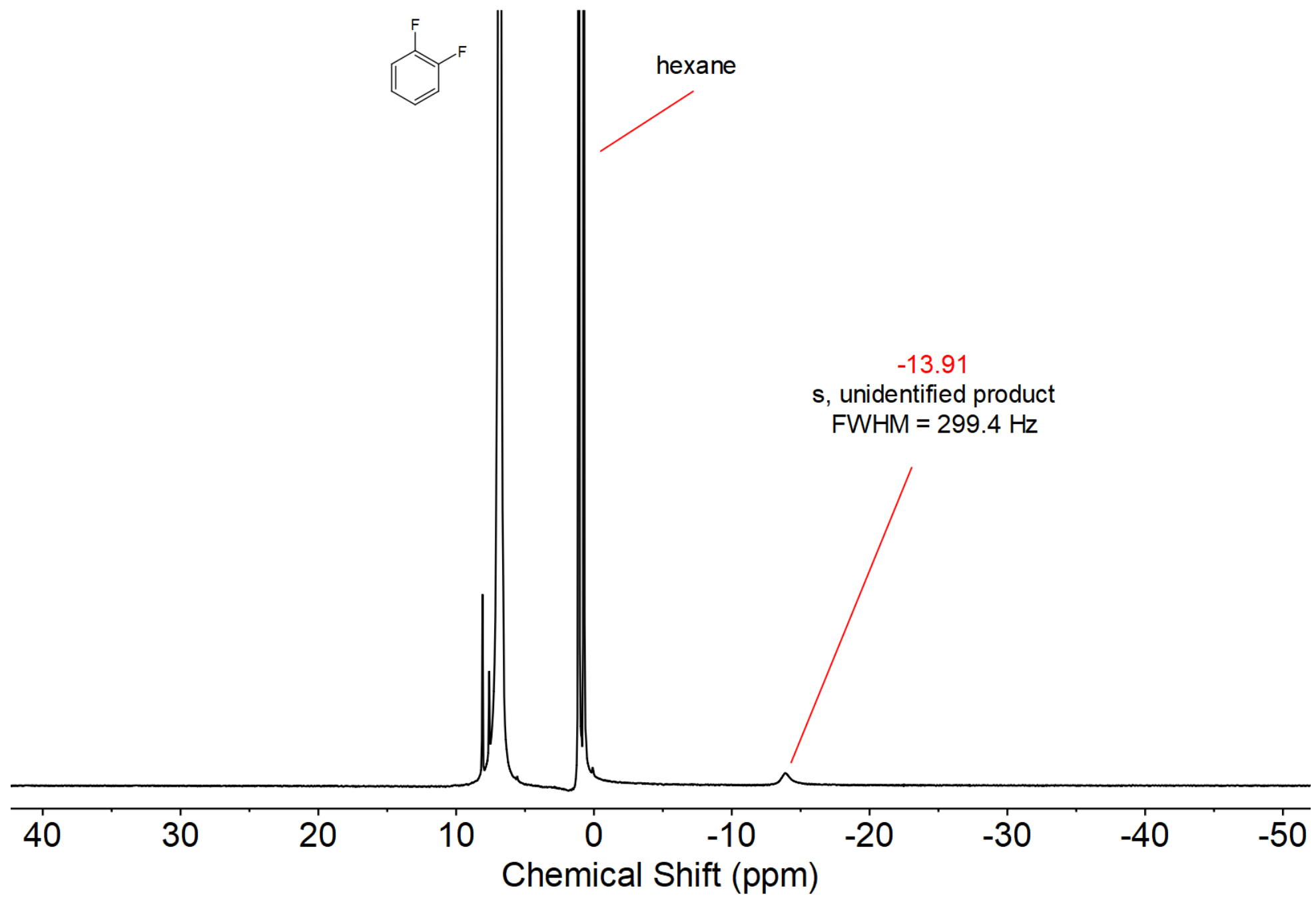

Figure S82. ^{1}H NMR spectrum of the reaction mixture between **1** and one equivalent of $Ag^I[B\{C_6H_3\text{-}3,5\text{-}(CF_3)_2\}_4]$ in *o*DFB after stirring overnight at room temperature showing from +40 to –50 ppm.

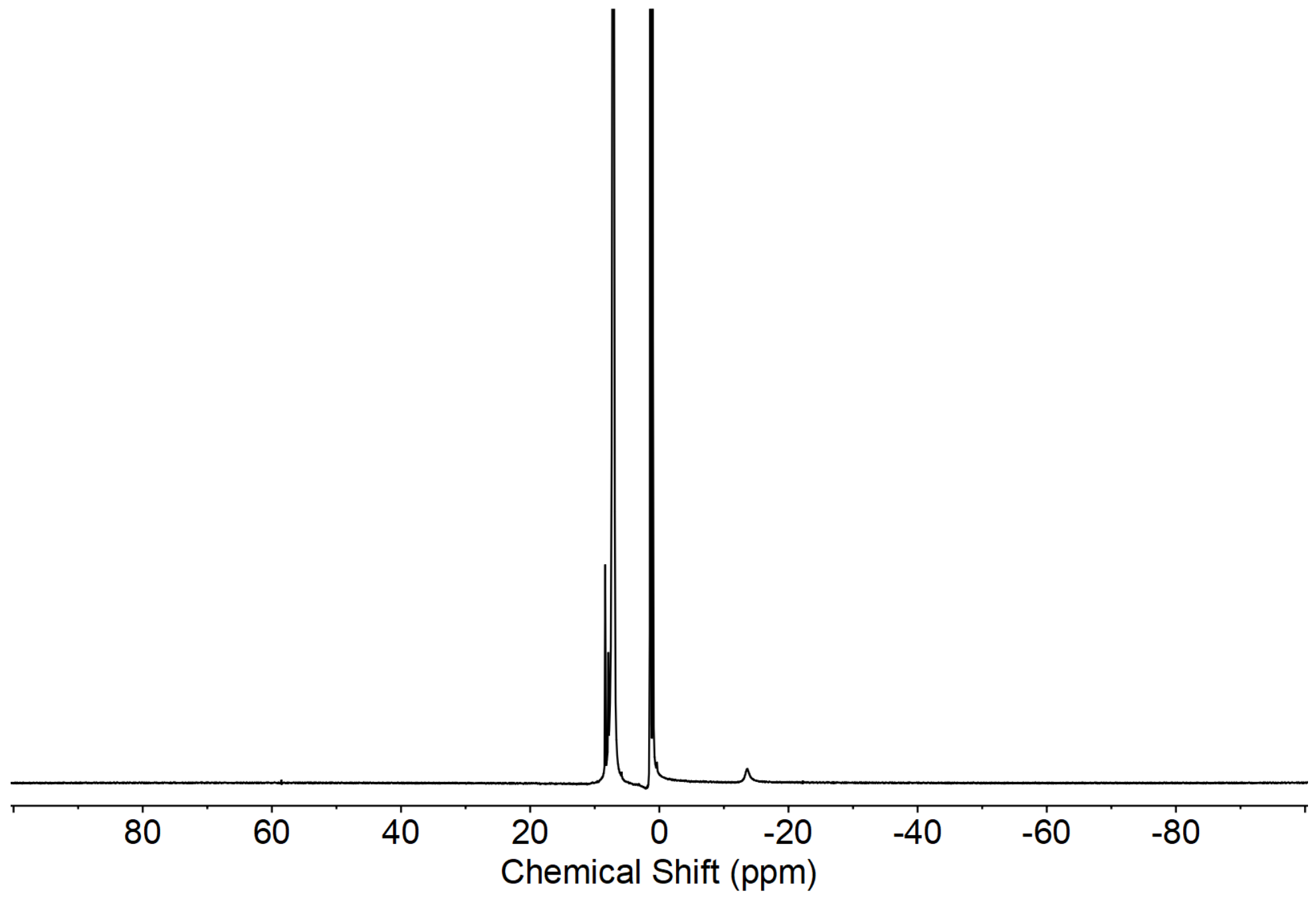

Figure S83. ^{1}H NMR spectrum of the reaction mixture between **1** and one equivalent of $Ag^{I}[B\{C_6H_3\text{-}3,5\text{-}(CF_3)_2\}_4]$ in *o*DFB after stirring overnight at room temperature showing from +100 to –100 ppm.

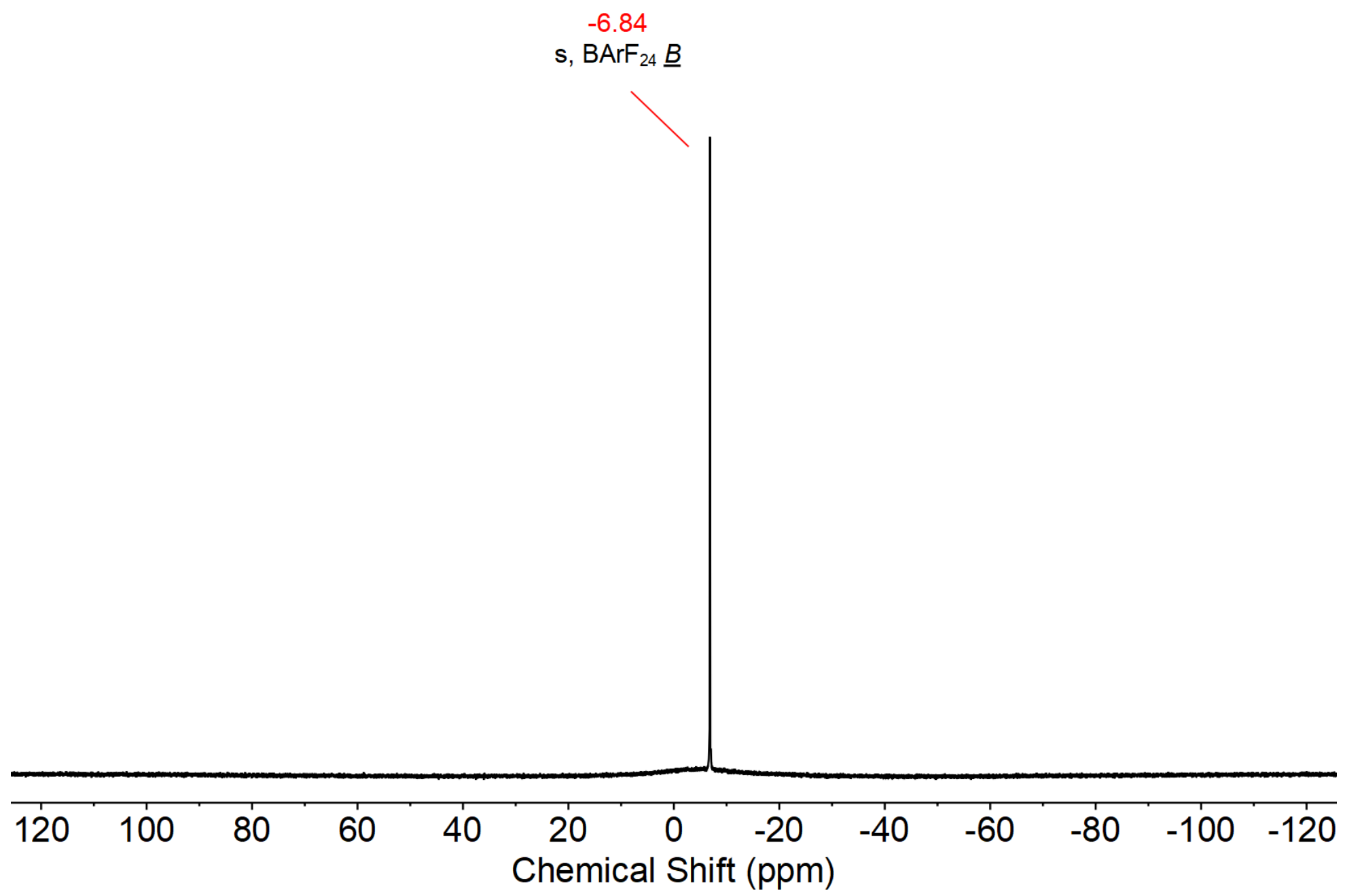

Figure S84. $^{11}B\{^{1}H\}$ NMR spectrum of the reaction mixture between **1** and one equivalent of $Ag^{I}[B\{C_6H_3\text{-}3,5\text{-}(CF_3)_2\}_4]$ in *o*DFB after stirring overnight at room temperature.

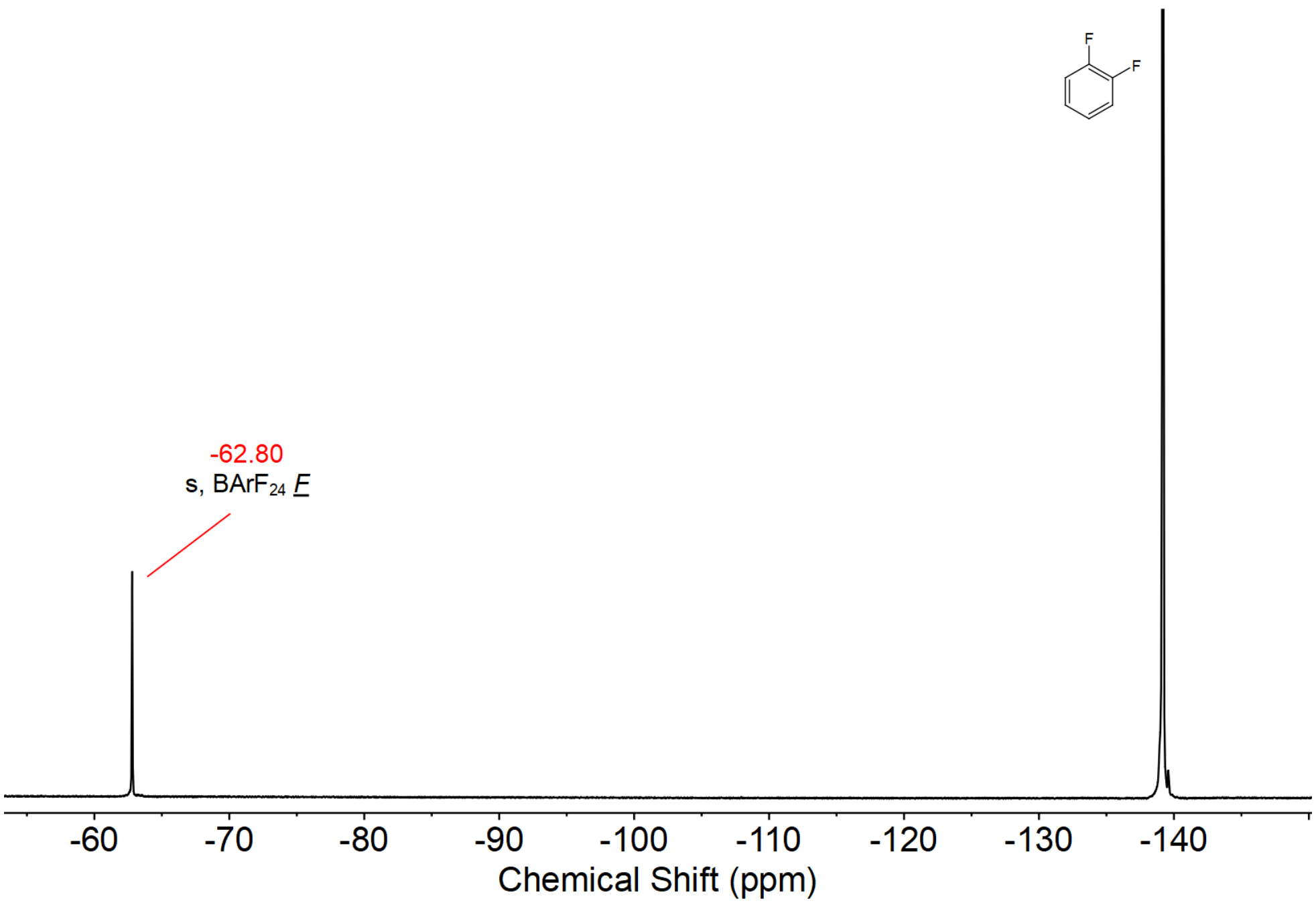

Figure S85. $^{19}F\{^1H\}$ NMR spectrum of the reaction mixture between **1** and one equivalent of $Ag^I[B\{C_6H_3\text{-}3,5\text{-}(CF_3)_2\}_4]$ in *o*DFB after stirring overnight at room temperature.

*The reaction between **1** and $Ag^{I}[SbF_6]$ in oDFB*

*o*DFB was added to a mixture of **1** (20 mg) and $Ag[SbF_6]$ (15 mg) in a J. Youngs appended NMR tube and resulted in a slow colour change to dark brown, along with the precipitation of a dark-coloured material and the formation of a silver mirror. After three hours, a ^{1}H NMR spectrum was collected of the suspension (below), which showed neutral COT as the only identifiable resonance.

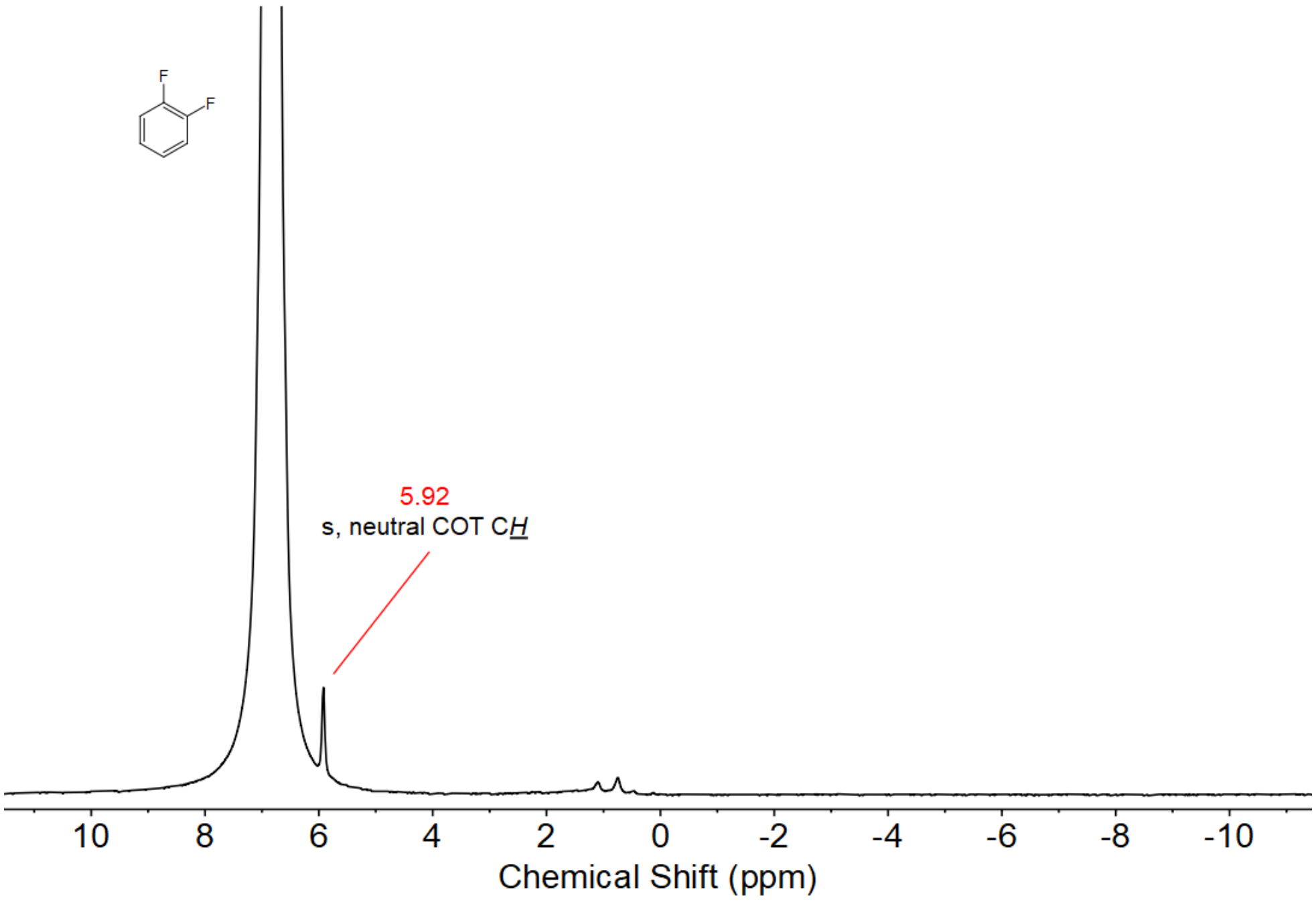

Figure S86. ^{1}H NMR spectrum of the reaction mixture between **1** and one equivalent of $Ag^{I}[SbF_6]$ in *o*DFB which yielded a black insoluble precipitate and produced a silver mirror on the walls of the NMR tube showing from +12 to –42 ppm.

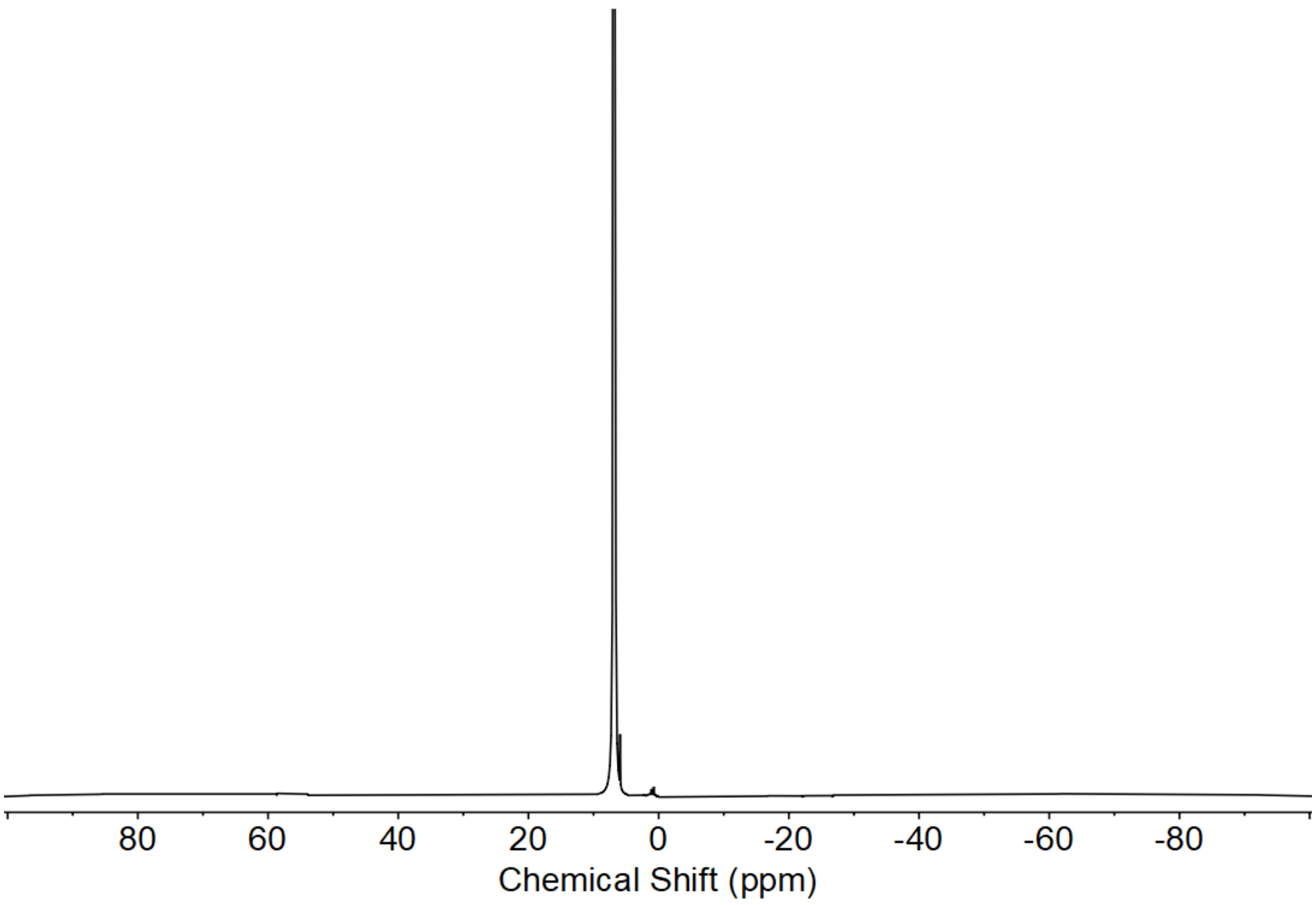

Figure S87. ^{1}H NMR spectrum of the reaction mixture between **1** and one equivalent of $Ag^{I}[SbF_6]$ in *o*DFB which yielded a black insoluble precipitate and produced a silver mirror on the walls of the NMR tube showing from +100 to –100 ppm.

*The reaction between **3** and [nBu$_4$N][PF$_6$]*

*o*DFB was added to a J. Youngs appended NMR tube containing the dark, insoluble product from the reaction between **3** and [*n*Bu$_4$N][PF$_6$] (20 mg). A ^{19}F{^{1}H} NMR spectrum was recorded of the suspension immediately, and then every 24 hours thereafter until 144 hours had elapsed. Over time, resonances corresponding to PF_5, PF_3 and POF_3 in the ^{19}F{^{1}H} spectra slowly grew in. Only a very weak resonance corresponding to POF_3 could be seen in the ^{31}P{^{1}H} spectrum after 144 hours. These data suggest a similar fluoride abstraction reaction occurs with this complex to that observed with the formation of **7** from **6**.

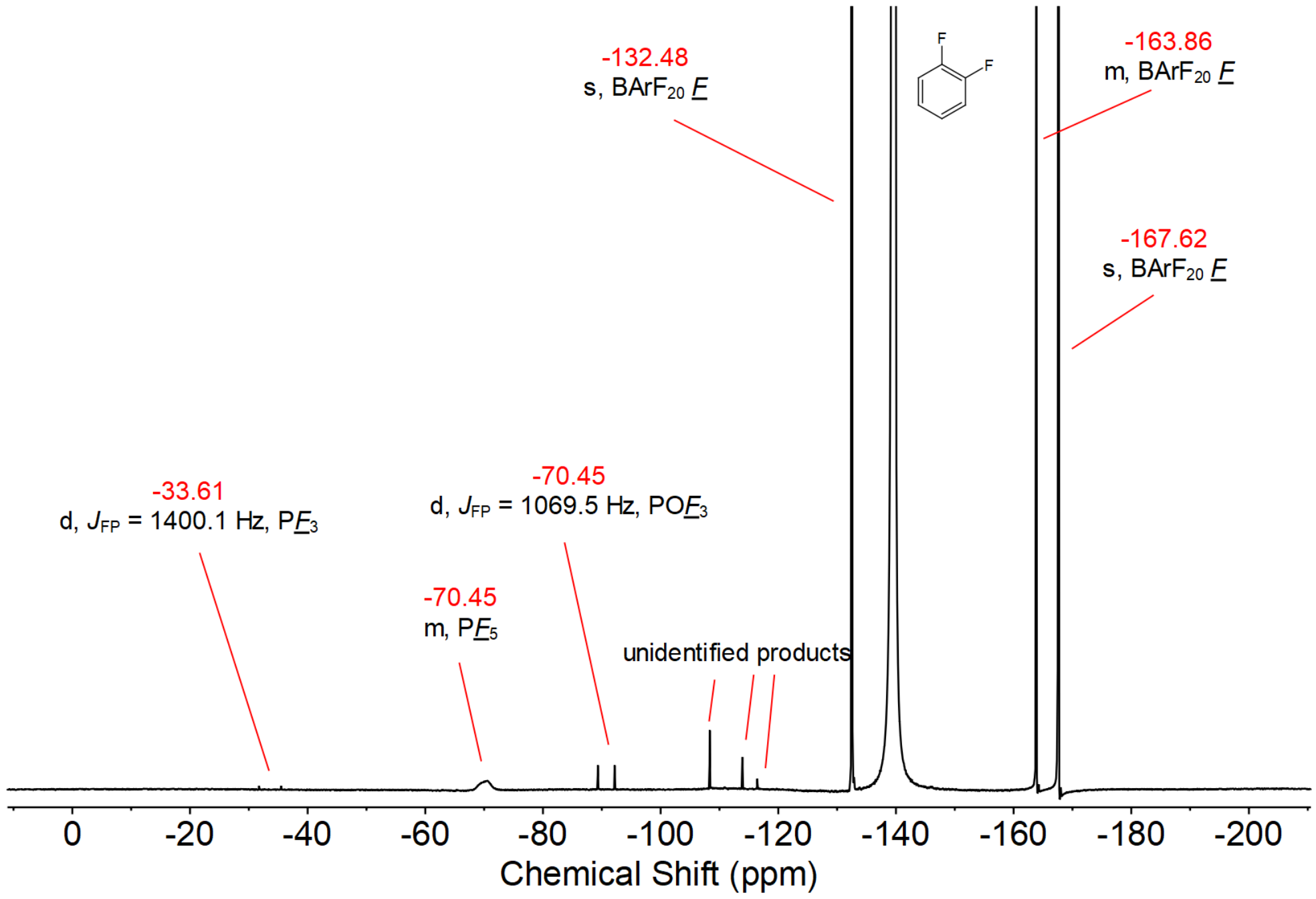

Figure S88. ^{19}F{^{1}H} NMR spectrum of the reaction between **3** and [*n*Bu$_4$N][PF$_6$] in *o*DFB after 144 hours at room temperature. The reaction yields dark insoluble material which slowly decomposes to PF_5, PF_3 and POF_3 in addition to other unidentified reaction products.

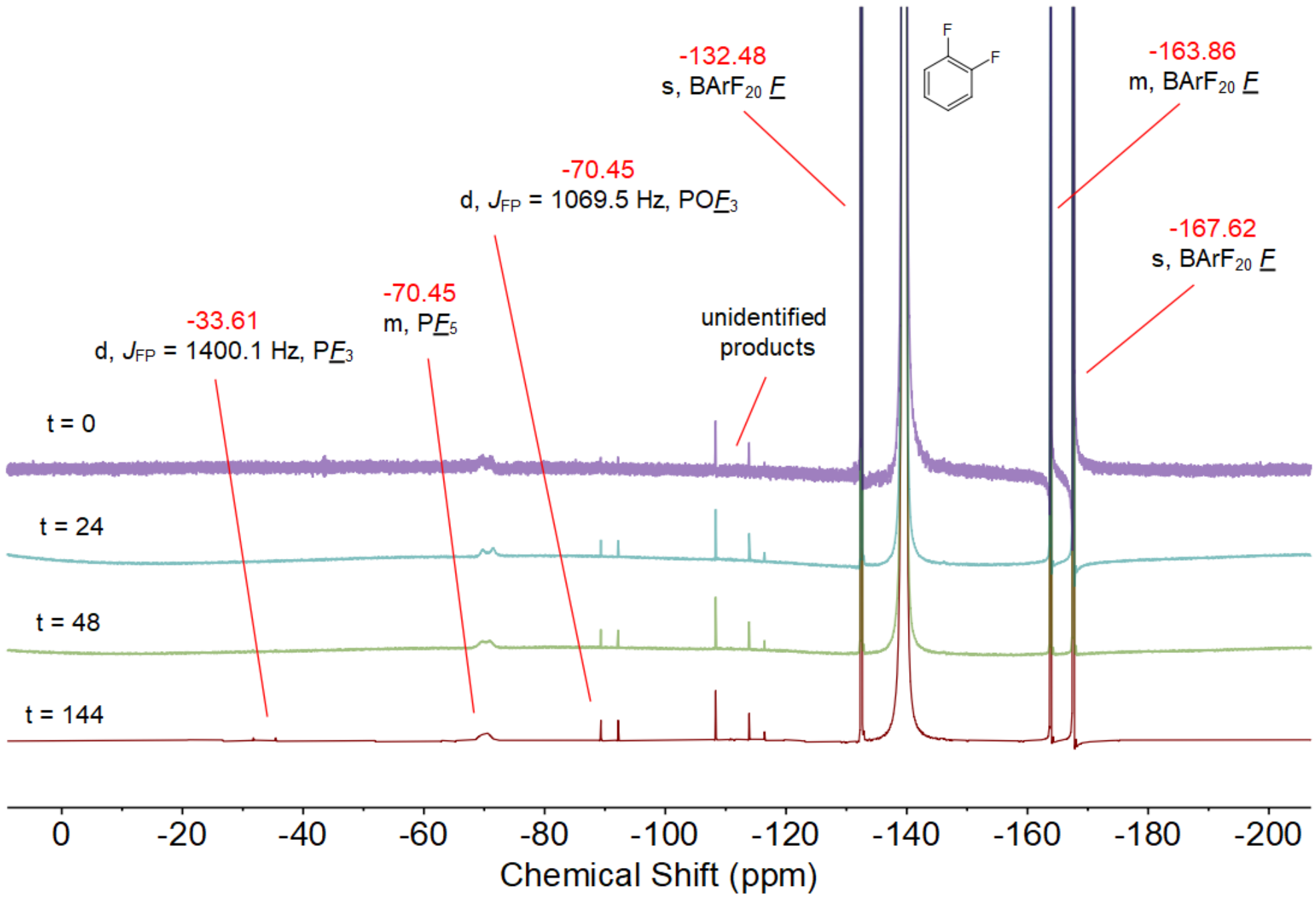

Figure S89. Sequential $^{19}F\{^1H\}$ NMR spectra of the reaction between **3** and $[nBu_4N][PF_6]$ in *o*DFB taken after 0, 24, 48 and 144 hours at room temperature.

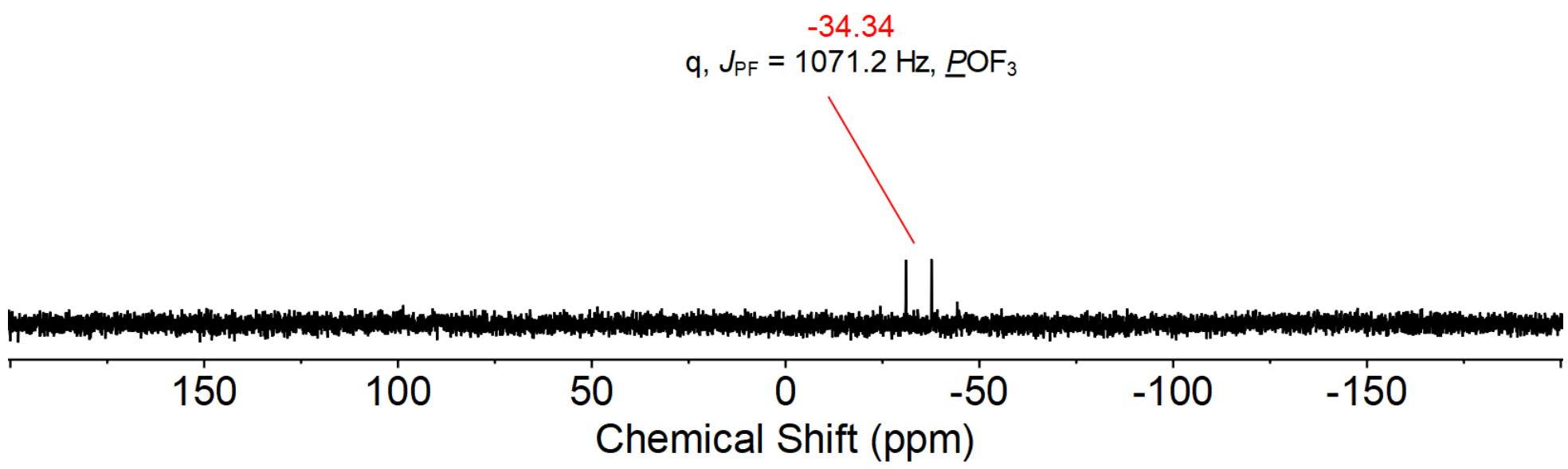

Figure S90. $^{31}P\{^1H\}$ NMR spectrum of the reaction between **3** and $[nBu_4N][PF_6]$ in *o*DFB after 144 hours at room temperature. The reaction yields dark insoluble material which slowly decomposes to PF_5, PF_3 and POF_3 in addition to other unidentified reaction products although only POF_3 could be identified in the ^{31}P NMR spectrum.

*The reaction between **6** and [nBu$_4$N][PF$_6$]*

To better understand the previously described irreversible oxidation events associated with electrochemistry of **1** in the presence of [*n*Bu$_4$N][PF$_6$], we exposed complex **6** to [*n*Bu$_4$N][PF$_6$] in d_5-bromobenzene and examined the resultant multinuclear NMR spectra. Complex **6** was studied due to the increased solubility provided by the COT†† ligand, as when the same reaction was explored with complex **3**, insoluble material was obtained which precluded characterization.

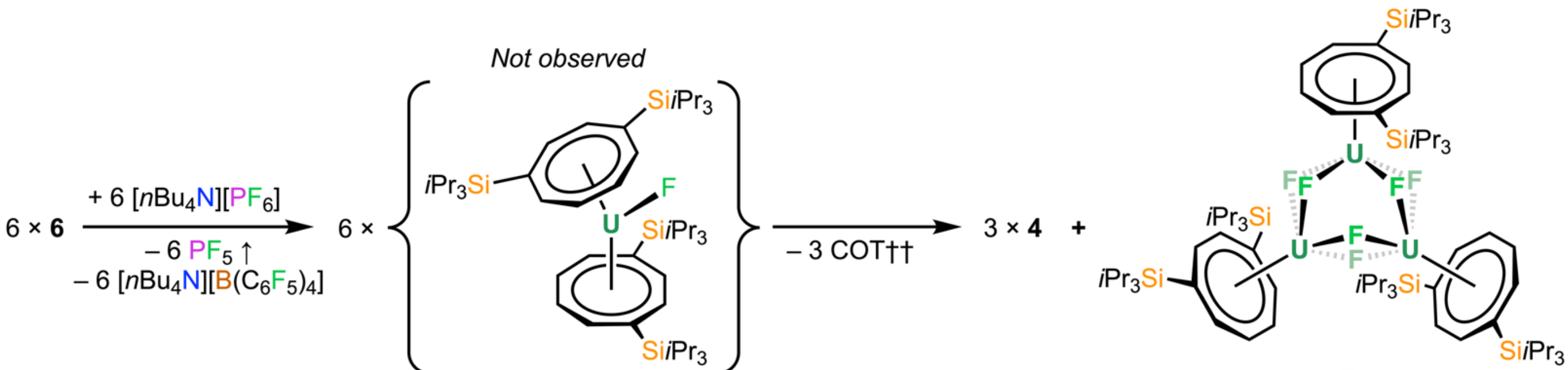

Scheme 1. The reaction between **6** and [*n*Bu$_4$N][PF$_6$], which forms **4**, **7**, C_8H_6-1,4-(Si*i*Pr$_3$)$_2$, and PF_5.

Integration of NMR spectra from the reaction described above allowed us to tentatively propose a reaction pathway for the formation of **7**, which is shown in **Scheme 1**. We were unable to conclusively assign the ^{19}F and ^{31}P NMR spectra of the reaction mixture in d_5-bromobenzene due to the paucity of spectral data reported for some of the reaction products, specifically PF_5. The reaction was repeated in *o*DFB, then, once complete, H$_8$-THF was added to form PF_5–THF adduct for comparison to a prior report [20].

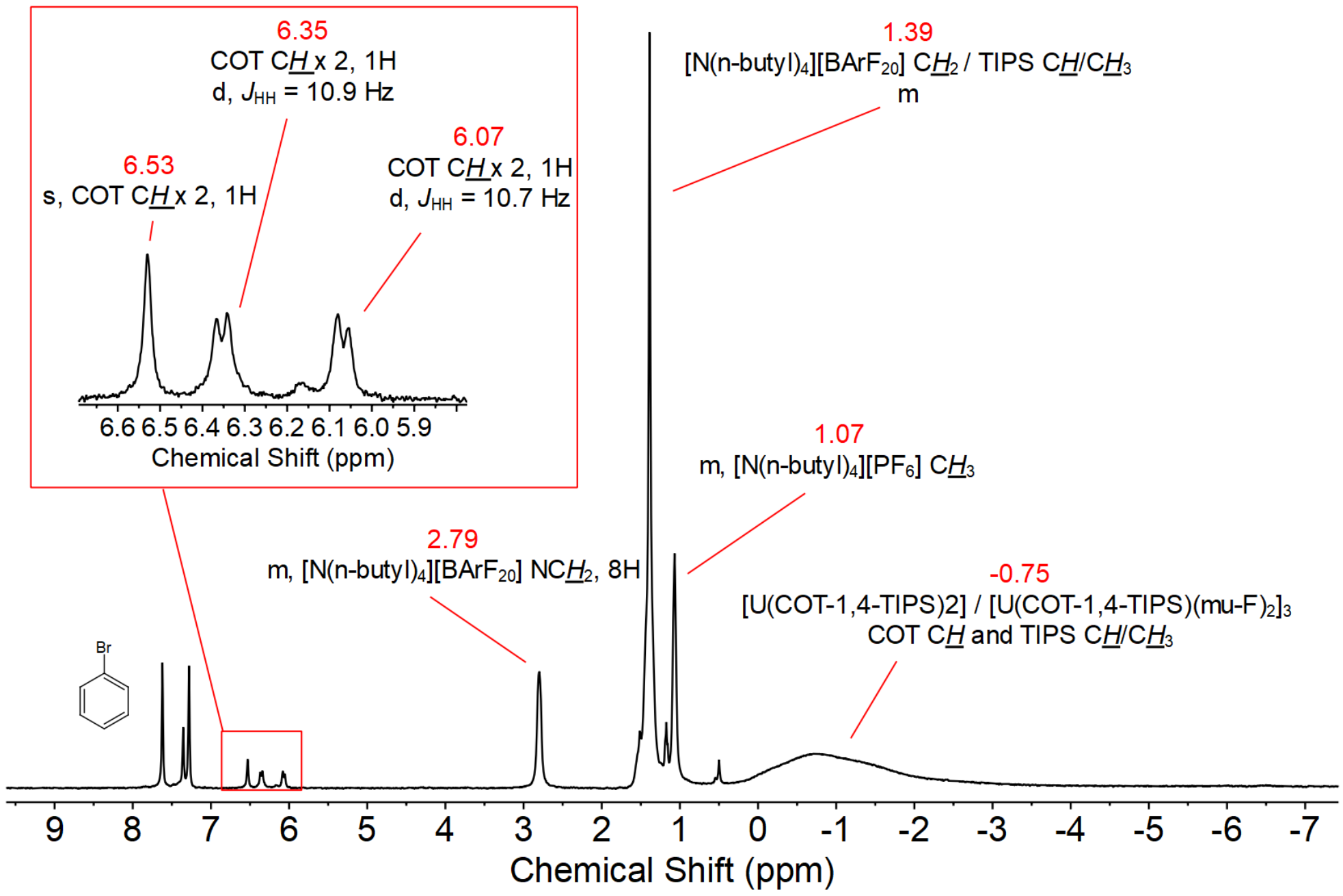

Figure S91. ^{1}H NMR spectrum of the crude reaction mixture between **6** and 1 eq. [nBu$_4$N][PF$_6$] in d_5-bromobenzene showing from +9 to –7 ppm.

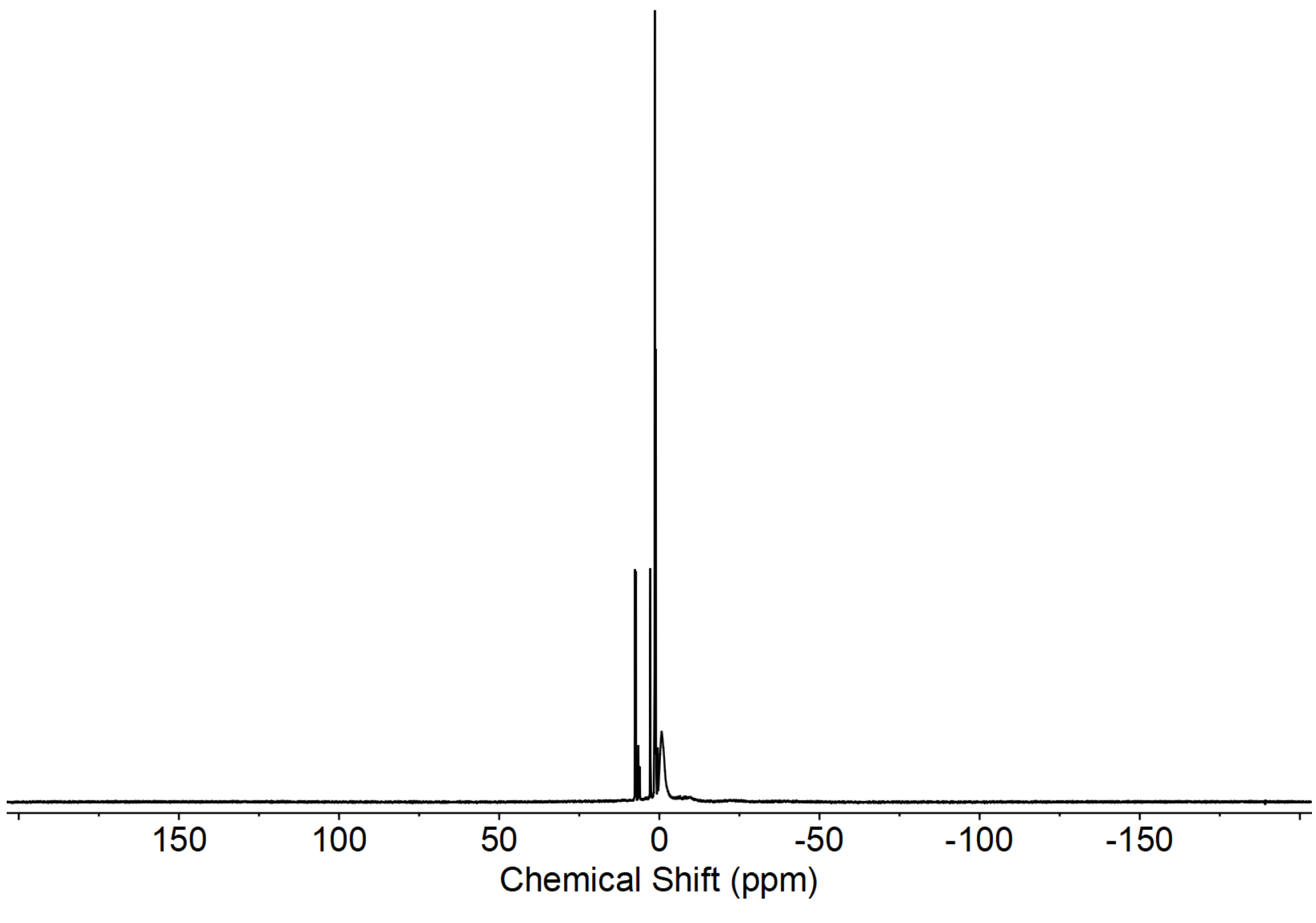

Figure S92. ^{1}H NMR spectrum of the crude reaction mixture between **6** and 1 eq. [nBu$_4$N][PF$_6$] in d_5-bromobenzene showing from +200 to –200 ppm.

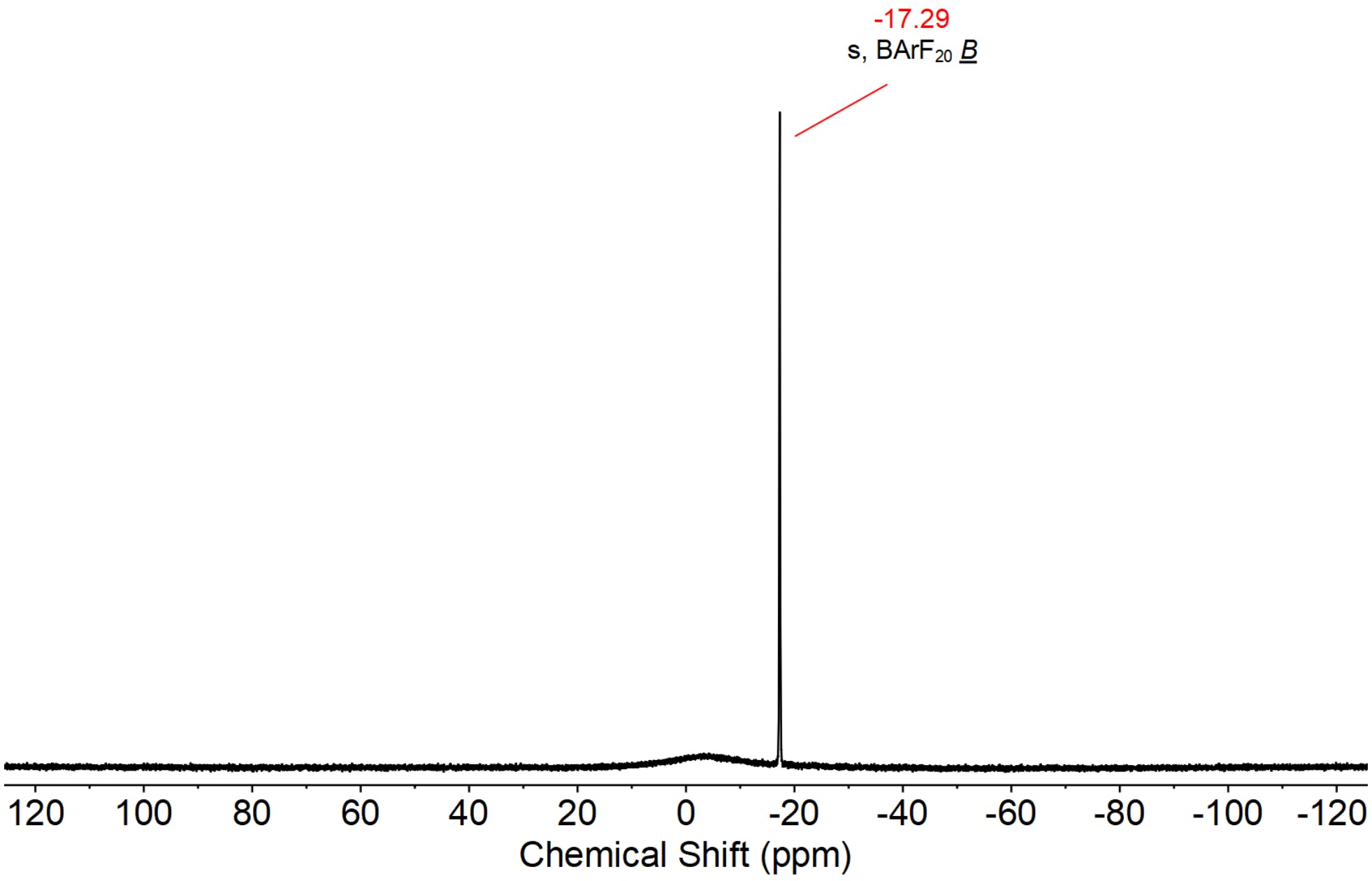

Figure S93. $^{11}B\{^1H\}$ NMR spectrum of the crude reaction mixture between **6** and 1 eq. [nBu$_4$N][PF$_6$] in d_5-bromobenzene.

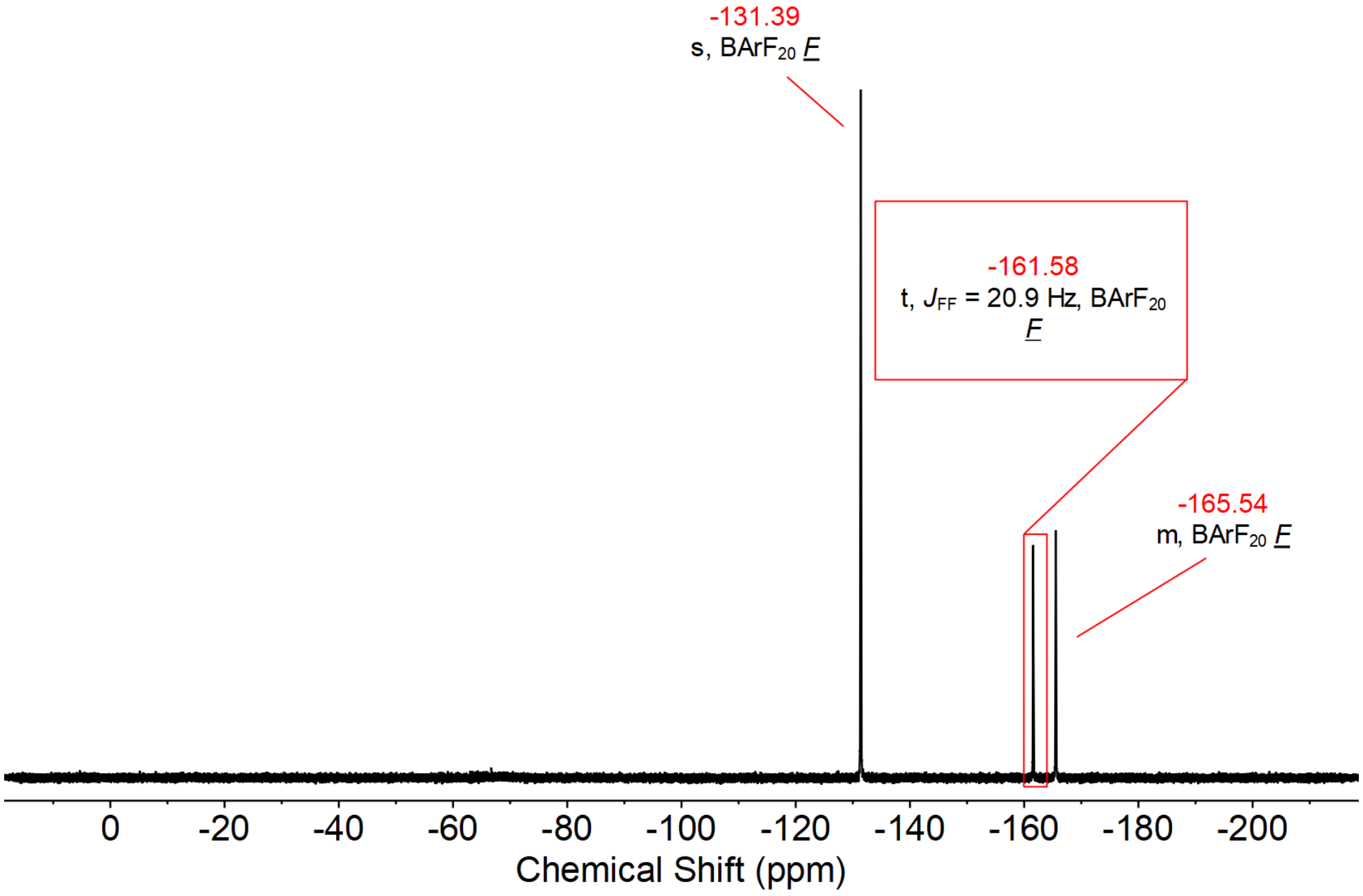

Figure S94. $^{19}F\{^1H\}$ NMR spectrum of the crude reaction mixture between **6** and 1 eq. [nBu$_4$N][PF$_6$] in d_5-bromobenzene.

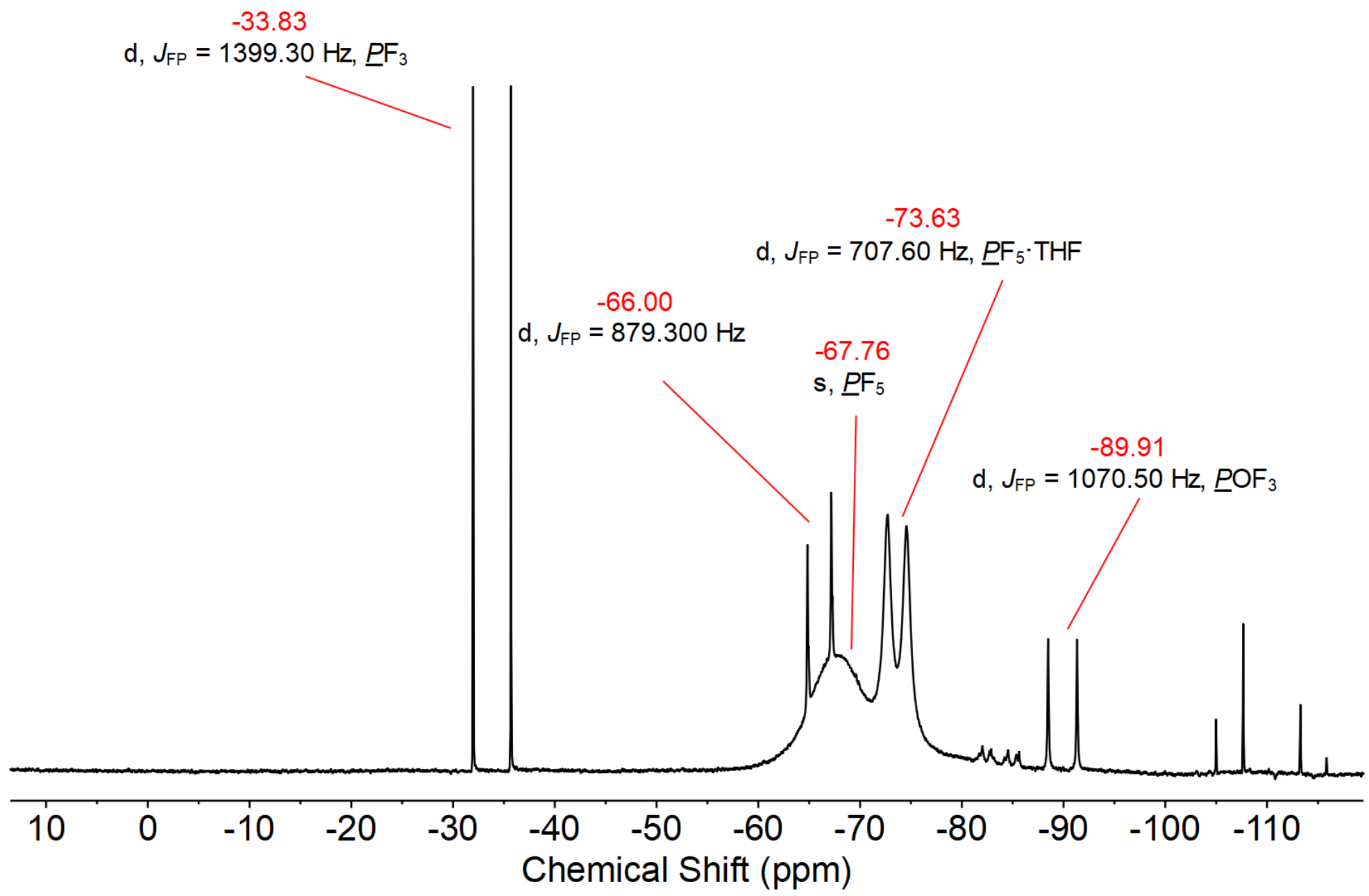

Figure S95. $^{19}F\{^{1}H\}$ NMR spectrum of the crude reaction mixture between **6** and 1 eq. [*n*Bu$_4$N][PF$_6$] in *o*DFB with a few drops of H$_8$-THF.

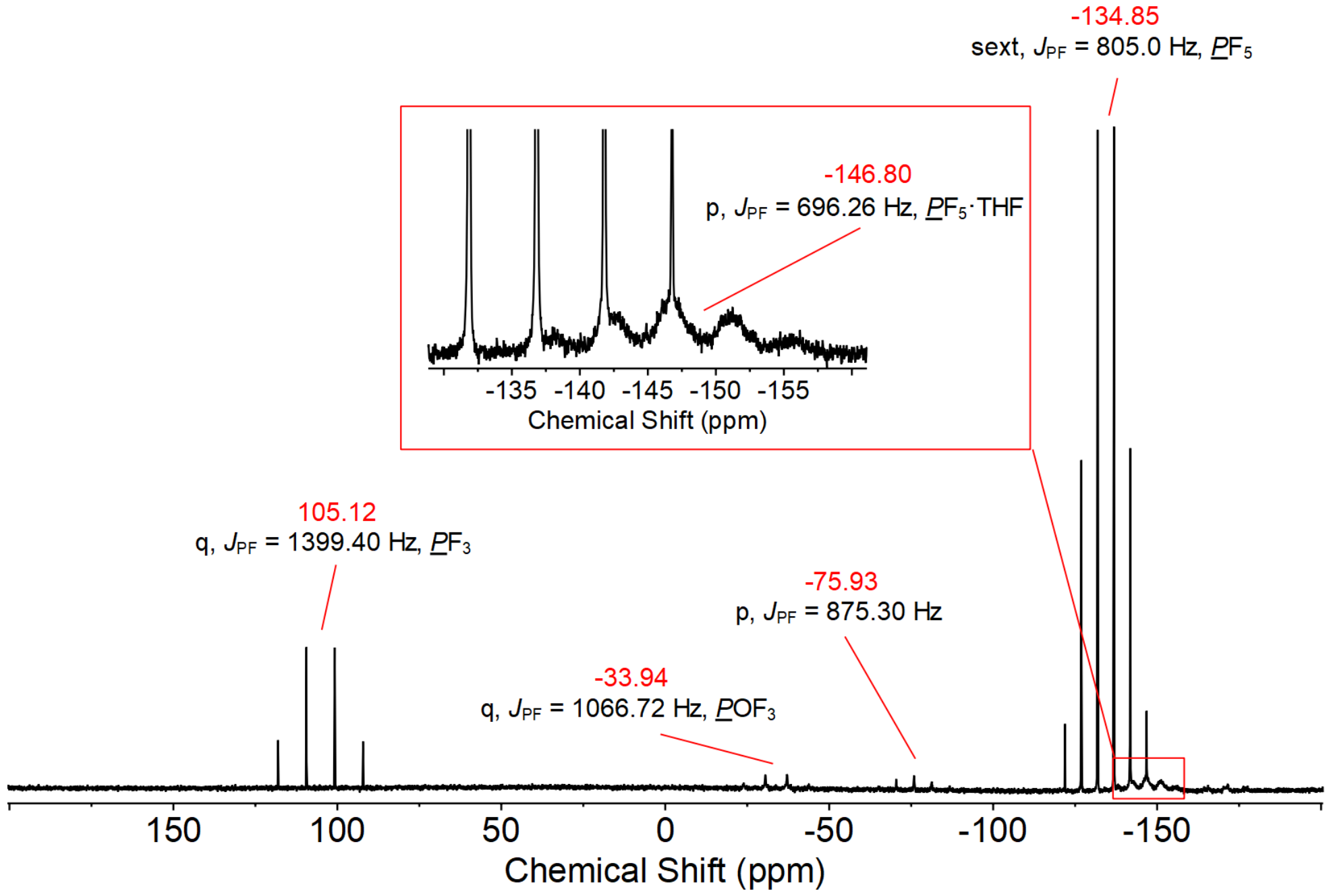

Figure S96. $^{31}P\{^{1}H\}$ NMR spectrum of the crude reaction mixture between **6** and 1 eq. [*n*Bu$_4$N][PF$_6$] in *o*DFB with a few drops of H$_8$-THF (to make THF–PF$_5$ and reduce fluxionality in the PF$_5$ unit).

*The reaction between **6** and [nBu$_4$N][SbF$_6$]*

d$_5$-bromobenzene was added to a mixture of **6** (10 mg) and [*n*Bu$_4$N][SbF$_6$] (2.73 mg) in a J. Youngs appended NMR tube and analysed immediately by multinuclear NMR spectroscopy. We observe an upfield shift in the ^{1}H NMR of the COT†† TIPS and CH peaks relative to **6** in *d*$_5$-bromobenzene, and we could also observe a broad peak in the ^{19}F{1H} NMR corresponding to the [SbF$_6$] anion in addition to three sharp resonances corresponding to the [B(C$_6$F$_5$)$_4$] anion. The red solution was found to decompose at room temperature over the course of a few hours, resulting in the formation of a black precipitate that is insoluble in both *d*$_5$-bromobenzene and THF. The decomposition process, which produces the insoluble material, starts immediately, evidenced by the presence of peaks corresponding to neutral COT†† in the ^{1}H NMR spectrum. After 24 hours, the suspension was analysed by multinuclear NMR spectroscopy again, and only showed peaks corresponding to neutral COT†† and the [*n*Bu$_4$N] cation in roughly 1:1 ratio by ^{1}H NMR spectroscopy, and only peaks corresponding to the [B(C$_6$F$_5$)$_4$] anion in the ^{19}F{1H} NMR spectrum. These data suggest that [*n*Bu$_4$N][B(C$_6$F$_5$)$_4$] is one product of the reaction, along with one equivalent of neutral COT††, and that the black precipitate contains all the uranium and antimony content.

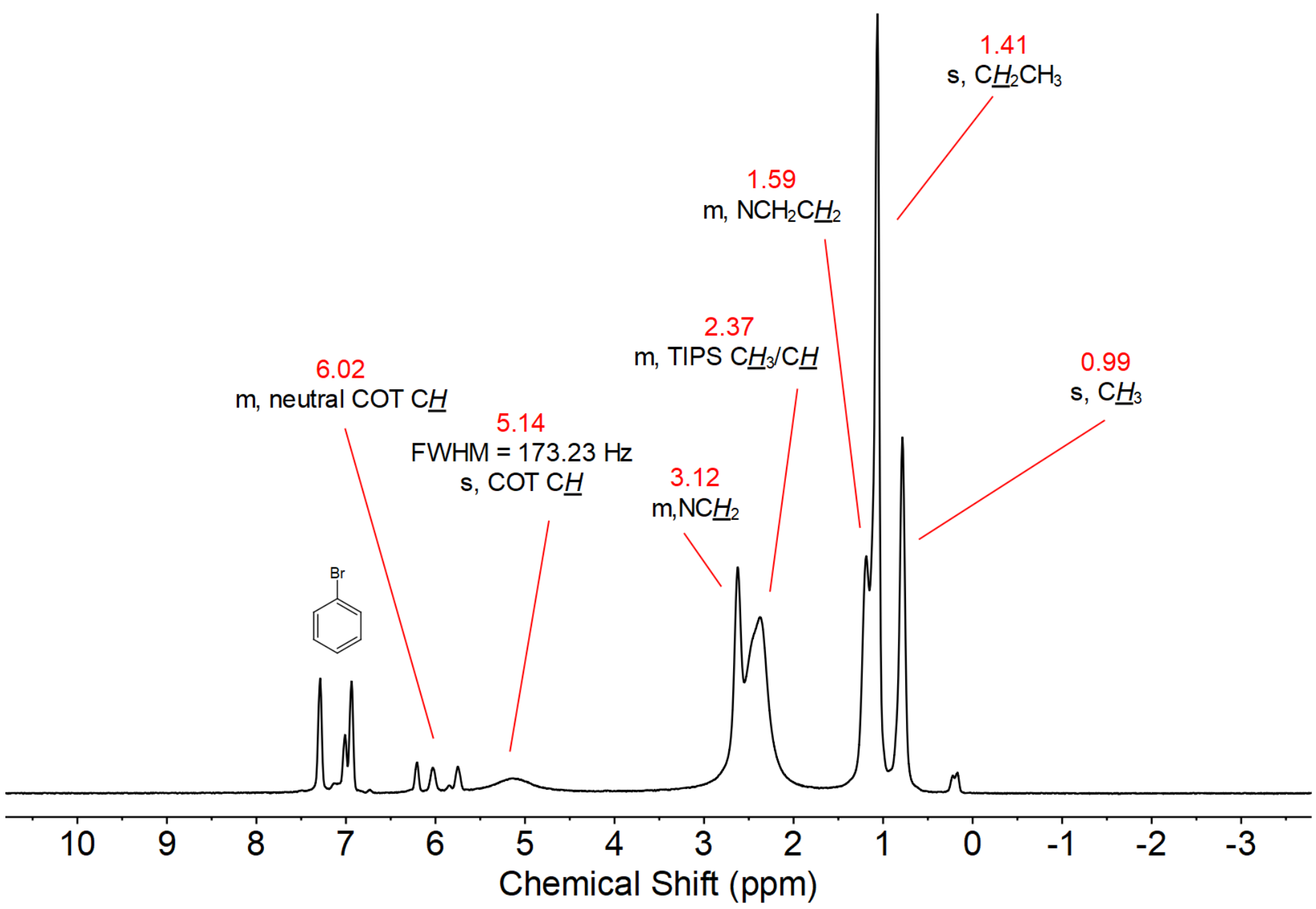

Figure S97. ^{1}H NMR spectrum of the crude reaction mixture between **6** and [*n*Bu$_4$N][SbF$_6$] in *d*$_5$-bromobenzene showing from +11 to –3 ppm.

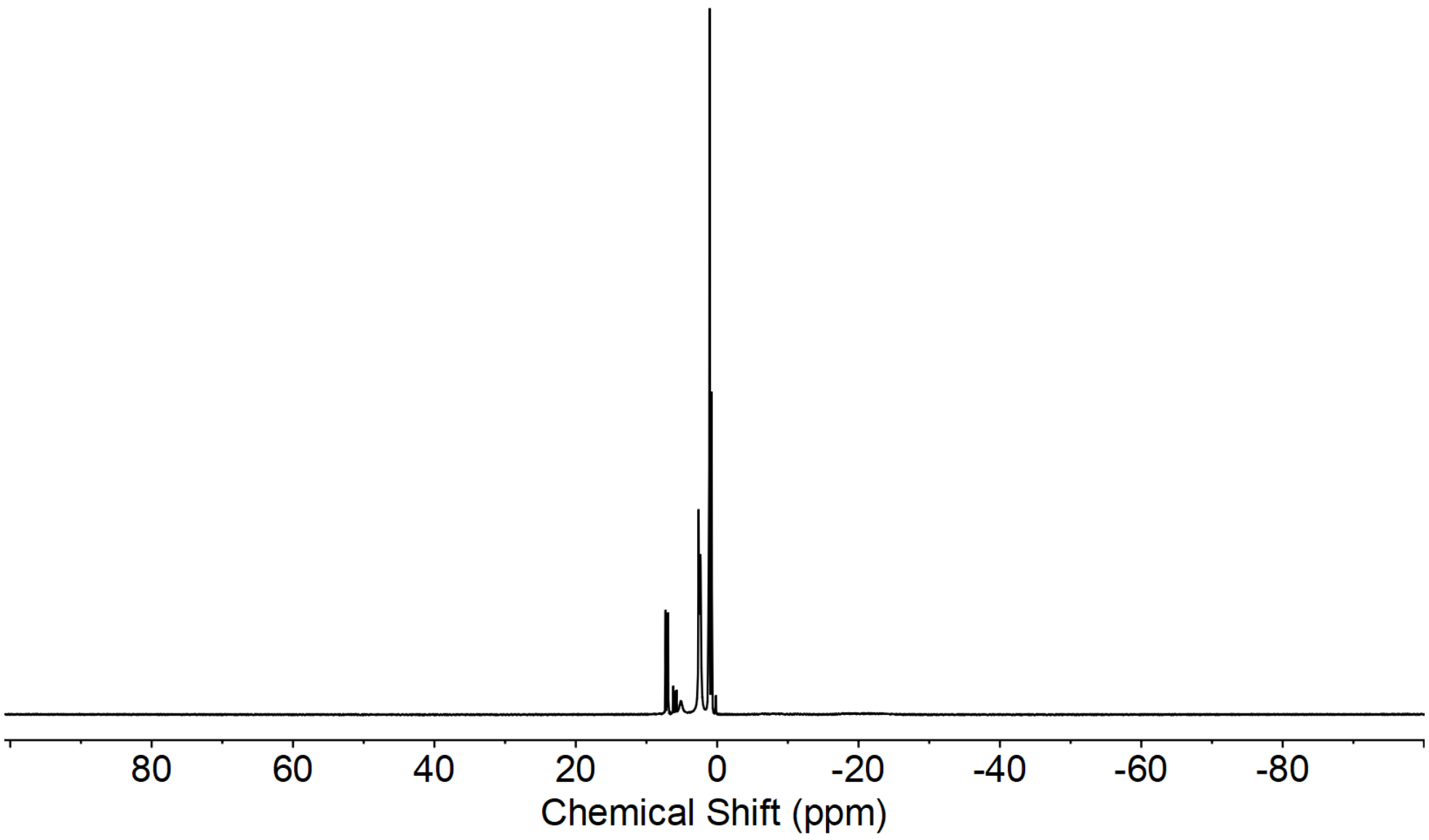

Figure S98. ^{1}H NMR spectrum of the crude reaction mixture between **6** and [nBu$_4$N][SbF$_6$] in d_5-bromobenzene showing from +11 to –3 ppm.

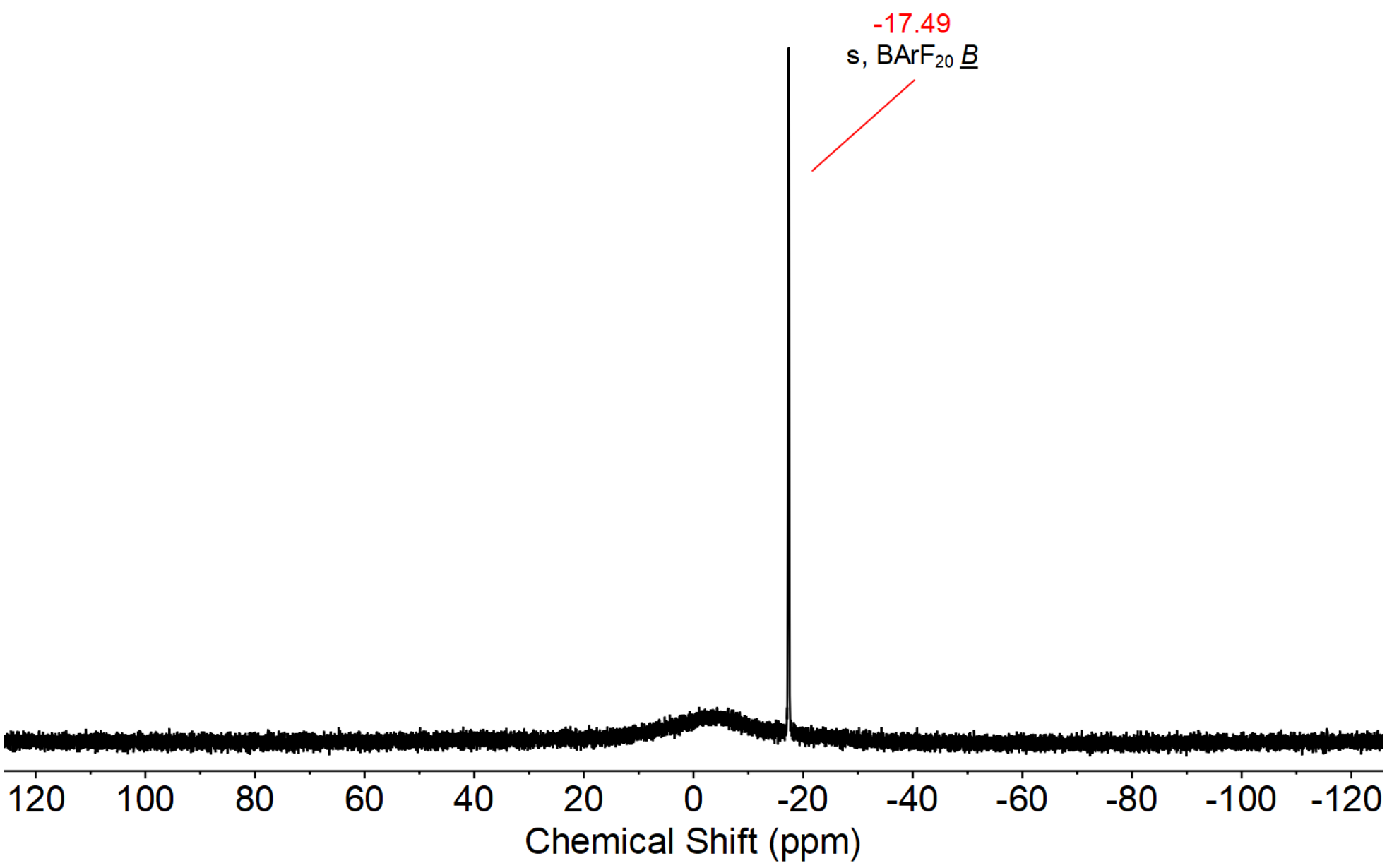

Figure S99. $^{11}B\{^{1}H\}$ NMR spectrum of the crude reaction mixture between **6** and [nBu$_4$N][SbF$_6$] in d_5-bromobenzene.

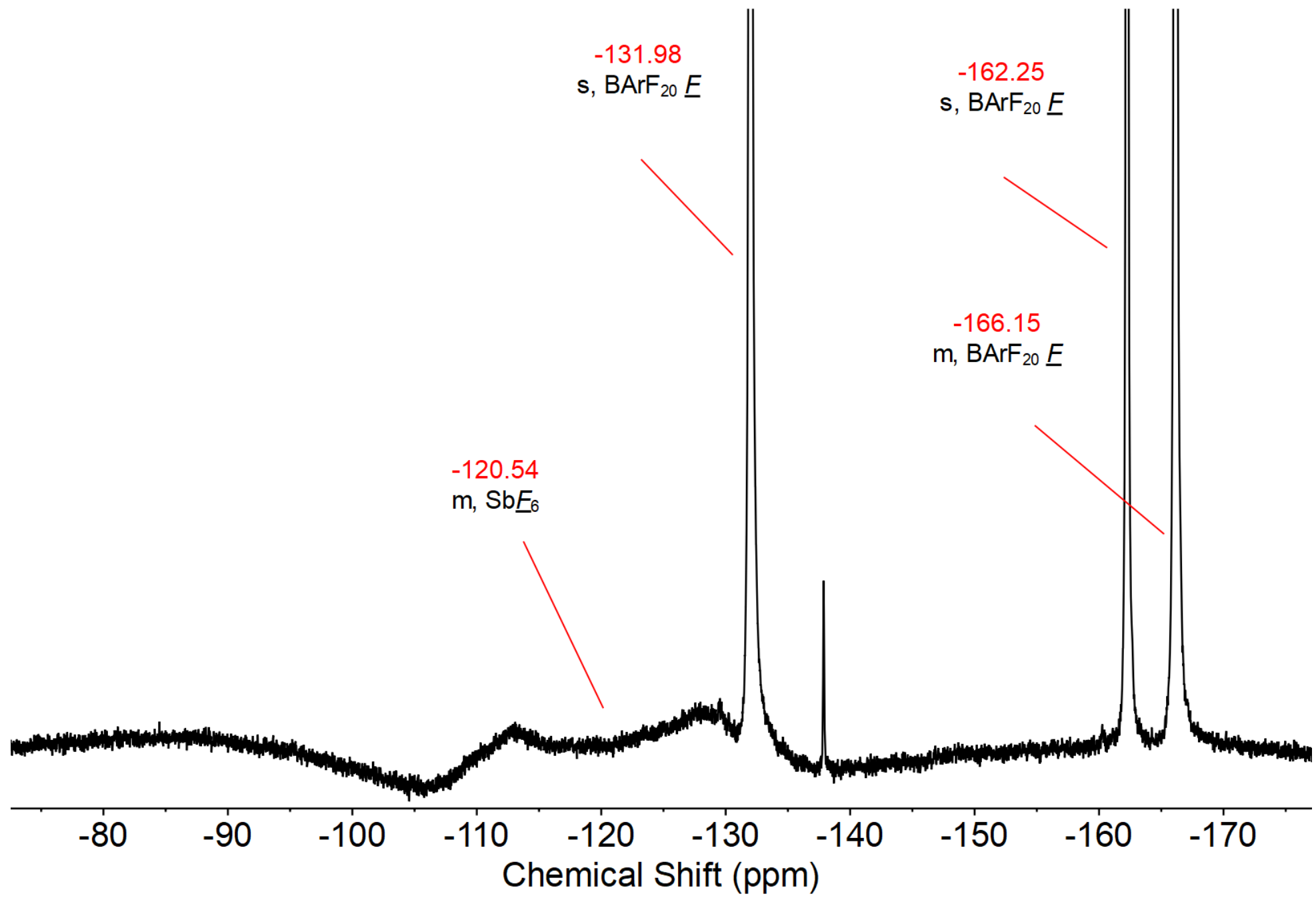

Figure S100. $^{19}F\{^{1}H\}$ NMR spectrum of the crude reaction mixture between **6** and [*n*Bu_4N][SbF_6] in d_5-bromobenzene.

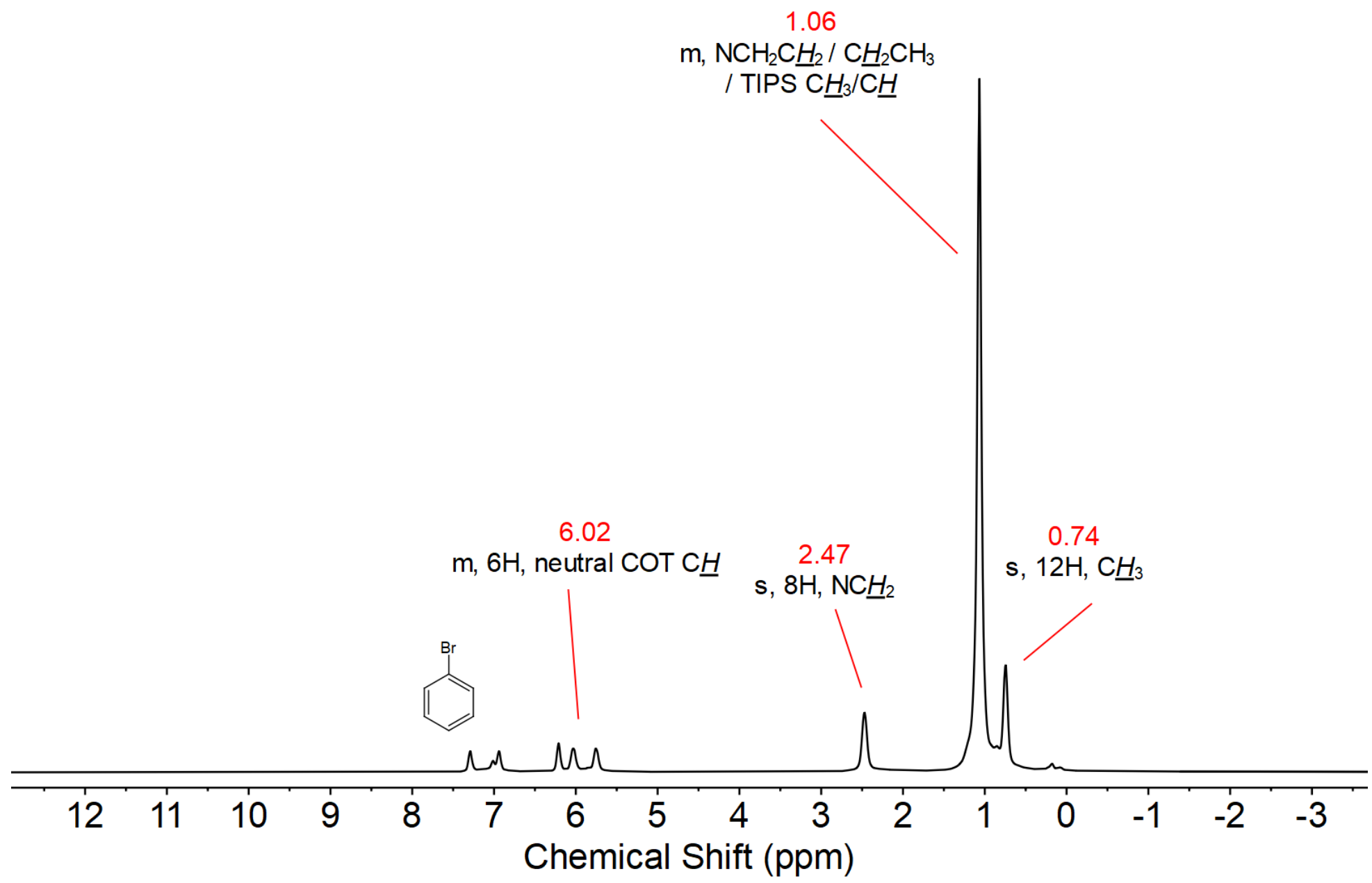

Figure S101. ^{1}H NMR spectrum of the crude reaction mixture between **6** and [*n*Bu_4N][SbF_6] in d_5-bromobenzene showing from +11 to –3 ppm after 24 hours at room temperature.

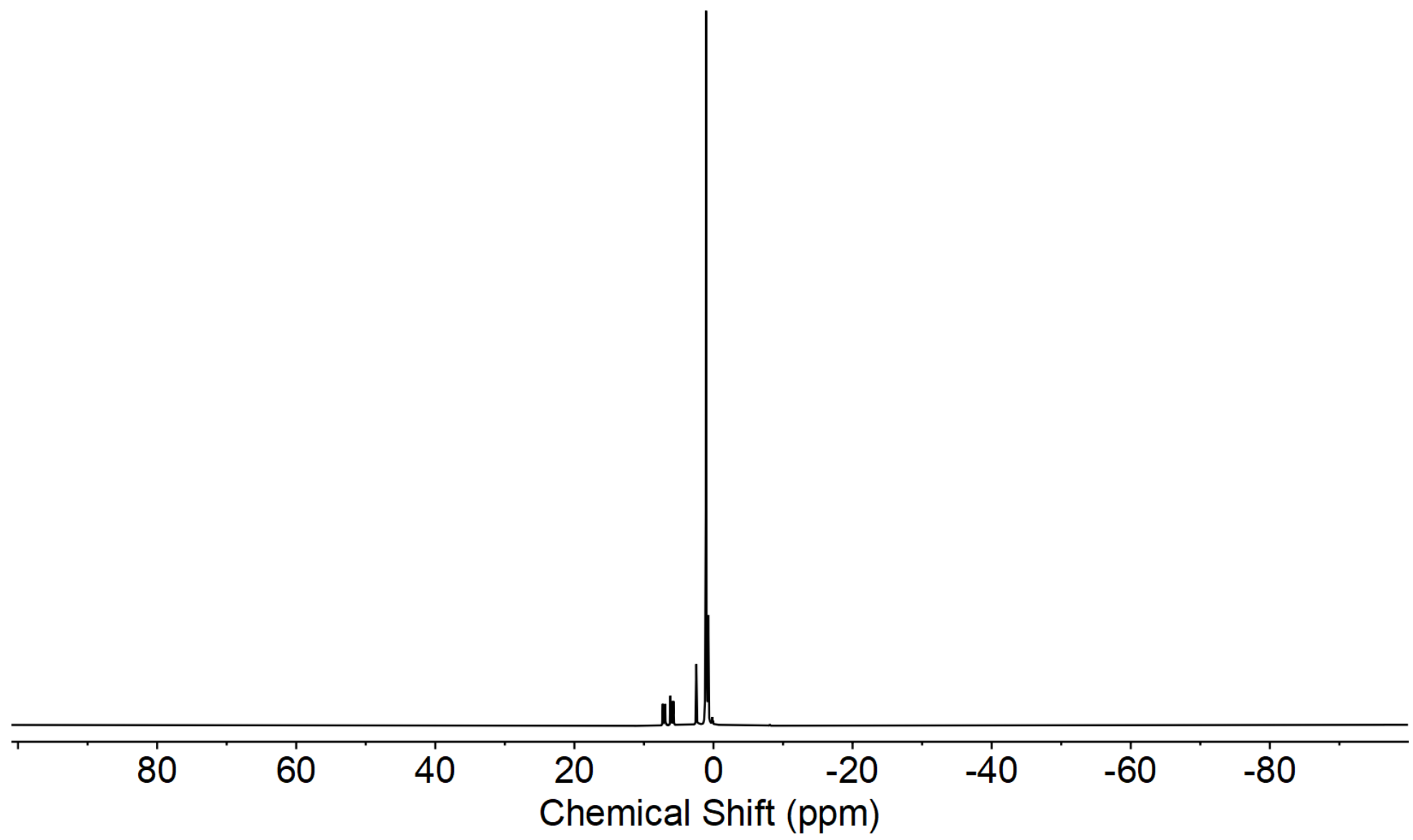

Figure S102. ^{1}H NMR spectrum of the crude reaction mixture between **6** and [nBu$_4$N][SbF$_6$] in d_5-bromobenzene showing from +11 to –3 ppm after 24 hours at room temperature.

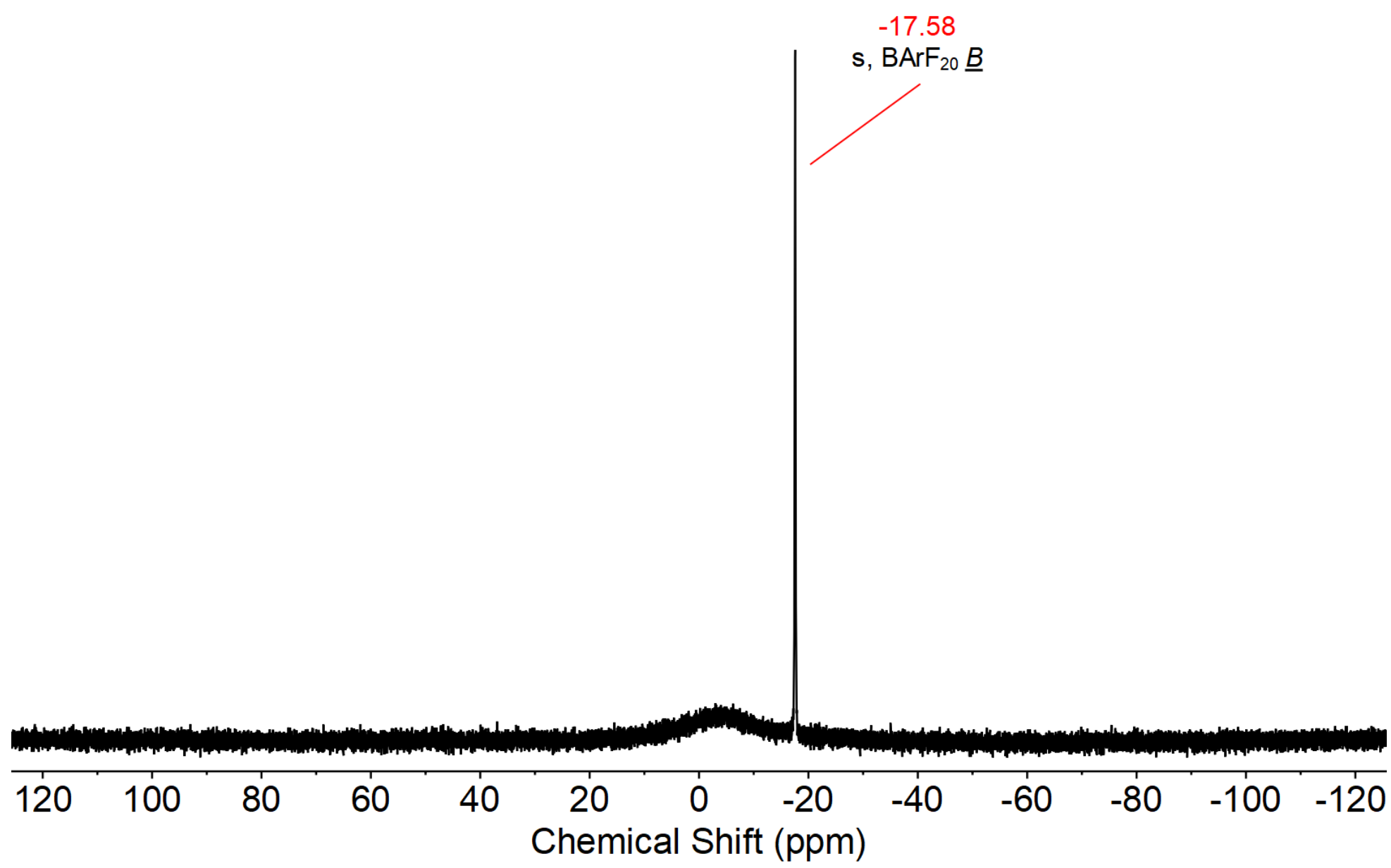

Figure S103. $^{11}B\{^{1}H\}$ NMR spectrum of the crude reaction mixture between **6** and [nBu$_4$N][SbF$_6$] in d_5-bromobenzene after 24 hours at room temperature.

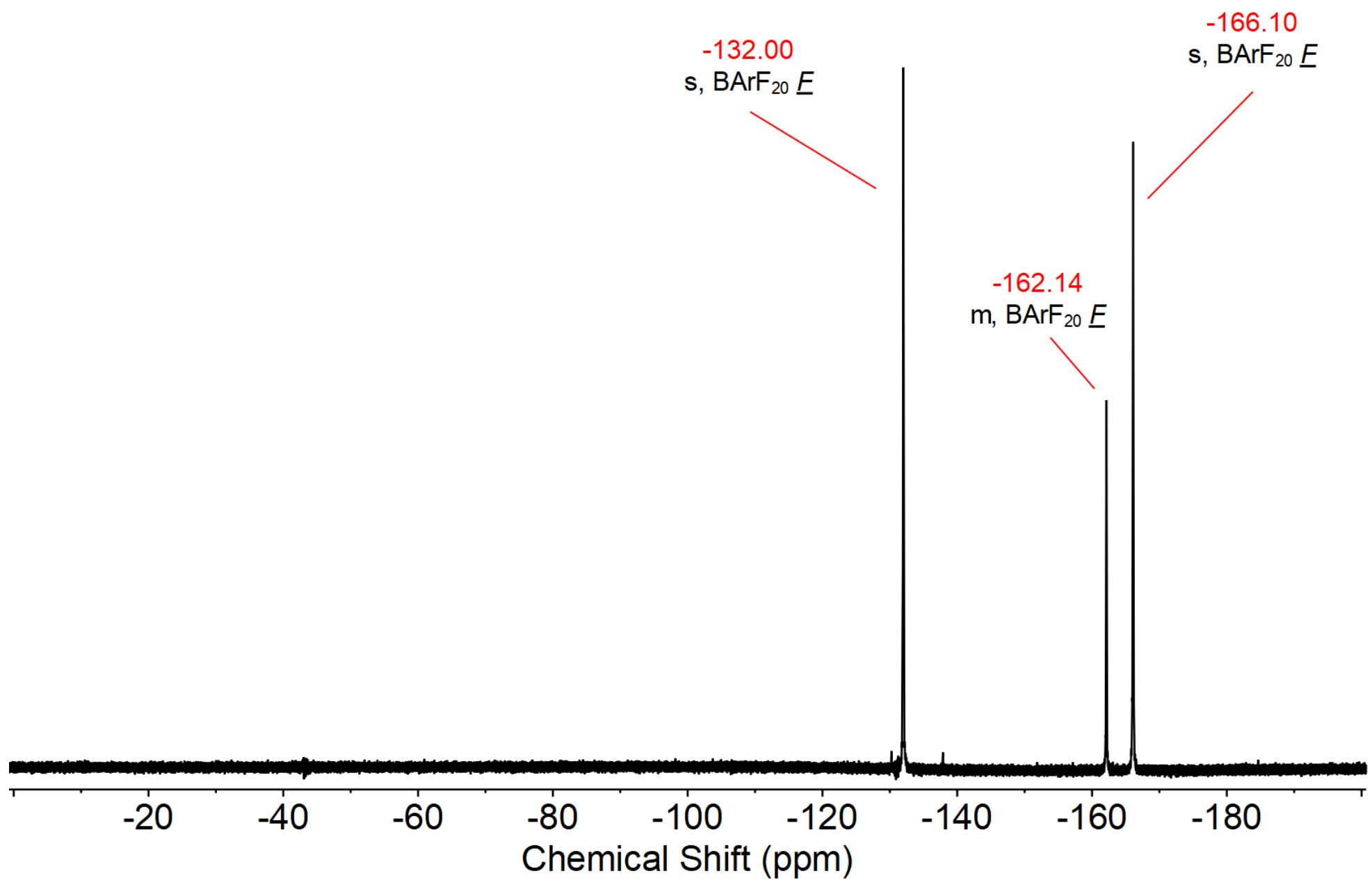

Figure S104. $^{19}F\{^{1}H\}$ NMR spectrum of the crude reaction mixture between **6** and [*n*Bu_4N][SbF_6] in d_5-bromobenzene after 24 hours at room temperature.

*The reaction between **3** and [Fc] in d_5-bromobenzene*

d_5-bromobenzene was added to a mixture of **3** (20 mg) and [Fc] (3.3 mg) in a J. Youngs appended NMR tube. No reaction was observed.

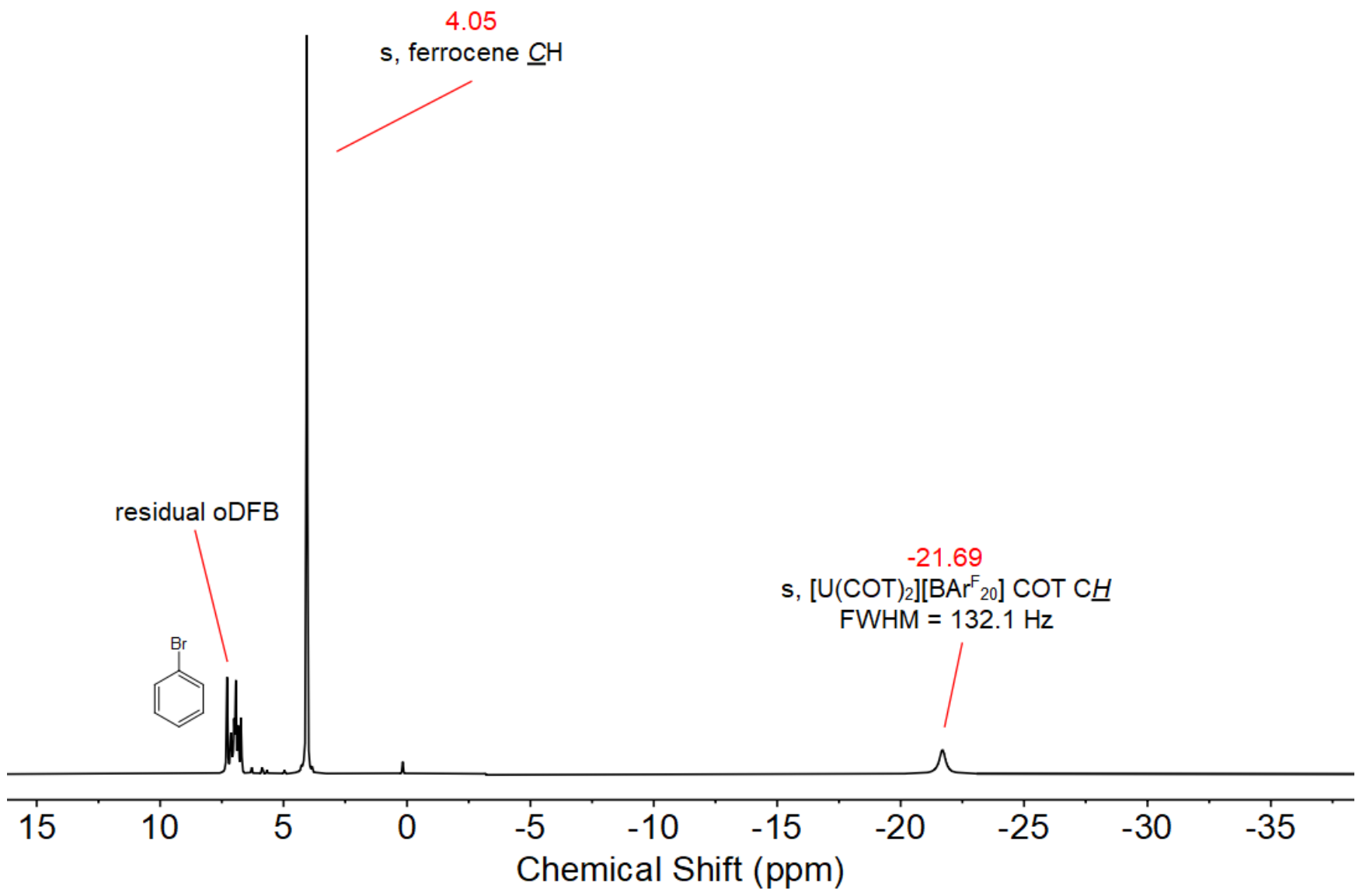

Figure S105. ¹H NMR spectrum of the crude reaction mixture between **3** and [Fc] in d_5-bromobenzene showing from +15 to –37 ppm.

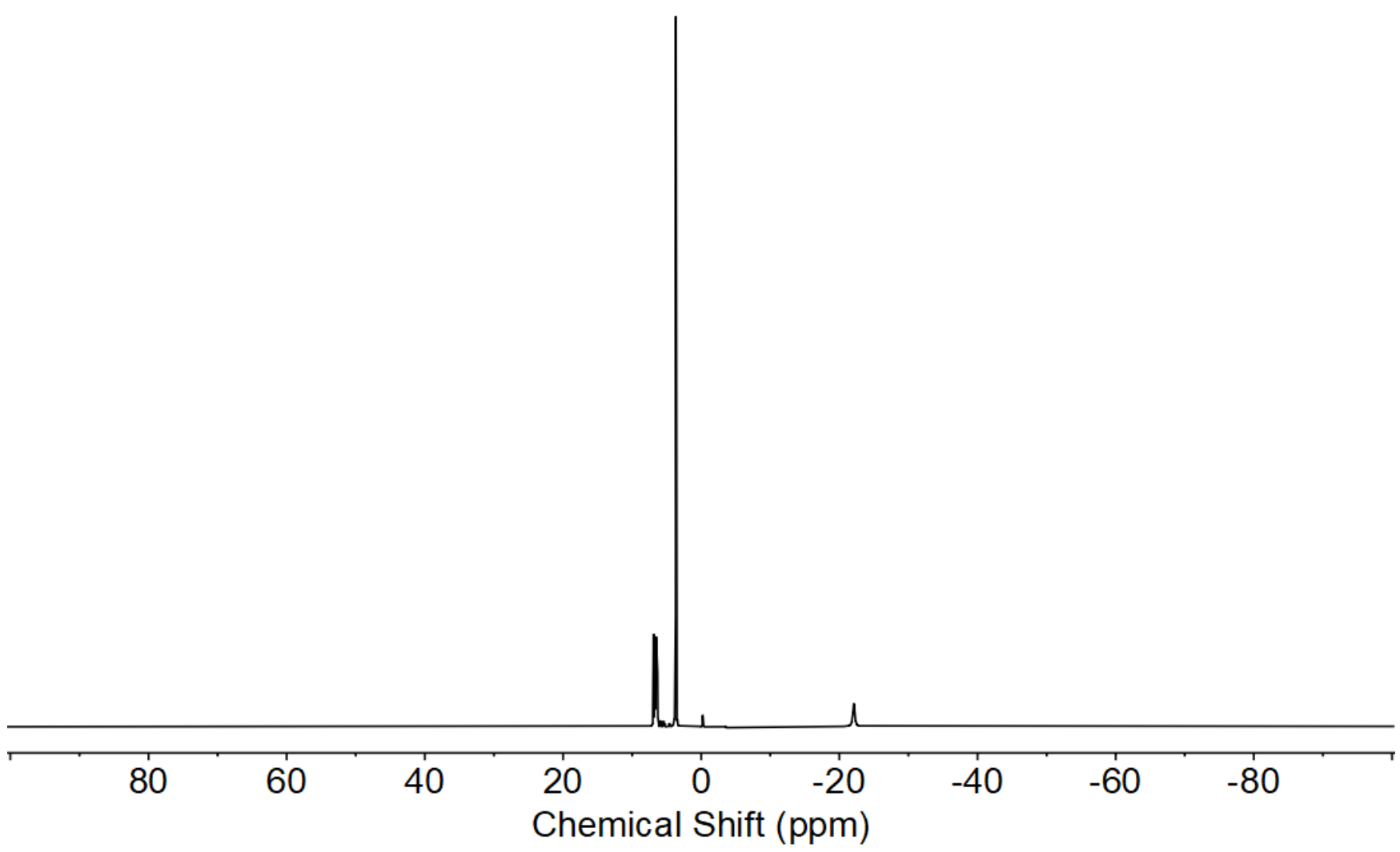

Figure S106. ¹H NMR spectrum of the crude reaction mixture between **3** and [Fc] in d_5-bromobenzene showing from +100 to –100 ppm.

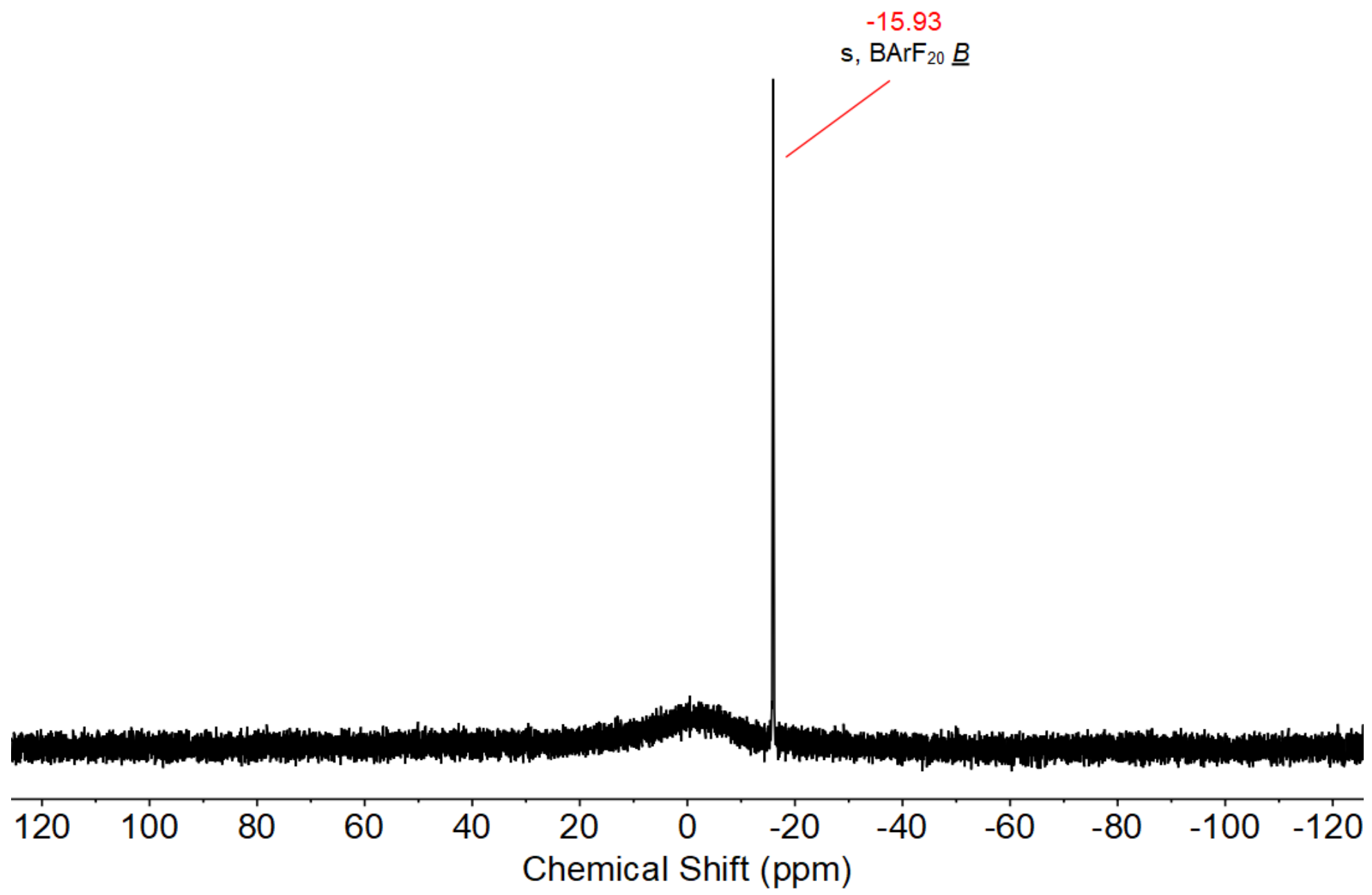

Figure S107. $^{11}B\{^1H\}$ NMR spectrum of the crude reaction mixture between **3** and [Fc] in *d*$_5$-bromobenzene.

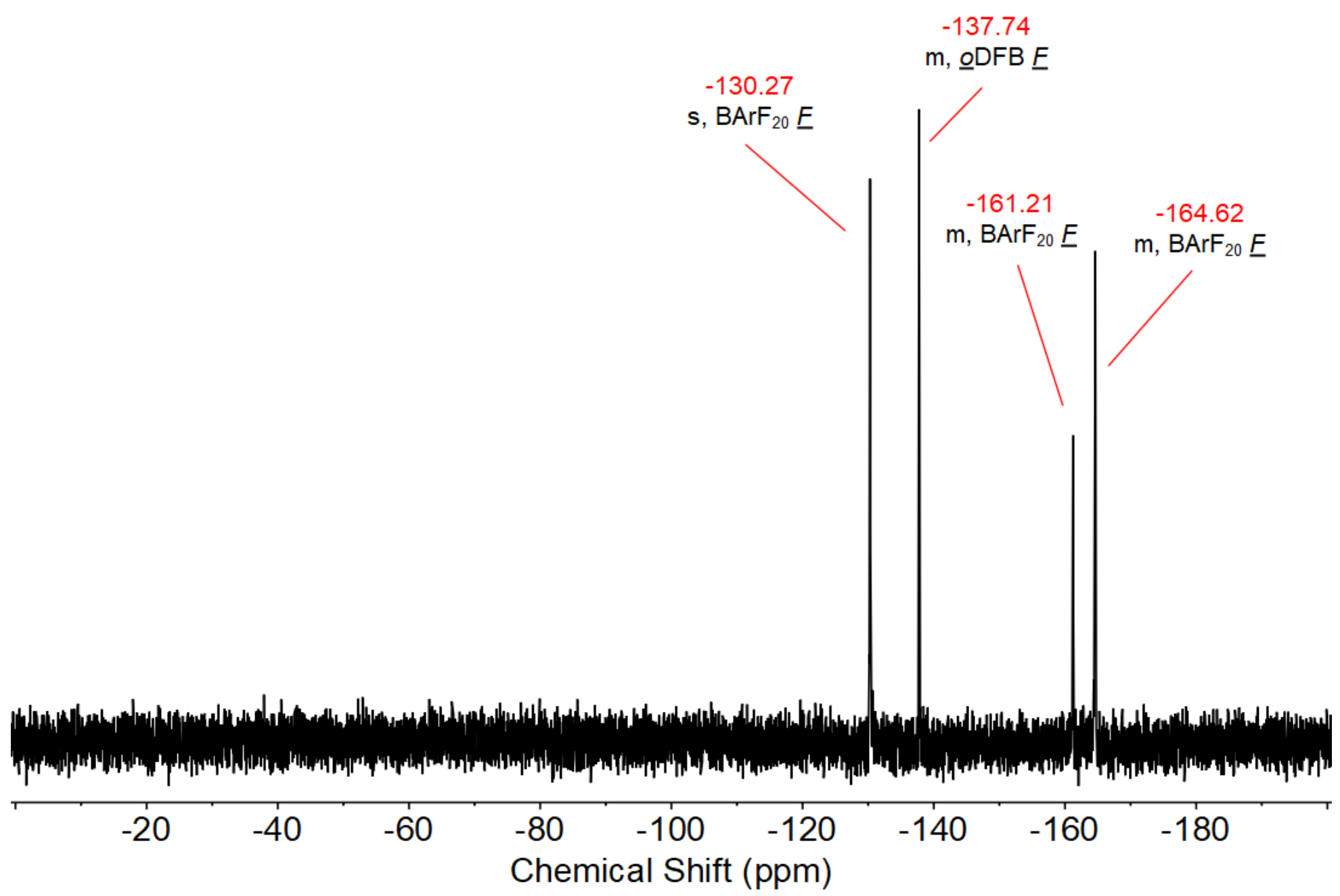

Figure S108. $^{19}F\{^1H\}$ NMR spectrum of the crude reaction mixture between **3** and [Fc] in *d*$_5$-bromobenzene.

*The reaction between **3** and [Fc*] in d_5-bromobenzene*

d_5-bromobenzene was added to a mixture of **3** (20 mg) and [Fc*] (5.8 mg) in a J. Youngs appended NMR tube resulting in an immediate colour change to dark green and the precipitation of **1** as dark green powder.

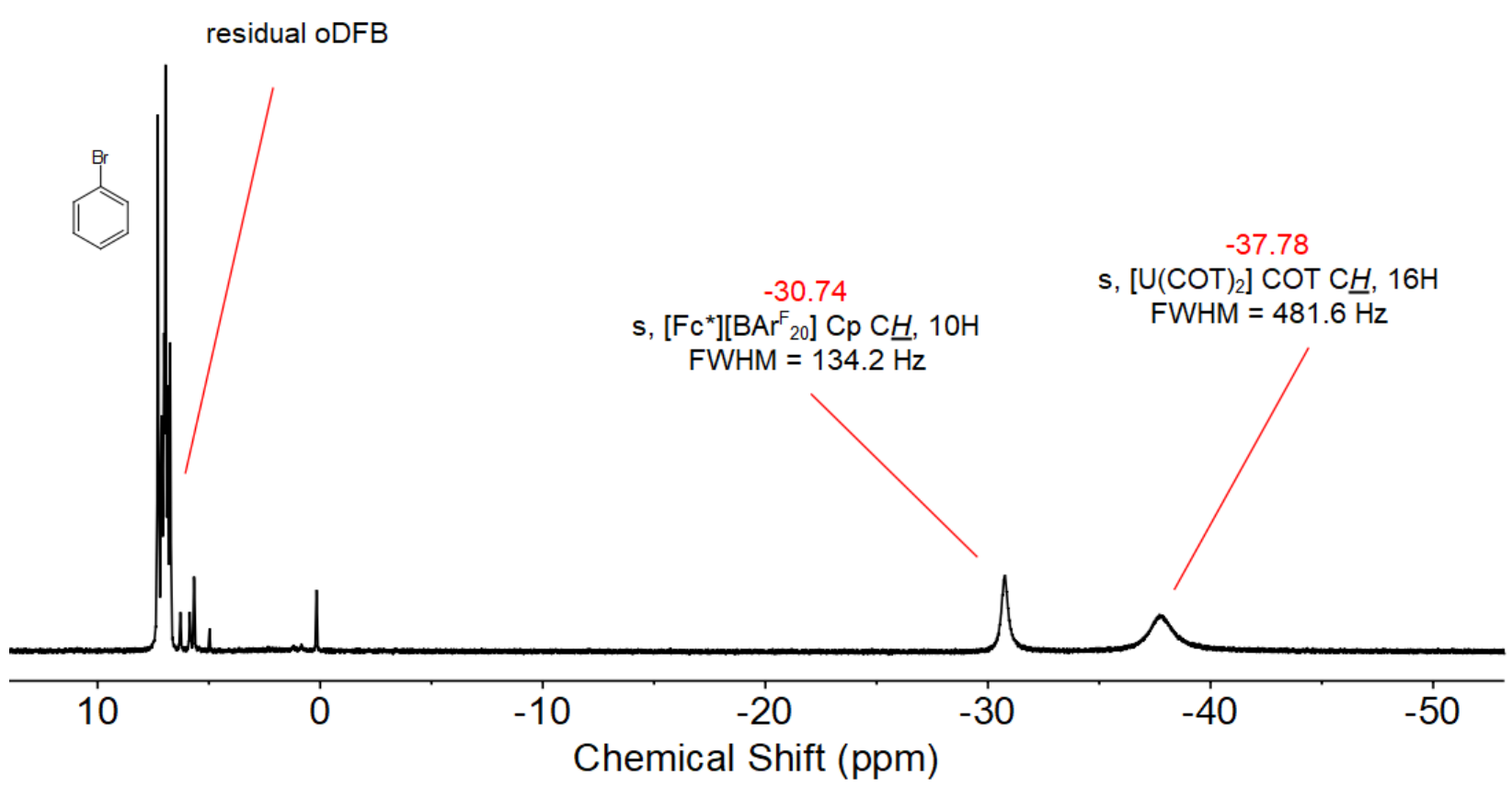

Figure S109. ^{1}H NMR spectrum of the crude reaction mixture between **3** and [Fc*] in d_5-bromobenzene yielding **1** and [Fc*][B(C_6F_5)$_4$] showing from +15 to –37 ppm.

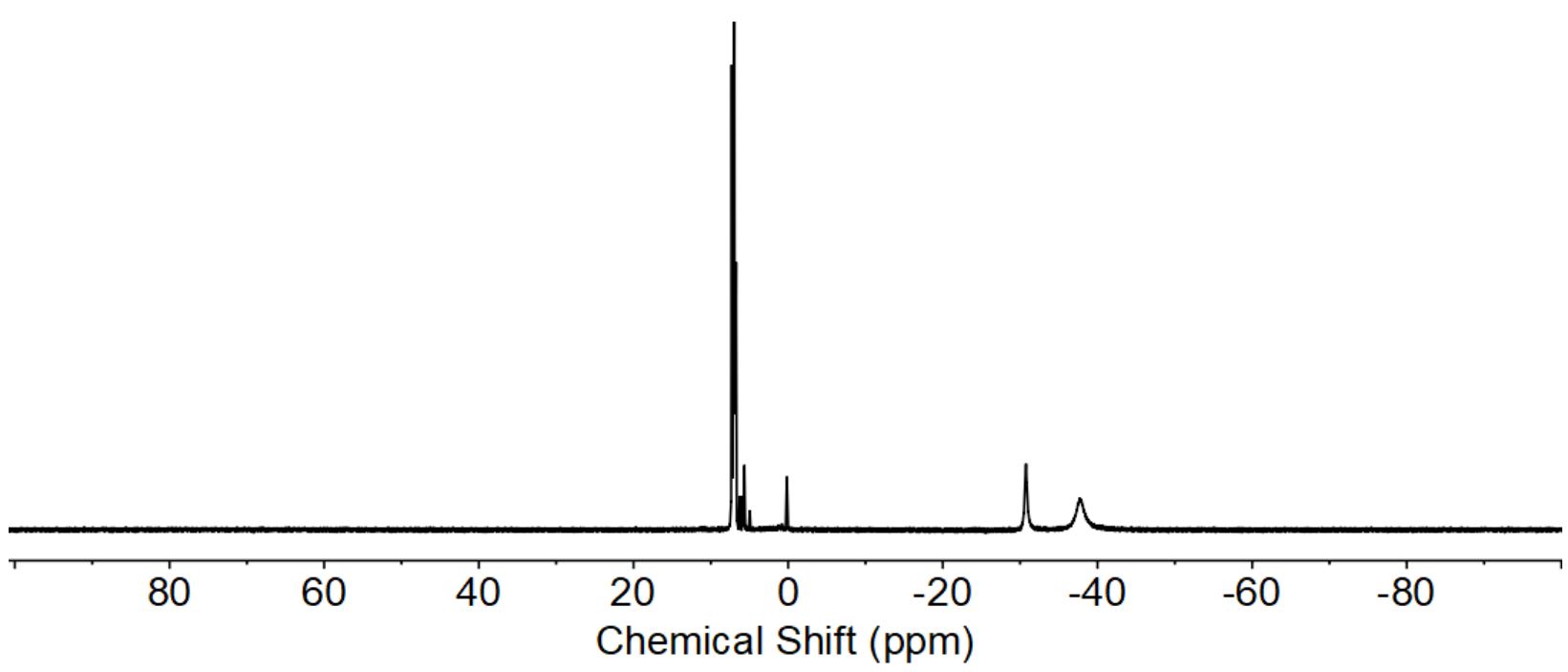

Figure S110. ^{1}H NMR spectrum of the crude reaction mixture between **3** and [Fc*] in *o*DFB yielding **1** and [Fc*][B(C_6F_5)$_4$] showing from +100 to –100 ppm.

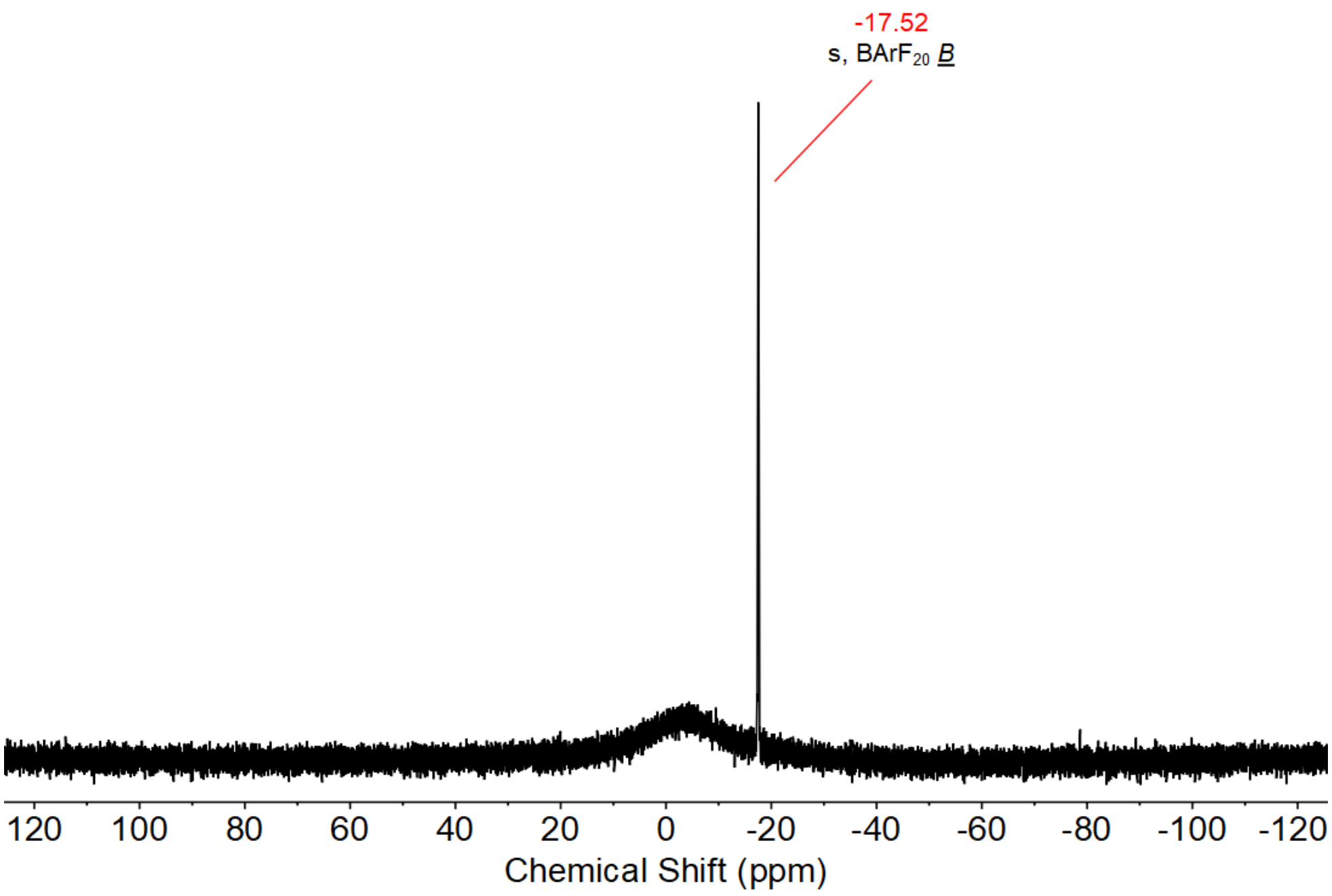

Figure S111. $^{11}B\{^1H\}$ NMR spectrum of the crude reaction mixture between **3** and [Fc*] in d_5-bromobenzene yielding **1** and [Fc*][B(C_6F_5)$_4$].

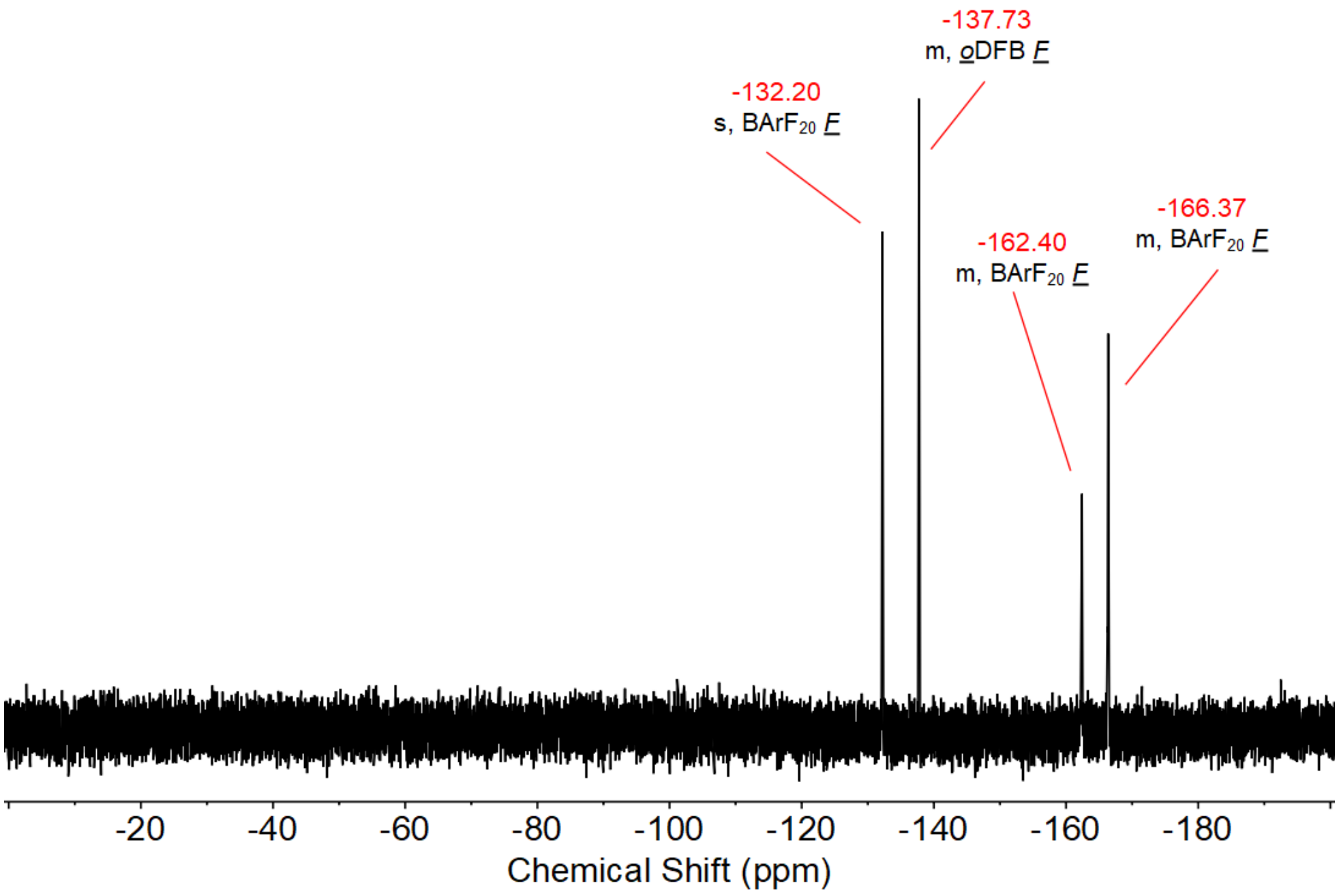

Figure S112. $^{19}F\{^1H\}$ NMR spectrum of the crude reaction mixture between **3** and [Fc*] in d_5-bromobenzene yielding **1** and [Fc*][B(C_6F_5)$_4$].

S5. Solution phase UV-Vis-NIR spectroscopy

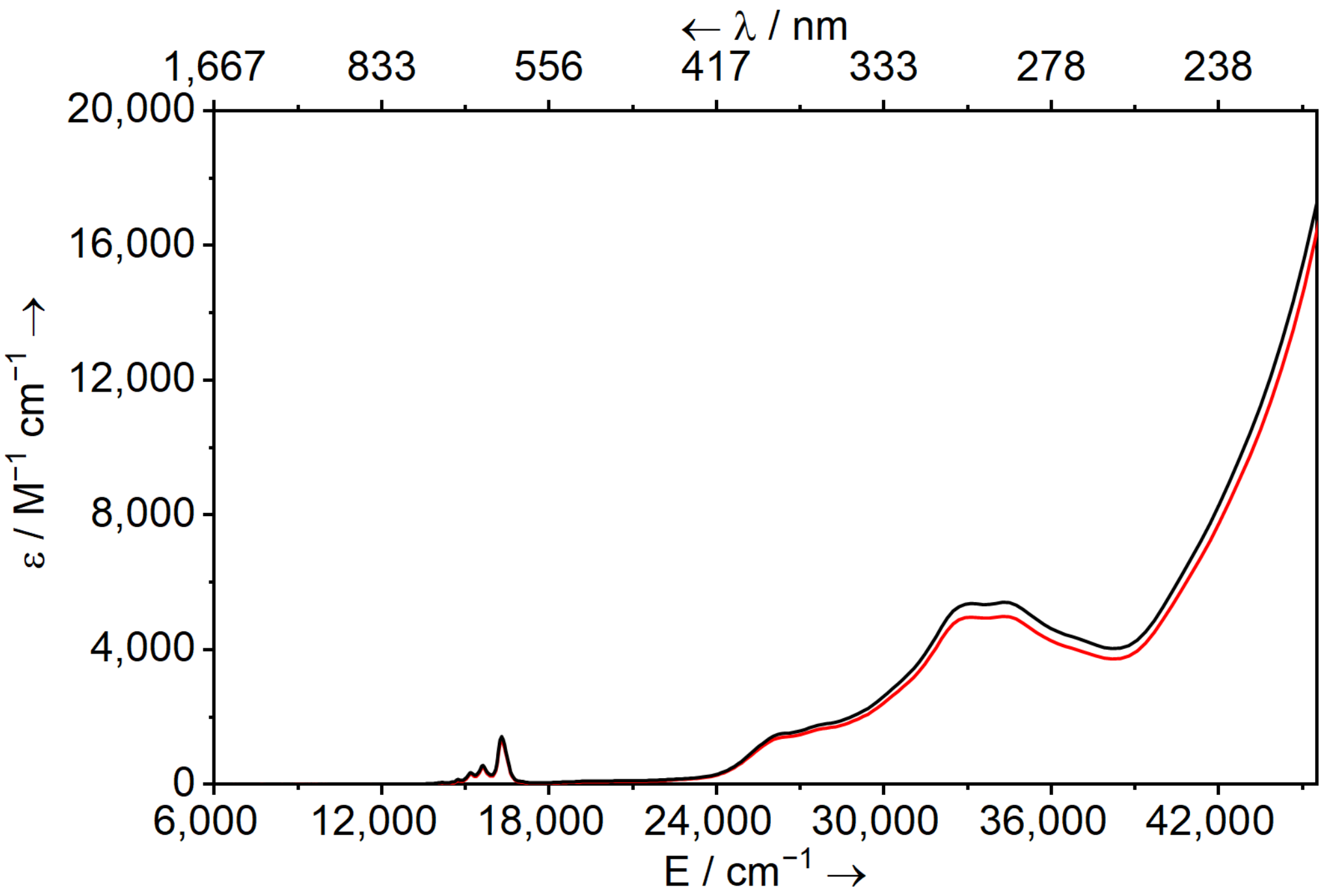

Figure S113. Solution UV-Vis-NIR spectrum of **1** (0.40 mM, black; 0.20 mM, red) in THF shown between 6,000–45,500 cm^{-1} (1,667–220 nm) at ambient temperature.

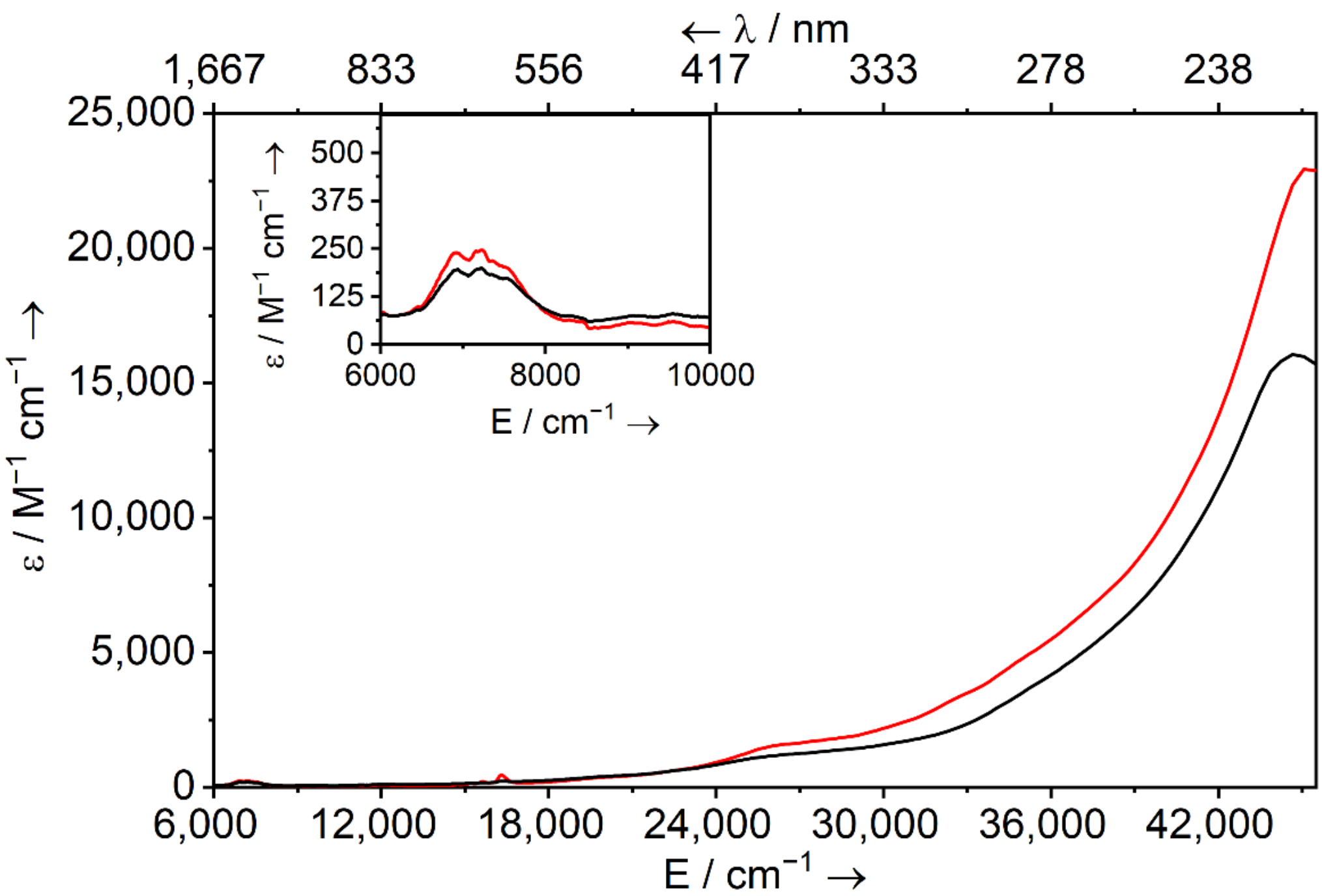

Figure S114. Solution UV-Vis-NIR spectrum of **2** (0.25 mM, black; 0.16 mM, red) in THF shown between 6,000–45,500 cm^{-1} (1,667–220 nm) at ambient temperature.

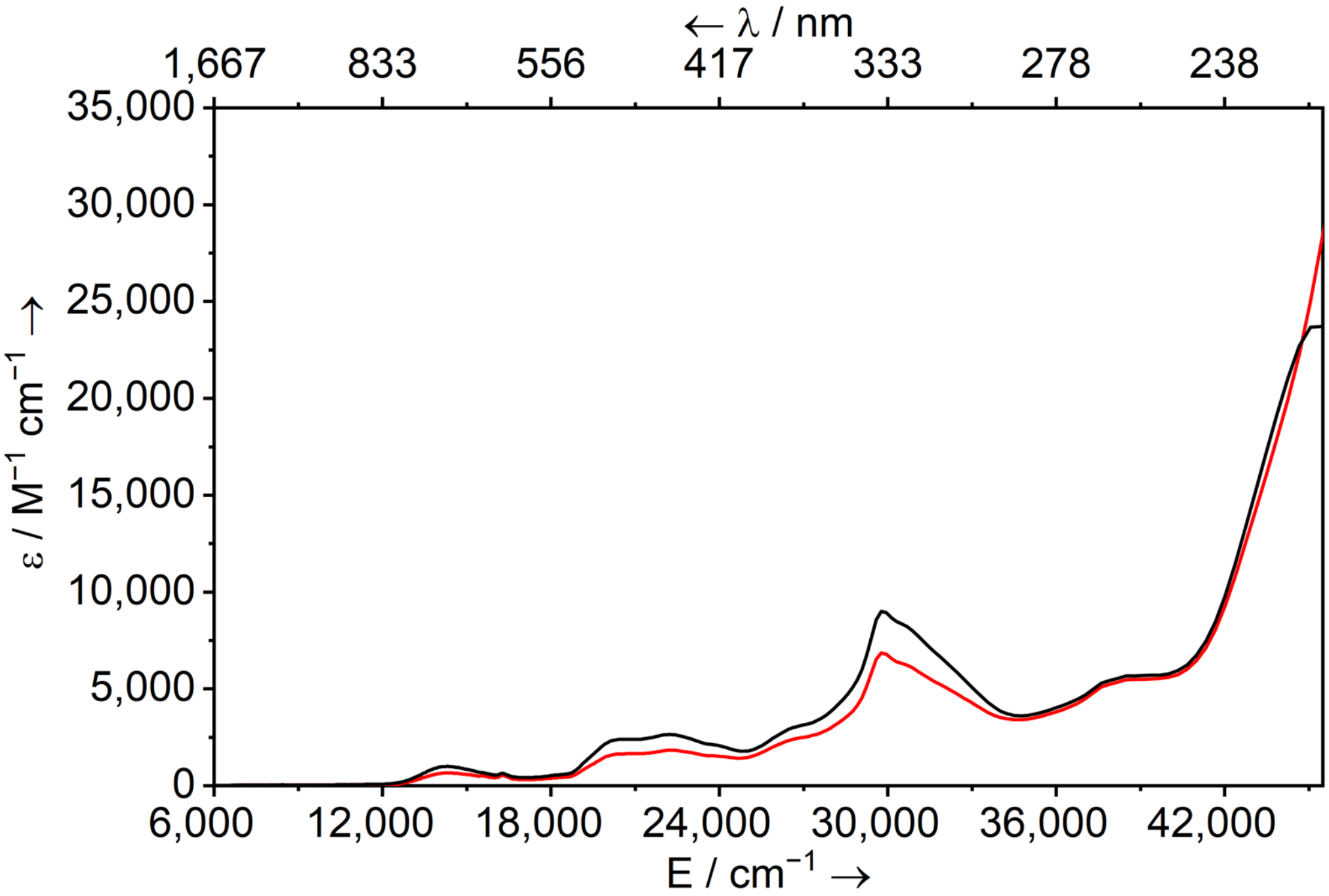

Figure S115. Solution UV-Vis-NIR spectrum of **3** (0.15 mM, black; 0.076 mM, red) in Et_2O shown between 6,000–45,500 cm^{-1} (1,667–220 nm) at ambient temperature.

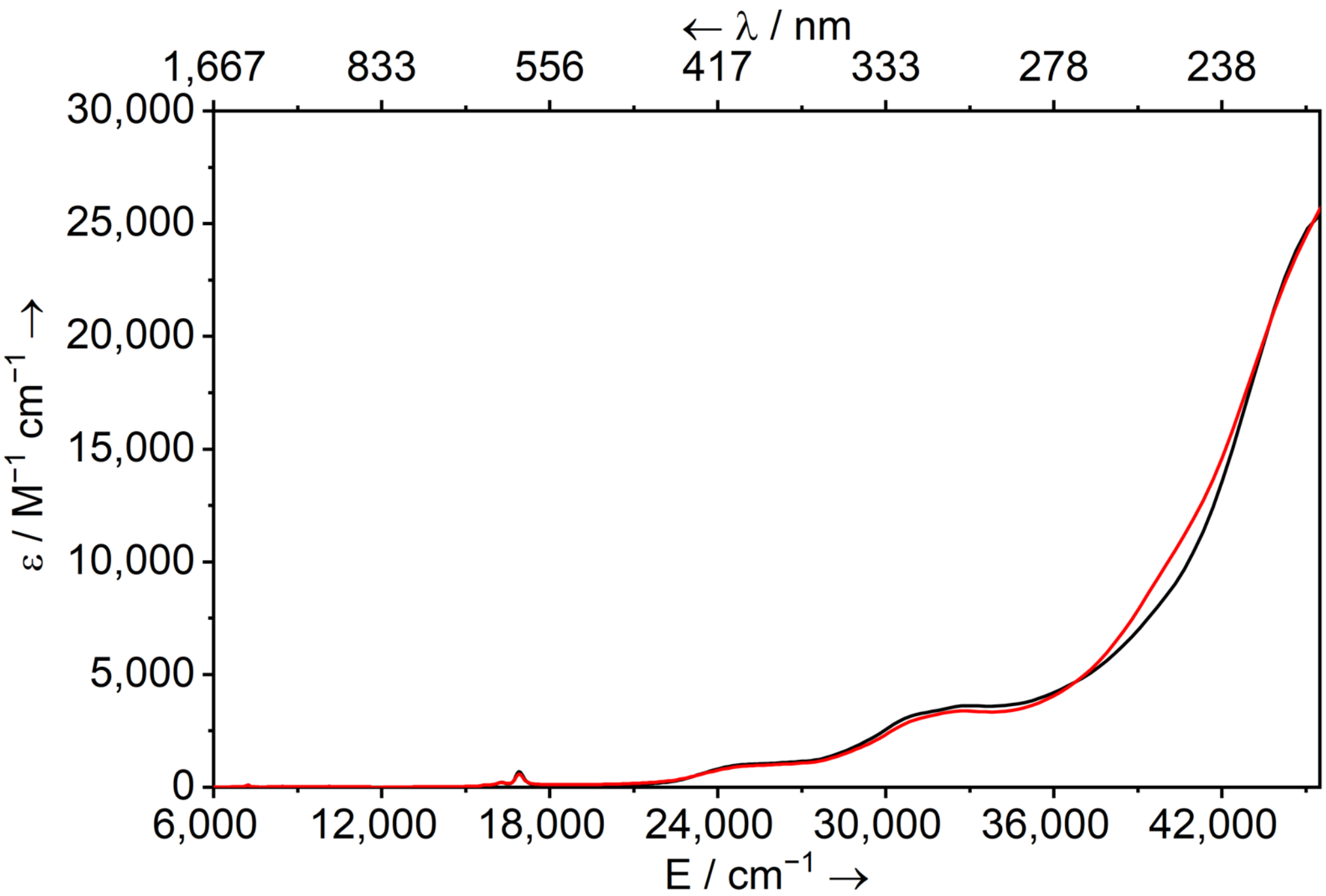

Figure S116. Solution UV-Vis-NIR spectrum of **4** (0.25 mM, black; 0.15 mM, red) in THF shown between 6,000–45,500 cm^{-1} (1,667–220 nm) at ambient temperature.

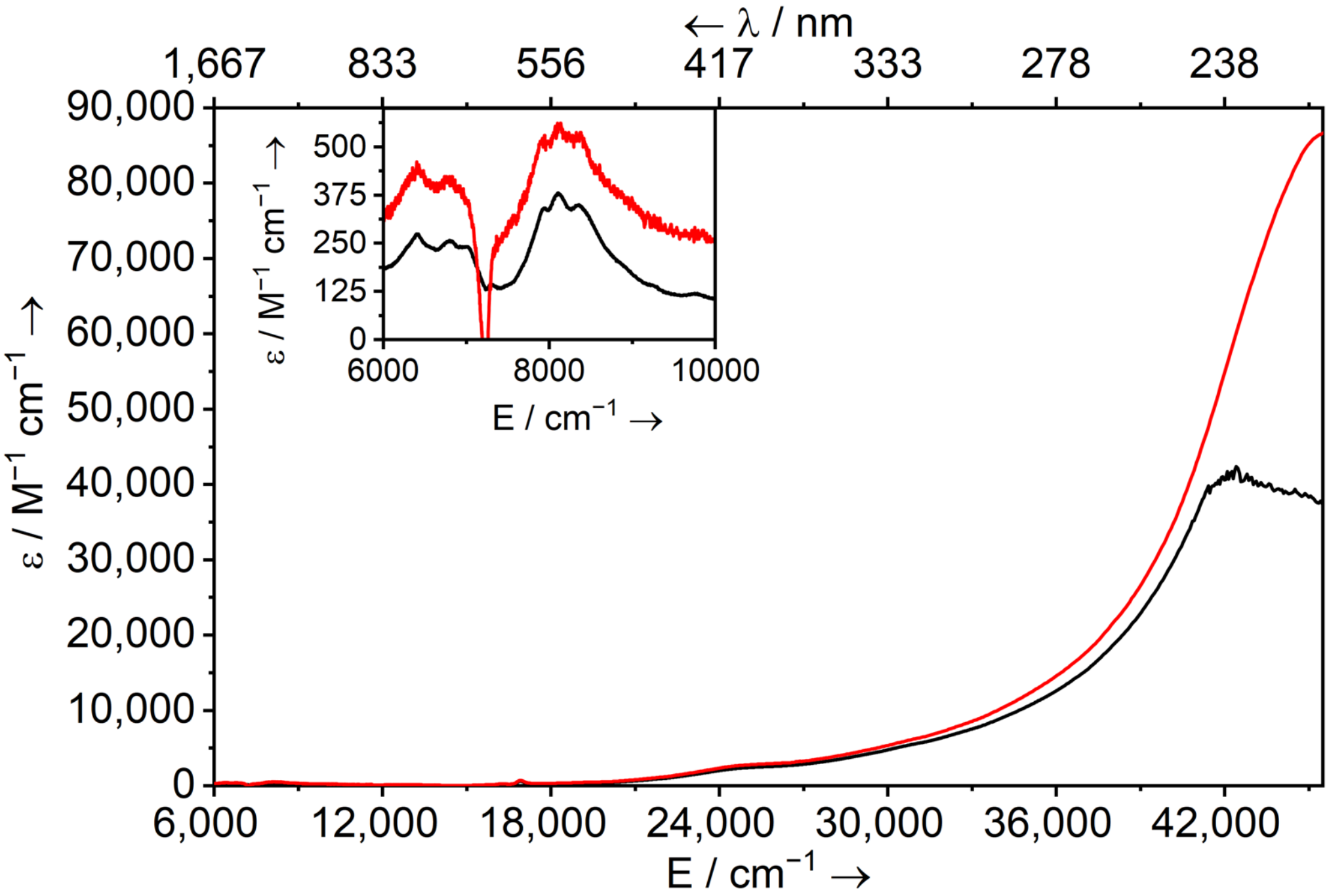

Figure S117. Solution UV-Vis-NIR spectrum of **5** (0.25 mM, black; 0.033 mM, red) in THF shown between 6,000–45,500 cm^{-1} (1,667–220 nm) at ambient temperature.

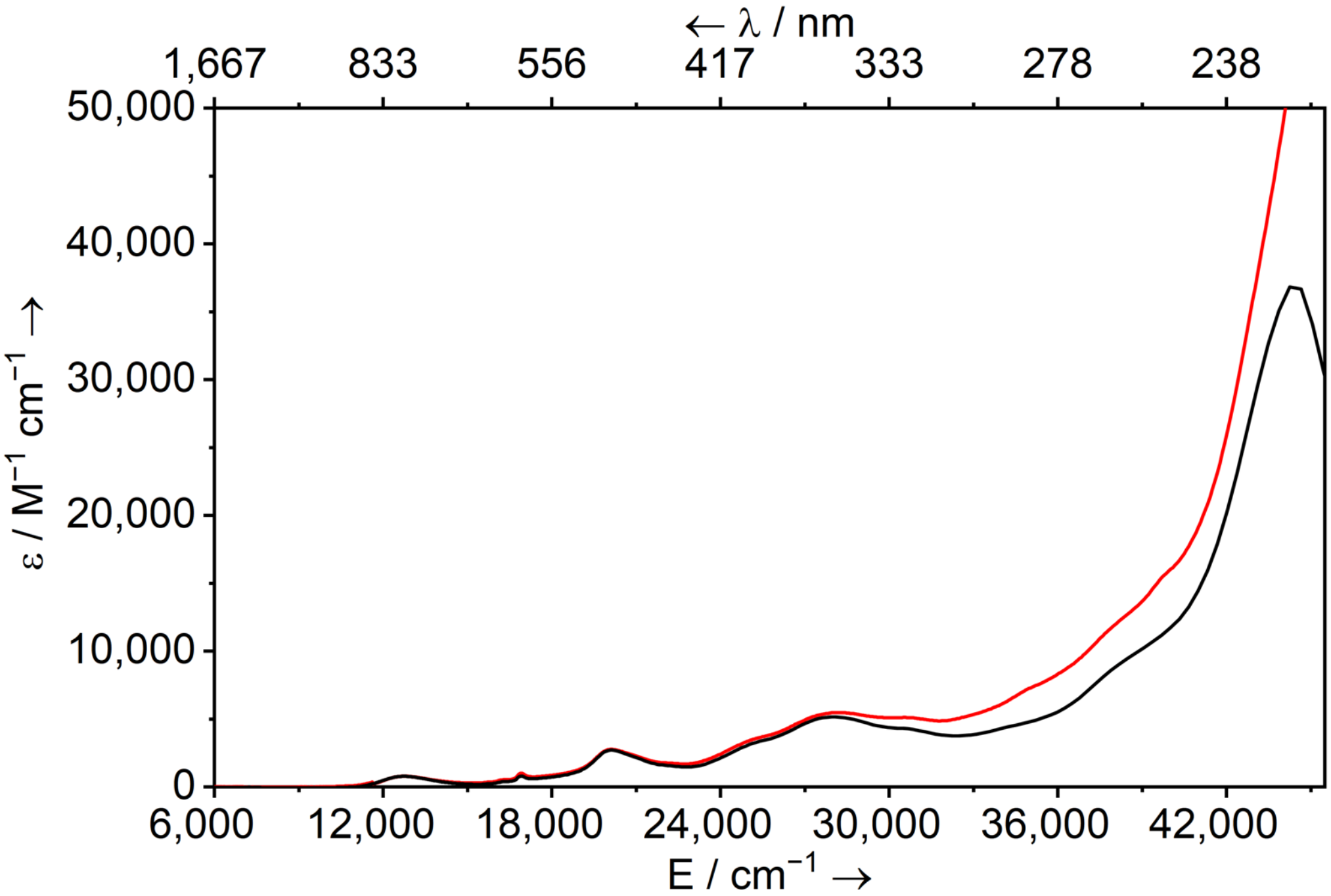

Figure S118. Solution UV-Vis-NIR spectrum of **6** (0.28 mM, black; 0.090 mM, red) in Et_2O shown between 6,000–45,500 cm^{-1} (1,667–220 nm) at ambient temperature.

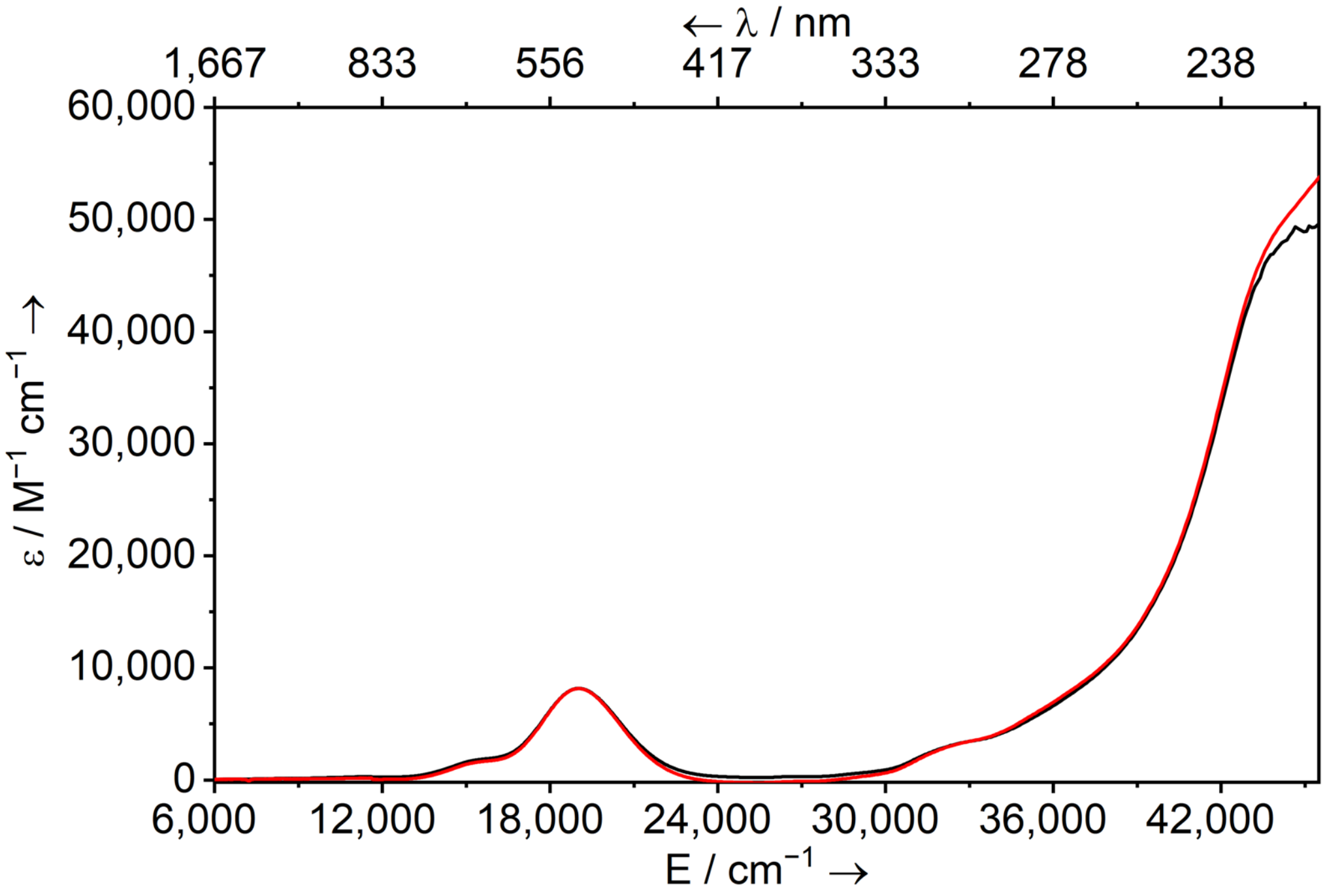

Figure S119. Solution UV-Vis-NIR spectrum of **9** (0.20 mM, black; 0.0622 mM, red) in THF shown between 6,000–45,500 cm^{-1} (1,667–220 nm) at ambient temperature.

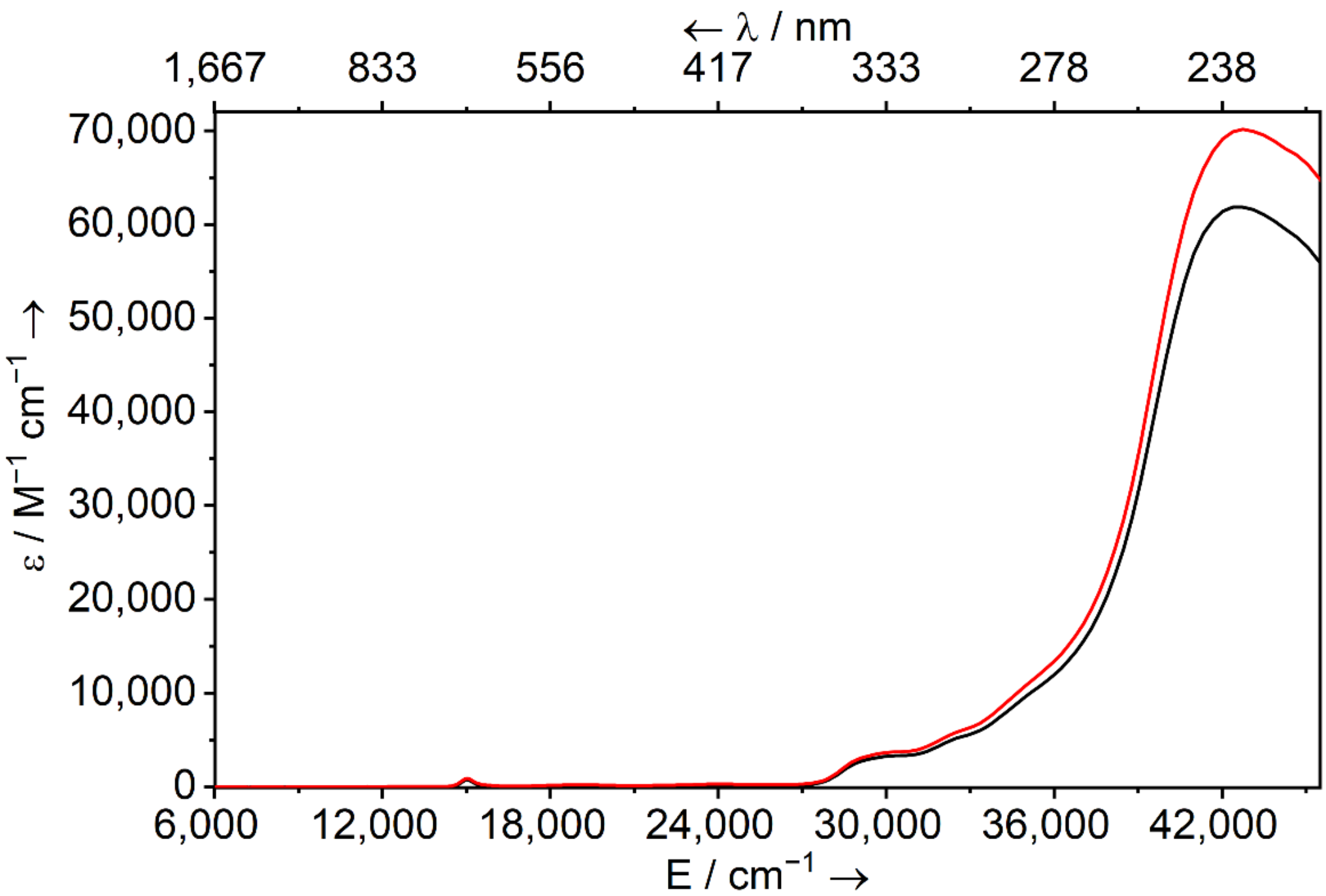

Figure S120. Solution UV-Vis-NIR spectrum of **10** (0.25 mM, black; 0.0625 mM, red) in THF shown between 6,000–45,500 cm^{-1} (1,667–220 nm) at ambient temperature.

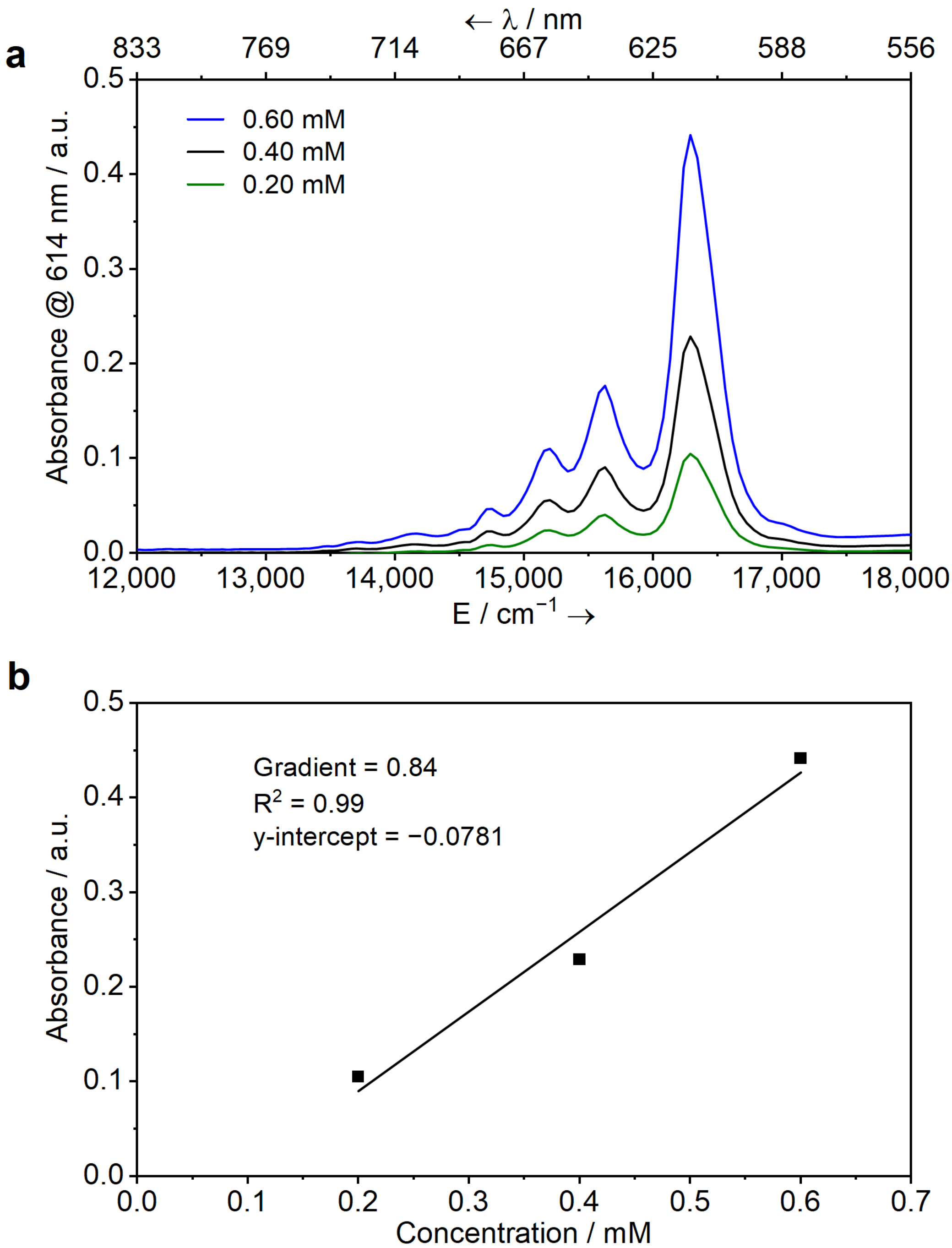

Figure S121. (**a**) Solution UV-Vis-NIR spectra of **1** at 0.20 mM, 0.40 mM and 0.60 mM in THF using a 0.4 cm path length cuvette shown between 12,000–18,000 cm^{-1} (833–556 nm) at ambient temperature. (**b**) Linear regression of absorbance at 16,287 cm^{-1} (614 nm) in **1** where ε = 2,100 M^{-1} cm^{-1}.

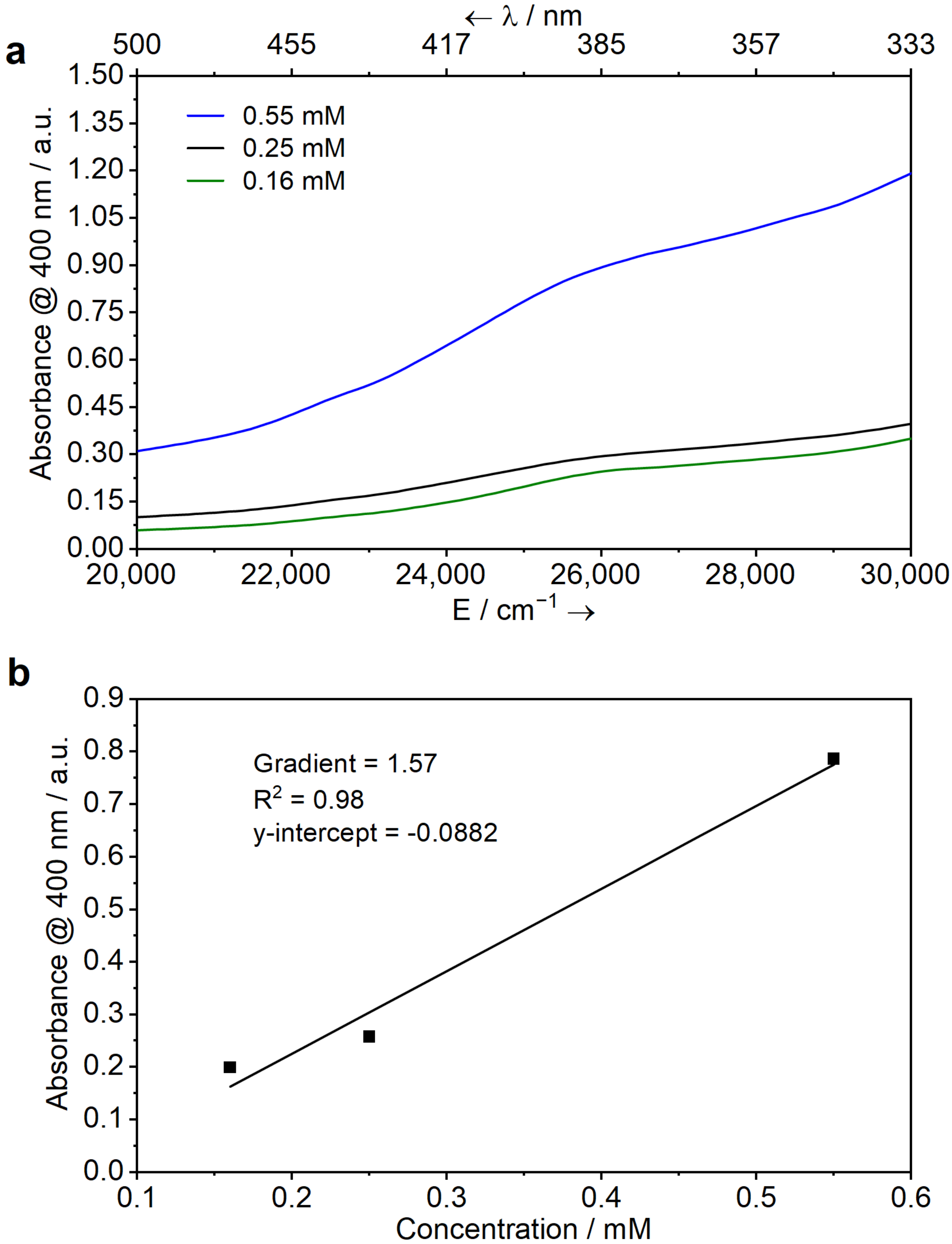

Figure S122. (**a**) Solution UV-Vis-NIR spectra of **2** at 0.16 mM, 0.25 mM and 0.55 mM in THF using a 1.0 cm path length cuvette shown between 20,000–30,000 cm^{-1} (500–333 nm) at ambient temperature. (**b**) Linear regression of absorbance at 25,000 cm^{-1} (400 nm) in **2** where ε = 1,570 M^{-1} cm^{-1}.

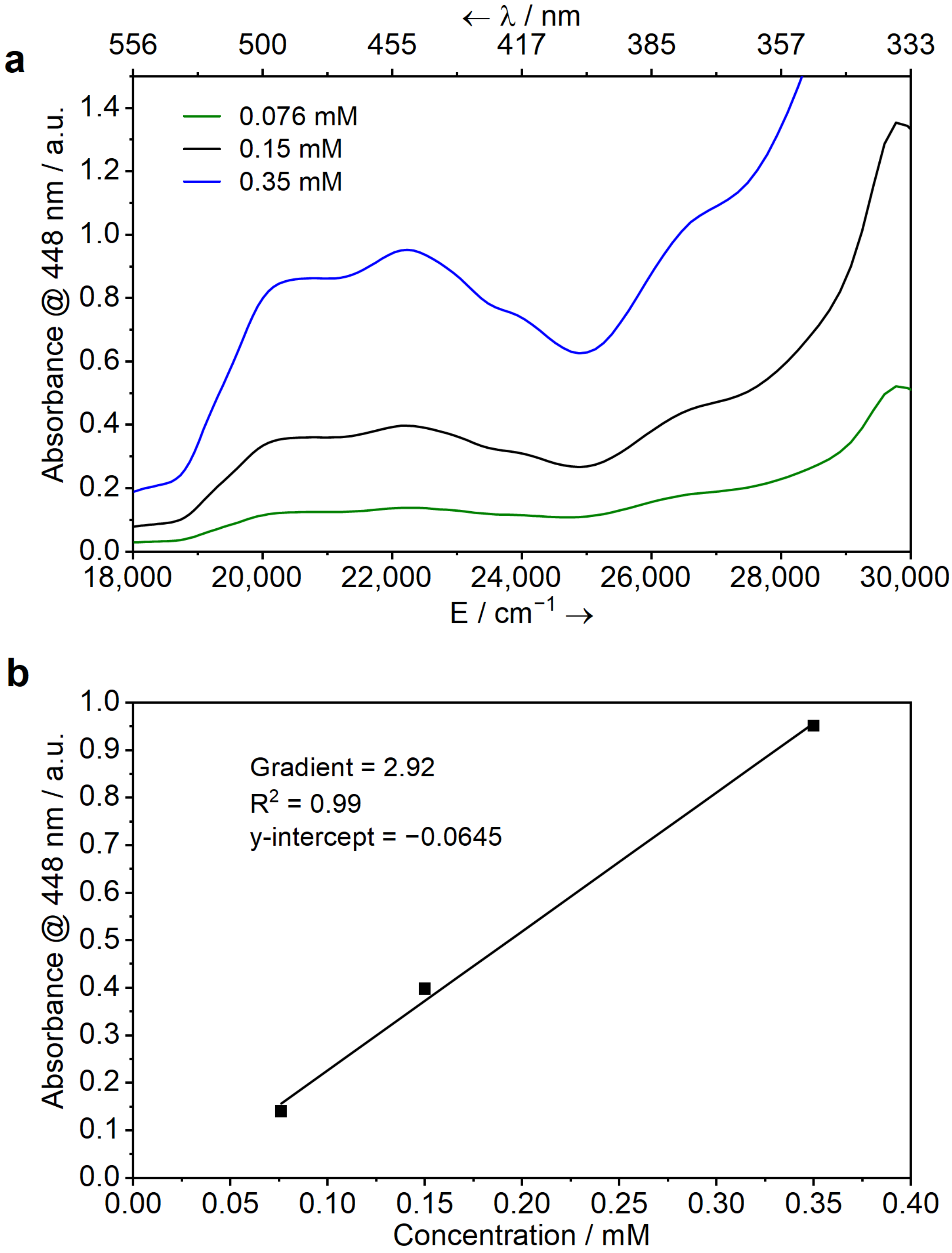

Figure S123. (**a**) Solution UV-Vis-NIR spectra of **3** at 0.076 mM, 0.15 mM and 0.35 mM in Et_2O using a 1 cm path length cuvette shown between 18,000–30,000 cm^{-1} (556–333 nm) at ambient temperature. (**b**) Linear regression of absorbance at 22,321 cm^{-1} (448 nm) in **3** where ε = 2,920 M^{-1} cm^{-1}.

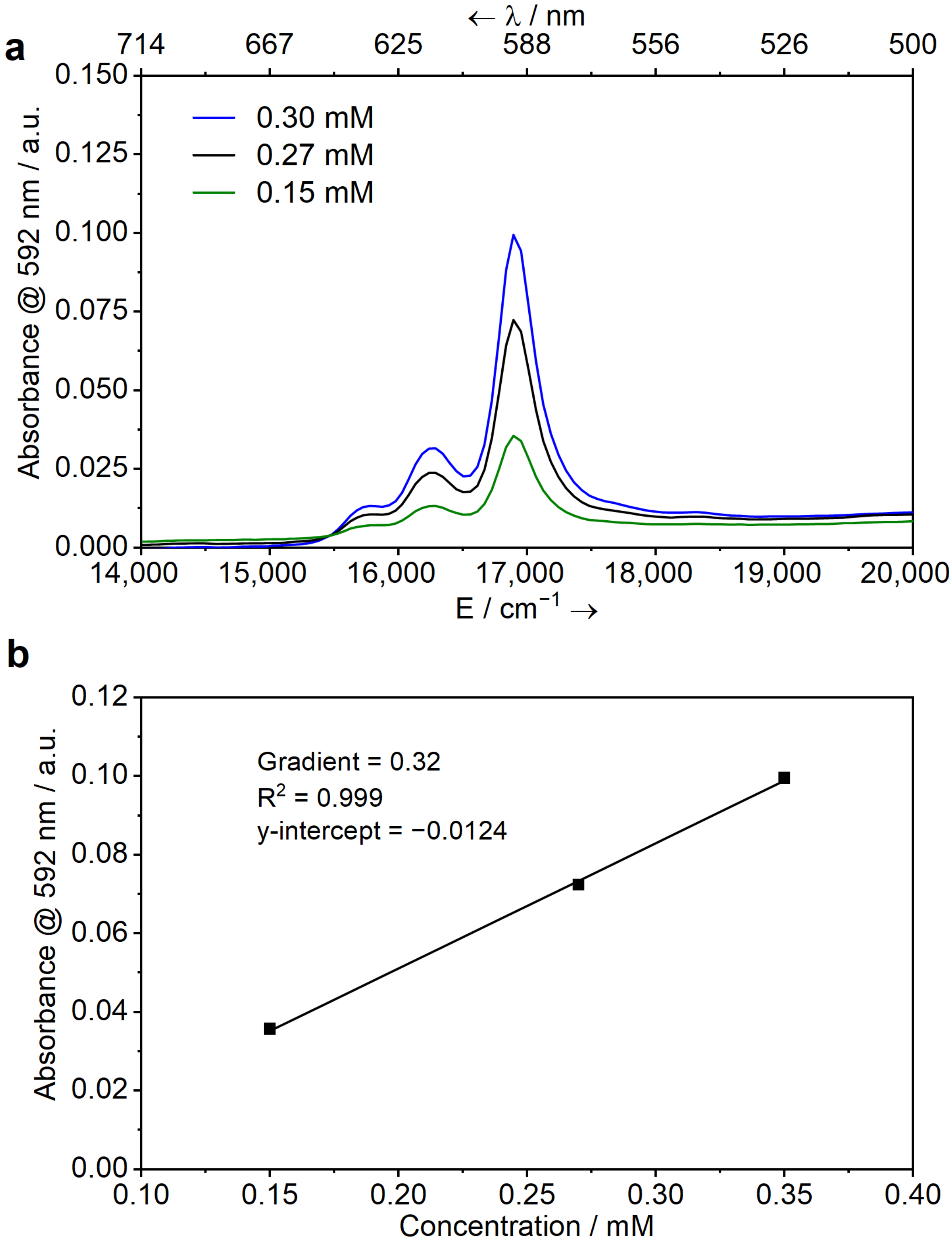

Figure S124. (**a**) Solution UV-Vis-NIR spectra of **4** at 0.15 mM, 0.27 mM and 0.35 mM in THF using a 0.4 cm cuvette shown between 14,000–20,000 cm^{-1} (714–500 nm) at ambient temperature. (**b**) Linear regression of absorbance at 16,892 cm^{-1} (592 nm) of **4** where ε = 800 M^{-1} cm^{-1}.

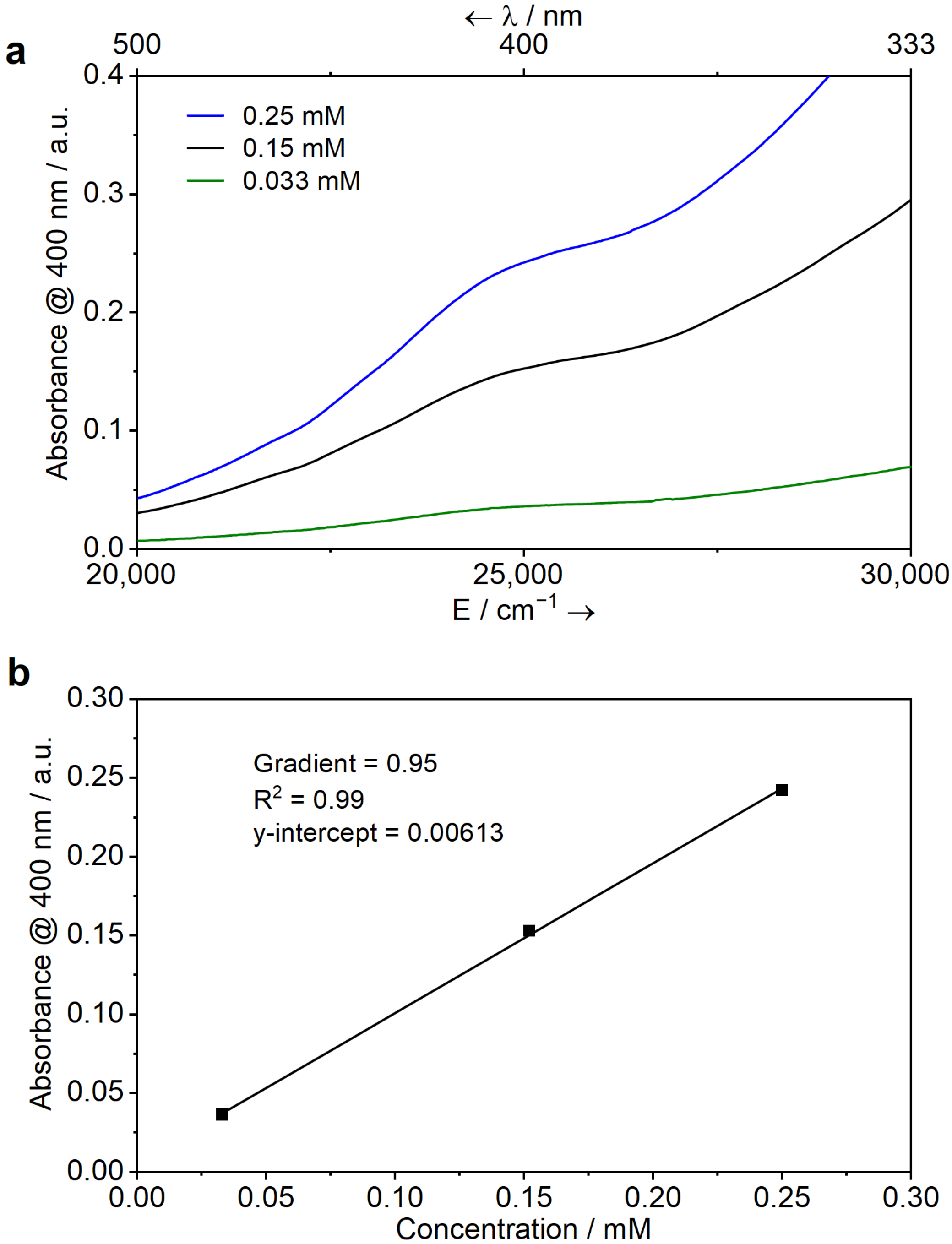

Figure S125. (**a**) Solution UV-Vis-NIR spectra of **5** at 0.033 mM, 0.15 mM and 0.25 mM in THF using a 0.4 cm cuvette shown between 20,000–30,000 cm^{-1} (500–333 nm) at ambient temperature. (**b**) Linear regression of absorbance at 25,000 cm^{-1} (400 nm) of **5** where ε = 2,375 M^{-1} cm^{-1}.

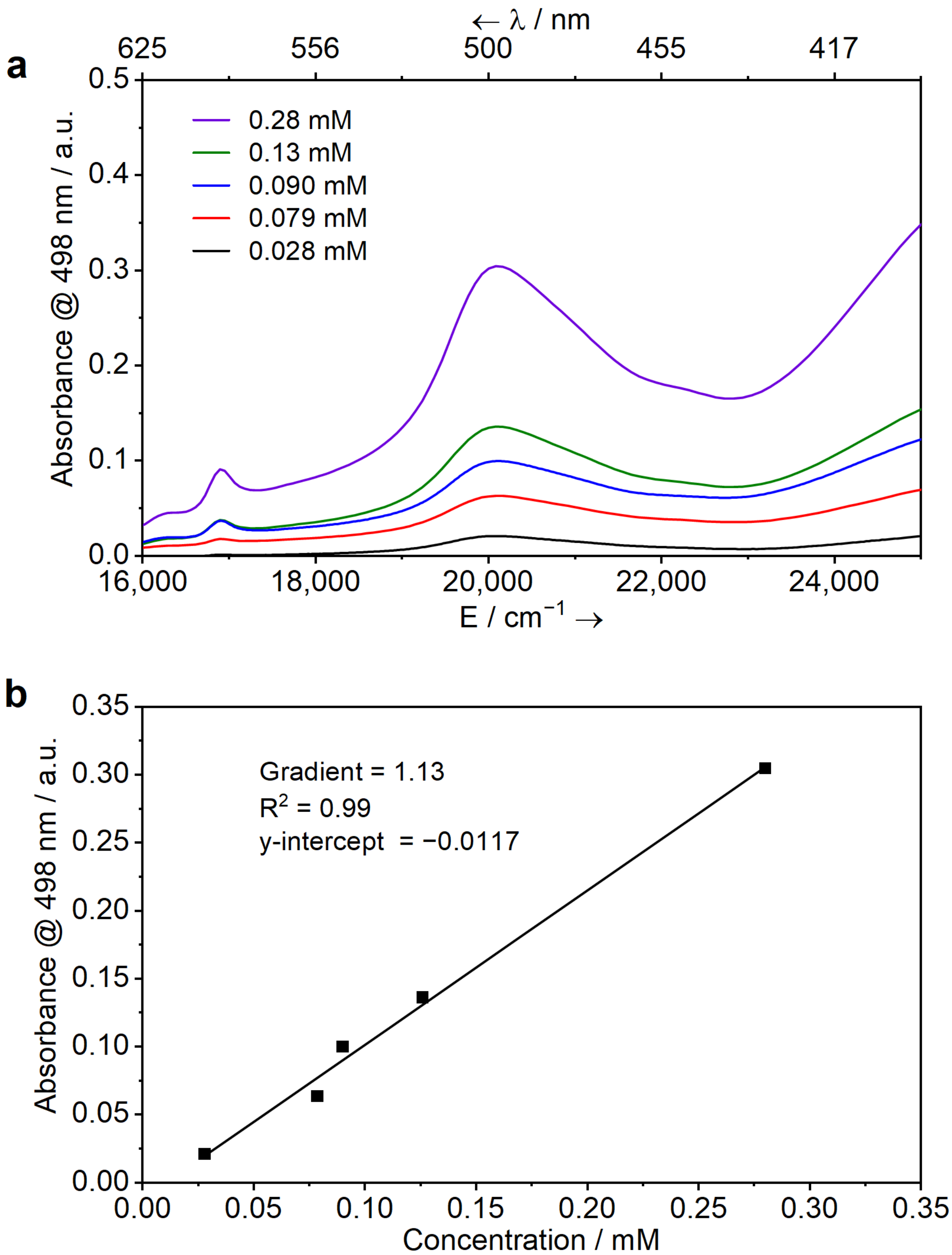

Figure S126. (**a**) Solution UV-Vis-NIR spectra of **6** at 0.028 mM, 0.079 mM, 0.090 mM, 0.13 mM and 0.28 mM in Et_2O using a 0.4 cm cuvette shown between 18,000–24,000 cm^{-1} (556–417 nm) at ambient temperature. (**b**) Linear regression of absorbance at 20,080 cm^{-1} (498 nm) in **6** where ε = 2,825 M^{-1} cm^{-1}.

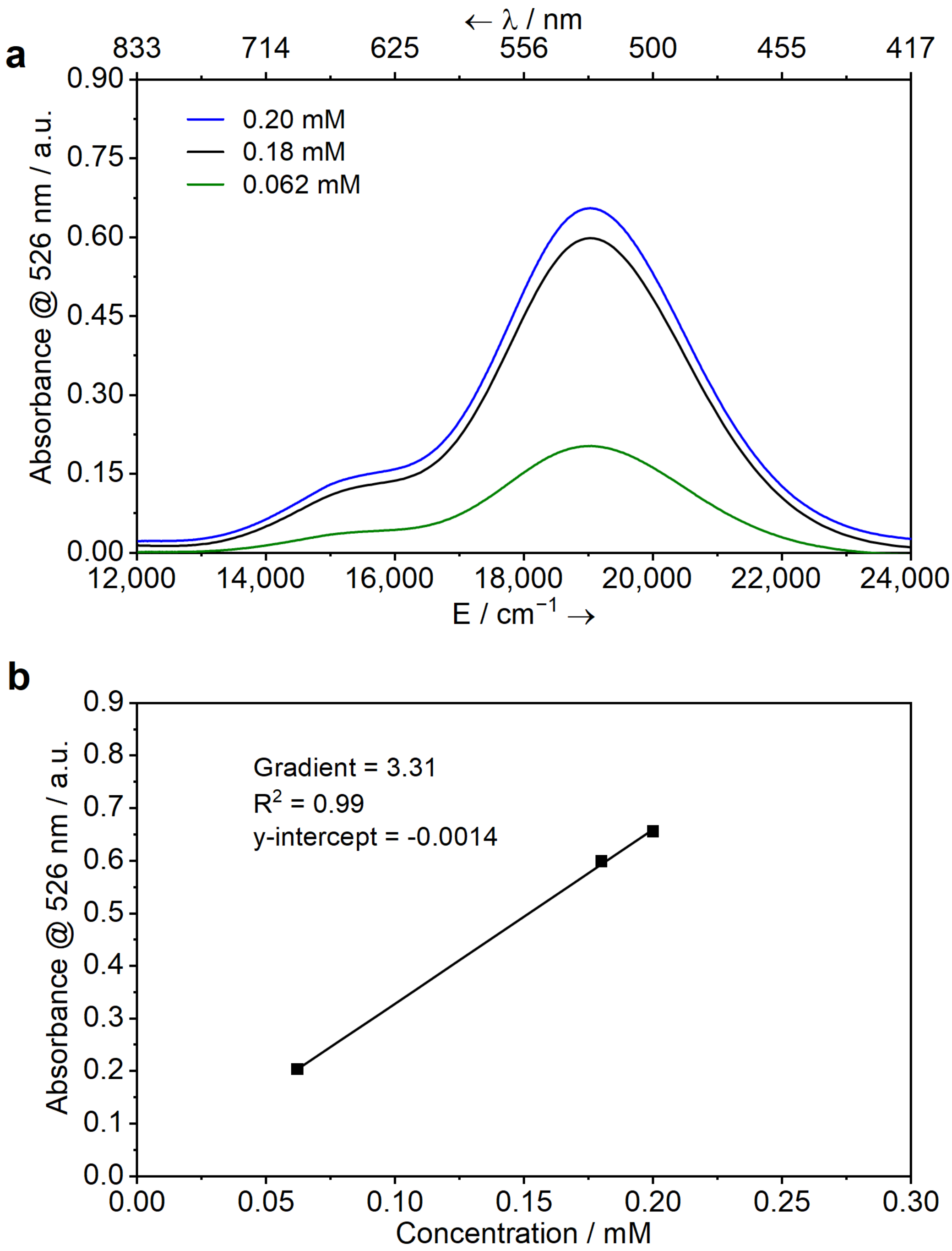

Figure S127. (**a**) Solution UV-Vis-NIR spectra of **9** at 0.062 mM, 0.18 mM and 0.20 mM in THF using a 0.4 cm cuvette shown between 12,000–24,000 cm^{-1} (833–556 nm) at ambient temperature. (**b**) Linear regression of absorbance at 19,011 cm^{-1} (526 nm) of **9** where ε = 8,275 M^{-1} cm^{-1}.

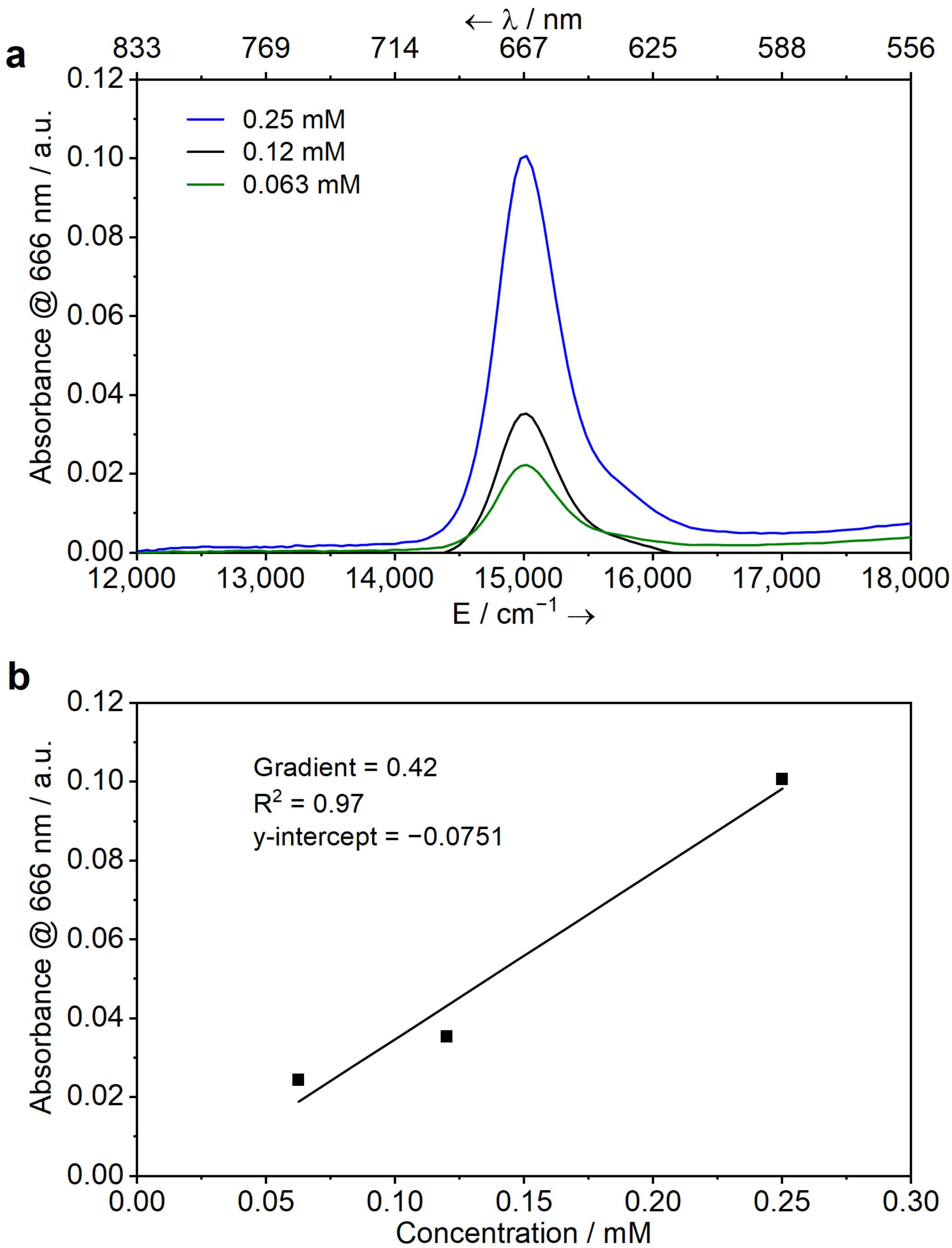

Figure S128. (**a**) Solution UV-Vis-NIR spectra of **10** at 0.063 mM, 0.12 mM and 0.25 mM in THF using a 0.4 cm cuvette shown between 12,000–18,000 cm^{-1} (833–556 nm) at ambient temperature. (**b**) Linear regression of absorbance at 15,015 cm^{-1} (666 nm) of **10** where ε = 1,050 M^{-1} cm^{-1}.

*Decomposition studies of complexes **3** and **6***

*Complex **3** with THF in oDFB*

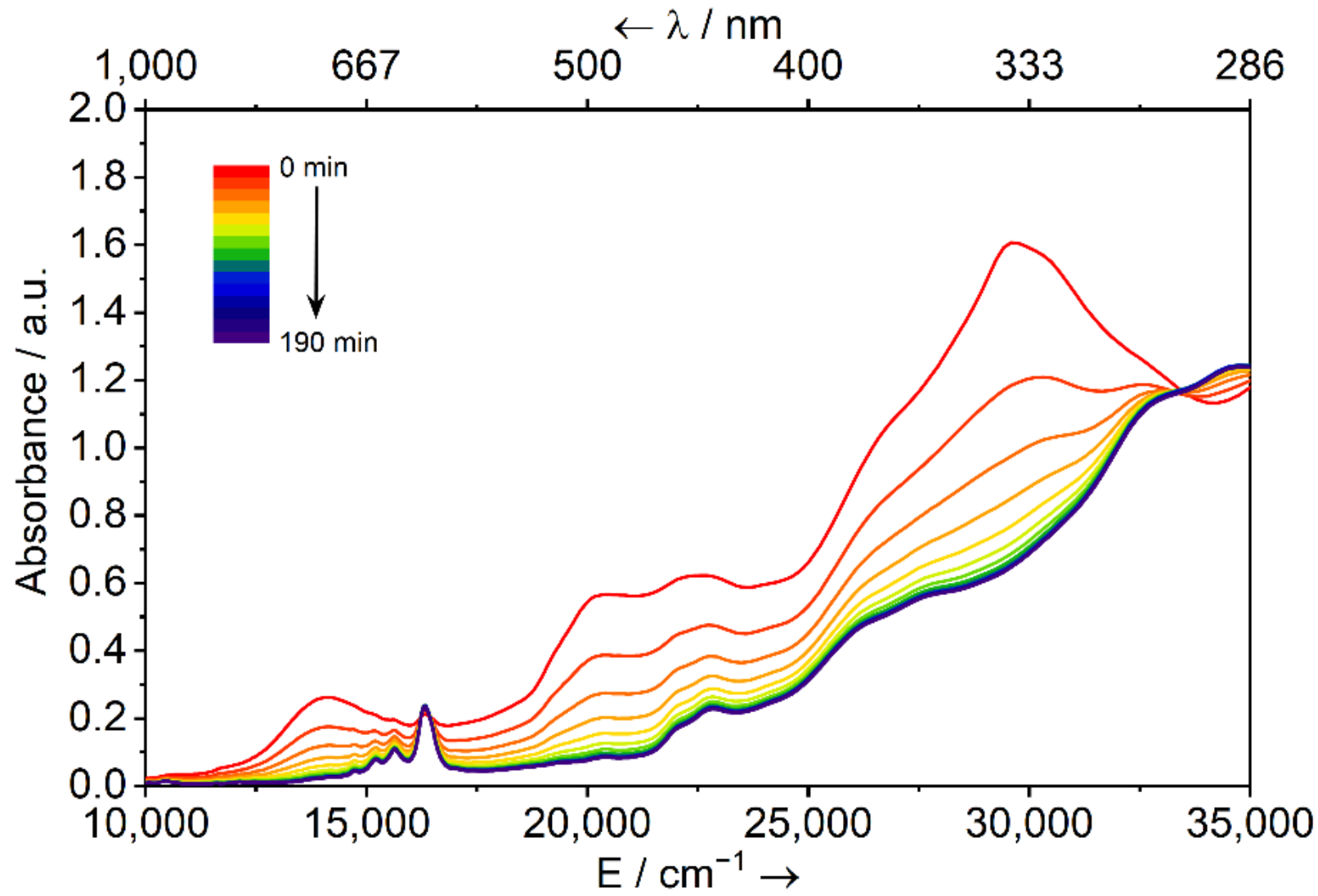

Figure S129. Sequential solution UV-Vis-NIR spectra taken every 10 minutes for 190 minutes of **3** at 0.36 mM in a 5:1 w/w *o*DFB:THF solution in a 1 cm cuvette shown between 10,000–35,000 cm^{-1} (1,000–286 nm) at ambient temperature. The final concentration of **1** was 0.35 mM.

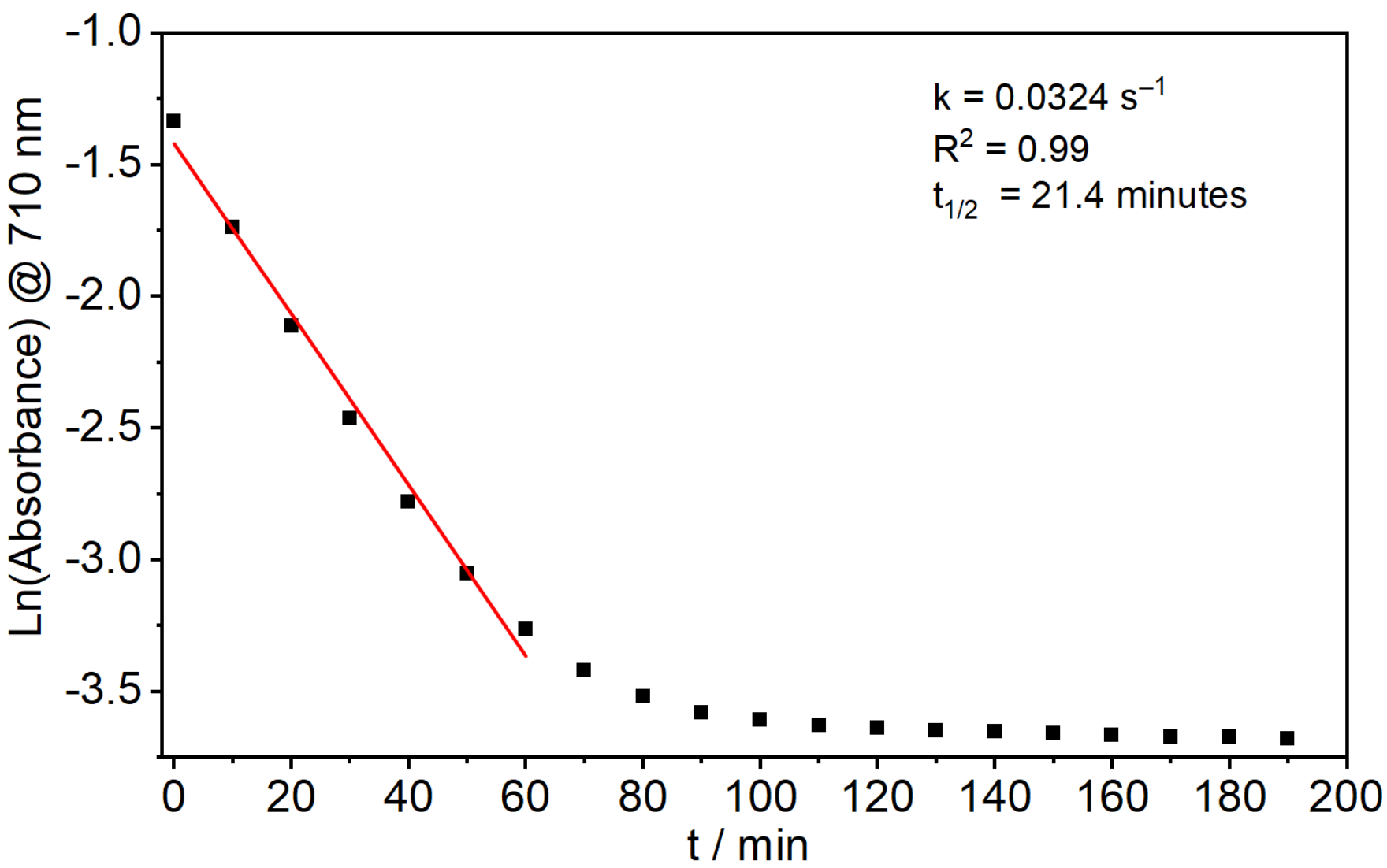

Figure S130. Natural log of the absorbance of **3** at 710 nm in a 5:1 w/w *o*DFB:THF solution in a 1 cm cuvette taken every 10 minutes for 190 minutes. Linear regression of the straight portion of the graph gave k = 0.0324 s^{-1} and $t_{1/2}$ = 21.4 minutes.

*Complex **6** with THF in oDFB*

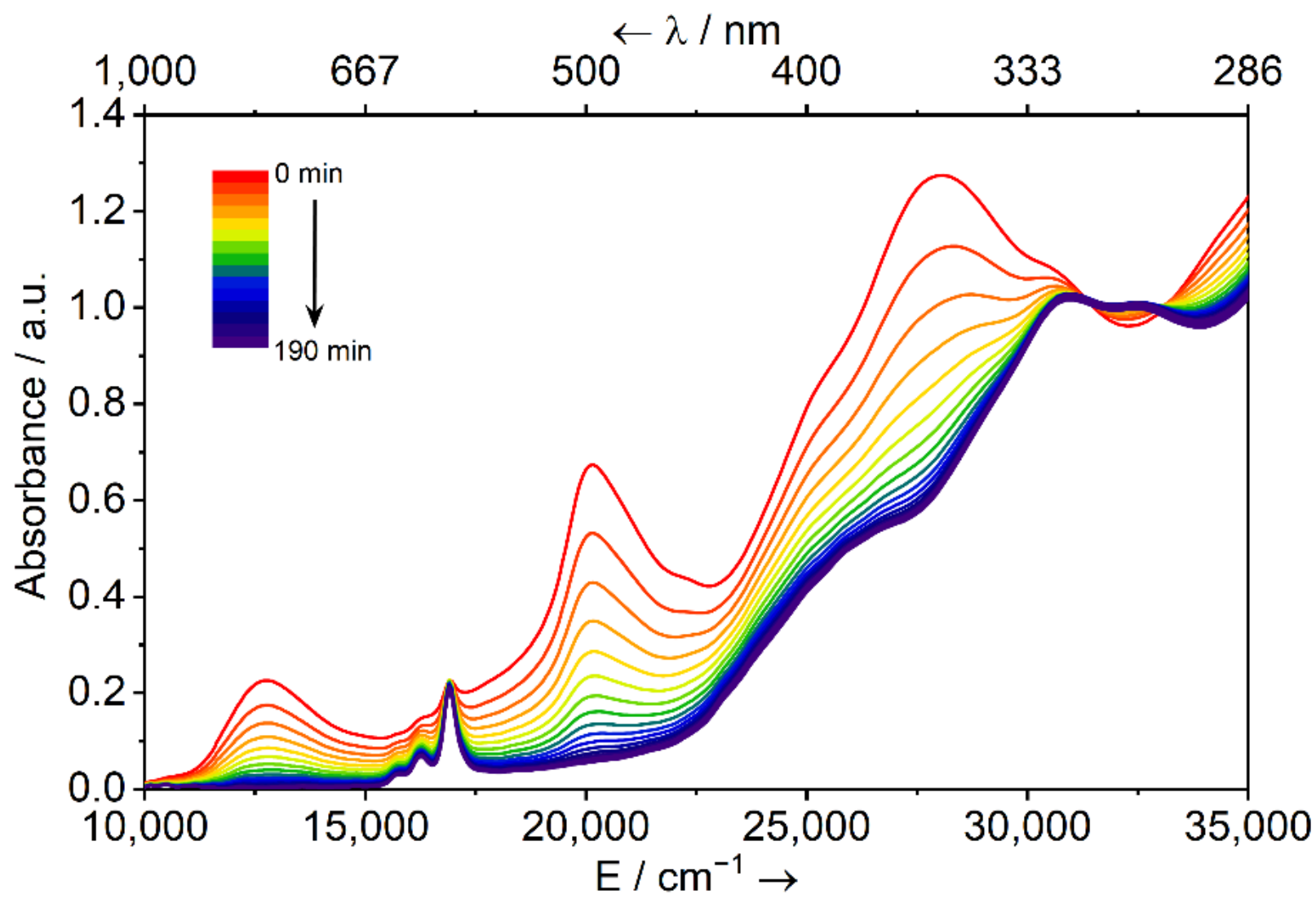

Figure S131. Sequential solution UV-Vis-NIR spectra taken every 10 minutes for 190 minutes of **6** at 0.30 mM in a 5:1 w/w *o*DFB:THF solution in a 1 cm cuvette shown between 10,000–35,000 cm^{-1} (1,000–286 nm) at ambient temperature. The final concentration of **4** was 0.27 mM.

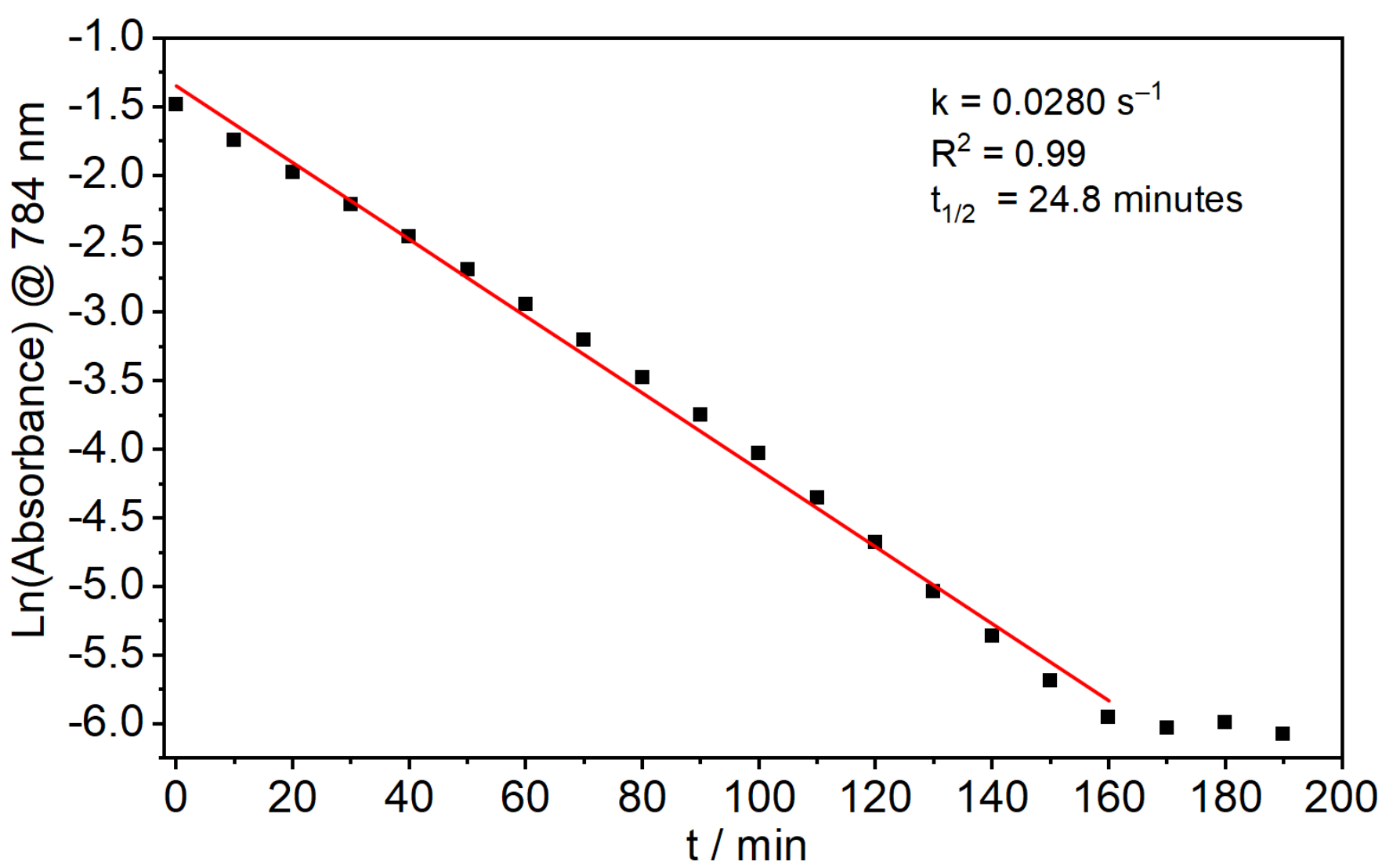

Figure S132. Natural log of the absorbance of **6** at 784 nm in a 5:1 w/w *o*DFB:THF solution in a 1 cm cuvette taken every 10 minutes for 190 minutes. Linear regression of the straight portion of the graph gave k = 0.0280 s^{-1} and $t_{1/2}$ = 24.8 minutes.

*Complex **3** with 2-MeTHF in oDFB*

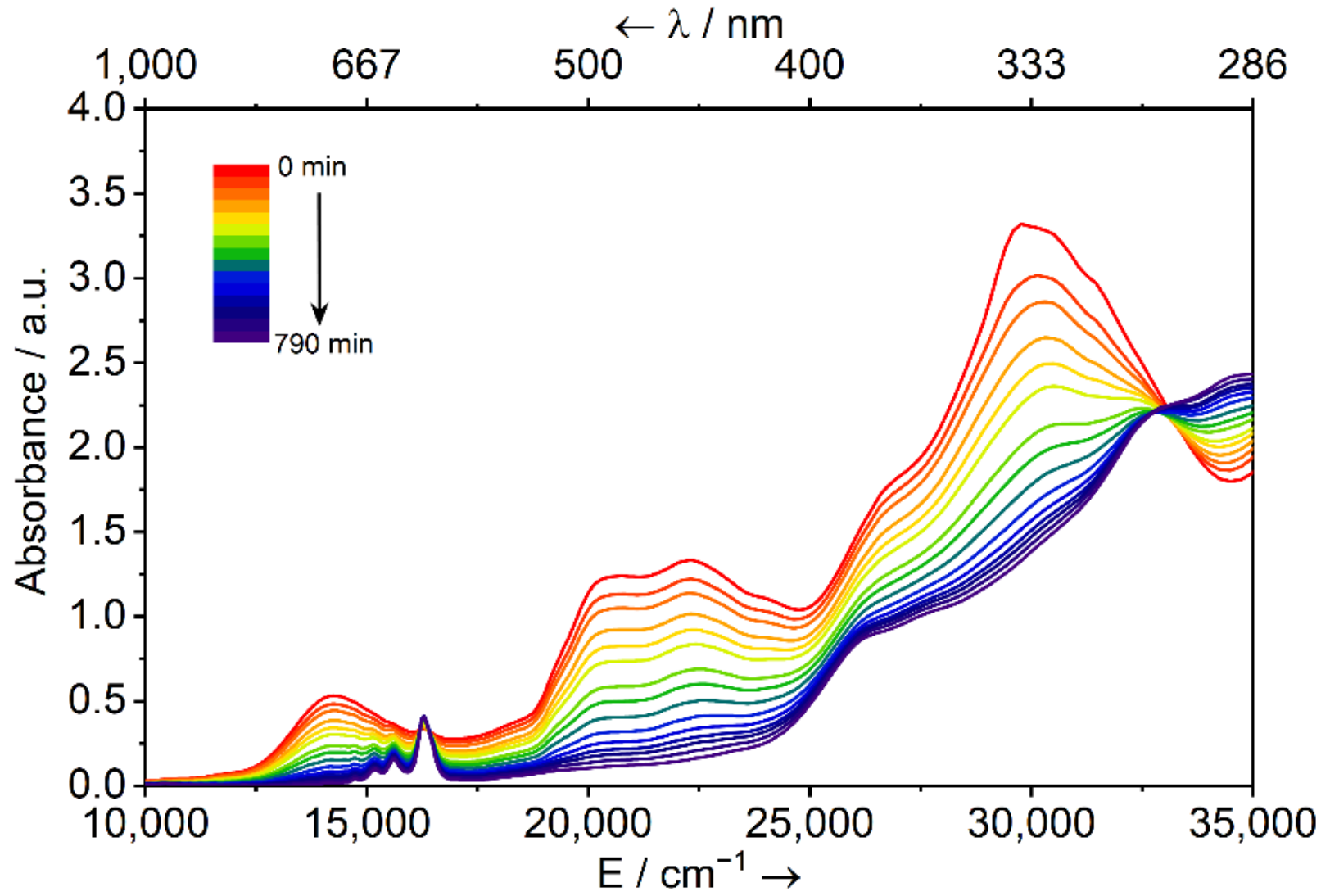

Figure S133. Selected sequential solution UV-Vis-NIR spectra taken every 10 minutes for 790 minutes of **3** at 0.30 mM in a 2-MeTHF solution in a 1 cm cuvette shown between 10,000–35,000 cm^{-1} (1,000–286 nm) at ambient temperature. The final concentration of **1** was 0.23 mM.

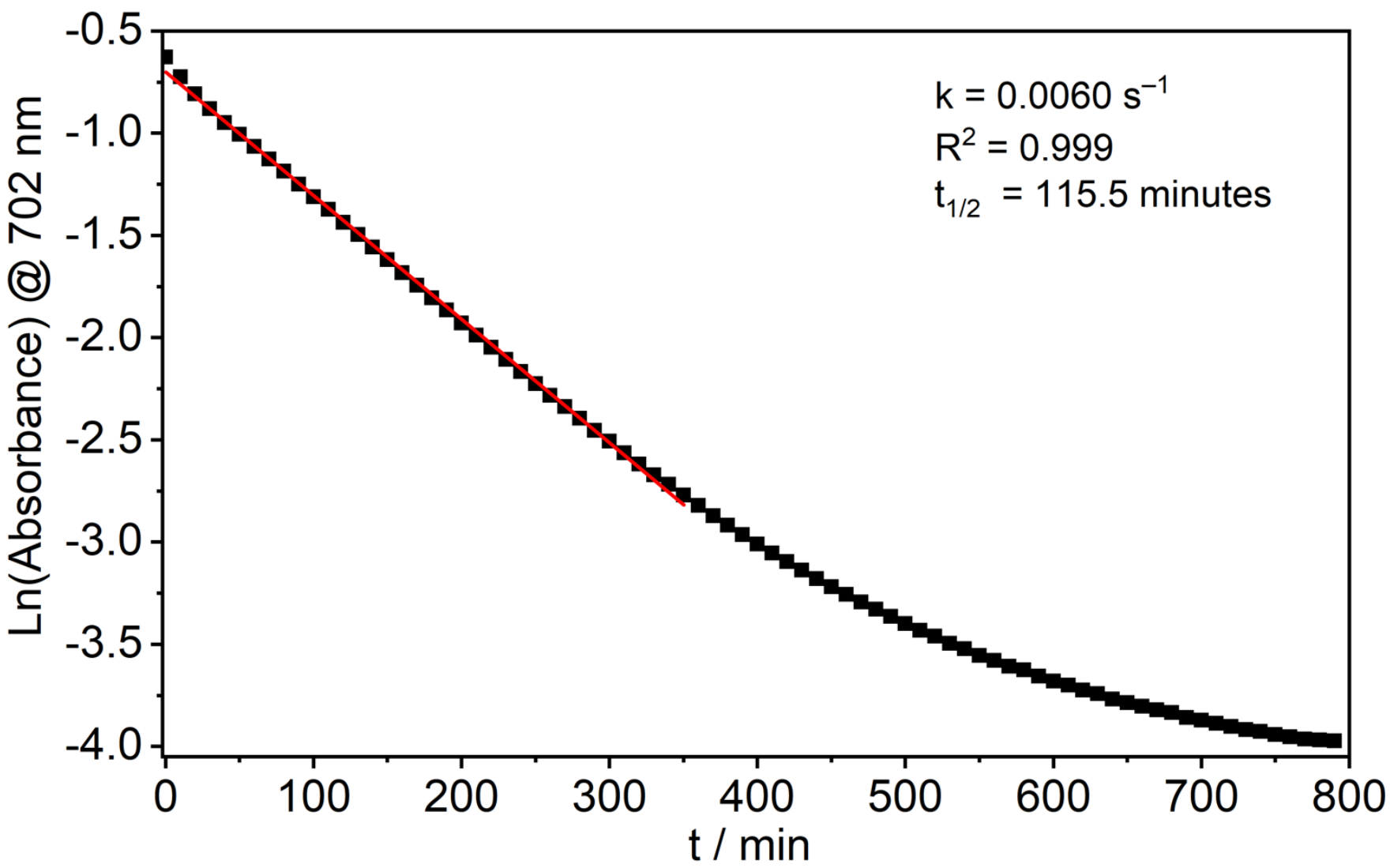

Figure S134. Natural log of the absorbance of **3** at 702 nm in a 2-MeTHF solution in a 1 cm cuvette taken every 10 minutes for 790 minutes. Linear regression of the straight portion of the graph gave k = 0.0060 s^{-1} and $t_{1/2}$ = 115.5 minutes.

*Complex **6** with 2-MeTHF in oDFB*

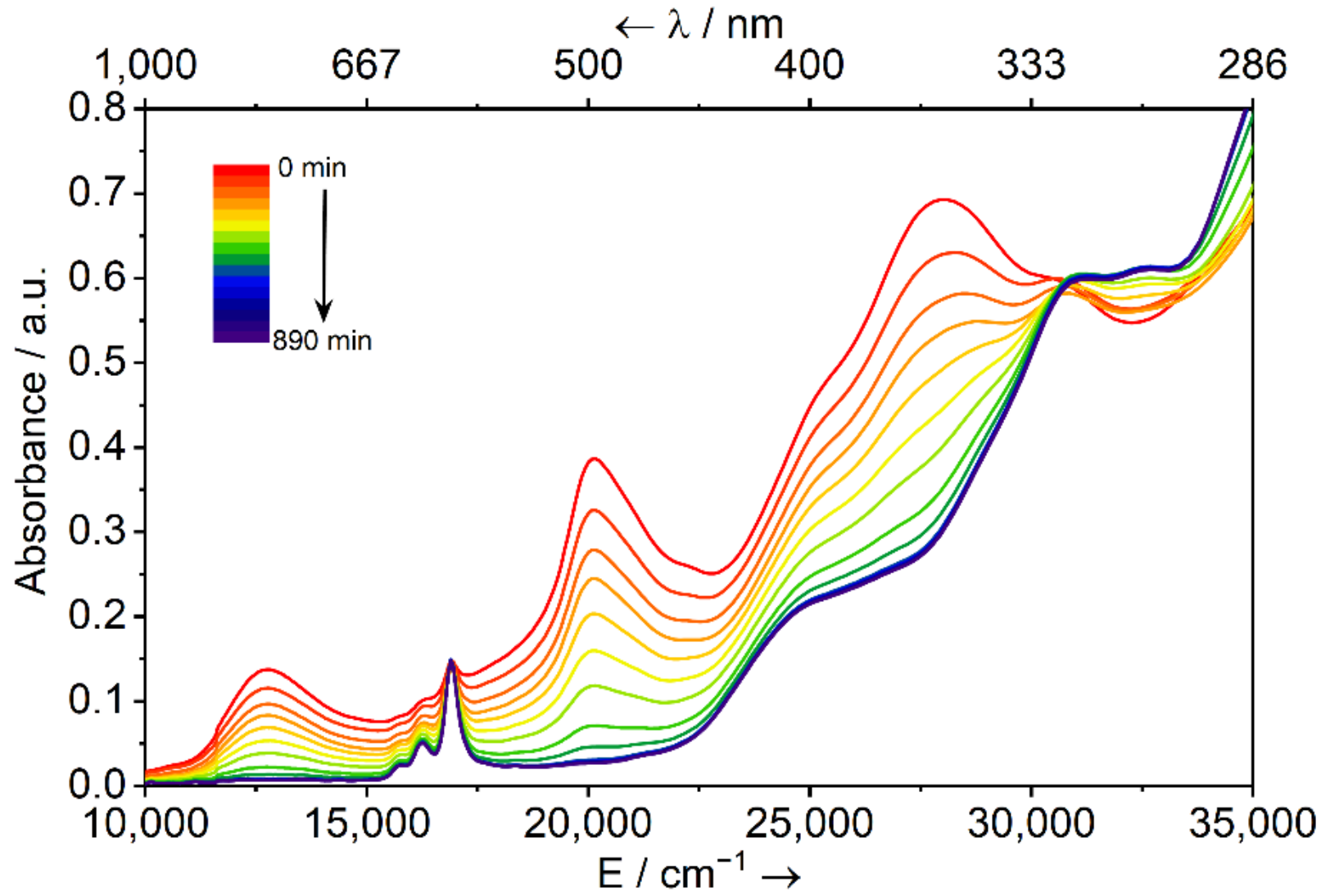

Figure S135. Selected sequential solution UV-Vis-NIR spectra taken every 10 minutes for 890 minutes of **6** at 0.18 mM in a 2-MeTHF solution in a 1 cm cuvette shown between 10,000–35,000 cm^{-1} (1,000–286 nm) at ambient temperature. The final concentration of **4** was 0.13 mM.

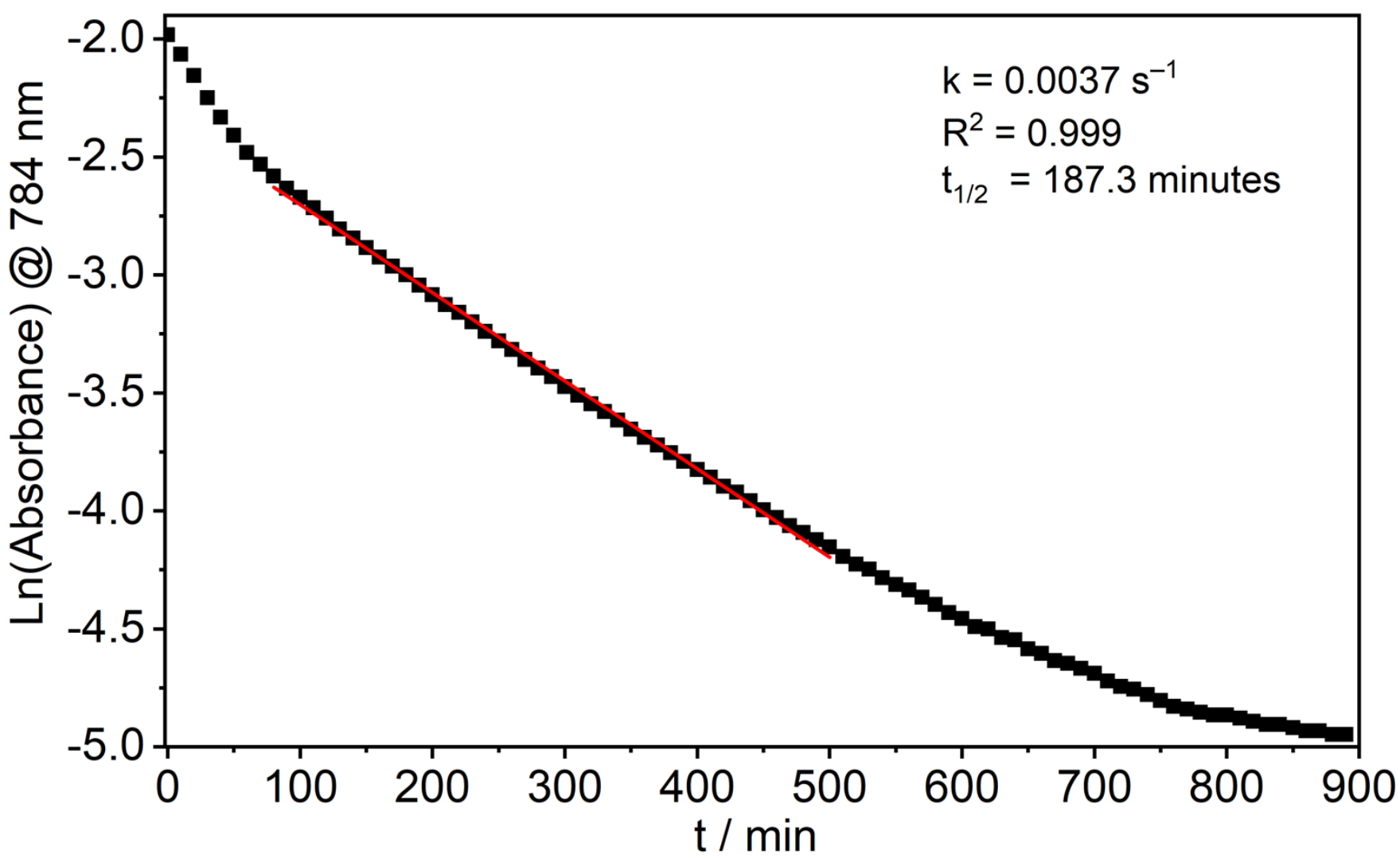

Figure S136. Natural log of the absorbance of **6** at 784 nm in a 2-MeTHF solution in a 1 cm cuvette taken every 10 minutes for 890 minutes. Linear regression of the straight portion of the graph gave k = 0.0037 s^{-1} and $t_{1/2}$ = 187.3 minutes.

*Stability of complexes **3** and **6** over time in solution*

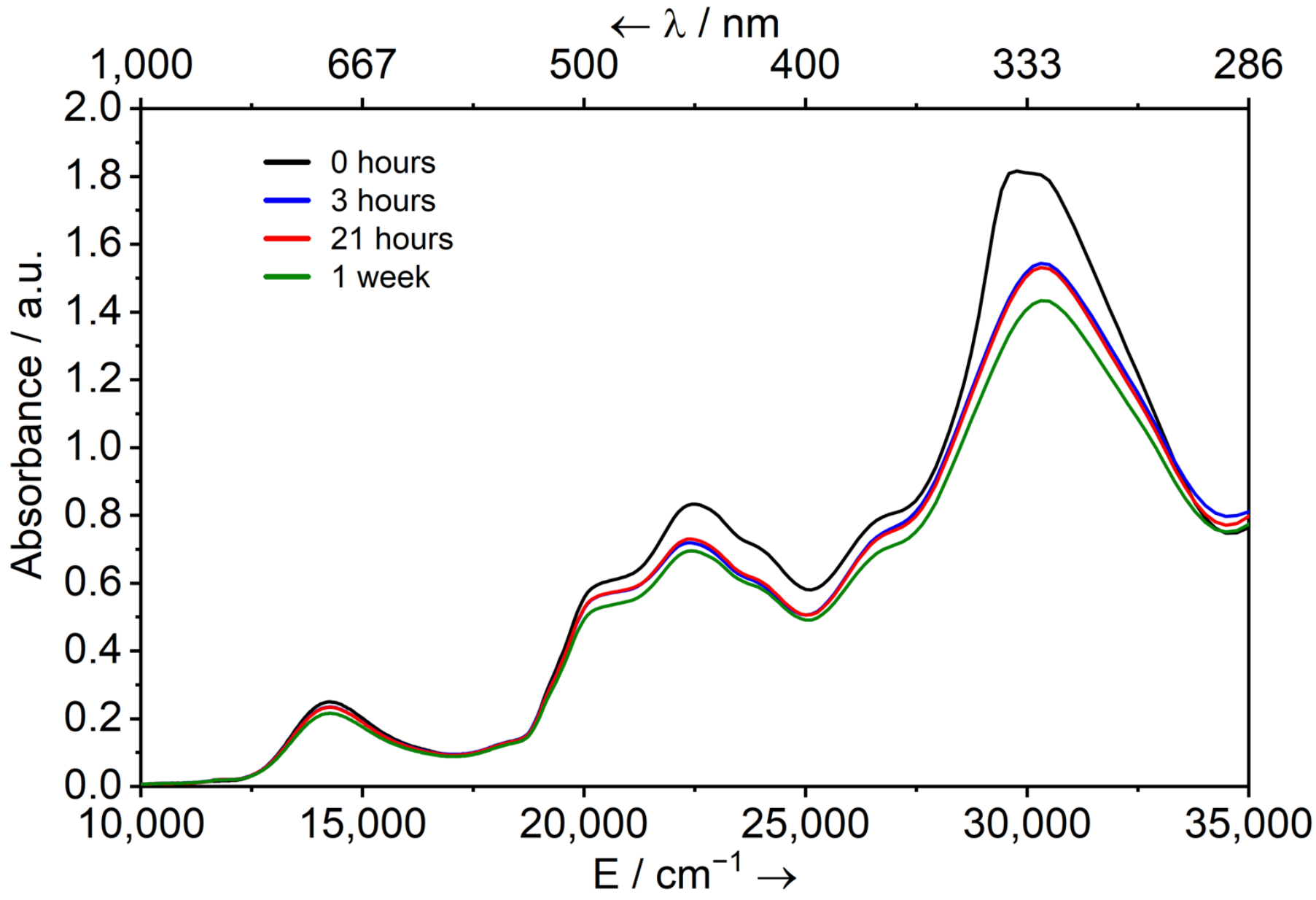

Figure S137. Sequential solution UV-Vis-NIR spectra of **3** at 0.36 mM in oDFB using a 1 cm cuvette shown between 10,000–35,000 cm^{-1} (1,000–286 nm) at ambient temperature.

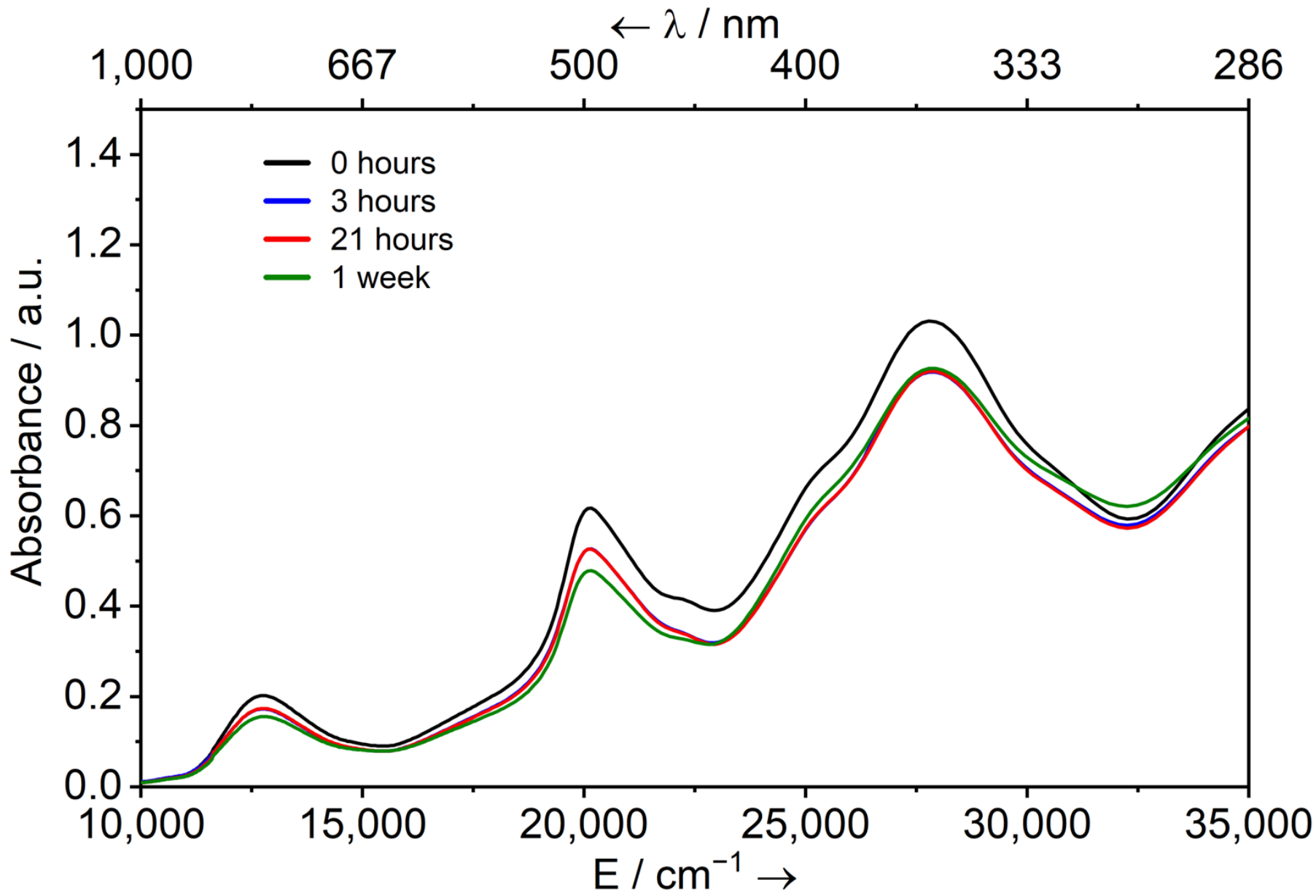

Figure S138. Sequential solution UV-Vis-NIR spectra of **6** at 0.17 mM in *o*DFB using a 1 cm cuvette shown between 10,000–35,000 cm^{-1} (1,000–286 nm) at ambient temperature.

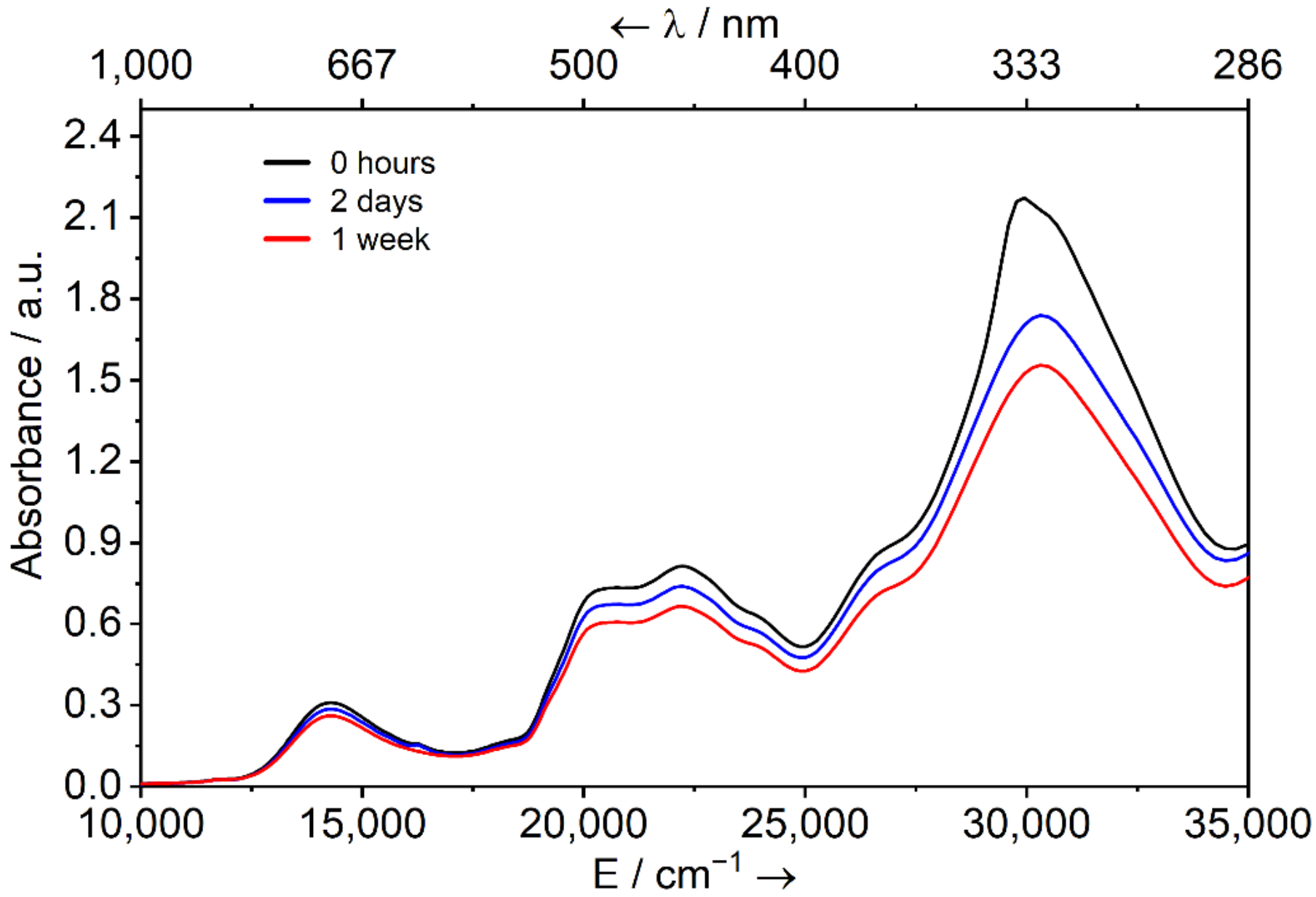

Figure S139. Sequential solution UV-Vis-NIR spectra of **3** at 0.31 mM in Et_2O using a 1 cm cuvette shown between 10,000–35,000 cm^{-1} (1,000–286 nm) at ambient temperature.

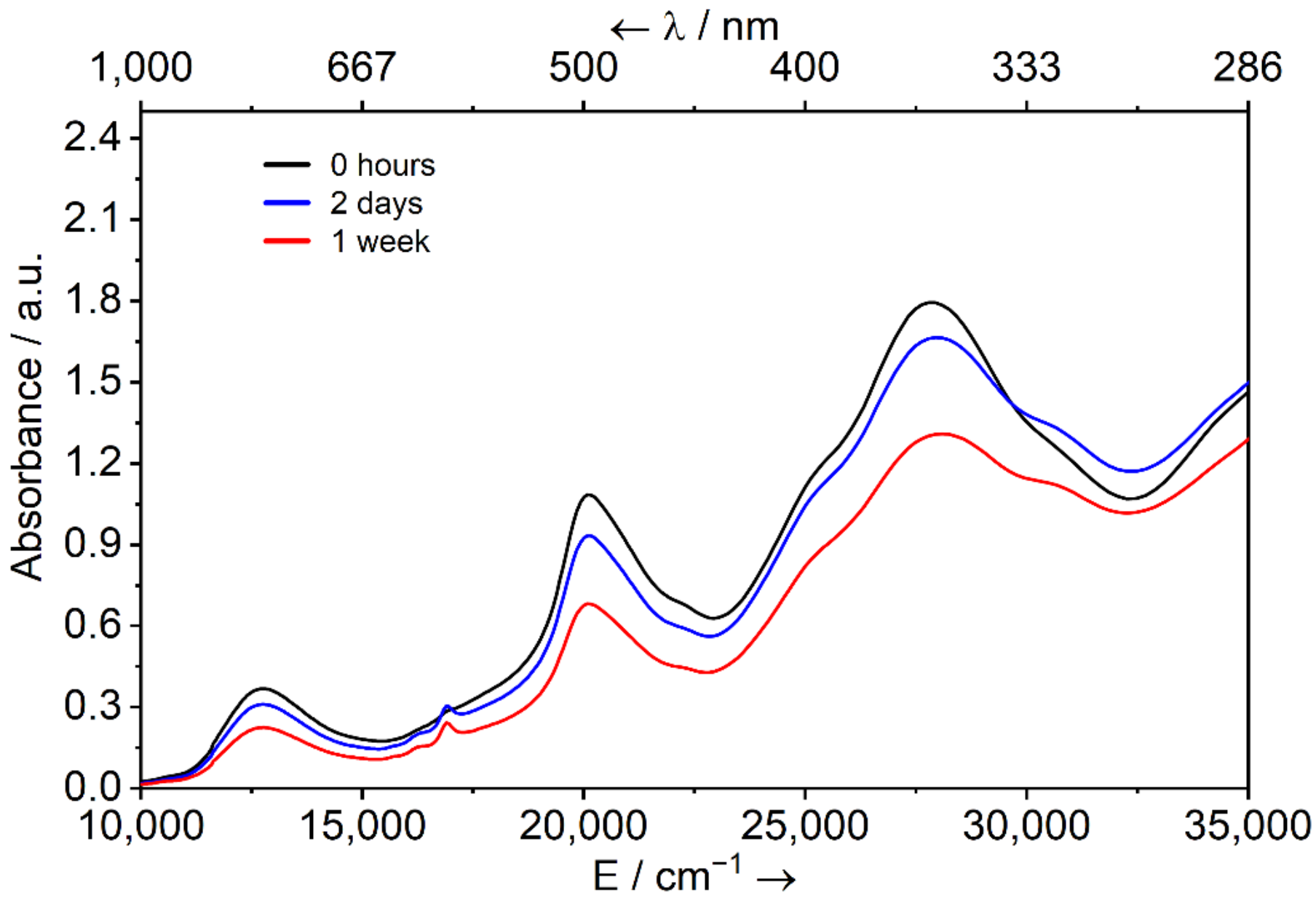

Figure S140. Sequential solution UV-Vis-NIR spectra of **6** at 0.37 mM in Et_2O using a 1 cm cuvette shown between 10,000–35,000 cm^{-1} (1,000–286 nm) at ambient temperature.

*Complex **6** with one equivalent of [nBu$_4$N][SbF$_6$] in oDFB*

A 4 mL 0.67 mM oDFB solution of **6** with one equivalent of [*n*Bu$_4$N][SbF$_6$] was added to a J. Youngs appended quartz cuvette. The decomposition of this mixture was followed by UV-Vis-NIR spectroscopy over 390 minutes with measurements taken every 10 minutes at ambient temperature.

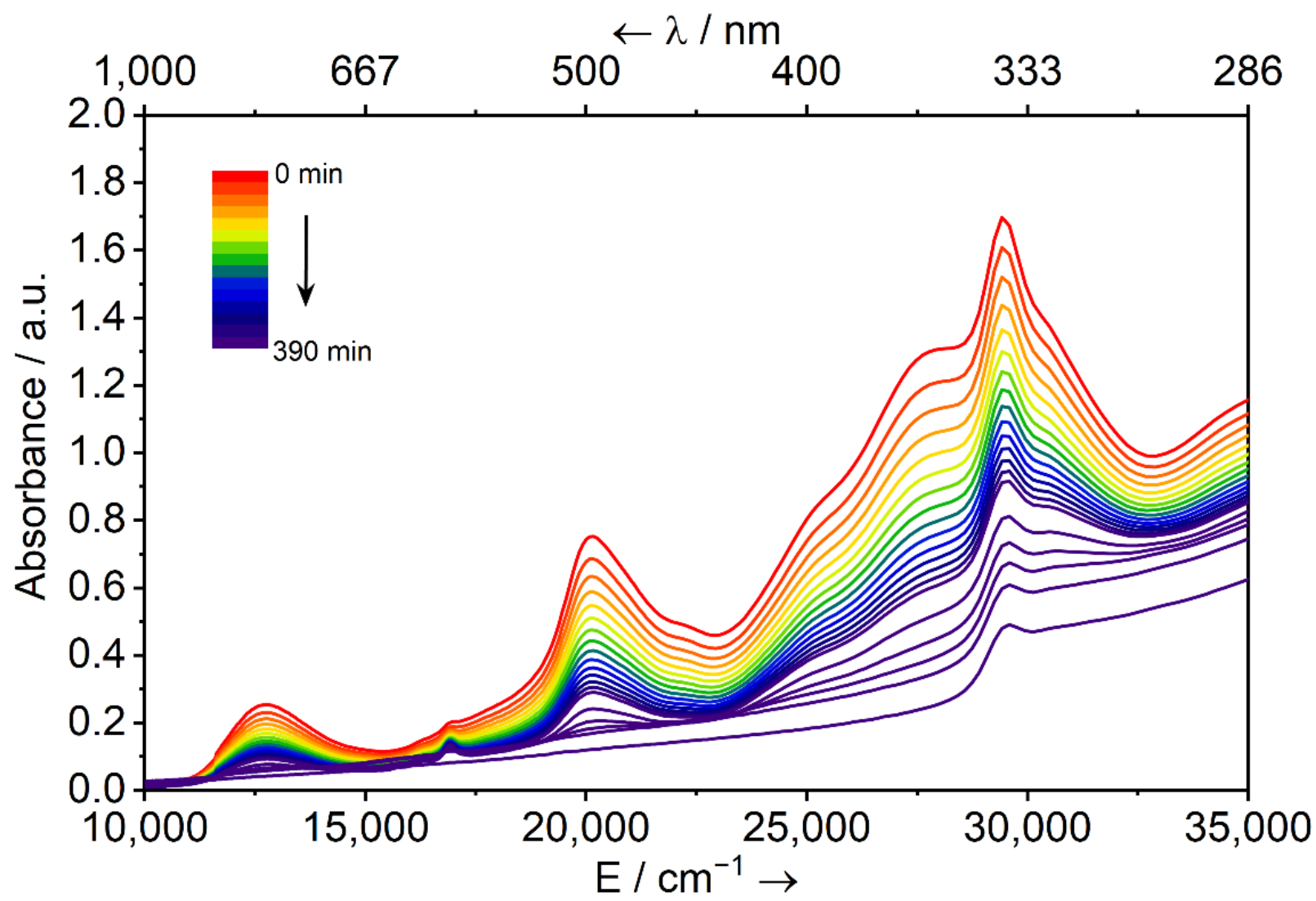

Figure S42. Sequential solution UV-Vis-NIR spectra taken every 10 minutes for 390 minutes of **6** (0.67 mM) and one equivalent of [*n*Bu$_4$N][SbF$_6$] in *o*DFB using a 1 cm path-length cuvette, shown between 10,000–35,000 cm–1 (1,000–286 nm) at ambient temperature.

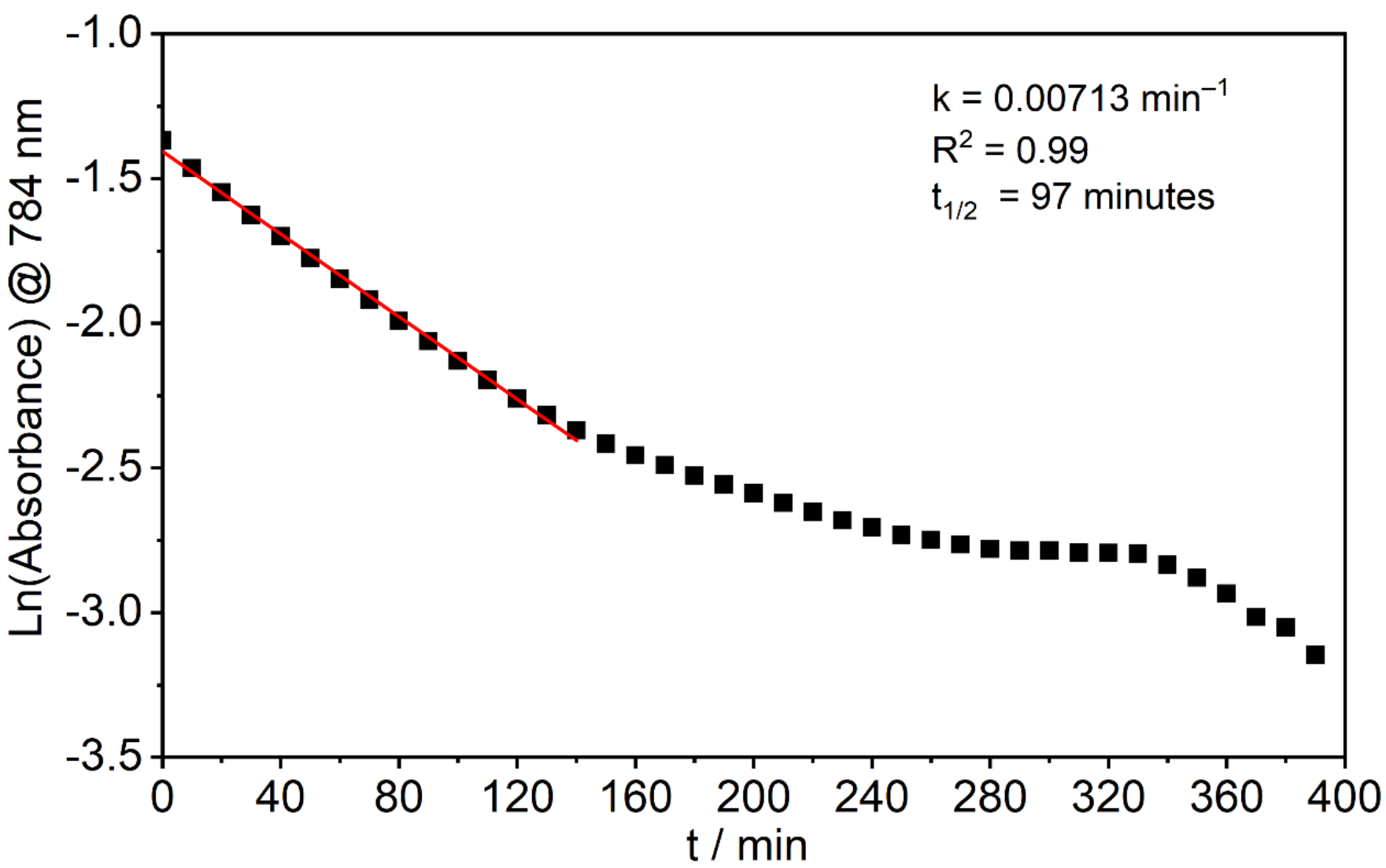

Figure S45. Natural log of the absorbance of **6** and one equivalent of [*n*Bu$_4$N][SbF$_6$] at 784 nm in *o*DFB using a 1 cm path-length cuvette, with measurements taken every 10 minutes for 390 minutes. Linear regression of the straight portion of the graph gave k = 0.00713 min^{-1} and $t_{1/2}$ = 97 minutes.

Solid-state UV-vis-NIR spectroscopy

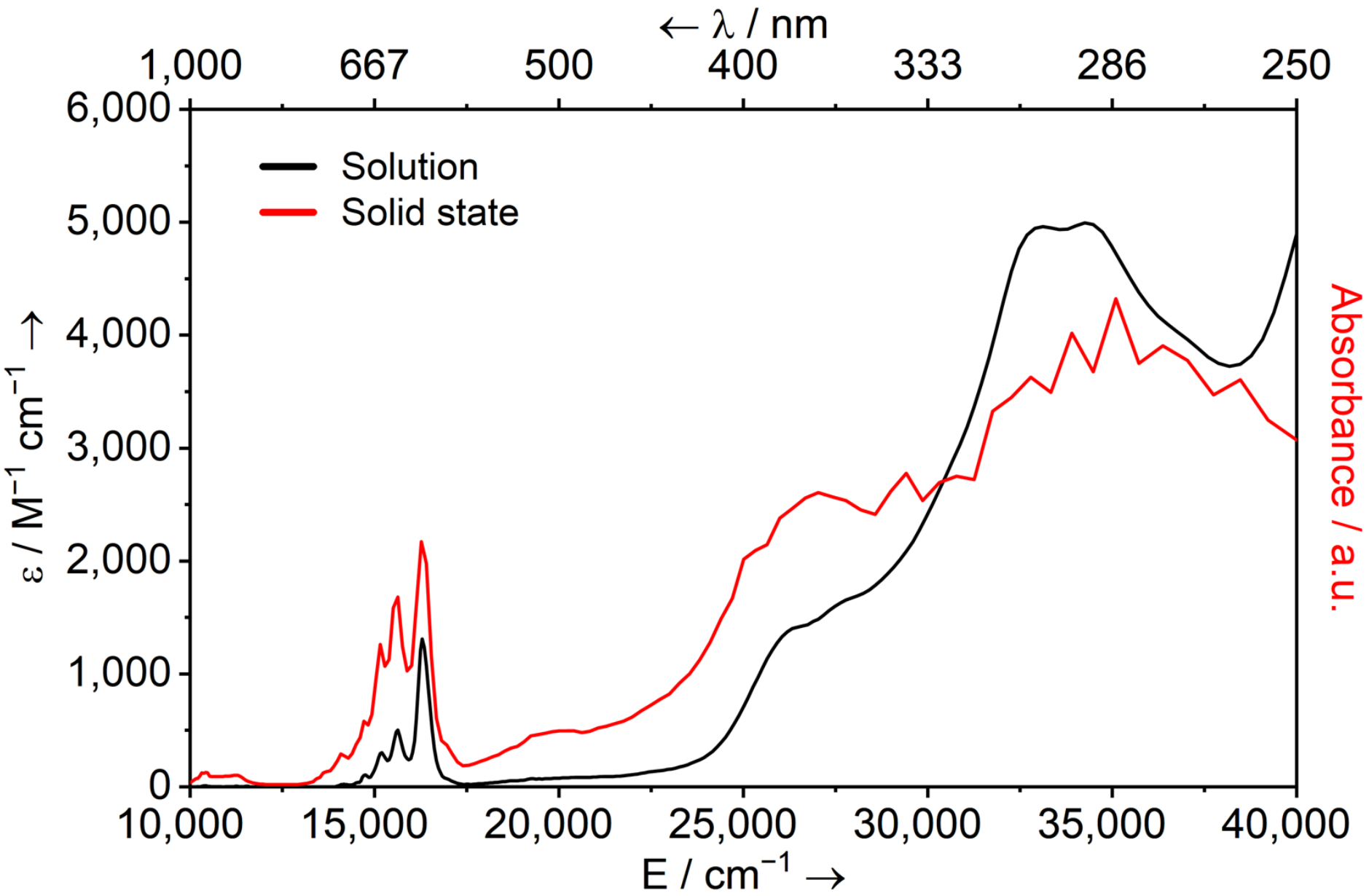

Figure S141. Solid-state UV-Vis-NIR spectrum of **1** (red) and solution UV-Vis-NIR spectrum of **1** (black) shown between 10,000–40,000 cm^{-1} (1,000–250 nm) at ambient temperature.

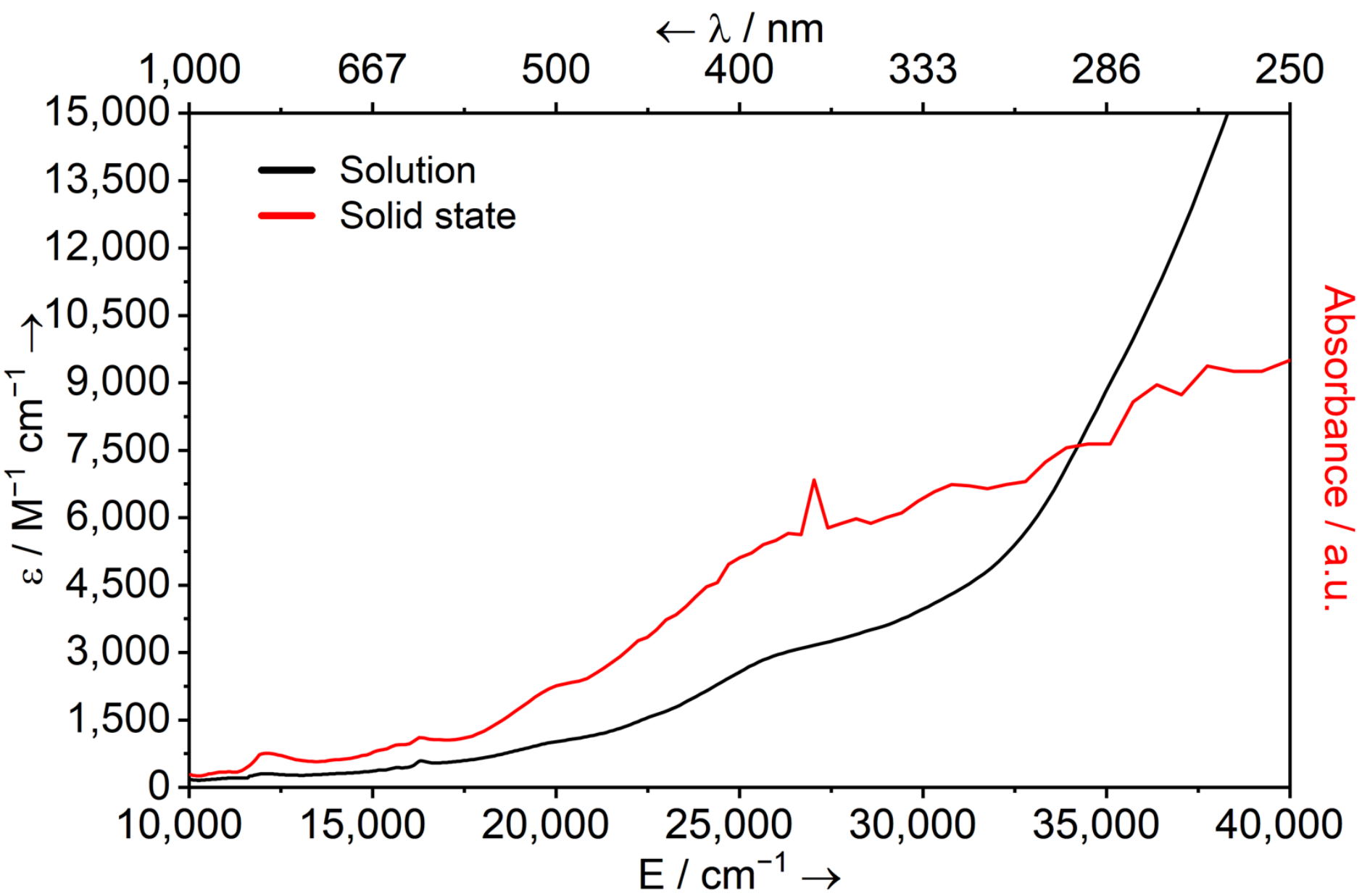

Figure S142. Solid-state UV-Vis-NIR spectrum of **2** (red) and solution UV-Vis-NIR spectrum of **2** (black) shown between 10,000–40,000 cm^{-1} (1,000–250 nm) at ambient temperature.

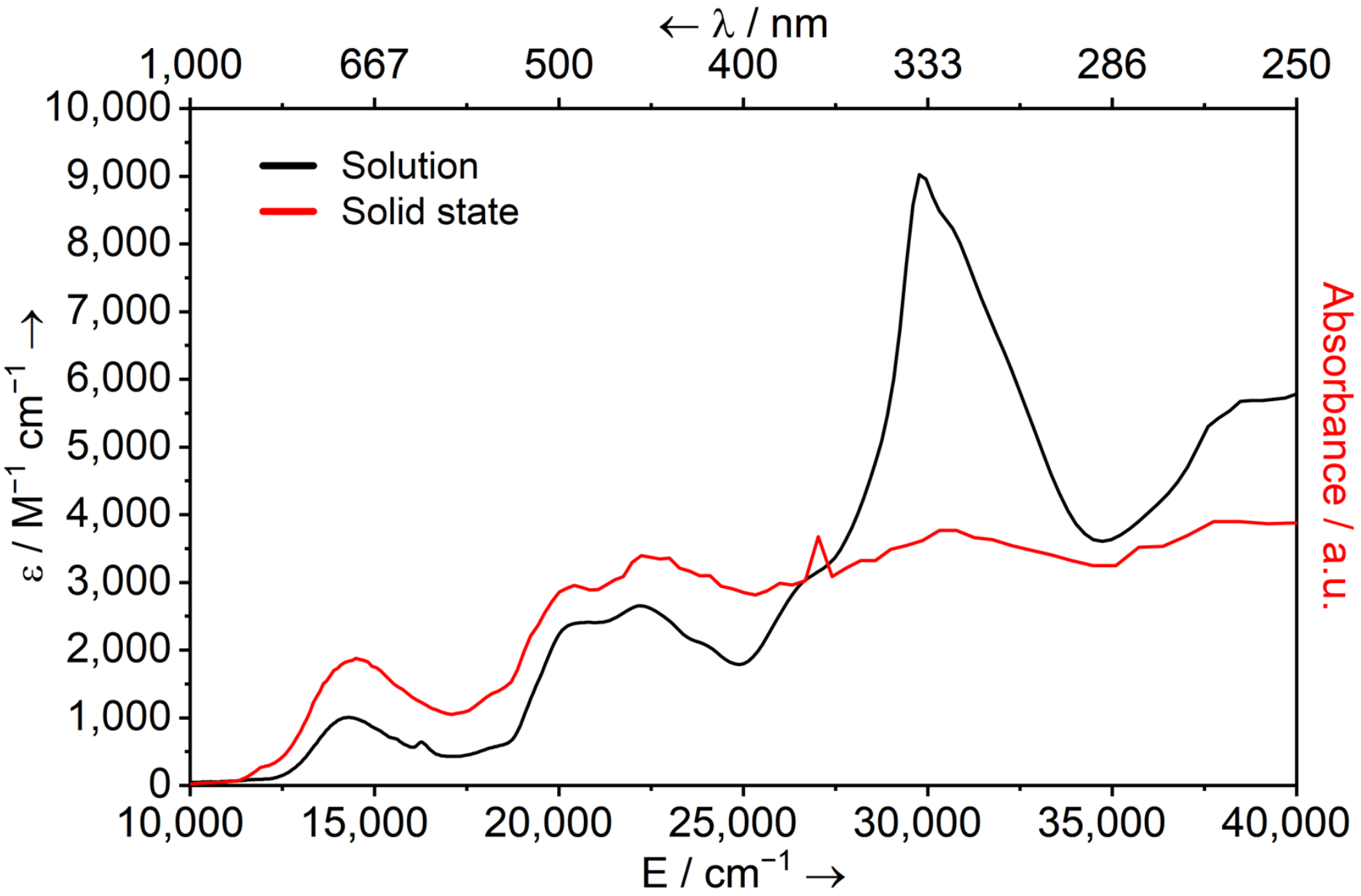

Figure S143. Solid-state UV-Vis-NIR spectrum of **3** (red) and solution UV-Vis-NIR spectrum of **3** (black) shown between 10,000–40,000 cm^{-1} (1,000–250 nm) at ambient temperature.

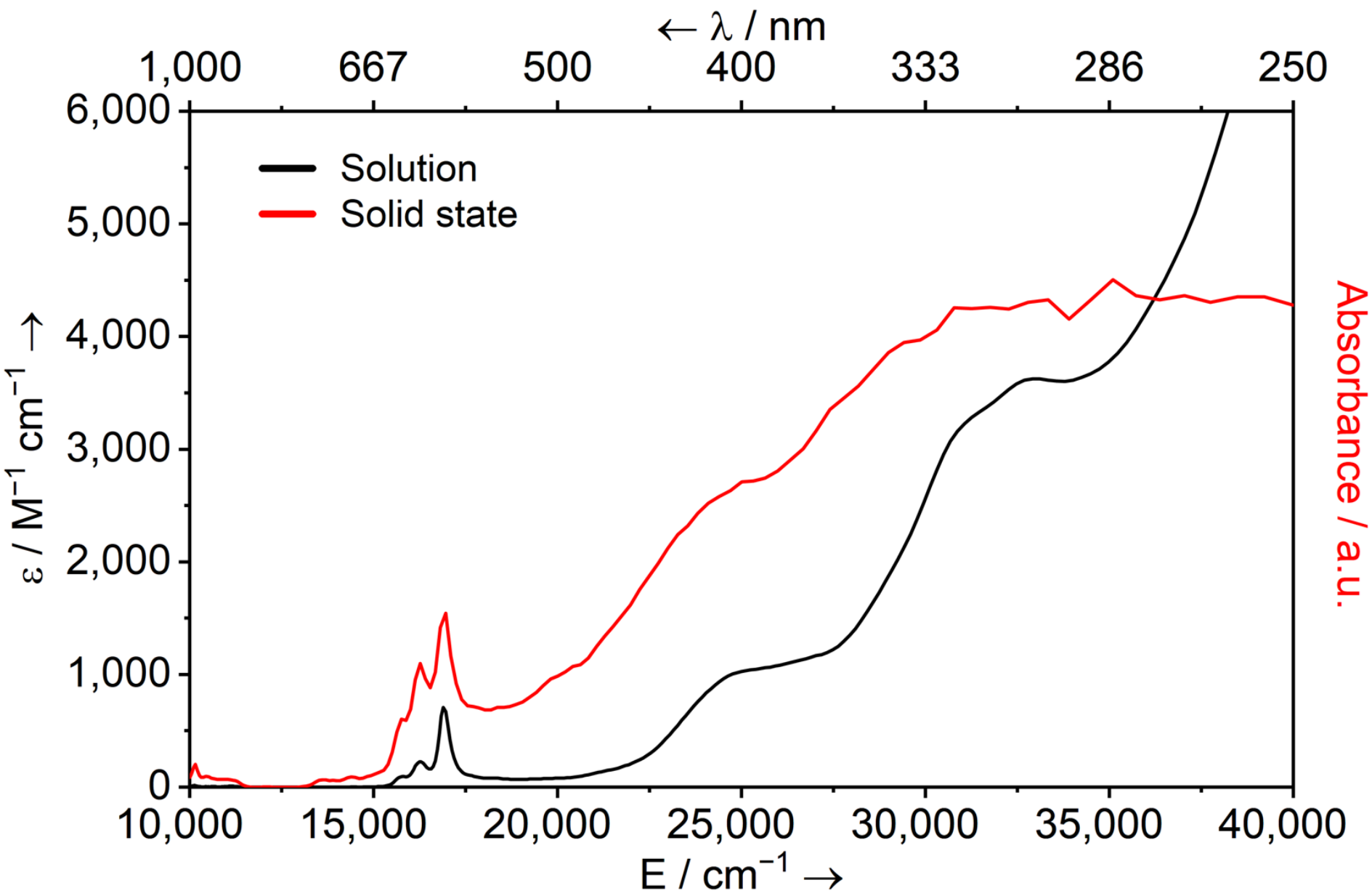

Figure S144. Solid-state UV-Vis-NIR spectrum of **4** (red) and solution UV-Vis-NIR spectrum of **4** (black) shown between 10,000–40,000 cm^{-1} (1,000–250 nm) at ambient temperature.

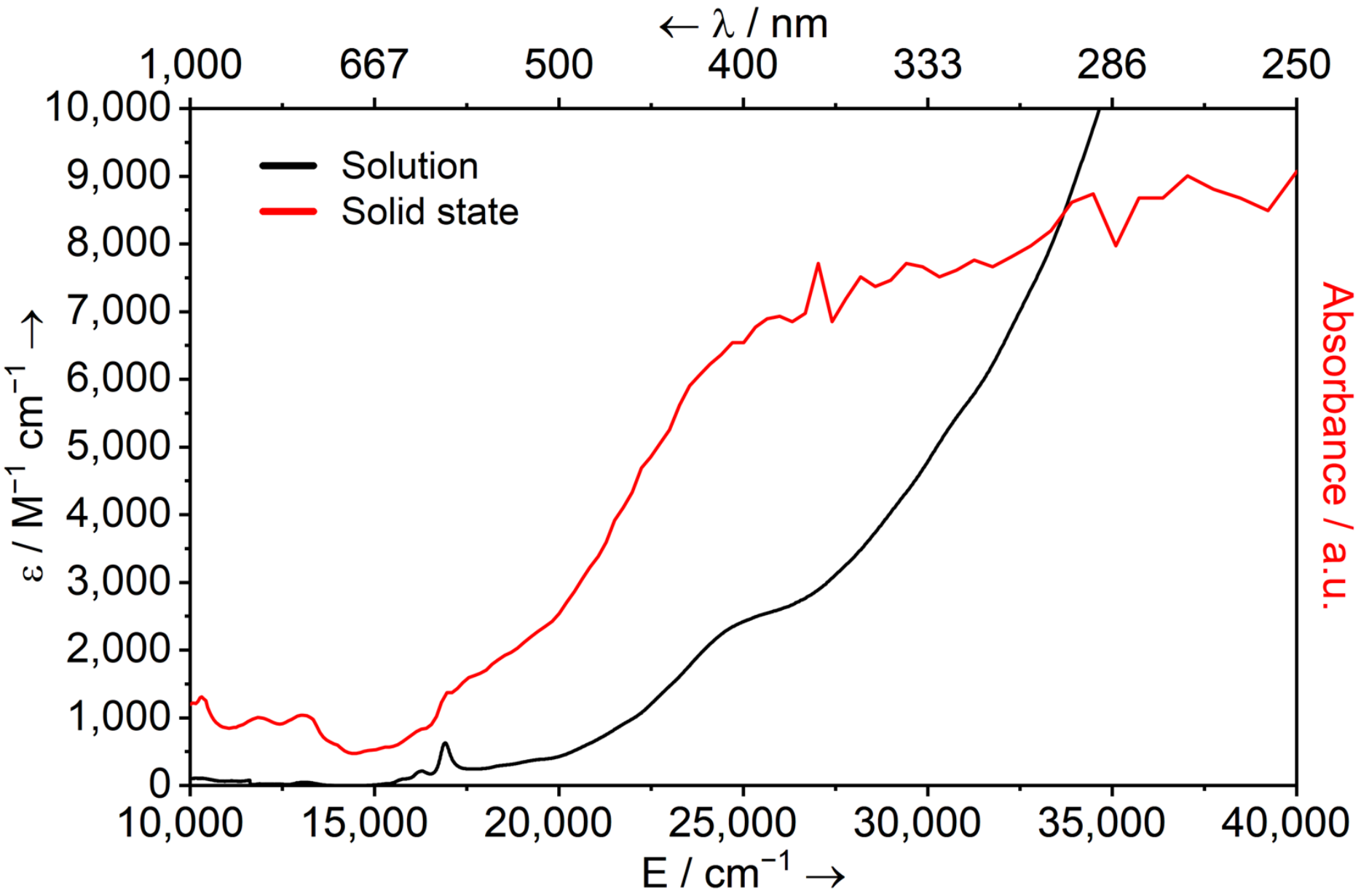

Figure S145. Solid-state UV-Vis-NIR spectrum of **5** (red) and solution UV-Vis-NIR spectrum of **5** (black) shown between 10,000–40,000 cm^{-1} (1,000–250 nm) at ambient temperature.

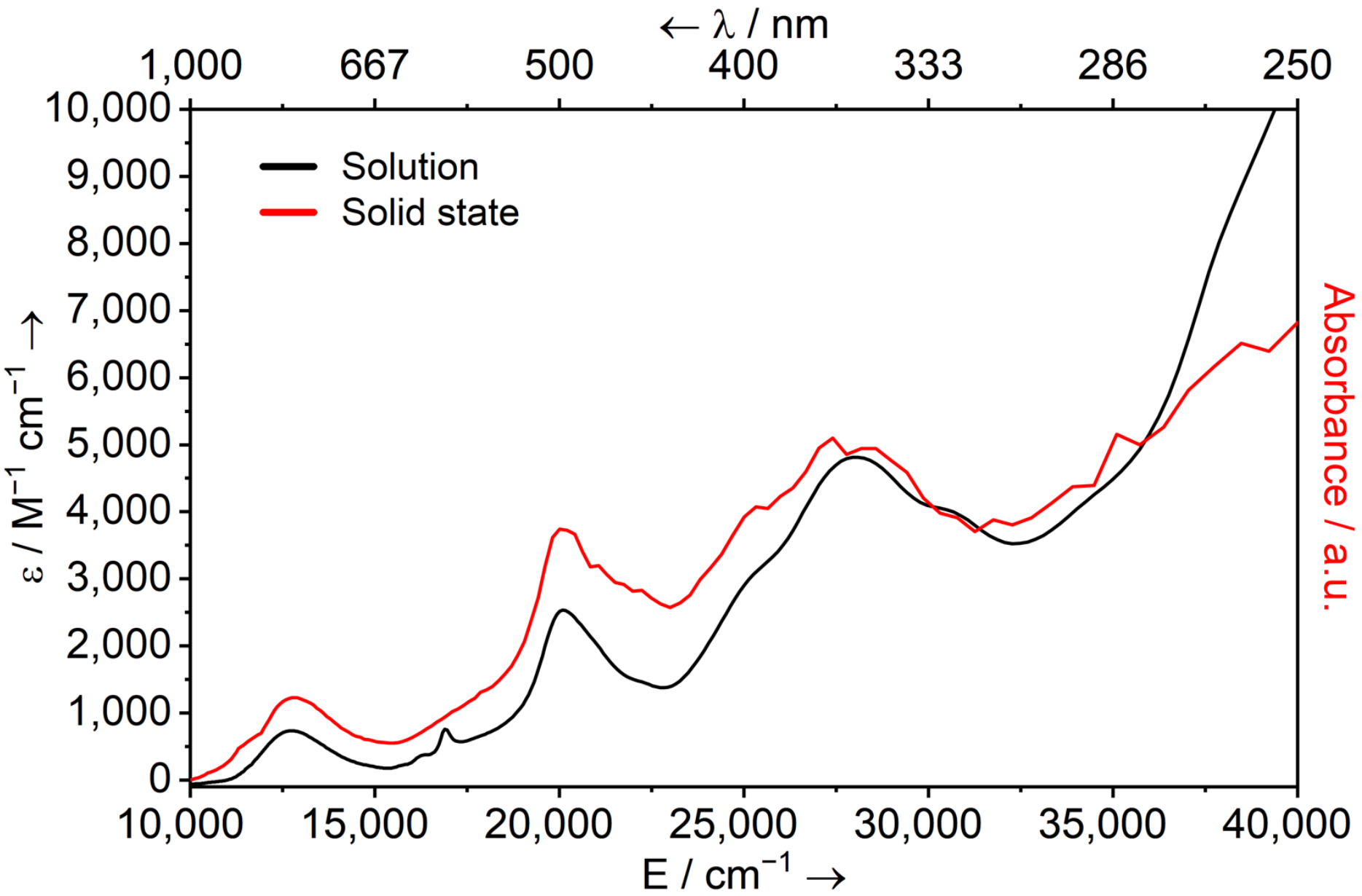

Figure S146. Solid-state UV-Vis-NIR spectrum of **6** (red) and solution UV-Vis-NIR spectrum of **6** (black) shown between 10,000–40,000 cm^{-1} (1,000–250 nm) at ambient temperature.

S6. Electrochemistry

Materials and general considerations

Electrochemical data were measured using an Ivium CompactStat.h20250. Data were collected using IviumSoft 4.1170 software in Advanced Mode. Measurements were performed using a three-electrode cell in an inert atmosphere glovebox under an atmosphere of argon with a glassy carbon working electrode (3 mm diameter, BASi MF-2012), a platinum wire counter electrode (7.5 cm length, BASi MW-1032), and a platinum wire auxiliary reference electrode (7.5 cm length, BASi MW-1032) at ambient temperature. The cell comprises a carved PTFE cap atop a 20 mL borosilicate glass scintillation vial (**Figure S147**). The cap holds electrodes securely in place and minimises solvent evaporation during measurement.

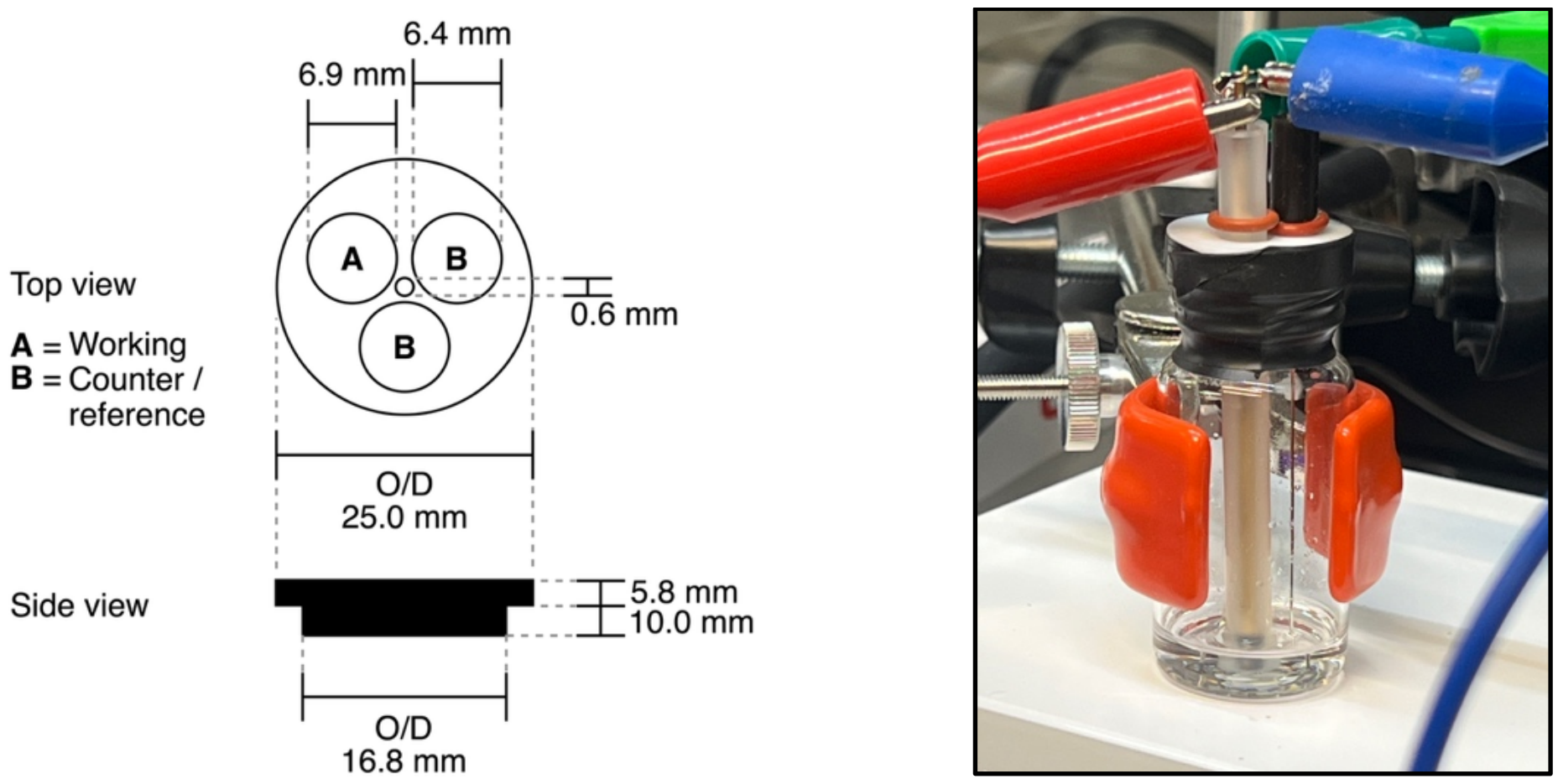

Figure S147. Custom PTFE cap used for the electrochemical cell, and an image of the cell.

The working electrode surface was polished prior to each set of experiments and cleaned between each scan. This was done to prevent the buildup of oxidized product on the electrode surfaces. All data were collected in a positive-feedback IR compensation mode. Prior to each scan, the open-circuit potential (OCP) and internal resistance (*i*R) were each recorded for 10 seconds, and the mean was used to calibrate each experiment. Measurements were performed using 0.2 M electrolyte concentration and 3.0 mM analyte concentration. All data is internally referenced to the $[Fc]^{+/0}$ couple by adding *ca.* 1 mg of Fc (*ca.* 3 mM) to solutions at the end of the experiment collection, unless otherwise stated. Small deviations in reported $E_{1/2}$ values between scans arise from the 20 mV step used in the experiment. While internal referencing to the $[Fc^*]^{+/0}$ couple is preferred due to the somewhat improved electrochemical characteristics compared to that of [Fc][21,22], the $[Fc^*]^{+/0}$ couple overlaps with data for some of the complexes herein. Furthermore, bulk samples of **3** and **6** react with Fc* to, to give an intractable mixture of products, and so its use as an internal reference would be inappropriate.

The U(IV) complexes **1** and **4** were found to be poorly soluble in *o*DFB, and so were studied only in THF using both [*n*Bu_4N][B$(C_6F_5)_4$] and [*n*Bu_4N][PF_6] supporting electrolytes. The U(V) complexes **3** and **6** are incompatible with THF and the [*n*Bu_4N][PF_6] electrolyte (see above), so both were only examined in *o*DFB with [*n*Bu_4N][B$(C_6F_5)_4$] supporting electrolyte.

*Data for **3**, **6**, and [Fe(Cp)$_2$] in oDFB supported by [nBu$_4$N][B(C$_6$F$_5$)$_4$]*

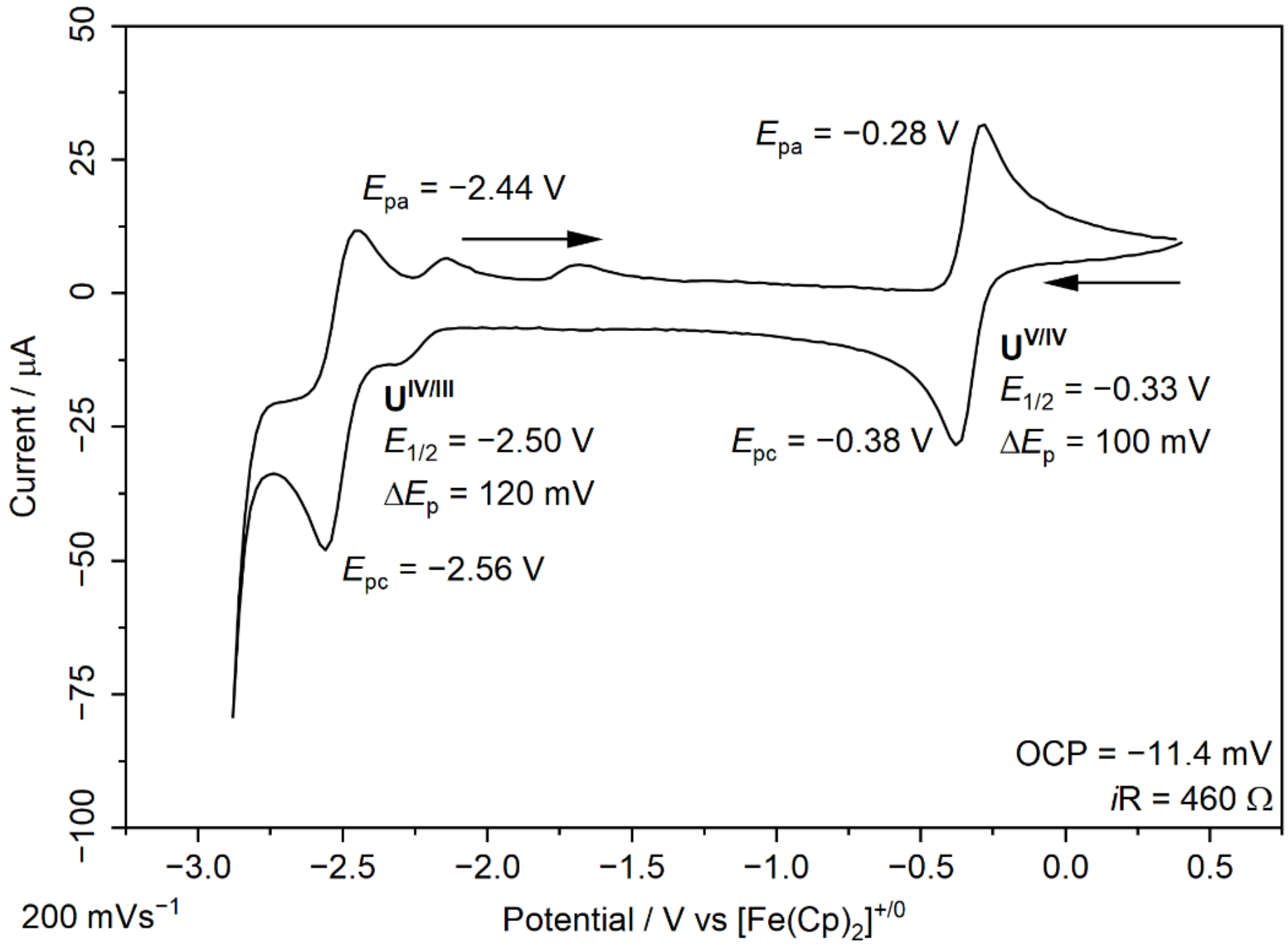

Figure S148. Cyclic voltammogram of **3** (3.0 mM) in *o*DFB supported by [*n*Bu$_4$N][B(C$_6$F$_5$)$_4$] (0.2 M) *vs* [Fc]$^{+/0}$ at 200 mV s^{-1}.

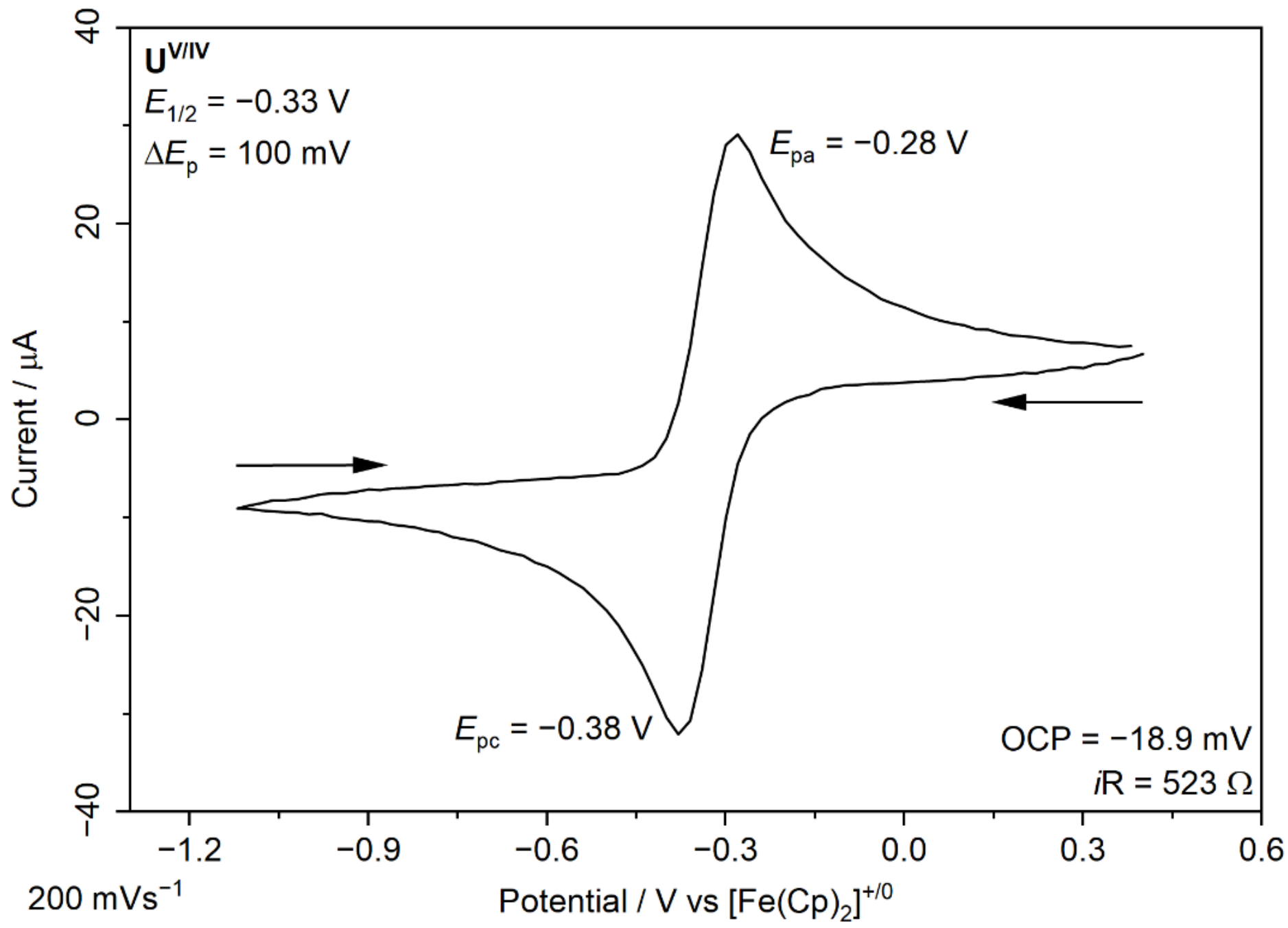

Figure S149. Cyclic voltammogram of the U(V/IV) couple of **3** (3.0 mM) in *o*DFB supported by [*n*Bu$_4$N][B(C$_6$F$_5$)$_4$] (0.2 M) *vs* [Fc]$^{+/0}$ at 200 mV s^{-1}.

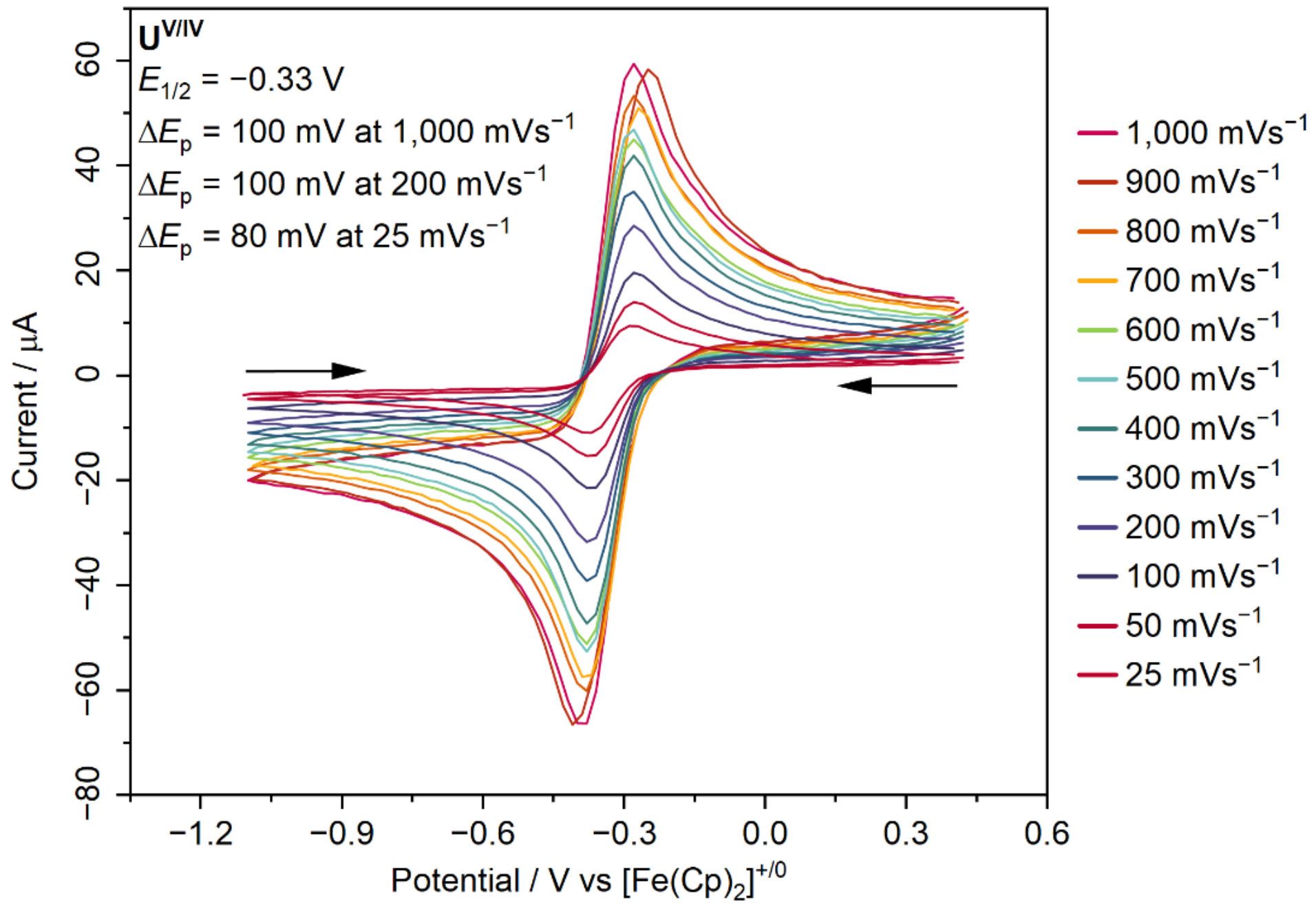

Figure S150. Cyclic voltammograms of the U(V/IV) couple of **3** (3.0 mM) in *o*DFB supported by [*n*Bu_4N][B(C_6F_5)$_4$] (0.2 M) *vs* $[Fc]^{+/0}$ showing the scan-rate dependence of the U(V/IV) couple.

Table S12. Electrochemical data for the U(V/IV) redox event *vs* $[Fc]^{+/0}$ for **3** (3.0 mM) in *o*DFB at room temperature, measured in 0.2 M [*n*Bu_4N][B(C_6F_5)$_4$].

Scan rate / mV s^{-1}	E_{pc} / V	E_{pa} / V	ΔE_p / mV	i_{pa}/i_{pc}	OCP / mV	*i*R / Ω
**25**	–0.37	–0.29	80	1.01	–9.54	576
**50**	–0.38	–0.28	100	1.01	–8.25	604
**100**	–0.38	–0.28	100	0.98	–8.70	550
**200**	–0.38	–0.28	100	0.99	–6.99	620
**300**	–0.38	–0.28	100	1.06	–2.53	531
**400**	–0.38	–0.28	100	0.98	–6.29	571
**500**	–0.38	–0.28	100	1.03	–4.20	535
**600**	–0.38	–0.28	100	1.02	–4.98	517
**700**	–0.39	–0.27	120	1.05	–3.33	632
**800**	–0.38	–0.28	100	1.05	–3.90	559
**900**	–0.41	–0.25	160	1.02	–1.91	894
**1,000**	–0.38	–0.28	100	1.04	–4.08	558

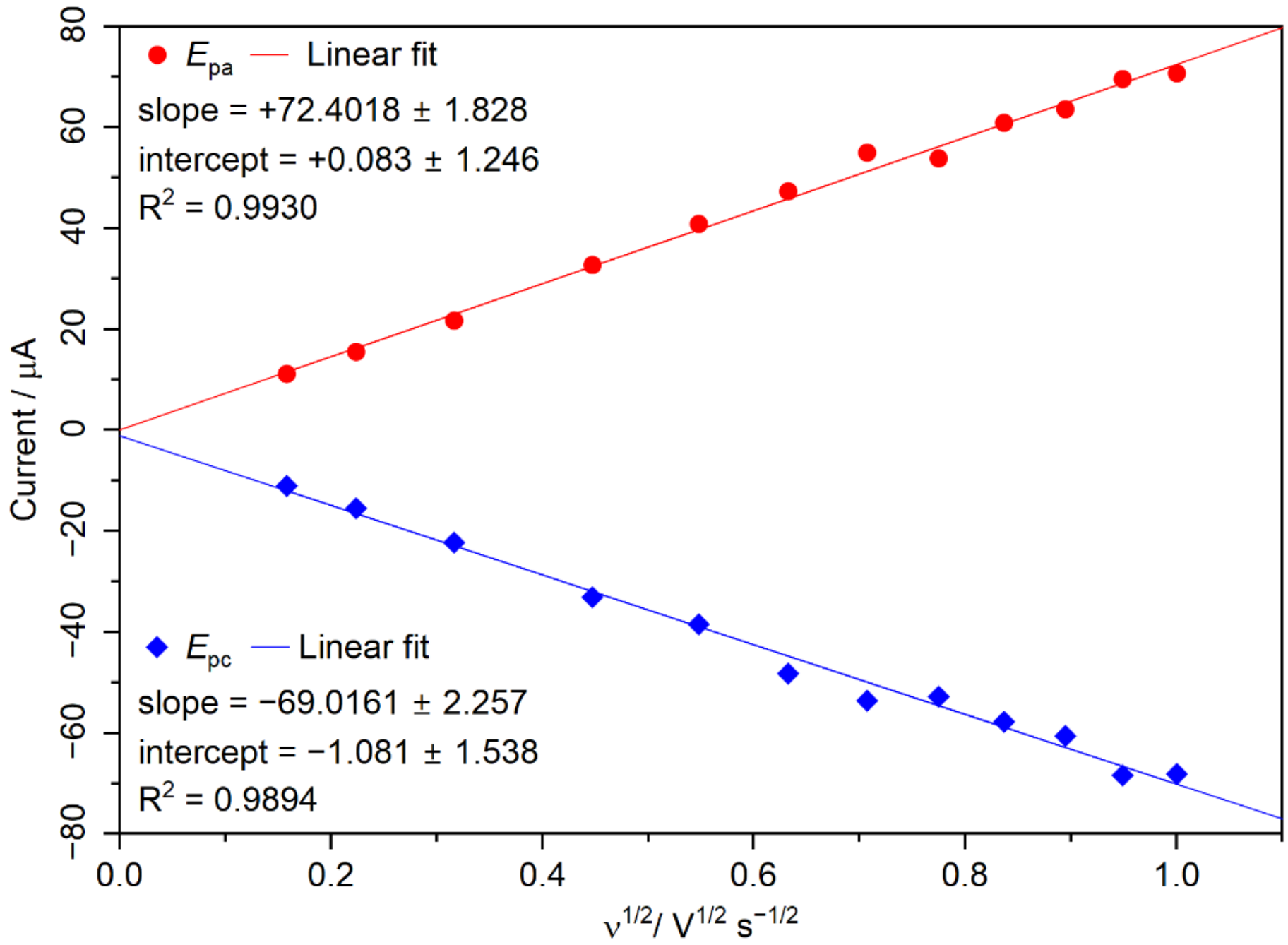

Figure S151. Randles-Sevcik plot for the U(V/IV) couple in **3**, measured in *o*DFB supported $[nBu_4N][B(C_6F_5)_4]$ (0.2 M).

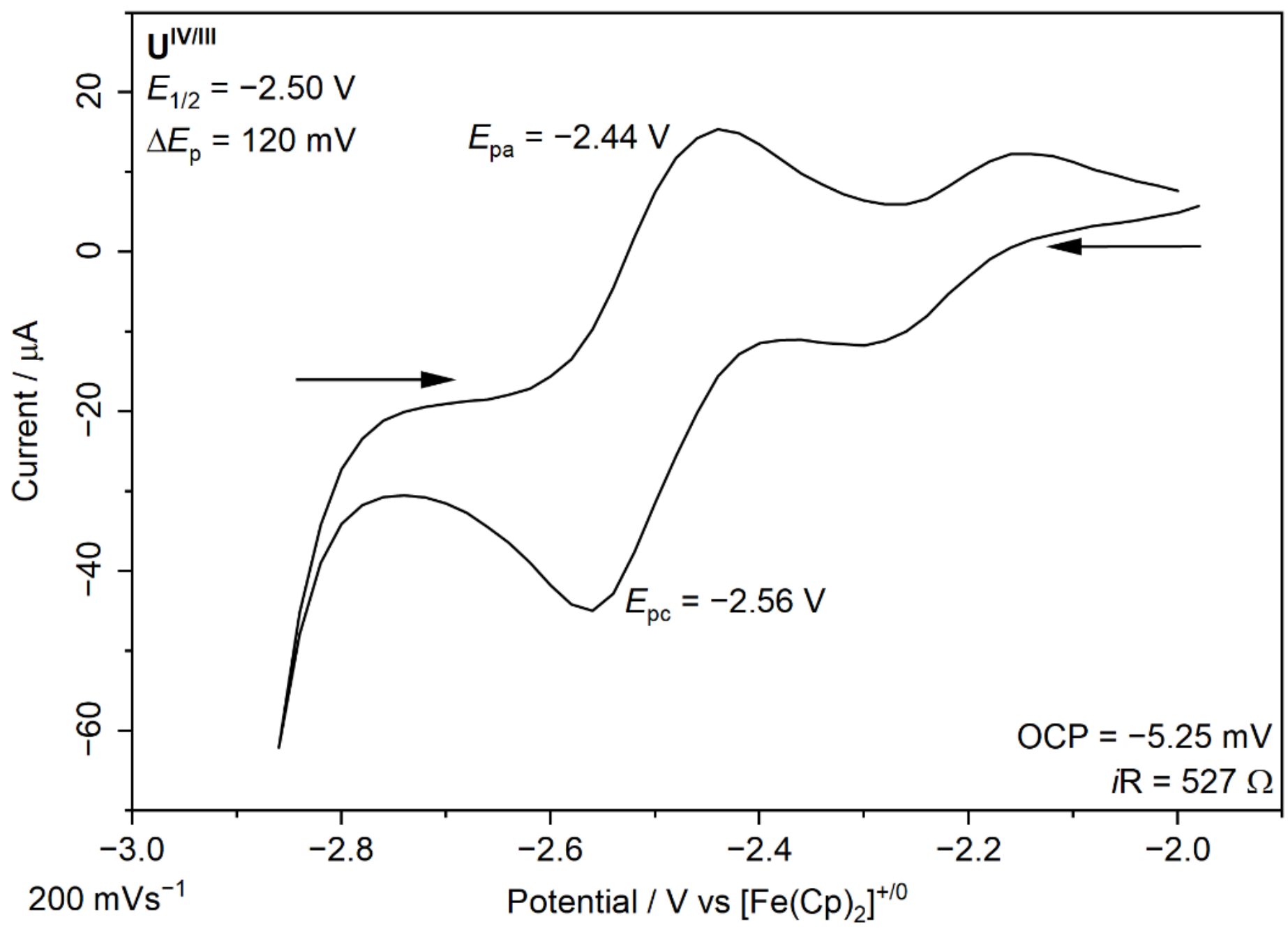

Figure S152. Cyclic voltammogram of the U(IV/III) couple of **3** (3.0 mM) in *o*DFB supported by $[nBu_4N][B(C_6F_5)_4]$ (0.2 M) *vs* $[Fc]^{+/0}$ at 200 mV s^{-1}.

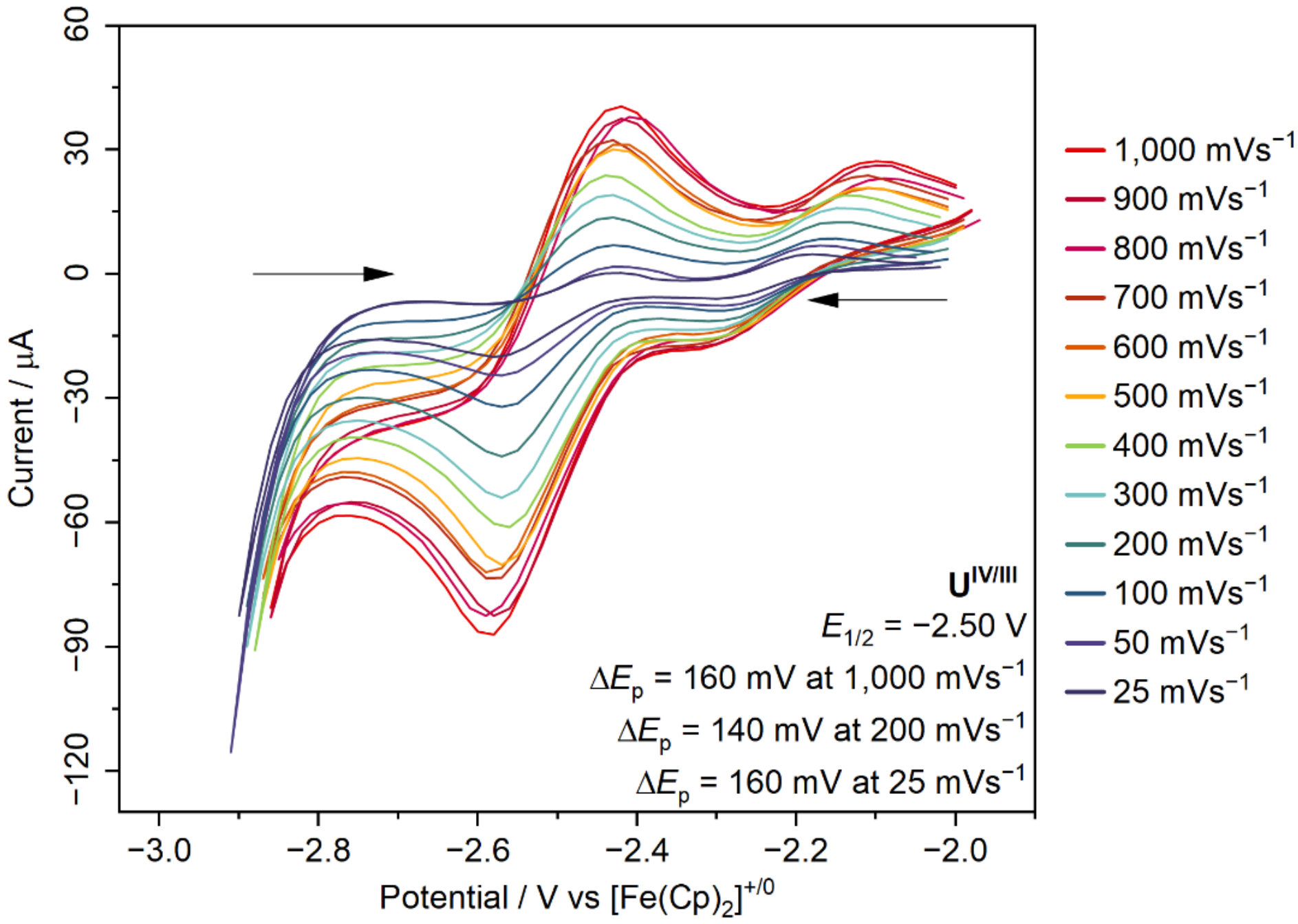

Figure S153. Cyclic voltammograms of the U(IV/III) couple of **3** (3.0 mM) in *o*DFB supported by [*n*Bu_4N][$B(C_6F_5)_4$] (0.2 M) *vs* $[Fc]^{+/0}$ showing the scan-rate dependence of the U(IV/III) couple.

Table S13. Electrochemical data for the U(IV/III) redox event *vs* $[Fc]^{+/0}$ for **3** (3.0 mM) in *o*DFB at room temperature, measured in 0.2 M [*n*Bu_4N][$B(C_6F_5)_4$].

Scan rate / mV s^{-1}	E_{pc} / V	E_{pa} / V	ΔE_p / mV	i_{pa}/i_{pc}	OCP / mV	*i*R / Ω
**25**	–2.58	–2.42	160	0.54	–36.2	598
**50**	–2.57	–2.43	140	0.49	–53.8	521
**100**	–2.57	–2.43	140	0.75	–40.5	601
**200**	–2.57	–2.43	140	0.86	–43.6	600
**300**	–2.57	–2.43	140	0.92	–42.2	582
**400**	–2.56	–2.44	120	1.00	–36.8	536
**500**	–2.57	–2.43	140	0.99	–37.5	667
**600**	–2.59	–2.41	180	1.03	–38.2	795
**700**	–2.59	–2.41	160	1.08	–29.8	602
**800**	–2.59	–2.41	180	1.04	–31.9	895
**900**	–2.58	–2.42	160	1.06	–33.2	682
**1,000**	–2.58	–2.42	160	1.05	–29.3	705

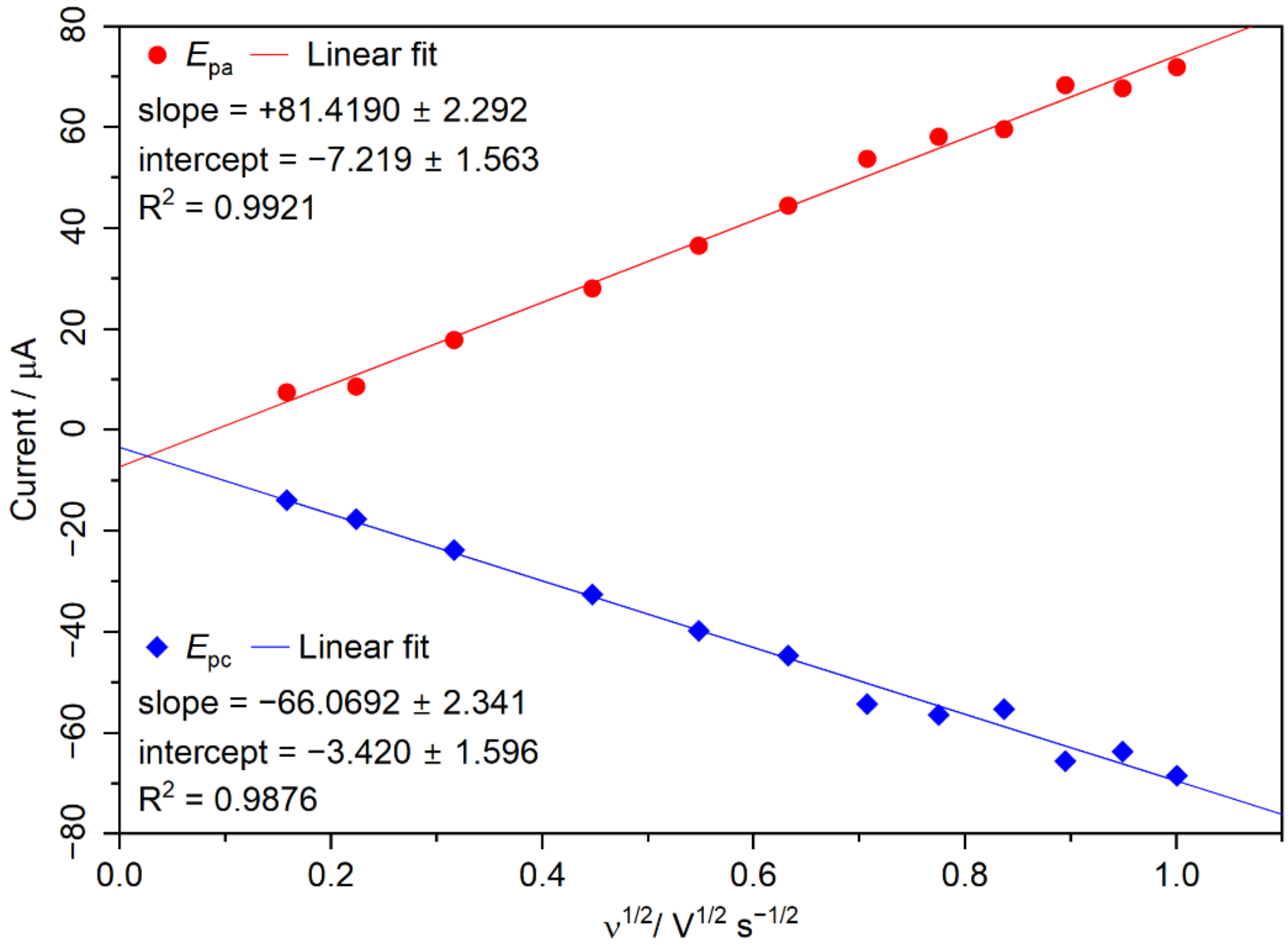

Figure S154. Randles-Sevcik plot for the U(IV/III) couple in **3**, measured in *o*DFB supported by [nBu$_4$N][B(C$_6$F$_5$)$_4$] (0.2 M).

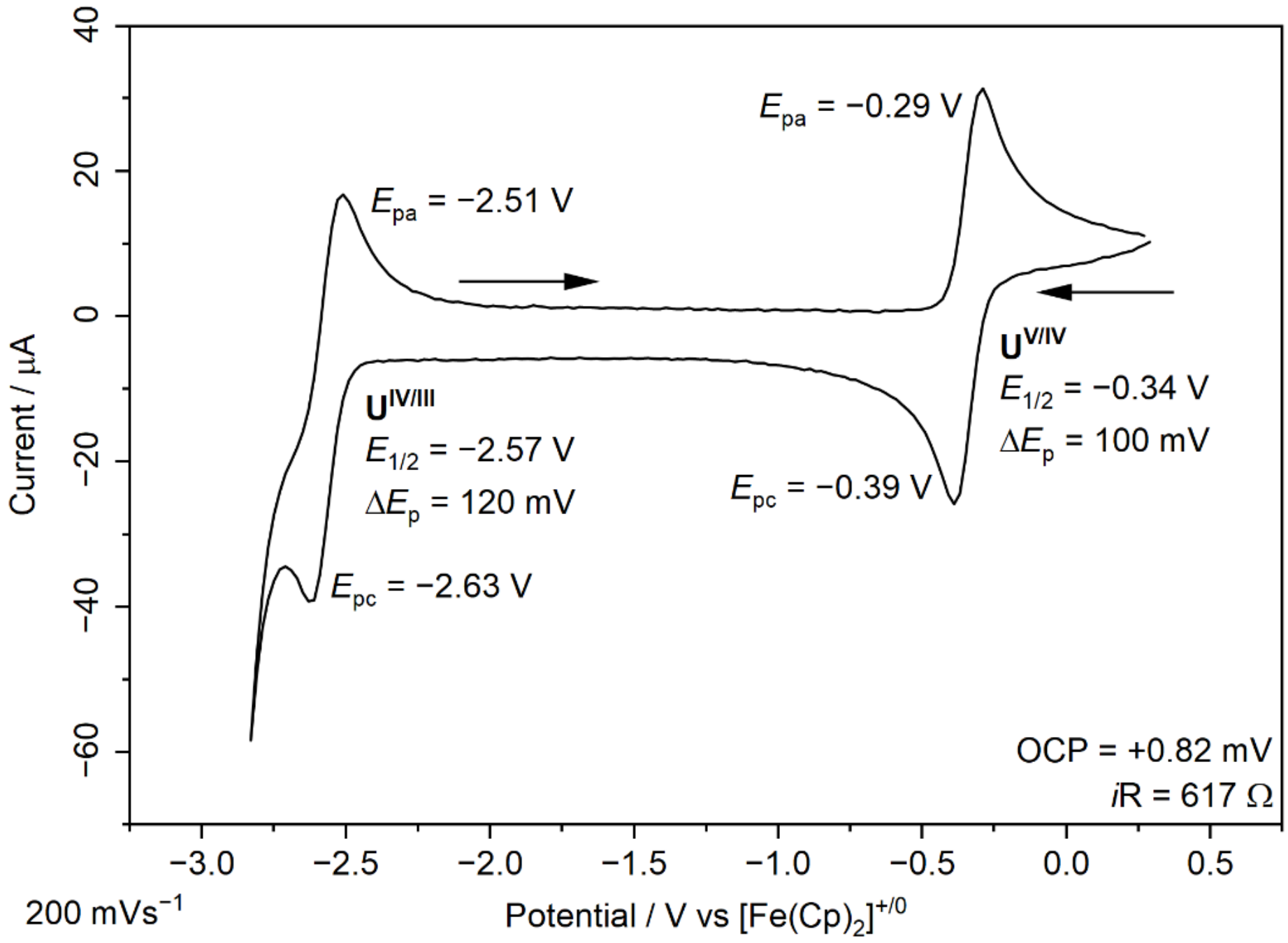

Figure S155. Cyclic voltammogram of **6** (3.0 mM) in *o*DFB supported by [nBu$_4$N][B(C$_6$F$_5$)$_4$] (0.2 M) *vs* $[Fc]^{+/0}$ at 200 mV s^{-1}.

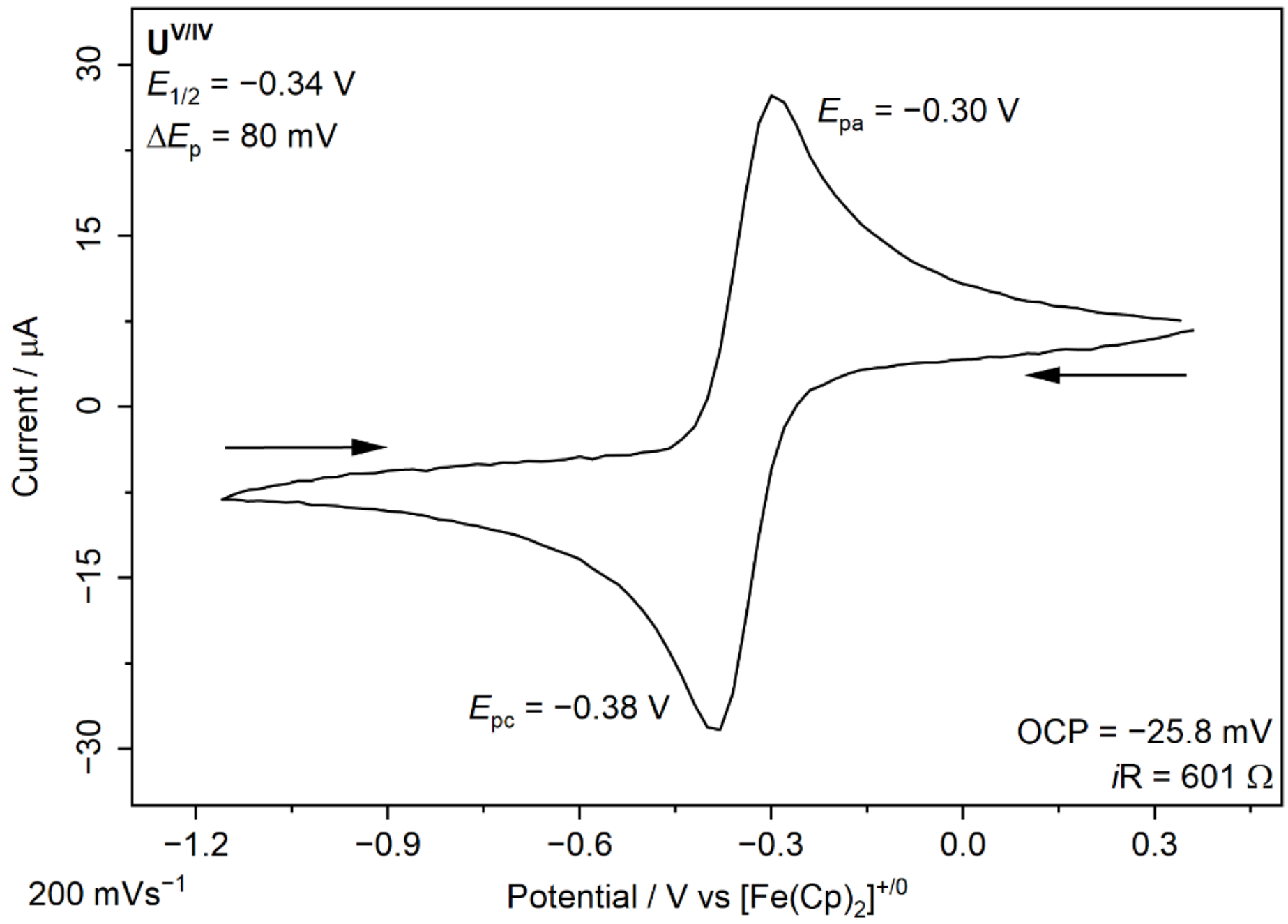

Figure S156. Cyclic voltammogram of the U(V/IV) couple of **6** (3.0 mM) in *o*DFB supported by [nBu$_4$N][B(C$_6$F$_5$)$_4$] (0.2 M) *vs* [Fc]$^{+/0}$ at 200 mV s^{-1}.

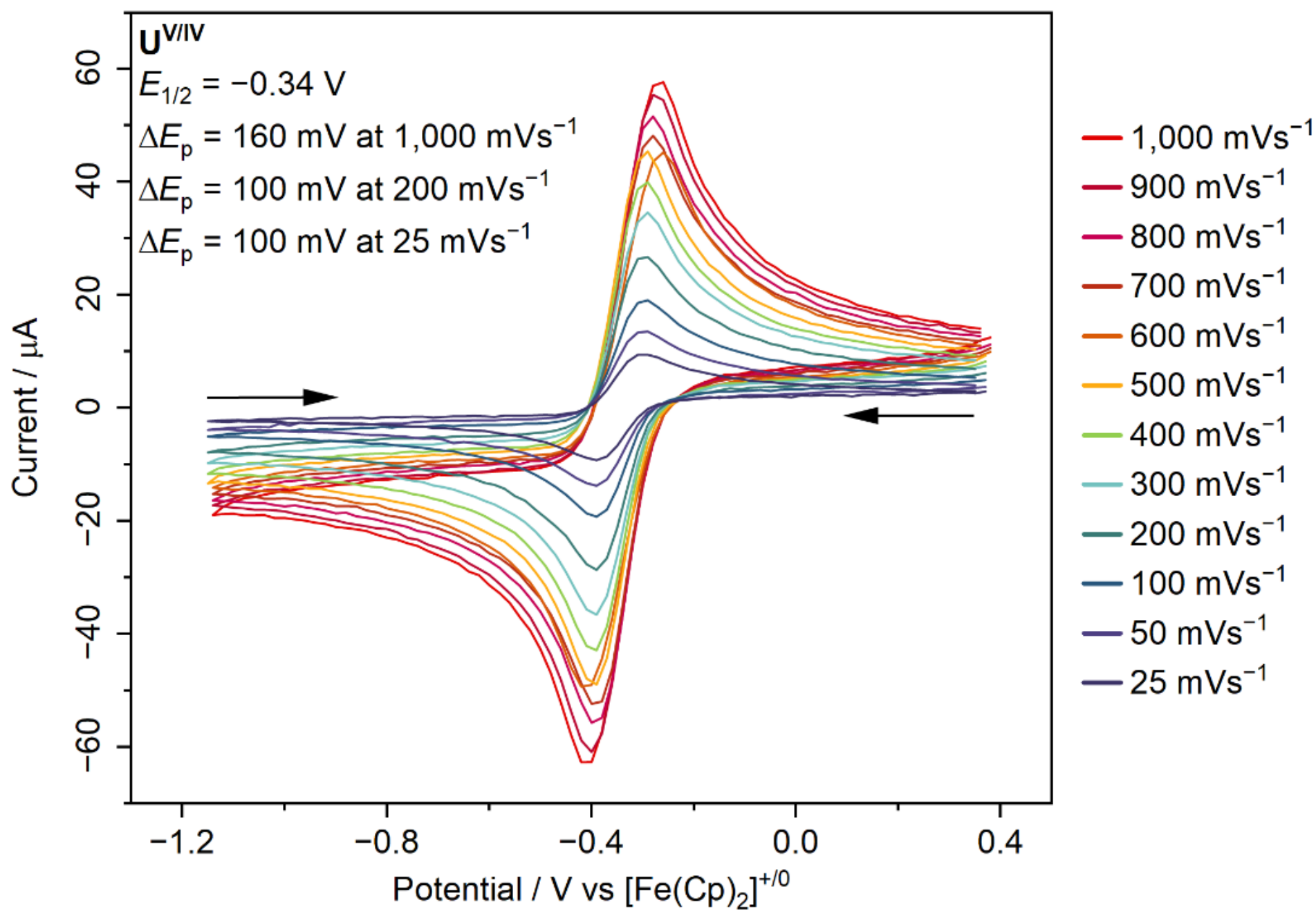

Figure S157. Cyclic voltammograms of the U(V/IV) couple of **6** (3.0 mM) in *o*DFB supported by [nBu$_4$N][B(C$_6$F$_5$)$_4$] (0.2 M) *vs* [Fc]$^{+/0}$ showing the scan-rate dependence of the U(V/IV) couple.

Table S14. Electrochemical data for the U(V/IV) redox event *vs* $[Fc]^{+/0}$ for **6** (3.0 mM) in *o*DFB at room temperature, measured in 0.2 M $[nBu_4N][B(C_6F_5)_4]$.

Scan rate / mV s^{-1}	E_{pc} / V	E_{pa} / V	ΔE_p / mV	i_{pa}/i_{pc}	OCP / mV	*i*R / Ω
**25**	–0.39	–0.29	100	0.99	–5.07	615
**50**	–0.39	–0.29	100	1.01	–5.56	665
**100**	–0.39	–0.29	100	0.99	–4.66	676
**200**	–0.39	–0.29	100	1.02	–3.65	616
**300**	–0.39	–0.29	100	1.03	–3.18	708
**400**	–0.39	–0.29	100	1.01	–1.62	650
**500**	–0.39	–0.29	100	1.01	–1.12	610
**600**	–0.42	–0.26	160	1.03	–1.18	992
**700**	–0.40	–0.28	120	1.00	+1.03	626
**800**	–0.40	–0.28	120	1.00	+1.64	616
**900**	–0.40	–0.28	120	1.00	+2.03	733
**1,000**	–0.42	–0.26	160	1.01	+3.44	804

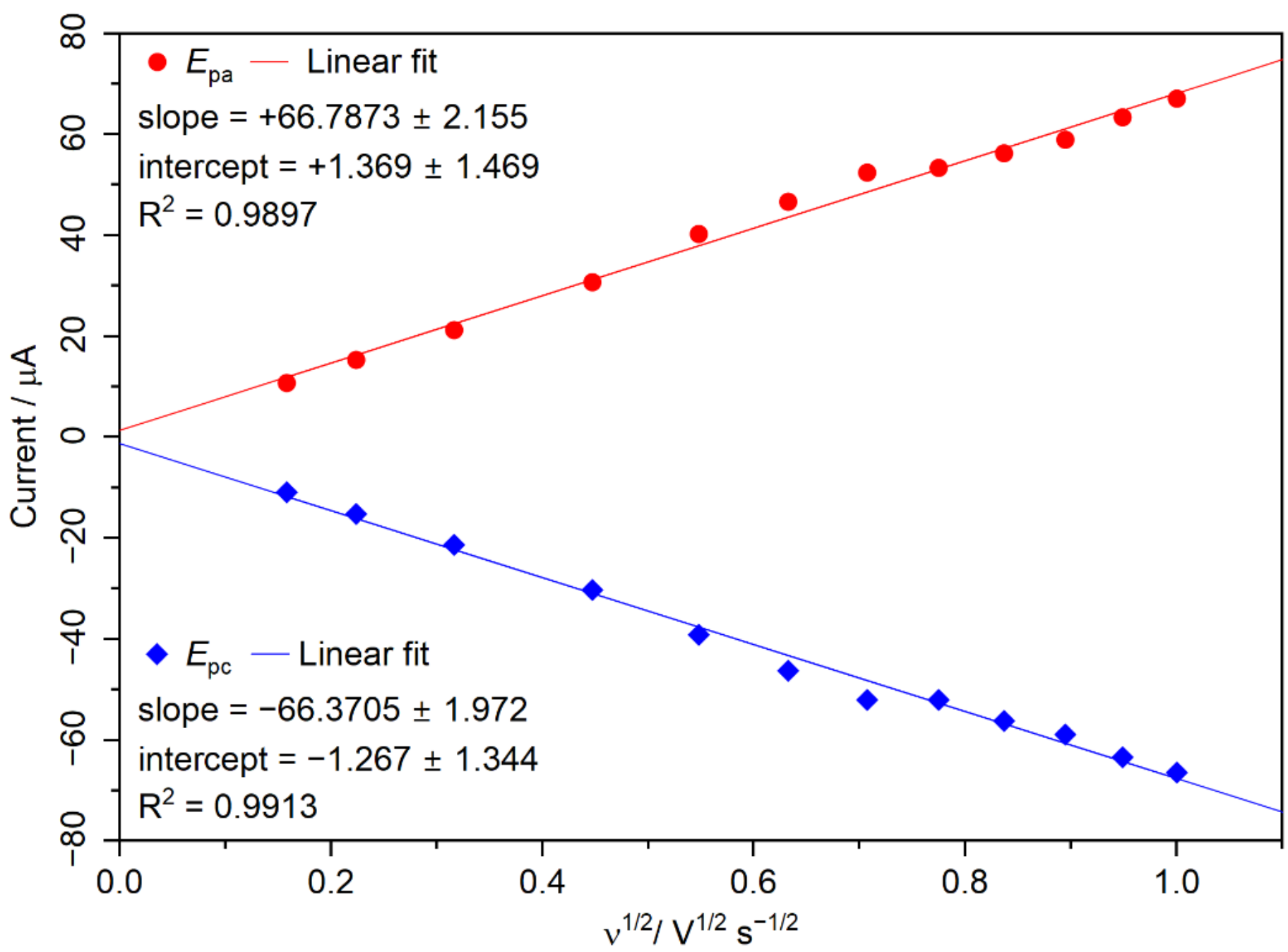

Figure S158. Randles-Sevcik plot for the U(V/IV) couple in **6**, measured in *o*DFB supported by $[nBu_4N][B(C_6F_5)_4]$ (0.2 M).

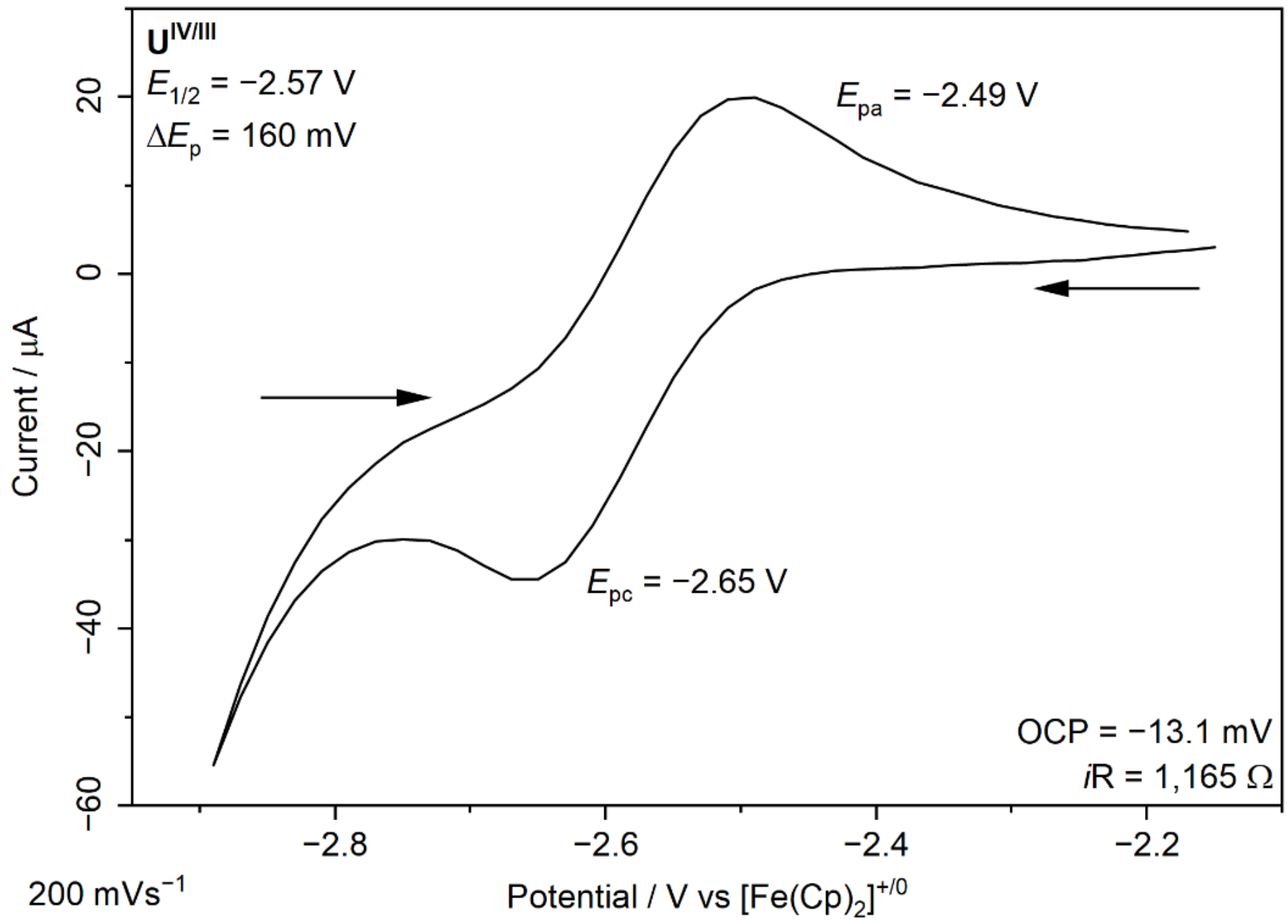

Figure S159. Cyclic voltammogram of the U(IV/III) couple of **3** (3.0 mM) in *o*DFB supported by $[nBu_4N][B(C_6F_5)_4]$ (0.2 M) *vs* $[Fc]^{+/0}$ at 200 mV s^{-1}.

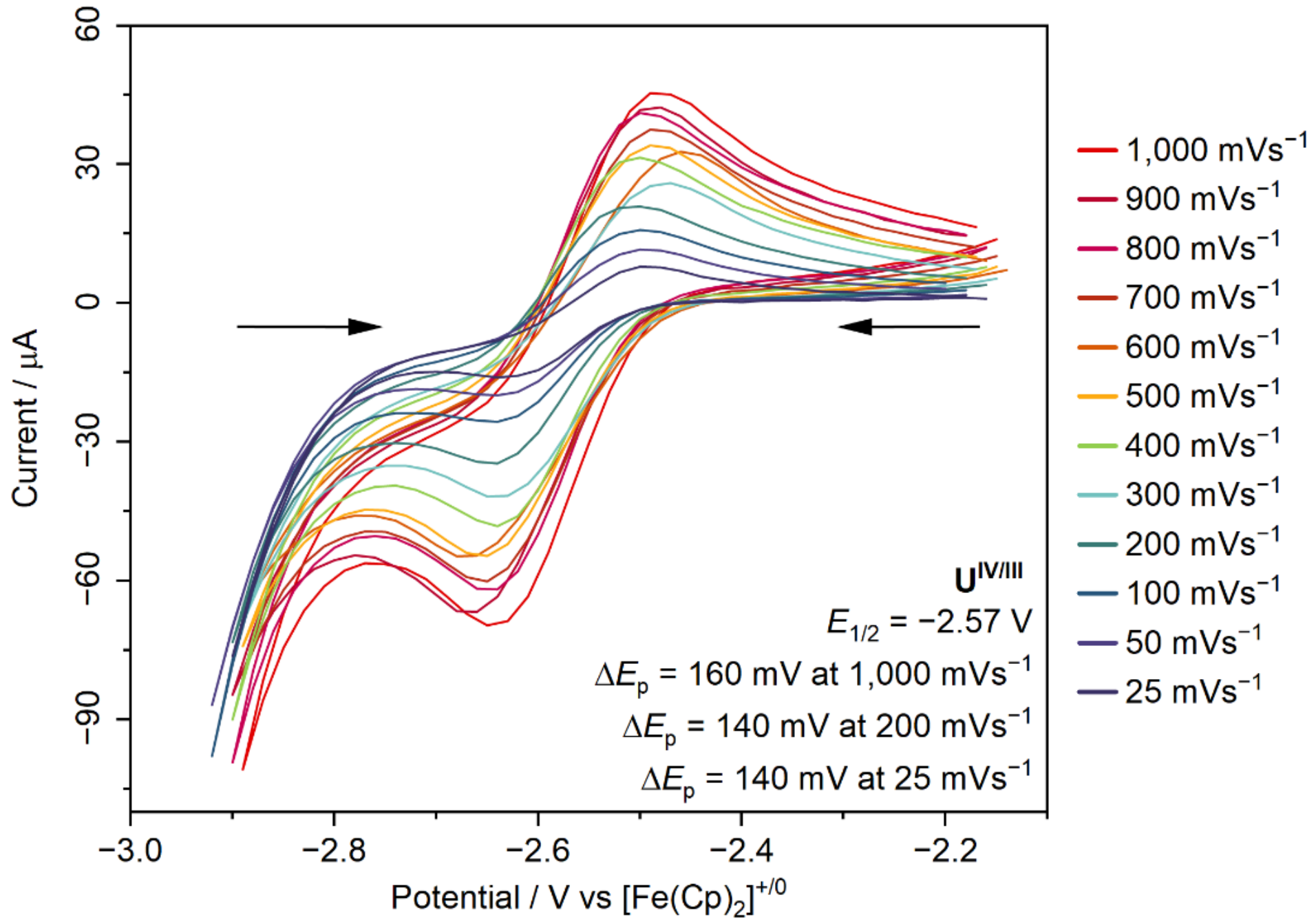

Figure S160. Cyclic voltammograms of the U(IV/III) couple of **6** (3.0 mM) in *o*DFB supported by $[nBu_4N][B(C_6F_5)_4]$ (0.2 M) *vs* $[Fc]^{+/0}$ showing the scan-rate dependence of the U(IV/III) couple.

Table S15. Electrochemical data for the U(IV/III) redox event *vs* $[Fc]^{+/0}$ for **6** (3.0 mM) in *o*DFB at room temperature, measured in 0.2 M $[nBu_4N][B(C_6F_5)_4]$.

Scan rate / mV s^{-1}	E_{pc} / V	E_{pa} / V	ΔE_p / mV	i_{pa}/i_{pc}	OCP / mV	iR / Ω
**25**	–2.64	–2.50	140	0.93	–32.1	869
**50**	–2.64	–2.50	140	0.99	–45.3	845
**100**	–2.64	–2.50	140	1.03	–40.8	701
**200**	–2.64	–2.50	140	0.97	–17.4	777
**300**	–2.65	–2.47	180	0.97	–32.9	1,049
**400**	–2.64	–2.50	140	0.96	–22.4	696
**500**	–2.65	–2.49	160	0.97	–19.0	930
**600**	–2.68	–2.46	220	0.96	–24.6	1,295
**700**	–2.65	–2.49	160	0.96	–20.6	880
**800**	–2.64	–2.50	140	1.05	–26.4	696
**900**	–2.66	–2.48	180	1.01	–25.4	896
**1,000**	–2.65	–2.49	160	1.03	–27.0	718

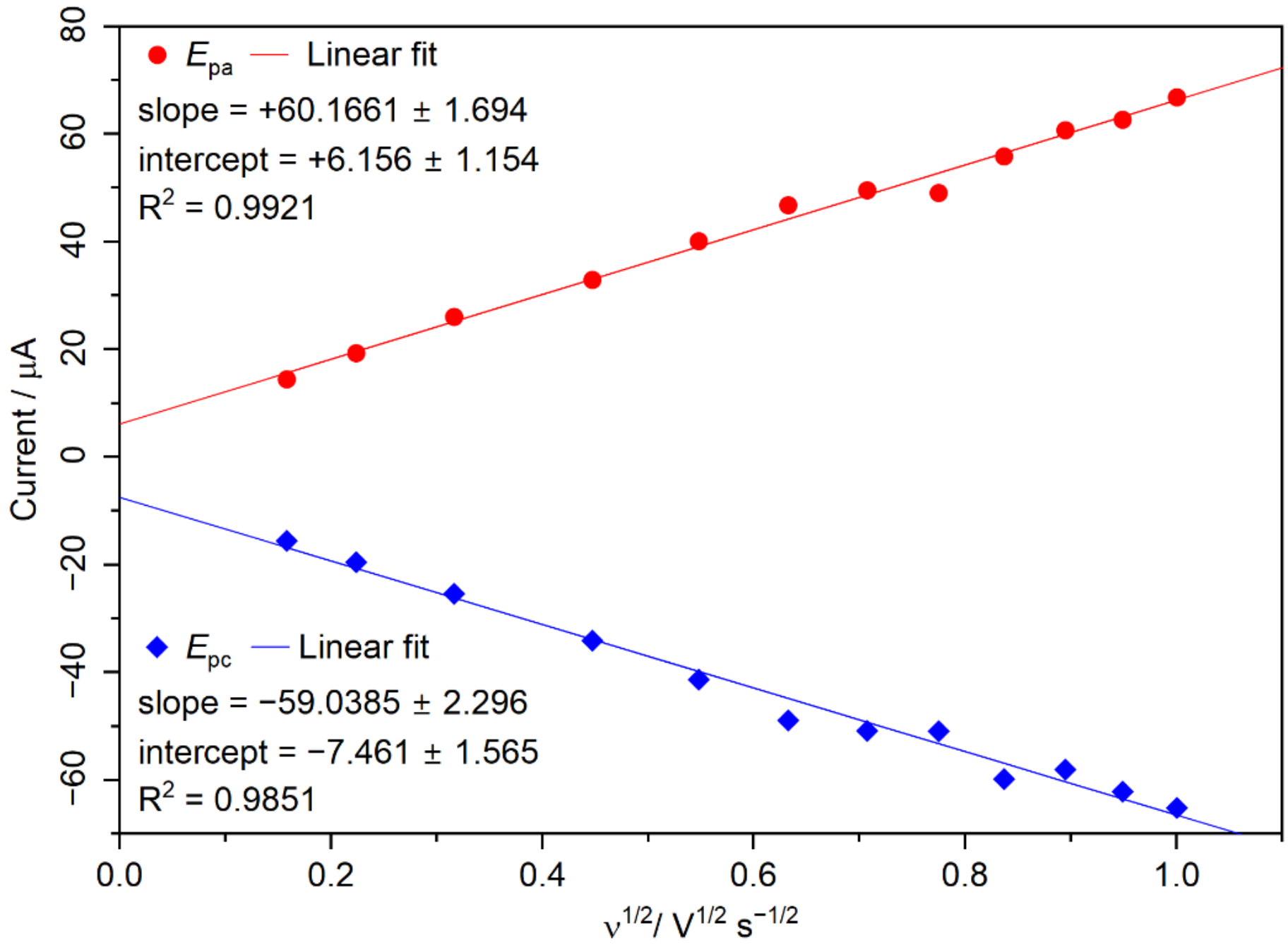

Figure S161. Randles-Sevcik plot for the U(IV/III) couple in **6**, measured in *o*DFB supported by $[nBu_4N][B(C_6F_5)_4]$ (0.2 M).

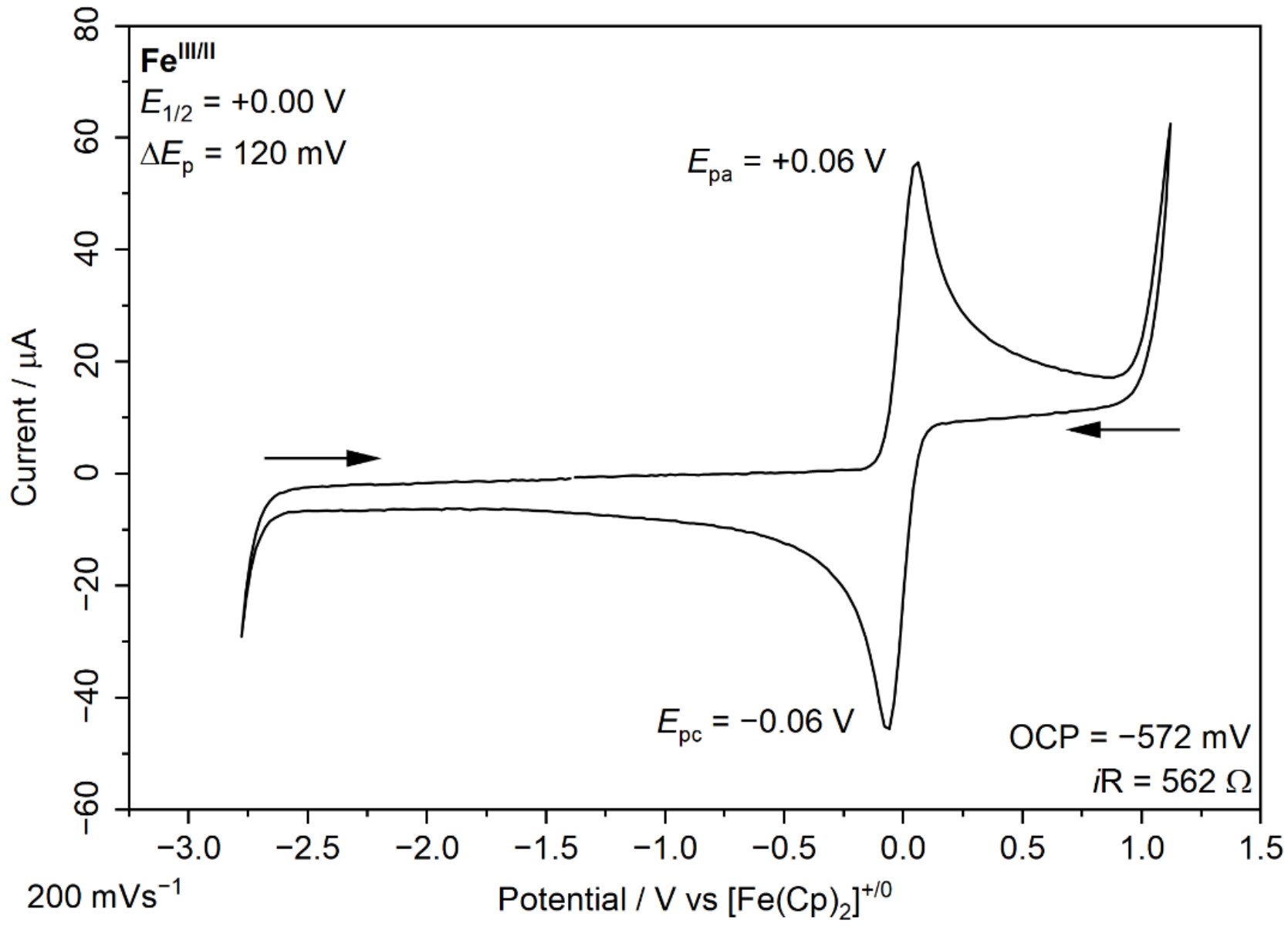

Figure S162. Top: Cyclic voltammogram of Fc (3.0 mM) in *o*DFB supported by [nBu$_4$N][B(C_6F_5)$_4$] (0.2 M) *vs* [Fc]$^{+/0}$ at 200 mV s^{-1}.

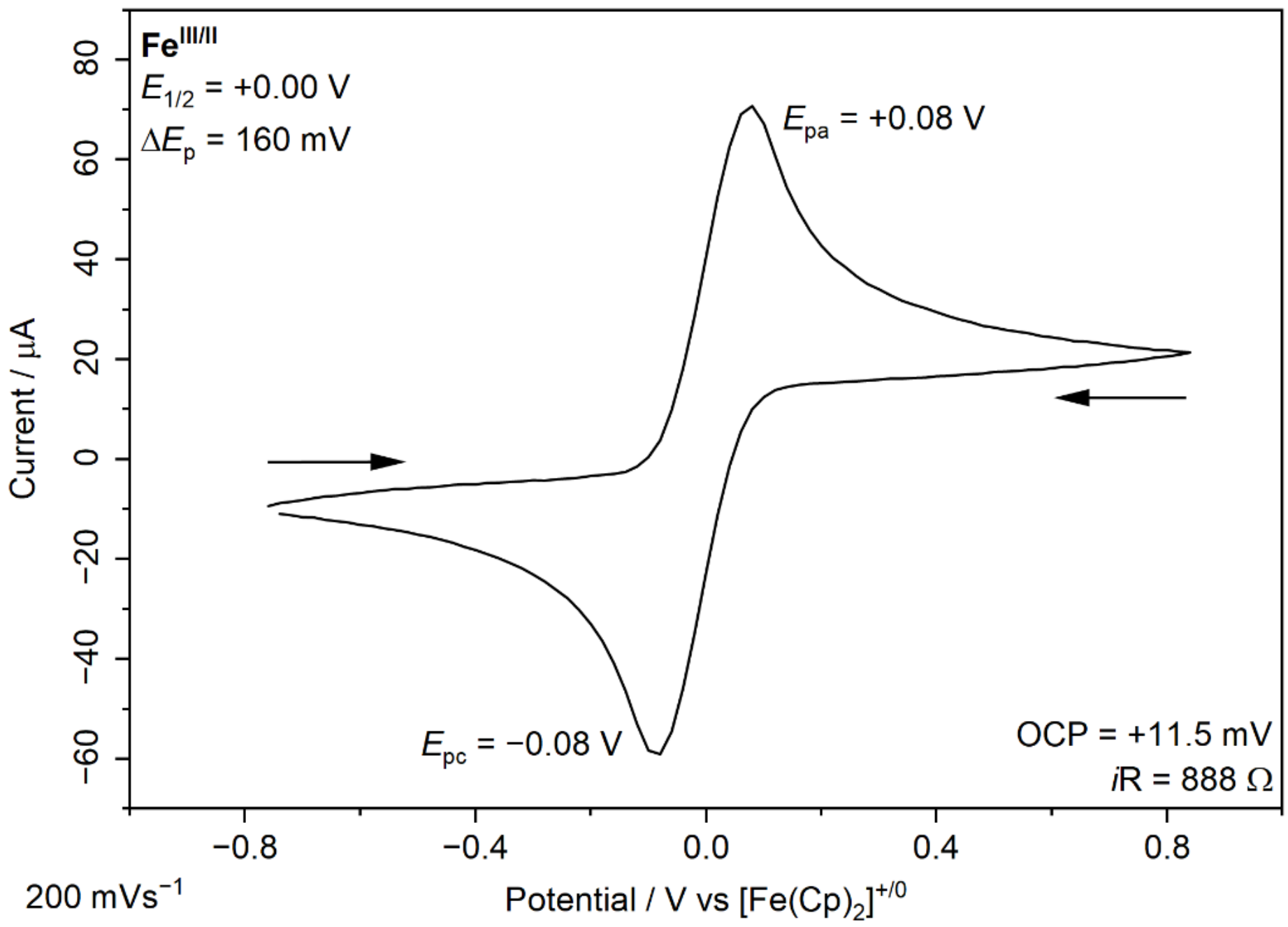

Figure S163. Cyclic voltammogram of the Fe(III/II) couple of Fc (3.0 mM) in *o*DFB supported by [nBu$_4$N][B(C_6F_5)$_4$] (0.2 M) *vs* [Fc]$^{+/0}$ at 200 mV s^{-1}.

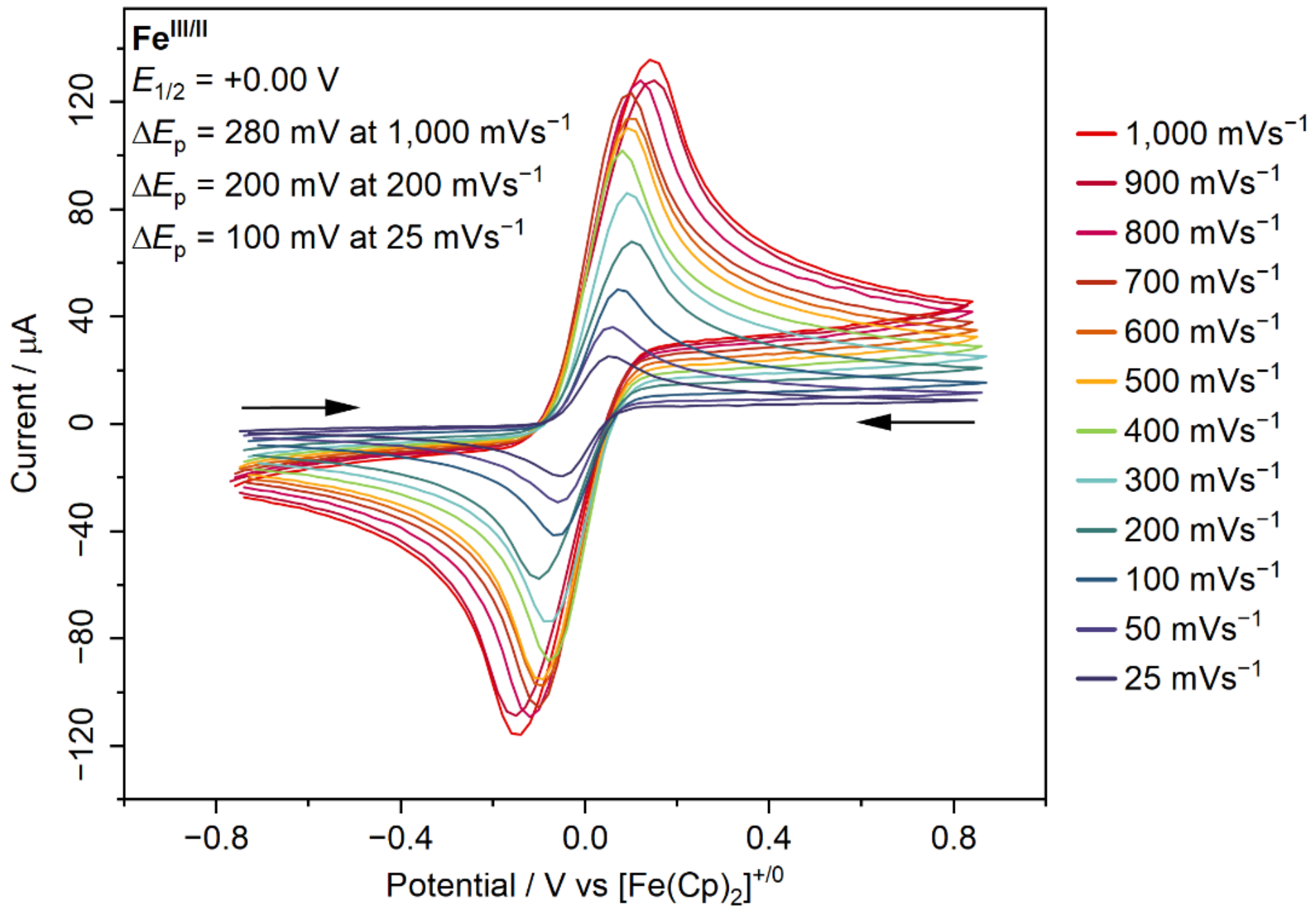

Figure S164. Cyclic voltammograms of the Fe(III/II) couple of Fc (3.0 mM) in *o*DFB supported by [*n*Bu_4N][$B(C_6F_5)_4$] (0.2 M) *vs* $[Fc]^{+/0}$ showing the scan-rate dependence of the Fe(III/II) couple.

Table S16. Electrochemical data for the Fe(III/II) redox event *vs* $[Fc]^{+/0}$ for Fc (3.0 mM) in *o*DFB at room temperature, measured in 0.2 M [*n*Bu_4N][$B(C_6F_5)_4$].

Scan rate / mV s^{-1}	E_{pc} / V	E_{pa} / V	ΔE_p / mV	i_{pa}/i_{pc}	OCP / mV	*i*R / Ω
**25**	–0.05	+0.05	100	0.97	+11.8	856
**50**	–0.06	+0.06	120	0.99	+14.3	798
**100**	–0.07	+0.07	140	1.01	+14.0	891
**200**	–0.10	+0.10	200	0.99	+11.8	1,172
**300**	–0.09	+0.09	180	1.01	+11.4	862
**400**	–0.08	+0.08	160	0.98	+5.66	719
**500**	–0.09	+0.09	180	1.01	+0.33	825
**600**	–0.09	+0.09	180	1.00	–1.97	819
**700**	–0.10	+0.10	200	1.00	–6.35	791
**800**	–0.12	+0.12	240	1.00	–8.98	901
**900**	–0.15	+0.15	300	0.99	–14.0	1,096
**1,000**	–0.14	+0.14	280	1.03	–5.36	1,029

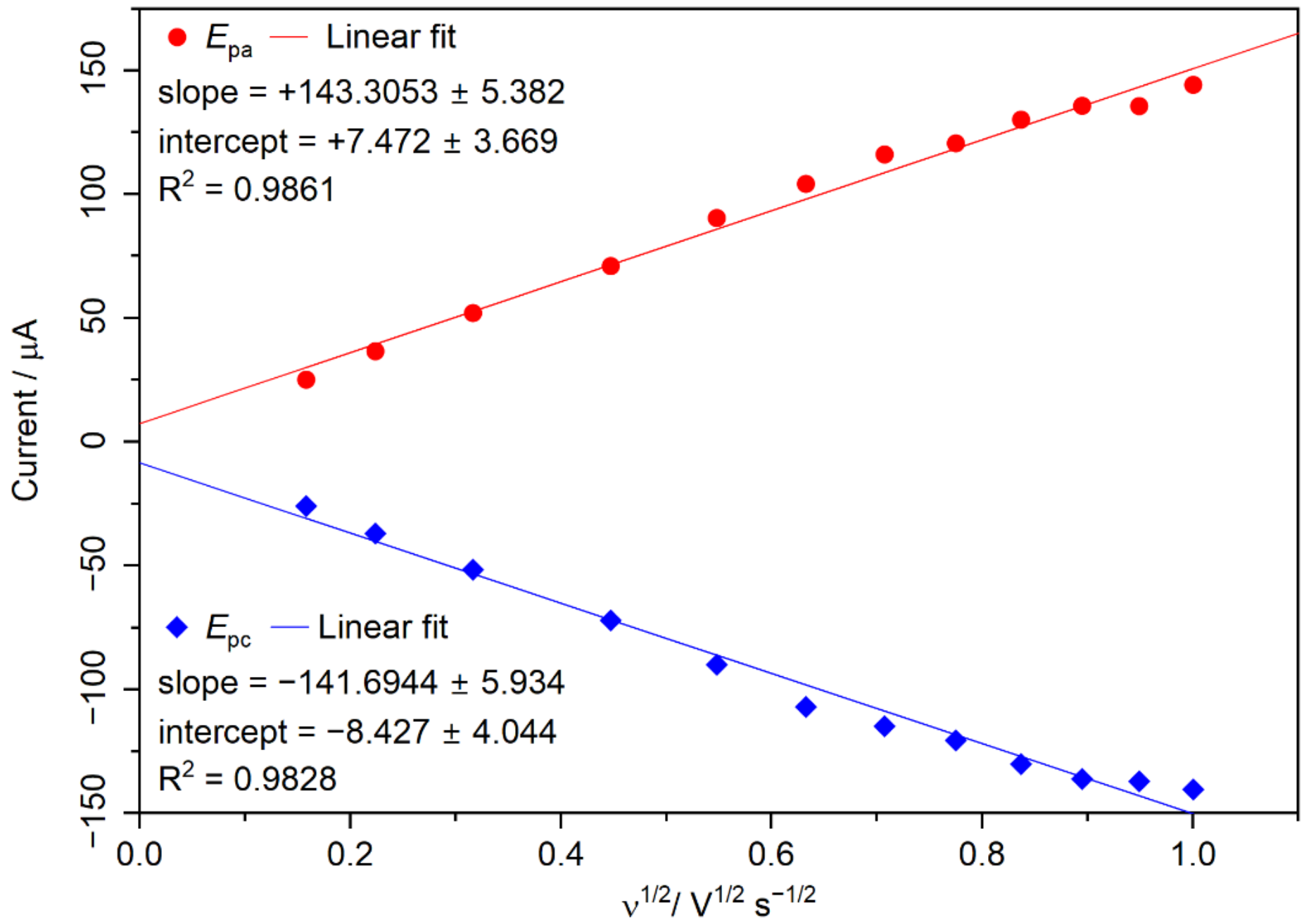

Figure S165. Randles-Sevcik plot for the Fe(III/II) couple in $[Fe(Cp)_2]$, measured in *o*DFB supported by $[n\mathrm{Bu}_4\mathrm{N}][\mathrm{B}(\mathrm{C}_6\mathrm{F}_5)_4]$ (0.2 M).

*Data for **1**, **4**, and [Fe(Cp)$_2$] in THF supported by [nBu$_4$N][B(C$_6$F$_5$)$_4$]*

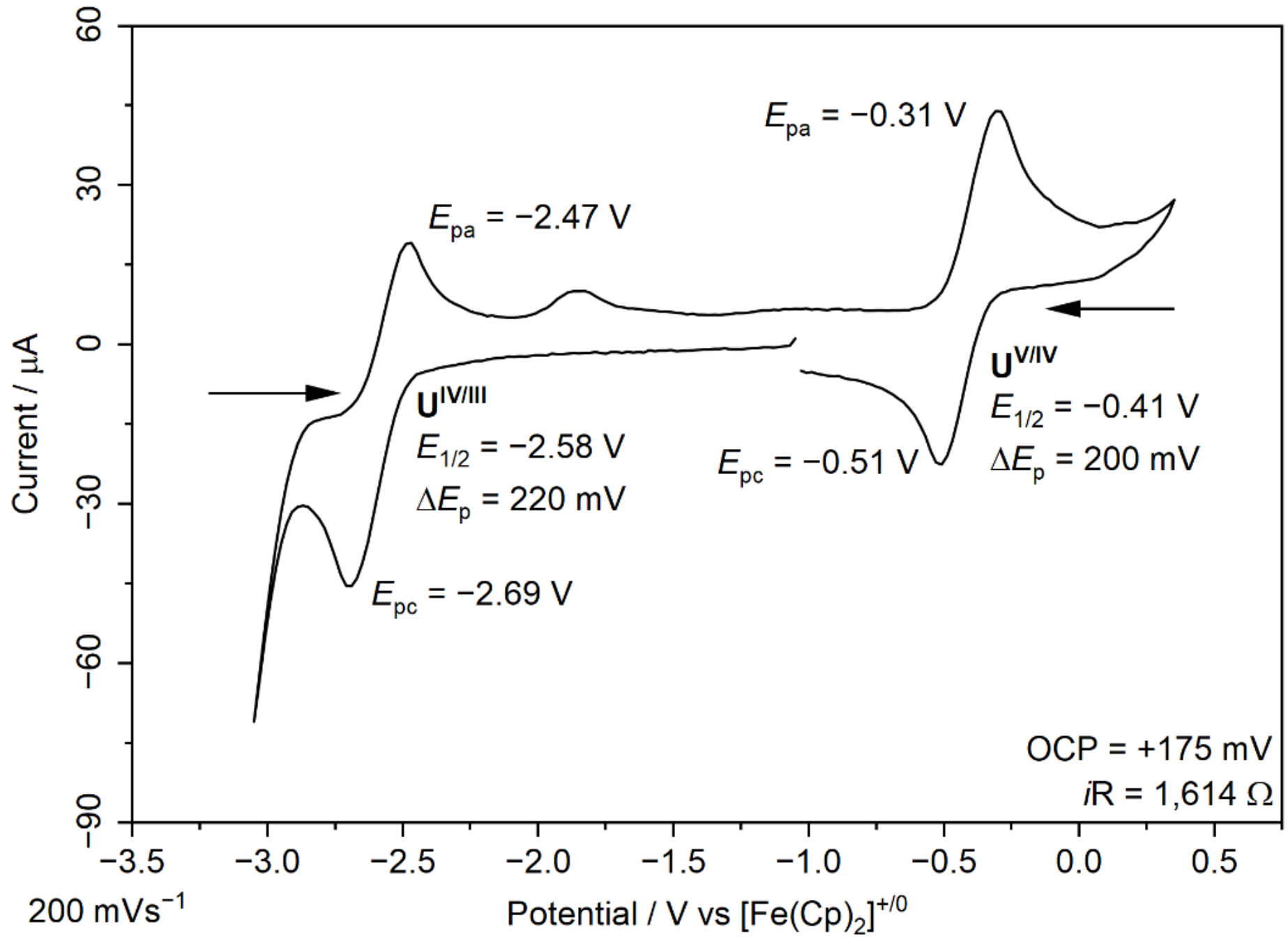

Figure S166. Cyclic voltammogram of **1** (3.0 mM) in THF supported by [*n*Bu$_4$N][B(C$_6$F$_5$)$_4$] (0.2 M) *vs* $[Fc]^{+/0}$ at 200 mV s^{-1}.

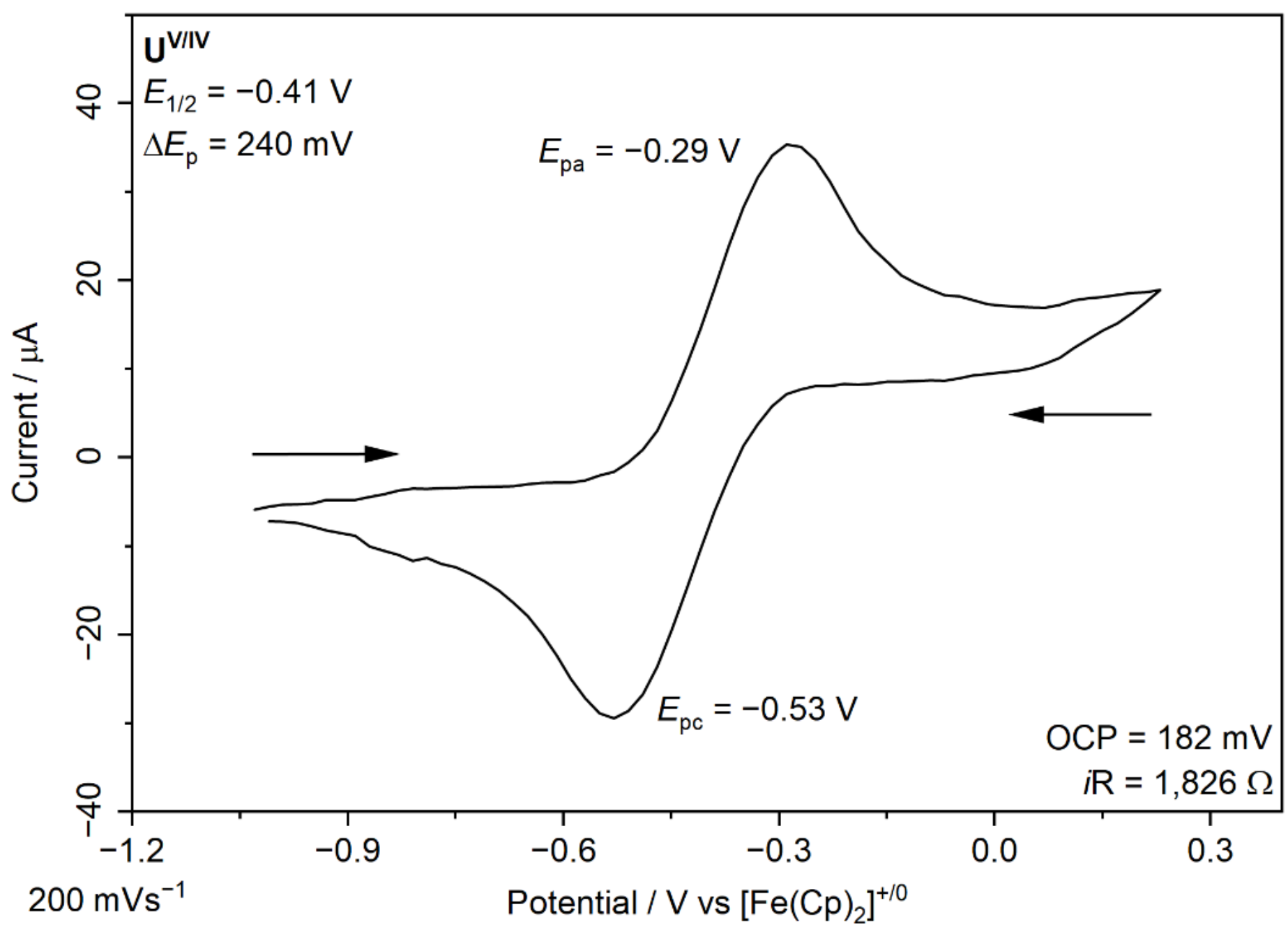

Figure S167. Cyclic voltammogram of the U(V/IV) couple of **1** (3.0 mM) in THF supported by [*n*Bu$_4$N][B(C$_6$F$_5$)$_4$] (0.2 M) *vs* $[Fc]^{+/0}$ at 200 mV s^{-1}.

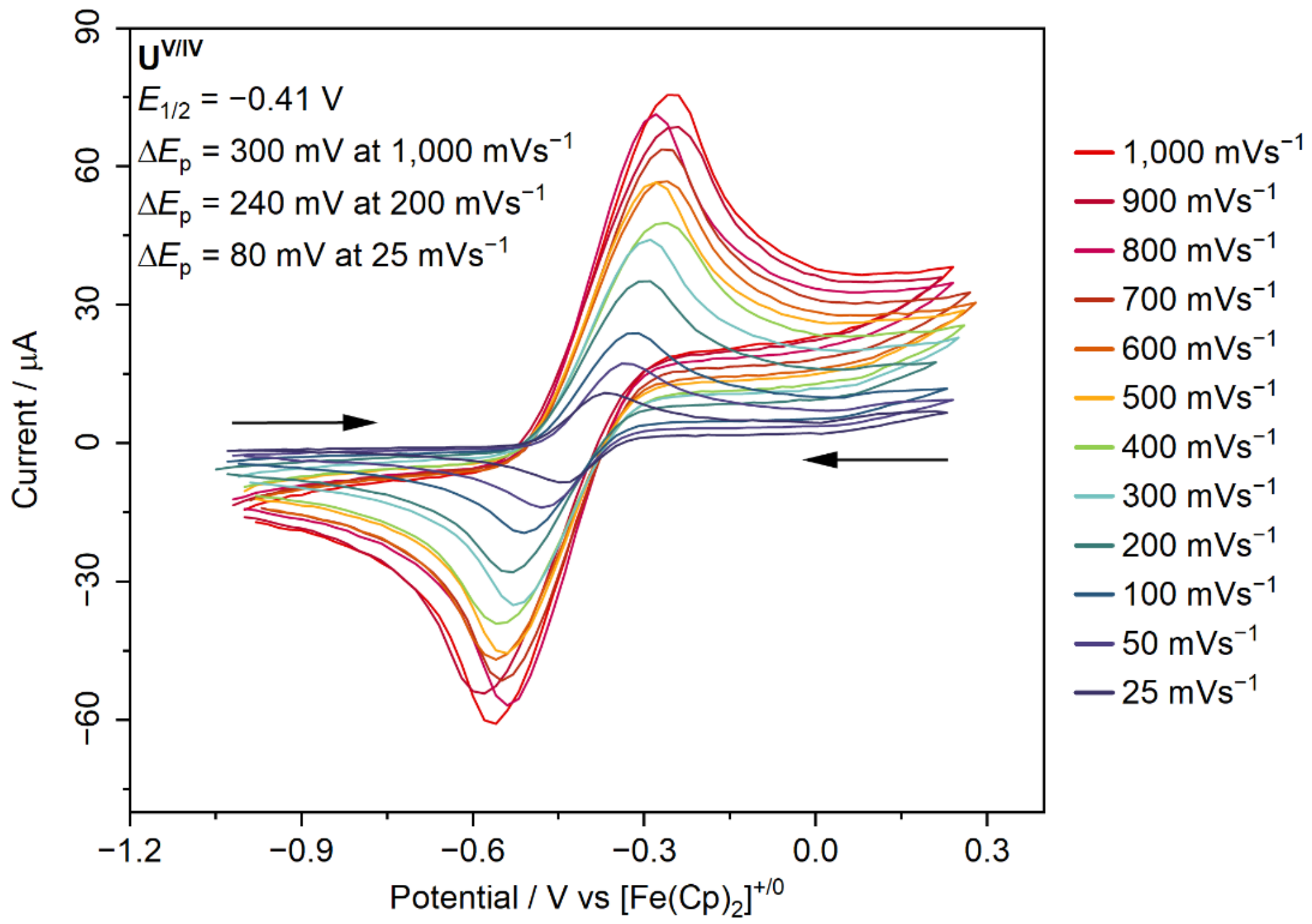

Figure S168. Cyclic voltammograms of the U(V/IV) couple of **1** (3.0 mM) in THF supported by [*n*Bu_4N][B(C_6F_5)$_4$] (0.2 M) *vs* $[Fc]^{+/0}$ showing the scan-rate dependence of the U(V/IV) couple.

Table S17. Electrochemical data for the U(V/IV) redox event *vs* $[Fc]^{+/0}$ for **1** (3.0 mM) in THF at room temperature, measured in 0.2 M [*n*Bu_4N][B(C_6F_5)$_4$].

Scan rate / mV s^{-1}	E_{pc} / V	E_{pa} / V	ΔE_p / mV	i_{pa}/i_{pc}	OCP / mV	*i*R / Ω
**25**	–0.45	–0.37	80	1.19	+211	1,716
**50**	–0.48	–0.34	140	1.06	+315	1,720
**100**	–0.51	–0.31	200	1.06	+317	2,010
**200**	–0.53	–0.29	240	1.05	+283	1,907
**300**	–0.53	–0.29	240	1.05	+206	1,647
**400**	–0.56	–0.26	300	0.97	+224	1,838
**500**	–0.54	–0.28	260	1.05	+209	1,483
**600**	–0.56	–0.26	300	1.03	+228	1,679
**700**	–0.55	–0.27	280	1.00	+218	1,528
**800**	–0.54	–0.28	260	1.02	+212	1,288
**900**	–0.58	–0.24	340	1.02	+190	1,684
**1,000**	–0.56	–0.26	300	1.02	+209	1,515

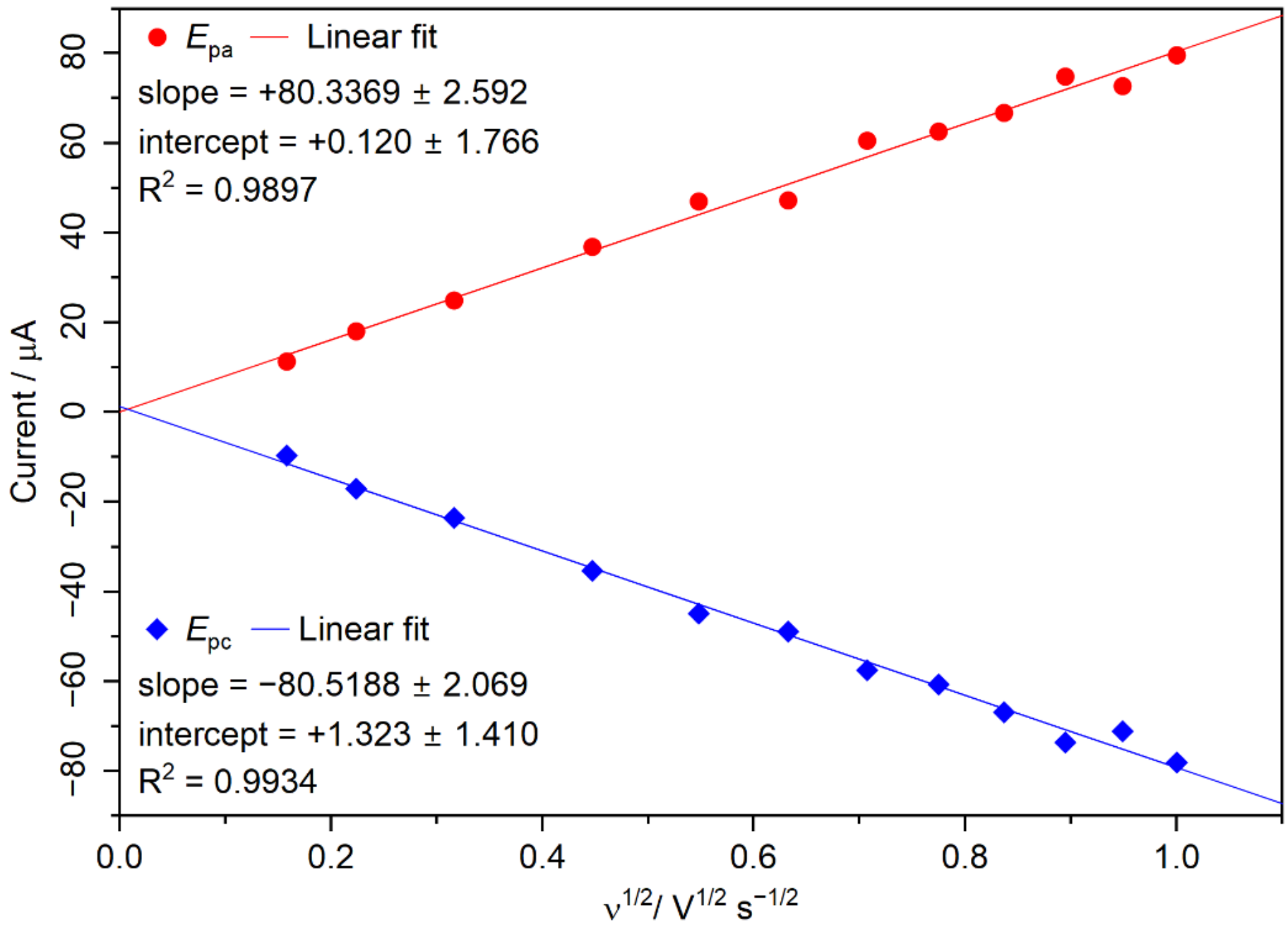

Figure S169. Randles-Sevcik plot for the U(V/IV) couple in **1**, measured in THF supported by $[nBu_4N][B(C_6F_5)_4]$ (0.2 M).

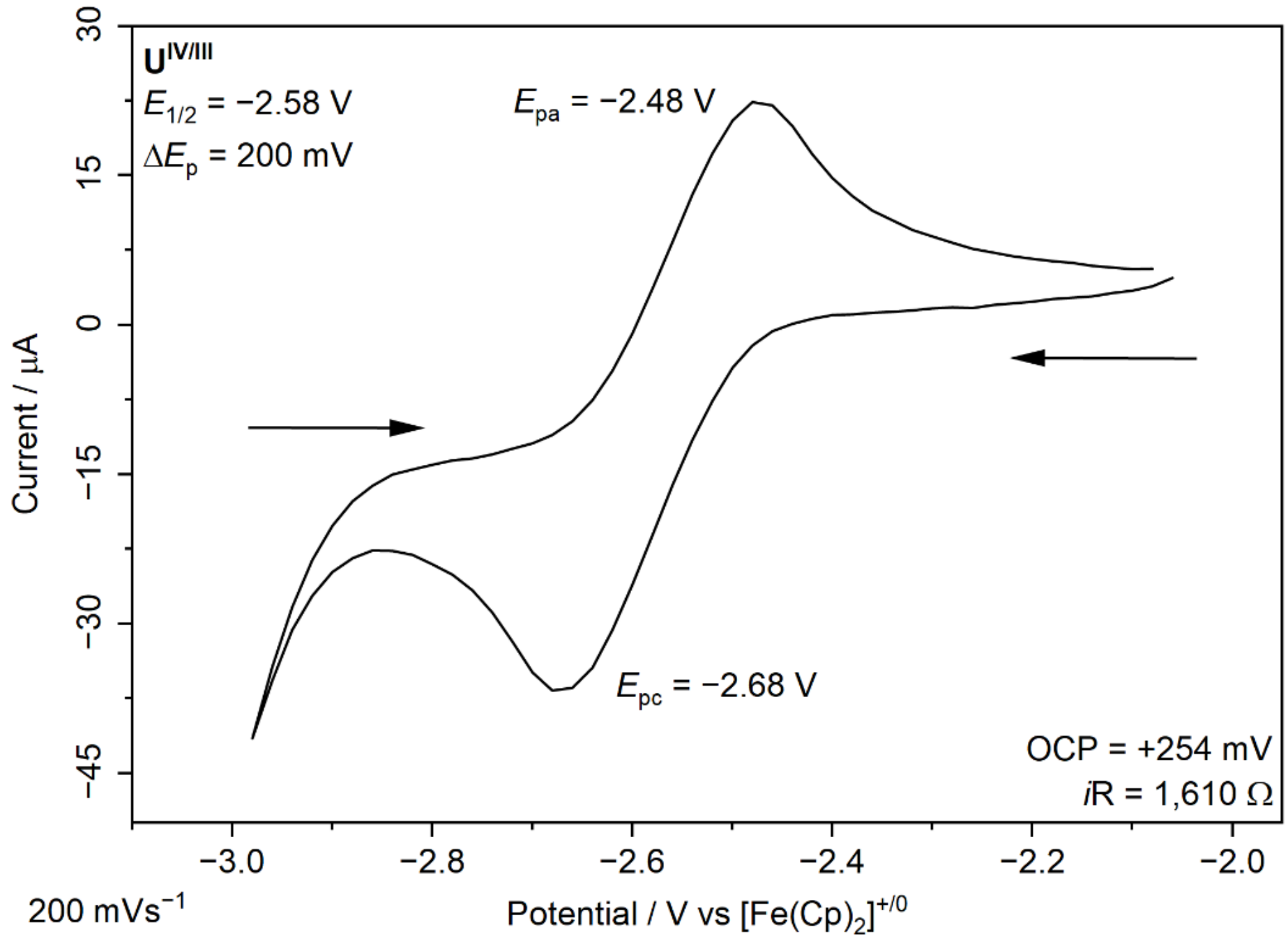

Figure S170. Cyclic voltammogram of the U(IV/III) couple of **1** (3.0 mM) in THF supported by $[nBu_4N][B(C_6F_5)_4]$ (0.2 M) *vs* $[Fc]^{+/0}$ at 200 mV s^{-1}.

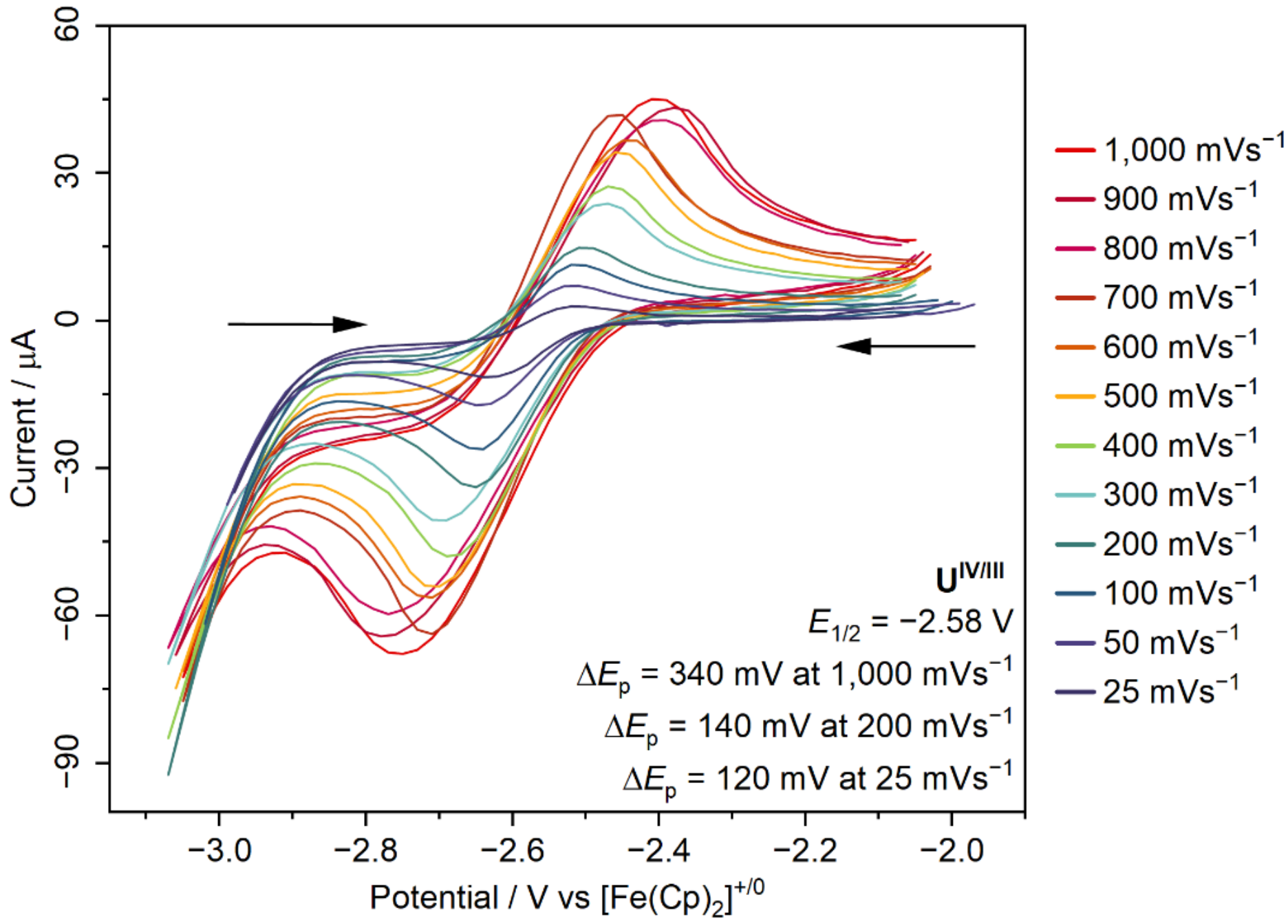

Figure S171. Cyclic voltammograms of the U(IV/III) couple of **1** (3.0 mM) in THF supported by [nBu$_4$N][B(C_6F_5)$_4$] (0.2 M) *vs* [Fc]$^{+/0}$ showing the scan-rate dependence of the U(IV/III) couple.

Table S18. Electrochemical data for the U(IV/III) redox event *vs* [Fc]$^{+/0}$ for **1** (3.0 mM) in THF at room temperature, measured in 0.2 M [nBu$_4$N][B(C_6F_5)$_4$].

Scan rate / mV s^{-1}	E_{pc} / V	E_{pa} / V	ΔE_p / mV	i_{pa}/i_{pc}	OCP / mV	iR / Ω
**25**	–2.64	–2.52	120	0.66	–257	1,578
**50**	–2.65	–2.51	140	0.76	–685	1,526
**100**	–2.64	–2.52	120	0.75	–587	1,219
**200**	–2.65	–2.51	140	0.63	–652	1,259
**300**	–2.69	–2.47	220	0.81	–664	1,672
**400**	–2.69	–2.47	220	0.78	–579	1,472
**500**	–2.70	–2.46	240	0.87	–571	1,571
**600**	–2.71	–2.45	260	0.91	–677	1,600
**700**	–2.71	–2.45	260	0.91	–658	1,400
**800**	–2.77	–2.39	380	0.96	–612	2,056
**900**	–2.78	–2.38	400	0.98	–605	2,126
**1,000**	–2.75	–2.41	340	0.95	–538	1,739

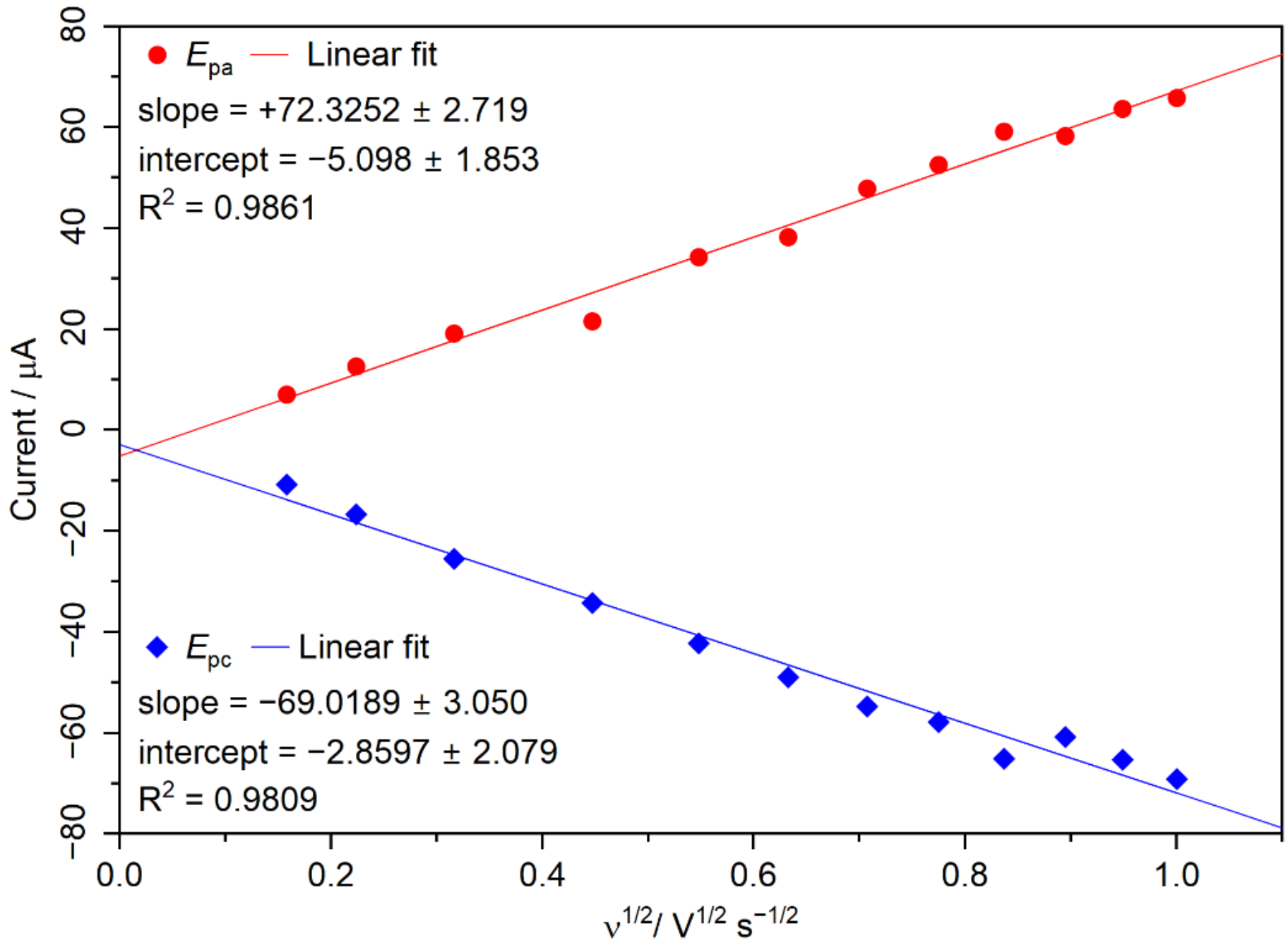

Figure S172. Randles-Sevcik plot for the U(IV/III) couple in **1**, measured in THF supported by $[nBu_4N][B(C_6F_5)_4]$ (0.2 M).

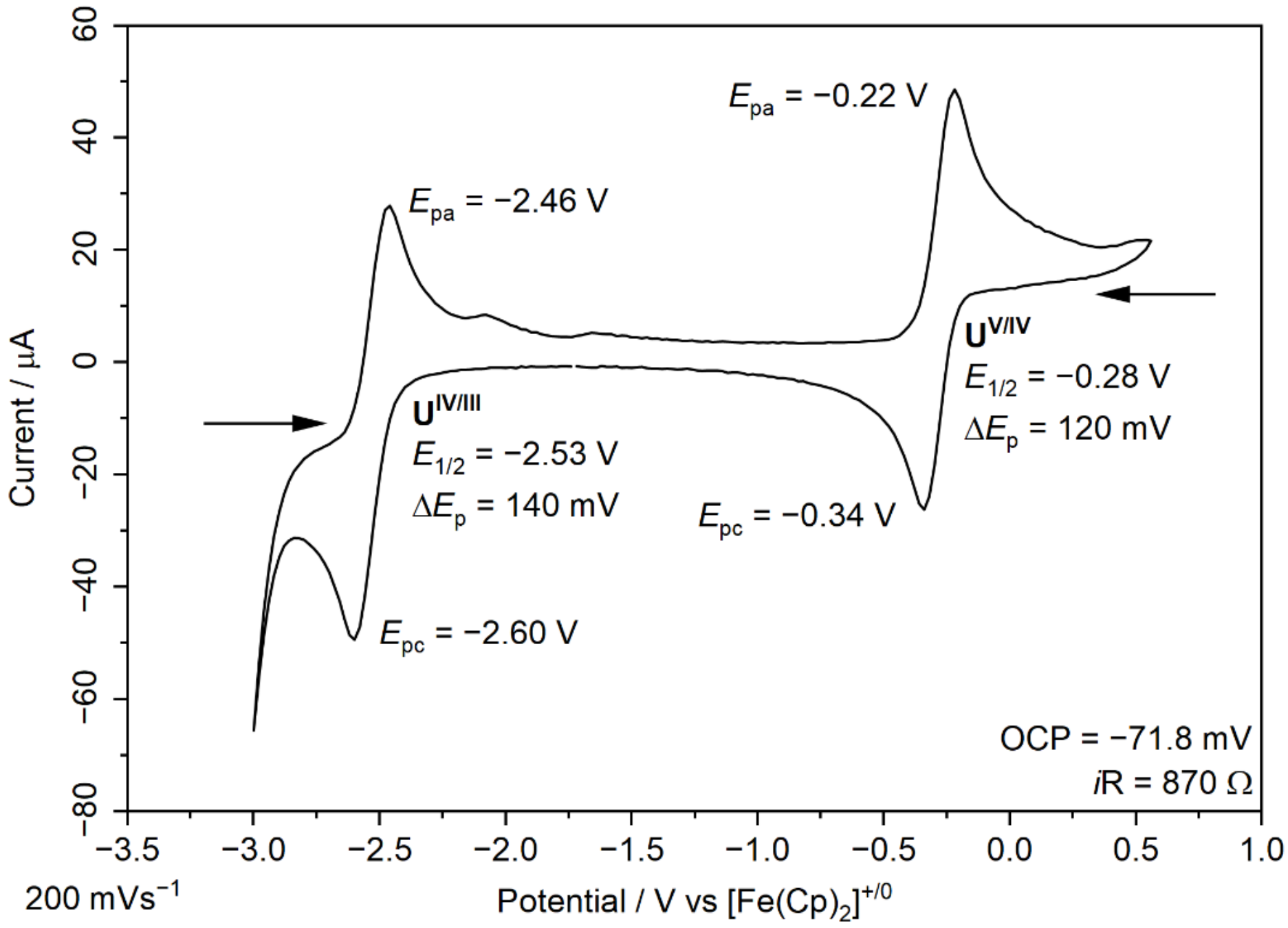

Figure S173. Cyclic voltammogram of **4** (3.0 mM) in THF supported by $[nBu_4N][B(C_6F_5)_4]$ (0.2 M) *vs* $[Fc]^{+/0}$ at 200 mV s^{-1}.

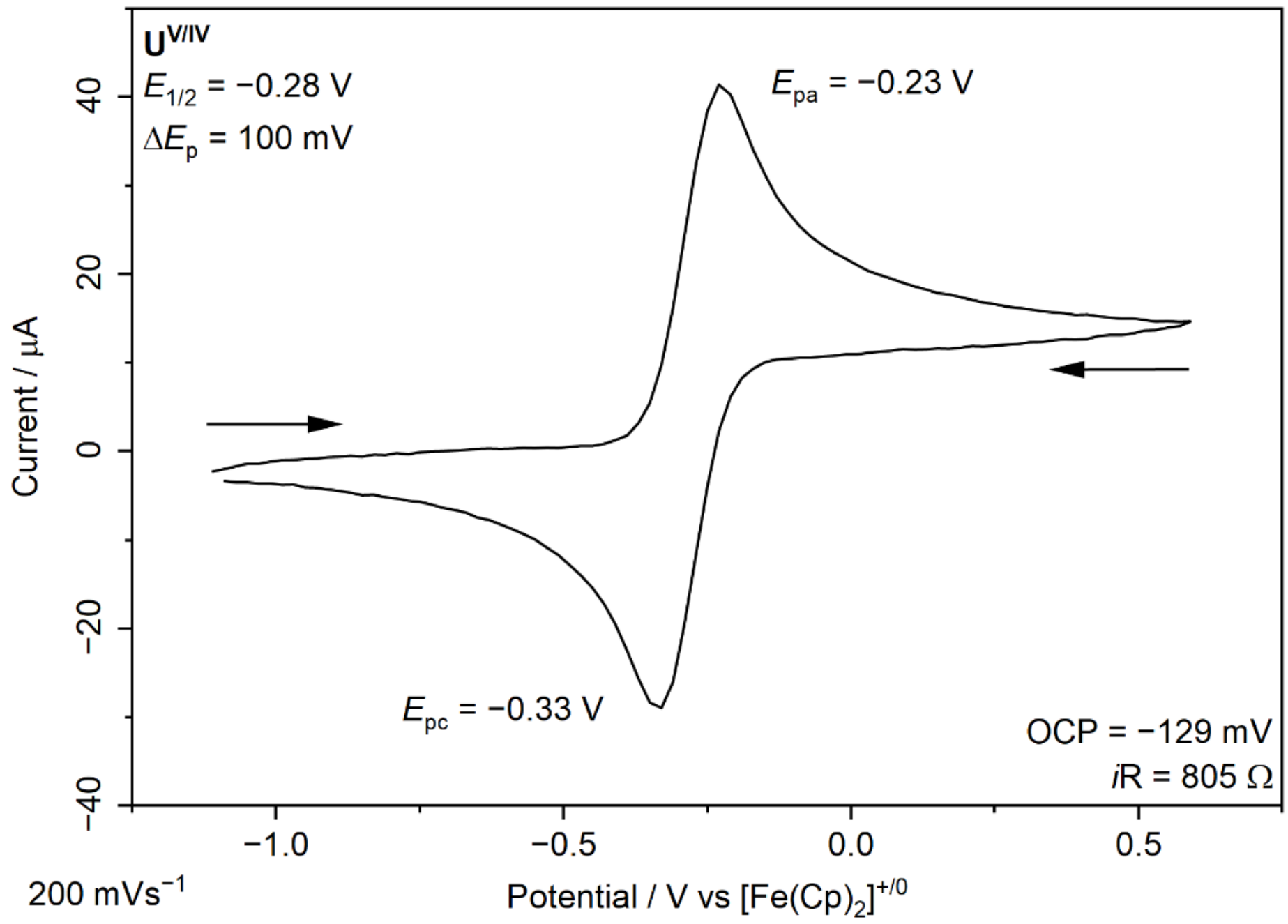

Figure S174. Cyclic voltammogram of the U(V/IV) couple of **4** (3.0 mM) in THF supported by $[nBu_4N][B(C_6F_5)_4]$ (0.2 M) *vs* $[Fc]^{+/0}$ at 200 mV s^{-1}.

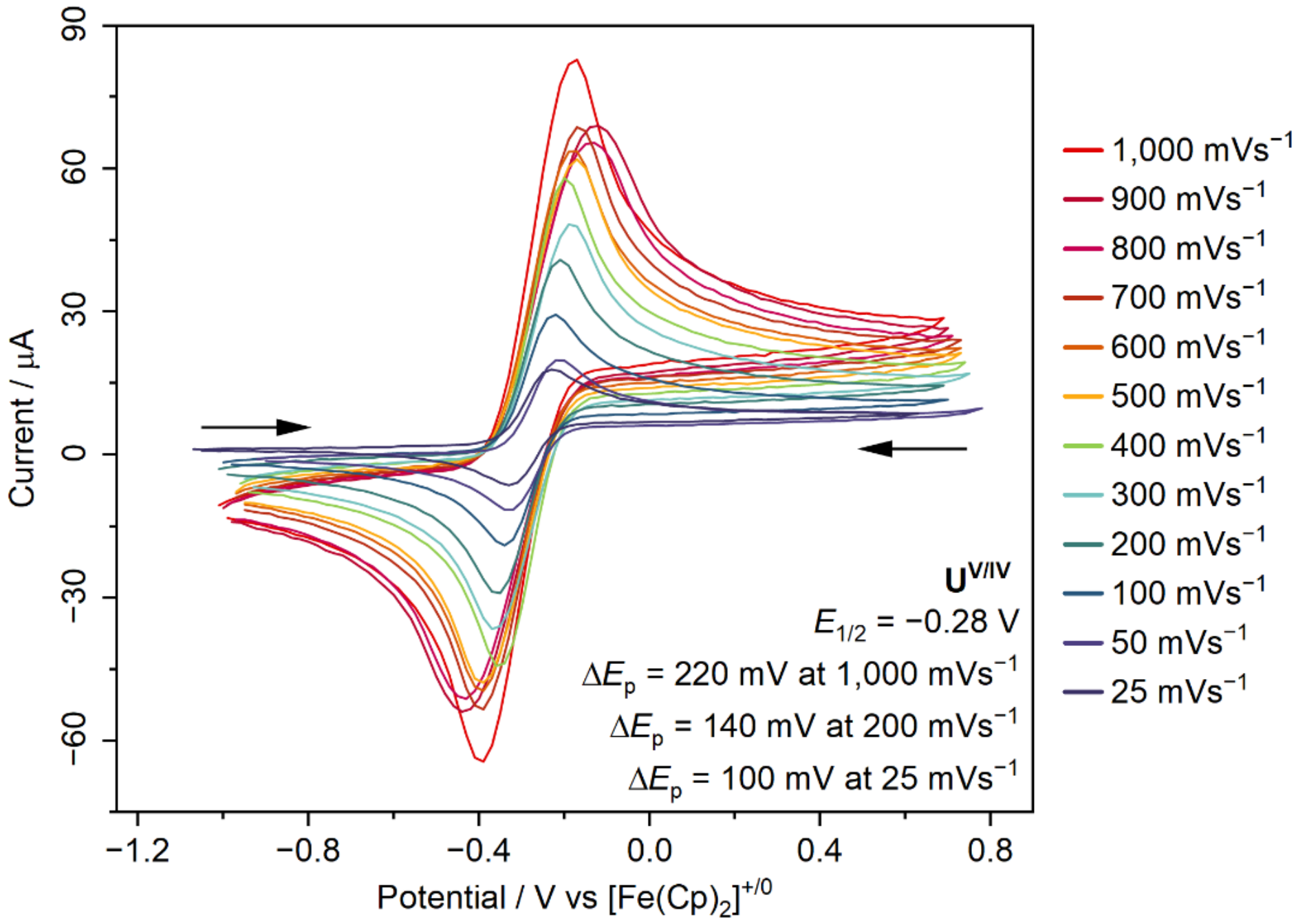

Figure S175. Cyclic voltammograms of the U(V/IV) couple of **4** (3.0 mM) in THF supported by $[nBu_4N][B(C_6F_5)_4]$ (0.2 M) *vs* $[Fc]^{+/0}$ showing the scan-rate dependence of the U(V/IV) couple.

Table S19. Electrochemical data for the U(V/IV) redox event *vs* $[Fc]^{+/0}$ for **4** (3.0 mM) in THF at room temperature, measured in 0.2 M $[nBu_4N][B(C_6F_5)_4]$.

Scan rate / mV s^{-1}	E_{pc} / V	E_{pa} / V	ΔE_p / mV	i_{pa}/i_{pc}	OCP / mV	iR / Ω
**25**	–0.33	–0.23	100	1.24	+10.0	889
**50**	–0.34	–0.22	120	1.10	+73.2	1,175
**100**	–0.34	–0.22	120	1.05	+255	1,037
**200**	–0.35	–0.21	140	1.05	+223	1,153
**300**	–0.37	–0.19	180	1.03	+243	1,294
**400**	–0.36	–0.20	160	1.02	+202	983
**500**	–0.39	–0.17	220	1.04	+197	1,291
**600**	–0.39	–0.17	220	1.03	+256	1,212
**700**	–0.39	–0.17	220	0.97	+267	1,250
**800**	–0.43	–0.13	300	1.03	+187	1,561
**900**	–0.44	–0.12	320	1.03	+210	1,561
**1,000**	–0.39	–0.17	220	1.04	+206	1,045

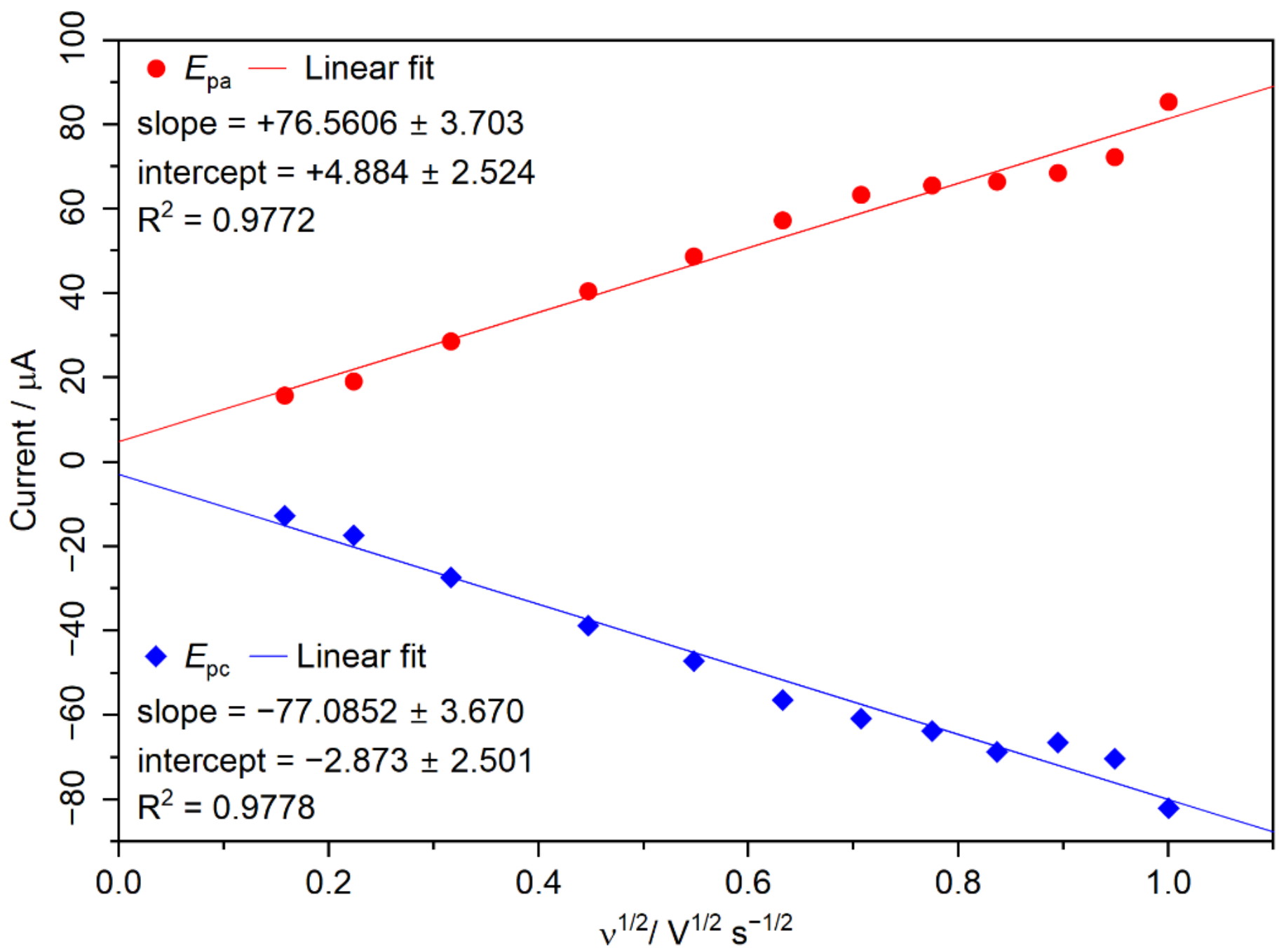

Figure S176. Randles-Sevcik plot for the U(V/IV) couple in **4**, measured in THF supported by $[nBu_4N][B(C_6F_5)_4]$ (0.2 M).

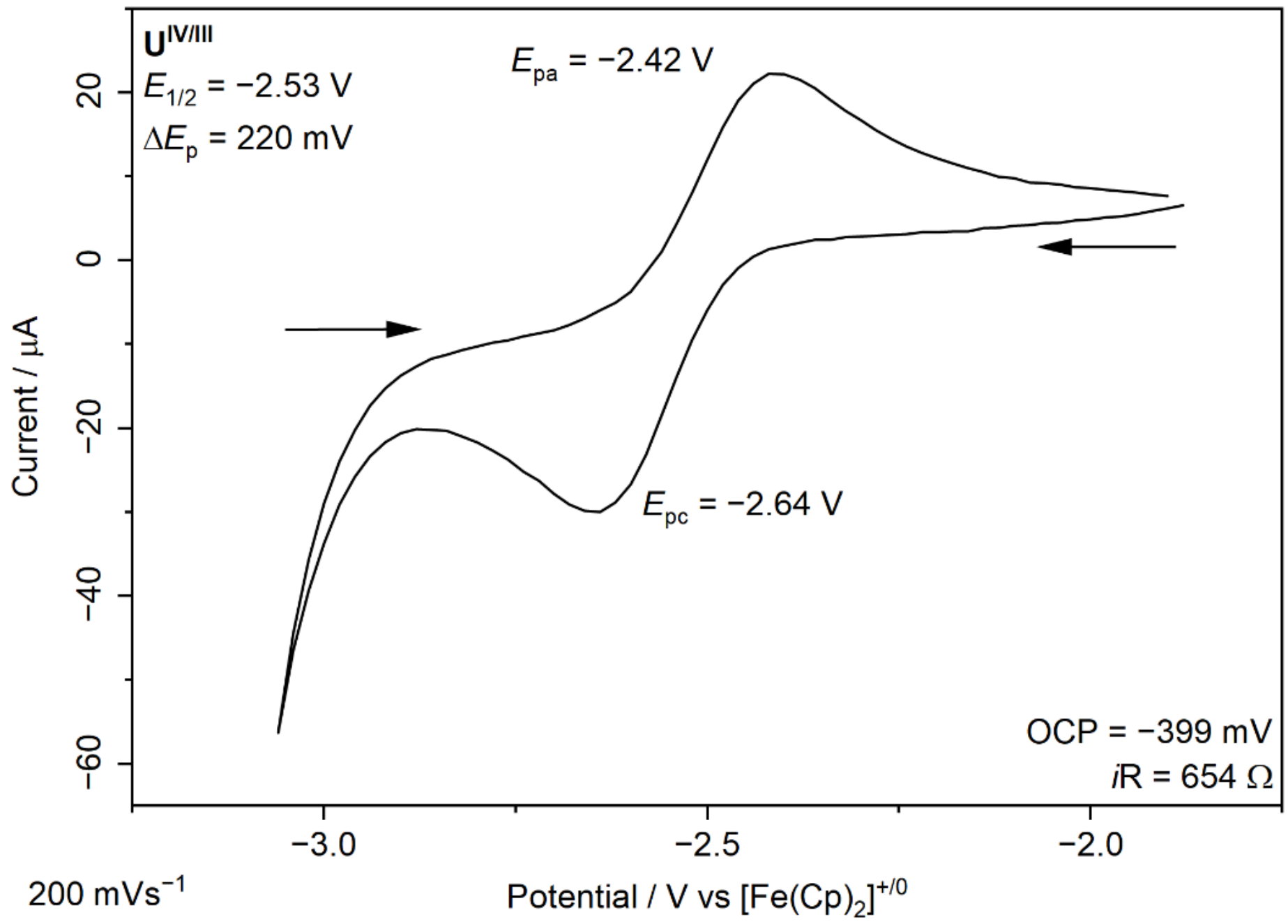

Figure S177. Cyclic voltammogram of the U(IV/III) couple of **4** (3.0 mM) in THF supported by [nBu$_4$N][B(C$_6$F$_5$)$_4$] (0.2 M) *vs* [Fc]$^{+/0}$ at 200 mV s^{-1}.

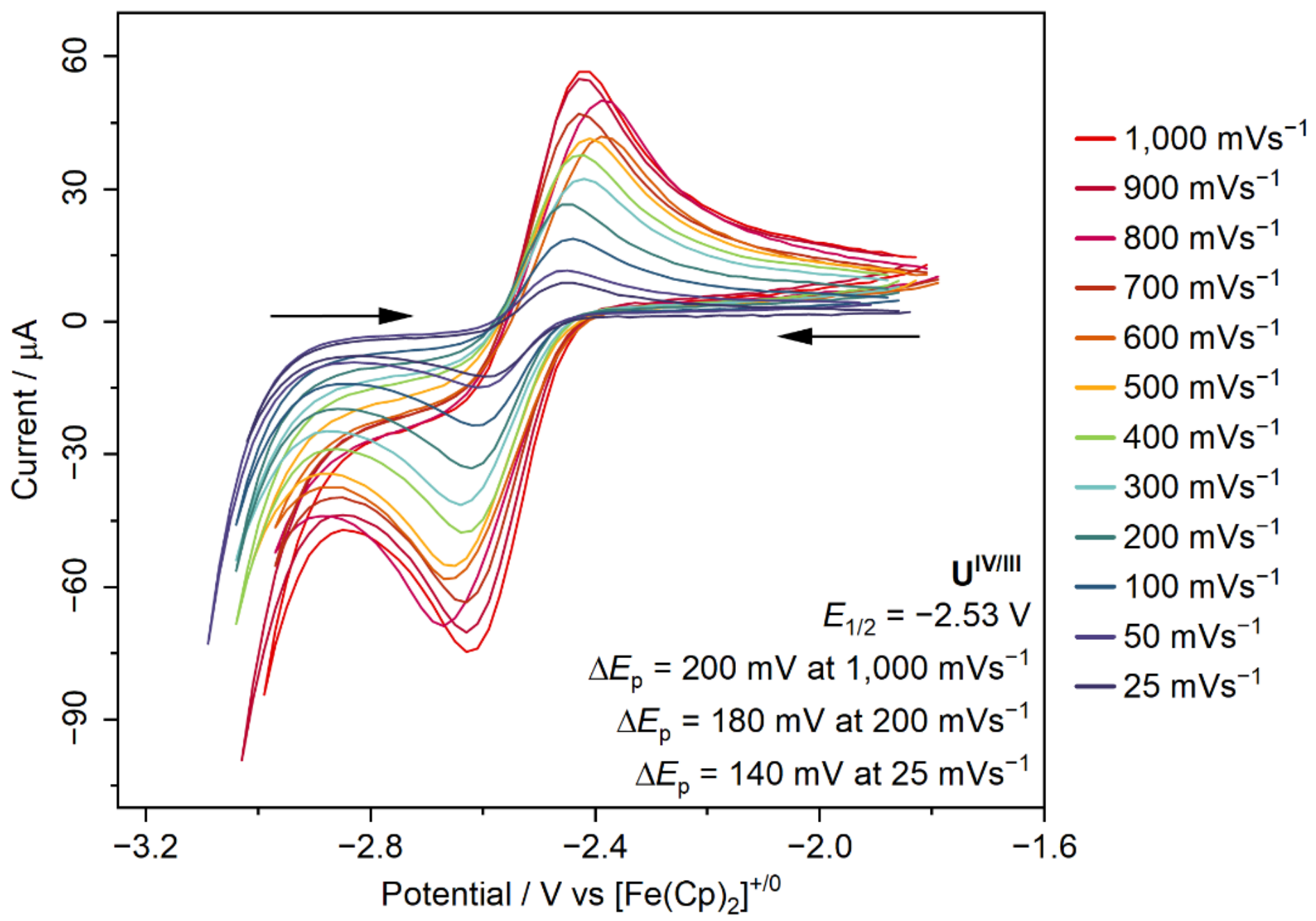

Figure S178. Cyclic voltammograms of the U(IV/III) couple of **4** (3.0 mM) in THF supported by [nBu$_4$N][B(C$_6$F$_5$)$_4$] (0.2 M) *vs* [Fc]$^{+/0}$ showing the scan-rate dependence of the U(IV/III) couple.

Table S20. Electrochemical data for the U(IV/III) redox event *vs* $[Fc]^{+/0}$ for **4** (3.0 mM) in THF at room temperature, measured in 0.2 M $[nBu_4N][B(C_6F_5)_4]$.

Scan rate / mV s^{-1}	E_{pc} / V	E_{pa} / V	ΔE_p / mV	i_{pa}/i_{pc}	OCP / mV	iR / Ω
**25**	–2.60	–2.46	140	0.91	–354	678
**50**	–2.61	–2.45	160	0.89	–422	637
**100**	–2.62	–2.44	180	0.98	–470	822
**200**	–2.62	–2.44	180	1.00	–465	704
**300**	–2.64	–2.42	220	0.99	–469	1,034
**400**	–2.64	–2.42	220	0.96	–471	808
**500**	–2.65	–2.41	240	0.96	–477	1,026
**600**	–2.67	–2.39	280	0.96	–461	1,243
**700**	–2.63	–2.43	200	0.96	–441	811
**800**	–2.67	–2.39	280	0.99	–422	1,251
**900**	–2.63	–2.43	200	0.98	–474	786
**1,000**	–2.63	–2.43	200	0.96	–467	743

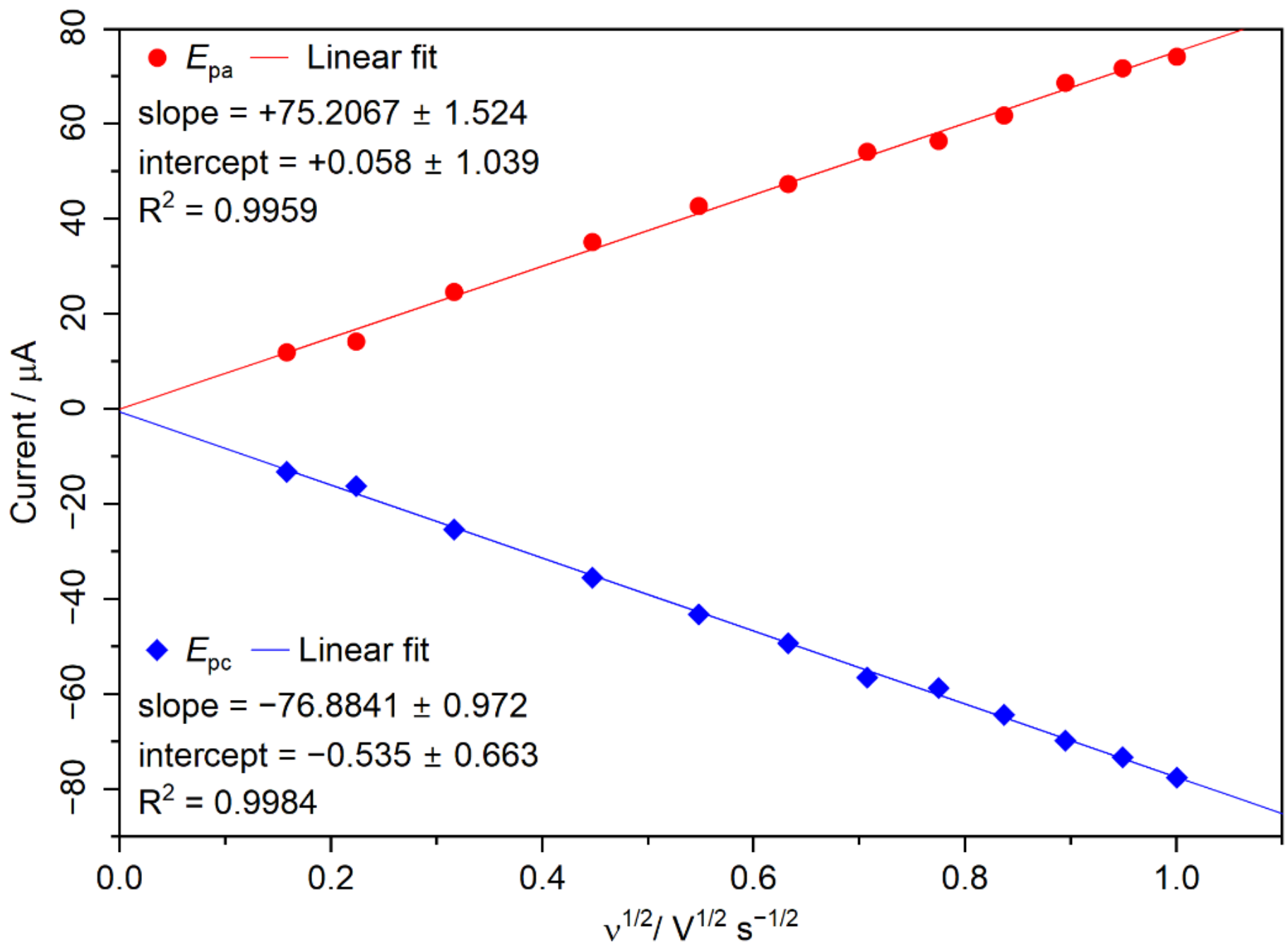

Figure S179. Randles-Sevcik plot for the U(IV/III) couple in **4**, measured in THF supported by $[nBu_4N][B(C_6F_5)_4]$ (0.2 M).

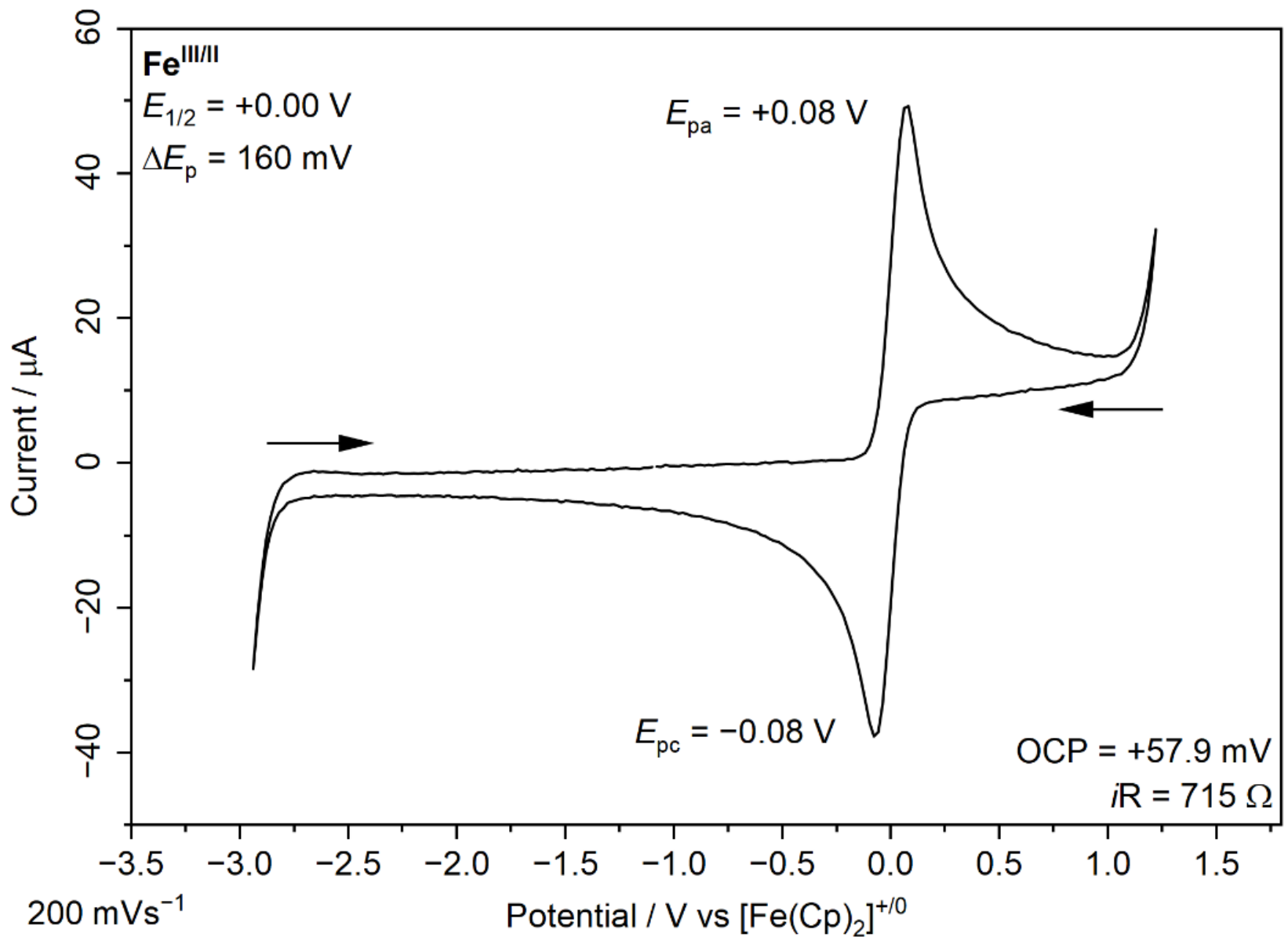

Figure S180. Top: Cyclic voltammogram of Fc (3.0 mM) in THF supported by $[nBu_4N][B(C_6F_5)_4]$ (0.2 M) *vs* $[Fc]^{+/0}$ at 200 mV s^{-1}.

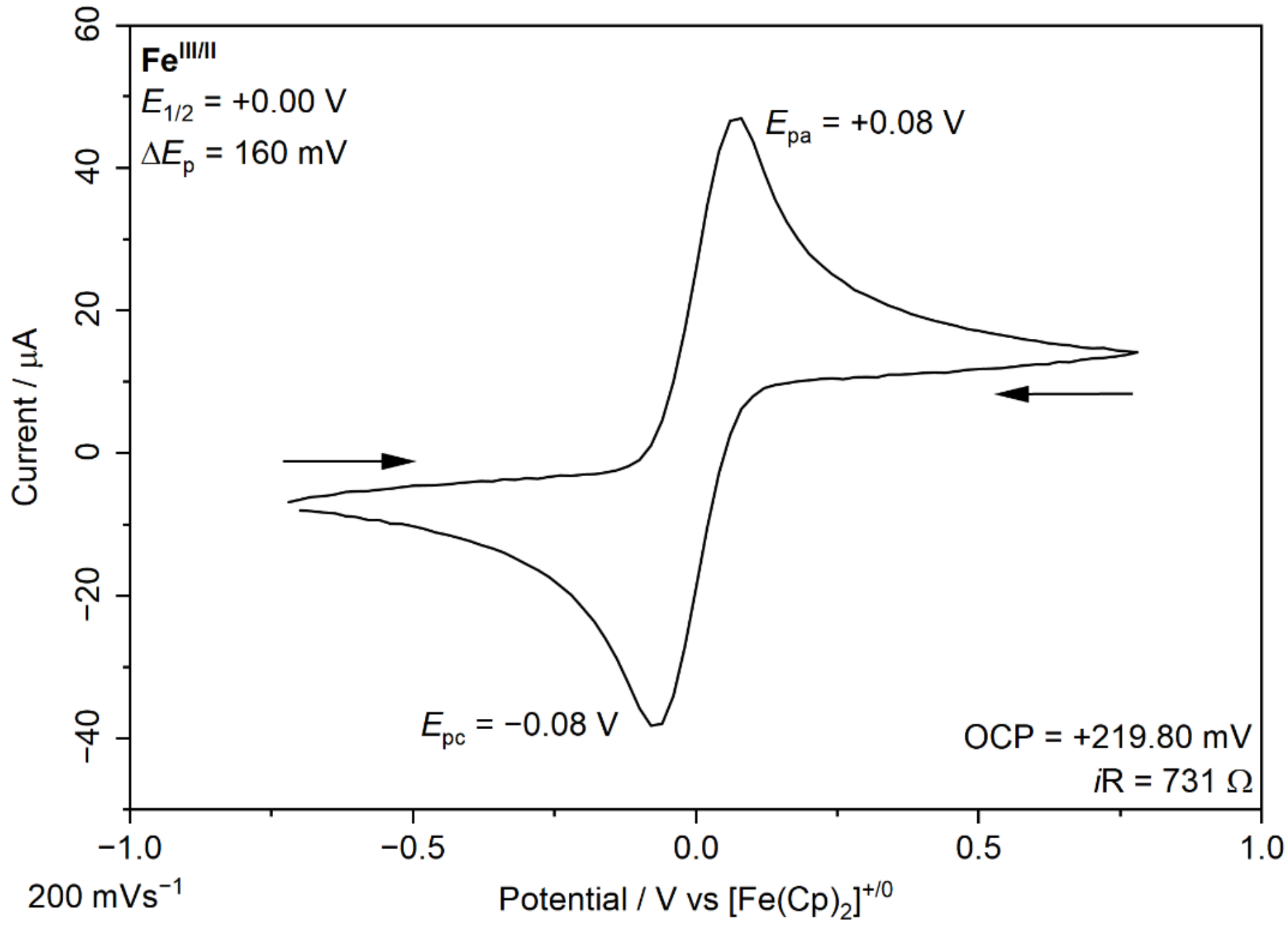

Figure S181. Cyclic voltammogram of the Fe(III/II) couple of Fc (3.0 mM) in THF supported by $[nBu_4N][B(C_6F_5)_4]$ (0.2 M) *vs* $[Fc]^{+/0}$ at 200 mV s^{-1}.

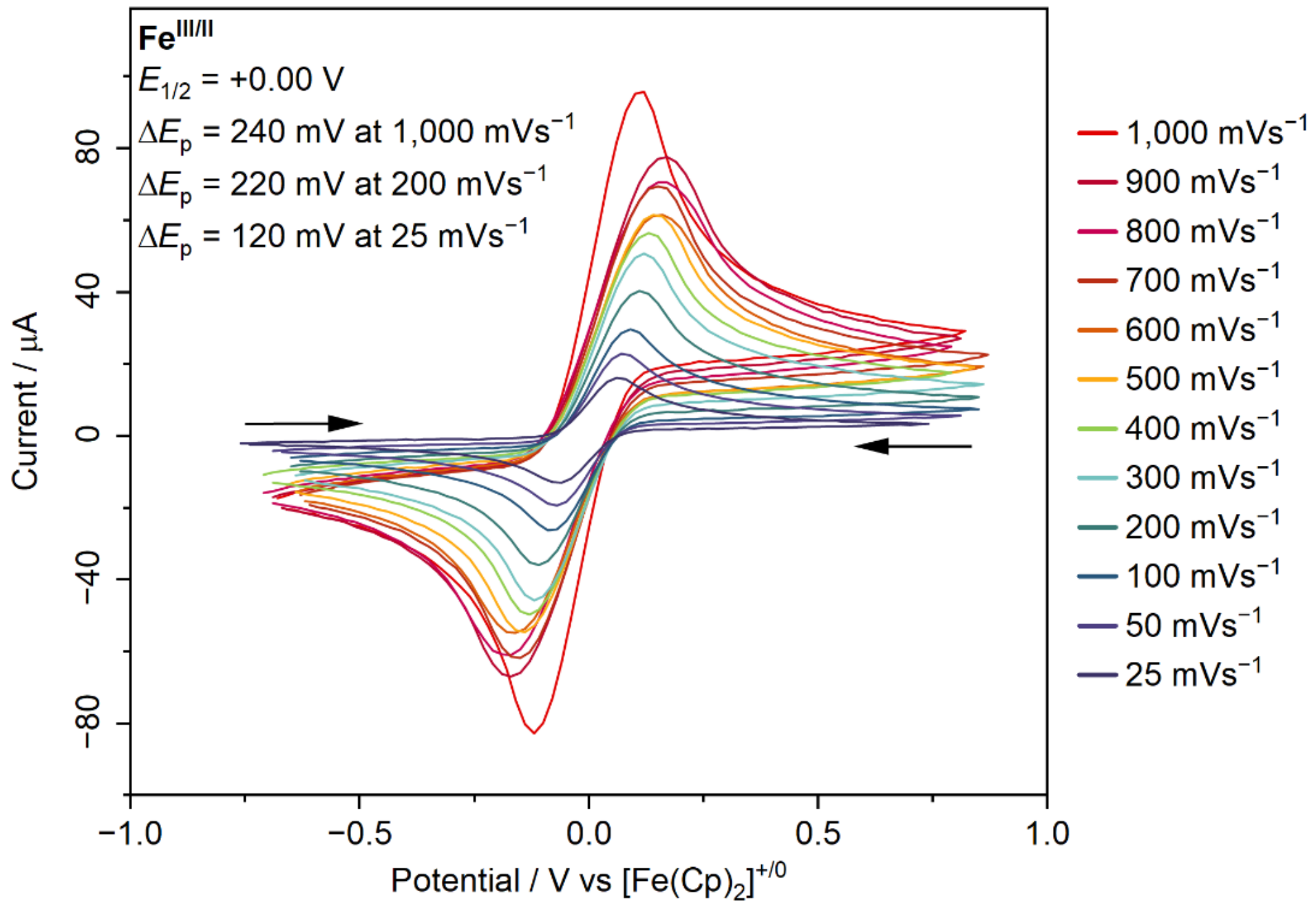

Figure S182. Cyclic voltammograms of the Fe(III/II) couple of Fc (3.0 mM) in THF supported by [nBu$_4$N][B(C$_6$F$_5$)$_4$] (0.2 M) *vs* [Fc]$^{+/0}$ showing the scan-rate dependence of the Fe(III/II) couple.

Table S21. Electrochemical data for the Fe(III/II) redox event *vs* [Fc]$^{+/0}$ for Fc (3.0 mM) in THF at room temperature, measured in 0.2 M [nBu$_4$N][B(C$_6$F$_5$)$_4$].

Scan rate / mV s^{-1}	E_{pc} / V	E_{pa} / V	ΔE_p / mV	i_{pa}/i_{pc}	OCP / mV	iR / Ω
**25**	–0.06	0.06	120	1.15	+273	1,456
**50**	–0.07	0.07	140	1.11	+320	1,319
**100**	–0.09	0.09	180	1.08	+344	1,361
**200**	–0.11	0.11	220	1.05	+346	1,451
**300**	–0.12	0.12	240	1.04	+343	1,322
**400**	–0.13	0.13	260	1.01	+267	1,355
**500**	–0.14	0.14	280	1.03	+314	1,412
**600**	–0.16	0.16	320	1.03	+336	1,521
**700**	–0.15	0.15	300	0.99	+343	1,350
**800**	–0.17	0.17	340	1.03	+277	1,474
**900**	–0.17	0.17	340	1.01	+287	1,368
**1,000**	–0.12	0.12	240	1.01	+282	827

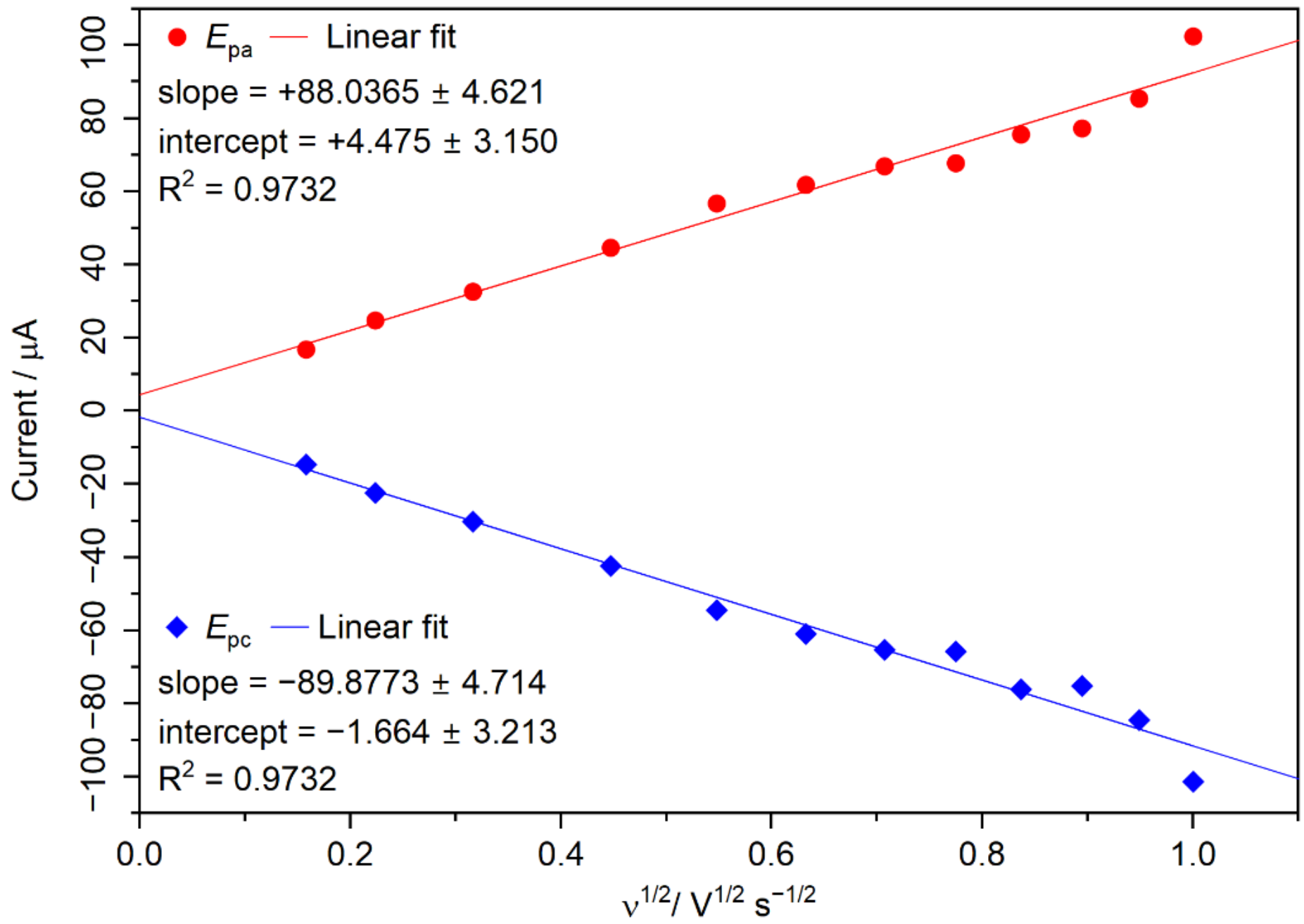

Figure S183. Randles-Sevcik plot for the Fe(III/II) couple in Fc, measured in THF supported by [nBu$_4$N][B(C_6F_5)$_4$] (0.2 M).

*Data for **1** and **4** in THF supported by [nBu$_4$N][PF$_6$]*

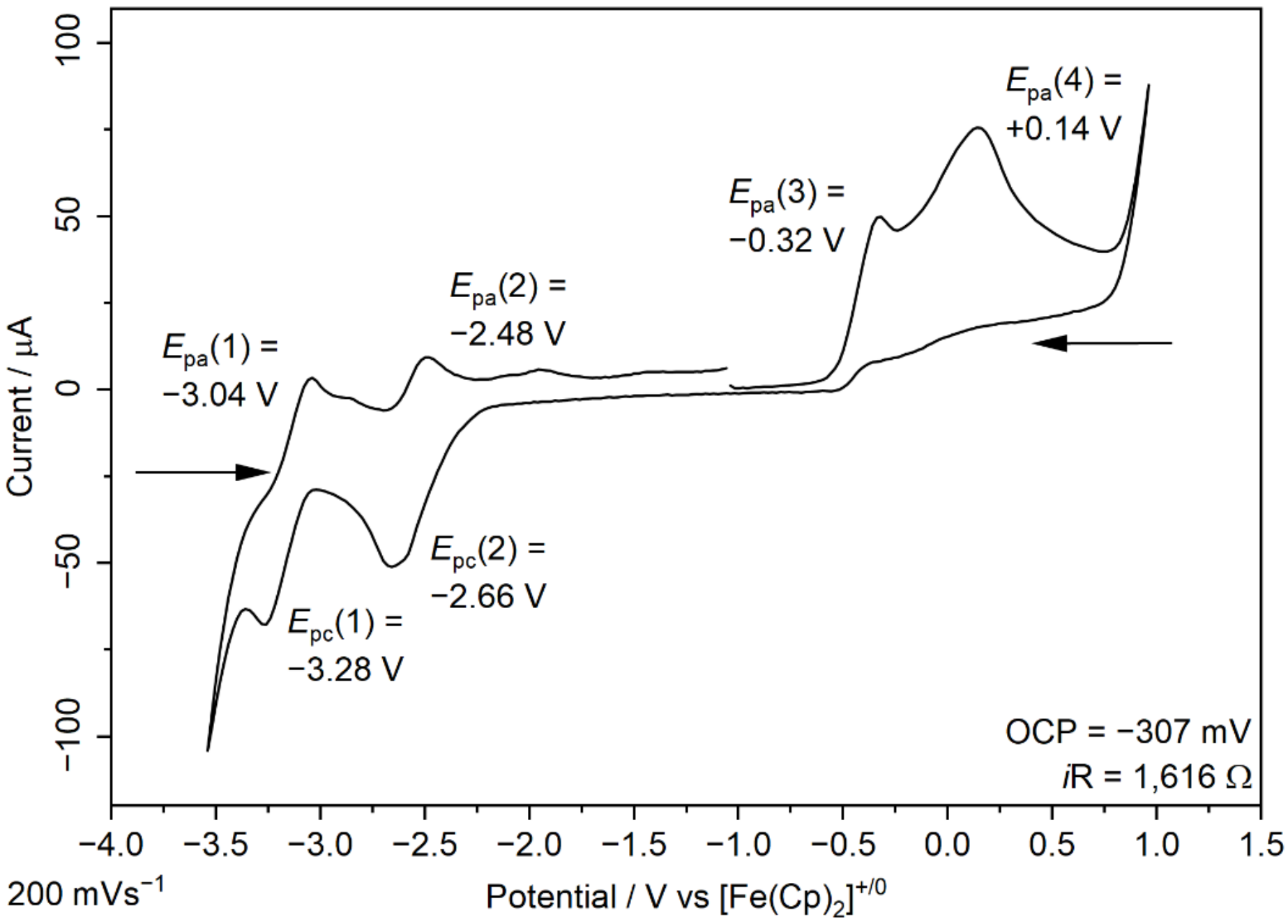

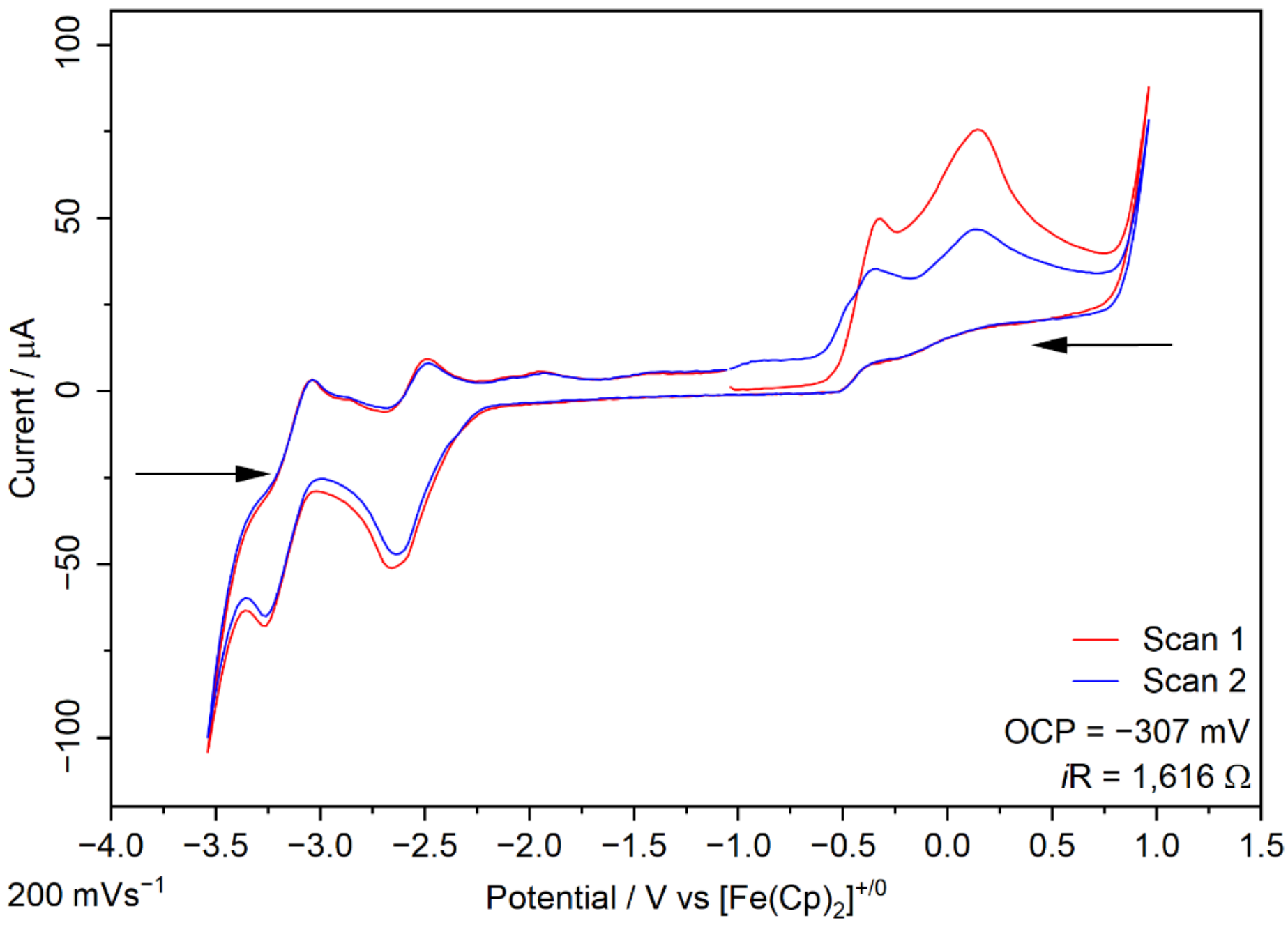

Figure S184. Top: Cyclic voltammogram of **1** (3.0 mM) in THF supported by [*n*Bu$_4$N][PF$_6$] (0.2 M) *vs* [Fc]$^{+/0}$ at 200 mV s^{-1}, starting at *ca.* –1.00 V and scanning in the positive direction first. **Bottom:** Repeat scans of **1**, scan 1 (red) followed by scan 2 (blue) showing a decrease in current intensity for oxidative peaks E_{pa}(3) and E_{pa}(4). The pair E_{pc}(1) and E_{pa}(1) likely arise from a product derived the reaction between [U^{III}(COT)$_2$]$^-$, generated at E_{pc}(2), and the and the PF$_6$- electrolyte. Comparison with the scans where [*n*Bu$_4$N][B(C$_6$F$_5$)$_4$] was used as the electrolyte show the generation of [U^{III}(COT)$_2$]$^-$ at a nearly-identical E_{pc} value (–2.69 V), without the additional cathodically shifted features shown here.

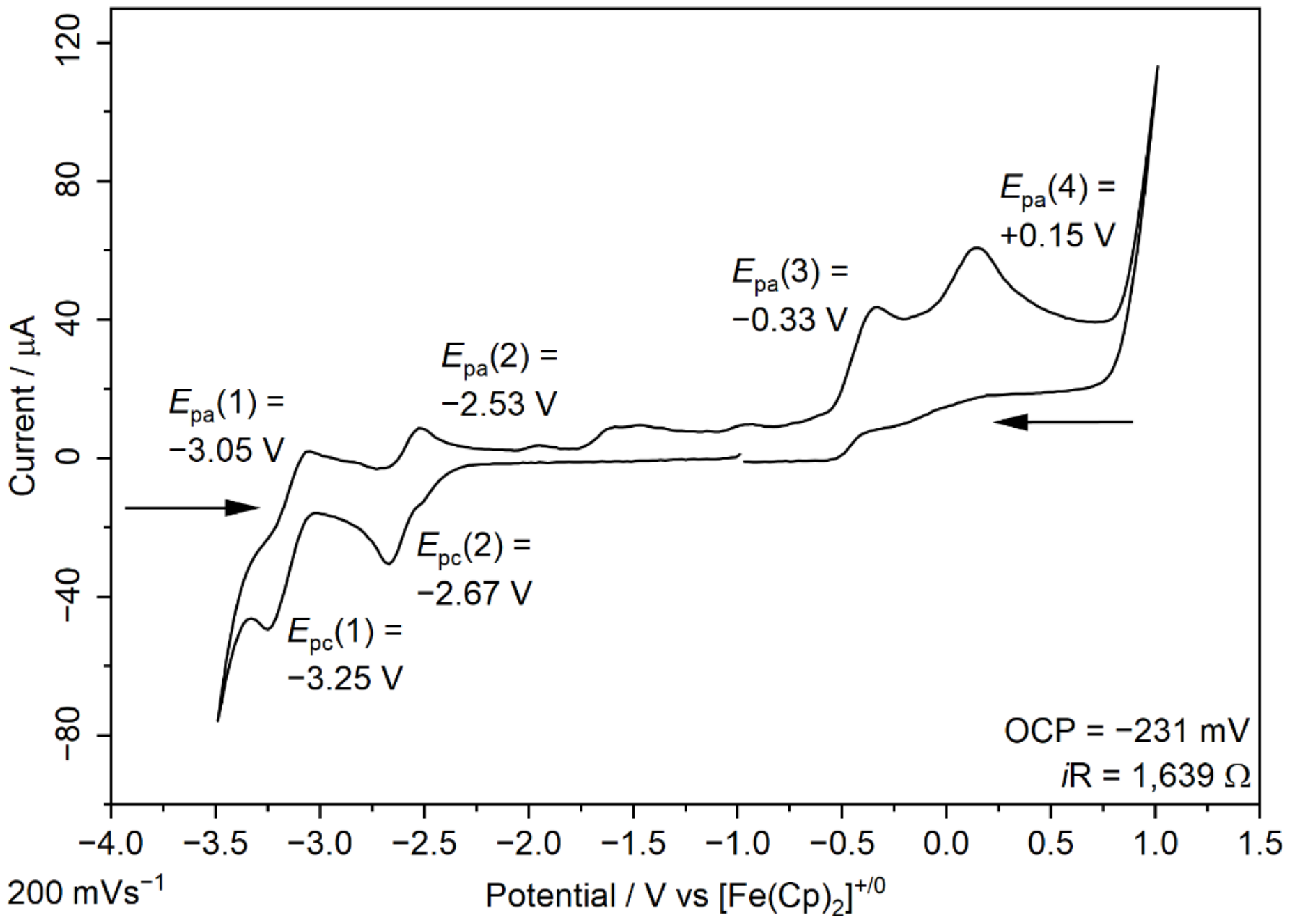

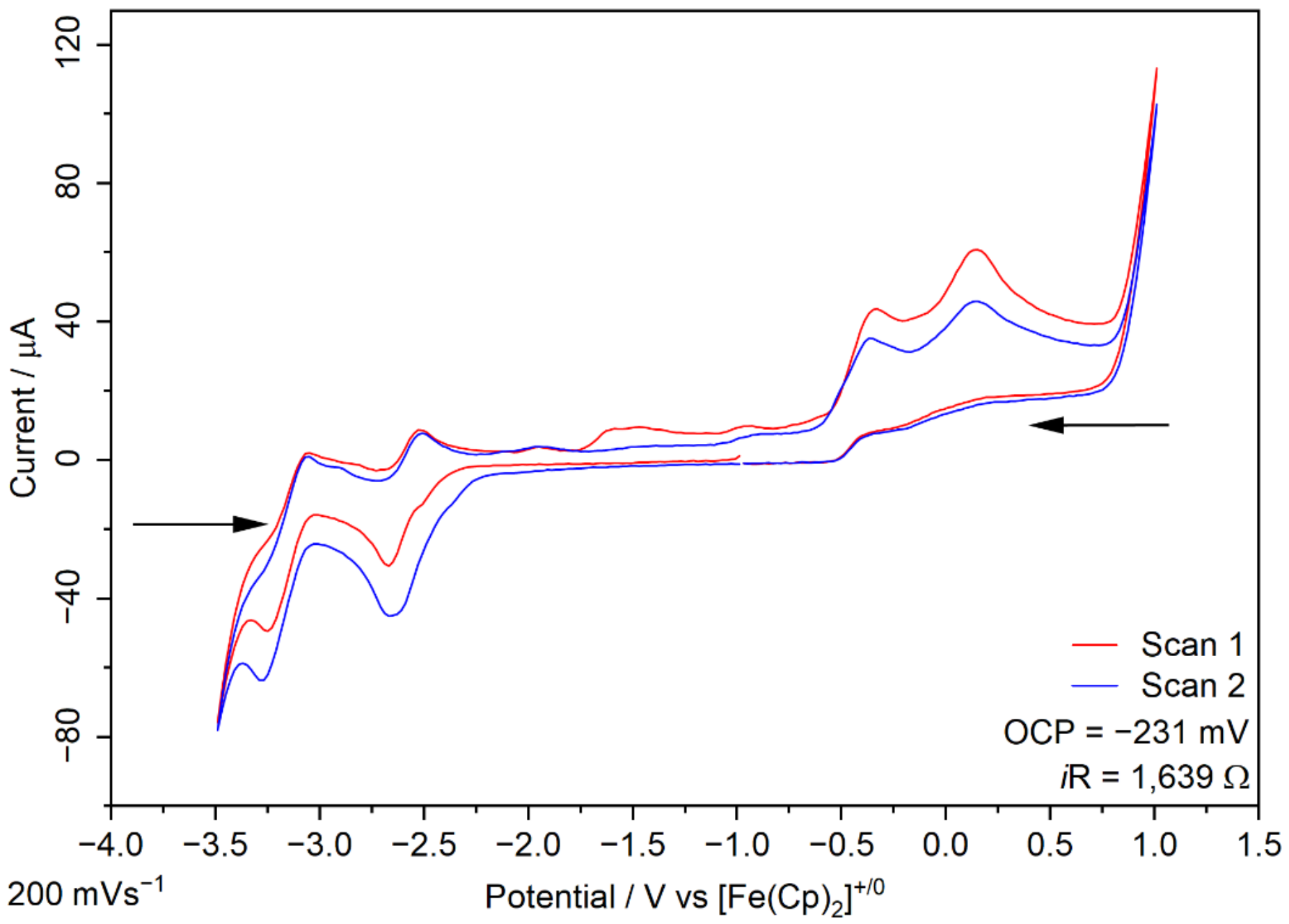

Figure S185. Top: Cyclic voltammogram of **1** (3.0 mM) in THF supported by [nBu$_4$N][PF$_6$] (0.2 M) *vs* [Fc]$^{+/0}$ at 200 mV s^{-1}, starting at *ca.* –1.00 V and scanning in the negative direction first. **Bottom:** Repeat scans of **1**, scan 1 (red) and scan 2 (blue) showing a slight decrease in current intensity for oxidative peaks $E_{pa}(3)$ and $E_{pa}(4)$, and a slight increase in current intensity for reductive peaks $E_{pc}(1)$ and $E_{pc}(2)$.

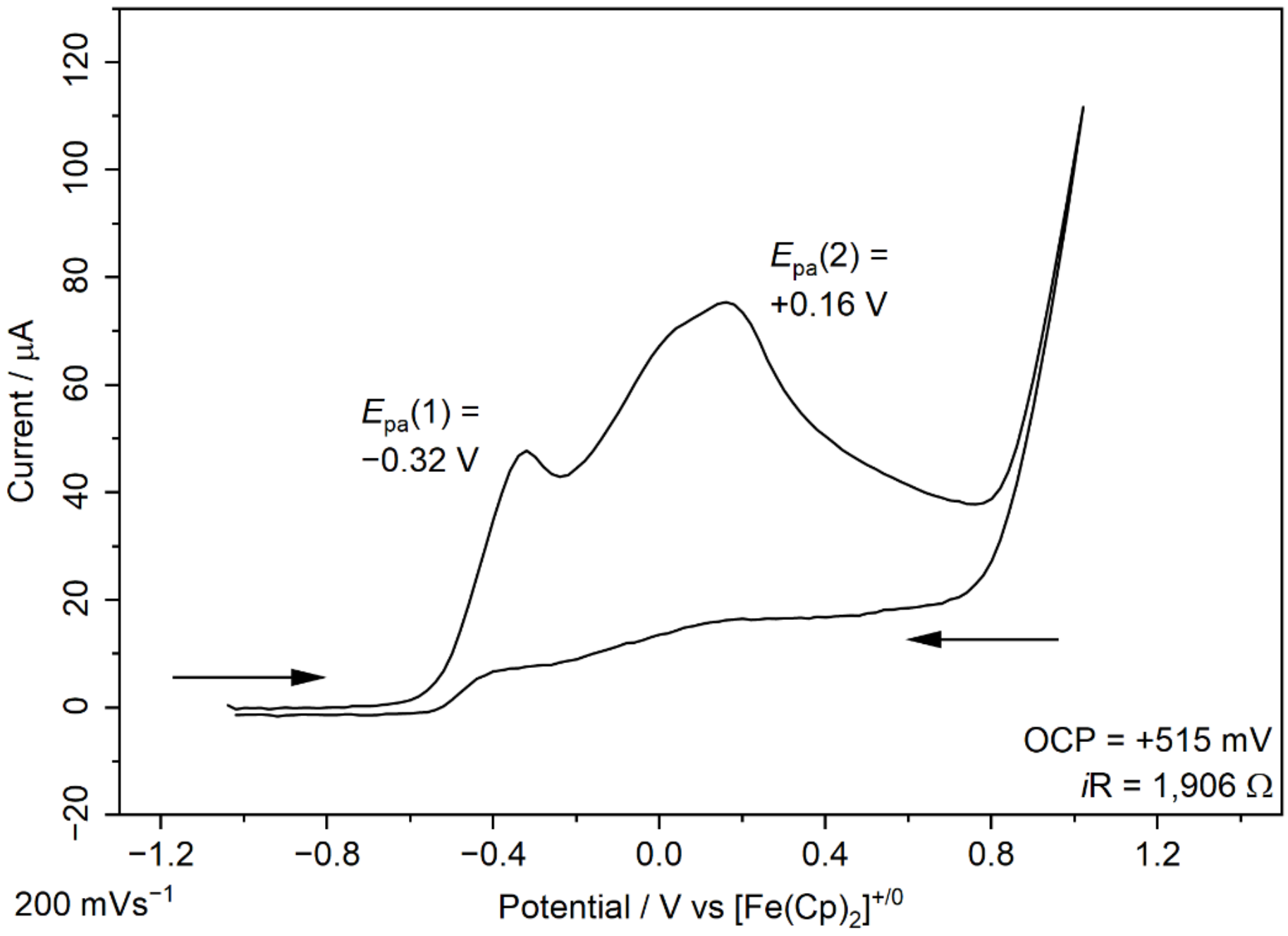

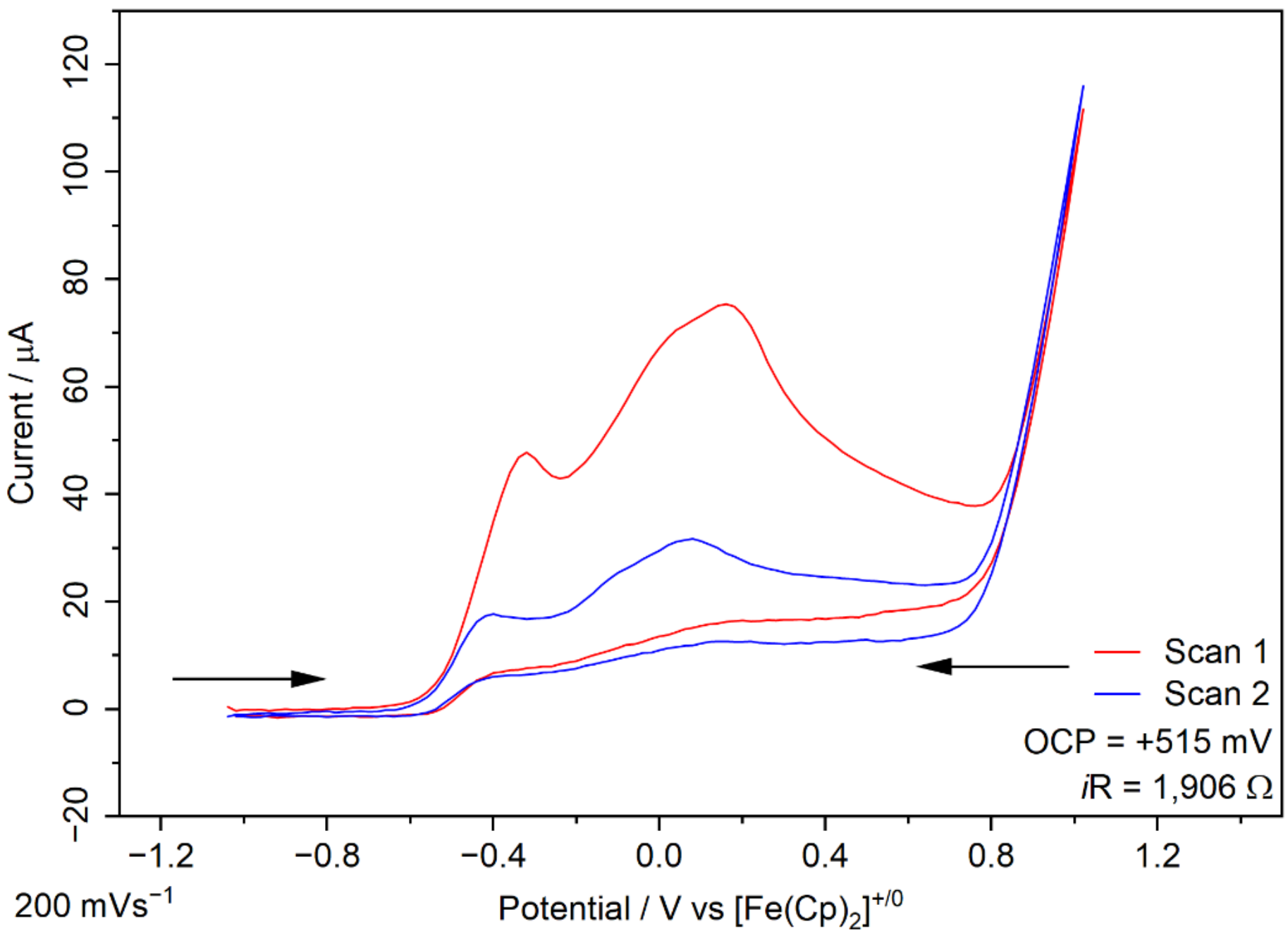

Figure S186. Top: Cyclic voltammogram of **1** (3.0 mM) in THF supported by [nBu$_4$N][PF$_6$] (0.2 M) *vs* [Fc]$^{+/0}$ at 200 mV s^{-1}, starting at *ca.* –1.00 V and scanning in the positive direction, only scanning between *ca*. –1.00 V and +1.00 V. **Bottom:** Repeat scans for the positive region of **1**, scan 1 (red) followed by scan 2 (blue) showing a significant decrease in current intensity for oxidative peaks E_{pa}(1) and E_{pa}(2).

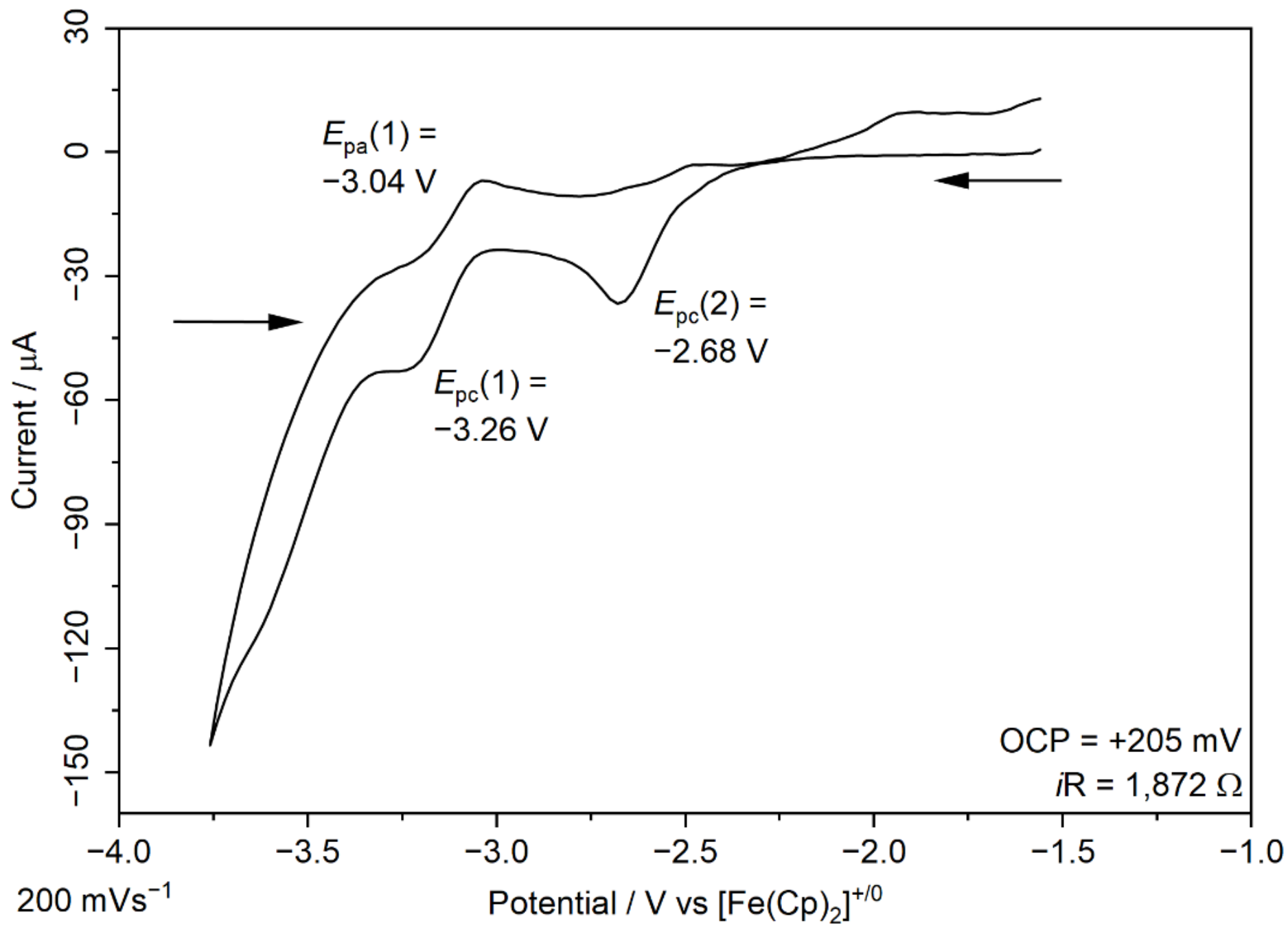

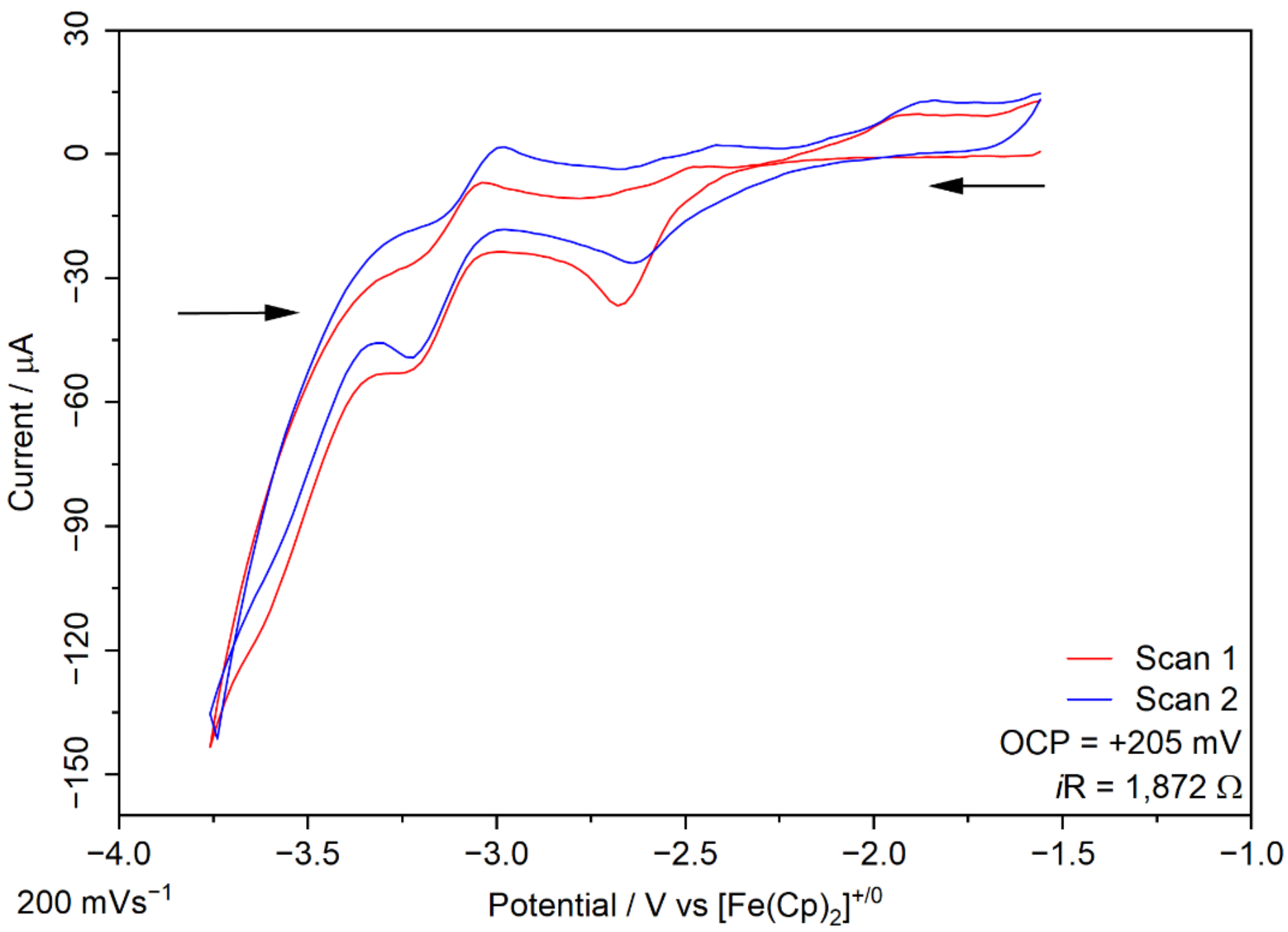

Figure S187. Top: Cyclic voltammogram of **1** (3.0 mM) in THF supported by [*n*Bu$_4$N][PF$_6$] (0.2 M) *vs* [Fc]$^{+/0}$ at 200 mV s^{-1}, starting at *ca.* –1.50 V and scanning in the negative direction, only scanning between *ca*. –1.50 V and –3.75 V. **Bottom:** Repeat scans of **1**, scan 1 (red) and scan 2 (blue) showing clarity in E_{pc}(1) and a decrease in current intensity of E_{pc}(2).

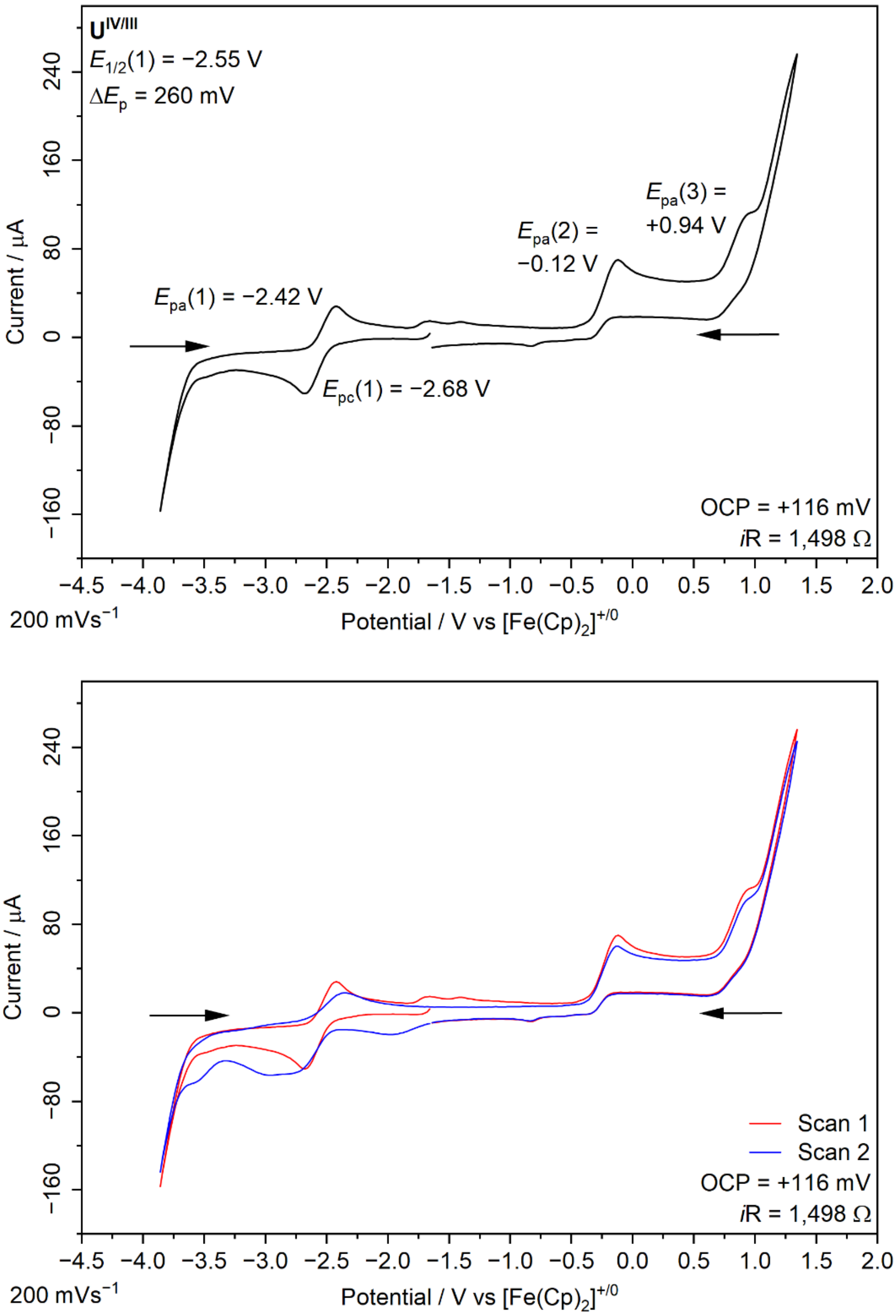

Figure S188. Top: Cyclic voltammogram of **4** (3.0 mM) in THF supported by $[nBu_4N][PF_6]$ (0.2 M) *vs* $[Fc]^{+/0}$ at 200 mV s^{-1}, starting at *ca.* –1.60 V and scanning in the negative direction first. **Bottom:** Repeat scans of **4**, scan 1 (red) followed by scan 2 (blue) showing a significant change to $E_{pc}(1)$ and $E_{pa}(1)$ because of scanning to positive potentials.

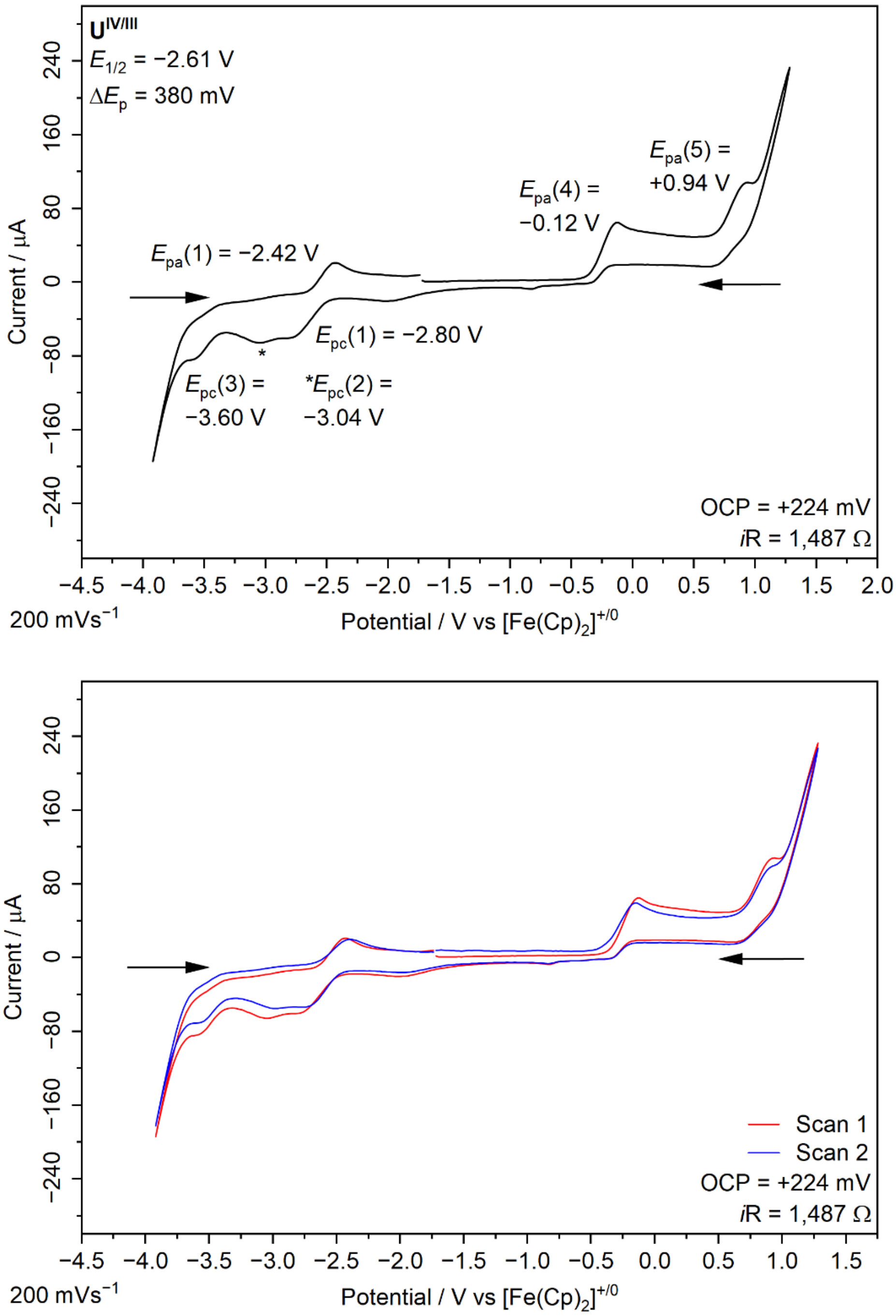

Figure S189. Top: Cyclic voltammogram of **4** (3.0 mM) in THF supported by $[nBu_4N][PF_6]$ (0.2 M) *vs* $[Fc]^{+/0}$ at 200 mV s^{-1}, starting at *ca.* –1.70 V and scanning in the positive direction first. **Bottom:** Repeat scans of **4**, scan 1 (red) followed by scan 2 (blue) showing no significant changes between subsequent scans, this suggests the oxidative events, E_{pa}(4) and E_{pa}(5) might influence the U(IV/III) couple of **4**.

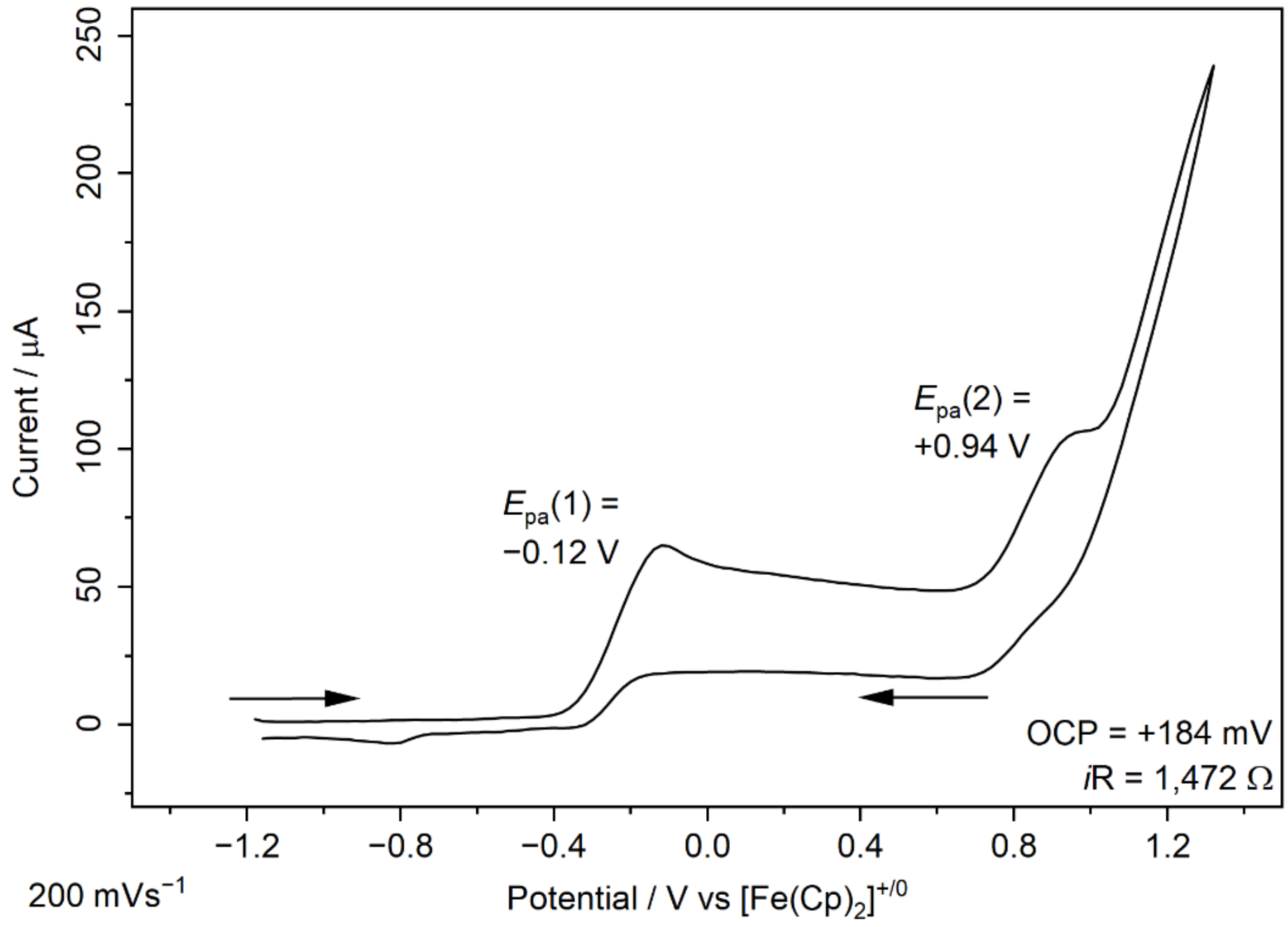

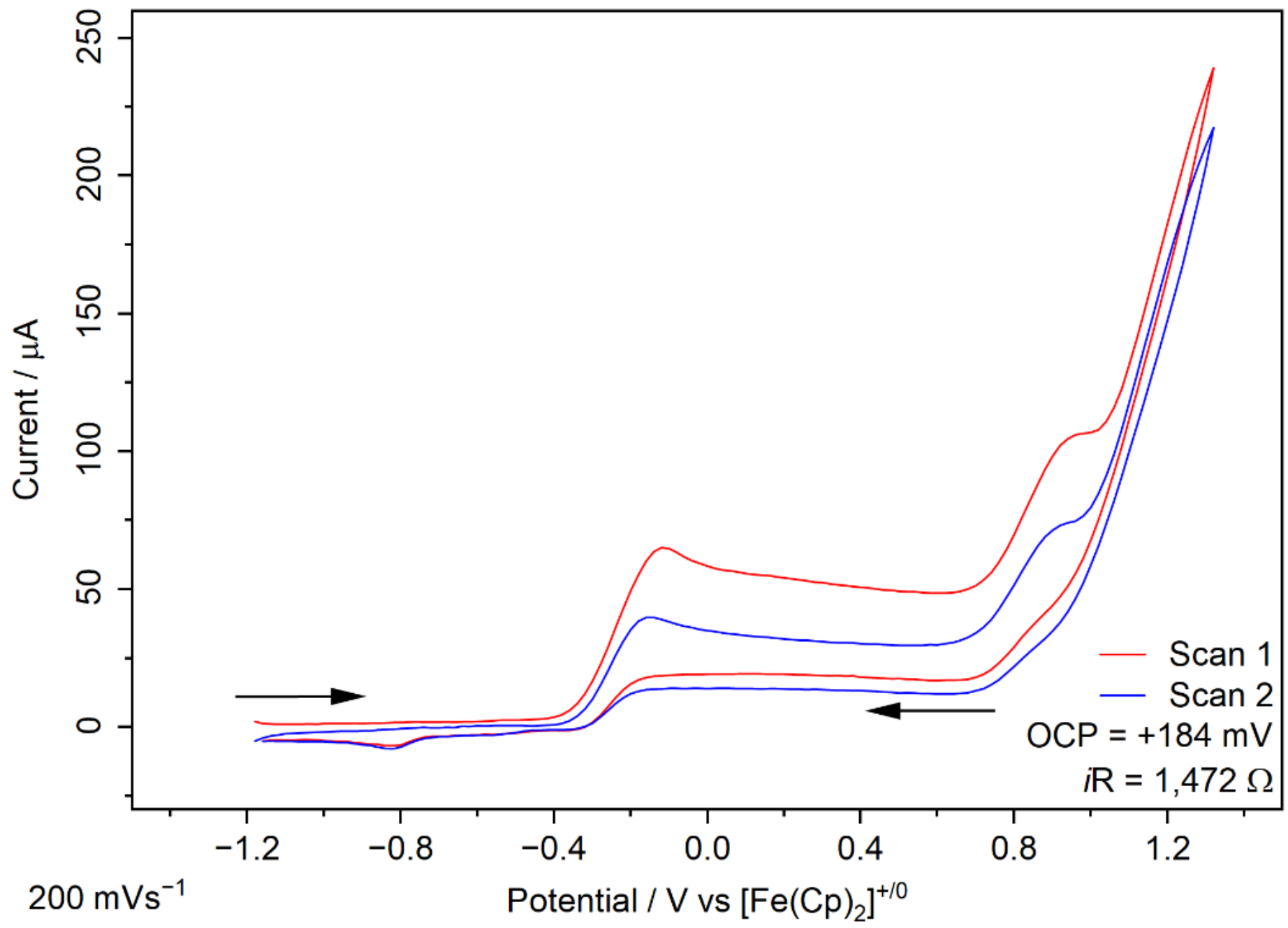

Figure S190. Top: Cyclic voltammogram of **4** (3.0 mM) in THF supported by [nBu$_4$N][PF$_6$] (0.2 M) *vs* $[Fc]^{+/0}$ at 200 mV s^{-1}, starting at *ca.* –1.20 V and scanning in the positive direction, only scanning between *ca.* –1.20 V and +1.30 V. **Bottom:** Repeat scans for the positive region of **4**, scan 1 (red) followed by scan 2 (blue) showing a decrease in current intensity for oxidative peaks E_{pa}(1) and E_{pa}(2).

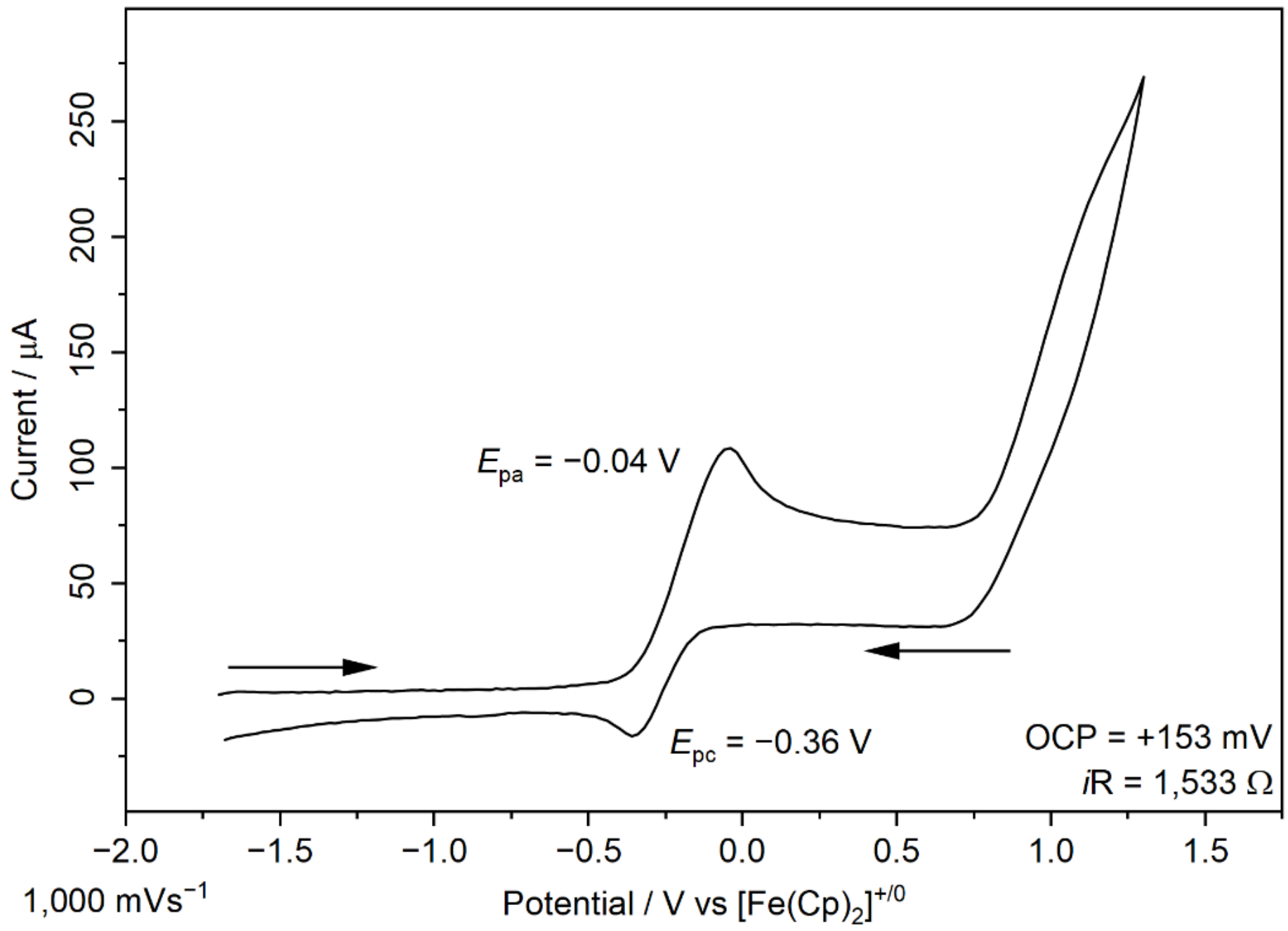

Figure S191. Cyclic voltammogram of **4** (3.0 mM) in THF supported by [nBu$_4$N][PF$_6$] (0.2 M) *vs* [Fc]$^{+/0}$ at 1,000 mV s^{-1}, starting at *ca.* –1.20 V and scanning in the positive direction first. At this higher scan rate, a reduction event was observed, E_{pc} = –0.36 V.

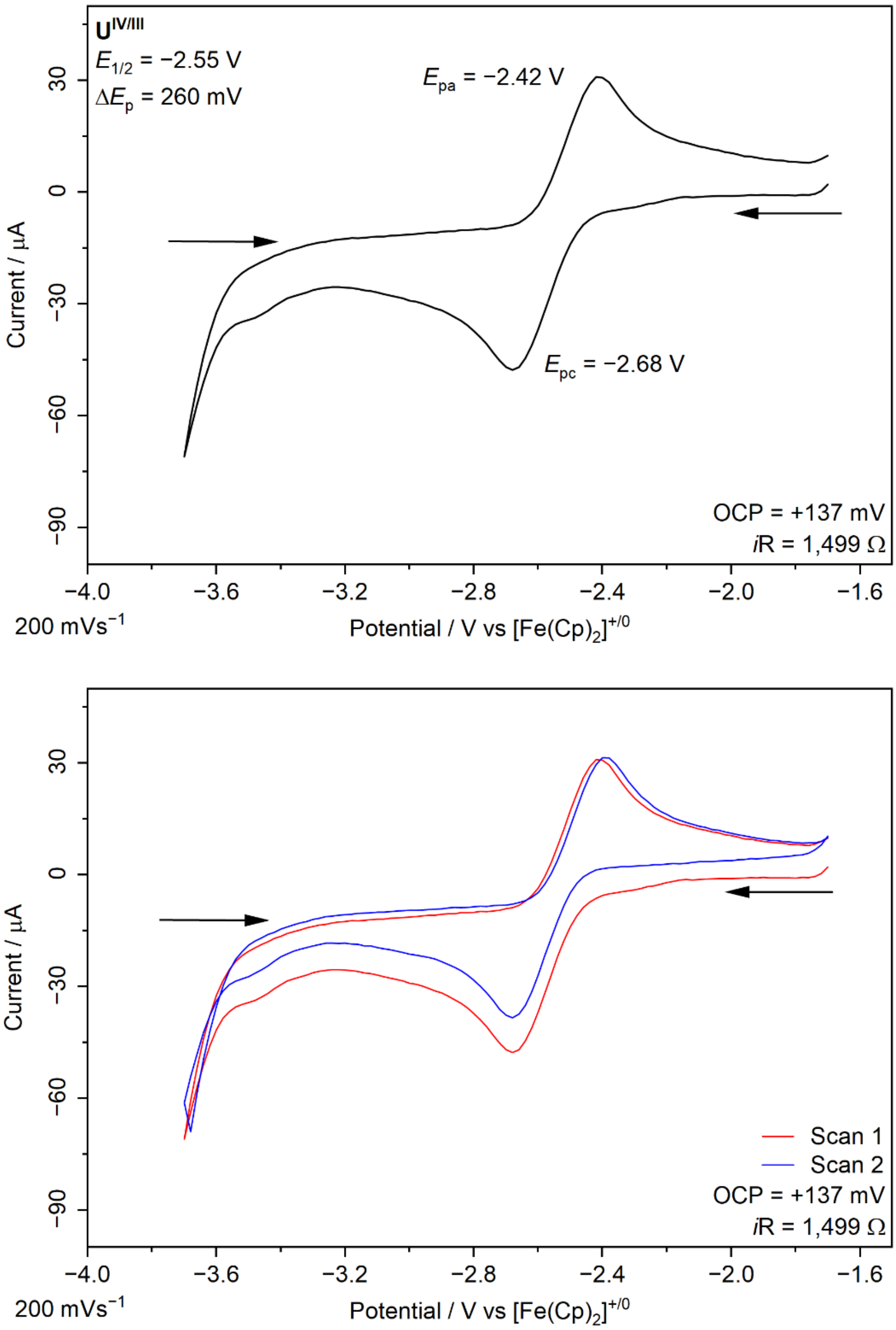

Figure S192. Top: Cyclic voltammogram of **4** (3.0 mM) in THF supported by [nBu$_4$N][PF$_6$] (0.2 M) *vs* [Fc]$^{+/0}$ at 200 mV s^{-1}, starting at *ca.* –1.70 V and scanning in the negative direction, only scanning between *ca*. –1.70 V and –3.70 V. **Bottom:** Repeat scans of **4**, scan 1 (red) and scan 2 (blue) showing a slight decrease in current intensity of E_{pc}.

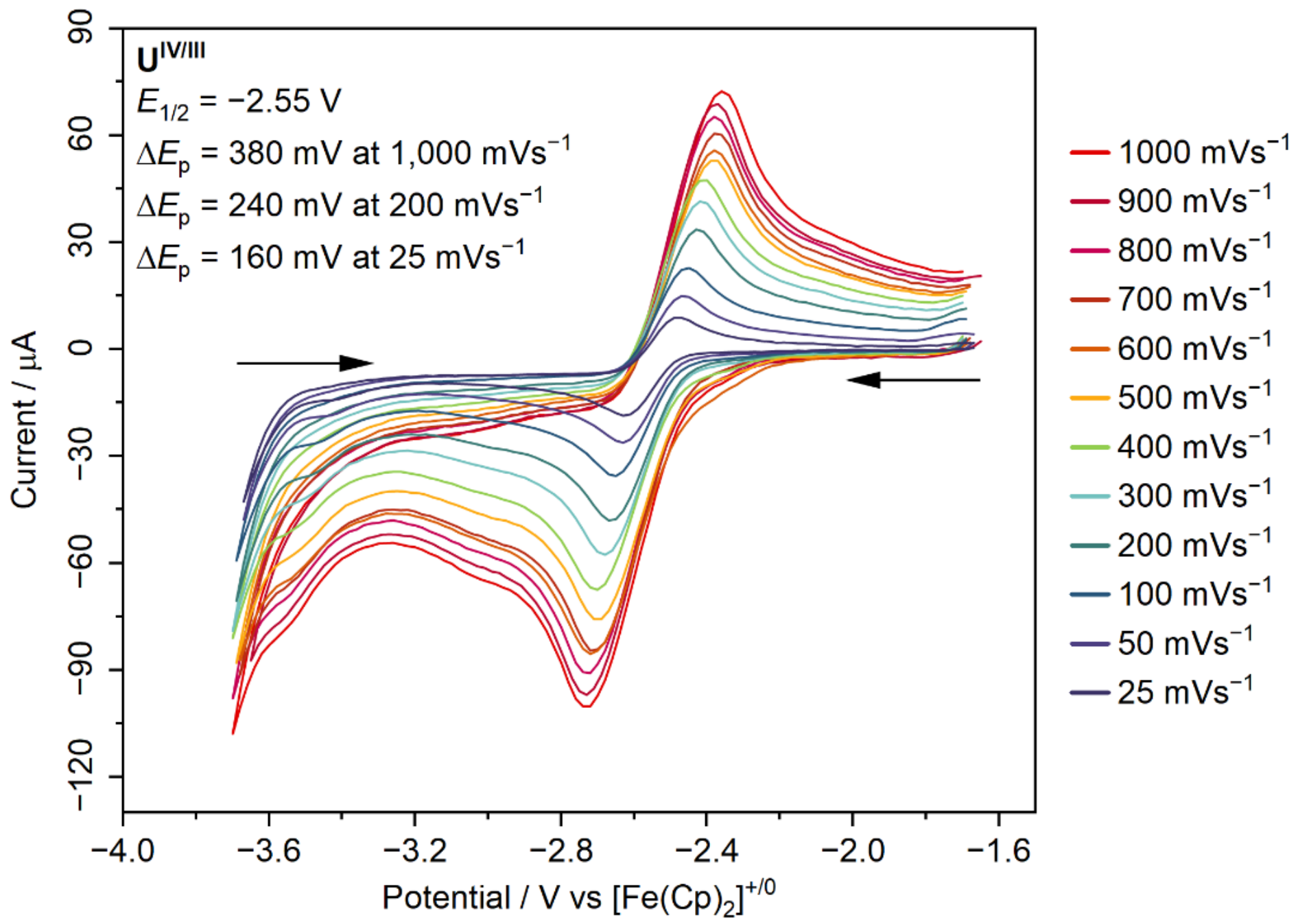

Figure S193. Cyclic voltammograms of the U(IV/III) couple of **4** (3.0 mM) in THF supported by [nBu$_4$N][PF$_6$] (0.2 M) *vs* $[Fc]^{+/0}$ showing the scan-rate dependence of the U(IV/III) couple.

Table S22. Electrochemical data for the U(IV/III) redox event *vs* $[Fc]^{+/0}$ for **4** (3.0 mM) in THF at room temperature, measured in 0.2 M [nBu$_4$N][PF$_6$].

Scan rate / mV s^{-1}	E_{pc} / V	E_{pa} / V	ΔE_p / mV	i_{pa}/i_{pc}	OCP / mV	iR / Ω
**25**	–2.63	–2.47	160	0.89	+73.4	1,509
**50**	–2.63	–2.47	160	0.89	+58.7	1,487
**100**	–2.65	–2.45	200	0.92	+28.7	1,490
**200**	–2.67	–2.43	240	0.97	+16.1	1,451
**300**	–2.68	–2.42	260	0.98	+4.51	1,433
**400**	–2.70	–2.40	300	0.97	+5.26	1,450
**500**	–2.71	–2.39	320	0.97	–6.74	1,458
**600**	–2.72	–2.38	340	0.93	+0.05	1,448
**700**	–2.72	–2.38	340	0.95	+3.33	1,446
**800**	–2.72	–2.38	340	0.97	–11.3	1,435
**900**	–2.73	–2.37	360	0.96	+46.2	1,392
**1,000**	–2.74	–2.36	380	0.98	–18.8	1,429

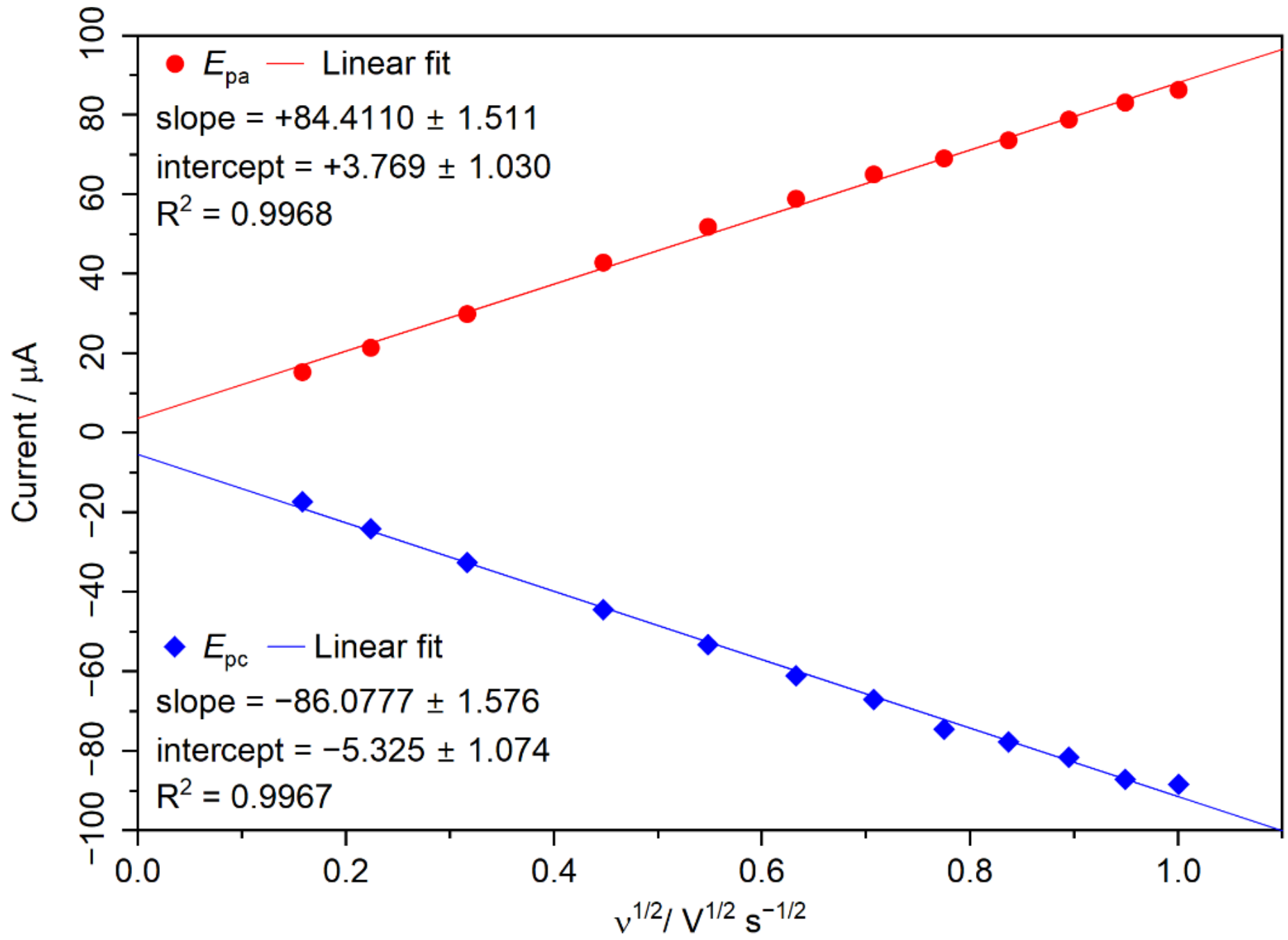

Figure S194. Randles-Sevcik plot for the U(IV/III) couple in **4**, measured in THF supported by [nBu$_4$N][PF$_6$] (0.2 M).

S7. SQUID Magnetometry

General details

Variable-temperature magnetic moment data were recorded in an applied direct current (DC) field of 0.1 Tesla on a Quantum Design MPMS3 superconducting quantum interference device magnetometer using recrystallized powdered samples. Measurements were performed in dc scan mode using 40 mm scan length and 6 s scan time. Samples were carefully checked for purity and data reproducibility between independently prepared batches. Samples were crushed with a mortar and pestle under an argon atmosphere and immobilized in an eicosane matrix within 400 MHz Wilmad borosilicate NMR tubes to prevent sample reorientation during measurements. The tube was flame-sealed under dynamic vacuum (10^{-3} mbar) to a length of approximately 3 cm and mounted in the centre of a drinking straw, with the straw fixed to the end of an MPMS 3 sample rod. Care was taken to ensure complete thermalization of the sample before each data point was measured by employing delays at each temperature point as well as a slow cooling rate (5 K/min from 300 to 100 K; 1 K/min from 100 to 1.8 K). The sample was held at 1.8 K for 60 minutes before isothermal magnetization measurements to account for slow thermal equilibration of the sample. Diamagnetic corrections were applied using tabulated Pascal constants. Measurements were corrected for the effect of the blank sample holders (flame-sealed Wilmad NMR tube and straw) and eicosane matrix.

Table S23. Mass of complex and eicosane used in samples analysed by SQUID magnetometry.

Complex	Chemical formula	M_r / g mol^{-1}	Sample mass / mg	Eicosane mass / mg
**1**	$[U^{IV}(COT)_2]$	446.33	22.90	14.95
**2**	$[Li(12\text{-crown-}4)(THF)][U^{III}(COT)_2]$	701.55	12.98	7.44
**3**	$[U^{V}(COT)_2][B(C_6F_5)_4]$	1125.31	17.52	10.78
**4**	$[U^{IV}(COT^{\dagger\dagger})_2]$	1071.69	25.70	16.90
**5**	$[Li(TMEDA)_2][U^{III}(COT^{\dagger\dagger})_2]\cdot(C_9H_{12})$	1431.12	15.51	9.05
**6**	$[U^{V}(COT^{\dagger\dagger})_2][B(C_6F_5)_4]$	1750.60	22.83	15.09
**10**	$[Li(TMEDA)_2][Ce^{III}(COT^{\dagger\dagger})_2]\cdot(C_9H_{12})$	1333.20	14.69	12.25

Variable-temperature SQUID magnetometry

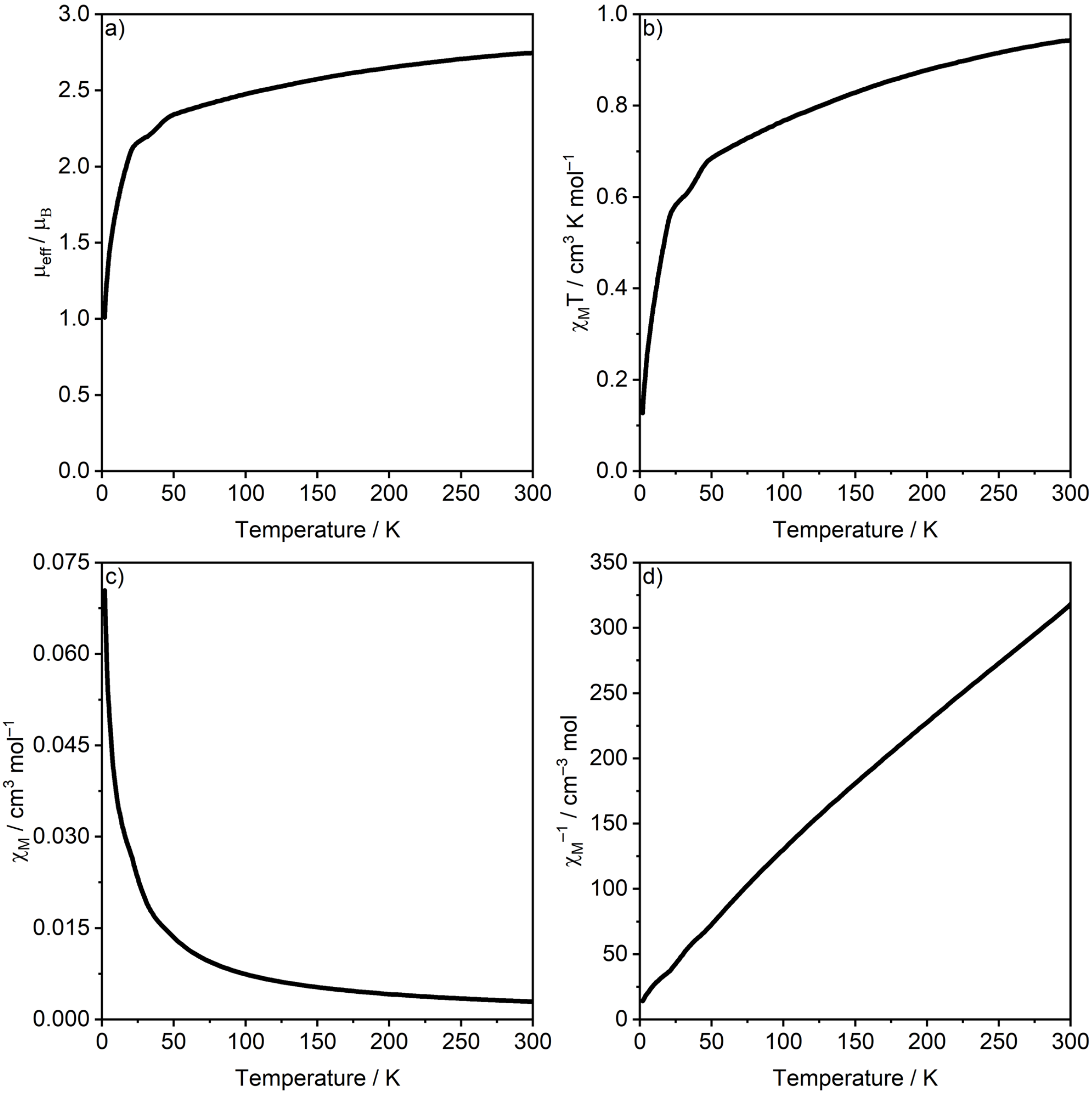

Figure S195. Variable-temperature SQUID magnetometry of **1** over the temperature range 1.8–300 K: (a) μ_{eff} vs T; (b) χ_MT vs T; (c) χ_M vs T; (d) $\chi_M{}^{-1}$ vs T.

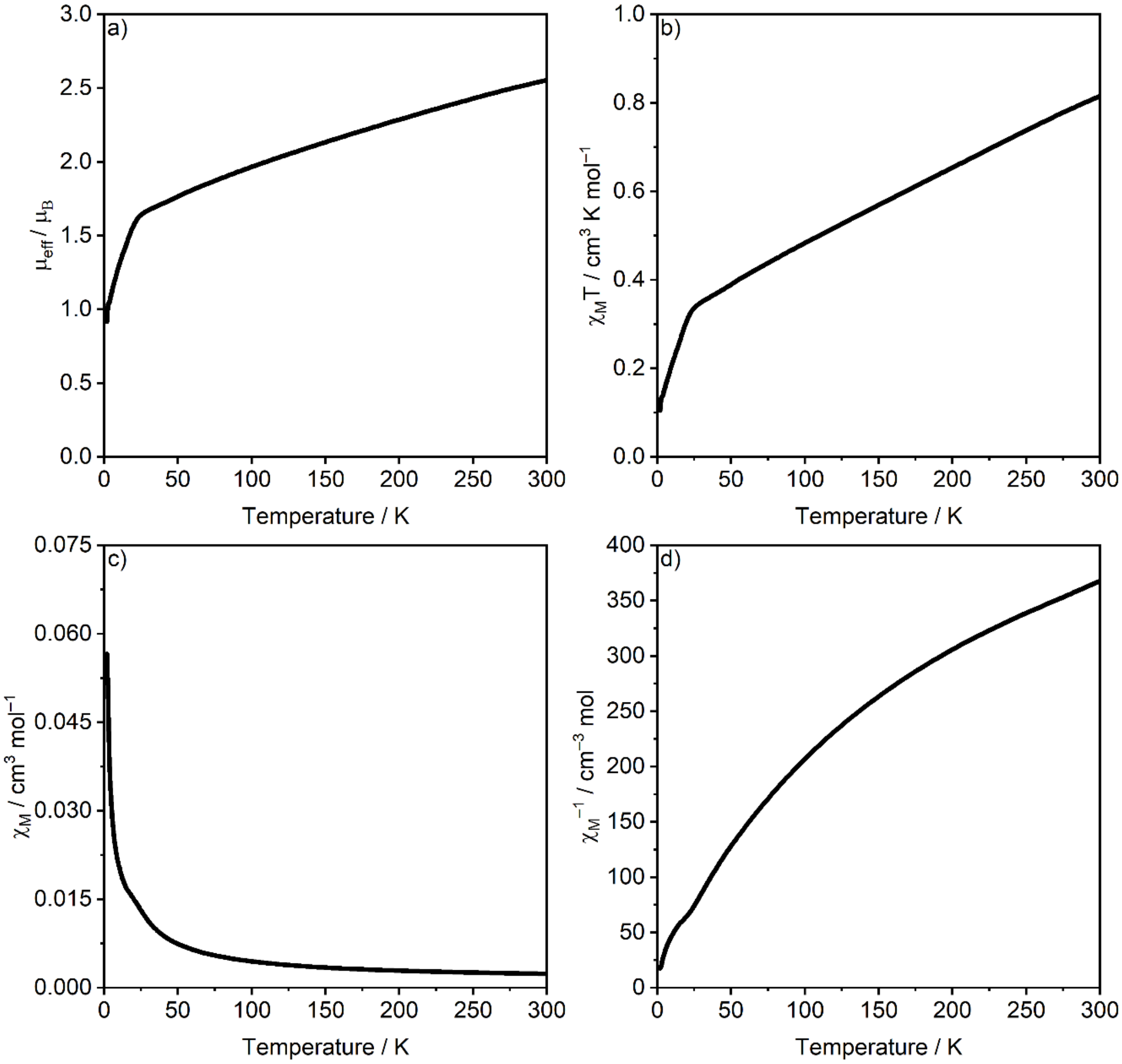

Figure S196. Variable-temperature SQUID magnetometry of **2** over the temperature range 1.8–300 K: (a) μ_{eff} vs T; (b) χ_MT vs T; (c) χ_M vs T; (d) $\chi_M{}^{-1}$ vs T.

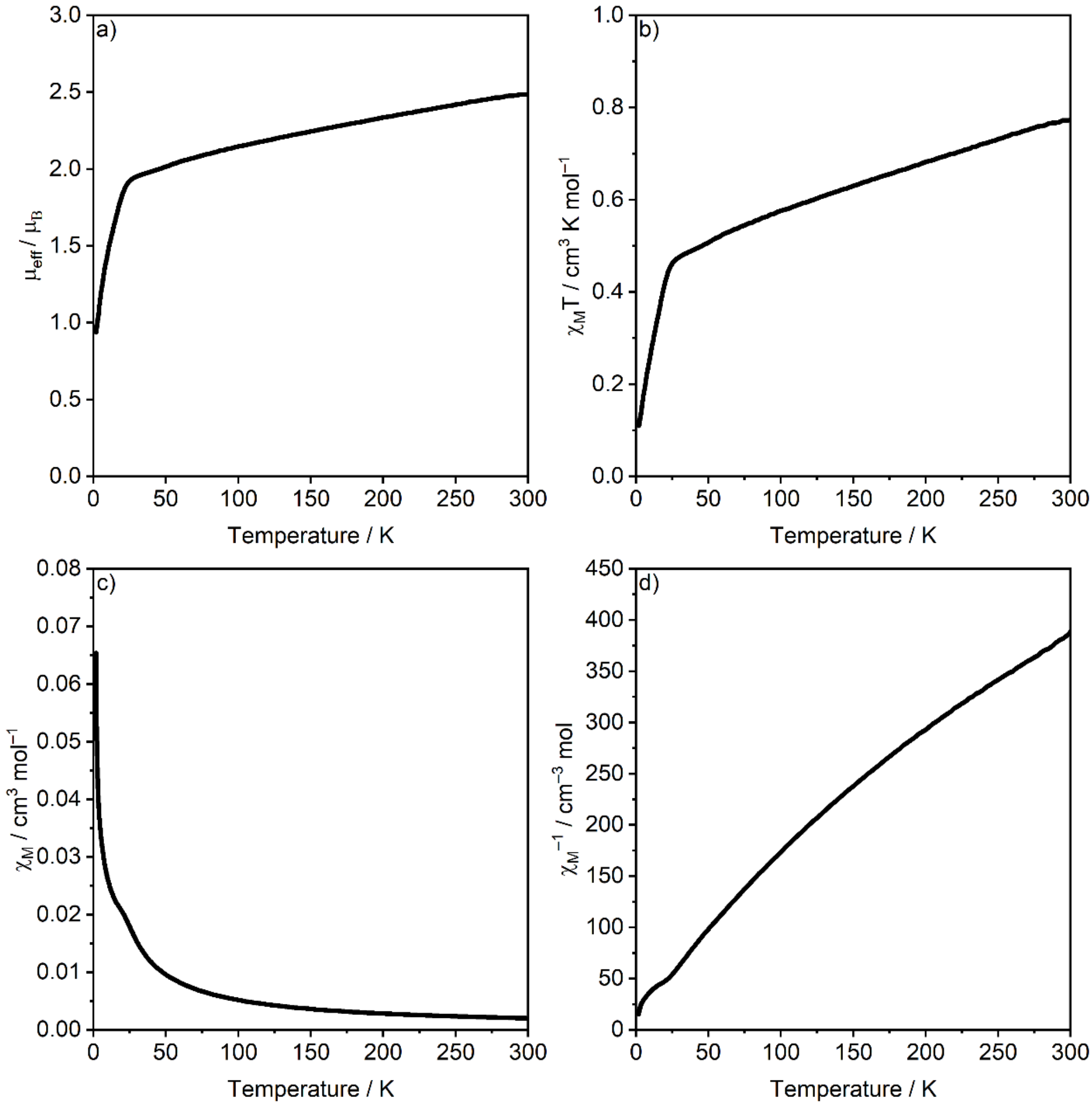

Figure S197. Variable-temperature SQUID magnetometry of **3** over the temperature range 1.8–300 K: (a) μ_{eff} vs T; (b) χ_MT vs T; (c) χ_M vs T; (d) $\chi_M{}^{-1}$ vs T.

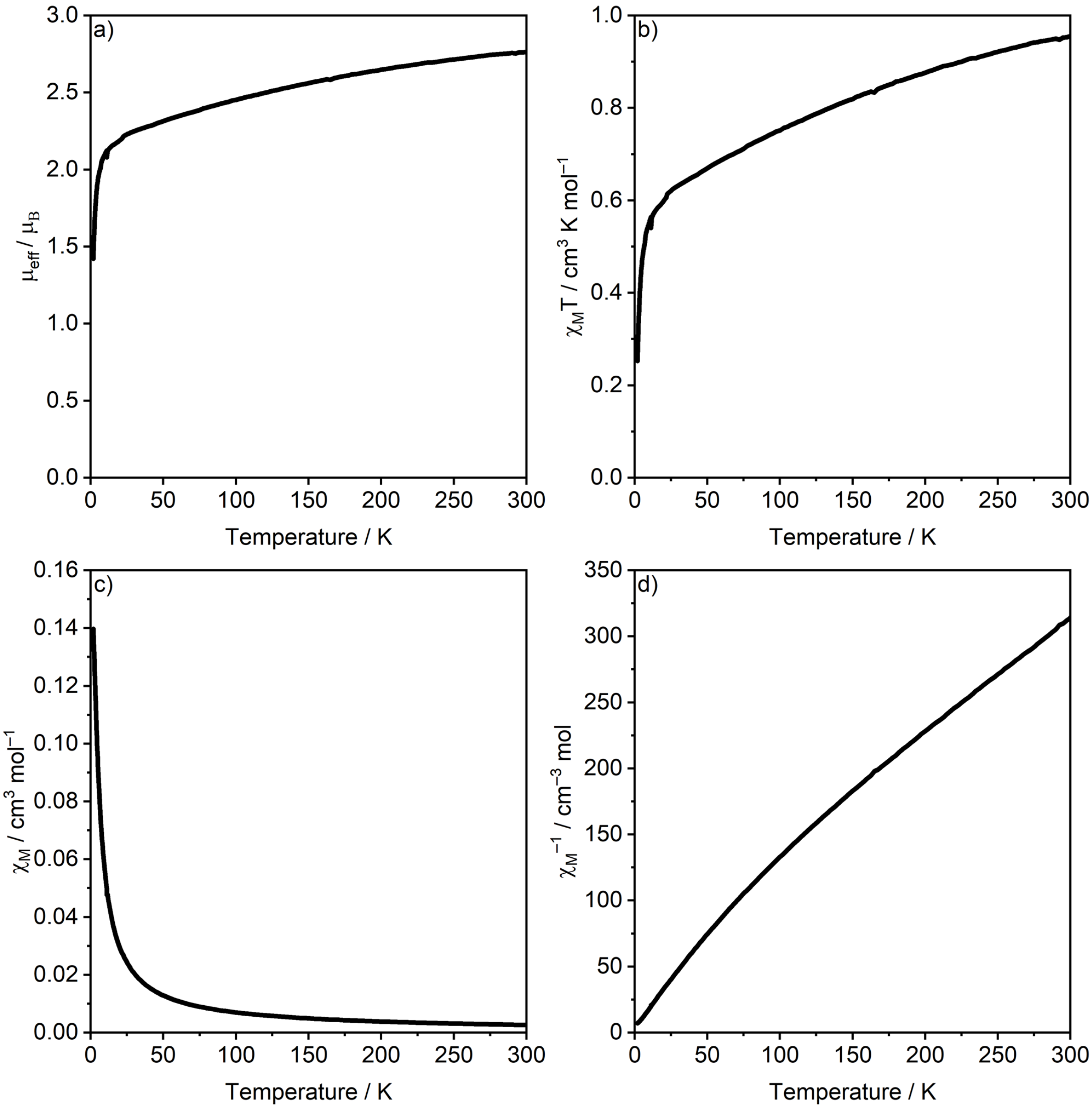

Figure S198. Variable-temperature SQUID magnetometry of **4** over the temperature range 1.8–300 K: (a) μ_{eff} vs T; (b) χ_MT vs T; (c) χ_M vs T; (d) $\chi_M{}^{-1}$ vs T.

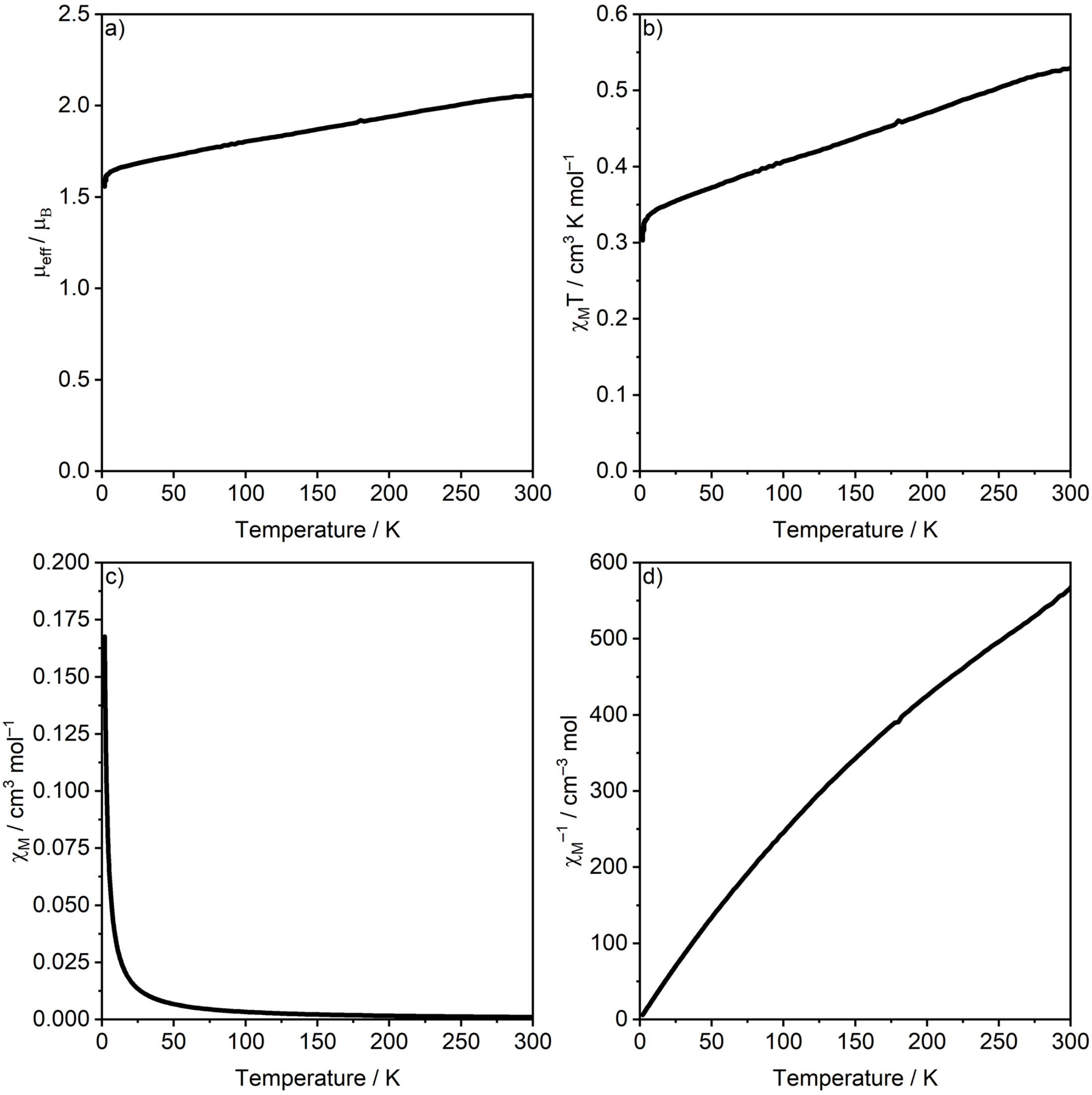

Figure S199. Variable-temperature SQUID magnetometry of **5** over the temperature range 1.8–300 K: (a) μ_{eff} vs T; (b) χ_MT vs T; (c) χ_M vs T; (d) $\chi_M{}^{-1}$ vs T.

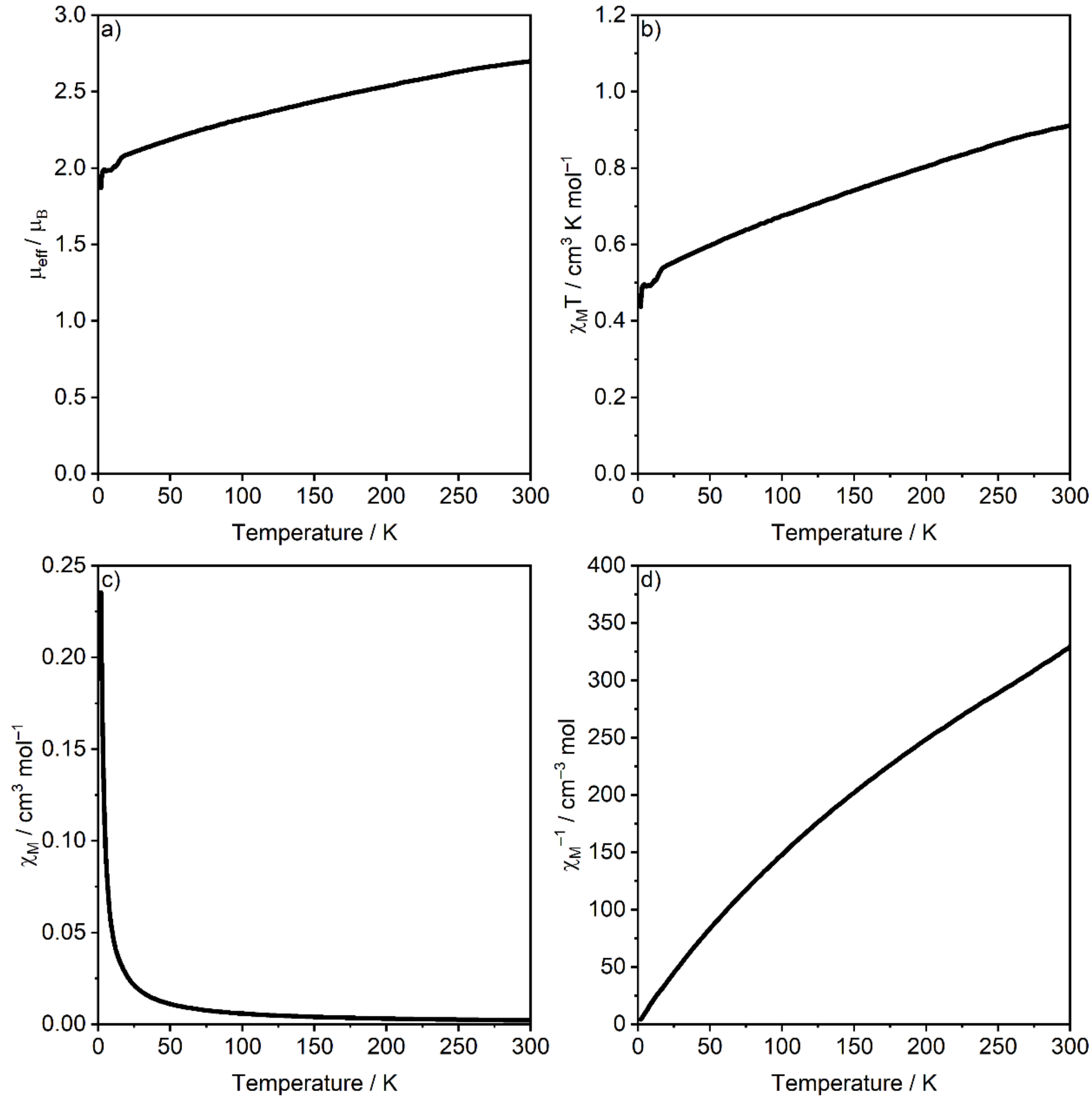

Figure S200. Variable-temperature SQUID magnetometry of **6** over the temperature range 1.8–300 K: (a) μ_{eff} vs T; (b) χ_MT vs T; (c) χ_M vs T; (d) ${\chi_M}^{-1}$ vs T.

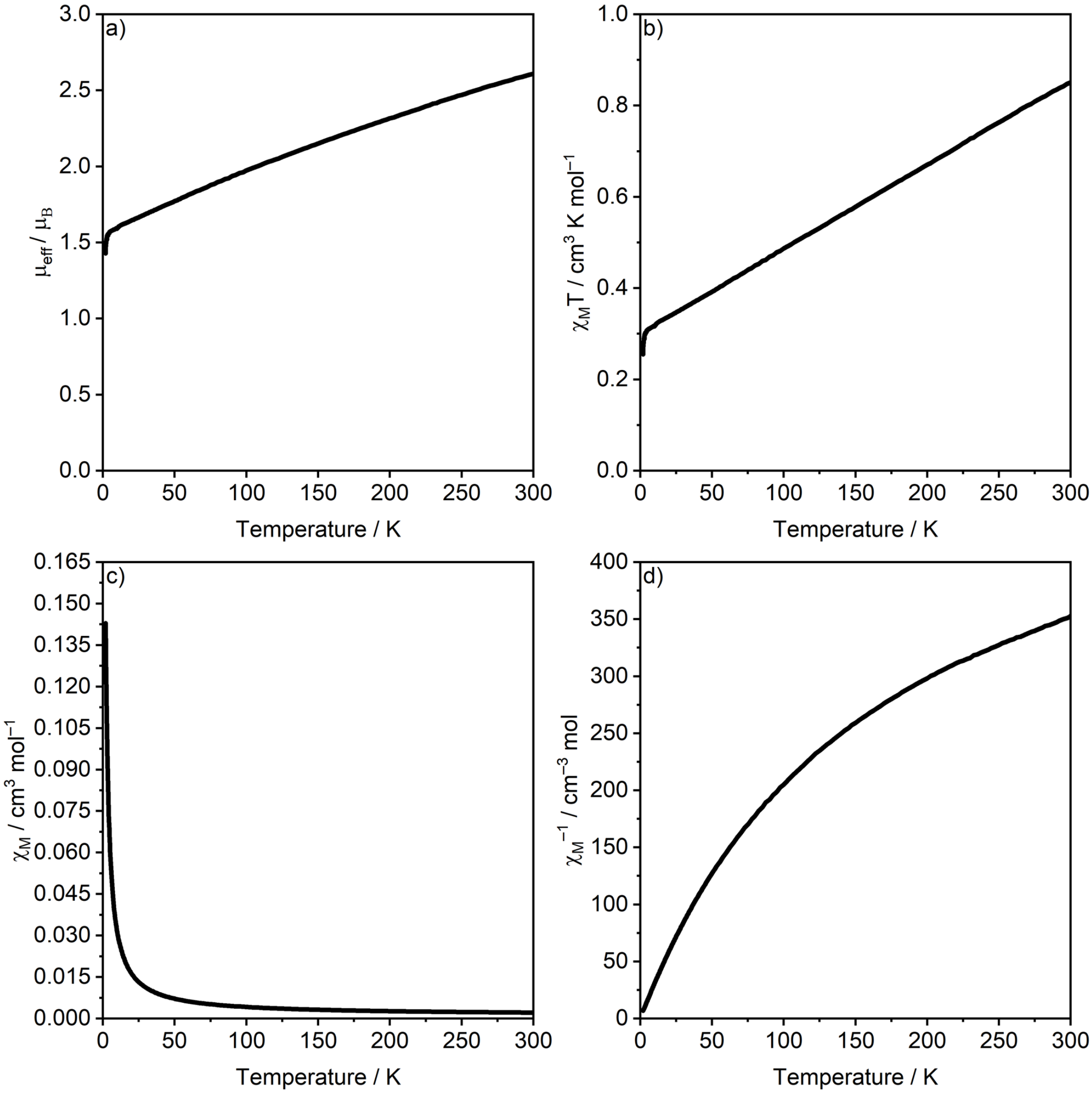

Figure S201. Variable-temperature SQUID magnetometry of **10** over the temperature range 1.8–300 K: (a) μ_{eff} vs T; (b) χ_MT vs T; (c) χ_M vs T; (d) $\chi_M{}^{-1}$ vs T.

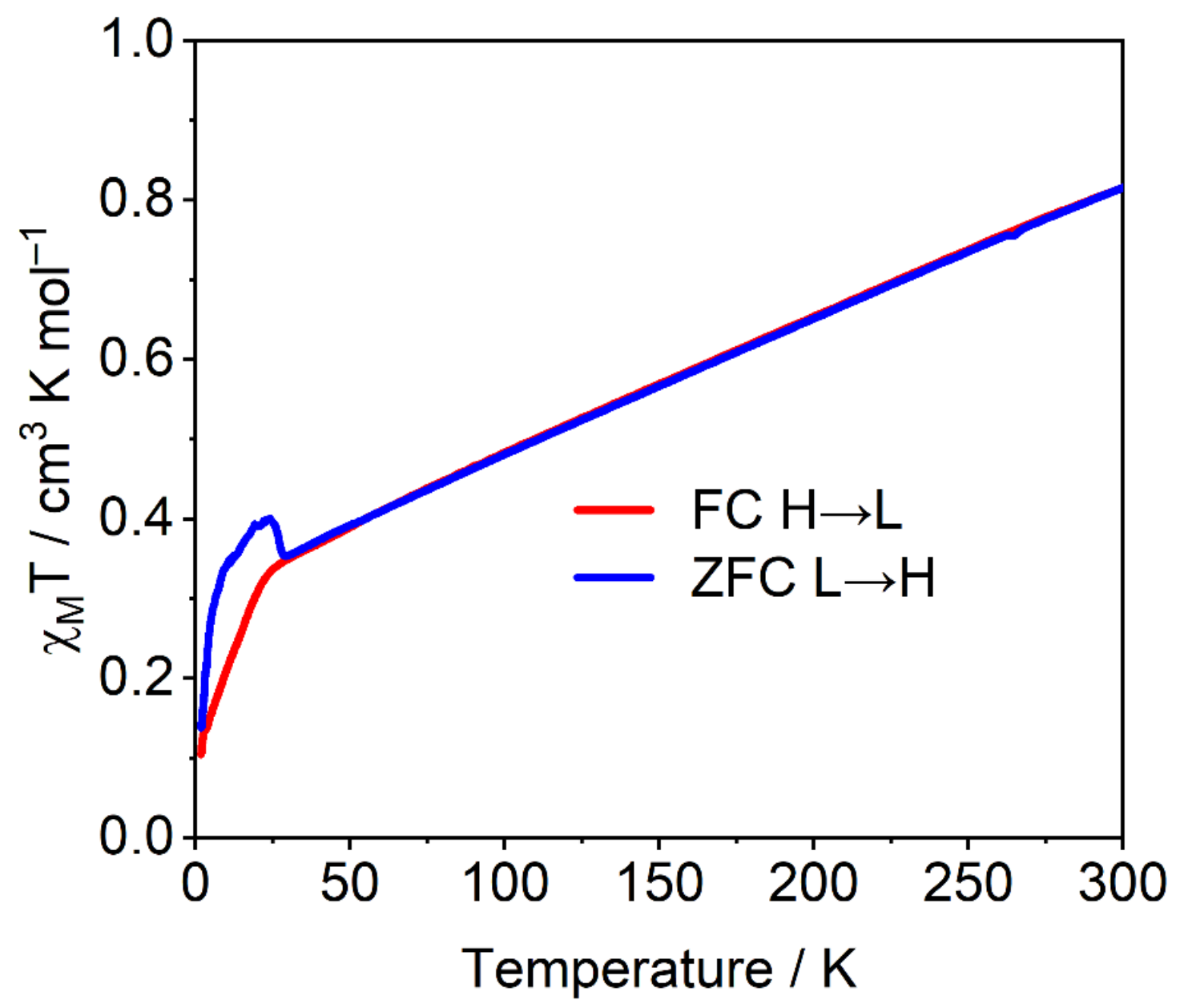

Figure S202. Variable-temperature SQUID magnetometry of **2** over the temperature range 1.8–300 K. Zero-field-cooled χ_MT vs T measured from 1.8K to 300 K (blue line) and field-cooled χ_MT vs T measured from 300 K to 1.8K (red line).

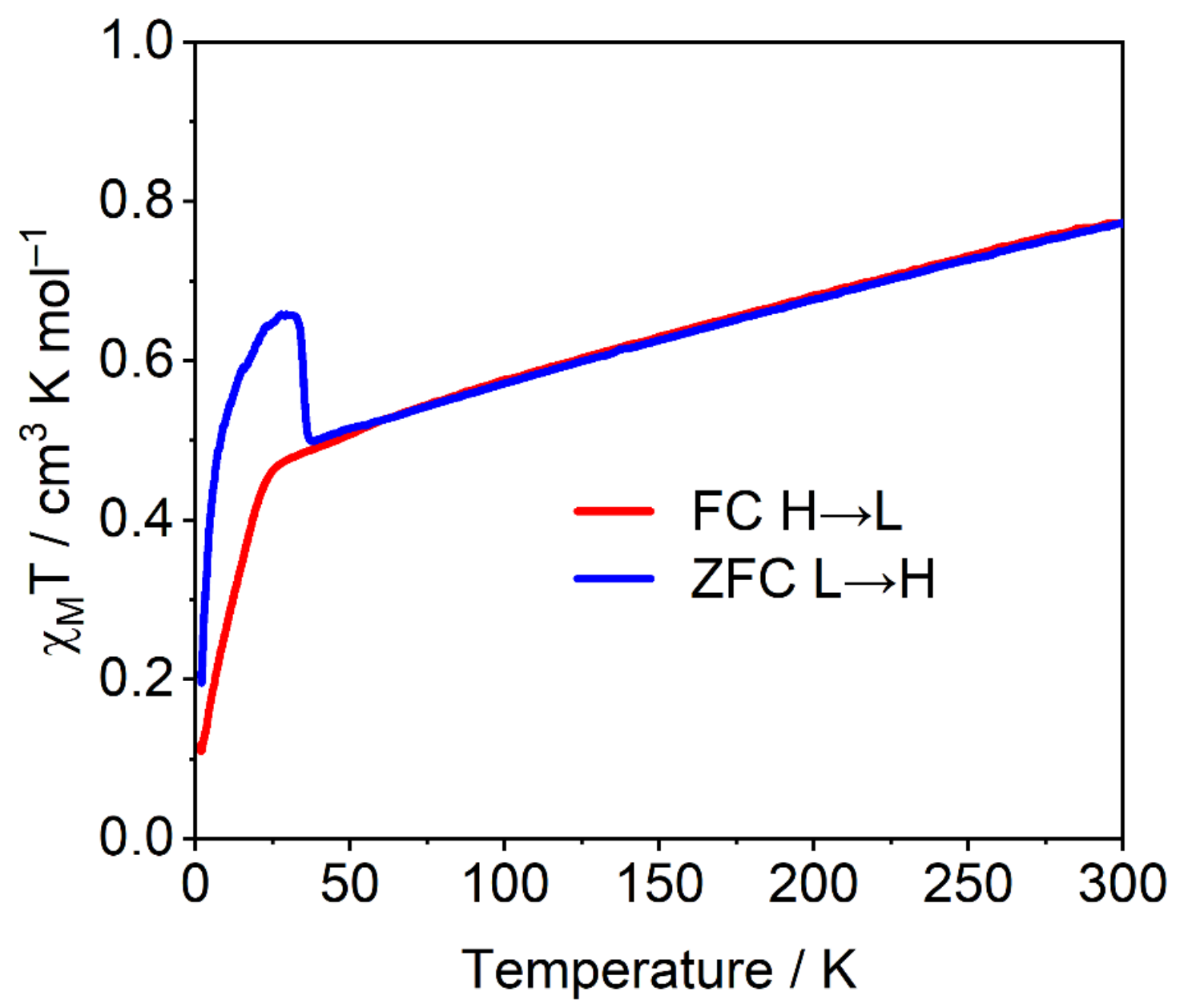

Figure S203. Variable-temperature SQUID magnetometry of **3** over the temperature range 1.8–300 K. Zero-field-cooled χ_MT vs T measured from 1.8K to 300 K (blue line) and field-cooled χ_MT vs T measured from 300 K to 1.8K (red line).

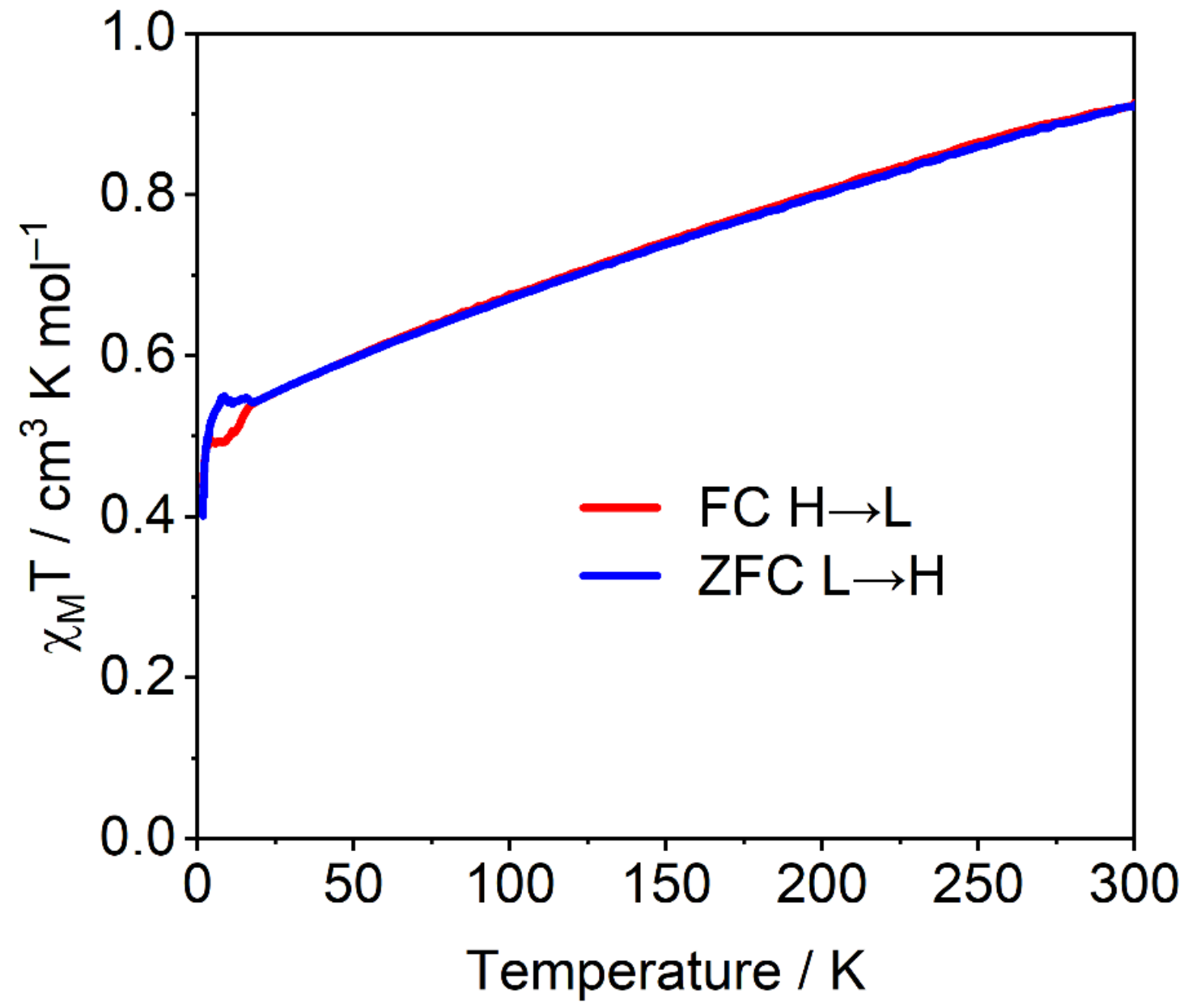

Figure S204. Variable-temperature SQUID magnetometry of **6** over the temperature range 1.8–300 K. Zero-field-cooled χ_MT vs T measured from 1.8K to 300 K (blue line) and field-cooled χ_MT vs T measured from 300 K to 1.8K (red line).

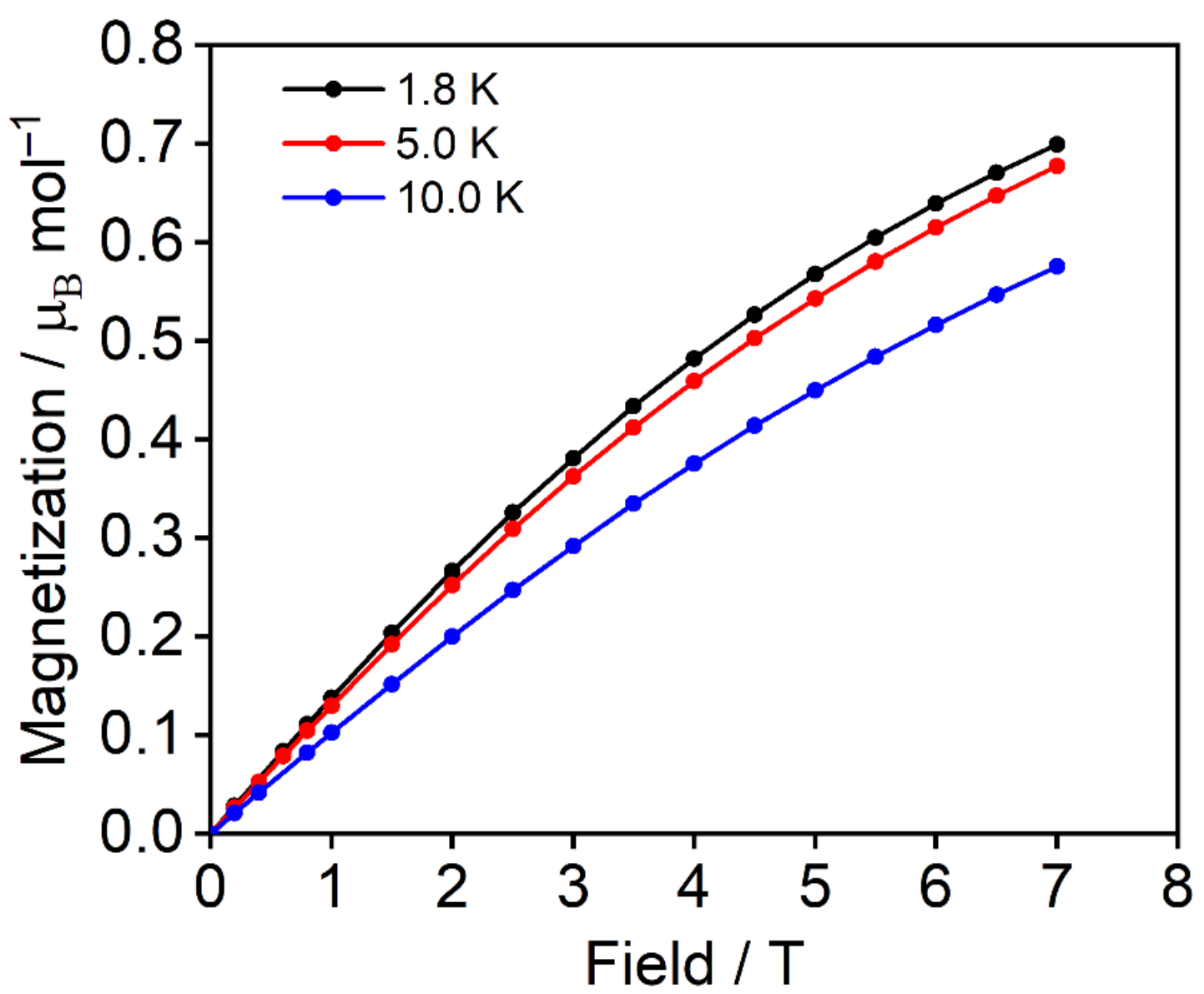

Figure S205. Variable-field SQUID magnetometry of **1** over the field range 0–7 T. M vs H data measured at 1.8 K (black line), 5 K (red line) and 10 K (blue line).

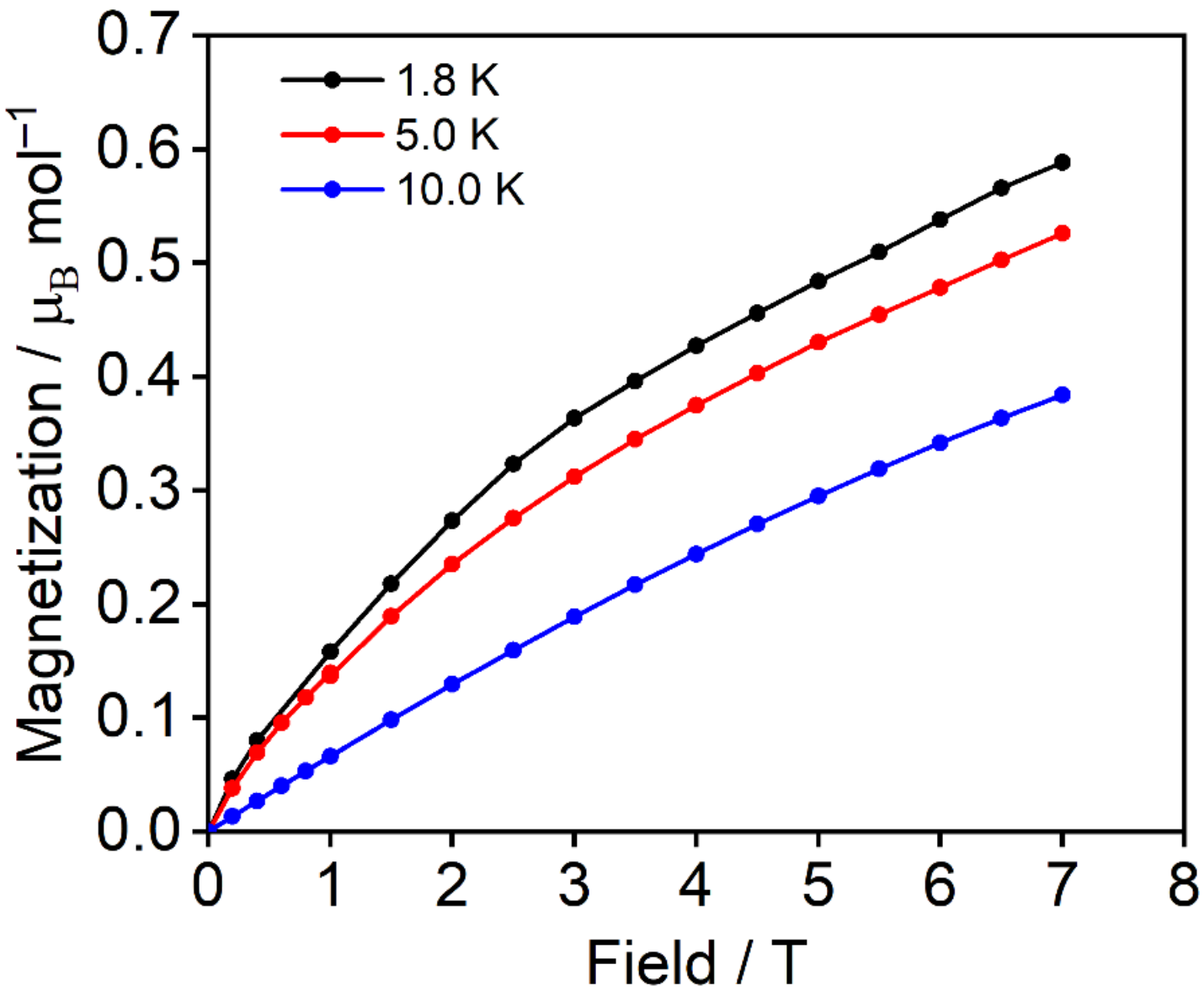

Figure S206. Variable-field SQUID magnetometry of **2** over the field range 0–7 T. M vs H data measured at 1.8 K (black line), 5 K (red line) and 10 K (blue line).

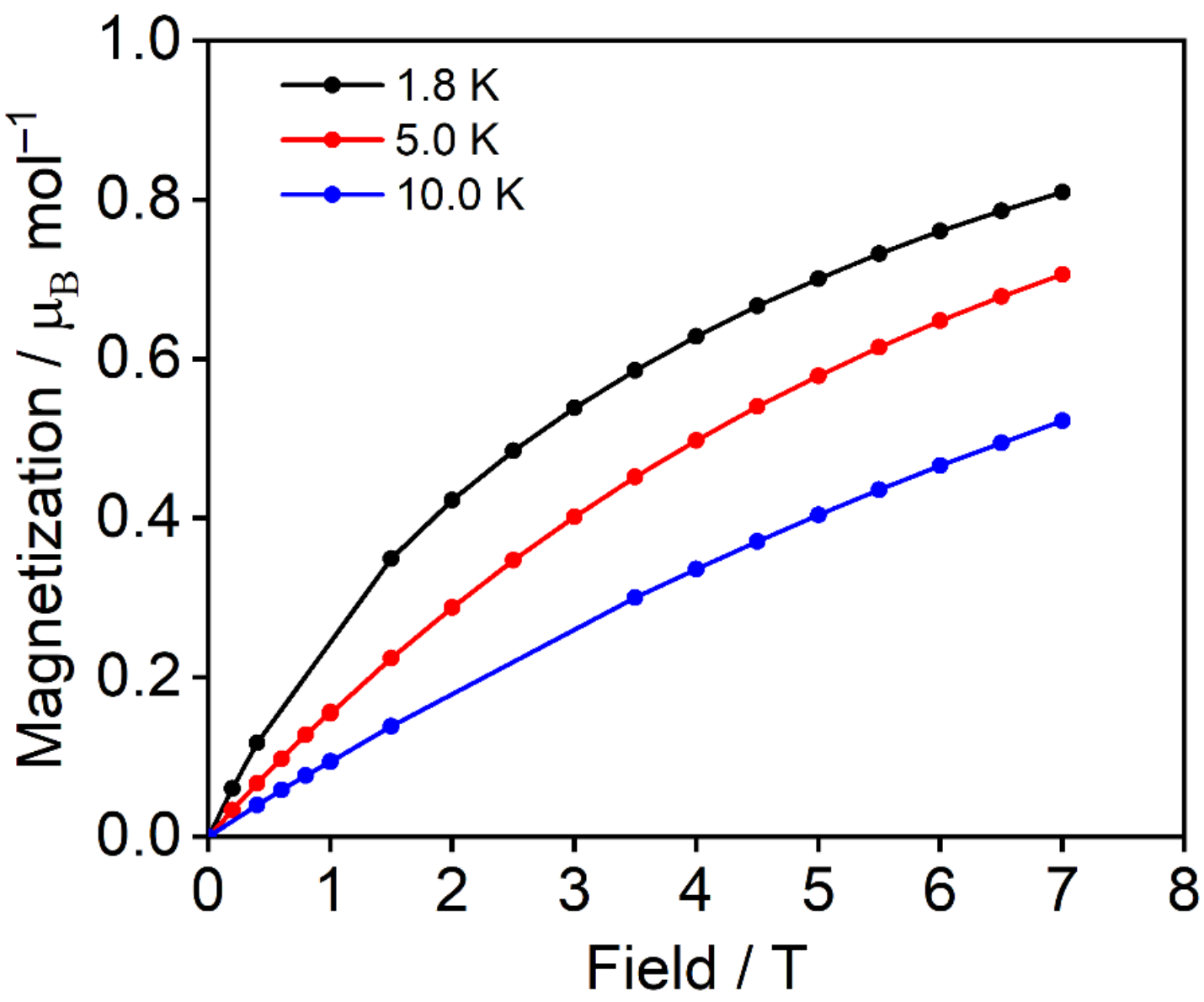

Figure S207. Variable-field SQUID magnetometry of **3** over the field range 0–7 T. M vs H data measured at 1.8 K (black line), 5 K (red line) and 10 K (blue line).

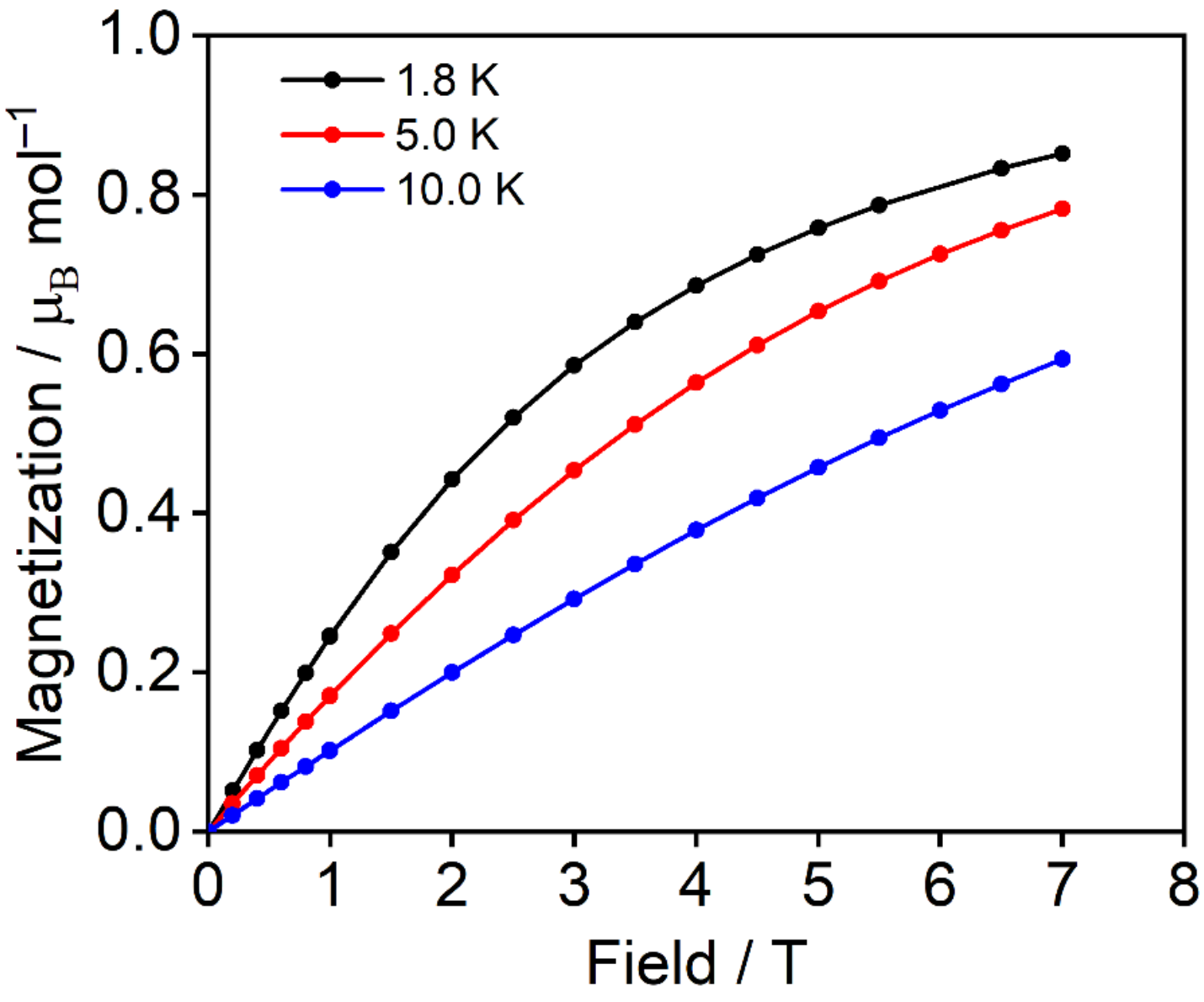

Figure S208. Variable-field SQUID magnetometry of **4** over the field range 0–7 T. M vs H data measured at 1.8 K (black line), 5 K (red line) and 10 K (blue line).

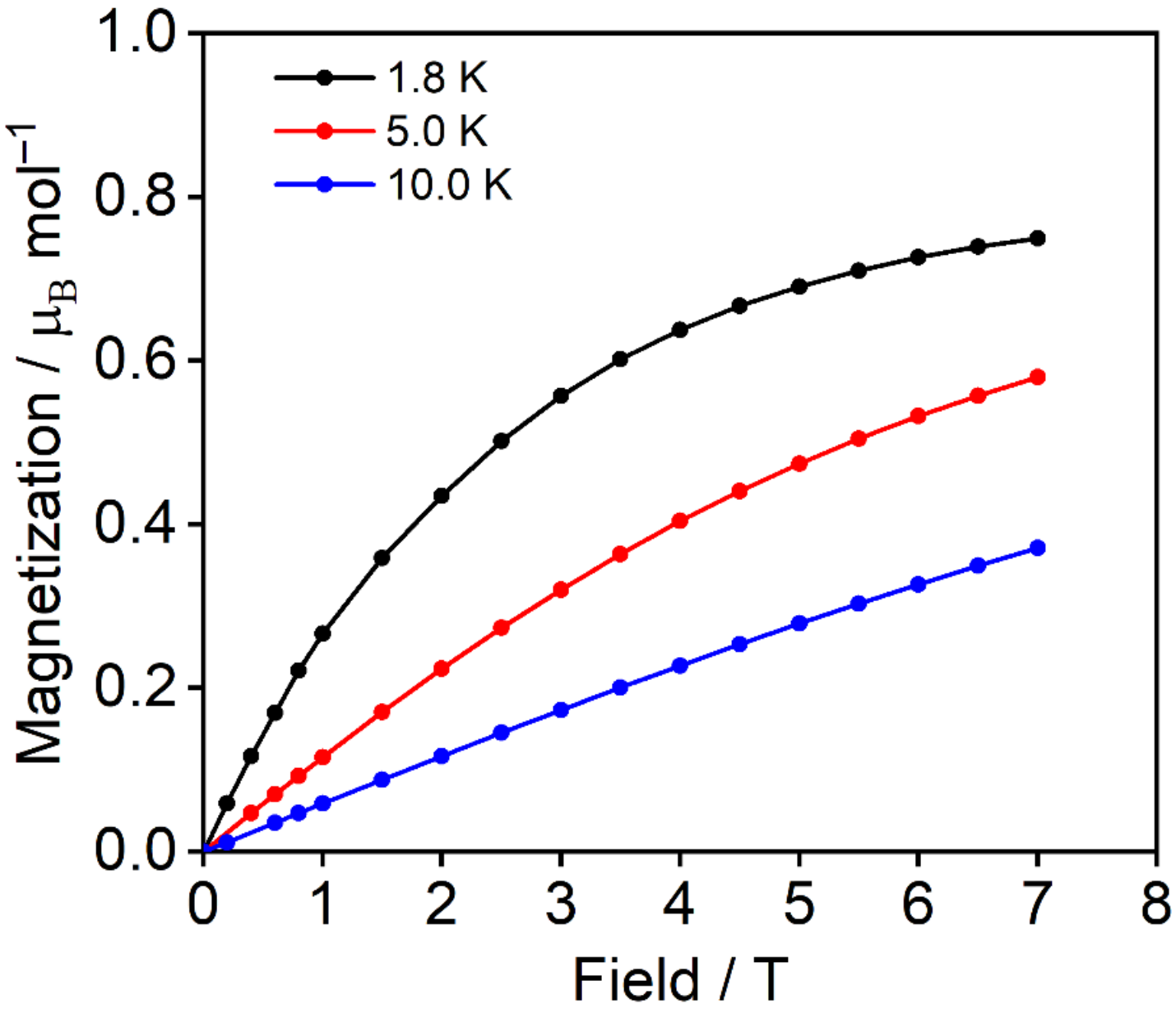

Figure S209. Variable-field SQUID magnetometry of **5** over the field range 0–7 T. M vs H data measured at 1.8 K (black line), 5 K (red line) and 10 K (blue line).

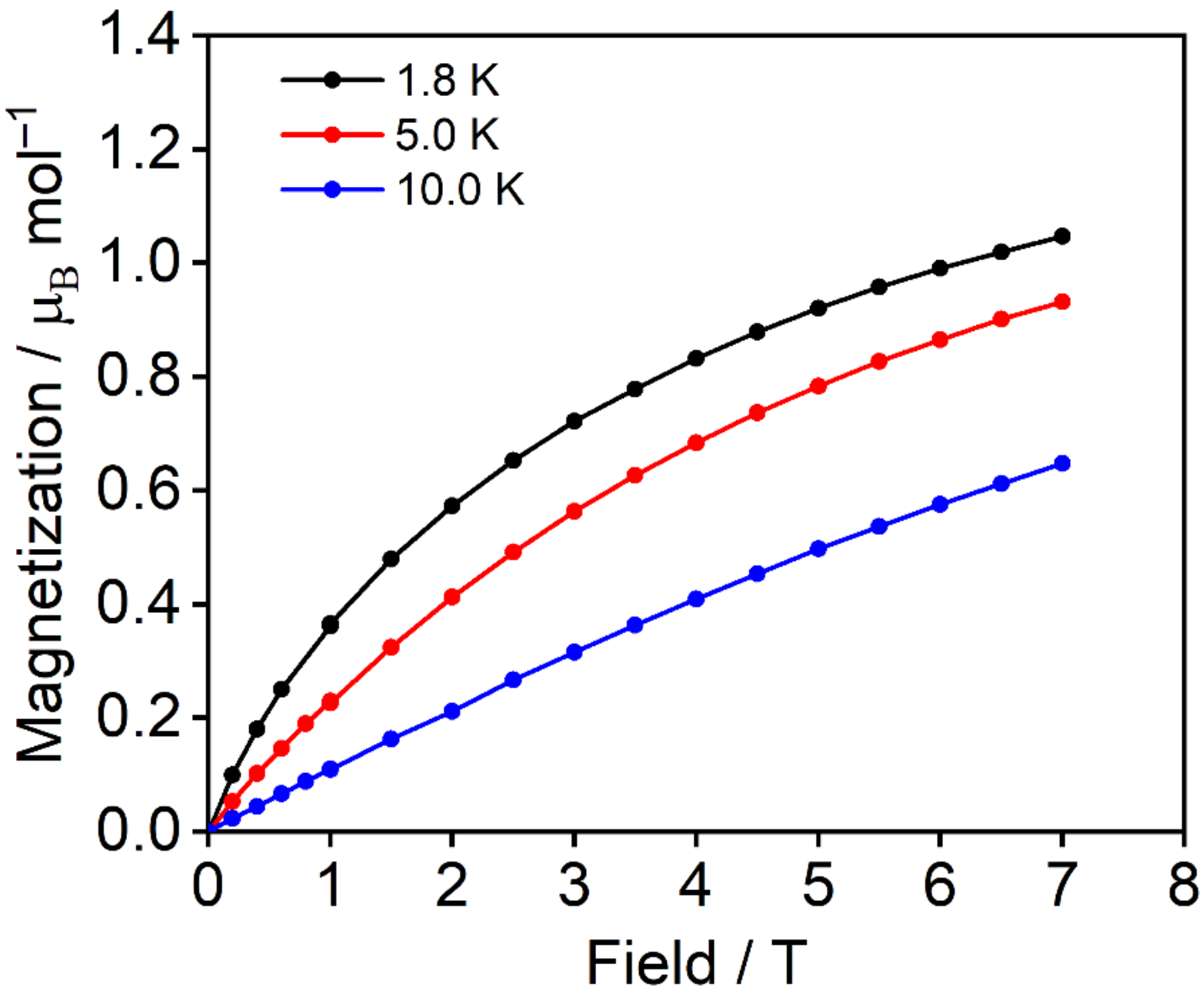

Figure S210. Variable-field SQUID magnetometry of **6** over the field range 0–7 T. M vs H data measured at 1.8 K (black line), 5 K (red line) and 10 K (blue line).

Analysis of magnetometry data

For all compounds, low-temperature χ_MT values were estimated by linear extrapolation of the linear high-temperature behaviour (T > 50 K) to base temperature (see χ_MT(experiment) in **Table S24**). This approach was used due to the abrupt decrease in χ_MT observed at low temperatures for all compounds (**Figure S195**–**Figure S201**), which is due to thermal relaxation effects as evidenced by divergence of zero-field-cooled and field-cooled χ_MT curves below these temperatures (**Figure S202**). Linear extrapolation, therefore, provides an approximation of the intrinsic low-temperature magnetic susceptibility, minimizing the influence of dynamic thermal effects.

This approach is further justified by the large energy gap between the ground and excited states in these families of actinocene and lanthanocene compounds, as evidenced by the high-temperature magnetic data and also supported by theoretical calculations [23-25]. Calculated energy gaps between the ground |m_J| doublets (or pseudo-doublets) and the first excited-states (≈ 1044, 631, 1,579, and 479 cm^{-1} for U(III), U(IV), U(V) and Ce(III) respectively in $[An/Ln(COT)_2]^{+/-}$) [23-25] greatly exceed the thermal energy at room temperature (≈ 207 cm^{-1}).

The low temperature χ_MT values from this analysis are in good agreement with those predicted theoretically from the experimentally observed ground-state effective *g*-values by EPR (**Table S24**).

Table S24. Ground state magnetic susceptibilities at low temperature as determined by extrapolation of the linear behaviour observed above 50 K.

	**M(OS) f^n**	**Chemical formula**	χ_M**T (EPR)** [A] **/ cm^3 K mol^{-1}**	χ_M**T (theory)** [B] **/ cm^3 K mol^{-1}**	χ_M**T (experiment)** [C] **/ cm^3 K mol^{-1}**
**2**	U(III)	$[Li(12\text{-crown-4})(THF)][U^{III}(COT)_2]$	–	0.41	0.31
**5**	$5f^3$	$[Li(TMEDA)_2][U^{III}(COT^{\dagger\dagger})_2]\cdot(C_9H_{12})$	0.35	0.41	0.34
**1**	U(IV)	$[U^{IV}(COT)_2]$	–	0.72	0.66
**4**	$5f^2$	$[U^{IV}(COT^{\dagger\dagger})_2]$	–	0.72	0.64
**3**	U(V)	$[U^{V}(COT)_2][B(C_6F_5)_4]$	0.53	0.44	0.47
**6**	$5f^1$	$[U^{V}(COT^{\dagger\dagger})_2][B(C_6F_5)_4]$	0.58	0.44	0.54
**10**	Ce(III) $4f^1$	$[Li(TMEDA)_2][Ce^{III}(COT^{\dagger\dagger})_2]\cdot(C_9H_{12})$	0.37	0.44	0.30

[A] Values calculated using **Eq. S4.** and the *g*-values from the ground Kramers Doublet as measured by EPR spectroscopy (see **Table S26**). [B] Theoretical values as determined by **Eq. S4.** using the effective *g*-values obtained when assuming pure $m_J = \pm 5/2$, $\pm 1/2$ and $\pm 1/2$ doublets for U^{III}, U^{V} and Ce^{III} species, respectively, and ± 3 m_J pseudo-doublet for U^{IV} (see *g*-values in **Table S27** with $g_{\perp}^{eff} = g_{xx} = g_{yy}$ and $g_{//}^{eff} = g_{zz}$). The ground state for U^{IV} is taken as a ± 3 m_J pseudo-doublet as found by calculations by Singh *et al.*[23] [C] χ_MT values linearly extrapolated to 0 K from variable-temperature SQUID magnetometry experiments.

$$\chi_M T_{(Low\ temp.)} = \frac{N_A \mu_B^2}{k_B} \times g_{eff}^2 \times s_{eff}(s_{eff} + 1)$$

Eq. S4.

With $g_{eff} = \sqrt{\frac{g_{xx}^2 + g_{yy}^2 + g_{zz}^2}{3}}$ and S_{eff} = ½, which gives in cgs units:

$$\chi_M T_{(Low\ temp.)} = \frac{1}{32} \times (g_{xx}^2 + g_{yy}^2 + g_{zz}^2)$$

S8. EPR Spectroscopy

General details

Continuous wave X Band (9.4GHz) EPR measurements were carried out using a Bruker EMX EPR spectrometer equipped with a Bruker ER-4122SHQ resonator. Cryogenic temperatures were achieved using a ColdEdge Stinger closed cycle helium system mated to an Oxford Instruments ESR900 cryostat. Temperature control and maintenance was handled via an Oxford Instruments Mercury ITC.

The air sensitive samples were loaded into clear fused quartz EPR tubes (Ilmasil® PN, QSIL, Germany) with 4 mm outer diameter and 3 mm inner diameter, glass-sealed in a glovebox, and flame-sealed under vacuum using an oxygen-propane torch outside the glovebox. The mass of each sample was recorded to enable relative comparison (see **Table S25**).

The experimental parameters used in these measurements were: microwave power 10 – 20 dB (2.2 – 22.2 mW), modulation amplitude 0.2 – 1 mT, modulation frequency 100 kHz, time constant 20.48 ms, conversion time 7.05 ms, sweep time 120 s, receiver gain 30 dB and an average microwave frequency of 9.42 GHz. For parameters that were varied between measurements, the specific values used for each recorded spectrum are summarized in **Table S25**.

All samples were measured in non-saturating conditions as confirmed by power dependent measurements. Field offset correction was carried out by comparison against the Bruker Strong Pitch standard (g = 2.0028) with the same parameters as the sample measurement. Measurement frequency was scaled post-measurement to 9.4 GHz to enable comparison across samples.

Experimental parameters

Table S25. Summary of measurement parameters for obtaining the powder CW-EPR spectra.

Complex	Powder	Sample Mass / mg	Microwave power / mW	Modulation amplitude / mT	Temp. / K
**2** U(III)	Unground	14.68	2.23	0.2	5, 100, 298
**5** U(III)	Unground	34.8	22.26	1	5
**3** U(V)	Ground	11.92	7.03	1	5
**6^A^** U(V)	Ground	30.04	2.23	0.2	5
**6^B^** U(V)	Ground	30.04	7.04	1	5
**10** Ce(III)	Ground	28.01	2.23	0.2	5

[A] Spectrum recorded shortly after sealing and showing important crystallinity effects – see **Figure S214**. [B] Spectrum recorded 2 months after sealing of sample **6**[A] and storage at –20°C, showing reduced crystallinity effects compared to **6**[A] which could be caused by loss of lattice solvent – see **Figure S215**.

Prompt sample measurement was required for the U(III) samples (**2** and **5**) because they were somewhat unstable when stored at –20°C, unlike the U(V) complexes (**3** and **6**).

Summary of experimental and simulated EPR parameters

To determine the experimental *g*-values, the relevant spectra (see below) were simulated with an effective *S* = ½ model using EasySpin[26] incorporated in MatLab[27] resulting in the parameters found in **Table S26.** The function "pepper" was used for simulation of the powder spectra. In the case of **6**, the differences in simulation parameters are justified as there are two molecules in the asymmetric unit with slightly different geometries (see geometries of **6 (U1)** and **6 (U2)** in **Table S8**).

Table S26. Summary of parameters derived from EPR for **2**, **3**, **5**, **6**, and **10**.

Complex	g_{xx}	g_{yy}	g_{zz}	g-strain (xx)	g-strain (yy)	g-strain (zz)	Weight
**2** U(III)	No EPR signal			–	–	–	–
**5** U(III)	0.033 [B]		3.336	0.007 [B]		0.057	–
**3** U(V)	2.827		1.057	0.162		0.036	–
**6** U(V) [A]	3.014	2.954	0.997	0.050	0.040	0.030	1.099
	2.955	2.887	1.027	0.070	0.035	0.030	0.902
**10** Ce(III)	2.352	2.252	1.090	0.037	0.046	0.011	–

[A] Simulated as two U^{V} species with different weights. [B] g_{xx} and g_{yy} must be < 0.4 as we do not observe them experimentally in the field range of the electromagnet (0 to 1.7 T). Arbitrarily small values of g_{xx}=g_{yy}=0.033 and *g*-strain(xx)=*g*-strain(yy)=0.007 were used for the simulations.

Table S27. Effective *g*-value predictions assuming pure m_J states with effective spin $S = ½$ for the ground J-multiplet of U(III), Ce(III) / U(V) and U(IV). In bold are the $|m_J|$ states identified as ground states for the respective metal ions and predicted effective *g*-values.

Metal ion	Electronic configuration	Ground RS term [A]	$g_{Landé}$	$|m_J|$ States	Theoretical $g_{\perp}^{eff}$	Theoretical $g_{//}^{eff}$
U(III)	$5f^3$	$^4I_{9/2}$ [B]	0.73	±1/2	3.64	0.73
				±3/2	0	2.18
				**±5/2**	**0**	**3.64**
				±7/2	0	5.07
				±9/2	0	6.55
Ce(III) / U(V)	$4f^1$ / $5f^1$	$^2F_{5/2}$ [B]	0.86	**±1/2**	**2.57**	**0.86**
				±3/2	0	2.57
				±5/2	0	4.29
U(IV)	$5f^2$	$^3H_4$ [C]	0.80	0	0	0
				±1	0	1.60
				±2	0	3.20
				**±3**	**0**	**4.80**
				±4	0	6.40

[A] Ground spectroscopic term using the Russel-Saunders coupling method. [B] Spectroscopic term splits into Kramers Doublets under the crystal field. [C] Spectroscopic term splits into a singlet and pseudo-doublets under axial crystal field. The high, only slightly distorted D_{8h} symmetry of the ligand field around the central metal ion allows to approximate the $|m_J|$ states as pseudo-doublets.

The theoretical $g_{\perp}^{eff}$ and $g_{//}^{eff}$ values in **Table S27** are determined by using an effective $S = ½$ spin Hamiltonian which allows calculation of effective *g*-values for each set of pure $|m_J|$ doublets, which gives under 1st order perturbation theory [28]:

- For $m_j = \pm\frac{1}{2}$:

$$g_{\perp}^{eff} = (J + \frac{1}{2})g_{Landé}$$

$$g_{\parallel}^{eff} = g_{Landé}$$

- For $|m_J| > \frac{1}{2}$:

$$g_{\perp}^{eff} = 0$$

$$g_{\parallel}^{eff} = 2|m_J|g_{Landé}$$

Experimental and simulated EPR spectra

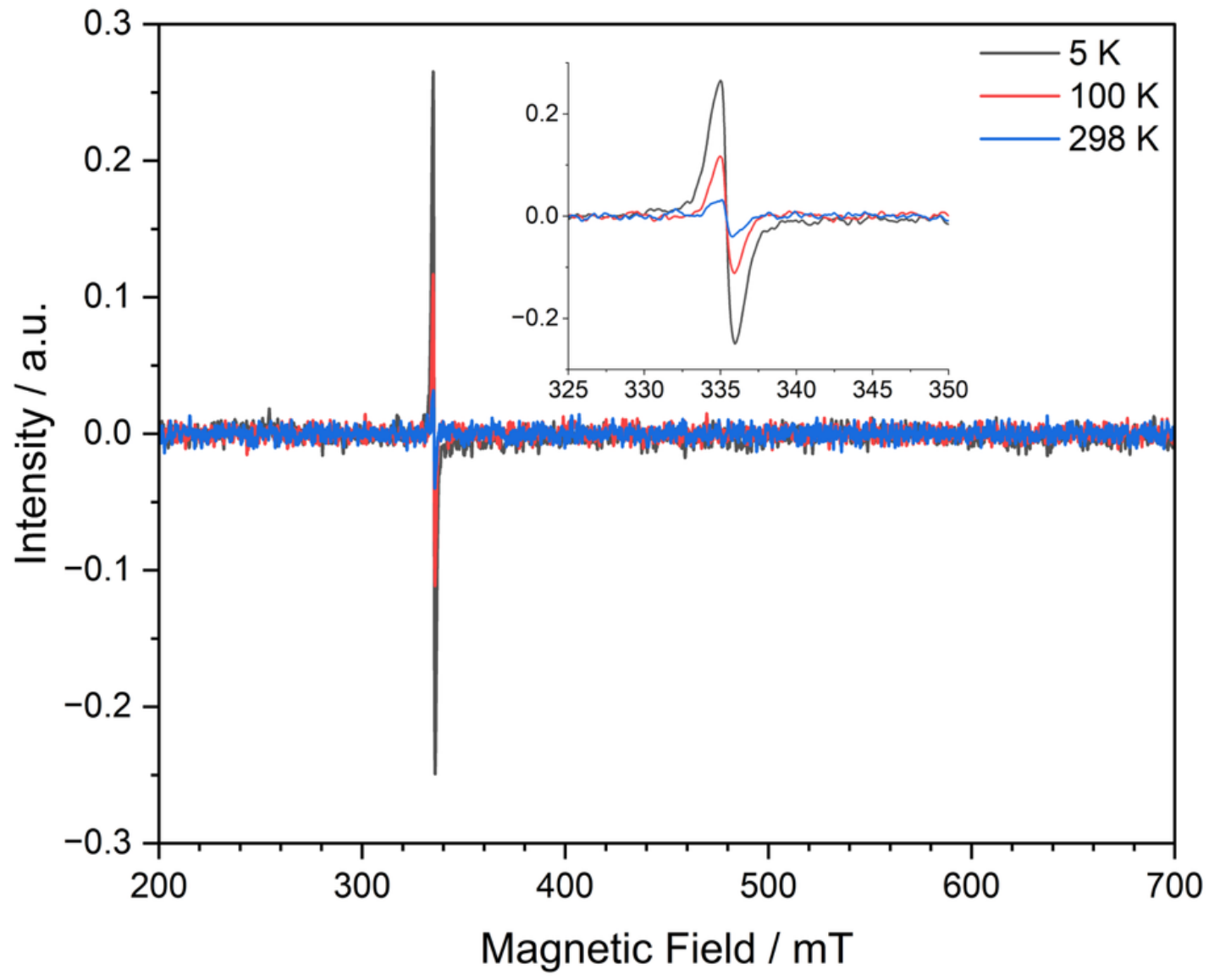

Figure S211. X-band powder EPR spectrum of **2**, U(III), at 5 K (black line), 100 K (red line) and 298 K (blue line). The inset shows a magnified view of the 325–350 mT magnetic field region. The signal at g = 2.0025, observable even at room temperature, is typical of an organic radical signal and is believed to come from traces of “$Li(C_{10}H_8)$” from the synthesis. The signal intensity of this radical is very weak, meaning that it is present in very small quantities. Therefore, **2** was found to be EPR-silent.

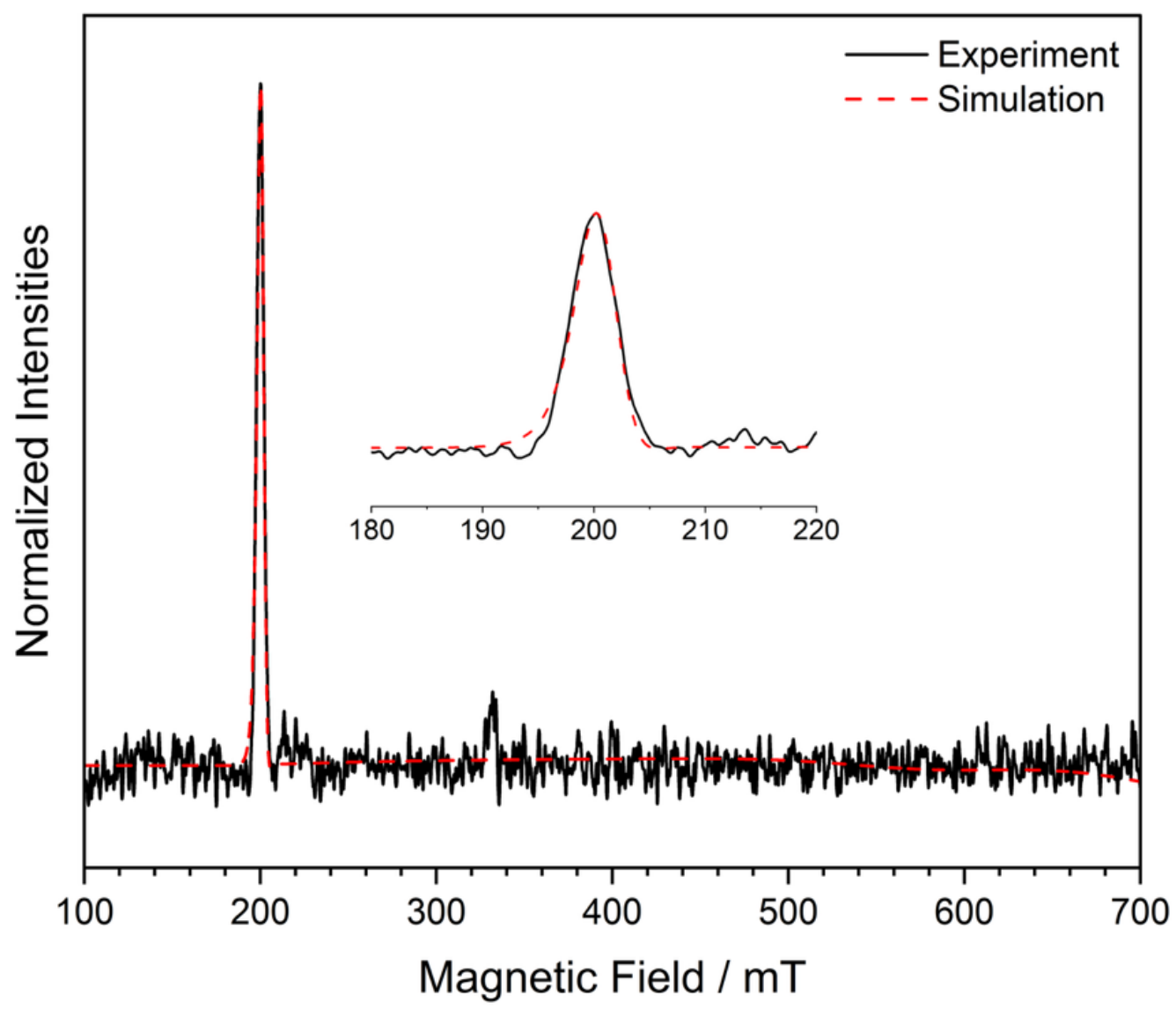

Figure S212. X-band powder EPR spectrum of **5**, U(III), at 5 K (black line) and simulation (red dashed line). Simulation performed with EasySpin as an effective S = ½ species with parameters: $g_\perp$= 0.033, $g_{//}$ = 3.336, g-strain$_\perp$ = 0.007 and g-strain$_{//}$ = 0.057. Arbitrarily small values of $g_\perp$ = 0.033 and g-strain$_\perp$ = 0.007 were used for the simulations as $g_\perp$ must be < 0.4 since we do not experimentally observe a $g_\perp$ feature in the field range of the electromagnet (0 to 1.7 T).

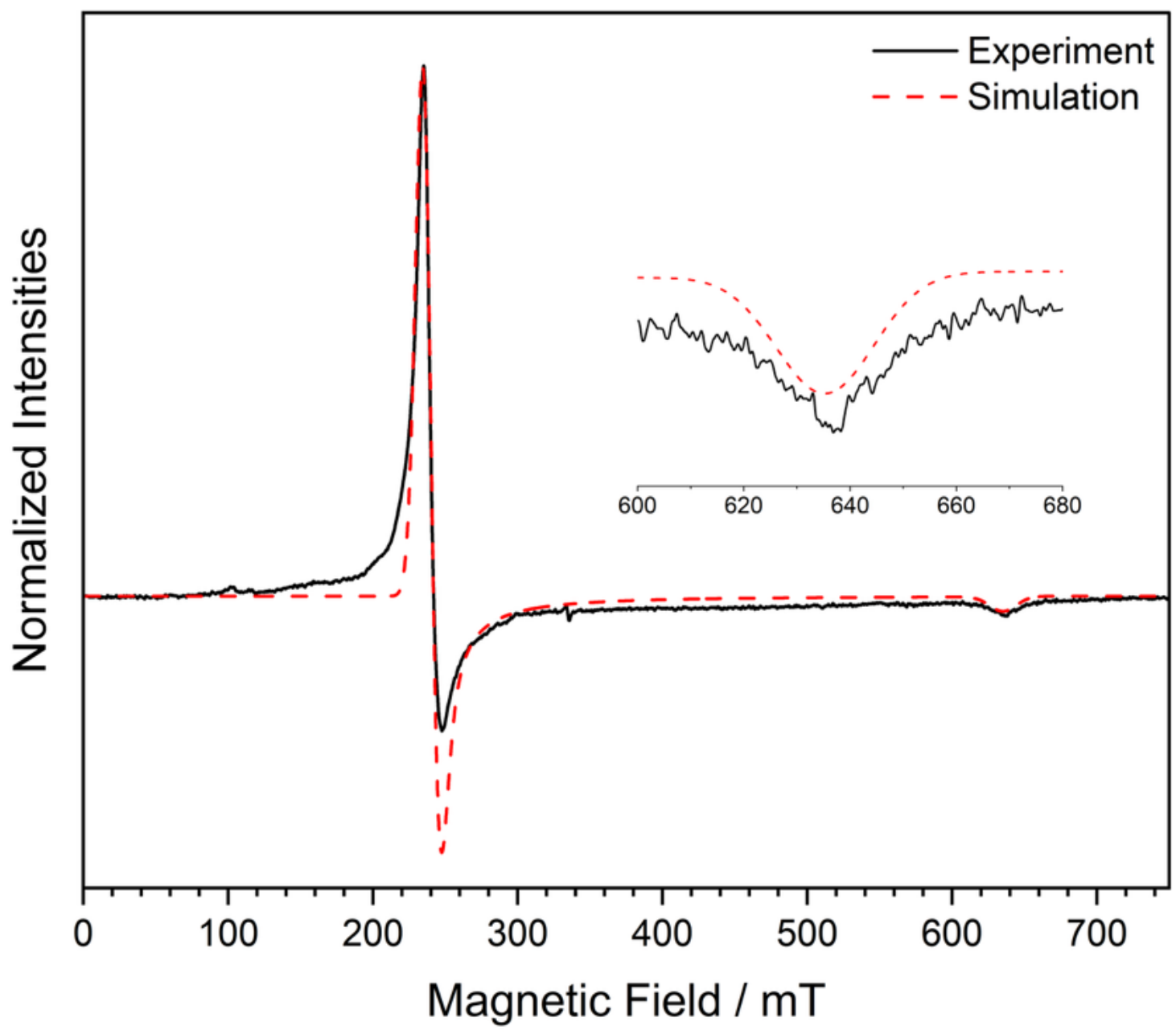

Figure S213. X-band powder EPR spectrum of **3**, U(V), at 5 K (black line) and simulation (red dashed line). Simulation performed with EasySpin as an effective S = ½ species with parameters: $g_{\perp}$= 2.827, $g_{//}$ = 1.057, g-strain$_{\perp}$ = 0.162 and g-strain$_{//}$ = 0.036.

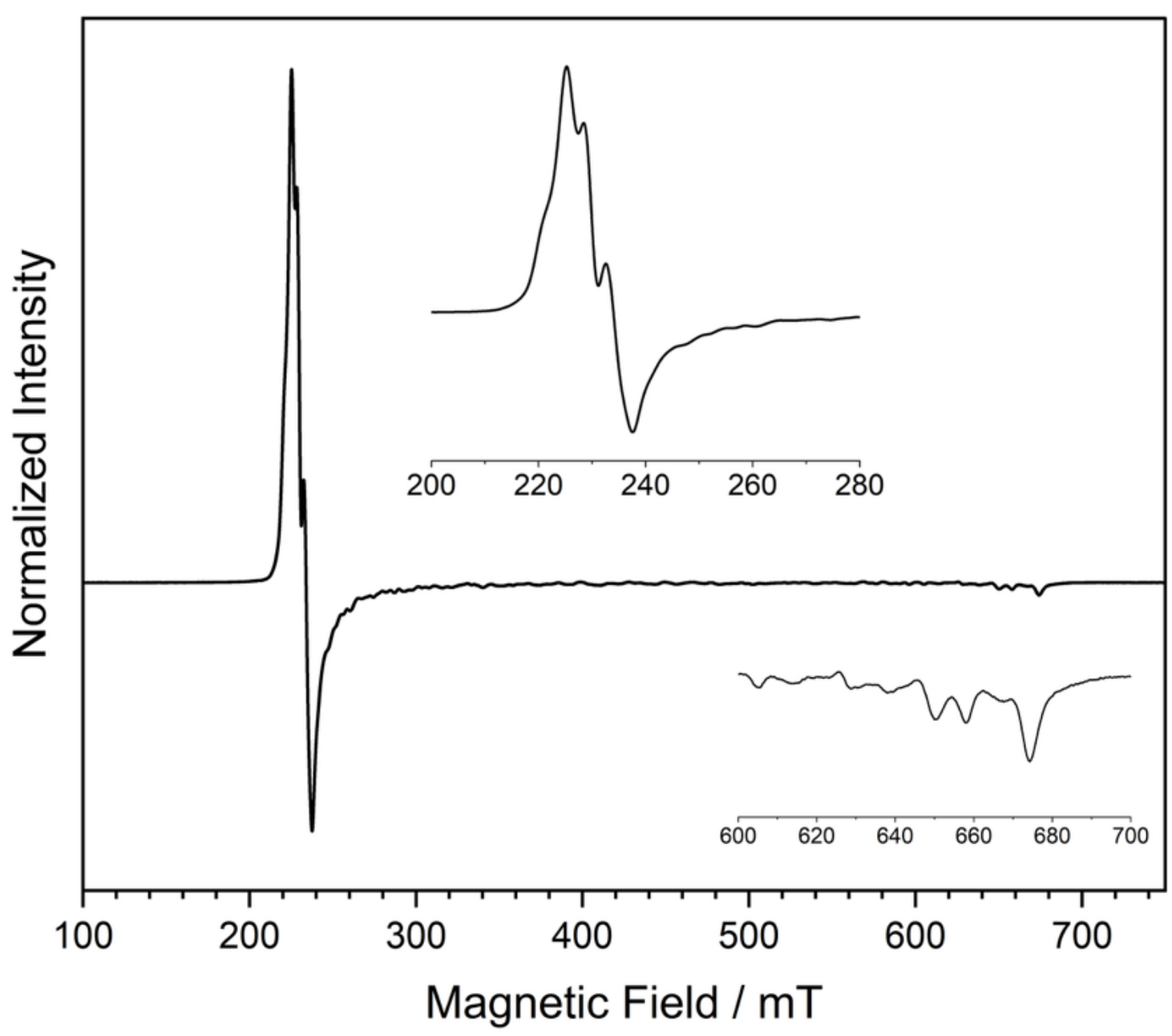

Figure S214. X-band powder EPR spectrum of **6^A^**, U(V), at 5 K. The features observed between *ca.* 200–700 mT are due to polycrystalline effects.

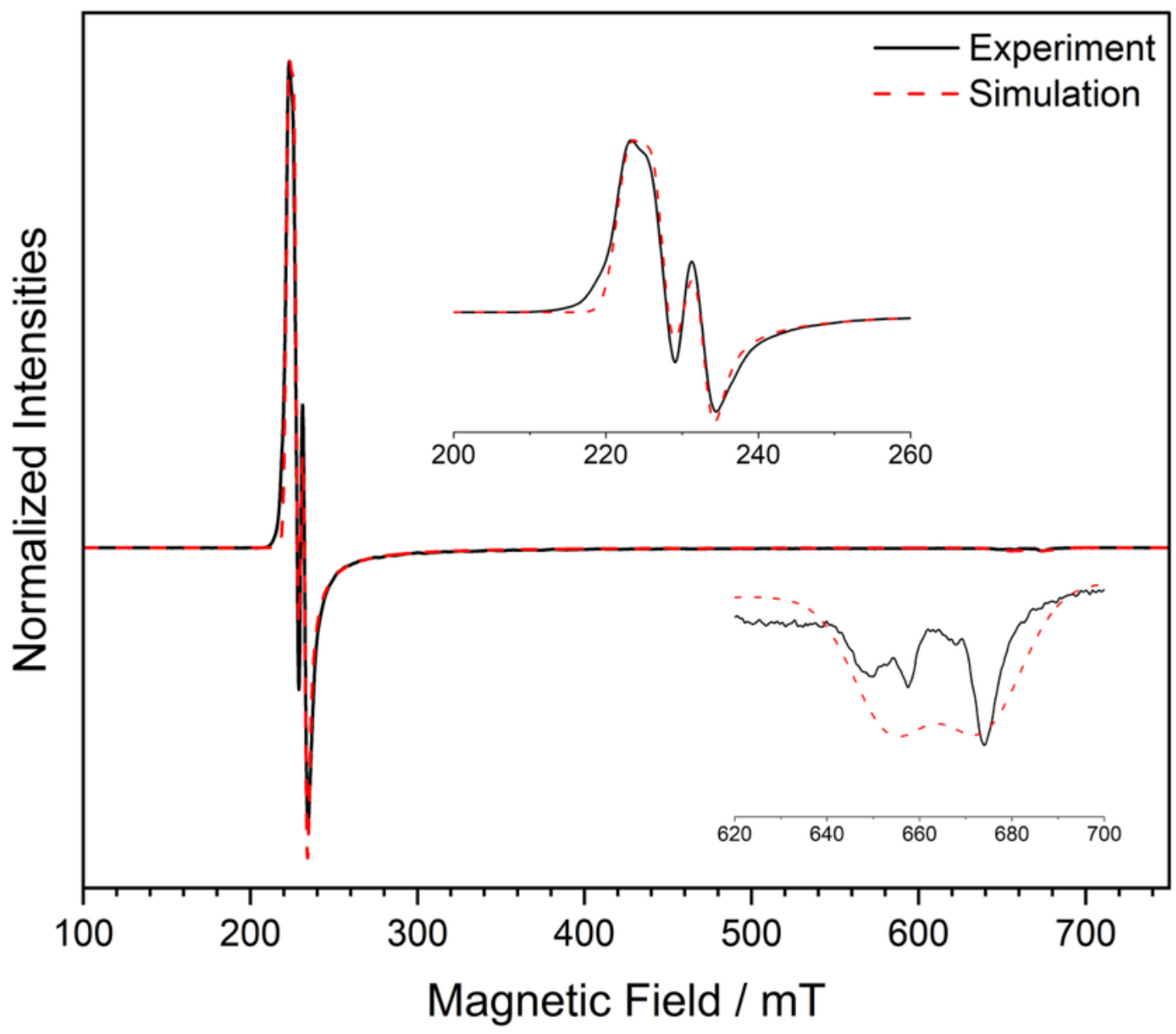

Figure S215. X-band powder EPR spectrum of **6^B**, U(V), at 5 K (black line) and simulation as two U(V) species (red dashed line). Simulation performed with EasySpin as two U(V) $S = {}^1/_2$ species: **$6_{(1)}$** and **$6_{(2)}$**; with parameters: $g_{(1)}$ = [3.014 2.954 0.997], g-strain$_{(1)}$ = [0.050 0.040 0.030], weight$_{(1)}$ = 1.099, $g_{(2)}$ = [2.955 2.887 1.027], g-strain$_{(2)}$ = [0.070 0.035 0.030] and weight$_{(2)}$ = 0.902.

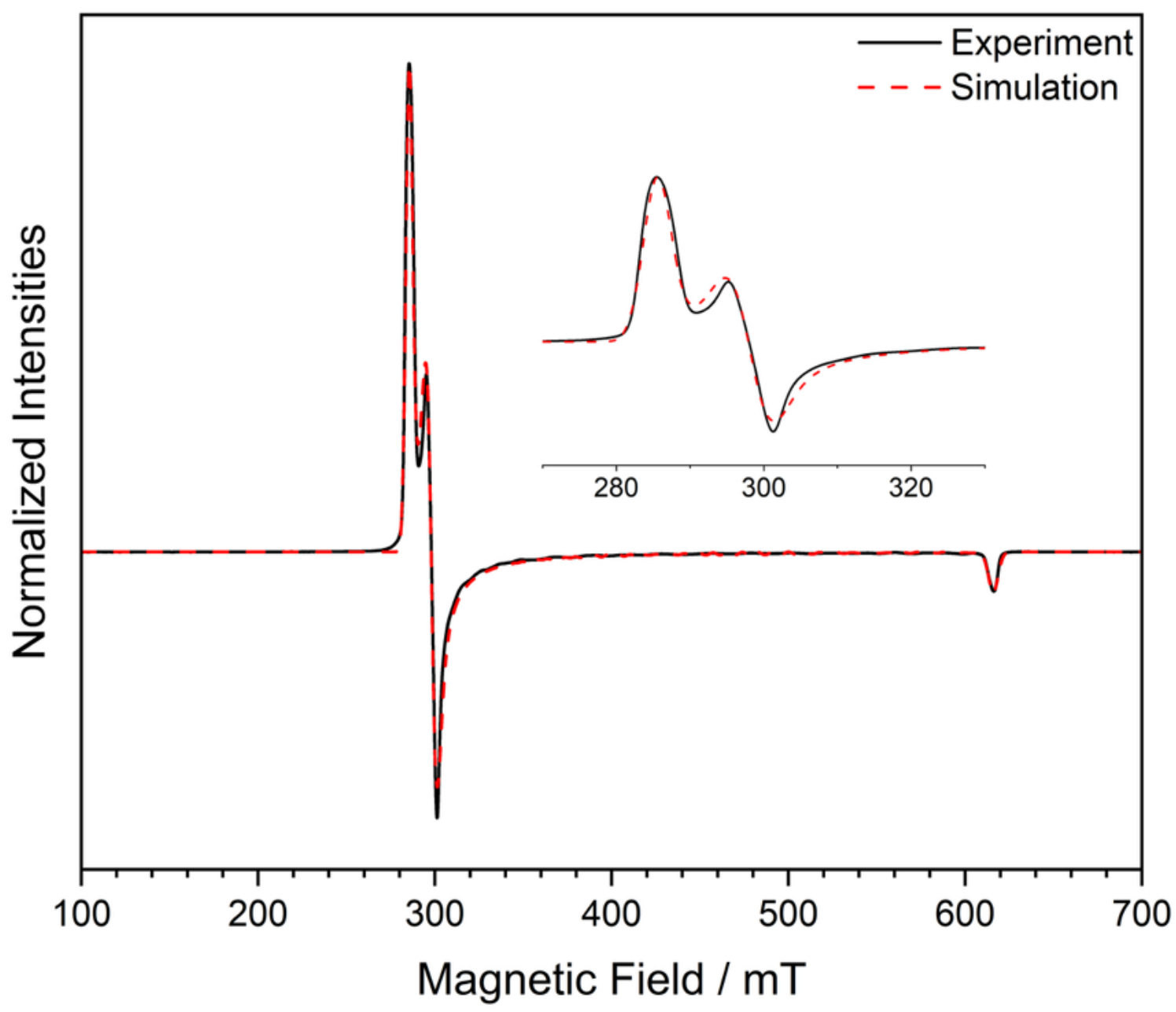

Figure S216. X-band powder EPR spectrum of **10**, Ce(III), at 5 K (black line) and simulation (red dashed line). Simulation performed with EasySpin as an effective S = ½ species with parameters: g = [2.352 2.252 1.090] and g-strain = [0.037 0.046 0.011].

Admixtures between $|J, \pm m_J\rangle$ states accounting for experimental Ce(III) g-values

The *g*-values measured for complex **10** – [Li(TMEDA)$_2$][Ce(III)(COT††)$_2$] – as a powder sample are: g_x = 2.35, g_y = 2.25 and g_z = 1.09 (see **Table S26**).

First, we can assess if a good fit of the experimental g-values is achieved by considering a ground state composed of admixtures of kets belonging to the ground $J = \frac{5}{2}$ multiplet, in a manner analogous to that described by Gourier *et al.* (ref. [29]). This leads to the following ground-state KD wavefunction with coefficients a, b and c controlling the extent of the participation of individual kets to the overall KD wavefunction:

$$|\pm\rangle = \pm a\left|\frac{5}{2}, \pm\frac{1}{2}\right\rangle \pm b\left|\frac{5}{2}, \pm\frac{5}{2}\right\rangle \pm c\left|\frac{5}{2}, \pm\frac{3}{2}\right\rangle$$

Such a ground state KD leads to the following *g*-value expressions[29]:

$$g_x = \pm\frac{6}{7}\left(2\sqrt{5}bc + 4\sqrt{2}ac + 3a^2\right)$$

$$g_y = \pm\frac{6}{7}\left(2\sqrt{5}bc - 4\sqrt{2}ac + 3a^2\right)$$

$$g_z = \pm\frac{6}{7}(5b^2 + a^2 - 3c^2)$$

The normalisation condition imposes:

$$a^2 + b^2 + c^2 = 1$$

Replacement of g_z with 1.09 and of $a^2 = 1 - b^2 - c^2$ (using the normalization condition) into the g_z expression above, gives the following condition on b:

$$b = \sqrt{0.0679167 + c^2}$$

Replacement of $a^2 = 1 - b^2 - c^2$and $b = \sqrt{0.0679167 + c^2}$ into the g_x expression gives:

$$g_x = \frac{6}{7}\left[2\sqrt{5}\sqrt{0.0679167 + c^2}c + 4\sqrt{2}\sqrt{1 - 0.0679167 - 2c^2}c + 3(1 - 0.0679167 - 2c^2)\right]$$

Eq. S5.

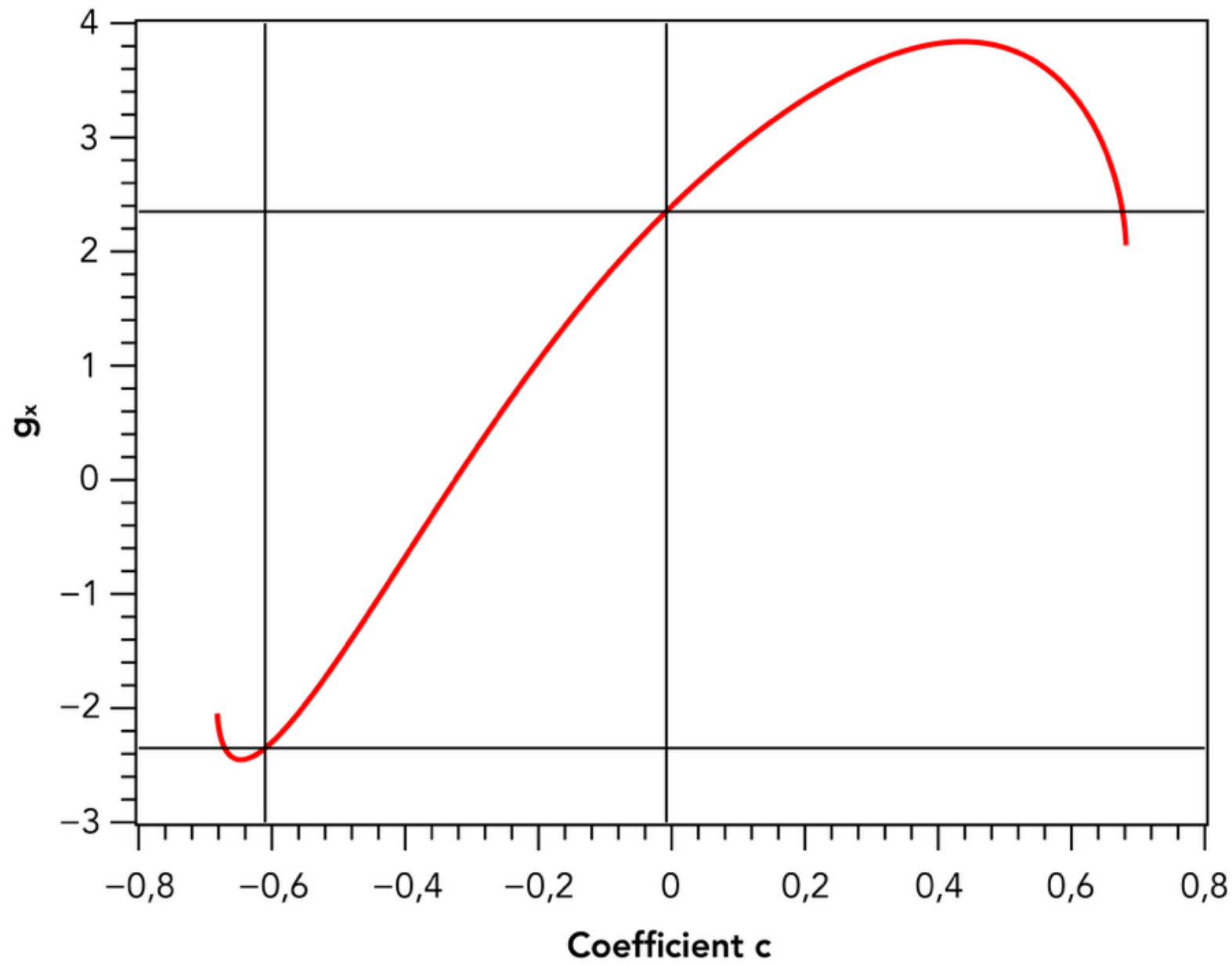

Figure S217. g_x as a function of coefficient c as described in **Eq. S5** Horizontal lines indicate $g_x = \pm 2.35$. Vertical lines indicate the values of c where $g_x = \pm 2.35$.

As shown in **Figure S217**, for $g_x = -2.35$:

$$c = -0.61$$

$$b = 0.6633$$

$$a = 0.4335$$

And subsequently, $g_y = 0.214$ which is very far from the experimental $g_y = 2.25$. The other solution is given for $g_x = +2.35$ (**Figure S217**):

$$c = -0.008$$

$$b = 0.26073$$

$$a = 0.96538$$

Which gives $g_y = 2.426$ which is still relatively far from $g_y = 2.25$. Therefore, this model, which considers admixtures only within the ground $J = \frac{5}{2}$ multiplet, doesn't give the best agreement with the experimental *g*-values.

Second, if we now consider a ground state composed of admixtures between $|J = \frac{5}{2}, m_J = \pm\frac{1}{2}\rangle$ and $|J = \frac{7}{2}, m_J = \pm\frac{1}{2}\rangle$ kets, in a manner analogous to that of Solis-Céspedes et al. (ref. [25]). This leads to the following ground-state KD wavefunction:

$$|\Psi\rangle = A|\frac{5}{2}, \pm\frac{1}{2}\rangle - B|\frac{7}{2}, \pm\frac{1}{2}\rangle$$

With *g*-values given by: $g_{x,y}$ and g_z [25]:

$$g_{x,y} = g_{\perp} = -\frac{18}{7}A^2 + \frac{32}{7}B^2 + \frac{4\sqrt{3}}{7}AB$$

$$g_z = g_{\parallel} = \frac{6}{7}A^2 + \frac{8}{7}B^2 + \frac{8\sqrt{3}}{7}AB$$

If we replace $A = \sqrt{1 - B^2}$ (normalization condition) into $g_{\parallel}$ we get:

$$g_{\parallel} = \frac{6}{7}(1 - B^2) + \frac{8}{7}B^2 + \frac{8\sqrt{3}}{7}\sqrt{1 - B^2}B$$

Eq. S6.

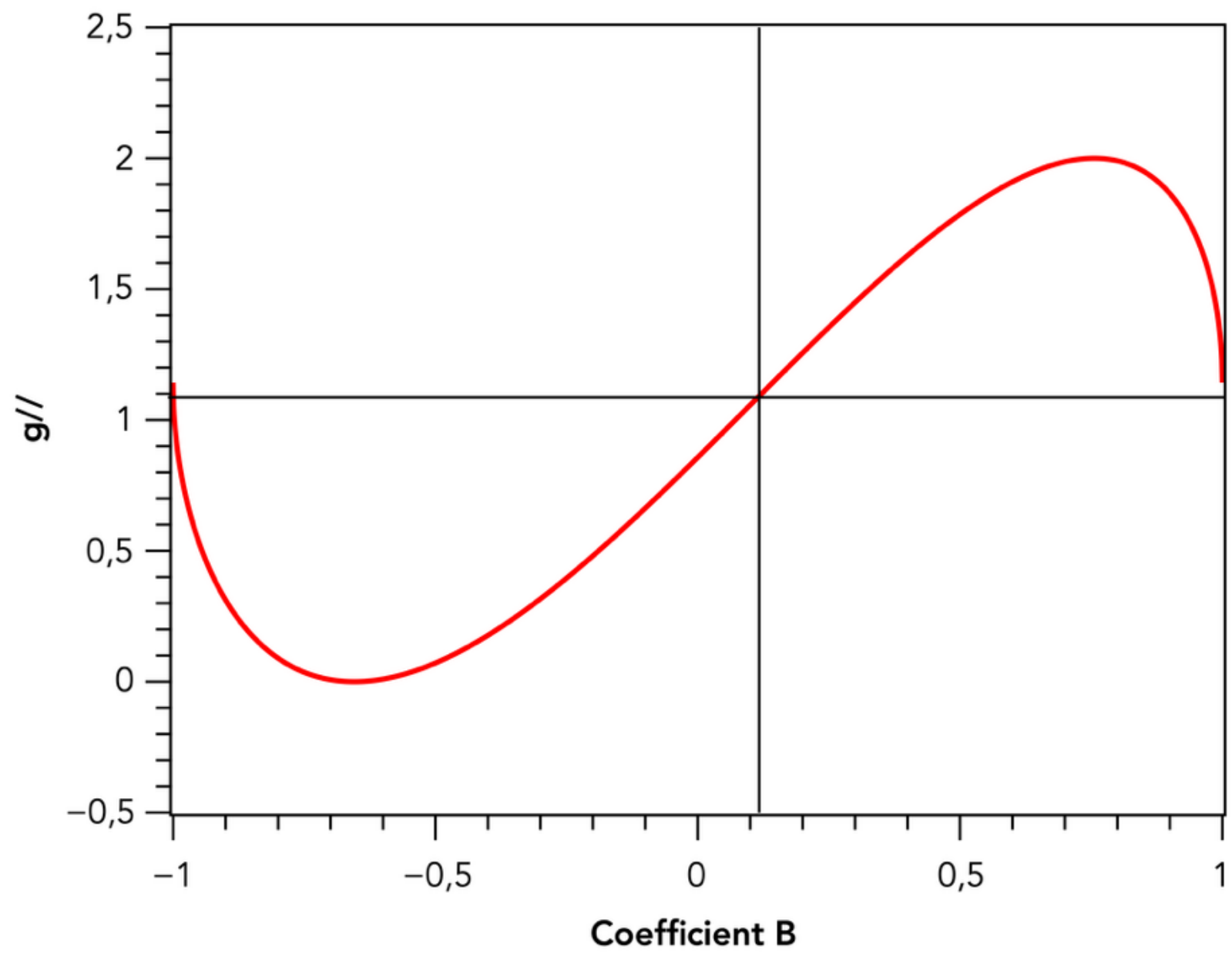

Figure S218. $g_{\parallel}$as a function of coefficient B as described in **Eq. S6**. The horizontal line indicates $g_{\parallel}$=1.09 and the vertical line, the corresponding value of B where $g_{\parallel} = 1.09$.

As shown in **Figure S218**, for $g_{\parallel} = 1.09$:

$$A = 0.984$$

$$B = 0.177$$

And, subsequently: $|g_{\perp}| = 2.358$ which fits well the experimental values: $g_x = 2.35$ and $g_y = 2.25$.

Therefore, a ground state composed of $98.6\%|J = \frac{5}{2}, m_J = \pm\frac{1}{2}\rangle$ and $3.1\%|J = \frac{7}{2}, m_J = \pm\frac{1}{2}\rangle$ can be considered a suitable *5f* composition of the SOMO of Ce(III) in the [Li(TMEDA)$_2$[Ce^{III}(COT††)$_2$] complex.

S9. Computational methodology – DFT

General considerations

Unrestricted Kohn-Sham density functional theory (DFT) calculations were performed at the scalar-relativistic (SR) DFT level on **1**–**6**, **9**, **10**, [$Ce^{IV}(COT)_2$], and [$Ce^{III}(COT)_2$]$^{-}$. Where appropriate, only the anion or cation was considered due to the separated nature of the anion and cation parts of the relevant complexes. Optimized coordinate sets were calculated in the gas phase in the following ways: (i) only H-atom positions were optimized, all others were held at the coordinates determined by SCXRD; (ii) all atom positions were optimized; (iii) symmetry constraints were applied and all atoms were optimized, but only for **1** (D_{8h}, **1-D8h**), **2** (C_{2v}, **2-C2v**), and **3** (C_{2v}, **3-C2v**; D_{8d}, **3-D8d**). Except where stated otherwise, all properties (MO and NLMO compositions, QTAIM properties, EDA-NOCV results, and others) are given from the H-atom optimized coordinates. The differences in orbital composition between the H-atom optimized data and the fully optimized coordinates were negligible. Despite these small differences in their electronic structures, the shallow potential energy surface for $COT_{centroid}\cdots M\cdots COT_{centroid}$ bending and rotation in the anion and cation complexes led to fully optimized coordinate sets that differed from those of the crystal structures, yet still represented local minima by vibrational harmonic analysis (except with **2-Opt** where a single small, *ca.* –5 cm^{-1} imaginary mode could not be removed through multiple iterations with extremely fine grid settings and convergence criteria). Optimizations performed using a solvent model or larger basis sets on all atoms did not converge to solutions that better represented the solid-state structures, but required increased computational costs for all subsequent calculations, and so a gas-phase model using H-atom-optimized coordinates was used.

All optimizations were performed using the multiplicity which best corresponds to their experimentally determined ground states (**1**, **4**, $S = 1$; **2**, **5**, $S = {}^{3}/_{2}$; **3**, **6**, **10**, [$Ce^{III}(COT)_2$]$^{-}$ $S = {}^{1}/_{2}$; **9**, [$Ce^{IV}(COT)_2$] $S = 0$). These calculations were performed with the ORCA 6.1.0 quantum chemistry program suite[30]. The PBE0[31,32] hybrid functional was used with the D3-BJ dispersion correction[33], a tight integration grid (DefGrid3), and the resolution of the identity 'chain of spheres' (RIJCOSX) approximation[34,35]. Scalar relativistic effects were accounted for using the exact two-component (X2C) Hamiltonian, along with the diagonal local approximation to the unitary transformation matrix (DLU-X2C) to decrease computational cost[36]. Segmented contracted all-electron error-consistent basis sets of triple-ζ valence quality were used for all atoms as follows: H, C, Si (X2C-SVPall for optimizations, X2C-TZVPall for properties)[37,38], Ce (X2C-TZVPPall for optimizations, X2C-QZVPall for properties)[37,38] along with the appropriate X2C/J auxiliary basis

sets[37,38]. For U, the cc-pVDZ-X2C basis set was used throughout[39-41], with automatic generation of auxiliary basis sets[42]. Picture-change effects were accounted for, and the full relativistic Hamiltonian derivative was calculated (PictureChange 2). Where appropriate, the optimized structures were confirmed to be true minima by analytical vibrational frequency calculations. Molecular orbital compositions were examined for **1** and **3** using additional density functionals which spanned GGA, meta-GGA, hybrid-GGA, and hybrid meta-GGA classes (see below for further details).

Further gas-phase single-point calculations were performed in ADF2025.102 (ref.[43]) to perform energy decomposition (EDA) and extended transition state natural orbitals for chemical valence (ETS-NOCV) analyses for the M + COT interactions as $\{M(COT^{R})\}^{n+}$ + $\{(COT^{R})\}$ (refs.[44-47]). These calculations employed the PBE0 functional, with D3-BJ dispersion correction[33,48] and a Slater-type doubly-polarized triple-ζ basis set (TZP) on U and Ce atoms, and a triple-ζ basis set (TZP) on H, C, Si atoms – all electrons were explicitly treated[49]. The ZORA (Zeroth Order Regular Approximation) scalar relativistic Hamiltonian was used throughout[50,51]. The VeryGood BeckeGrid was employed with a RadialGridBoost (level 3) applied to avoid linear dependencies.

Electron density-based analysis of metal–ligand bonding utilized the AIMAll software package[52]. using WFX files generated from the DFT calculations (in ORCA 6.1.0 using the orca_2aim module). The default settings were used to complete the electron density topology and integration of atomic basins. The NBO (7.0) software package was used to compute NLMOs to analyse bonding orbitals[53]. Chemcraft was used for the visualization of molecular orbitals from DFT calculations, and the generation of starting point symmetrized coordinates for geometry optimizations[54].

In the following sections, MO isosurfaces are all given at the same isovalue (0.05 e Å^{-3}). This makes direct comparison between the unsubstituted (**1**–**3**) and substituted (**4**–**6**) complexes challenging due to the diffuse nature of Kohn-Sham MOs. As such, we also present tables giving the composition of relevant MOs to provide a clearer picture of the metal *vs* ligand involvement.

*Metal spin and charge analysis for **1**–**6***

Table S28. Charge analyses for the central metal atoms in H-atom optimized **1**–**6** using the QTAIM, natural charge, Löwdin, and Hirshfeld formalisms. Shown in order of metal oxidation state.

Complex	QTAIM	NPA	Löwdin	Hirshfeld
**2** U(III) 5f^3	1.86	0.926	0.119	0.183
**5** U(III) 5f^3	1.88	0.943	0.066	0.180
**10** Ce(III) 4f^1	1.83	0.891	–1.209	0.453
**1** U(IV) 5f^2	2.06	1.011	0.325	0.389
**4** U(IV) 5f^2	2.09	1.004	0.216	0.334
**9** Ce(IV) 4f^0	1.91	0.991	–1.221	0.544
**3** U(V) 5f^1	2.15	0.999	0.444	0.503
**6** U(V) 5f^1	2.14	1.081	0.320	0.422

Table S29. Spin density analyses for the central metal atoms in H-atom optimized **1**–**6** using the QTAIM, natural spin, Löwdin, and Hirshfeld formalisms. Shown in order of metal oxidation state.

Complex	Formal	QTAIM	NSD	Löwdin	Hirshfeld
**2** U(III) 5f^3	3	2.91	3.018	2.988	2.889
**5** U(III) 5f^3	3	2.91	3.008	2.994	2.888
**10** Ce(III) 4f^1	1	1.02	1.025	1.037	1.015
**1** U(IV) 5f^2	2	2.16	2.226	2.197	2.126
**4** U(IV) 5f^2	2	2.15	2.216	0.192	2.120
**9** Ce(IV) 4f^0	0	–	–	–	–
**3** U(V) 5f^1	1	1.38	1.393	1.370	1.339
**6** U(V) 5f^1	1	1.54	1.553	1.533	1.501

Frontier molecular orbital compositions and visualizations and compositions

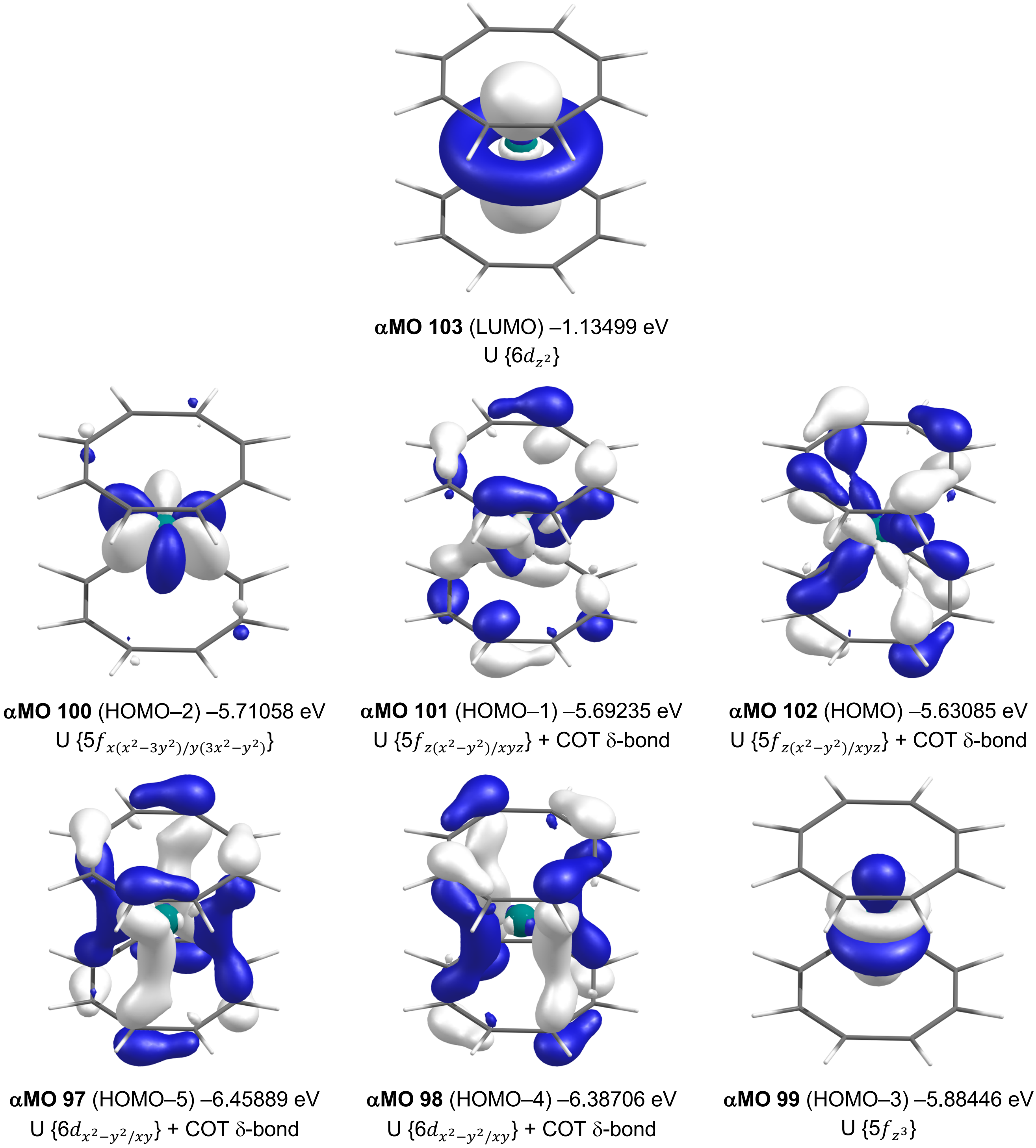

Figure S219. Selected MOs and their compositions for **1**. Isosurface values 0.05 e Å^{-3}.

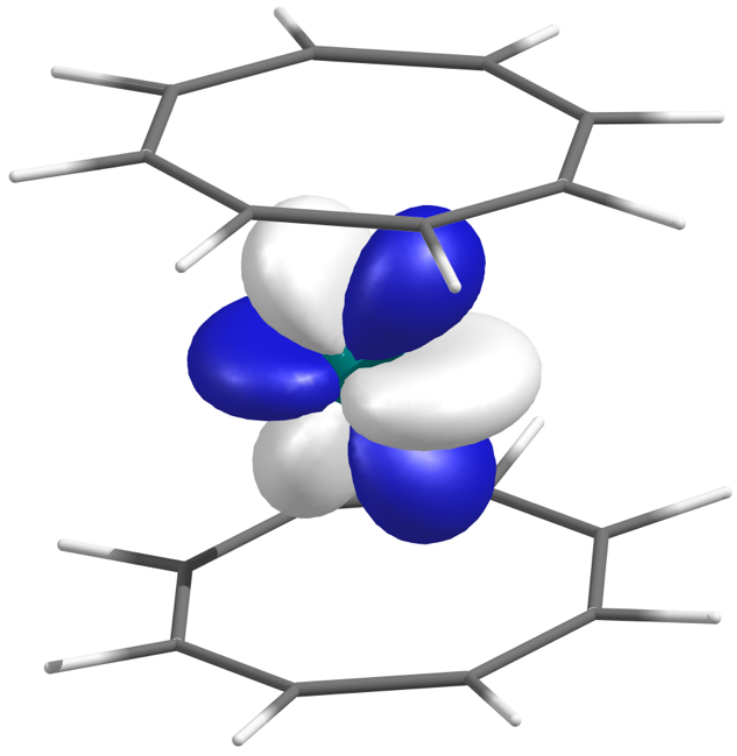

αMO 103 (HOMO) 0.13061 eV
U {$5f_{yz^2/xz^2}$}

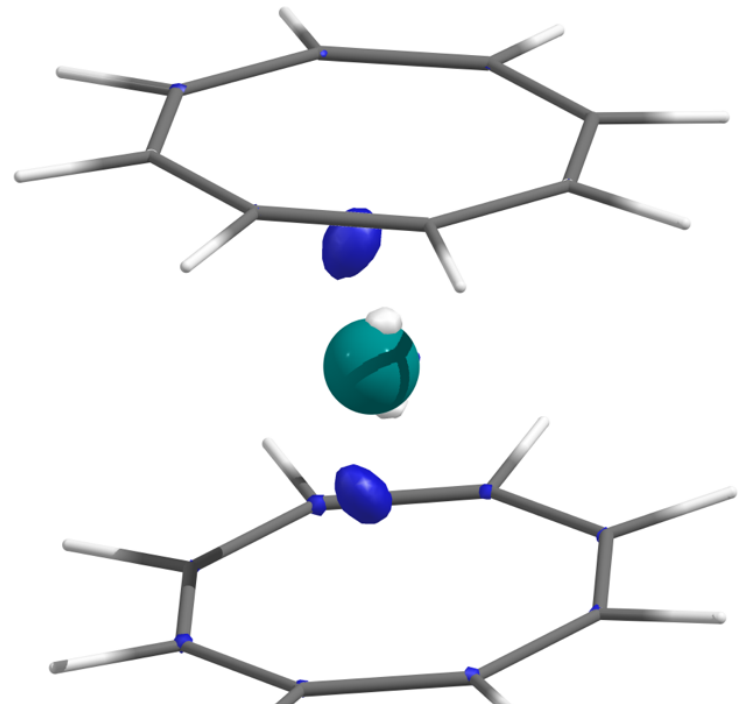

αMO 104 (LUMO) 2.75842 eV
U {$7s$ + $6d_{z^2}$}

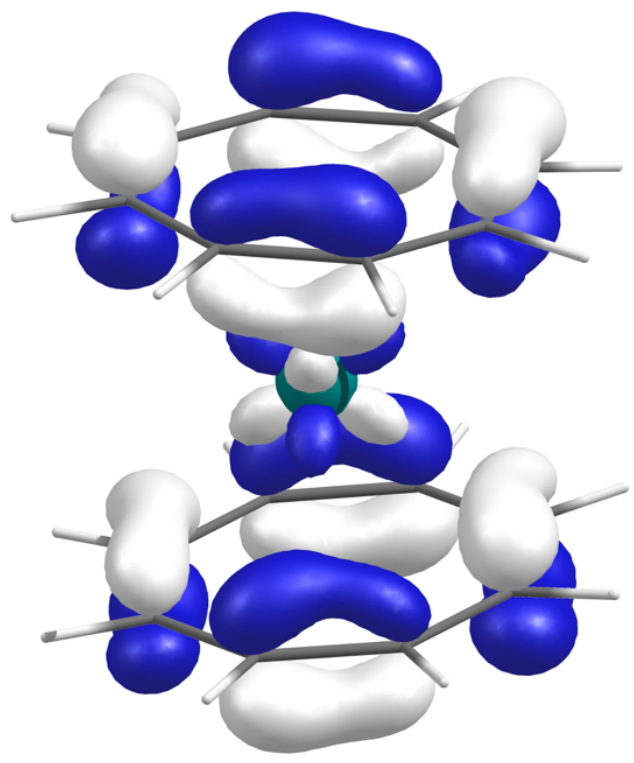

αMO 100 (HOMO–3) –1.32138 eV
U {$5f_{z(x^2-y^2)/xyz}$} + COT δ-bond

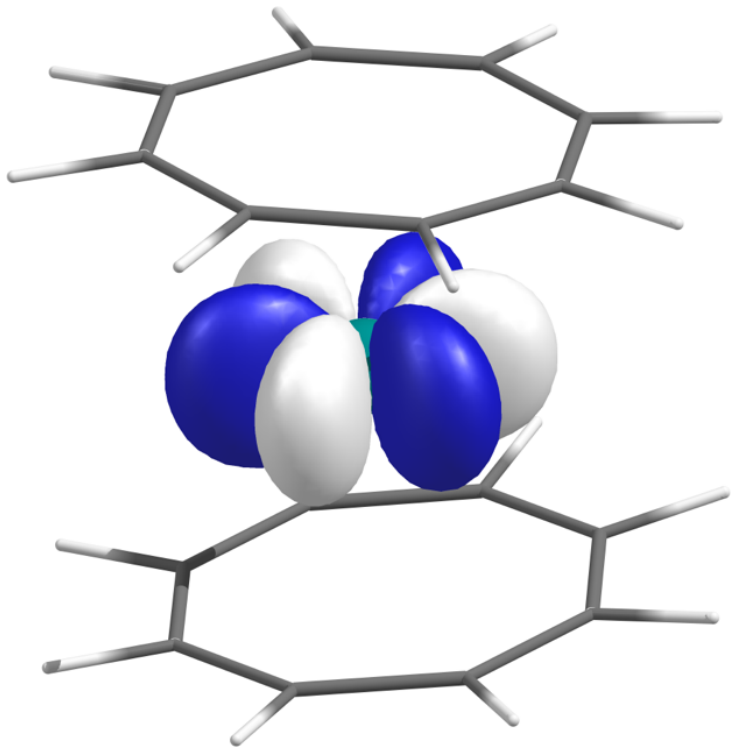

αMO 101 (HOMO–2) –0.06123 eV
$5f_{x(x^2-3y^2)/y(3x^2-y^2)}$

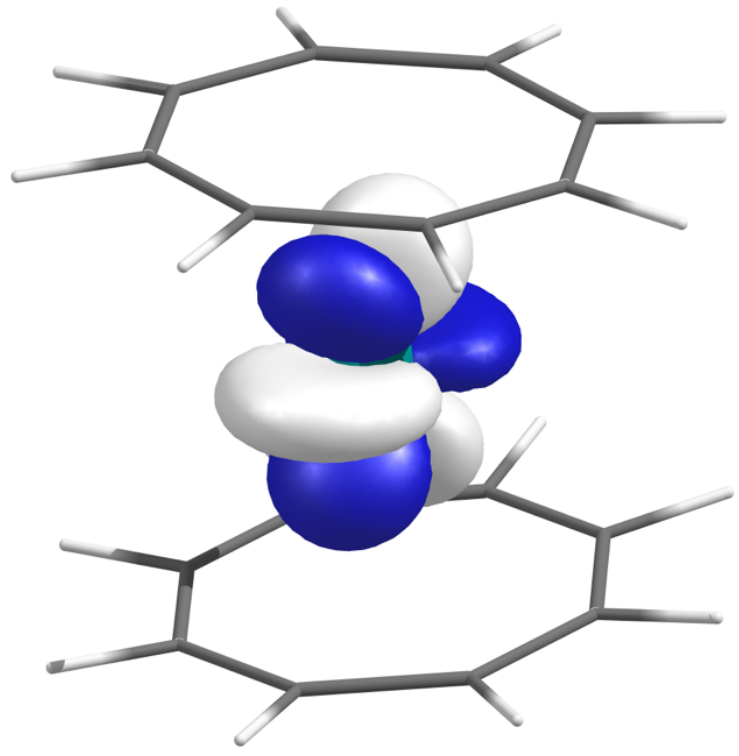

αMO 102 (HOMO–1) 0.12925 eV
U {$5f_{yz^2/xz^2}$}

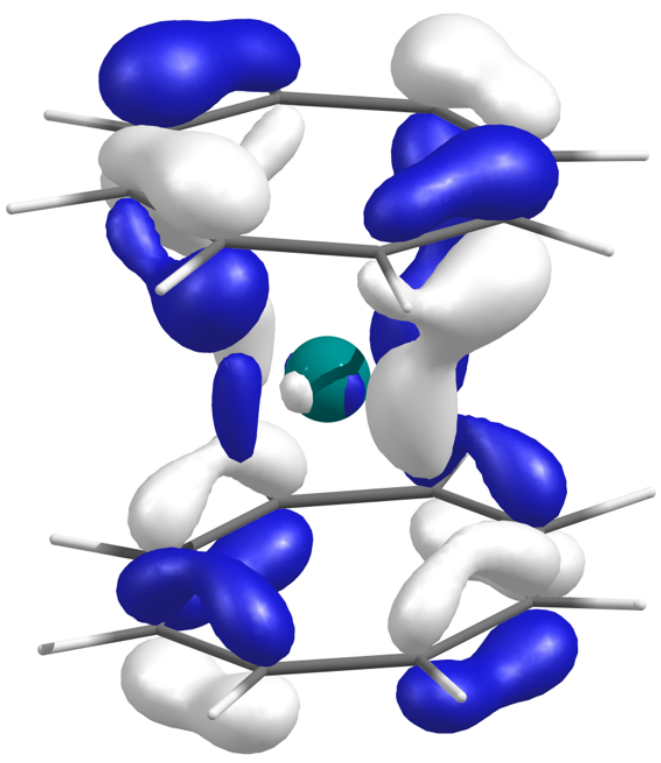

αMO 97 (HOMO–6) –2.24793 eV
U {$6d_{x^2-y^2/xy}$} + COT δ-bond

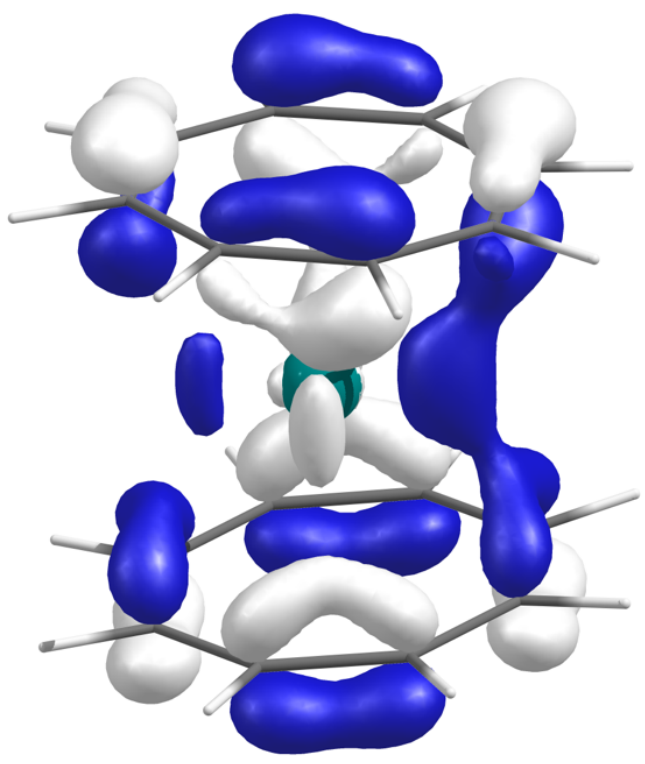

αMO 98 (HOMO–5) –2.20929 eV
U {$6d_{x^2-y^2/xy}$} + COT δ-bond

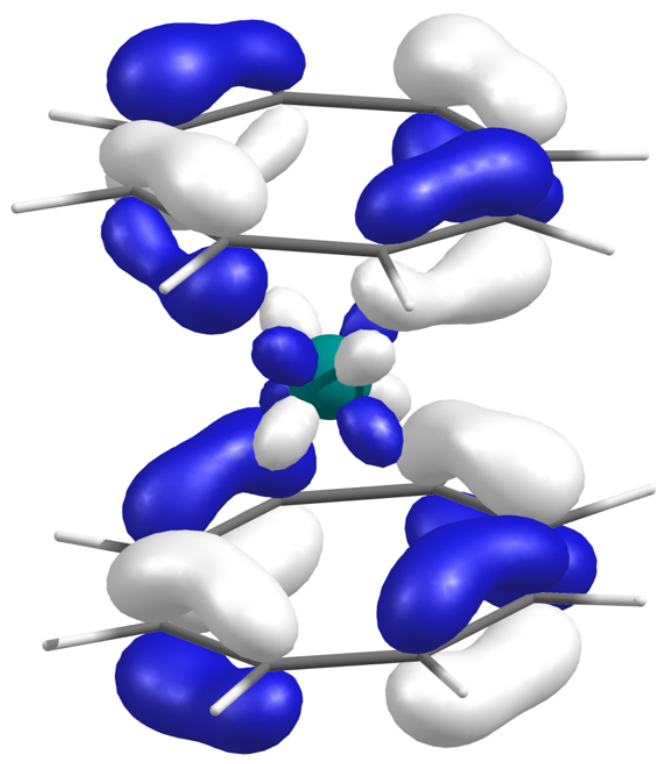

αMO 99 (HOMO–4) –1.36492 eV
U {$5f_{z(x^2-y^2)/xyz}$} + COT δ-bond

Figure S220. Selected MOs and their compositions for **2**. Isosurface values 0.05 e Å^{-3}.

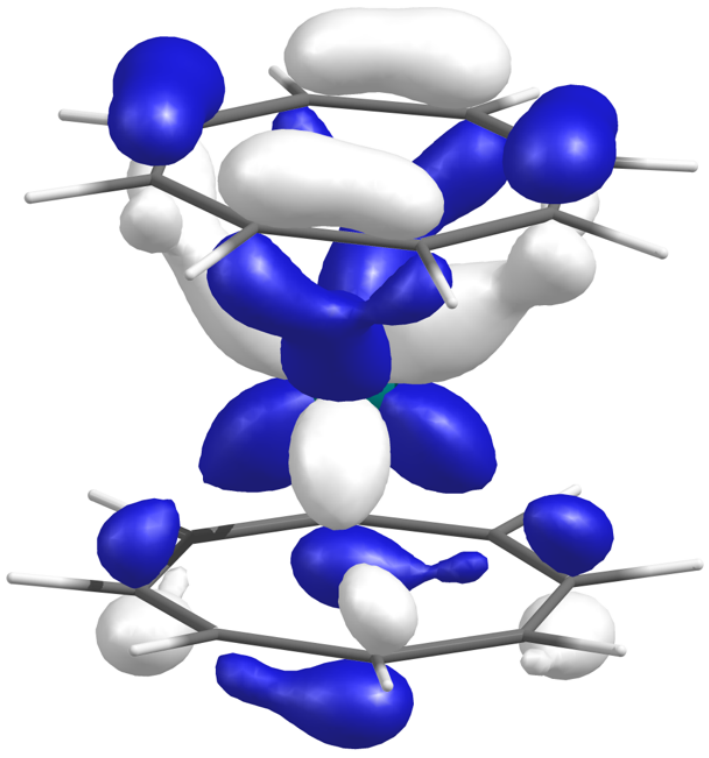

αMO 101 (HOMO) –10.21461 eV
U $\{5f_{z(x^2-y^2)/xyz}\}$ + COT δ-bond

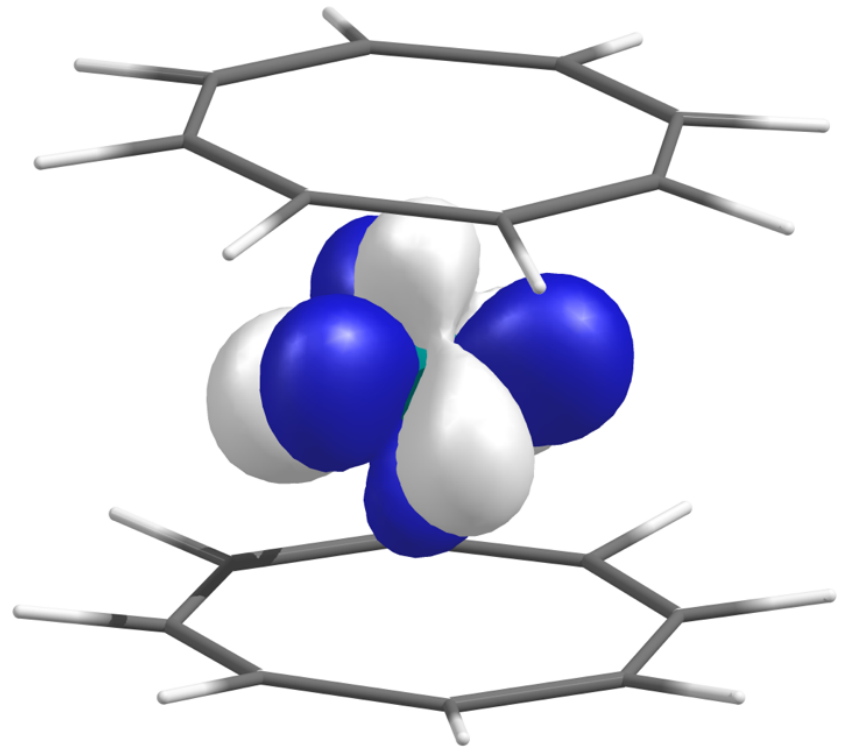

αMO 102 (LUMO) –6.67250 eV
U $\{5f_{x(x^2-3y^2)/y(3x^2-y^2)/z^3}\}$

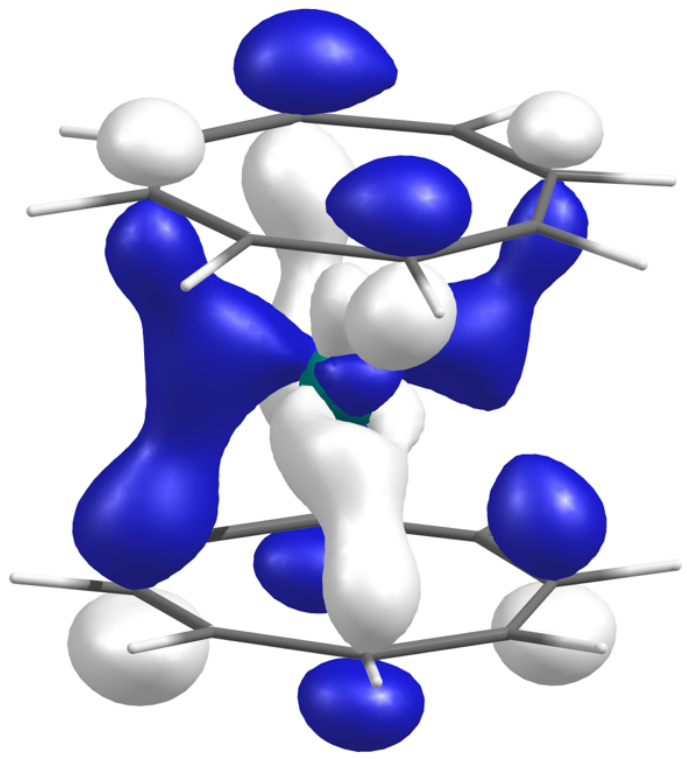

αMO 99 (HOMO–2) –10.64564 eV
U $\{6d_{x^2-y^2/xy}$ + $5f_{z^3}\}$ + COT δ-bond

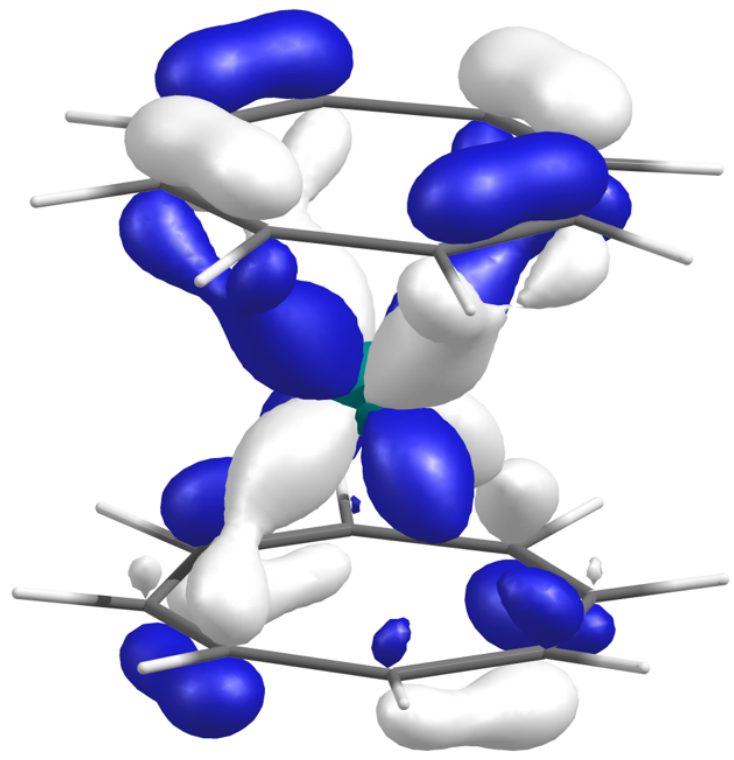

αMO 100 (HOMO–1) –10.23692 eV
U $\{5f_{z(x^2-y^2)/xyz}\}$ + COT δ-bond

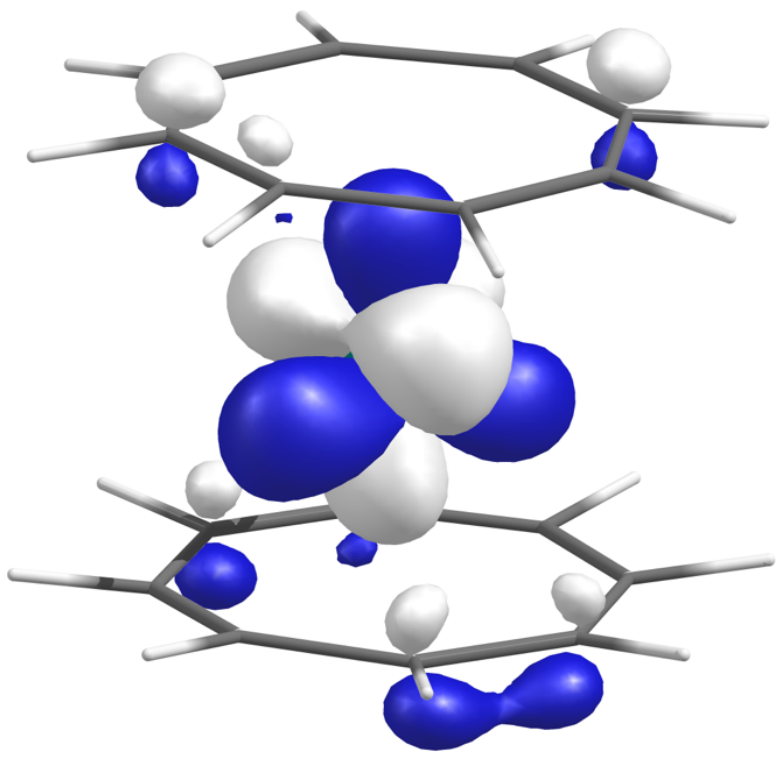

αMO 97 (HOMO–4) –10.72292 eV
U $\{5f_{x(x^2-3y^2)/y(3x^2-y^2)/z^3}\}$

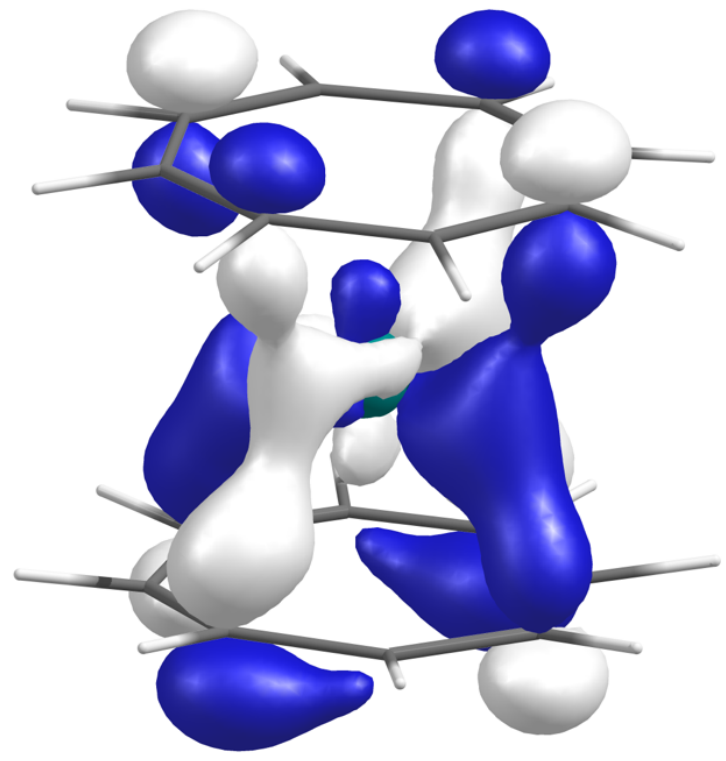

αMO 98 (HOMO–3) –10.67884 eV
U $\{6d_{x^2-y^2/xy}$ + $5f_{z^3}\}$ + COT δ-bond

Figure S221. Selected MOs and their compositions for **3**. Isosurface values 0.05 e Å^{-3}.

Table S30. Löwdin orbital populations for selected orbitals and interactions in **1**.

**Description**	**U 5*d*–COT δ-bond**		**U 5*f*–COT δ-bond**		**5*f* localized**	
**MO#**	**97** HOMO–5	**98** HOMO–4	**101** HOMO–1	**102** HOMO	**99** HOMO–3	**100** HOMO–2
**Energy (eV)**	−6.45889	−6.38706	−5.69235	−5.63085	−5.88446	−5.71058
M *s* (%)	0.0	0.0	0.0	0.0	0.0	0.0
M *p* (%)	0.0	0.0	0.0	0.0	0.4	0.0
M *d* (%)	21.2	21.3	0.0	0.0	0.0	0.0
M *f* (%)	0.0	0.0	33.7	28.1	91.3	88.4
**Total M (%)**	21.2	21.3	33.7	28.1	91.7	88.4

Table S31. Löwdin orbital populations for selected orbitals and interactions in **2**.

**Description**	**U 5*d*–COT δ-bond**		**U 5*f*–COT δ-bond**		**5*f* localized**		
**MO#**	**97** HOMO–6	**98** HOMO–5	**99** HOMO–4	**100** HOMO–3	**101** HOMO–2	**102** HOMO–1	**103** HOMO
**Energy (eV)**	−2.24793	−2.20929	−1.36492	−1.32138	−0.06123	0.12925	0.13061
M *s* (%)	0.0	0.0	0.0	0.0	0.0	0.0	0.0
M *p* (%)	0.0	0.0	0.0	0.0	0.0	1.0	0.9
M *d* (%)	19.5	19.6	0.0	0.0	0.1	0.1	0.1
M *f* (%)	0.1	0.2	14.4	14.5	93.0	92.7	92.8
**Total M (%)**	19.6	19.8	14.4	14.5	93.1	93.8	93.8

Table S32. Löwdin orbital populations for selected orbitals and interactions in **3**.

**Description**	**U 5*d*–COT δ-bond**		**U 5*f*–COT δ-bond**		**5*f* localized**
**MO#**	**98** HOMO–3	**99** HOMO–2	**100** HOMO–1	**101** HOMO	**97** HOMO–4
**Energy (eV)**	−10.67884	−10.64564	−10.23692	−10.21461	−10.72292
M *s* (%)	0.0	0.0	0.0	0.0	0.0
M *p* (%)	0.0	0.1	0.0	0.0	0.3
M *d* (%)	20.3	19.2	0.2	0.3	5.0
M *f* (%)	7.7	13.5	42.8	42.4	69.9
**Total M (%)**	28.0	32.8	43.0	42.7	75.2

*Symmetrized complexes **1–3***

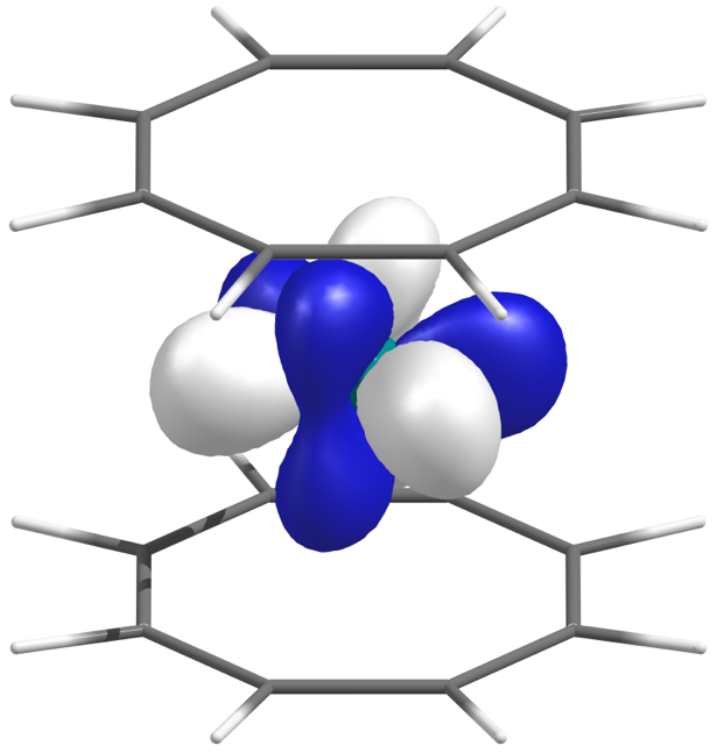

αMO 103 (LUMO) –1.51214 eV
U {6d_{z^2}}

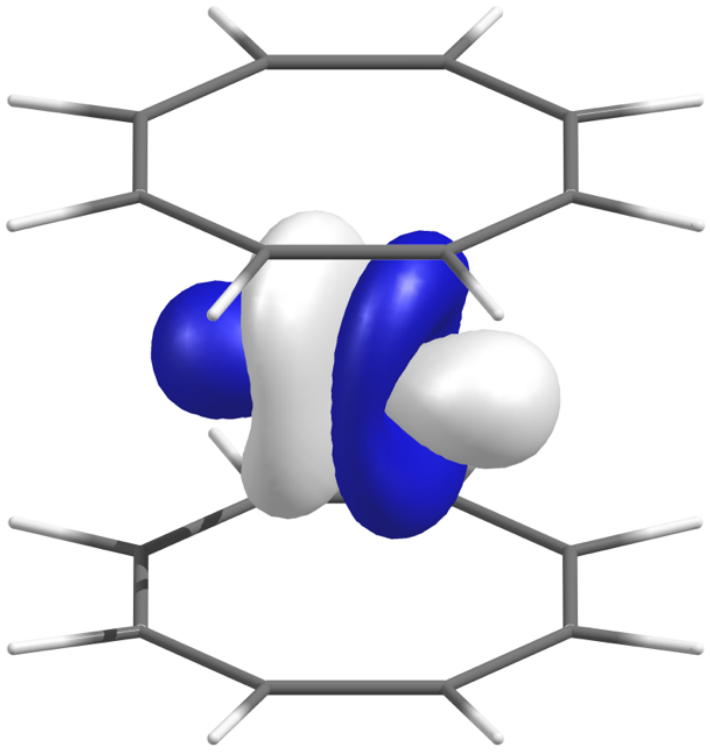

αMO 100 (HOMO–2) –5.65643 eV
U {5$f_{x(x^2-3y^2)/y(3x^2-y^2)}$}

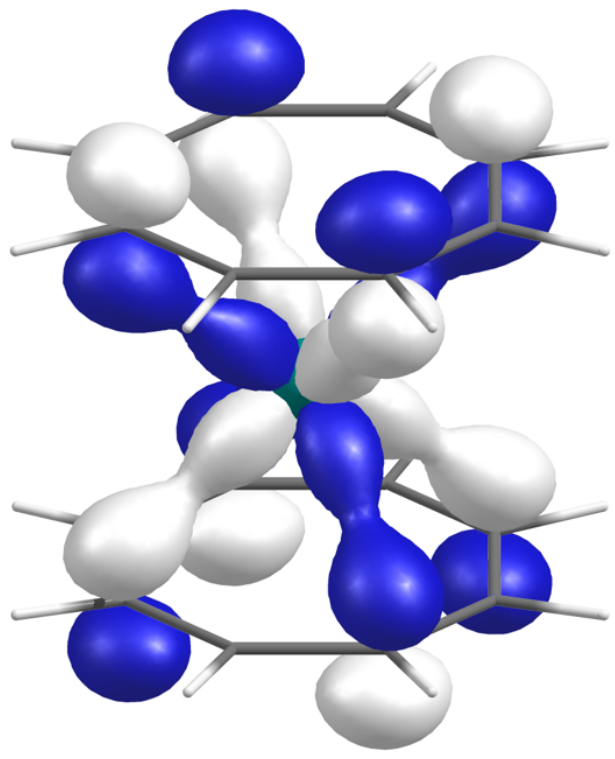

αMO 101 (HOMO–1) –5.62650 eV
U {5$f_{z(x^2-y^2)/xyz}$} + COT δ-bond

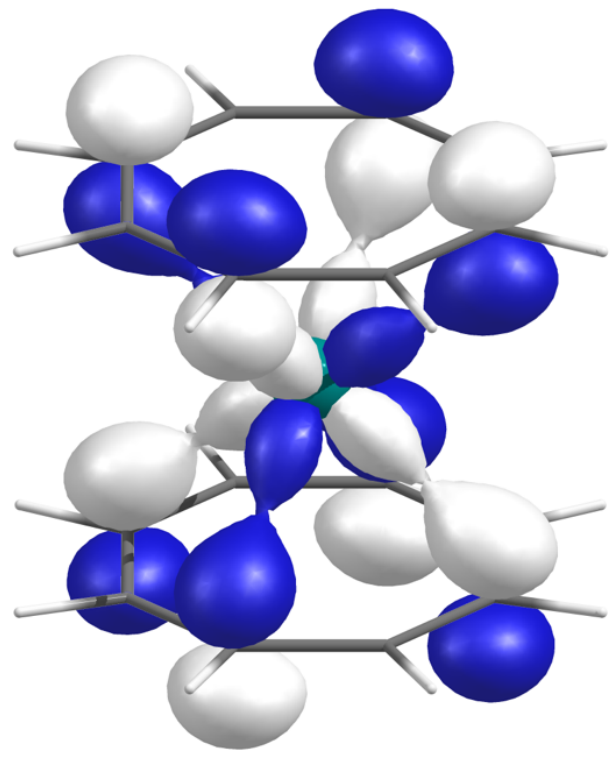

αMO 102 (HOMO) –5.45126 eV
U {5$f_{z(x^2-y^2)/xyz}$} + COT δ-bond

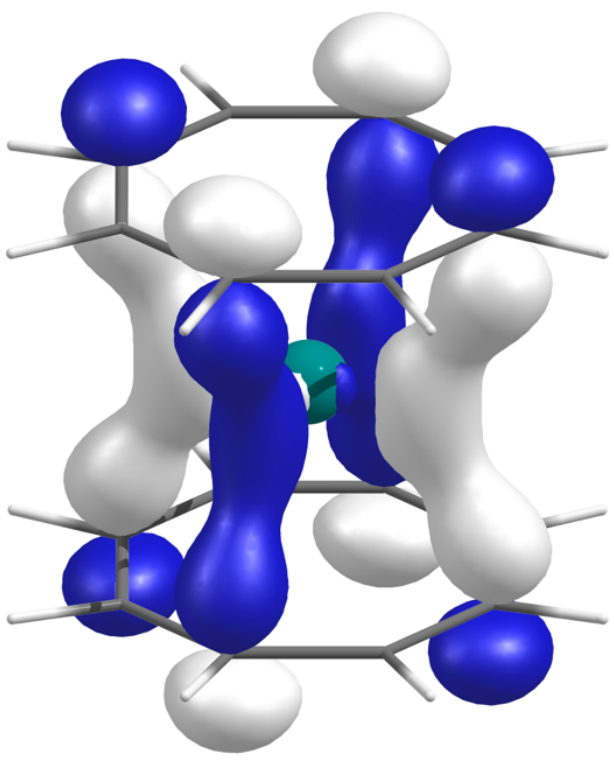

αMO 97 (HOMO–5) –6.41508 eV
U {6$d_{x^2-y^2/xy}$} + COT δ-bond

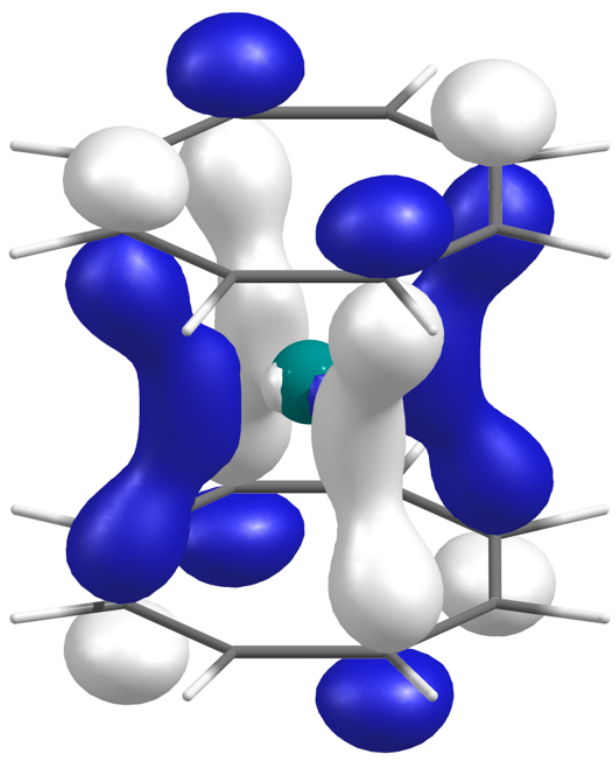

αMO 98 (HOMO–4) –6.33263 eV
U {6$d_{x^2-y^2/xy}$} + COT δ-bond

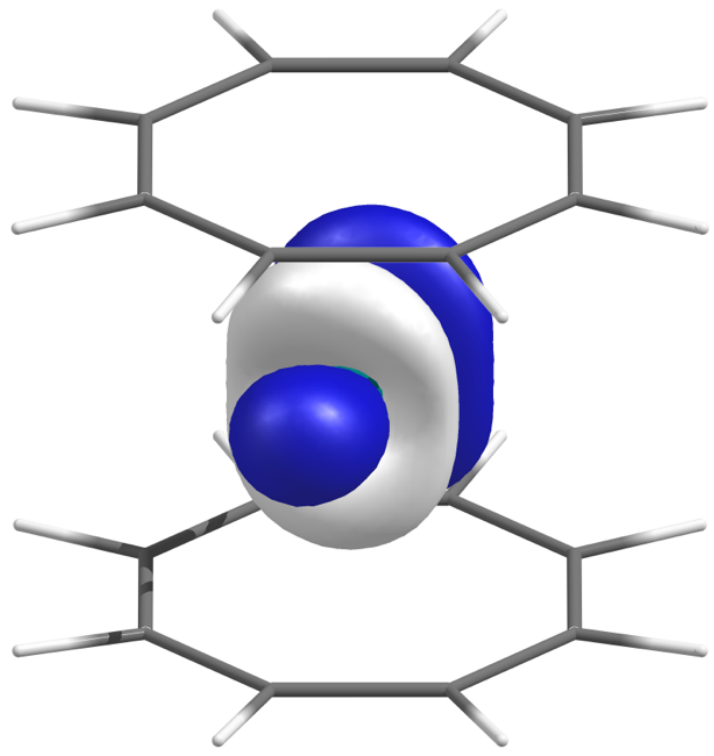

αMO 99 (HOMO–3) –5.65698 eV
U {5f_{z^3}}

Figure S222. Selected MOs and their compositions for **1-D8h**. Isosurface values 0.05 e Å^{-3}.

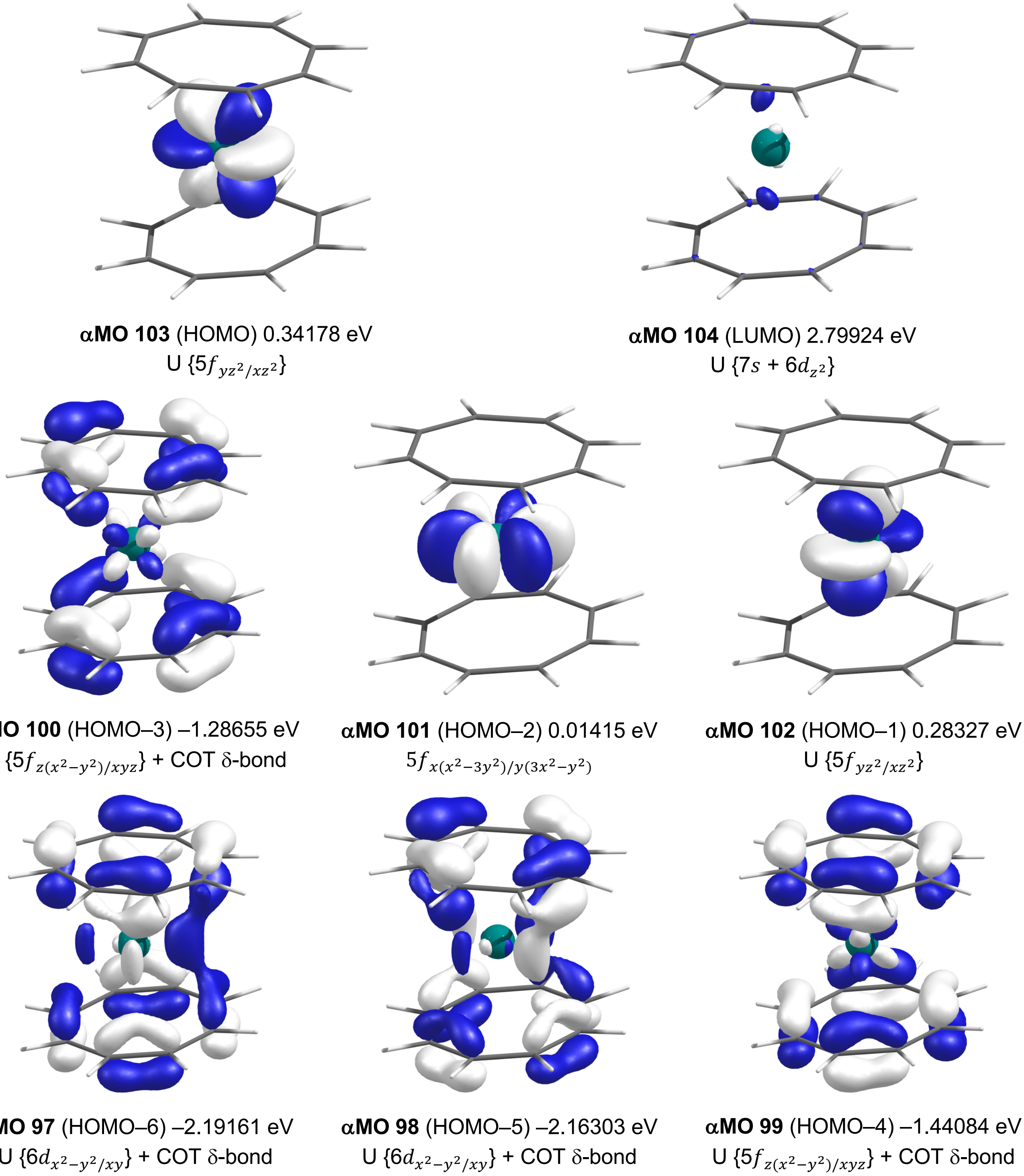

Figure S223. Selected MOs and their compositions for **2-C2v**. Isosurface values 0.05 e Å^{-3}.

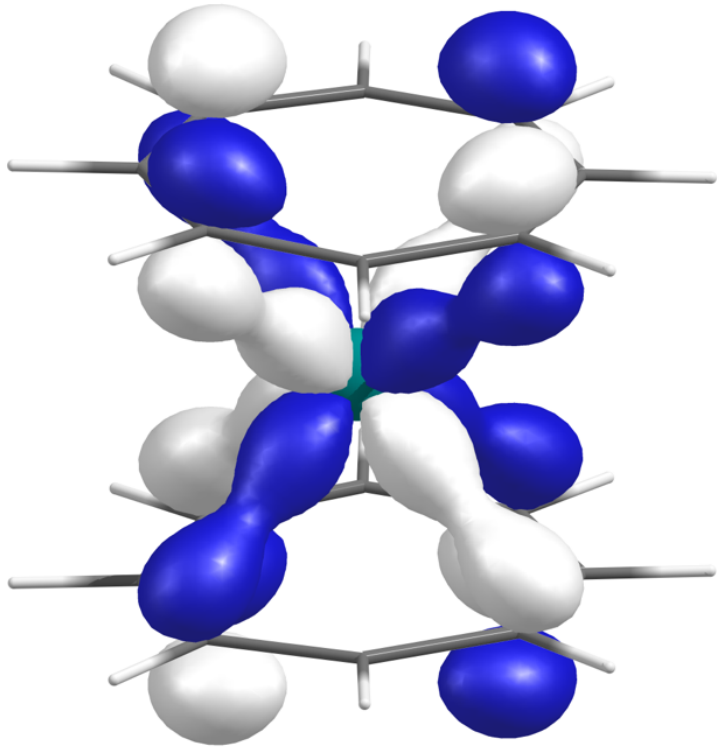

αMO 101 (HOMO) –9.85107 eV
U {$5f_{z(x^2-y^2)/xyz}$} + COT δ-bond

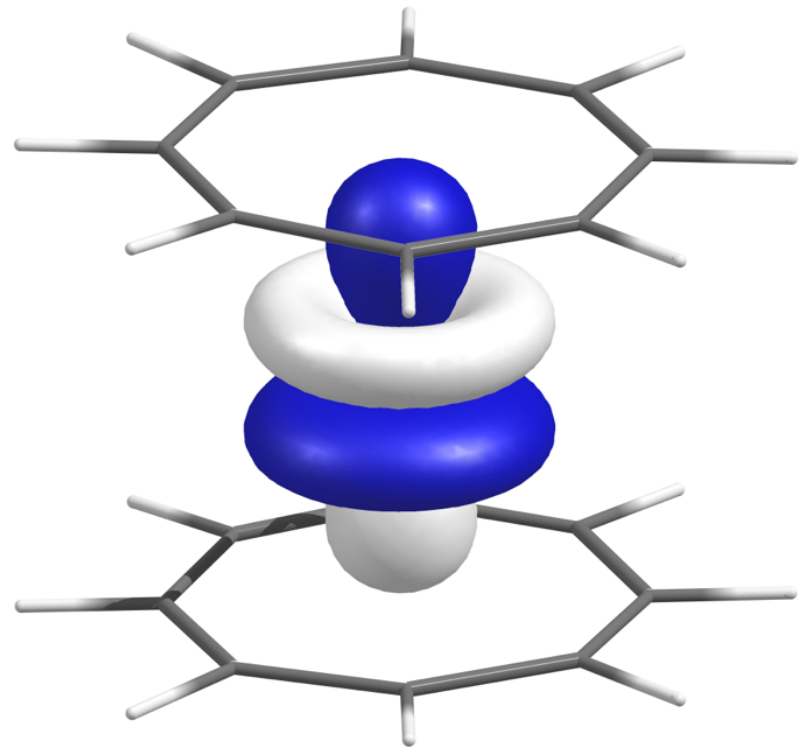

αMO 102 (LUMO) –6.53754 eV
U {$5f_{x(x^2-3y^2)/y(3x^2-y^2)/z^3}$}

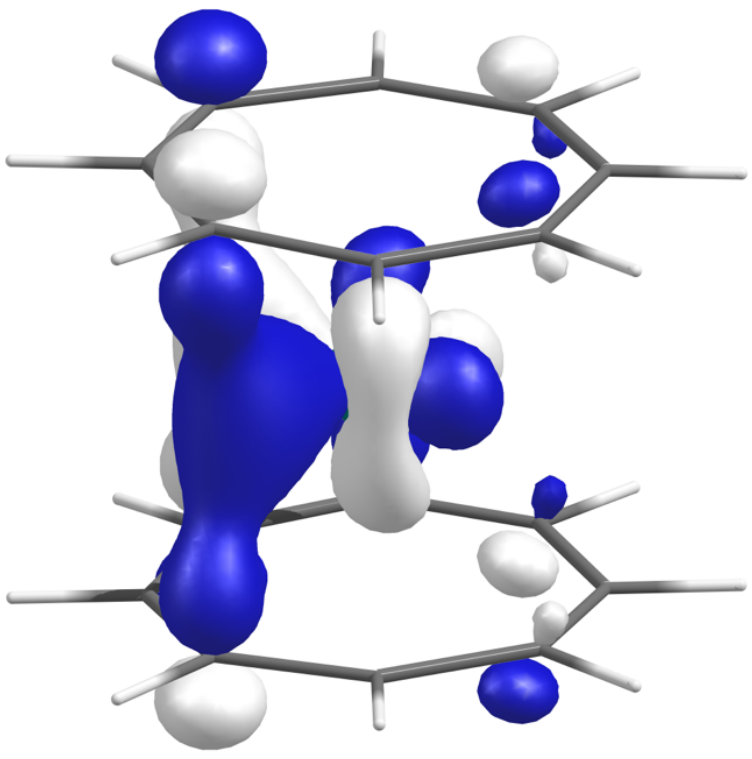

αMO 99 (HOMO–2) –10.53788 eV
U {$6d_{x^2-y^2/xy}$ + $5f_{z^3}$} + COT δ-bond

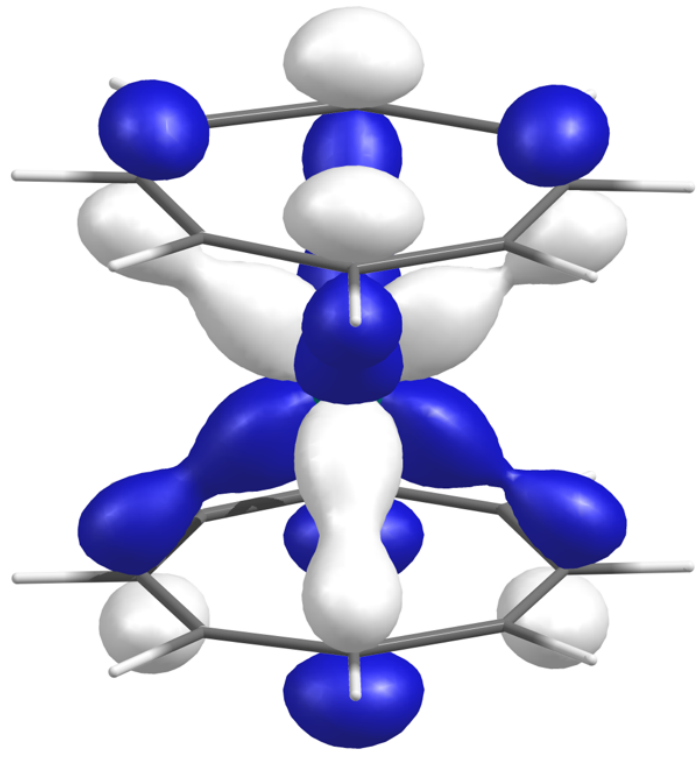

αMO 100 (HOMO–1) –10.16318 eV
U {$5f_{z(x^2-y^2)/xyz}$} + COT δ-bond

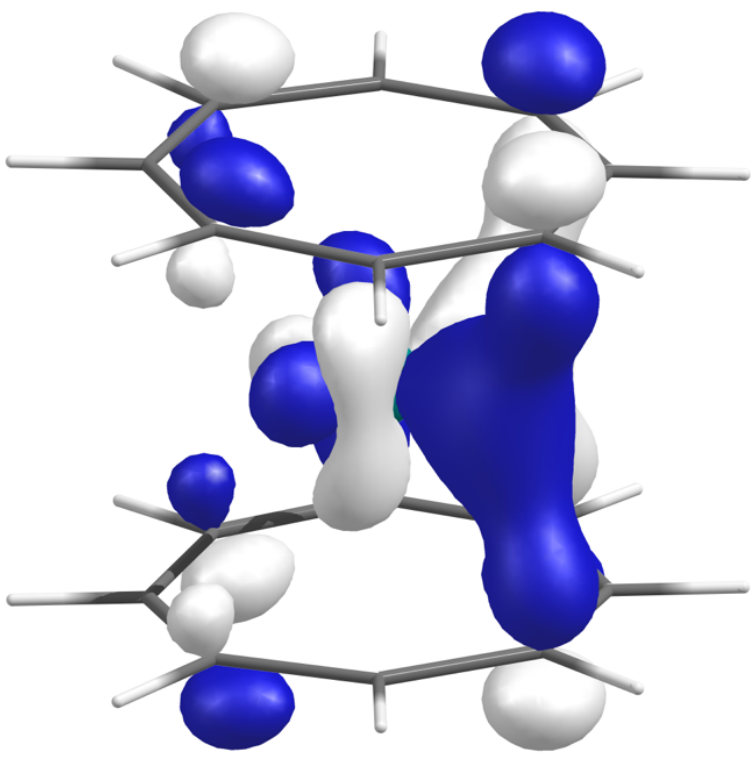

αMO 97 (HOMO–4) –10.62577 eV
U {$5f_{x(x^2-3y^2)/y(3x^2-y^2)/z^3}$}

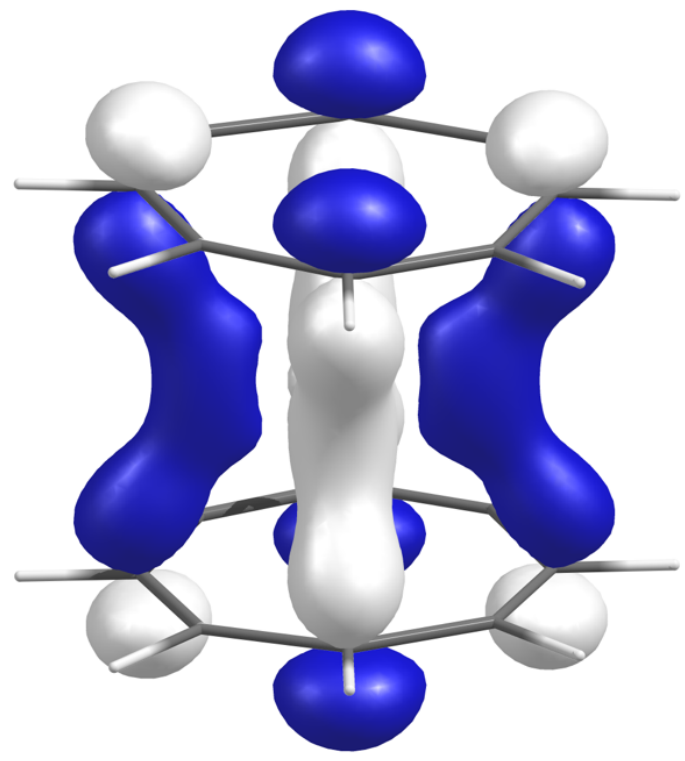

αMO 98 (HOMO–3) –10.60156 eV
U {$6d_{x^2-y^2/xy}$ + $5f_{z^3}$} + COT δ-bond

Figure S224. Selected MOs and their compositions for **3-C2v**. Isosurface values 0.05 e Å^{-3}.

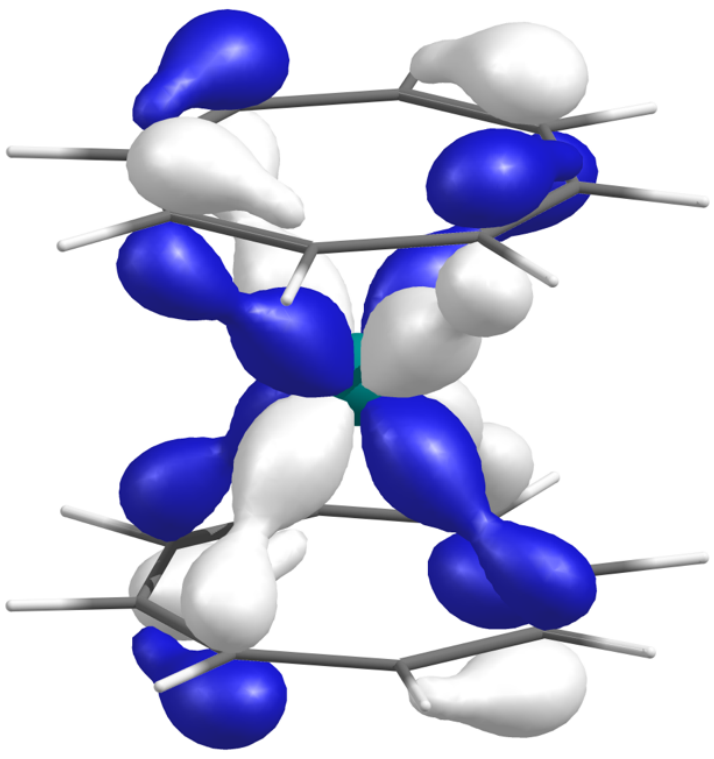

αMO 101 (HOMO) –10.05107 eV
U {5$f_{z(x^2-y^2)/xyz}$} + COT δ-bond

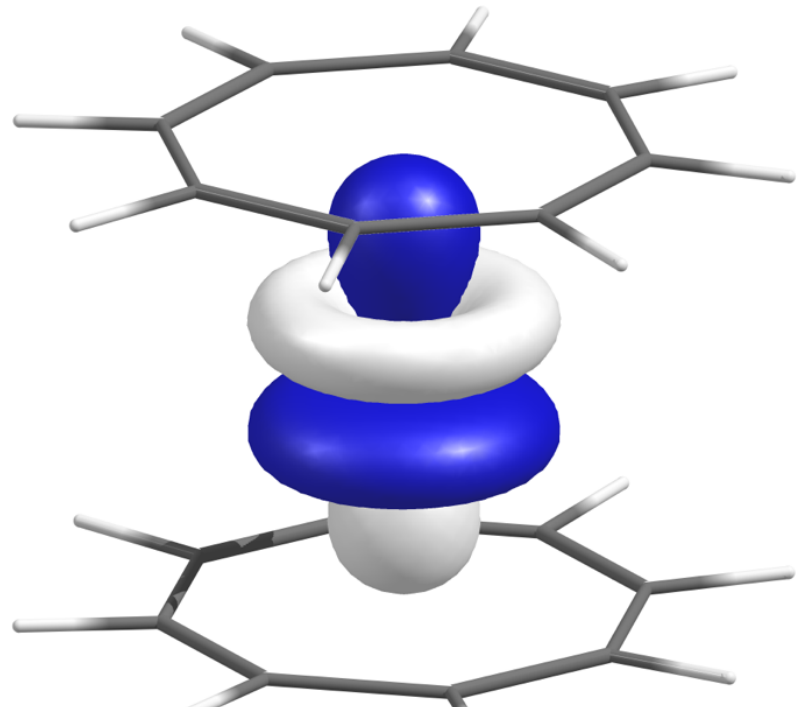

αMO 102 (LUMO) –6.80502 eV
U {5$f_{x(x^2-3y^2)/y(3x^2-y^2)/z^3}$}

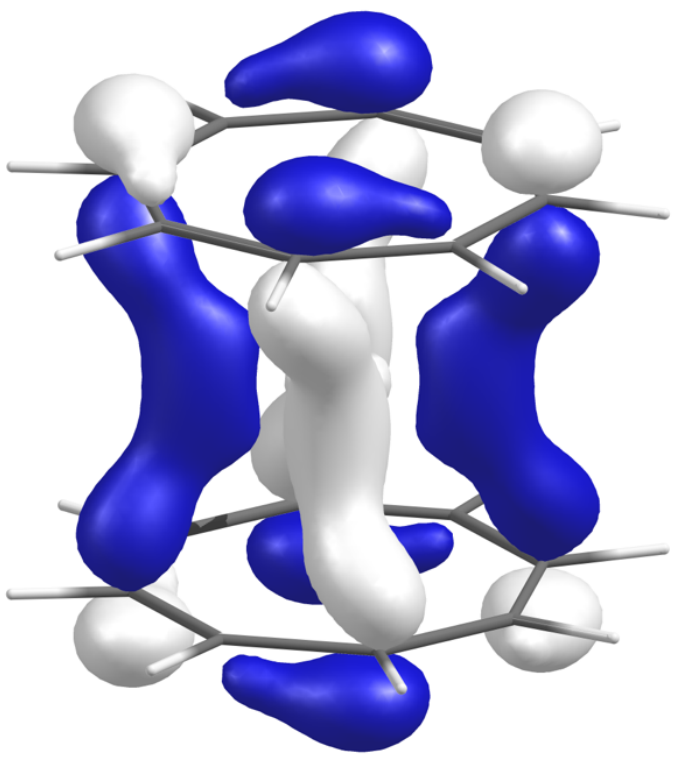

αMO 99 (HOMO–2) –10.50550 eV
U {6$d_{x^2-y^2/xy}$ + 5f_{z^3}} + COT δ-bond

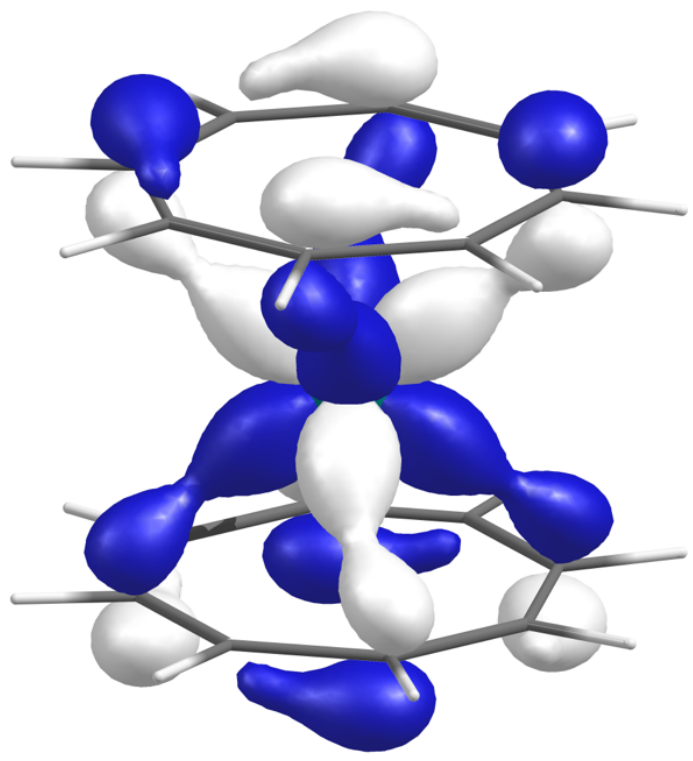

αMO 100 (HOMO–1) –10.07583 eV
U {5$f_{z(x^2-y^2)/xyz}$} + COT δ-bond

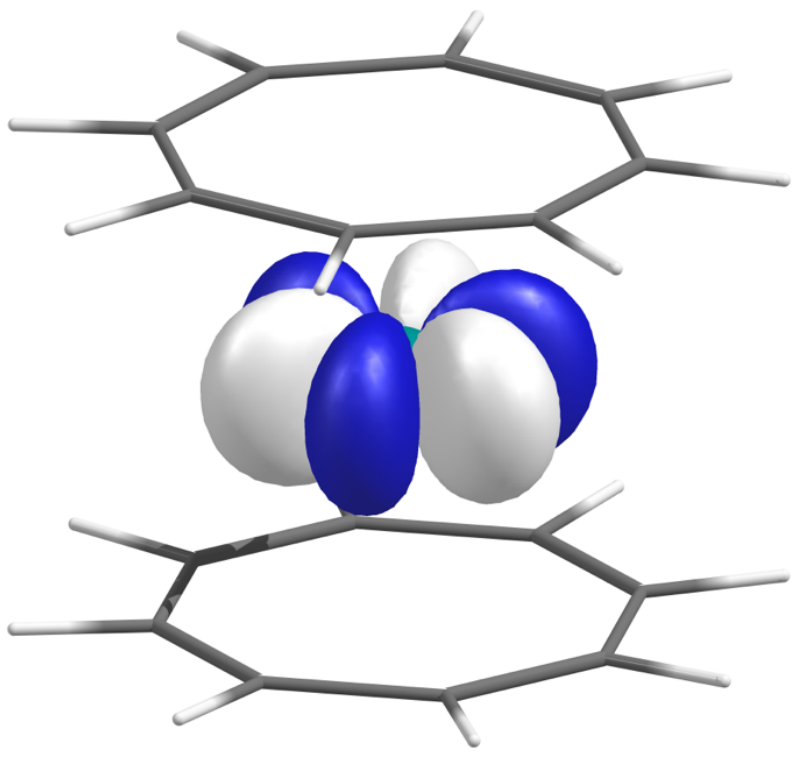

αMO 97 (HOMO–4) –10.52019 eV
U {5$f_{x(x^2-3y^2)/y(3x^2-y^2)/z^3}$}

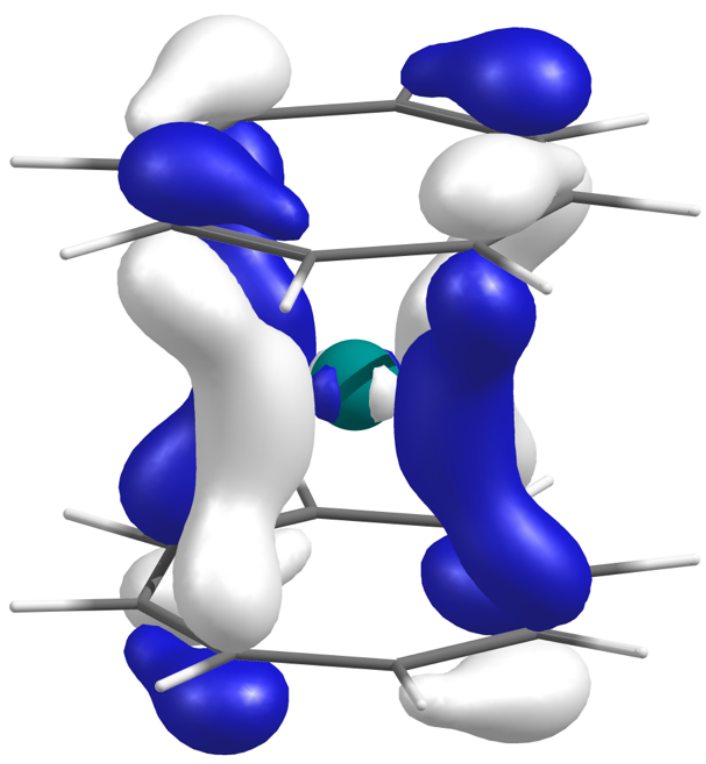

αMO 98 (HOMO–3) –10.50877 eV
U {6$d_{x^2-y^2/xy}$ + 5f_{z^3}} + COT δ-bond

Figure S225. Selected MOs and their compositions for *D*8d **3**. Isosurface values 0.05 e Å^{-3}.

Table S33. Löwdin orbital populations for selected orbitals and interactions in **1-D8h**.

Description	U 5*d*–COT δ-bond		U 5*f*–COT δ-bond		5*f* localized	
**MO#**	**97** HOMO–5	**98** HOMO–4	**101** HOMO–1	**102** HOMO	**99** HOMO–3	**100** HOMO–2
**Energy (eV)**	−6.41508	−6.33263	−5.62650	−5.45126	−5.65698	−5.65643
M *s* (%)	0.0	0.0	0.0	0.0	0.0	0.0
M *p* (%)	0.0	0.0	0.0	0.0	0.6	0.6
M *d* (%)	21.9	21.2	0.0	0.0	0.0	0.0
M *f* (%)	0.0	0.0	28.6	21.7	93.5	93.5
**Total M (%)**	21.9	21.1	28.6	21.7	94.1	94.1

Table S34. Löwdin orbital populations for selected orbitals and interactions in **2-C2v**.

Description	U 5*d*–COT δ-bond		U 5*f*–COT δ-bond		5*f* localized		
**MO#**	**97** HOMO–6	**98** HOMO–5	**99** HOMO–4	**100** HOMO–3	**101** HOMO–2	**102** HOMO–1	**103** HOMO
**Energy (eV)**	−2.19161	−2.16303	−1.44084	−1.28655	0.01415	0.28327	0.34178
M *s* (%)	0.0	0.0	0.0	0.0	0.0	0.0	0.0
M *p* (%)	0.0	0.0	0.0	0.0	0.0	1.0	0.2
M *d* (%)	19.6	19.5	0.0	0.0	0.0	0.0	0.1
M *f* (%)	0.1	0.0	23.6	14.7	92.8	92.6	83.8
**Total M (%)**	19.7	19.5	23.6	14.7	92.8	93.6	84.1

Table S35. Löwdin orbital populations for selected orbitals and interactions in **3-C2v**.

Description	U 5*d*–COT δ-bond		U 5*f*–COT δ-bond		5*f* localized
**MO#**	**98** HOMO–3	**99** HOMO–2	**100** HOMO–1	**101** HOMO	**97** HOMO–4
**Energy (eV)**	−10.60156	−10.53788	−10.16318	−9.85107	−10.62577
M *s* (%)	0.0	0.0	0.0	0.0	0.0
M *p* (%)	0.0	0.2	0.0	0.0	0.1
M *d* (%)	21.1	9.4	0.0	0.0	11.7
M *f* (%)	0.0	53.3	44.5	35.9	40.4
**Total M (%)**	21.1	62.9	44.5	35.9	52.2

Table S36. Löwdin orbital populations for selected orbitals and interactions in **3-D8d**.

**Description**	**U 5*d*–COT δ-bond**		**U 5*f*–COT δ-bond**		**5*f* localized**
**MO#**	**98**<br>HOMO–3	**99**<br>HOMO–2	**100**<br>HOMO–1	**101**<br>HOMO	**97**<br>HOMO–4
**Energy (eV)**	−10.50877	−10.50550	−10.07583	−10.05107	−10.52019
M *s* (%)	0.0	0.0	0.0	0.0	0.0
M *p* (%)	0.0	0.0	0.0	0.0	0.0
M *d* (%)	22.1	22.1	0.0	0.0	0.0
M *f* (%)	0.0	0.0	44.1	44.3	95.7
**Total M (%)**	22.1	22.1	44.1	44.3	95.7

Table S37. A comparison of Löwdin orbital populations for selected orbitals and interactions for H-atom optimized (**1**), fully optimized (**1-Op**), and D_{8h} (**1-D8h**) coordinates for **1**.

**Description**	**U 5*d*–COT δ-bond**			**U 5*f*–COT δ-bond**			**5*f* localized**		
	**1**	**1-Op**	**1-D8h**	**1**	**1-Op**	**1-D8h**	**1**	**1-Op**	**1-D8h**
**Energy (eV)**	−6.45889<br>−6.38706	−6.43114<br>−6.42978	−6.41508<br>−6.33263	−5.69235<br>−5.63085	−5.65126<br>−5.64772	−5.62650<br>−5.45126	−5.88446<br>−5.71058	−5.73344<br>−5.57099	−5.65698<br>−5.65643
M *s* (%)	0.0<br>0.0	0.0<br>0.0	0.0<br>0.0	0.0<br>0.0	0.0<br>0.0	0.0<br>0.0	0.0<br>0.0	0.0<br>0.0	0.0<br>0.0
M *p* (%)	0.0<br>0.0	0.0<br>0.0	0.0<br>0.0	0.0<br>0.0	0.0<br>0.0	0.0<br>0.0	0.4<br>0.0	0.0<br>0.0	0.6<br>0.6
M *d* (%)	21.2<br>21.3	21.3<br>21.3	21.9<br>21.2	0.0<br>0.0	0.0<br>0.0	0.0<br>0.0	0.0<br>0.0	0.0<br>0.0	0.0<br>0.0
M *f* (%)	0.0<br>0.0	0.0<br>0.0	0.0<br>0.0	33.7<br>28.1	26.6<br>26.3	28.6<br>21.7	91.3<br>88.4	92.1<br>95.2	93.5<br>93.5
**Total M (%)**	21.2<br>21.3	21.3<br>21.3	21.9<br>21.2	33.7<br>28.1	26.6<br>26.3	28.6<br>21.7	91.7<br>88.4	92.1<br>95.2	94.1<br>94.1

Table S38. A comparison of Löwdin orbital populations for selected orbitals and interactions for H-atom optimized (**2**), fully optimized (**2-Op**), and C_{2v} (**2-C2v**) coordinates for **2**.

**Description**	**U 5*d*–COT δ-bond**			**U 5*f*–COT δ-bond**			**5*f* localized**		
	**2**	**2-Op**	**2-C2v**	**2**	**2-Op**	**2-C2v**	**2**	**2-Op**	**2-C2v**
**Energy (eV)**	−2.24793<br>−2.20929	−2.26317<br>−2.26154	−2.19161<br>−2.16303	−1.36492<br>−1.32138	−1.42016<br>−1.41799	−1.44084<br>−1.28655	−0.06123<br>0.12925<br>0.13061	−0.60981<br>0.53525<br>0.54613	0.01415<br>0.28327<br>0.34178
M *s* (%)	0.0<br>0.0	0.0<br>0.0	0.0<br>0.0	0.0<br>0.0	0.0<br>0.0	0.0<br>0.0	0.0<br>0.0<br>0.0	0.0<br>0.0<br>0.0	0.0<br>0.0<br>0.0
M *p* (%)	0.0<br>0.0	0.0<br>0.0	0.0<br>0.0	0.0<br>0.0	0.0<br>0.0	0.0<br>0.0	0.0<br>1.0<br>0.9	0.4<br>0.0<br>0.0	0.0<br>1.0<br>0.2
M *d* (%)	19.5<br>19.6	19.7<br>19.6	19.6<br>19.5	0.0<br>0.0	0.0<br>0.0	0.0<br>0.0	0.1<br>0.1<br>0.1	0.0<br>0.0<br>0.0	0.0<br>0.0<br>0.1
M *f* (%)	0.1<br>0.2	0.0<br>0.0	0.1<br>0.0	14.4<br>14.5	16.6<br>16.5	23.6<br>14.7	93.0<br>92.7<br>92.8	94.1<br>90.0<br>89.2	92.8<br>92.6<br>83.8
**Total M (%)**	19.6<br>19.8	19.7<br>19.6	19.7<br>19.5	14.4<br>14.5	16.6<br>16.5	23.6<br>14.7	93.1<br>93.8<br>93.8	94.5<br>90.0<br>89.2	92.8<br>93.6<br>84.1

Table S39. A comparison of Löwdin orbital populations for selected orbitals and interactions for H-atom optimized (**3**), symmetry optimized (**3-Op**), C_{2v} (**3-C2v**), and D_{8d} (**3-D8d**) coordinates for **3**.

Description	U 5*d*–COT δ-bond				U 5*f*–COT δ-bond				5*f* localized			
	**3**	**3-Op**	**3-C2v**	**3-D8d**	**3**	**3-Op**	**3-C2v**	**3-D8d**	**3**	**3-Op**	**3-C2v**	**3-D8d**
**Energy (eV)**	−10.67884<br>−10.64564	−10.62686<br>−10.62605	−10.60156<br>−10.53788	−10.50877<br>−10.50550	−10.23692<br>−10.21461	−10.17434<br>−10.17379	−10.16318<br>−9.85107	−10.07583<br>−10.05107	−10.72292<br>−6.67250	−10.56618<br>−6.84502	−10.62577<br>−6.53754	−10.52019<br>−6.80502
M *s* (%)	0.0<br>0.0	0.0<br>0.0	0.0<br>0.0	0.0<br>0.0	0.0<br>0.0	0.0<br>0.0	0.0<br>0.0	0.0<br>0.0	0.0<br>0.0	0.0<br>0.0	0.0<br>0.0	0.0<br>0.0
M *p* (%)	0.0<br>0.1	0.0<br>0.0	0.0<br>0.2	0.0<br>0.0	0.0<br>0.0	0.0<br>0.0	0.0<br>0.0	0.0<br>0.0	0.3<br>0.1	0.0<br>0.5	0.1<br>0.6	0.0<br>0.5
M *d* (%)	20.3<br>19.2	22.2<br>22.3	21.2<br>9.4	22.1<br>22.1	0.2<br>0.3	0.0<br>0.0	0.0<br>0.0	0.0<br>0.0	5.0<br>0.0	0.0<br>0.0	11.7<br>0.0	0.0<br>0.0
M *f* (%)	7.7<br>13.5	0.0<br>0.0	0.0<br>53.3	0.0<br>0.0	42.8<br>42.4	42.0<br>42.0	44.5<br>35.9	44.1<br>43.2	69.9<br>89.6	95.7<br>92.0	40.4<br>91.6	95.7<br>92.0
**Total M (%)**	28.0<br>32.8	22.2<br>22.3	21.2<br>62.9	22.1<br>22.1	43.0<br>42.7	42.0<br>42.0	44.5<br>35.9	44.1<br>43.2	75.2<br>89.7	95.7<br>92.5	52.2<br>92.2	95.7<br>92.5

Table S40. A comparison of Löwdin orbital population for selected orbitals and interactions for H-atom optimized coordinates for **1–3**.

**Description**	**U 5*d*–COT δ-bond**			**U 5*f*–COT δ-bond**			**5*f* localized**		
	**1 U(IV)**	**2 U(III)**	**3 U(V)**	**1 U(IV)**	**2 U(III)**	**3 U(V)**	**1 U(IV)**	**2 U(III)**	**3 U(V)**
**Energy (eV)**	−6.45889<br>−6.38706	−2.24793<br>−2.20929	−10.67884<br>−10.64564	−5.69235<br>−5.63085	−1.36492<br>−1.32138	−10.23692<br>−10.21461	−5.88446<br>−5.71058	−0.06123<br>0.12925<br>0.13061	−10.72292<br>−6.67250
M *s* (%)	0.0<br>0.0	0.0<br>0.0	0.0<br>0.0	0.0<br>0.0	0.0<br>0.0	0.0<br>0.0	0.0<br>0.0	0.0<br>0.0<br>0.0	0.0<br>0.0
M *p* (%)	0.0<br>0.0	0.0<br>0.0	0.0<br>0.1	0.0<br>0.0	0.0<br>0.0	0.0<br>0.0	0.4<br>0.0	0.0<br>1.0<br>0.9	0.3<br>0.1
M *d* (%)	21.2<br>21.3	19.5<br>19.6	20.3<br>19.2	0.0<br>0.0	0.0<br>0.0	0.2<br>0.3	0.0<br>0.0	0.1<br>0.1<br>0.1	5.0<br>0.0
M *f* (%)	0.0<br>0.0	0.1<br>0.2	7.7<br>13.5	33.7<br>28.1	14.4<br>14.5	42.8<br>42.4	91.3<br>88.4	93.0<br>92.7<br>92.8	69.9<br>89.6
**Total M (%)**	21.2<br>21.3	19.6<br>19.8	28.0<br>32.8	33.7<br>28.1	14.4<br>14.5	43.0<br>42.7	91.7<br>88.4	93.1<br>93.8<br>93.8	75.2<br>89.7

*Complexes **4**–**6***

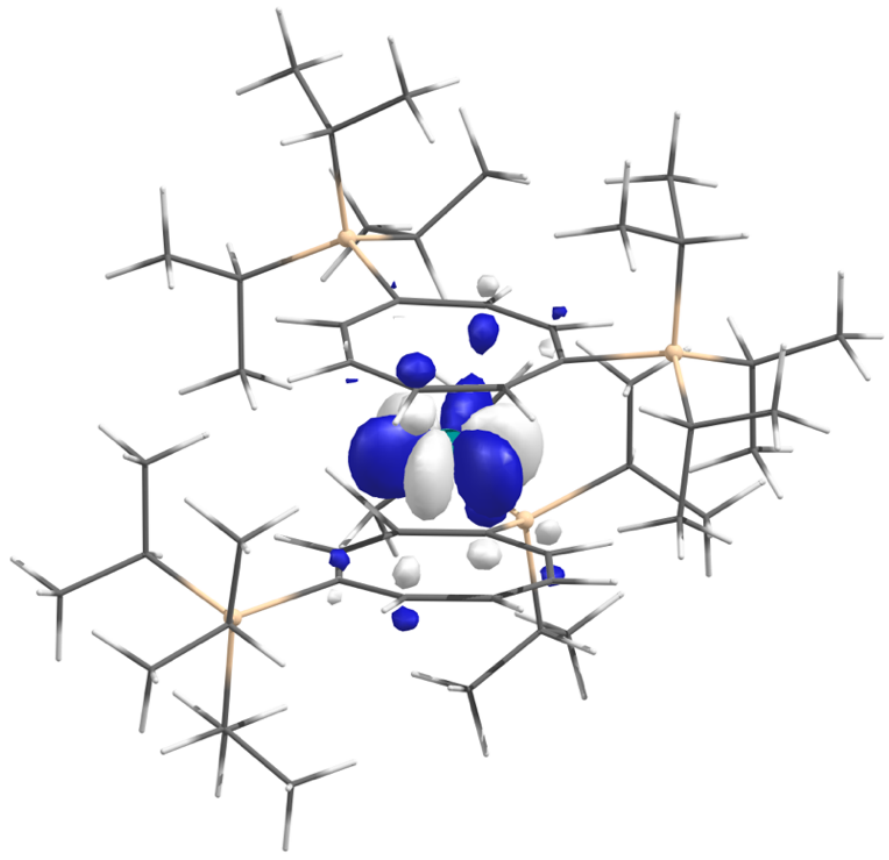

αMO 279 (LUMO) –1.32329 eV
U {5$f_{x(x^2-3y^2)/y(3x^2-y^2)}$}

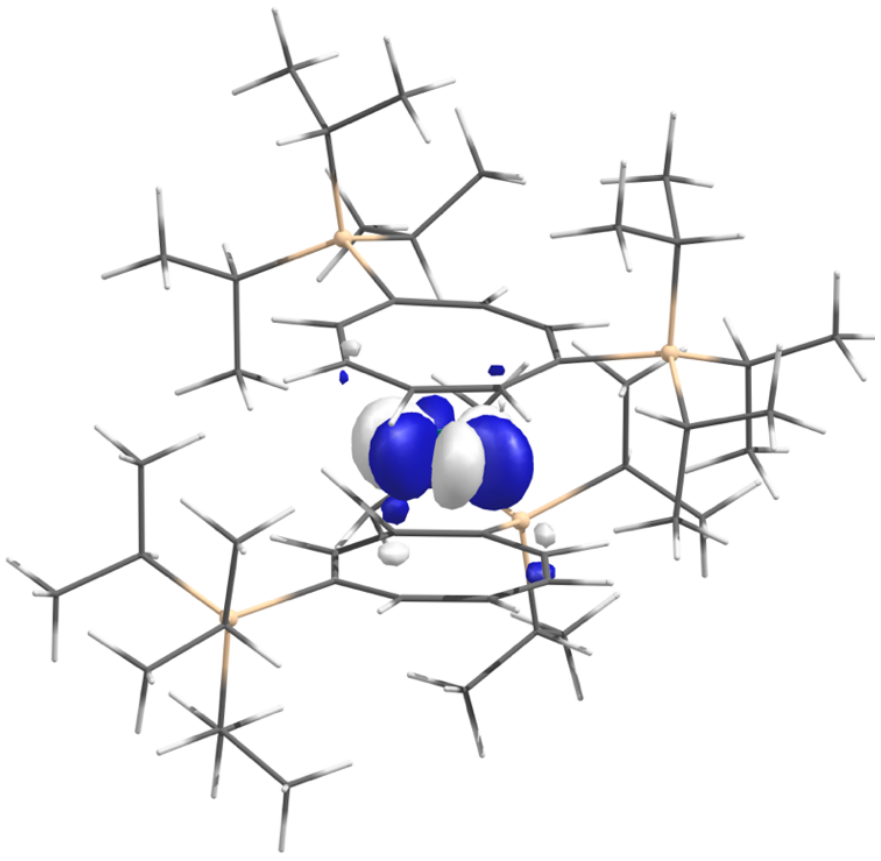

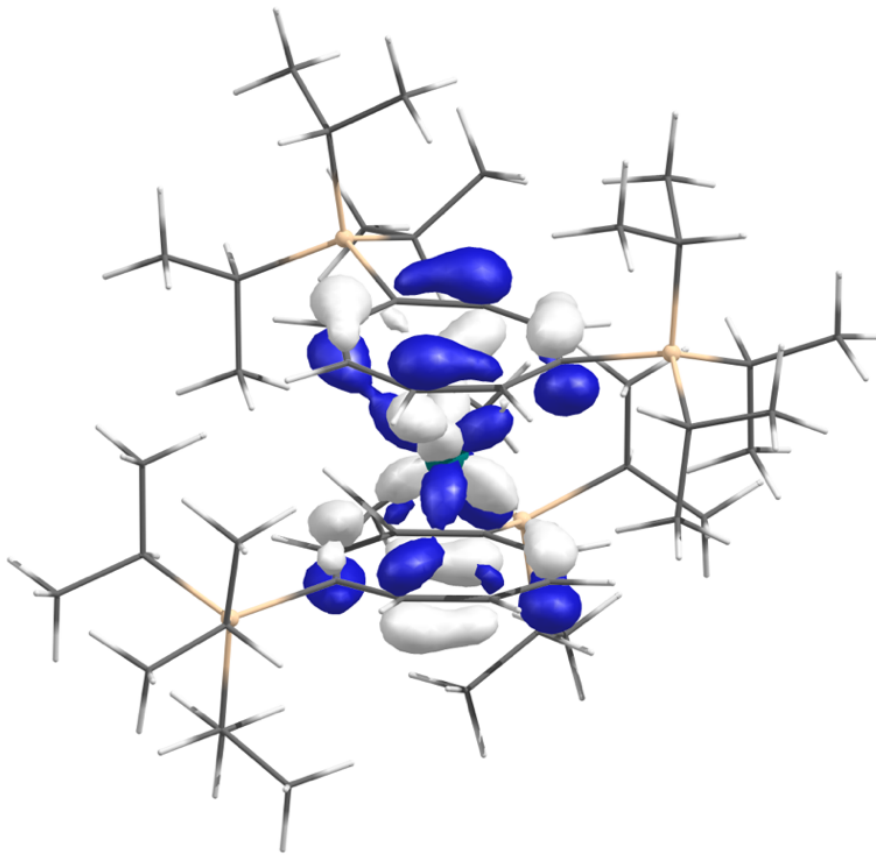

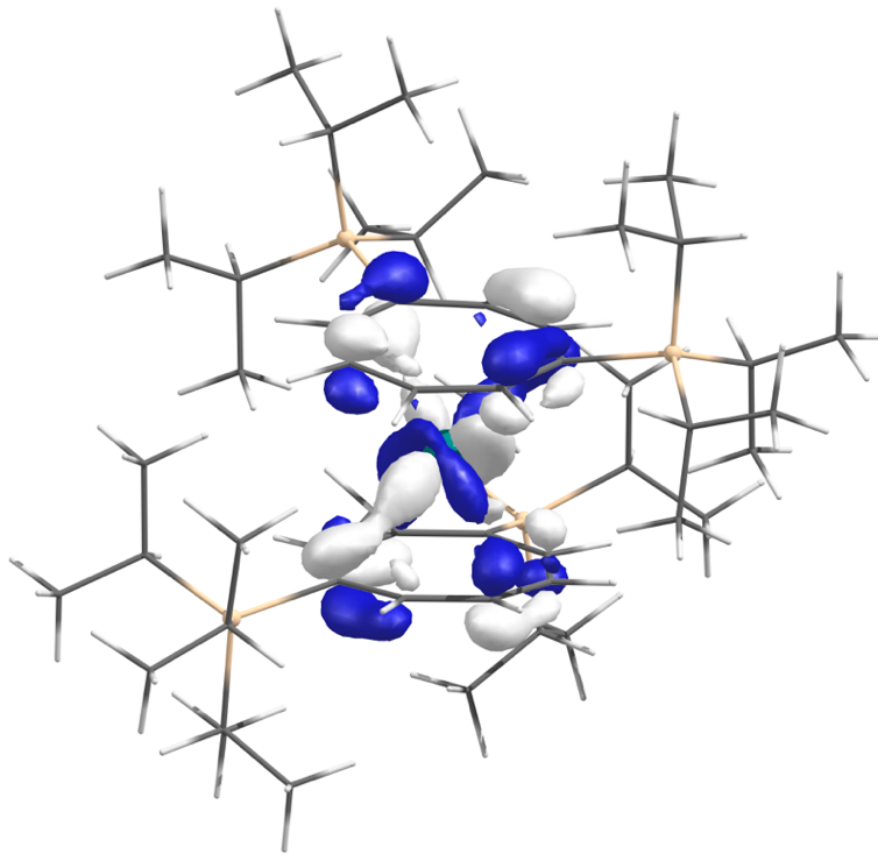

αMO 276 (HOMO–2) –5.61262 eV
U {5$f_{x(x^2-3y^2)/y(3x^2-y^2)}$}

αMO 277 (HOMO–1) –5.55139 eV
U {5$f_{z(x^2-y^2)/xyz}$} + COT δ-bond

αMO 278 (HOMO) –5.52609 eV
U {5$f_{x(x^2-3y^2)/y(3x^2-y^2)/yz^2/xz^2}$} + COT δ-bond

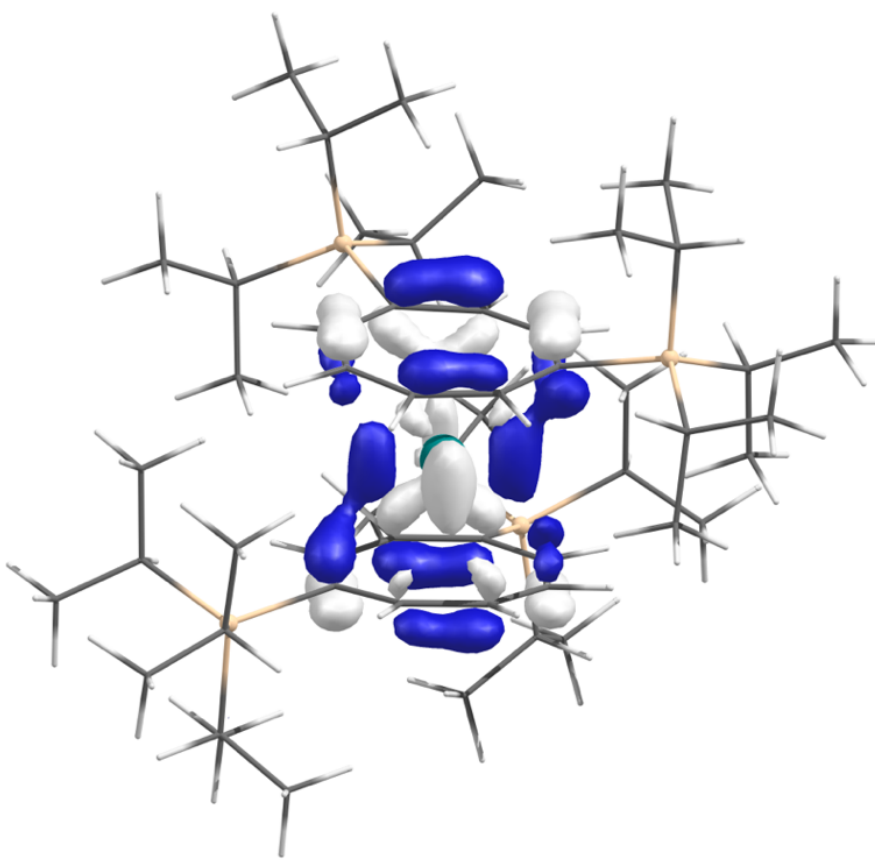

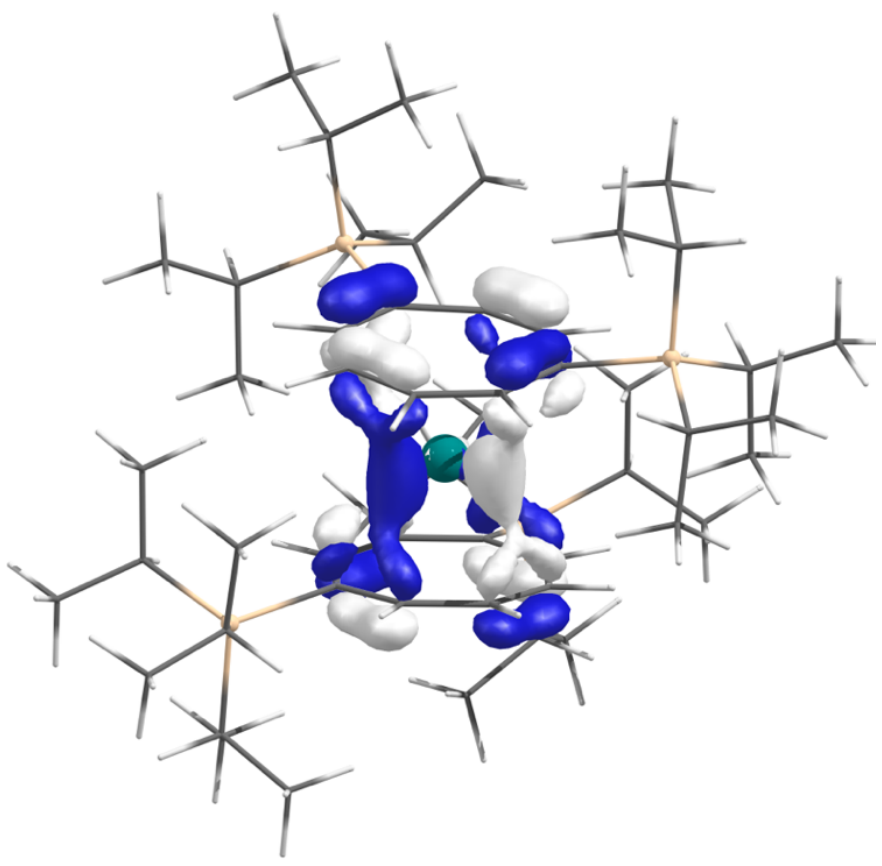

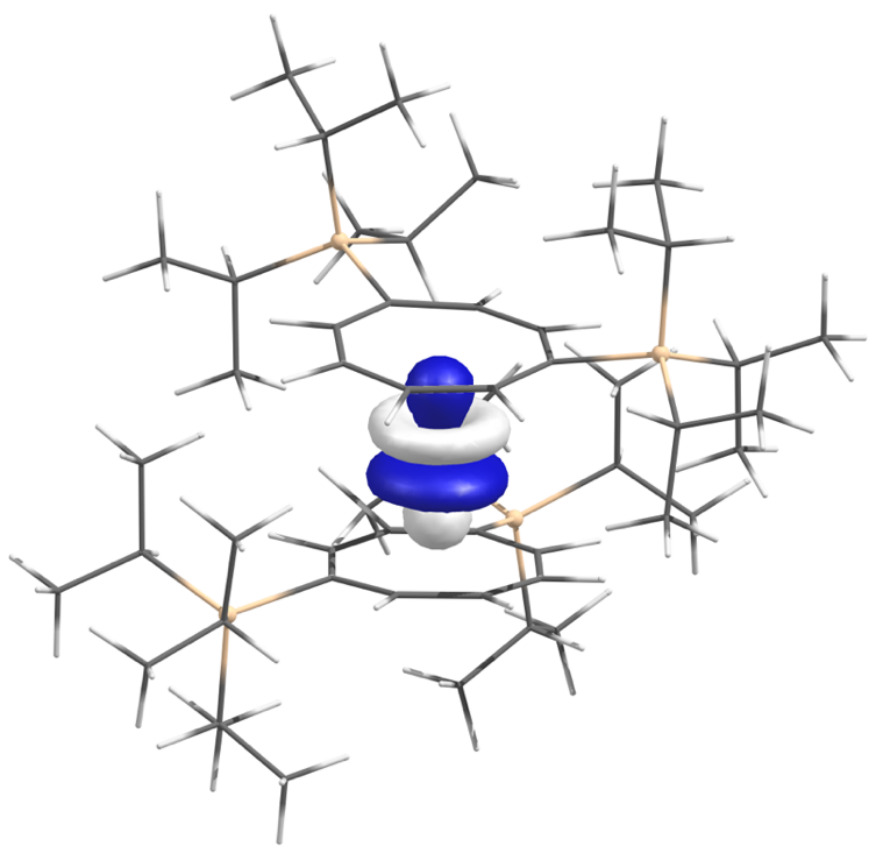

αMO 273 (HOMO–5) –6.23658 eV
U {6$d_{x^2-y^2/xy}$} + COT δ-bond

αMO 274 (HOMO–4) –6.21399 eV
U {6$d_{x^2-y^2/xy}$} + COT δ-bond

αMO 275 (HOMO–3) –5.38432 eV
U {5f_{z^3}}

Figure S226. Selected MOs and their compositions for **4**. Isosurface values 0.05 e Å^{-3}.

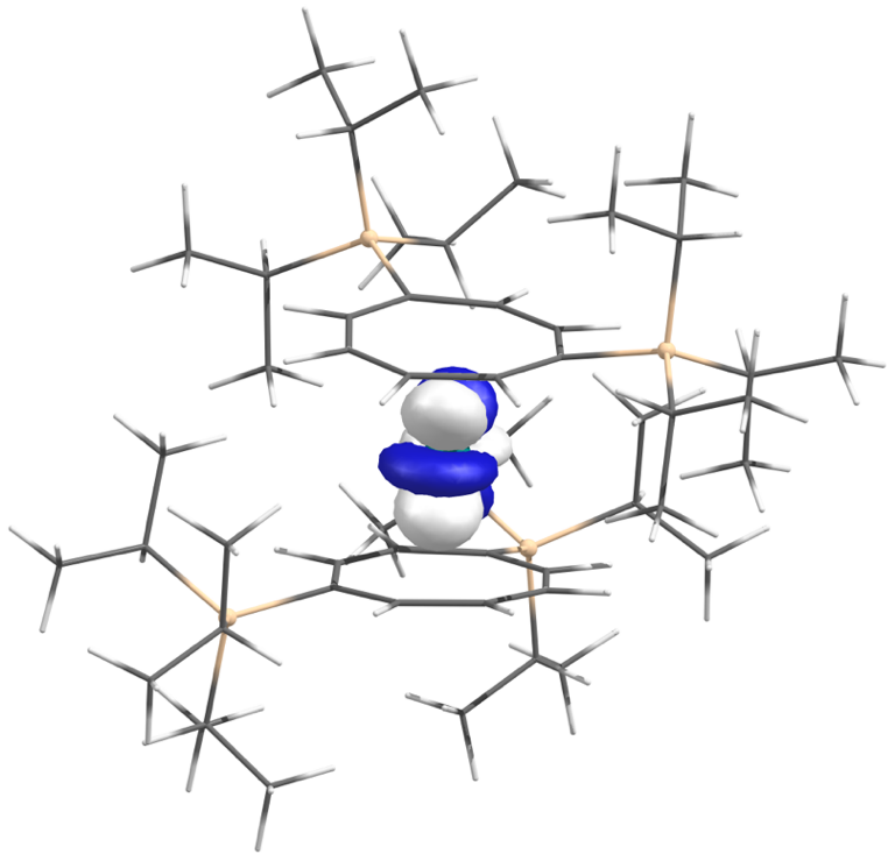

αMO 279 (HOMO) –0.88355 eV
U {$5f_{yz^2/xz^2}$}

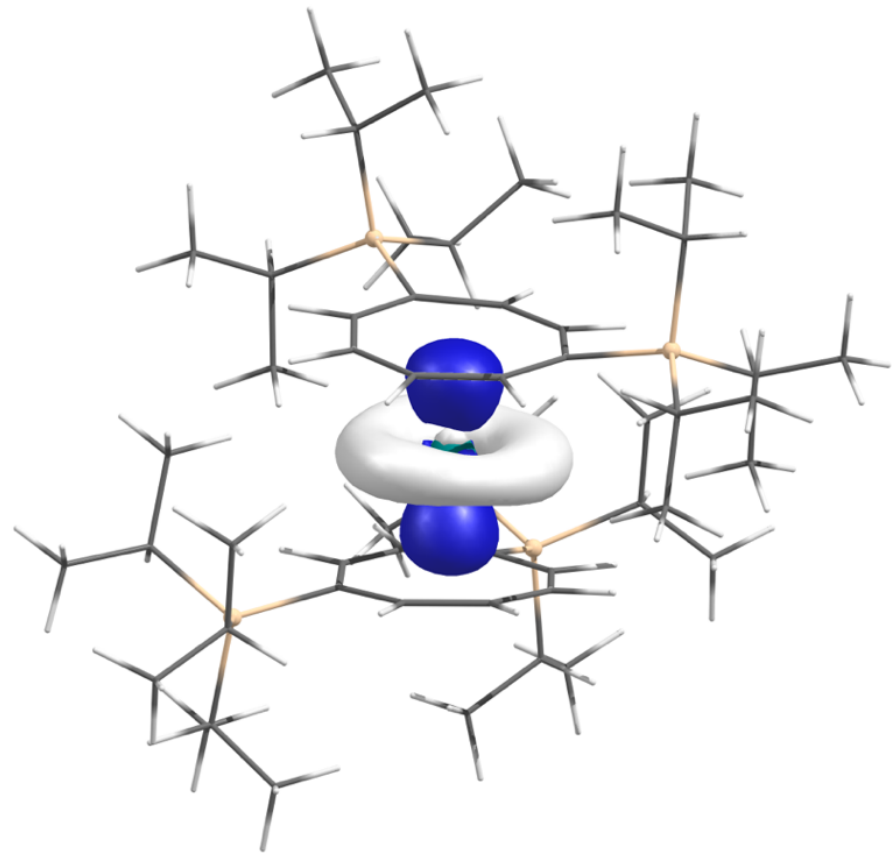

αMO 280 (LUMO) 2.19950 eV
U {$7s$ + $6d_{z^2}$}

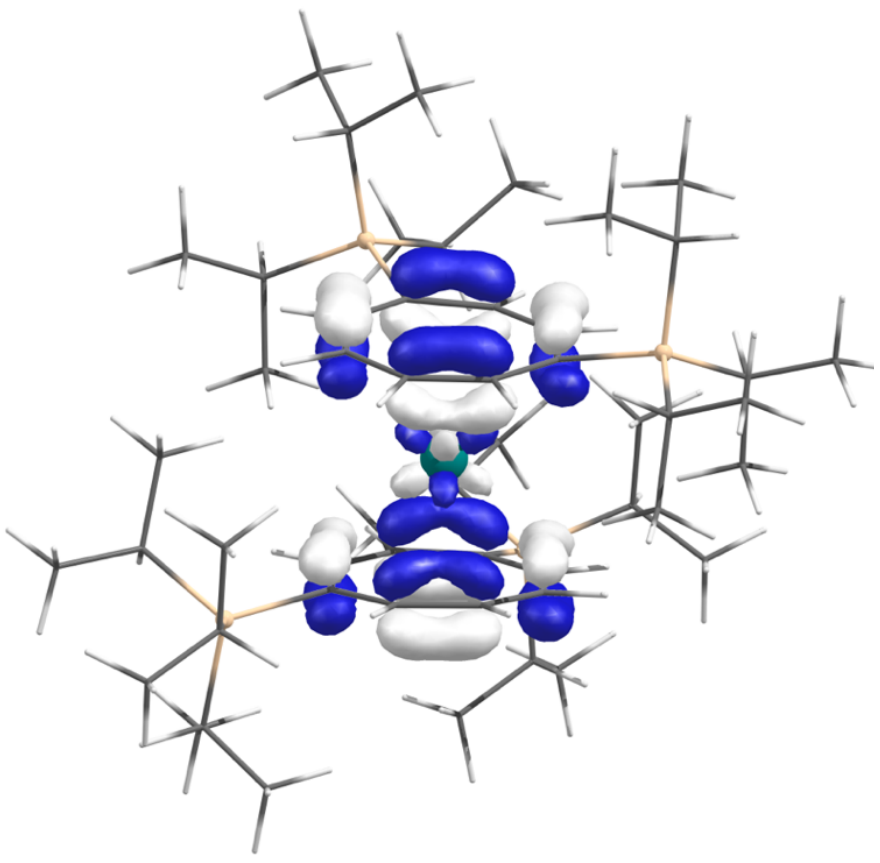

αMO 276 (HOMO–3) –2.15596 eV
U {$5f_{z(x^2-y^2)/xyz}$} + COT δ-bond

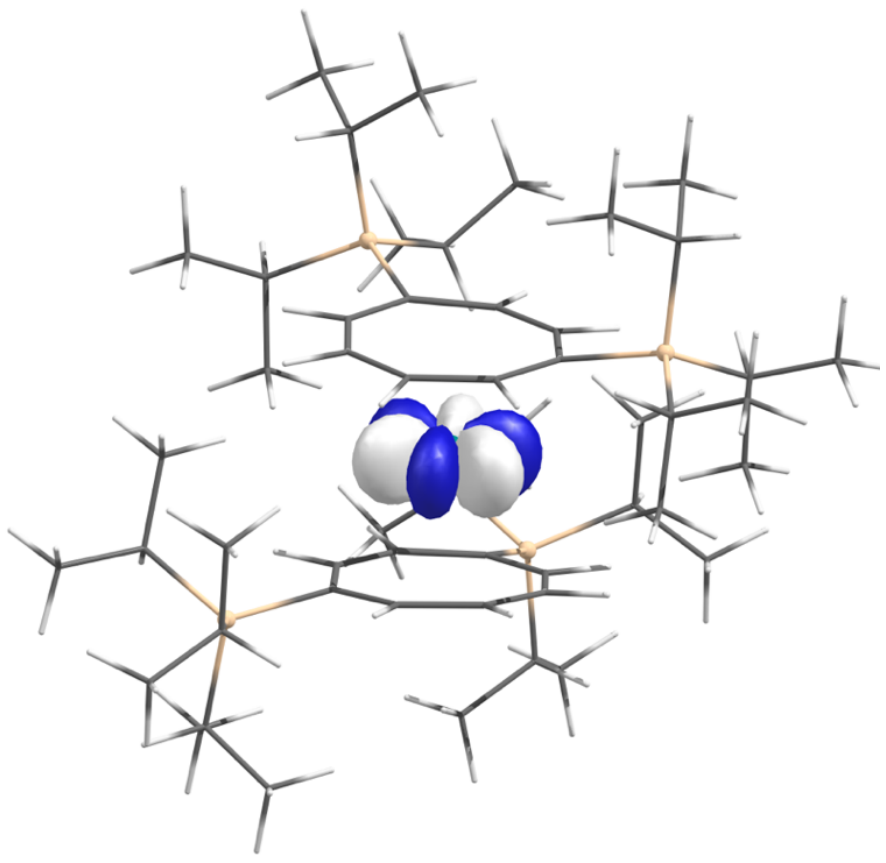

αMO 277 (HOMO–2) –1.07594 eV
U {$5f_{x(x^2-3y^2)/y(3x^2-y^2)}$}

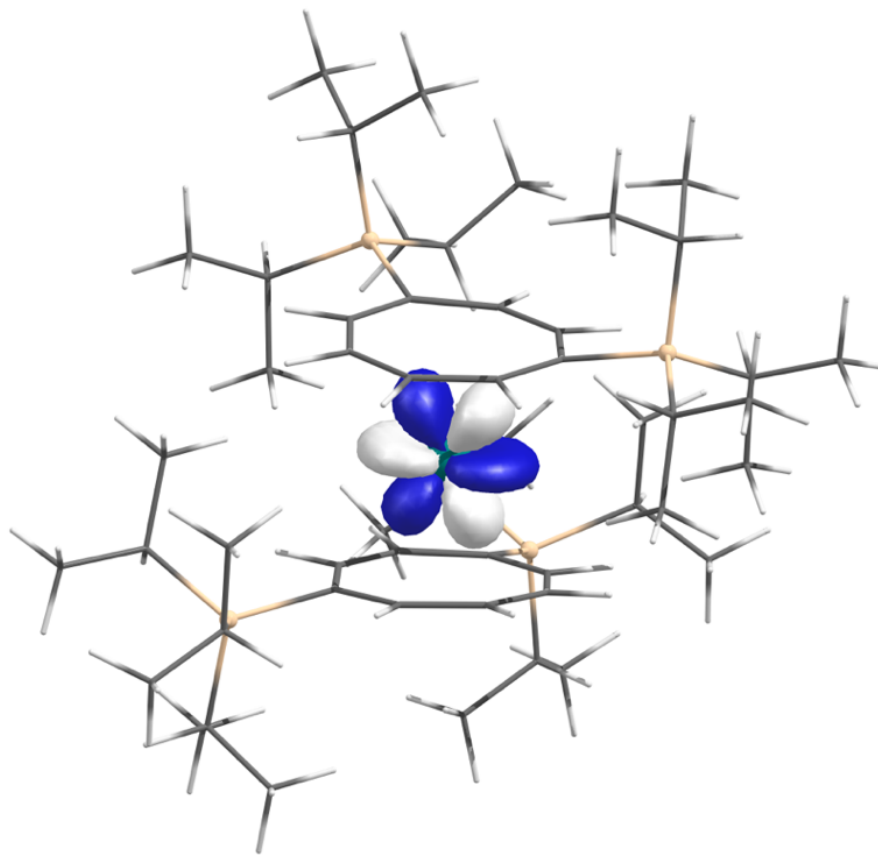

αMO 278 (HOMO–1) –0.88437 eV
U {$5f_{yz^2/xz^2}$}

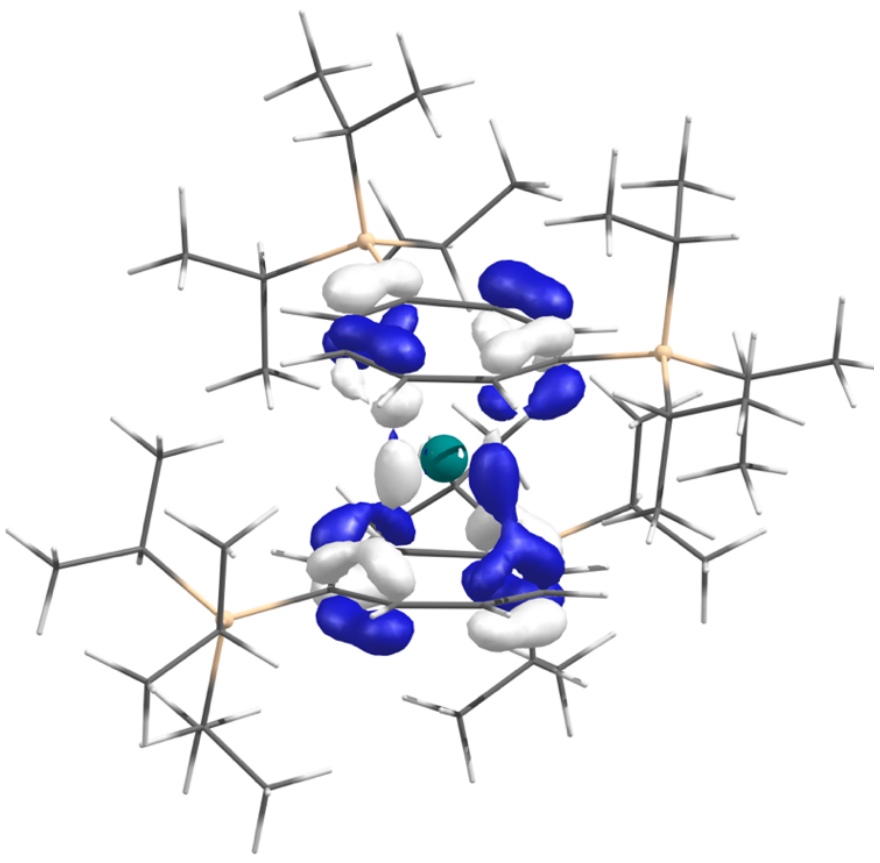

αMO 273 (HOMO–6) –3.04550 eV
U {$6d_{x^2-y^2/xy}$} + COT δ-bond

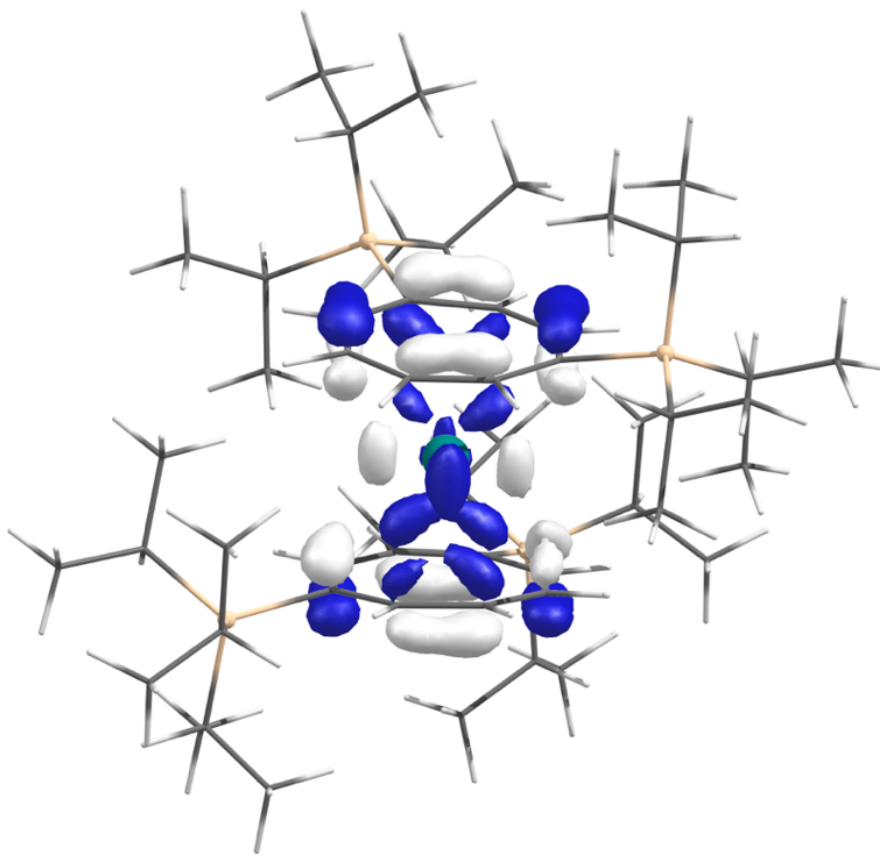

αMO 274 (HOMO–5) –2.98318 eV
U {$6d_{x^2-y^2/xy}$} + COT δ-bond

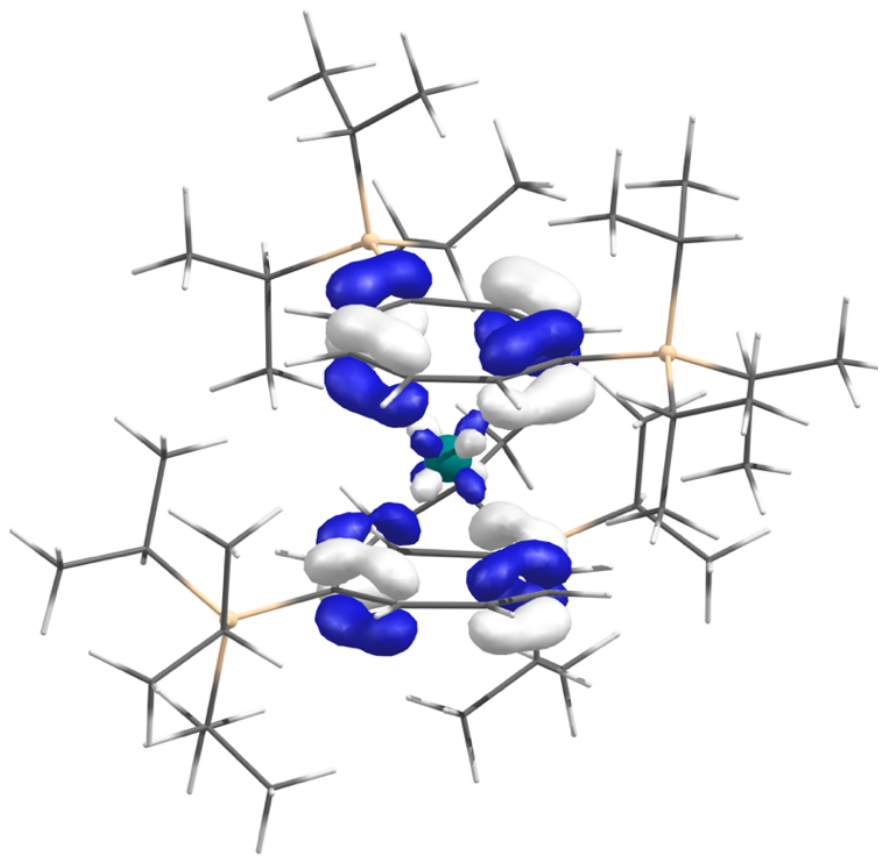

αMO 275 (HOMO–4) –2.23487 eV
U {$5f_{z(x^2-y^2)/xyz}$} + COT δ-bond

Figure S227. Selected MOs and their compositions for **5**. Isosurface values 0.05 e Å^{-3}.

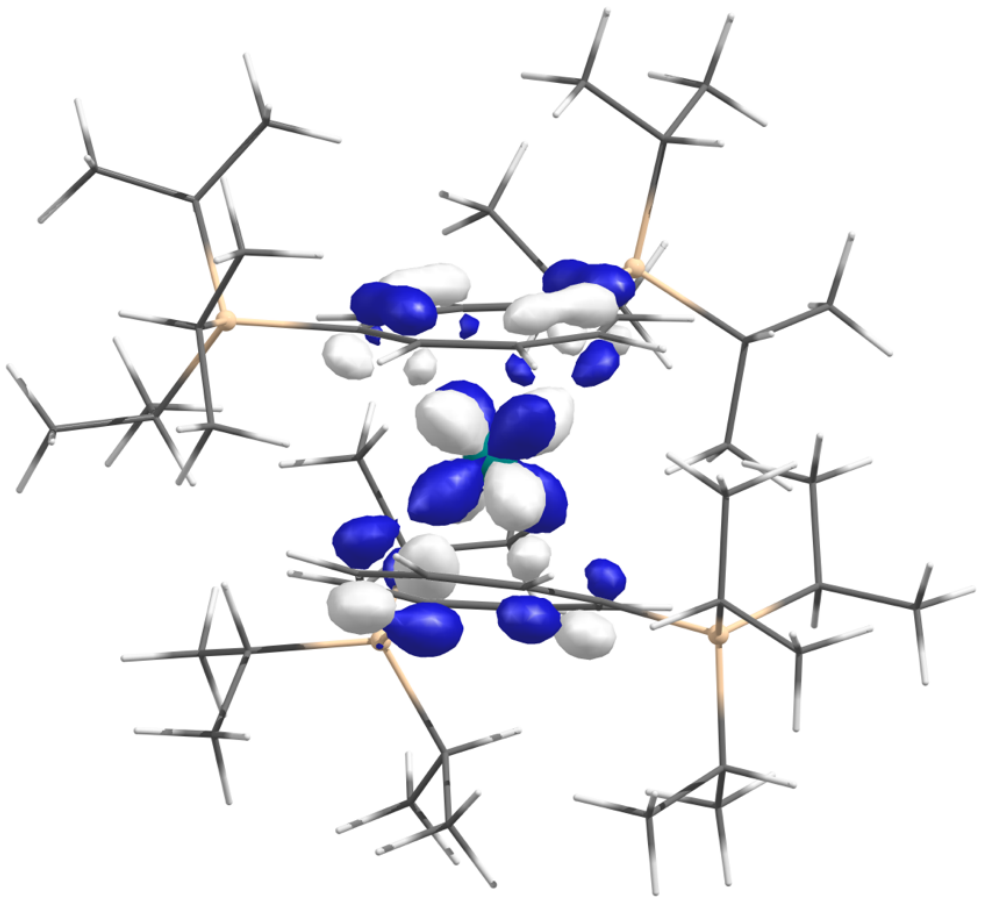

αMO 277 (HOMO) –9.04207 eV
U $\{5f_{z(x^2-y^2)/xyz}\}$ + COT δ-bond

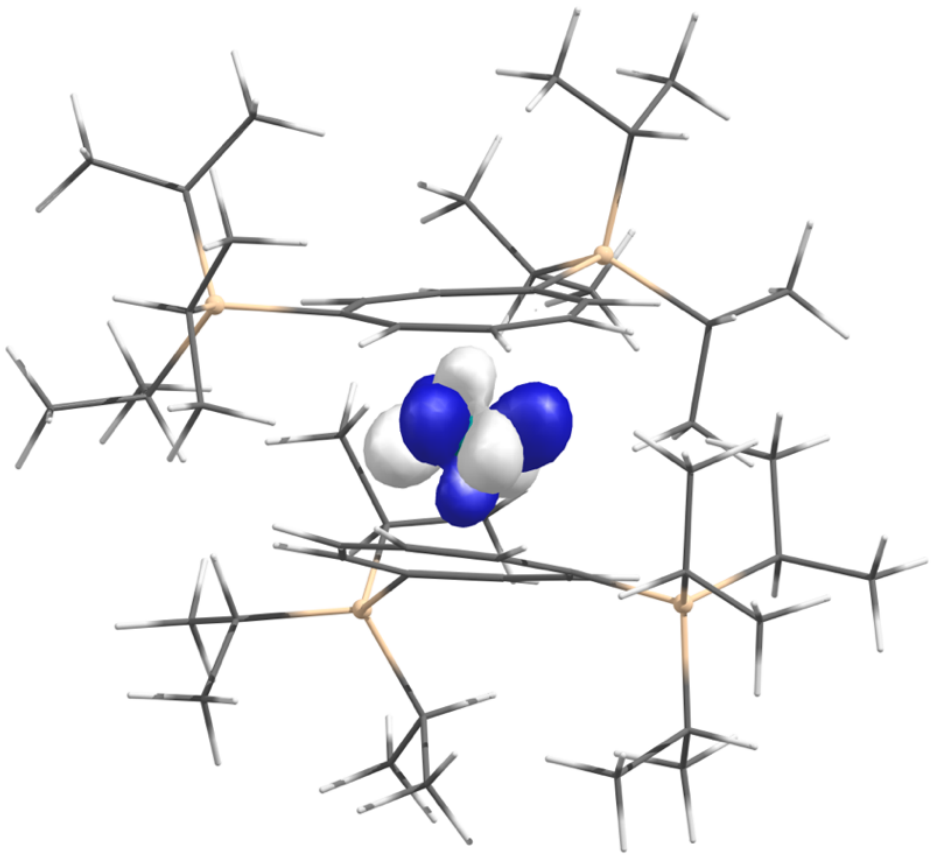

αMO 278 (LUMO) –5.71521 eV
U $\{5f_{x(x^2-3y^2)/y(3x^2-y^2)/z^3}\}$

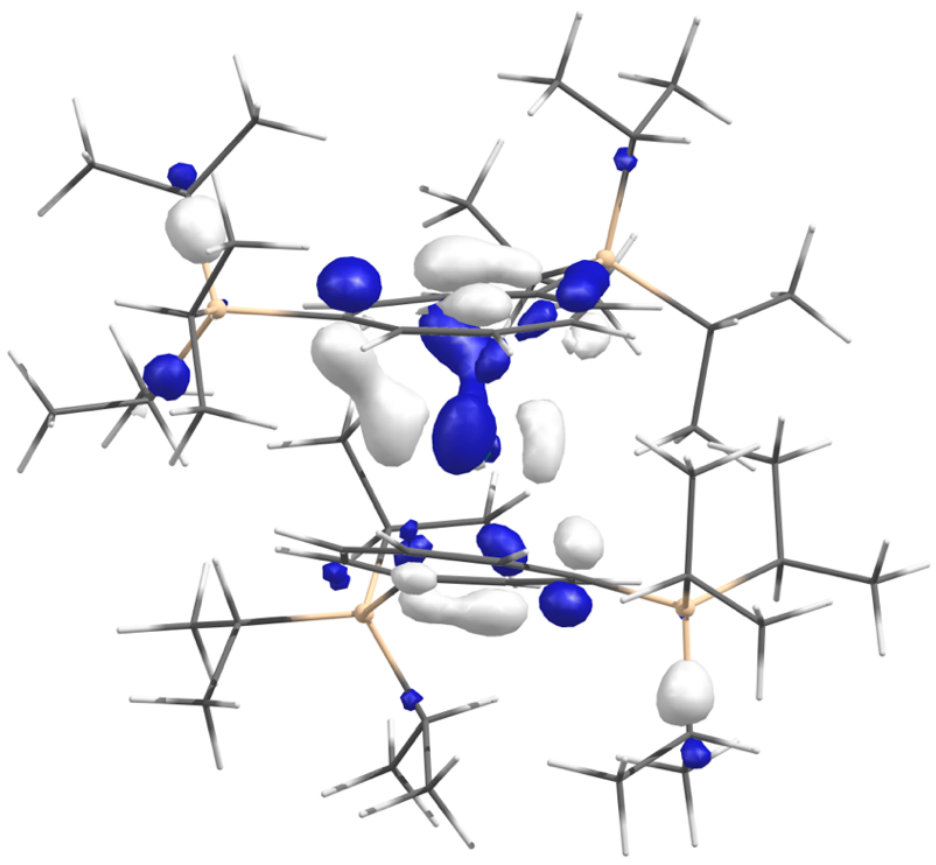

αMO 275 (HOMO–2) –9.31500 eV
U $\{6d_{x^2-y^2/xy}$ + $5f_{z^3}\}$ + COT δ-bond

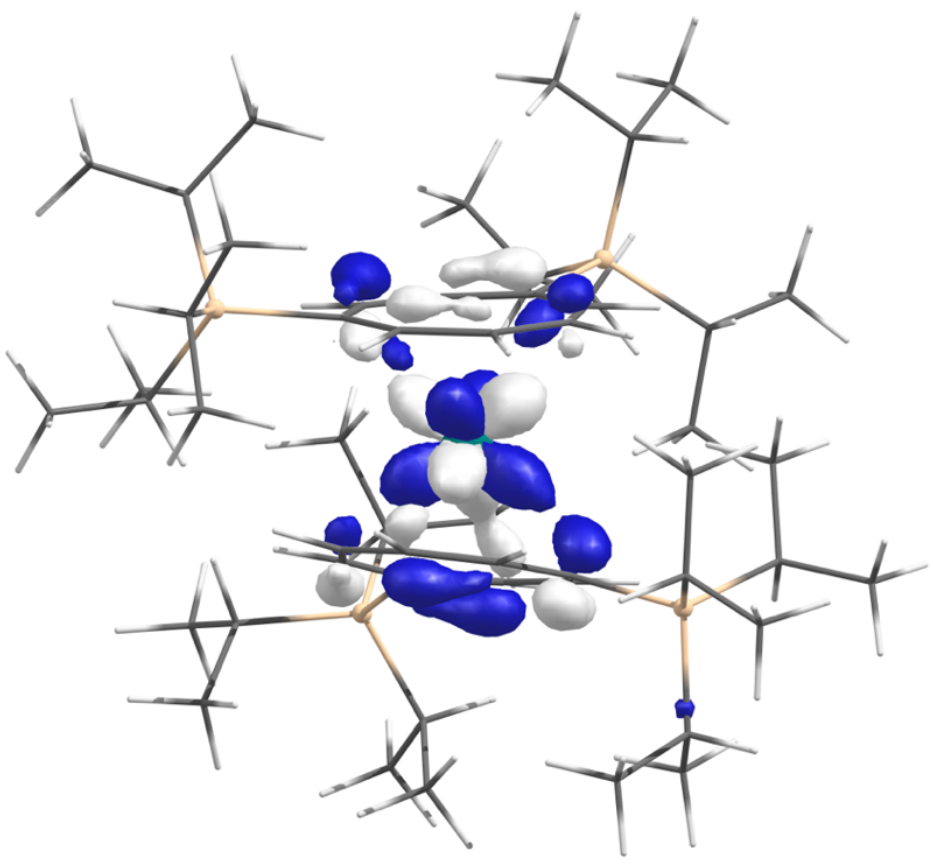

αMO 276 (HOMO–1) –9.07282 eV
U $\{5f_{z(x^2-y^2)/xyz}\}$ + COT δ-bond

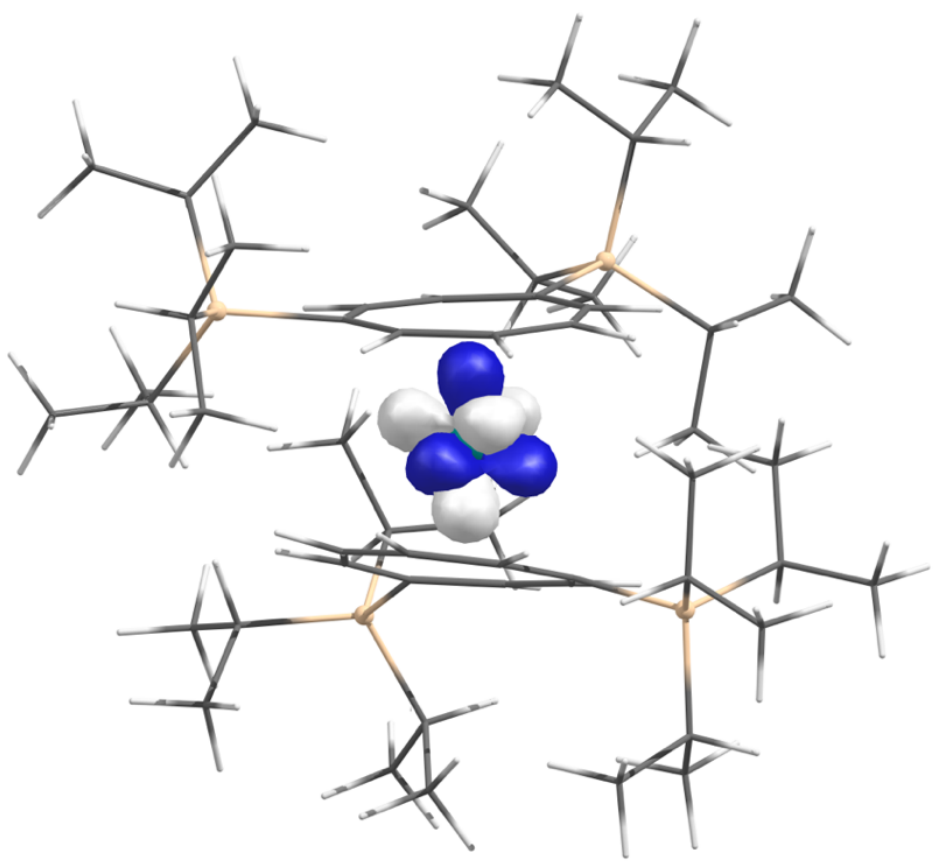

αMO 272 (HOMO–4) –9.75528 eV
U $\{5f_{x(x^2-3y^2)/y(3x^2-y^2)/z^3}\}$

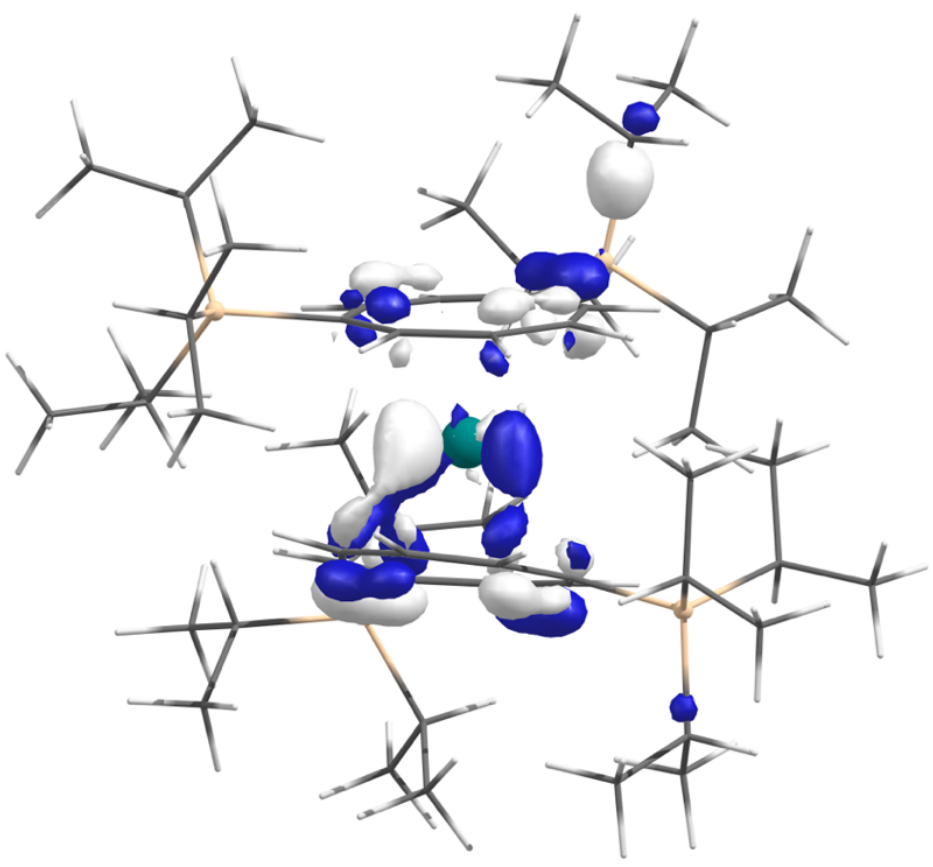

αMO 274 (HOMO–3) –9.36616 eV
U $\{6d_{x^2-y^2/xy}$ + $5f_{z^3}\}$ + COT δ-bond

Figure S228. Selected MOs and their compositions for **6**. Isosurface values 0.05 e Å^{-3}.

Table S41. Löwdin orbital populations for selected orbitals and interactions in **4**.

**Description**	**U 5*d*–COT δ-bond**		**U 5*f*–COT δ-bond**		**5*f* localized**	
**MO#**	**273**<br>HOMO–5	**274**<br>HOMO–4	**277**<br>HOMO–1	**278**<br>HOMO	**275**<br>HOMO–3	**276**<br>HOMO–2
**Energy (eV)**	−6.30706	−6.28774	−5.55139	−5.52609	−5.71766	−5.61262
M *s* (%)	0.0	0.0	0.0	0.0	0.0	0.0
M *p* (%)	0.1	0.0	0.0	0.0	0.4	0.0
M *d* (%)	18.4	18.4	0.0	0.1	0.0	0.1
M *f* (%)	0.1	0.5	25.2	33.5	90.9	85.1
**Total M (%)**	18.6	18.9	25.2	33.6	91.3	85.2

Table S42. Löwdin orbital populations for selected orbitals and interactions in **5**.

**Description**	**U 5*d*–COT δ-bond**		**U 5*f*–COT δ-bond**		**5*f* localized**		
**MO#**	**273**<br>HOMO–6	**274**<br>HOMO–5	**275**<br>HOMO–4	**276**<br>HOMO–3	**277**<br>HOMO–2	**278**<br>HOMO–1	**279**<br>HOMO
**Energy (eV)**	−3.04550	−2.98318	−2.23487	−2.15596	−1.07594	−0.88437	−0.88355
M *s* (%)	0.0	0.0	0.0	0.0	0.0	0.0	0.0
M *p* (%)	0.0	0.0	0.0	0.0	0.0	0.9	0.9
M *d* (%)	18.0	18.5	0.0	0.0	0.0	0.0	0.1
M *f* (%)	0.2	0.0	13.3	14.0	92.8	92.7	92.9
**Total M (%)**	18.2	18.5	13.3	14.0	92.8	93.6	93.9

Table S43. Löwdin orbital populations for selected orbitals and interactions in **6**.

**Description**	**U 5*d*–COT δ-bond**		**U 5*f*–COT δ-bond**		**5*f* localized**
**MO#**	**274**<br>HOMO–3	**275**<br>HOMO–2	**276**<br>HOMO–1	**277**<br>HOMO	**272**<br>HOMO–4
**Energy (eV)**	−9.36616	−9.31500	−9.07282	−9.04207	−9.75528
M *s* (%)	0.0	0.0	0.0	0.0	0.0
M *p* (%)	0.0	0.2	0.1	0.1	0.3
M *d* (%)	15.1	15.1	0.6	0.2	0.5
M *f* (%)	1.9	1.3	42.2	41.0	75.4
**Total M (%)**	17.0	16.6	42.9	41.3	76.2

Table S44. A comparison of Löwdin orbital populations for selected orbitals and interactions for H-atom optimized coordinates for **4**–**6**.

**Description**	**U 5*d*–COT δ-bond**			**U 5*f*–COT δ-bond**			**5*f* localized**		
	**4 U(IV)**	**5U U(III)**	**6 U(V)**	**4 U(IV)**	**5U U(III)**	**6 U(V)**	**4 U(IV)**	**5U U(III)**	**6 U(V)**
**Energy (eV)**	−6.30706<br>−6.28774	−3.04550<br>−2.98318	−9.36616<br>−9.31500	−5.55139<br>−5.52609	−2.23487<br>−2.15596	−9.07282<br>−9.04207	−5.71766<br>−5.61262	−1.07594<br>−0.88437<br>−0.88355	−9.75528
M *s* (%)	0.0<br>0.0	0.0<br>0.0	0.0<br>0.0	0.0<br>0.0	0.0<br>0.0	0.0<br>0.0	0.0<br>0.0	0.0<br>0.0<br>0.0	0.0
M *p* (%)	0.1<br>0.0	0.0<br>0.0	0.0<br>0.0	0.0<br>0.0	0.0<br>0.0	0.1<br>0.1	0.4<br>0.0	0.0<br>0.9<br>0.9	0.3
M *d* (%)	18.4<br>18.4	18.0<br>18.5	15.1<br>15.1	0.0<br>0.1	0.0<br>0.0	0.6<br>0.2	0.0<br>0.1	0.0<br>0.0<br>0.1	0.5
M *f* (%)	0.1<br>0.5	0.2<br>0.0	1.9<br>1.3	25.2<br>33.5	13.3<br>14.0	42.2<br>41.0	90.9<br>85.1	92.8<br>92.7<br>92.9	75.4
**Total M (%)**	18.6<br>18.9	18.2<br>18.5	17.0<br>16.6	25.2<br>33.5	13.3<br>14.0	42.9<br>41.3	91.3<br>85.2	92.8<br>93.6<br>93.9	76.2

Natural bonding molecular orbital compositions and visualizations

Table S45. NLMO compositions for selected orbitals of **1** with metal content >4%.

NLMO#	M (%)					COT C8 total (%)	Description
	Total	*s*	*p*	*d*	*f*		
**60**	99.481	0.00	0.00	0.00	100.00	0.00	U {5f_{z3}}
**61**	98.041	0.00	0.00	0.00	100.00	0.00	U {5$f_{y(3x2-y2)}$}
**62**	34.226	0.00	0.00	0.00	100.00	65.749	(U 5*f*) + (COT π) δ
**63**	18.344	0.00	0.00	98.99	0.99	81.361	(U 6*d*) + (COT π) δ
**66**	10.617	4.11	0.13	42.43	53.33	87.039	(U 5*f* / 6*d*) + (COT π)
**69**	8.203	7.24	0.20	44.93	47.63	90.914	(U 5*f* / 6*d*) + (COT π)
**74**	10.400	5.04	0.14	45.35	49.46	86.715	(U 5*f* / 6*d*) + (COT π)
**79**	7.620	6.41	0.21	46.52	46.86	91.396	(U 5*f* / 6*d*) + (COT π)
**86**	10.708	3.88	0.13	41.02	54.96	87.623	(U 5*f* / 6*d*) + (COT π)
**89**	8.205	7.08	0.20	43.55	49.17	90.947	(U 5*f* / 6*d*) + (COT π)
**94**	10.464	4.98	0.14	44.73	50.15	88.858	(U 5*f* / 6*d*) + (COT π)
**99**	7.630	6.39	0.21	46.76	46.64	91.420	(U 5*f* / 6*d*) + (COT π)

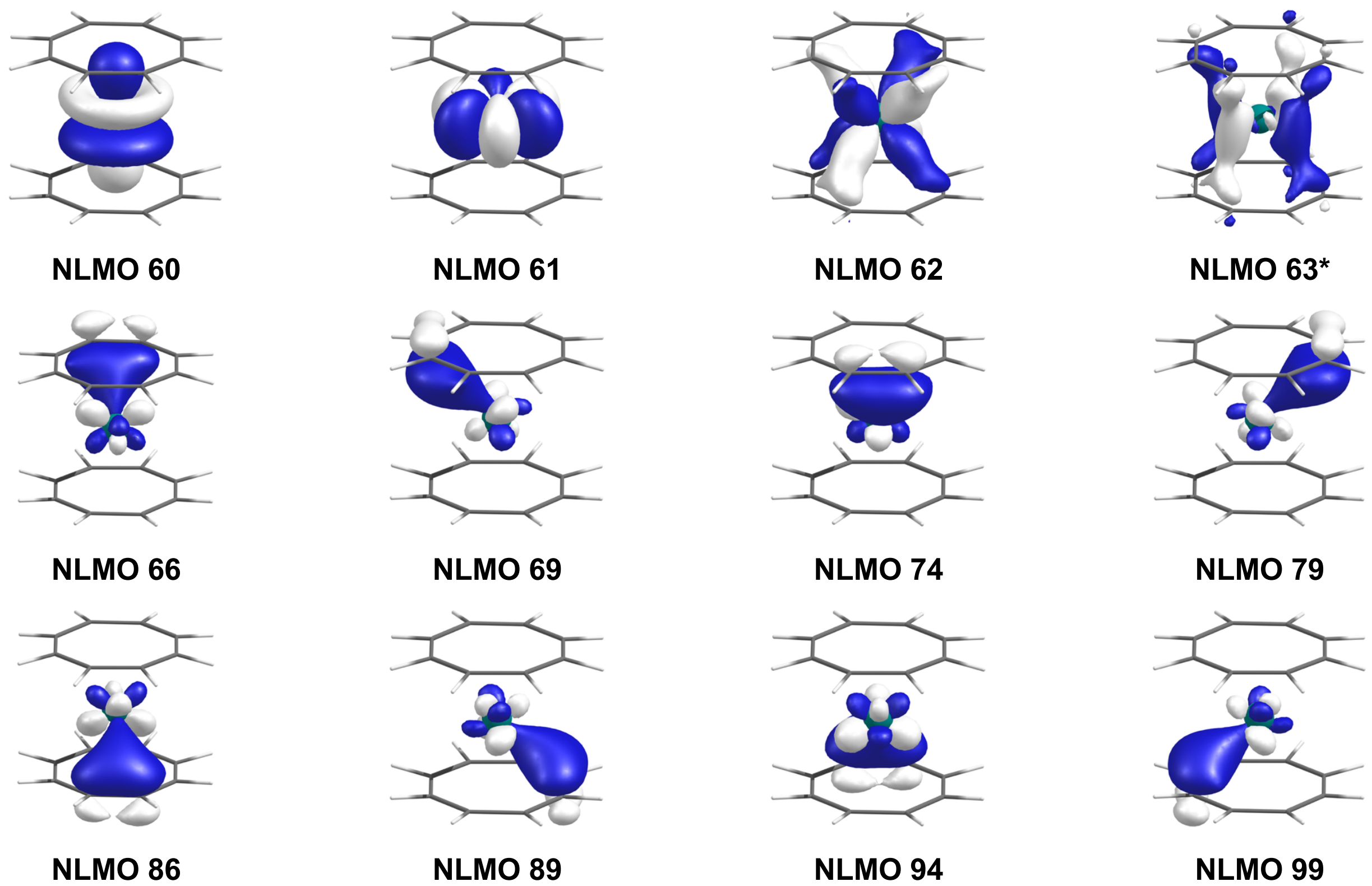

Figure S229. Selected NLMO isosurfaces for **1**. Isosurface values 0.10 e Å^{-3}.* NLMO 63 shown at 0.08 e Å^{-3} for clarity.

Table S46. NLMO compositions for selected orbitals of **2** with metal content >4%.

**NLMO#**	**M (%)**					**COT C_8 total (%)**	**Description**
	**Total**	***s***	***p***	***d***	***f***		
**60**	99.053	0.00	2.25	0.02	97.72	0.00	U {$5f_{xz2}$}
**61**	98.878	0.03	2.18	0.11	97.68	0.00	U {$5f_{yz2}$}
**62**	95.042	0.00	0.00	0.07	99.93	0.00	U {$5f_{y(3x2-y2)}$}
**63**	10.527	1.41	0.74	60.73	37.11	84.904	(U 5*f* / 6*d*) + (COT π)
**64**	12.163	1.86	0.43	71.51	26.19	87.136	(U 5*f* / 6*d*) + (COT π)
**65**	10.388	3.88	0.68	11.92	83.52	87.468	(U 5*f* / 6*d*) + (COT π)
**66**	11.371	0.97	0.41	45.32	53.30	88.163	(U 5*f* / 6*d*) + (COT π)
**69**	7.081	7.13	1.46	42.38	49.02	92.057	(U 5*f* / 6*d*) + (COT π)
**83**	6.208	8.21	1.49	45.11	45.19	92.119	(U 5*f* / 6*d*) + (COT π)
**87**	5.714	7.74	2.06	57.63	32.56	93.756	(U 5*f* / 6*d*) + (COT π)
**93**	6.552	7.74	1.32	54.32	36.61	91.408	(U 5*f* / 6*d*) + (COT π)
**98**	6.792	6.99	1.13	56.23	35.64	92.103	(U 5*f* / 6*d*) + (COT π)
**103**	5.707	6.43	1.76	57.58	34.23	93.665	(U 5*f* / 6*d*) + (COT π)

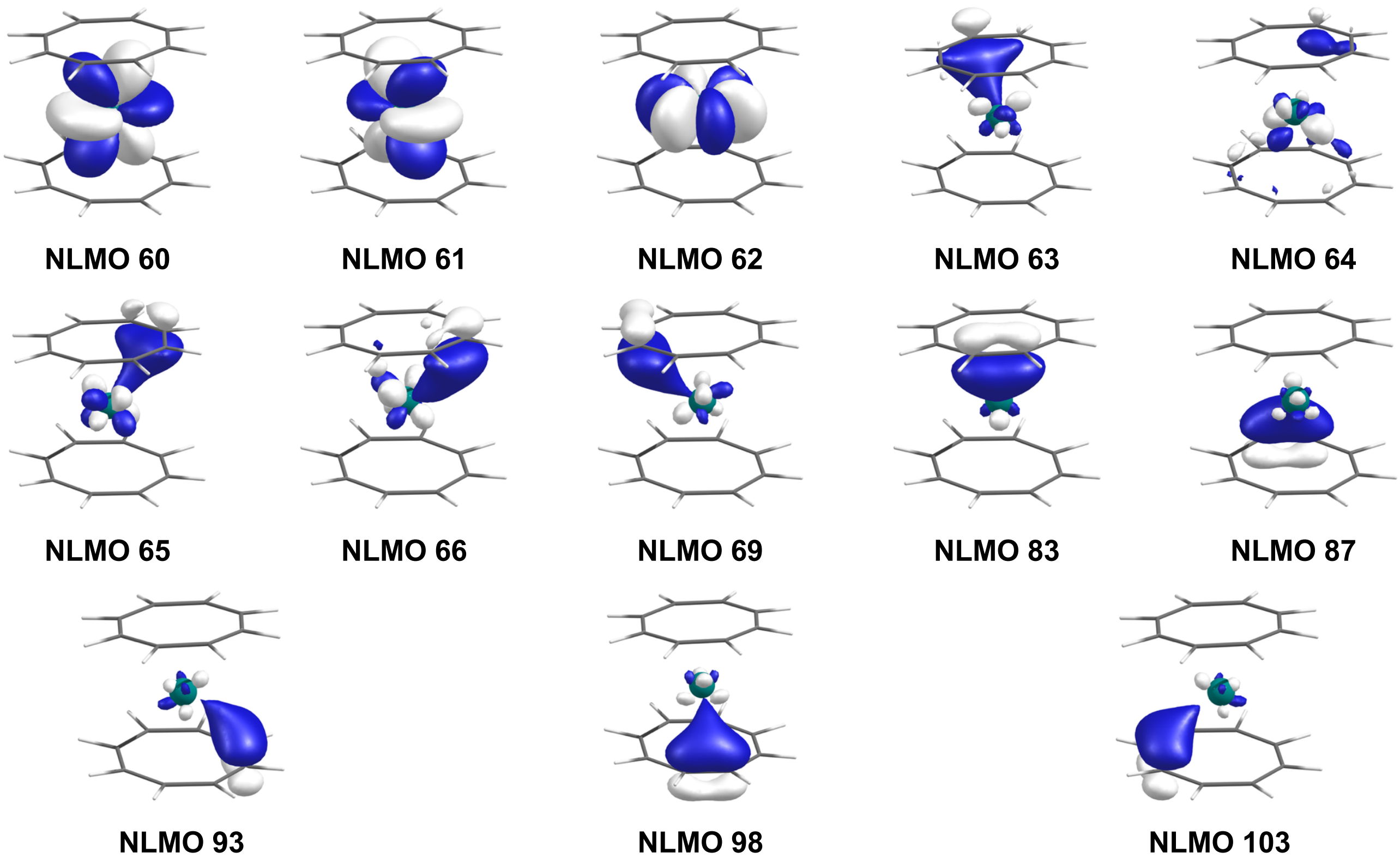

Figure S230. Selected NLMO isosurfaces for **2**. Isosurface values 0.10 e Å^{-3}.

Table S47. NLMO compositions for selected orbitals of **3** with metal content >4%.

NLMO#	M (%)					COT C_8 total (%)	Description
	Total	*s*	*p*	*d*	*f*		
**60**	98.968	0.00	0.01	0.01	99.98	0.00	U {$5f_{xyz}$}
**61**	45.855	0.05	0.00	11.22	88.73	52.199	(U 5*f* / 6*d*) + (COT π) δ
**62**	40.978	0.13	0.00	19.36	80.50	56.101	(U 5*f* / 6*d*) + (COT π) δ
**66**	13.435	5.19	0.10	49.85	44.85	83.094	(U 5*f* / 6*d*) + (COT π)
**69**	9.910	4.72	0.20	49.08	45.99	87.447	(U 5*f* / 6*d*) + (COT π)
**74**	11.614	4.67	0.11	51.08	44.14	85.841	(U 5*f* / 6*d*) + (COT π)
**79**	11.632	4.19	0.14	49.89	45.77	85.161	(U 5*f* / 6*d*) + (COT π)
**85**	11.763	5.23	0.11	52.15	42.49	85.339	(U 5*f* / 6*d*) + (COT π)
**91**	11.223	4.26	0.10	55.25	40.38	85.698	(U 5*f* / 6*d*) + (COT π)
**96**	10.323	5.04	0.12	57.63	37.19	86.225	(U 5*f* / 6*d*) + (COT π)
**101**	12.152	4.48	0.11	52.05	43.36	84.532	(U 5*f* / 6*d*) + (COT π)

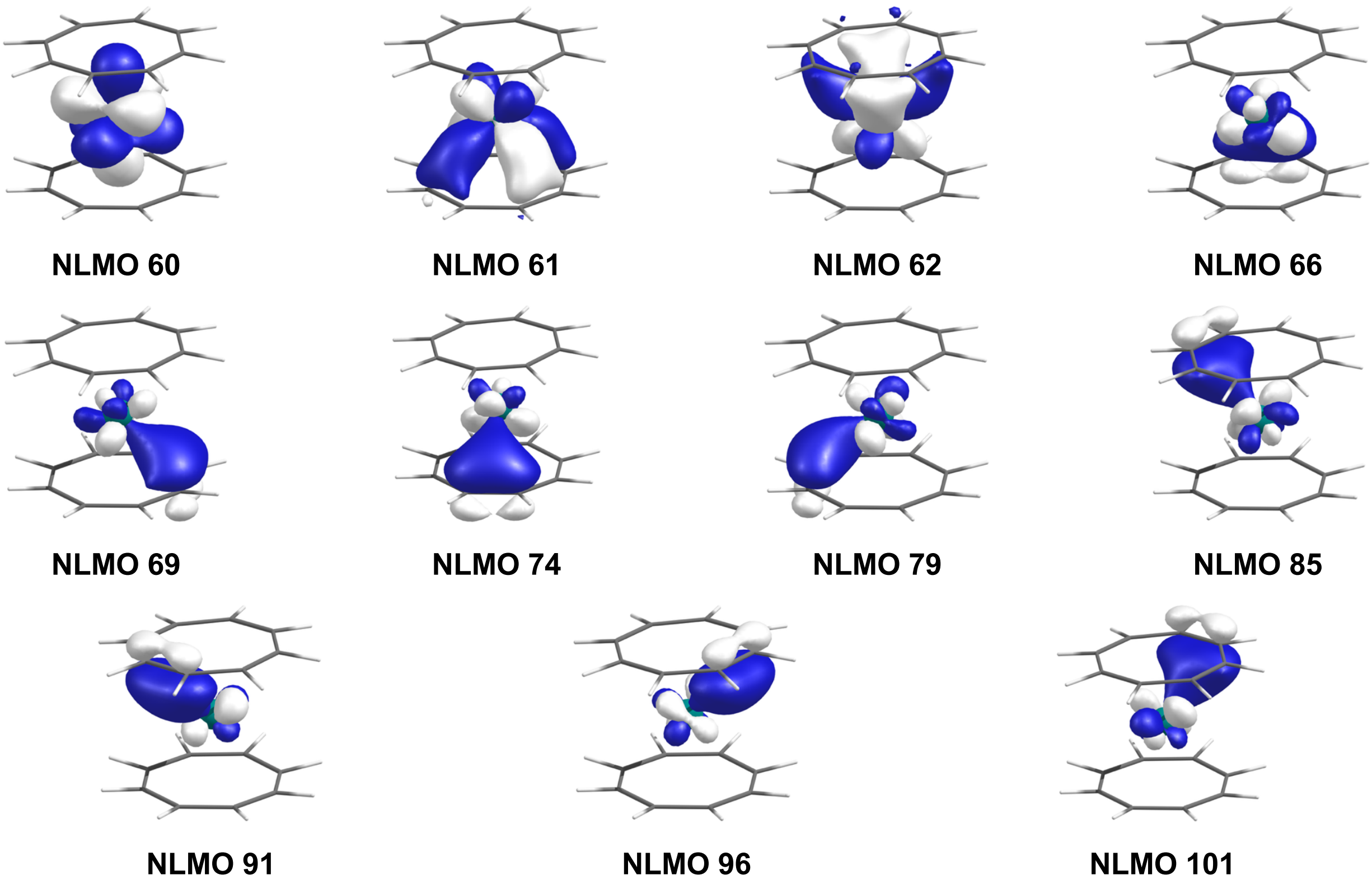

Figure S231. Selected NLMO isosurfaces for **3**. Isosurface values 0.10 e Å^{-3}.

*Symmetrized complexes **1–3***

Table S48. NLMO compositions for selected orbitals of **1-D8h** with metal content >4%.

NLMO#	M (%)					COT C_8 total (%)	Description
	Total	*s*	*p*	*d*	*f*		
**60**	98.727	0.00	1.43	0.00	98.57	0.00	U {5f_{xyz}}
**61**	98.722	0.00	1.42	0.00	98.58	0.00	U {5$f_{z(x2-y2)}$}
**62**	34.696	0.00	0.00	0.02	99.98	64.811	(U 5*f*) + (COT π) δ
**63**	18.577	0.00	0.00	99.72	0.26	77.692	(U 6*d*) + (COT π) δ
**66**	9.392	5.84	0.65	46.28	47.23	88.054	(U 5*f* / 6*d*) + (COT π)
**69**	9.407	5.83	0.64	46.21	47.32	88.043	(U 5*f* / 6*d*) + (COT π)
**74**	9.392	5.84	0.65	46.28	47.23	88.055	(U 5*f* / 6*d*) + (COT π)
**79**	9.407	5.83	0.64	46.21	47.32	88.043	(U 5*f* / 6*d*) + (COT π)
**86**	9.390	5.84	0.65	46.27	47.24	88.309	(U 5*f* / 6*d*) + (COT π)
**89**	9.405	5.83	0.64	46.20	47.33	88.297	(U 5*f* / 6*d*) + (COT π)
**94**	9.390	5.84	0.65	46.27	47.24	88.309	(U 5*f* / 6*d*) + (COT π)
**99**	9.405	5.83	0.64	46.20	47.33	88.477	(U 5*f* / 6*d*) + (COT π)

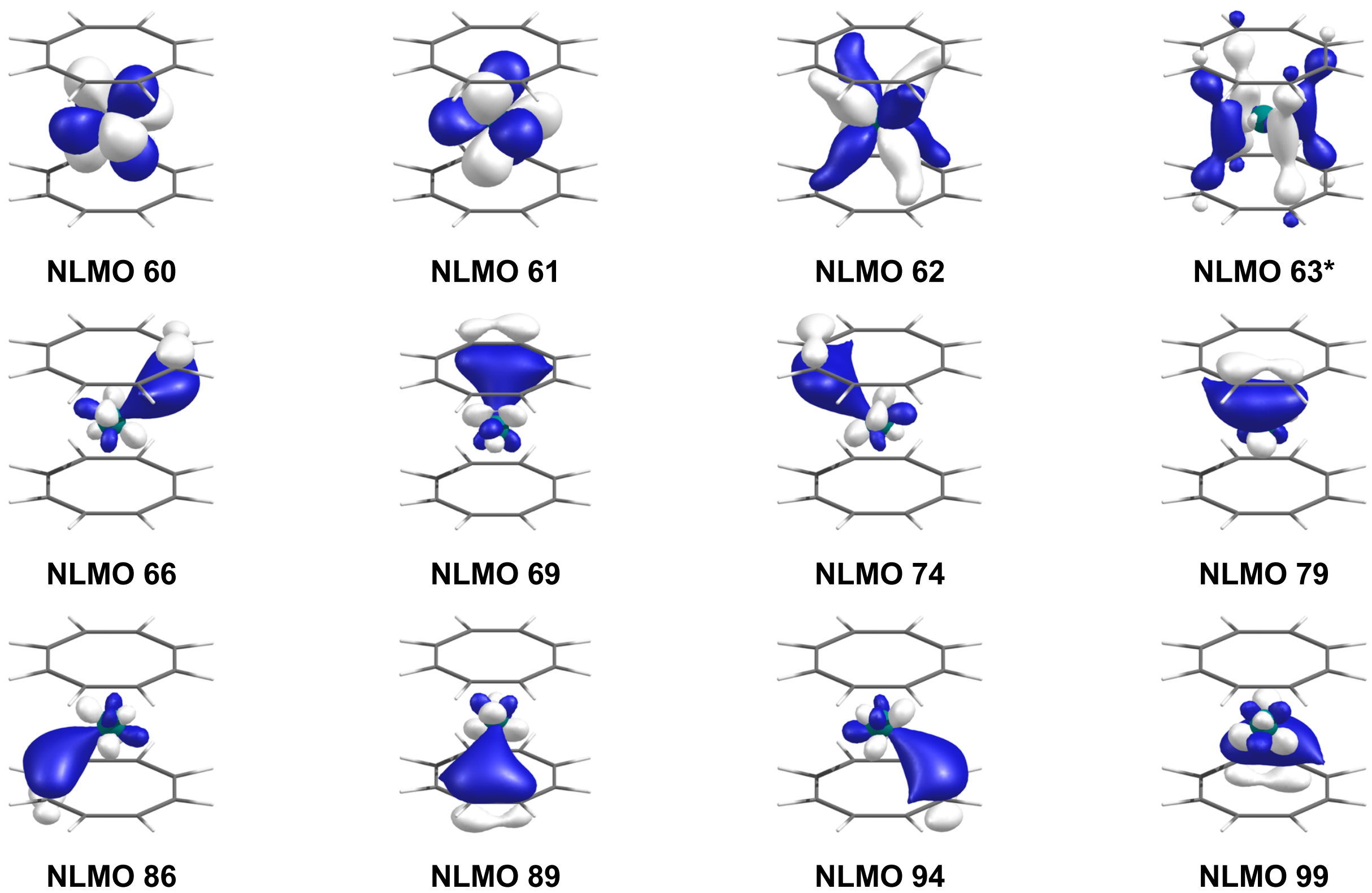

Figure S232. Selected NLMO isosurfaces for **1-D8h**. Isosurface values 0.10 e Å^{-3}. * NLMO 63 shown at 0.08 e Å^{-3} for clarity.

Table S49. NLMO compositions for selected orbitals of **2-C2v** with metal content >4%.

NLMO#	M (%)					COT C_8 total (%)	Description
	Total	*s*	*p*	*d*	*f*		
**60**	98.874	0.00	0.00	0.13	99.87	0.00	U {5f_{xyz}}
**61**	98.855	0.00	1.08	0.01	98.90	0.00	U {5f_{xz2}}
**62**	95.199	0.00	0.00	0.02	99.98	0.00	U {5$f_{y(3x2-y2)}$}
**63**	11.244	1.65	0.32	56.13	41.89	88.607	(U 5*f* / 6*d*) + (COT π)
**64**	11.845	2.22	0.35	48.38	49.05	88.026	(U 5*f* / 6*d*) + (COT π)
**65**	11.244	1.65	0.32	56.13	41.89	88.607	(U 5*f* / 6*d*) + (COT π)
**66**	11.845	2.22	0.35	48.38	49.05	88.026	(U 5*f* / 6*d*) + (COT π)
**69**	5.683	8.65	1.39	52.85	37.11	93.032	(U 5*f* / 6*d*) + (COT π)
**79**	6.540	8.69	1.00	48.61	41.69	92.147	(U 5*f* / 6*d*) + (COT π)
**84**	5.572	6.62	0.83	56.75	35.81	94.220	(U 5*f* / 6*d*) + (COT π)
**91**	5.683	8.65	1.39	52.85	37.11	93.032	(U 5*f* / 6*d*) + (COT π)
**96**	5.572	6.62	0.83	56.75	35.81	94.220	(U 5*f* / 6*d*) + (COT π)
**101**	6.540	8.69	1.00	48.61	41.69	92.147	(U 5*f* / 6*d*) + (COT π)

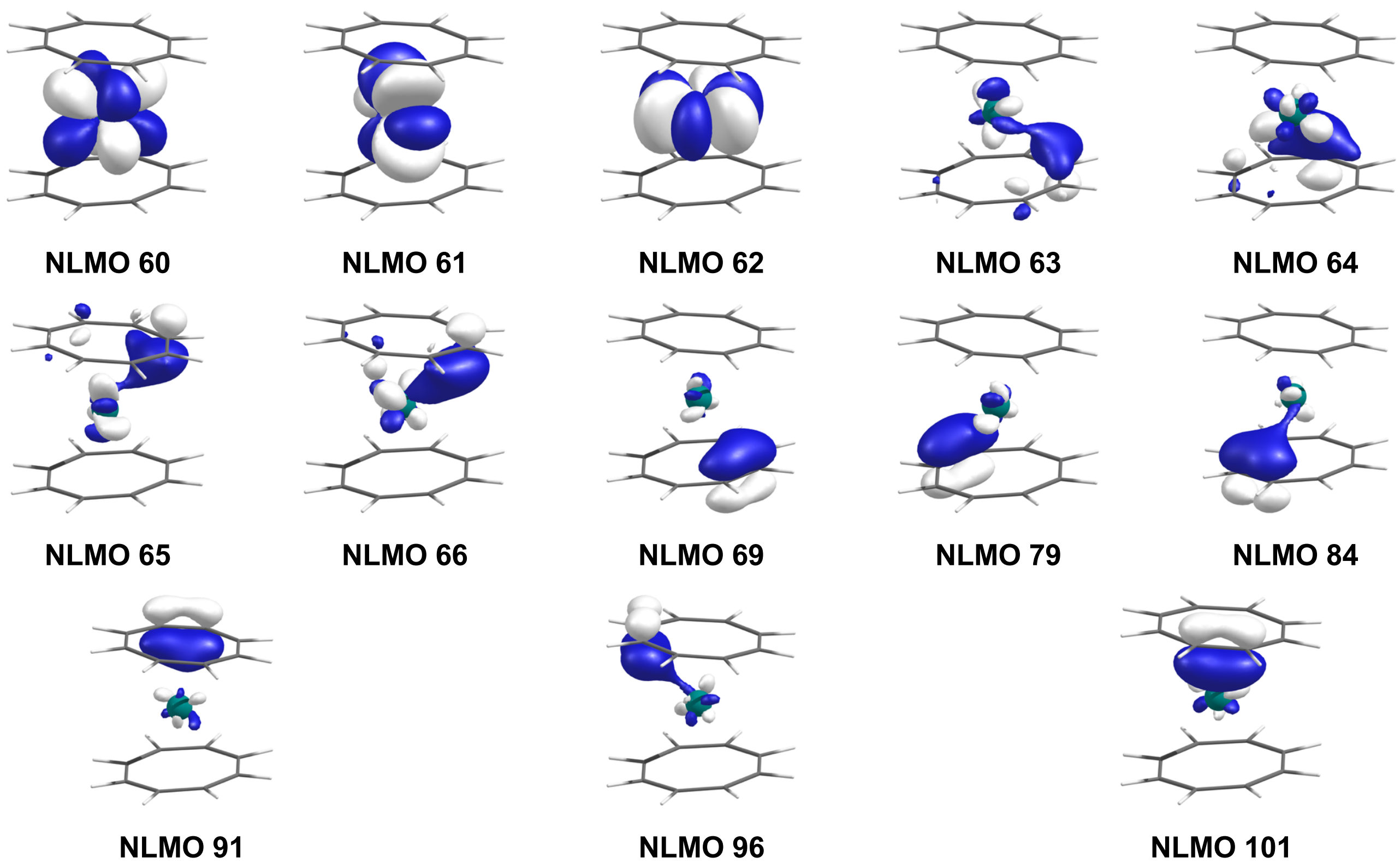

Figure S233. Selected NLMO isosurfaces for **2-C2v**. Isosurface values 0.10 e Å^{-3}.

Table S50. NLMO compositions for selected orbitals of **3-C2v** with metal content >4%.

NLMO#	M (%)					COT C_8 total (%)	Description
	Total	*s*	*p*	*d*	*f*		
**60**	97.966	0.00	0.31	0.01	99.68	0.00	U {$5f_{y(3x2-y2)}$}
**61**	57.421	0.00	0.00	0.11	99.89	37.811	(U 5*f*) + (COT π)
**62**	22.108	0.01	0.00	97.07	2.91	75.973	(U 6*d*) + (COT π) δ
**64**	13.685	3.23	0.11	42.61	54.05	84.926	(U 5*f* / 6*d*) + (COT π)
**70**	11.743	5.40	0.24	36.29	58.06	86.090	(U 5*f* / 6*d*) + (COT π)
**75**	16.460	3.17	0.11	34.15	62.57	81.133	(U 5*f* / 6*d*) + (COT π)
**80**	11.234	6.13	0.18	47.31	46.37	87.584	(U 5*f* / 6*d*) + (COT π)
**84**	11.261	6.09	0.18	47.43	46.30	87.124	(U 5*f* / 6*d*) + (COT π)
**90**	16.433	3.20	0.11	34.04	62.65	81.554	(U 5*f* / 6*d*) + (COT π)
**95**	11.767	5.36	0.24	36.51	57.88	86.391	(U 5*f* / 6*d*) + (COT π)
**100**	13.627	3.27	0.11	42.30	54.32	84.951	(U 5*f* / 6*d*) + (COT π)

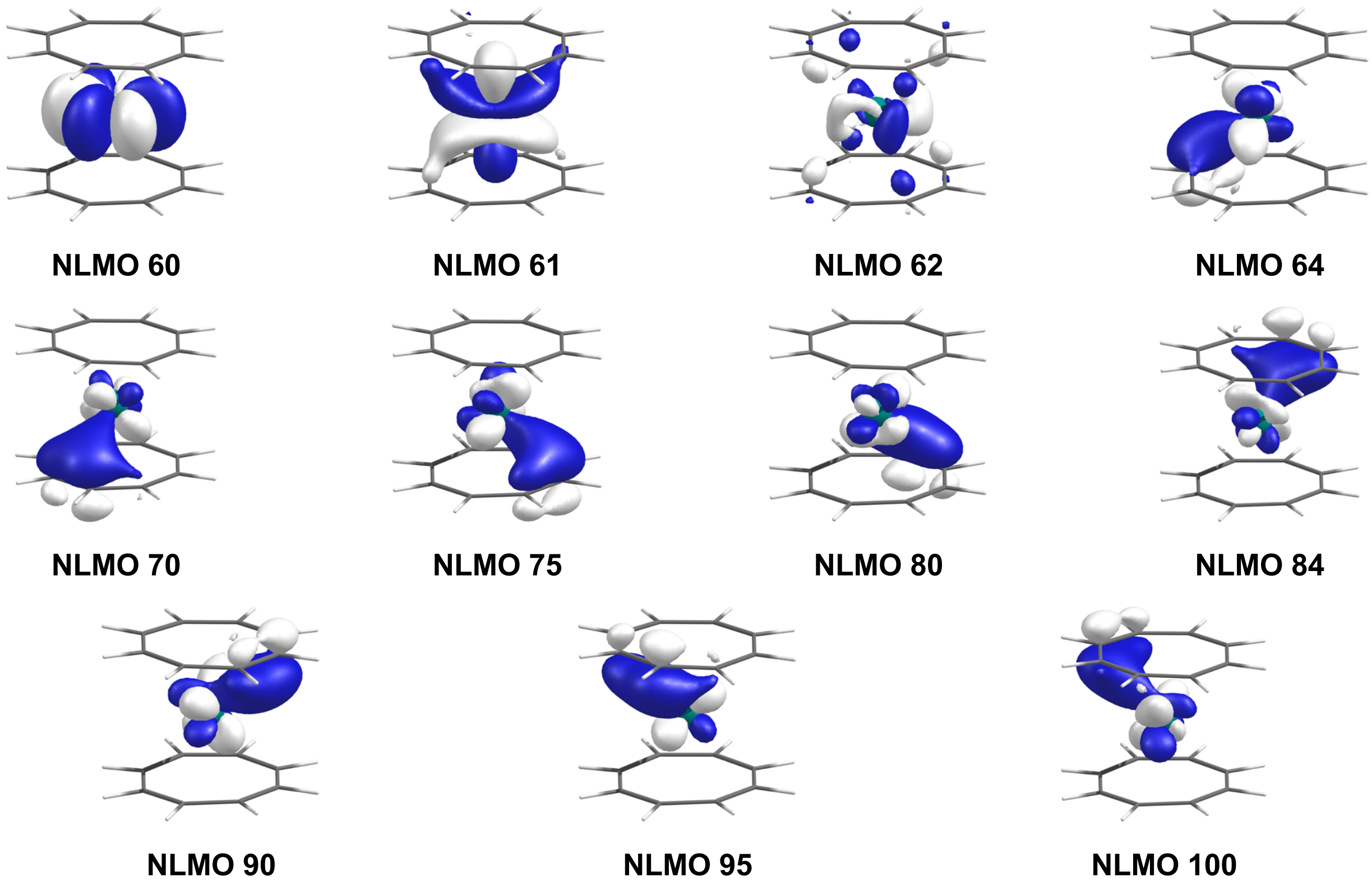

Figure S234. Selected NLMO isosurfaces for **3-C2v**. Isosurface values 0.10 e Å^{-3}.

Table S51. NLMO compositions for selected orbitals of **3-D8d** with metal content >4%.

NLMO#	M (%)					COT C_8 total (%)	Description
	Total	*s*	*p*	*d*	*f*		
**60**	98.158	0.00	0.00	0.00	100.00	0.00	U $\{5f_{y(3x2-y2)}\}$
**61**	55.410	0.07	0.00	1.04	98.89	39.299	(U 5*f*) + (COT π) δ
**62**	31.223	0.00	0.00	49.41	50.58	60.234	(U 5*f* / 6*d*) + (COT π) δ
**64**	13.441	3.16	0.07	49.64	47.12	83.809	(U 5*f* / 6*d*) + (COT π)
**70**	12.247	6.07	0.09	39.89	53.95	85.678	(U 5*f* / 6*d*) + (COT π)
**75**	13.441	3.16	0.07	49.64	47.12	83.809	(U 5*f* / 6*d*) + (COT π)
**80**	12.247	6.07	0.09	39.89	53.95	85.678	(U 5*f* / 6*d*) + (COT π)
**84**	12.247	6.07	0.09	39.89	53.95	85.678	(U 5*f* / 6*d*) + (COT π)
**90**	13.441	3.16	0.07	49.64	47.12	83.809	(U 5*f* / 6*d*) + (COT π)
**95**	12.247	6.07	0.09	39.89	53.95	85.678	(U 5*f* / 6*d*) + (COT π)
**100**	13.441	3.16	0.07	49.64	47.12	83.809	(U 5*f* / 6*d*) + (COT π)

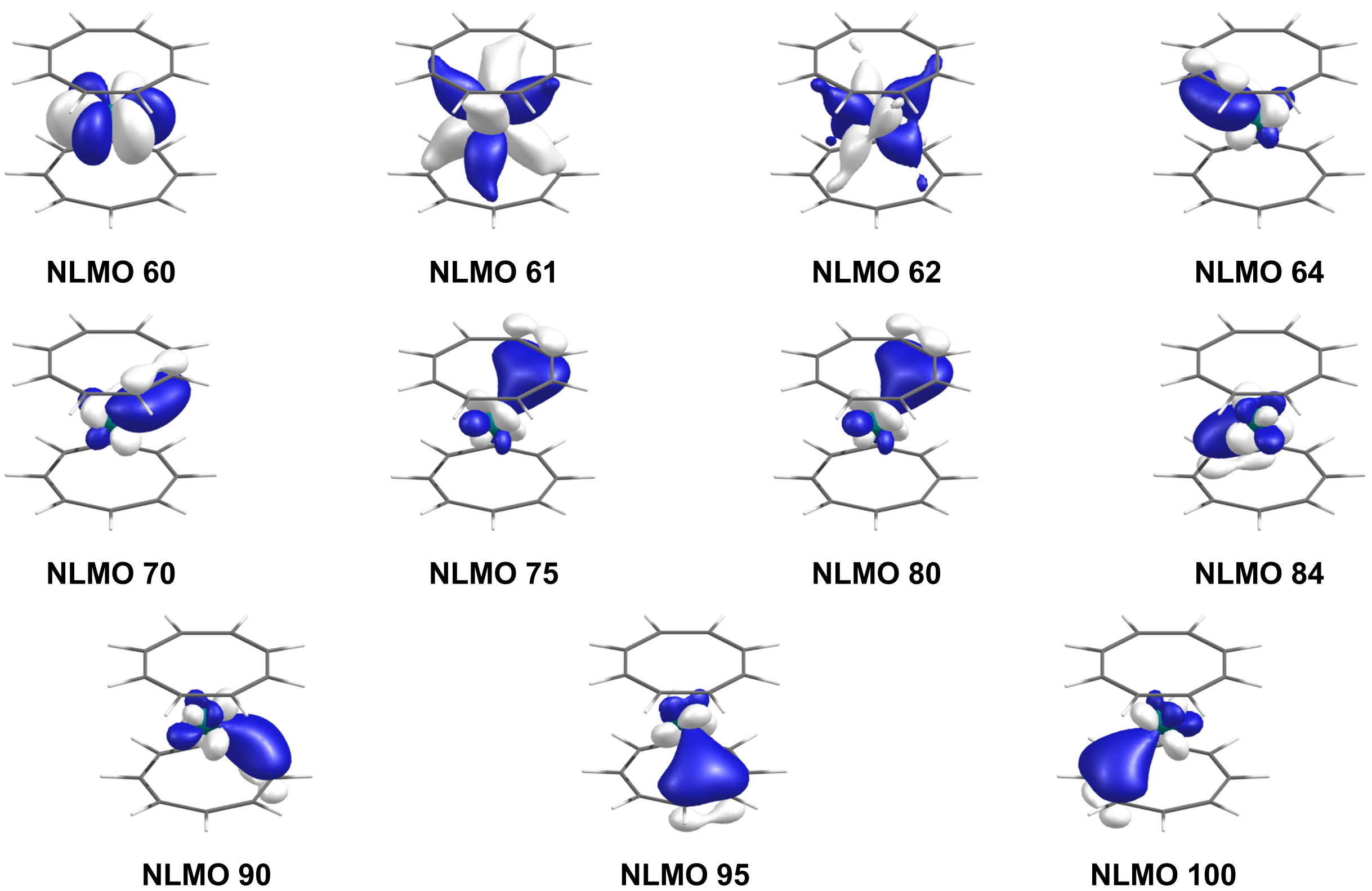

Figure S235. Selected NLMO isosurfaces for **3-D8d**. Isosurface values 0.10 e Å^{-3}.

*Complexes **4**–**6***

Table S52. NLMO compositions for selected orbitals of **4** with metal content >4%.

**NLMO#**	**M (%)**					**COT C_8 total (%)**	**Description**
	**Total**	***s***	***p***	***d***	***f***		
**116**	99.536	0.00	0.00	0.06	99.94	0.00	U {5f_{z3}}
**117**	97.601	0.00	0.00	0.04	99.96	0.00	U {5$f_{y(3x2-y2)}$}
**118**	25.495	0.17	0.01	28.97	70.84	73.578	(U 5*f* / 6*d*) + (COT π) δ
**119**	24.061	0.00	0.01	38.57	61.41	73.420	(U 5*f* / 6*d*) + (COT π) δ
**122**	9.596	4.74	0.36	30.73	64.16	88.539	(U 5*f* / 6*d*) + (COT π)
**126**	9.062	3.81	0.33	34.45	61.40	89.637	(U 5*f* / 6*d*) + (COT π)
**130**	8.171	5.59	0.42	34.78	59.20	89.013	(U 5*f* / 6*d*) + (COT π)
**136**	10.782	4.01	0.15	32.17	63.67	88.014	(U 5*f* / 6*d*) + (COT π)
**142**	8.102	5.22	0.43	48.56	45.78	89.481	(U 5*f* / 6*d*) + (COT π)
**146**	8.471	4.01	0.36	50.52	45.10	90.401	(U 5*f* / 6*d*) + (COT π)
**150**	7.318	5.87	0.47	52.08	41.57	90.353	(U 5*f* / 6*d*) + (COT π)
**156**	9.816	4.18	0.16	52.30	43.35	86.668	(U 5*f* / 6*d*) + (COT π)

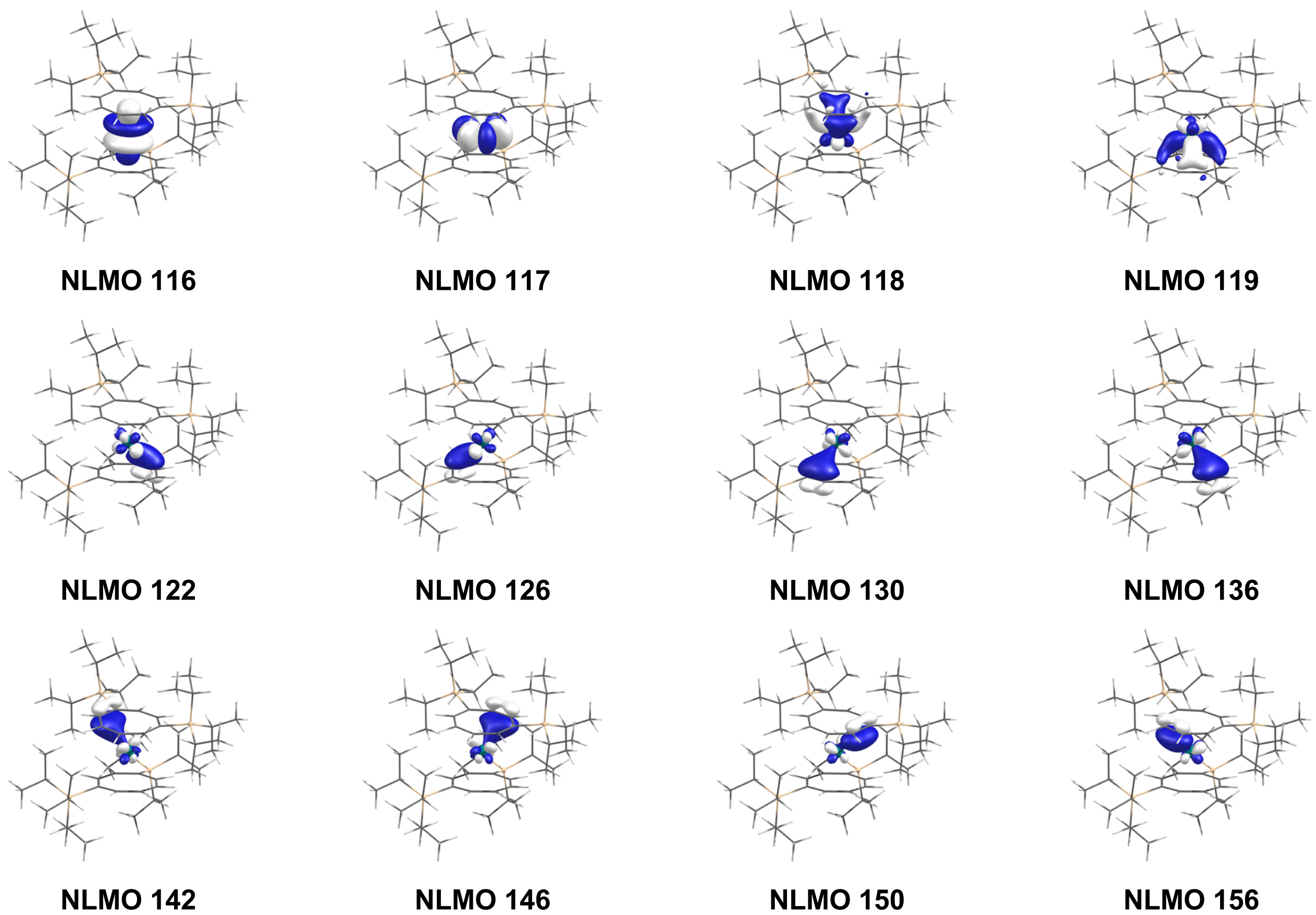

Figure S236. Selected NLMO isosurfaces for **4**. Isosurface values 0.10 e Å^{-3}.

Table S53. NLMO compositions for selected orbitals of **5** with metal content >4%.

NLMO#	M (%)					COT C_8 total (%)	Description
	Total	*s*	*p*	*d*	*f*		
**116**	98.920	0.00	1.62	0.06	98.32	0.00	U {5f_{xz2}}
**117**	98.858	0.01	1.70	0.07	98.21	0.00	U {5f_{yz2}}
**118**	95.209	0.00	0.00	0.01	99.98	0.00	U {5$f_{y(3x2-y2)}$}
**119**	10.759	2.13	0.57	61.48	35.81	85.451	(U 5*f* / 6*d*) + (COT π)
**120**	10.017	1.18	1.13	35.13	62.56	86.881	(U 5*f* / 6*d*) + (COT π)
**121**	10.755	1.36	0.63	37.40	60.61	86.400	(U 5*f* / 6*d*) + (COT π)
**122**	10.509	2.24	0.72	64.98	32.05	86.518	(U 5*f* / 6*d*) + (COT π)
**128**	5.188	6.05	2.46	54.61	36.88	93.505	(U 5*f* / 6*d*) + (COT π)
**137**	6.738	6.21	1.30	52.82	39.65	91.727	(U 5*f* / 6*d*) + (COT π)
**143**	5.524	6.54	2.15	46.30	45.00	92.885	(U 5*f* / 6*d*) + (COT π)
**147**	6.632	5.10	1.72	44.01	49.16	91.983	(U 5*f* / 6*d*) + (COT π)
**151**	5.582	5.28	2.69	45.74	46.29	92.922	(U 5*f* / 6*d*) + (COT π)
**157**	5.411	6.58	1.68	48.28	43.46	93.703	(U 5*f* / 6*d*) + (COT π)

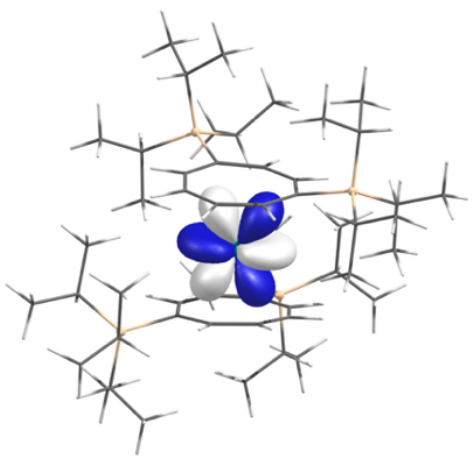
NLMO 116

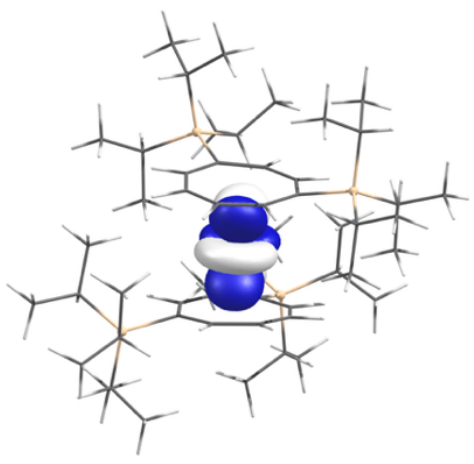
NLMO 117

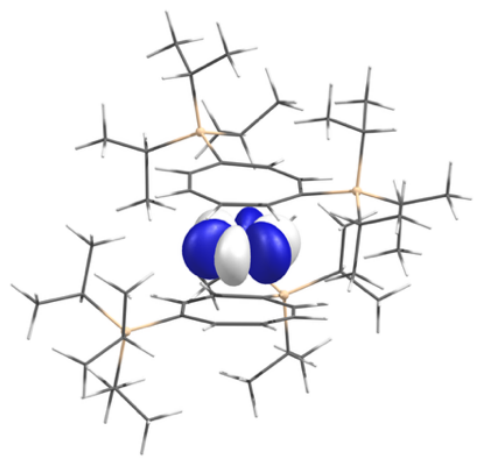
NLMO 118

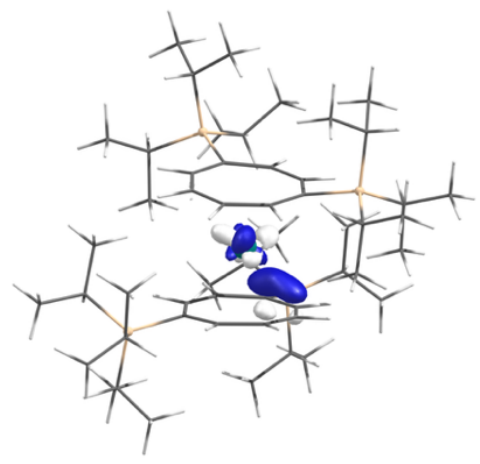
NLMO 119

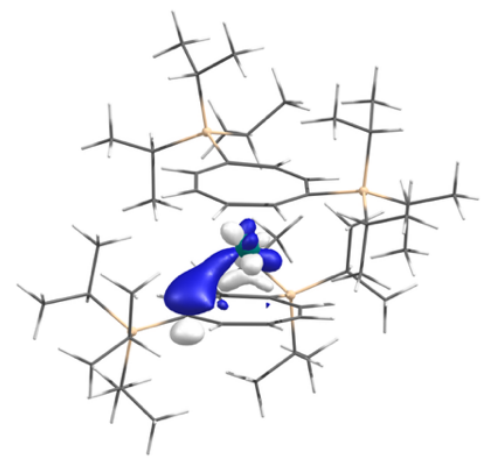
NLMO 120

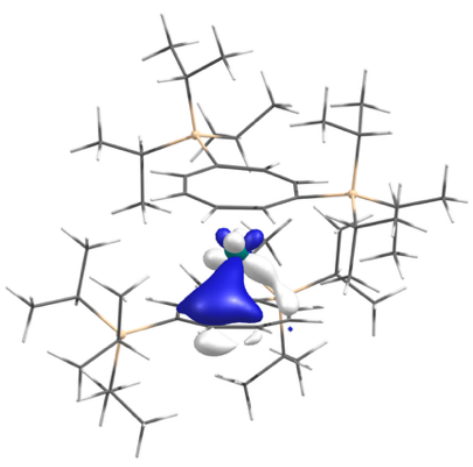
NLMO 121

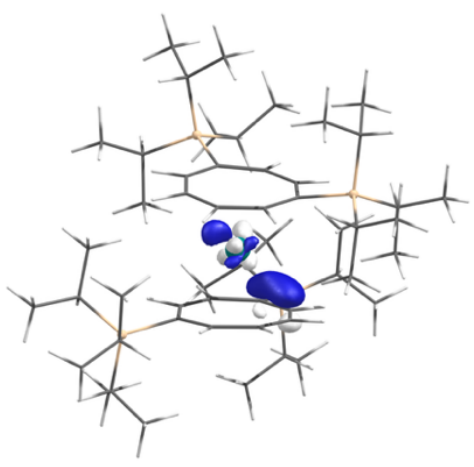
NLMO 122

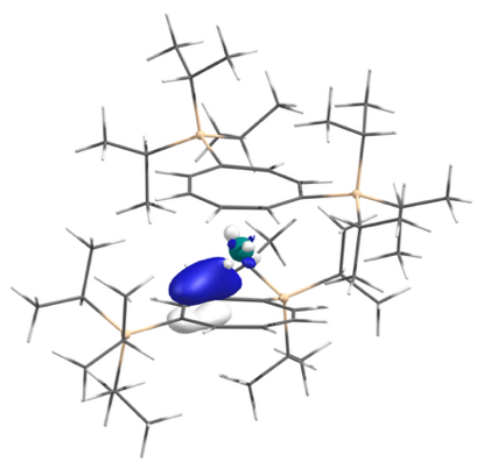
NLMO 128

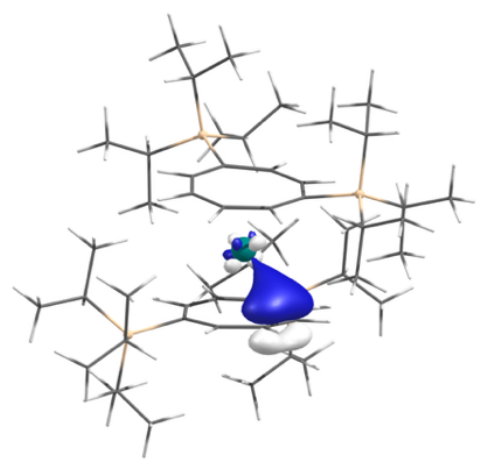
NLMO 137

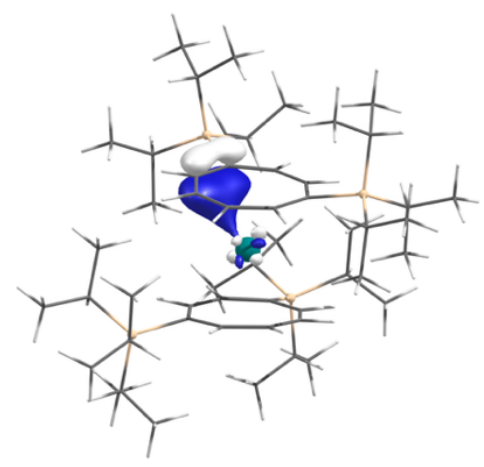
NLMO 143

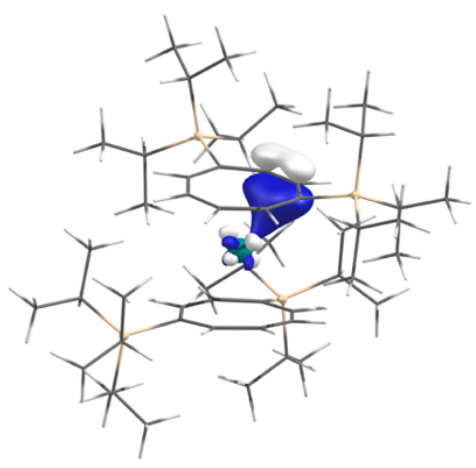
NLMO 147

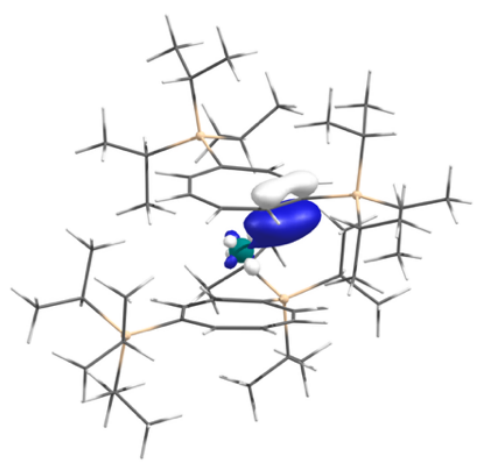
NLMO 151

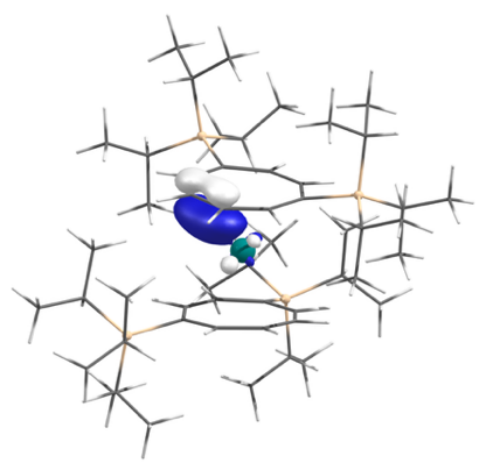
NLMO 157

Figure S237. Selected NLMO isosurfaces for **5**. Isosurface values 0.10 e Å^{-3}.

Table S54. NLMO compositions for selected orbitals of **6** with metal content >4%.

NLMO#	M (%)					COT C_8 total (%)	Description
	Total	*s*	*p*	*d*	*f*		
**116**	99.245	0.00	0.01	0.26	99.73	0.00	U {5f_{xyz}}
**117**	48.509	0.00	0.01	10.31	89.68	49.979	(U 5*f* / 6*d*) + (COT π) δ
**118**	45.962	0.01	0.00	12.07	87.92	50.768	(U 5*f* / 6*d*) + (COT π) δ
**120**	8.285	4.70	0.38	52.19	42.73	88.129	(U 5*f* / 6*d*) + (COT π)
**127**	8.318	3.69	0.31	46.22	49.78	88.472	(U 5*f* / 6*d*) + (COT π)
**132**	11.602	4.67	0.17	46.81	48.33	84.868	(U 5*f* / 6*d*) + (COT π)
**137**	14.524	3.73	0.17	48.24	47.86	81.306	(U 5*f* / 6*d*) + (COT π)
**141**	10.312	2.83	0.15	47.29	49.72	85.846	(U 5*f* / 6*d*) + (COT π)
**145**	13.127	2.83	0.15	47.29	49.72	82.668	(U 5*f* / 6*d*) + (COT π)
**149**	8.703	4.61	0.32	48.26	46.80	87.140	(U 5*f* / 6*d*) + (COT π)
**155**	8.865	5.30	0.30	59.76	34.63	87.309	(U 5*f* / 6*d*) + (COT π)

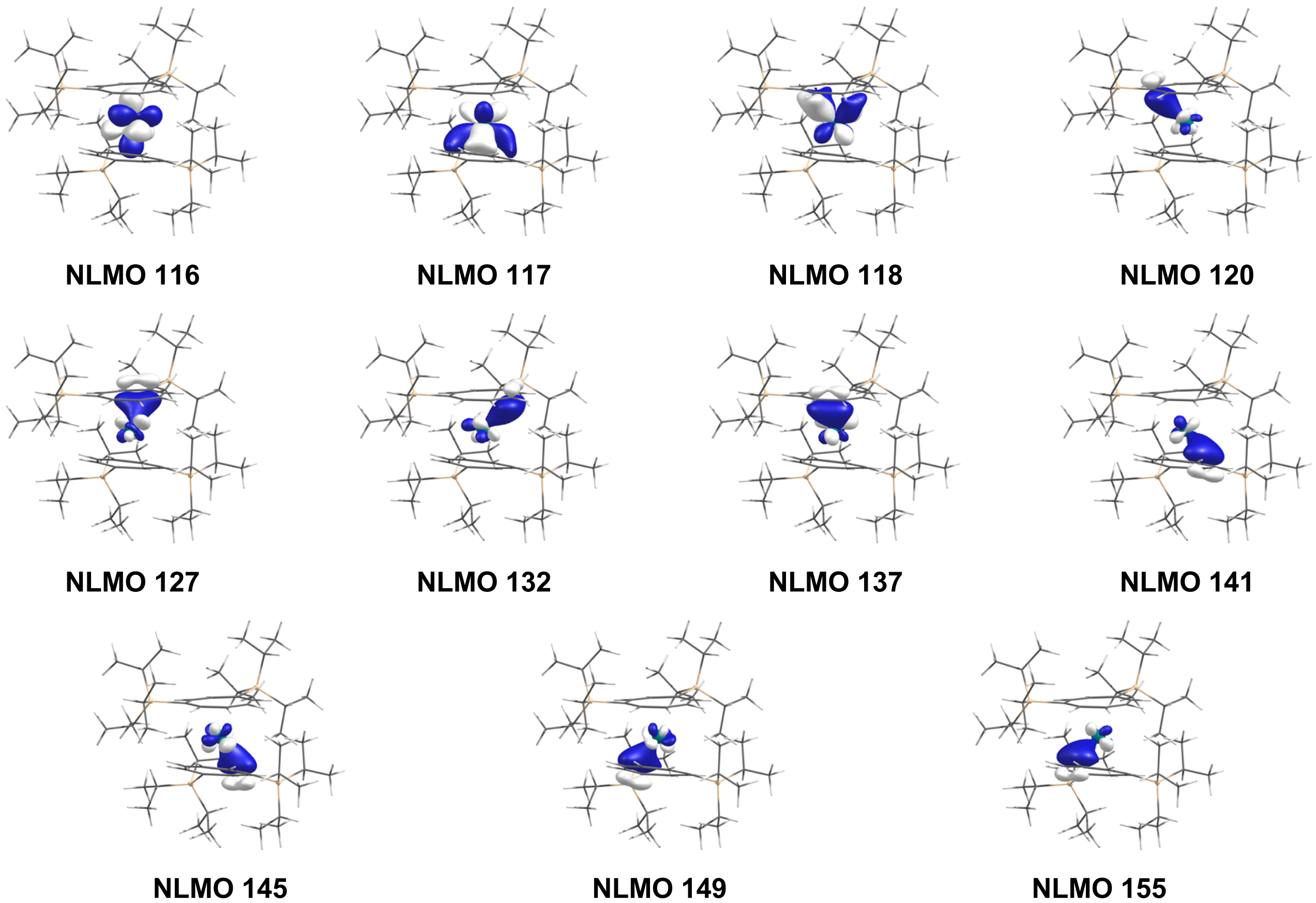

Figure S238. Selected NLMO isosurfaces for **6**. Isosurface values 0.10 e Å^{-3}.

Summary of δ-bonding MO and NLMO compositions for U(IV/V) complexes

Table S55. MO compositions for the δ-bonding orbitals for the specified complexes (U(IV): **1**, **1-D8h**, **4**; U(V): **3**, **3-C2v**, **3-D8d**, **6**).

Complex [A]	MO	M (%)					COT C_8 total (%)
		Total	*s*	*p*	*d*	*f*	
1	**102**	28.1	0.0	0.0	0.0	28.1	68.4
	**101**	33.7	0.0	0.0	0.0	33.7	62.8
	**98**	21.3	0.0	0.0	21.3	0.0	73.8
	**97**	21.2	0.0	0.0	21.2	0.0	74.6
1-D8h	**102**	21.7	0.0	0.0	0.0	21.7	74.4
	**101**	28.6	0.0	0.0	0.0	28.6	68.8
	**98**	21.1	0.0	0.0	21.2	0.0	76.8
	**97**	21.9	0.0	0.0	21.9	0.0	73.6
4	**278**	33.6	0.0	0.0	0.1	33.5	57.3
	**277**	25.2	0.0	0.0	0.0	25.2	65.0
	**274**	18.9	0.0	0.0	18.4	0.5	65.5
	**273**	18.6	0.0	0.1	18.4	0.1	64.7
3	**101**	42.7	0.0	0.0	0.3	42.4	53.6
	**100**	43.0	0.0	0.0	0.2	42.8	52.4
	**99**	32.8	0.0	0.1	19.2	13.5	63.6
	**98**	28.0	0.0	0.0	20.3	7.7	67.9
3-C2v	**101**	35.9	0.0	0.0	0.0	35.9	60.6
	**100**	44.5	0.0	0.0	0.0	44.5	52.0
	**99**	62.9	0.0	0.2	9.4	53.3	34.2
	**98**	21.1	0.0	0.0	21.1	21.1	75.2
3-D8d	**101**	44.3	0.0	0.0	0.0	44.3	52.0
	**100**	44.1	0.0	0.0	0.0	44.1	51.2
	**99**	22.1	0.0	0.0	22.1	0.0	73.2
	**98**	22.1	0.0	0.0	22.1	0.0	72.8
6	**277**	41.3	0.0	0.1	0.2	41.0	43.9
	**276**	42.9	0.0	0.1	0.6	42.2	41.6
	**275**	16.6	0.0	0.2	15.1	1.3	49.8
	**274**	17.0	0.0	0.0	15.1	1.9	51.2

[A] Only the U(IV) and U(V) complexes are compared as the U(III) complexes do not show well-resolved δ-bonding interactions within the NLMO framework.

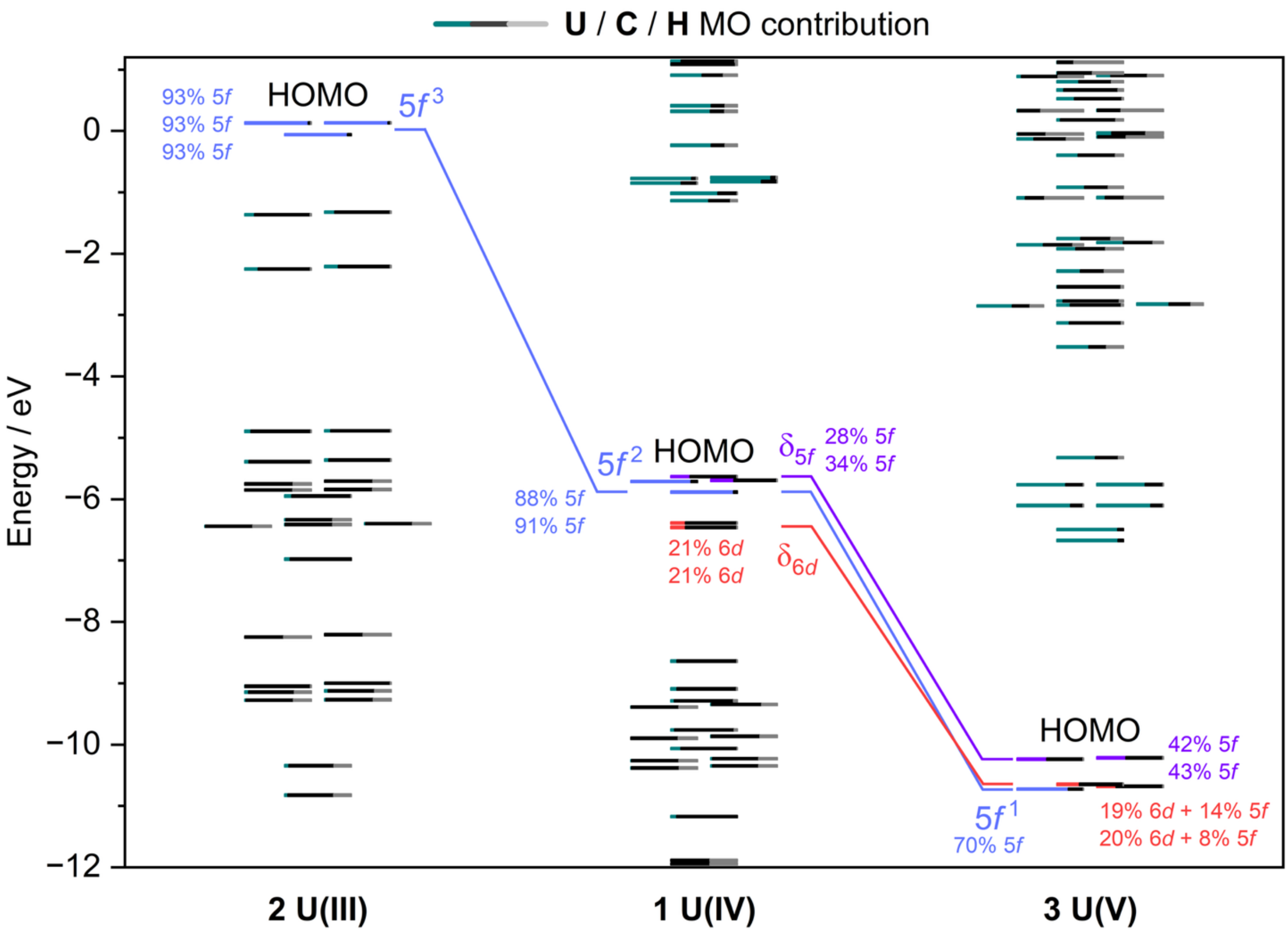

Figure S239. A partial molecular orbital diagram for complexes **1**–**3**, highlighting the positions of the valence *5f* electrons, and the δ-bonding MOs with *5f* and 6*d* contributions. Bar sizes denote relative weighting of U and COT-ring C/H contributions. No shift has been applied to the energy scale.

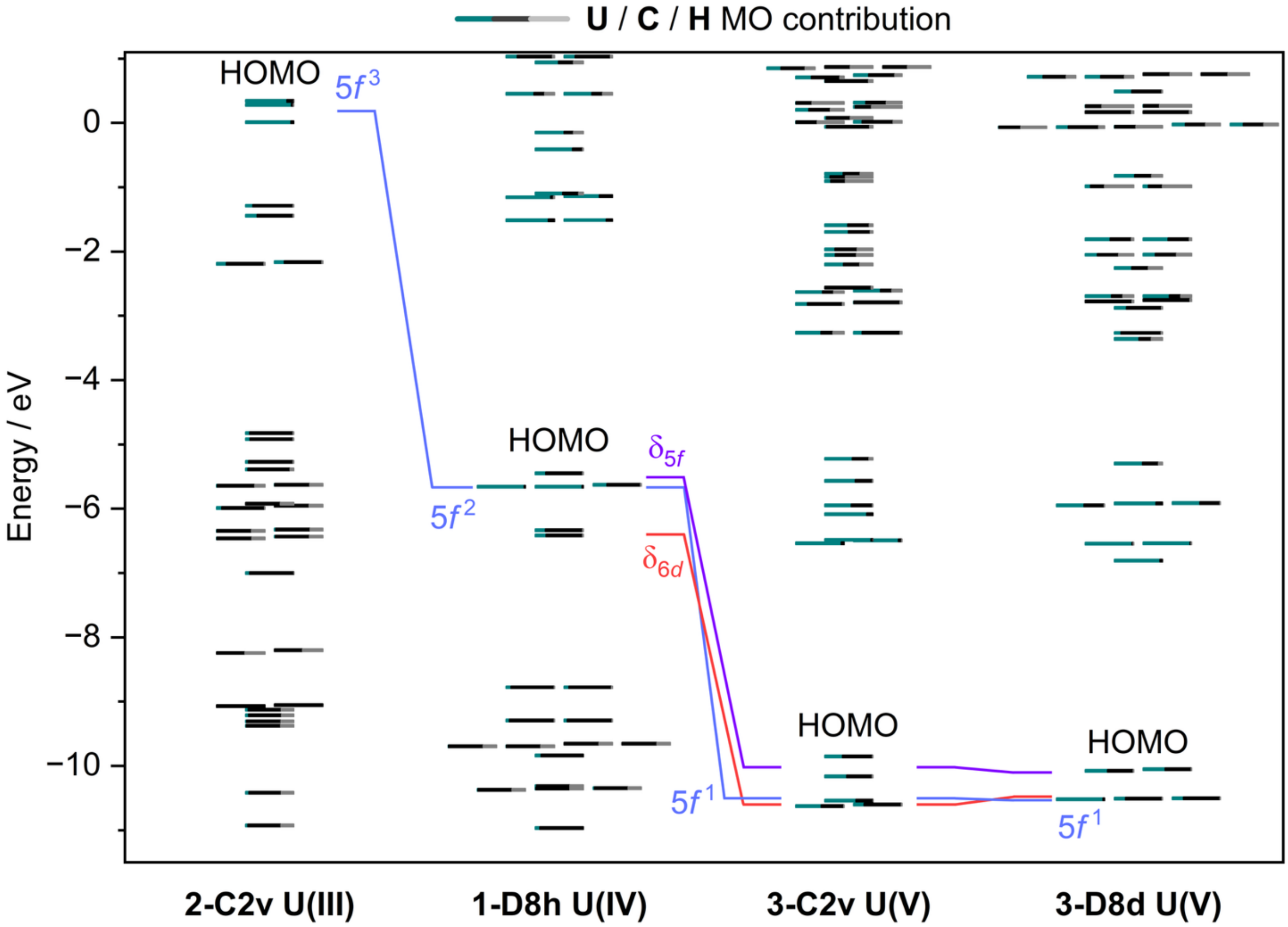

Figure S240. A partial molecular orbital diagram for symmetrized complexes **1**–**3**, highlighting the positions of the valence *5f* electrons, and the δ-bonding MOs with 5*f* and 6*d* contributions. Bar sizes denote relative weighting of U and COT-ring C/H contributions. No shift has been applied to the energy scale.

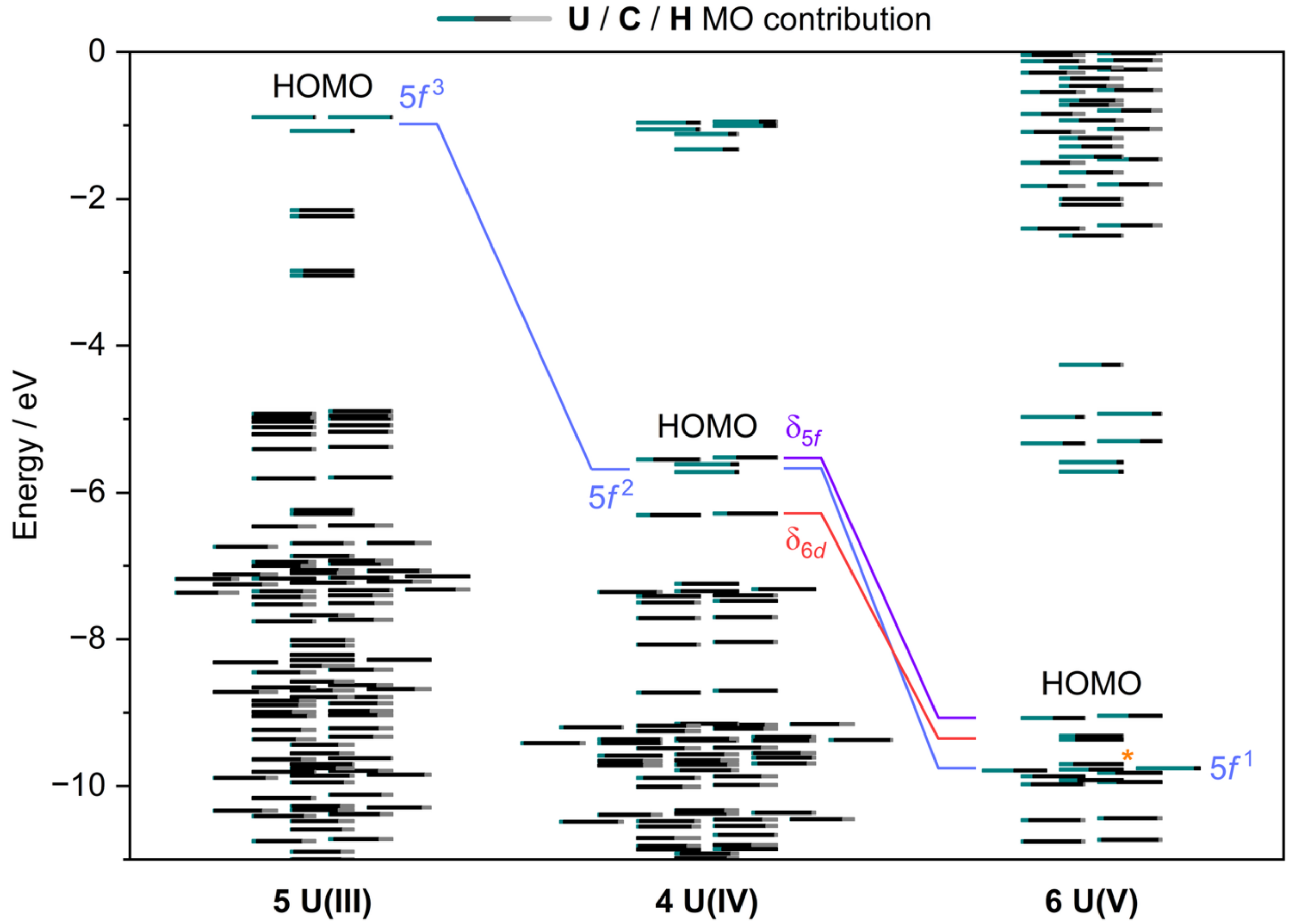

Figure S241. A partial molecular orbital diagram for complexes **4**–**6**, highlighting the positions of the valence 5*f* electrons, and the δ-bonding MOs with 5*f* and 6*d* contributions. Bar sizes denote relative weighting of U and COT-ring C/H contributions. MO indicated with * is dominated by Si-atom contributions, which are not shown for clarity. No shift has been applied to the energy scale.

Table S56. NLMO compositions for selected δ-bonding orbitals with metal content >4% for the specified complexes (U(IV): **1**, **1-D8h**, **4**; U(V): **3**, **3-C2v**, **3-D8d**, **6**).

Complex	NLMO#	M (%)					COT C_8 total (%)
		Total	*s*	*p*	*d*	*f*	
**1**	**62**	34.226	0.00	0.00	0.00	100.00	65.749
	**63**	18.344	0.00	0.00	98.99	0.99	81.361
**1-D8h**	**62**	34.696	0.00	0.00	0.02	99.98	64.811
	**63**	18.577	0.00	0.00	99.72	0.26	77.692
**4**	**118**	25.495	0.17	0.01	28.97	70.84	73.578
	**119**	24.061	0.00	0.01	38.57	61.41	73.420
**3**	**61**	45.855	0.05	0.00	11.22	88.73	52.199
	**62**	40.978	0.13	0.00	19.36	80.50	56.101
**3-C2v** [A]	**62**	22.108	0.01	0.00	97.07	2.91	75.973
**3-D8d**	**61**	55.410	0.07	0.00	1.04	98.89	39.299
	**62**	31.223	0.00	0.00	49.41	50.58	60.234
**6**	**117**	48.509	0.00	0.01	10.31	89.68	49.979
	**118**	45.962	0.01	0.00	12.07	87.92	50.768

[A] Only one δ-bonding orbital was located for **3-C2v**.

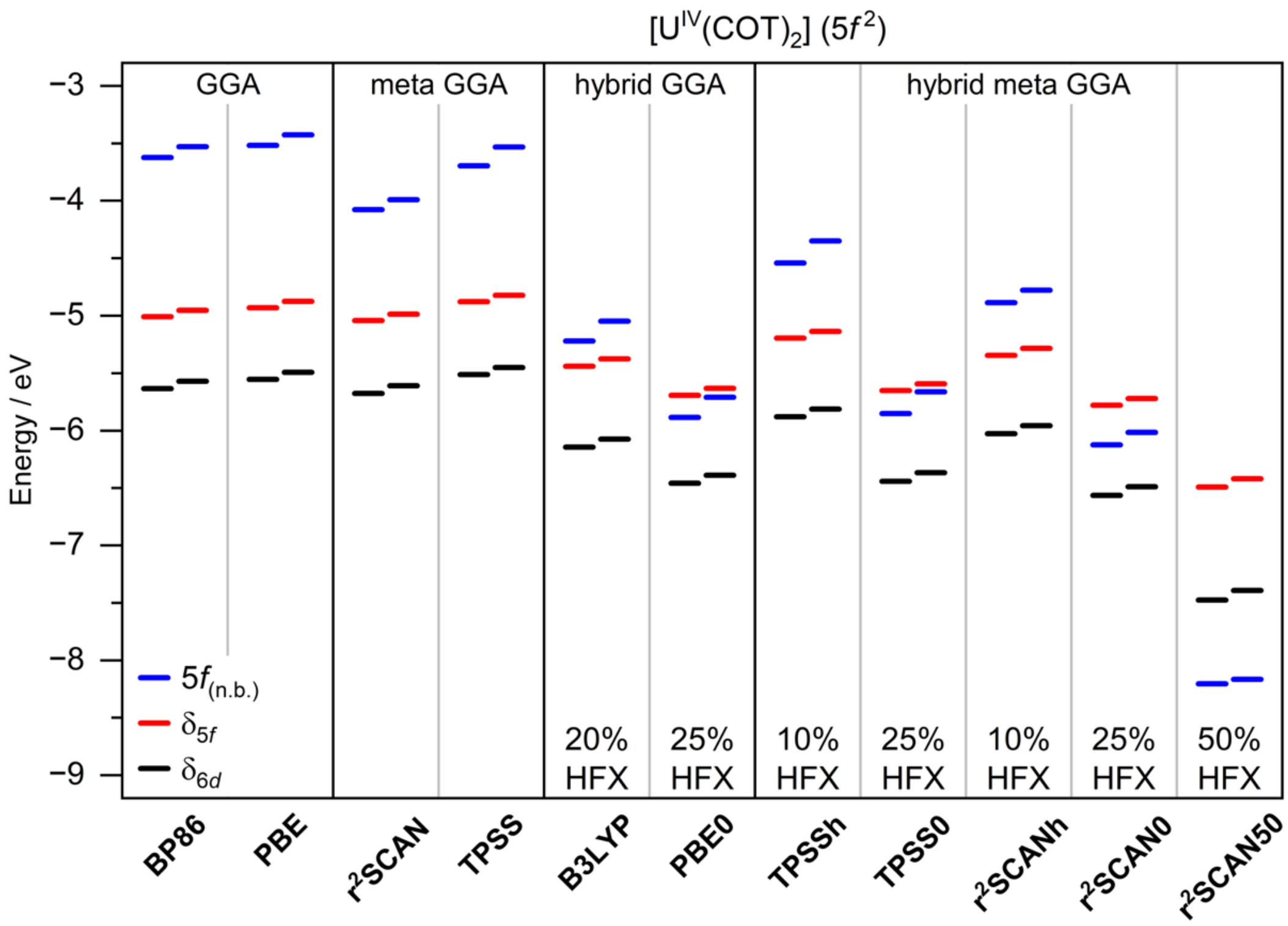

Figure S242. A partial molecular orbital diagram for **1**, highlighting the change in energy for the frontier δ-bonding and 5*f* non-bonding orbitals for different DFT functionals.

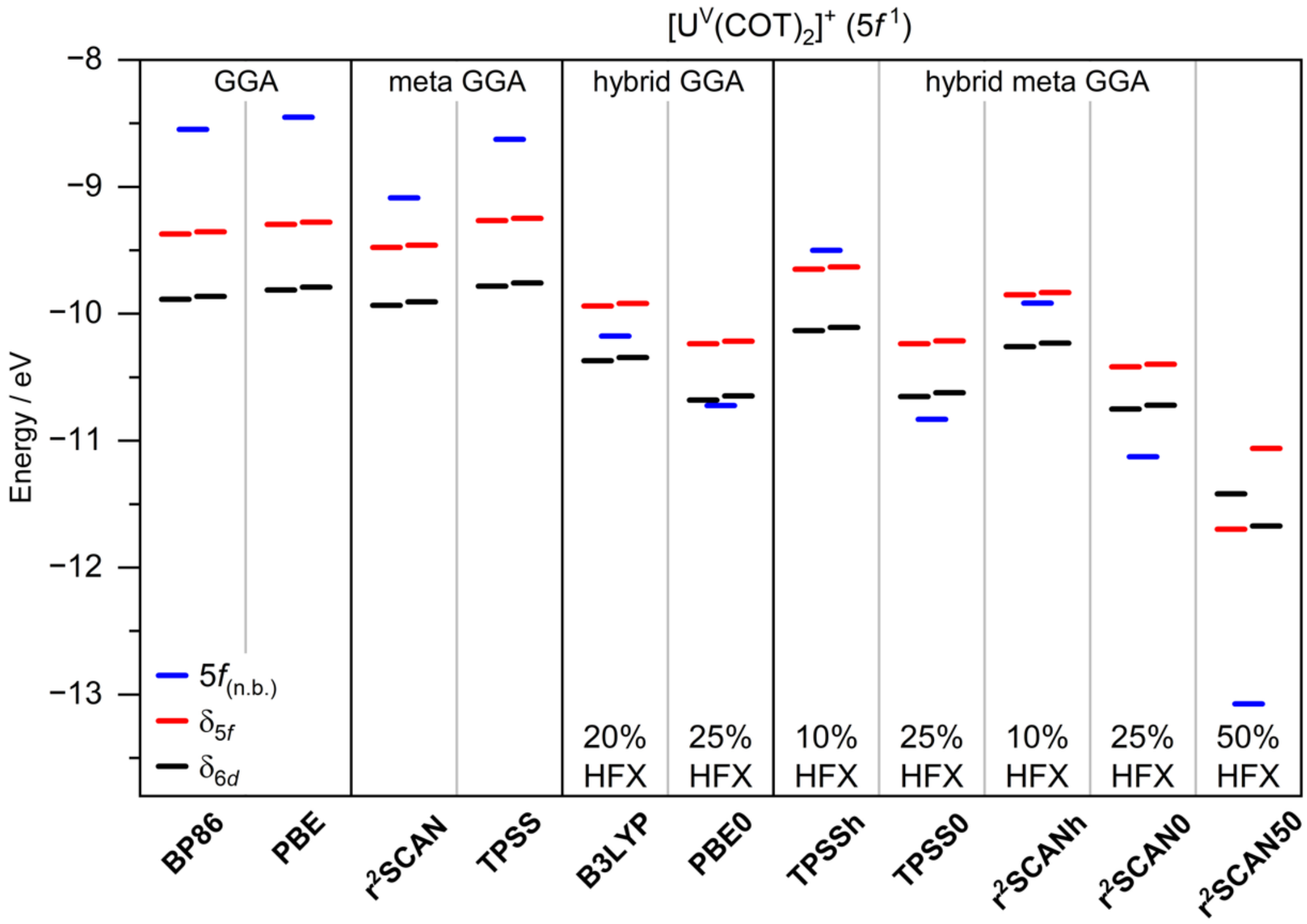

Figure S243. A partial molecular orbital diagram for **3**, highlighting the change in energy for the frontier δ-bonding and 5*f* non-bonding orbitals for different DFT functionals.

*Energy decomposition analysis for $\{M(COT^R)\}^{n+}$ + $\{COT^R\}^{2-}$ with **1–6***

Table S57. Energy decomposition and bonding analysis for H-atom optimized (**1**), fully optimized (**1-Opt**), and D_{8h} (**1-D8h**) coordinates for **1**.

**1**	**Hartrees**	**eV**	**Kcal mol^{-1}**	**kJ mol^{-1}**
**Δ*E*$_{Pauli}$**	0.28	7.61	175.52	734.37
**Δ*E*$_{elstat}$**	–0.77	–20.87	–481.17	–2013.24
**Δ*E*$_{steric}$**	–0.49	–13.25	–305.66	–1278.87
**Δ*E*$_{disp}$**	–0.01	–0.30	–6.87	–28.73
**Δ*E*$_{orb}$ (Total)**	–0.41	–11.10	–256.08	–1071.44
Δ*E*$_{orb}$ (Kinetic)	–1.45	–39.44	–909.44	–3805.12
Δ*E*$_{orb}$ (Coulomb)	1.01	27.37	631.22	2641.02
**Δ*E*$_{int}$**	–0.91	–24.66	–568.60	–2379.04

**1-Opt**	**Hartrees**	**eV**	**Kcal mol^{-1}**	**kJ mol^{-1}**
**Δ*E*$_{Pauli}$**	0.27	7.44	171.49	717.51
**Δ*E*$_{elstat}$**	–0.77	–20.83	–480.28	–2009.50
**Δ*E*$_{steric}$**	–0.49	–13.39	–308.79	–1292.00
**Δ*E*$_{disp}$**	–0.01	–0.30	–6.89	–28.81
**Δ*E*$_{orb}$ (Total)**	–0.40	–10.93	–252.01	–1054.41
Δ*E*$_{orb}$ (Kinetic)	–1.39	–37.75	–870.60	–3642.61
Δ*E*$_{orb}$ (Coulomb)	0.96	26.25	605.34	2532.76
**Δ*E*$_{int}$**	–0.90	–24.62	–567.69	–2375.22

**1-D8h**	**Hartrees**	**eV**	**Kcal mol^{-1}**	**kJ mol^{-1}**
**Δ*E*$_{Pauli}$**	0.26	7.07	163.09	682.36
**Δ*E*$_{elstat}$**	–0.76	–20.59	–474.76	–1986.39
**Δ*E*$_{steric}$**	–0.50	–13.52	–311.67	–1304.04
**Δ*E*$_{disp}$**	–0.01	–0.29	–6.67	–27.89
**Δ*E*$_{orb}$ (Total)**	–0.40	–10.96	–252.82	–1057.80
Δ*E*$_{orb}$ (Kinetic)	–1.35	–36.68	–845.79	–3538.78
Δ*E*$_{orb}$ (Coulomb)	0.91	24.68	569.21	2381.56
**Δ*E*$_{int}$**	–0.91	–24.77	–571.16	–2389.73

Table S58. Energy decomposition and bonding analysis for H-atom optimized (**2**), fully optimized (**2-Opt**), and C_{2v} (**2-C2v**) coordinates for **2**.

**2**	**Hartrees**	**eV**	**Kcal mol^{-1}**	**kJ mol^{-1}**
**ΔE_{Pauli}**	0.21	5.69	131.18	548.85
**ΔE_{elstat}**	–0.48	–13.03	–300.47	–1257.15
**ΔE_{steric}**	–0.27	–7.34	–169.29	–708.30
**ΔE_{disp}**	–0.01	–0.27	–6.14	–25.70
**ΔE_{orb} (Total)**	–0.25	–6.93	–159.83	–668.72
ΔE_{orb} (Kinetic)	–1.89	–51.44	–1186.24	–4963.22
ΔE_{orb} (Coulomb)	1.57	42.85	988.09	4134.18
**ΔE_{int}**	–0.53	–14.54	–335.26	–1402.72

**2-Opt**	**Hartrees**	**eV**	**Kcal mol^{-1}**	**kJ mol^{-1}**
**ΔE_{Pauli}**	0.21	5.79	133.56	558.82
**ΔE_{elstat}**	–0.48	–13.09	–301.97	–1263.42
**ΔE_{steric}**	–0.27	–7.30	–168.40	–704.60
**ΔE_{disp}**	–0.01	–0.27	–6.12	–25.60
**ΔE_{orb} (Total)**	–0.26	–7.01	–161.58	–676.06
ΔE_{orb} (Kinetic)	–1.53	–41.66	–960.59	–4019.13
ΔE_{orb} (Coulomb)	1.22	33.07	762.58	3190.64
**ΔE_{int}**	–0.54	–14.57	–336.10	–1406.26

**2-C2v**	**Hartrees**	**eV**	**Kcal mol^{-1}**	**kJ mol^{-1}**
**ΔE_{Pauli}**	0.24	6.52	150.33	628.98
**ΔE_{elstat}**	–0.48	–13.12	–302.63	–1266.18
**ΔE_{steric}**	–0.24	–6.60	–152.29	–637.20
**ΔE_{disp}**	–0.01	–0.26	–6.09	–25.47
**ΔE_{orb} (Total)**	–0.25	–6.88	–158.68	–663.93
ΔE_{orb} (Kinetic)	–1.94	–52.76	–1216.74	–5090.83
ΔE_{orb} (Coulomb)	1.62	44.18	1018.70	4262.26
**ΔE_{int}**	–0.51	–13.75	–317.06	–1326.60

Table S59. Energy decomposition and bonding analysis for H-atom optimized (**3**), fully optimized (**3-Opt**), C_{2v} (**3-C2v**), and D_{8d} (**3-D8d**) coordinates for **3**.

**3**	**Hartrees**	**eV**	**Kcal mol^{-1}**	**kJ mol^{-1}**
**Δ*E*$_{Pauli}$**	0.31	8.48	195.58	818.31
**Δ*E*$_{elstat}$**	–1.03	–28.09	–647.80	–2710.38
**Δ*E*$_{steric}$**	–0.72	–19.61	–452.22	–1892.07
**Δ*E*$_{disp}$**	–0.01	–0.32	–7.36	–30.80
**Δ*E*$_{orb}$ (Total)**	–0.58	–15.79	–364.16	–1523.66
Δ*E*$_{orb}$ (Kinetic)	–1.50	–40.72	–938.97	–3928.64
Δ*E*$_{orb}$ (Coulomb)	0.83	22.59	520.96	2179.68
**Δ*E*$_{int}$**	–1.31	–35.72	–823.74	–3446.54

**3-Opt**	**Hartrees**	**eV**	**Kcal mol^{-1}**	**kJ mol^{-1}**
**Δ*E*$_{Pauli}$**	0.31	8.30	191.52	801.30
**Δ*E*$_{elstat}$**	–1.04	–28.26	–651.74	–2726.86
**Δ*E*$_{steric}$**	–0.73	–19.96	–460.22	–1925.56
**Δ*E*$_{disp}$**	–0.01	–0.32	–7.31	–30.59
**Δ*E*$_{orb}$ (Total)**	–0.59	–15.93	–367.33	–1536.92
Δ*E*$_{orb}$ (Kinetic)	–1.24	–33.77	–778.64	–3257.84
Δ*E*$_{orb}$ (Coulomb)	0.66	17.99	414.89	1735.90
**Δ*E*$_{int}$**	–1.33	–36.20	–834.86	–3493.07

**3-C2v**	**Hartrees**	**eV**	**Kcal mol^{-1}**	**kJ mol^{-1}**
**Δ*E*$_{Pauli}$**	0.34	9.14	210.67	881.46
**Δ*E*$_{elstat}$**	–1.04	–28.39	–654.71	–2739.31
**Δ*E*$_{steric}$**	–0.71	–19.26	–444.04	–1857.84
**Δ*E*$_{disp}$**	–0.01	–0.32	–7.39	–30.93
**Δ*E*$_{orb}$ (Total)**	–0.58	–15.88	–366.32	–1532.66
Δ*E*$_{orb}$ (Kinetic)	–1.62	–43.96	–1013.75	–4241.53
Δ*E*$_{orb}$ (Coulomb)	0.94	25.54	589.00	2464.36
**Δ*E*$_{int}$**	–1.30	–35.46	–817.74	–3421.44

**3-D8d**	**Hartrees**	**eV**	**Kcal mol^{-1}**	**kJ mol^{-1}**
**Δ*E*$_{Pauli}$**	0.30	8.17	188.38	788.19
**Δ*E*$_{elstat}$**	–1.02	–27.84	–641.90	–2685.72
**Δ*E*$_{steric}$**	–0.72	–19.67	–453.52	–1897.53
**Δ*E*$_{disp}$**	–0.01	–0.31	–7.13	–29.82
**Δ*E*$_{orb}$ (Total)**	–0.57	–15.44	–356.12	–1490.01
Δ*E*$_{orb}$ (Kinetic)	–1.48	–40.37	–930.85	–3894.67
Δ*E*$_{orb}$ (Coulomb)	0.81	21.96	506.30	2118.36
**Δ*E*$_{int}$**	–1.30	–35.42	–816.77	–3417.36

Table S60. Energy decomposition and bonding analysis for H-atom optimized coordinates for **1**–**3**, ordered by uranium oxidation state.

**2**	**Hartrees**	**eV**	**Kcal mol^{-1}**	**kJ mol^{-1}**
**Δ*E*$_{Pauli}$**	0.21	5.69	131.18	548.85
**Δ*E*$_{elstat}$**	–0.48	–13.03	–300.47	–1257.15
**Δ*E*$_{steric}$**	–0.27	–7.34	–169.29	–708.30
**Δ*E*$_{disp}$**	–0.01	–0.27	–6.14	–25.70
**Δ*E*$_{orb}$ (Total)**	–0.25	–6.93	–159.83	–668.72
Δ*E*$_{orb}$ (Kinetic)	–1.89	–51.44	–1186.24	–4963.22
Δ*E*$_{orb}$ (Coulomb)	1.57	42.85	988.09	4134.18
**Δ*E*$_{int}$**	–0.53	–14.54	–335.26	–1402.72

**1**	**Hartrees**	**eV**	**Kcal mol^{-1}**	**kJ mol^{-1}**
**Δ*E*$_{Pauli}$**	0.28	7.61	175.52	734.37
**Δ*E*$_{elstat}$**	–0.77	–20.87	–481.17	–2013.24
**Δ*E*$_{steric}$**	–0.49	–13.25	–305.66	–1278.87
**Δ*E*$_{disp}$**	–0.01	–0.30	–6.87	–28.73
**Δ*E*$_{orb}$ (Total)**	–0.41	–11.10	–256.08	–1071.44
Δ*E*$_{orb}$ (Kinetic)	–1.45	–39.44	–909.44	–3805.12
Δ*E*$_{orb}$ (Coulomb)	1.01	27.37	631.22	2641.02
**Δ*E*$_{int}$**	–0.91	–24.66	–568.60	–2379.04

**3**	**Hartrees**	**eV**	**Kcal mol^{-1}**	**kJ mol^{-1}**
**Δ*E*$_{Pauli}$**	0.31	8.48	195.58	818.31
**Δ*E*$_{elstat}$**	–1.03	–28.09	–647.80	–2710.38
**Δ*E*$_{steric}$**	–0.72	–19.61	–452.22	–1892.07
**Δ*E*$_{disp}$**	–0.01	–0.32	–7.36	–30.80
**Δ*E*$_{orb}$ (Total)**	–0.58	–15.79	–364.16	–1523.66
Δ*E*$_{orb}$ (Kinetic)	–1.50	–40.72	–938.97	–3928.64
Δ*E*$_{orb}$ (Coulomb)	0.83	22.59	520.96	2179.68
**Δ*E*$_{int}$**	–1.31	–35.72	–823.74	–3446.54

Table S61. Energy decomposition and bonding analysis for fully optimized (**1-Opt**, **2-Opt**, **3-Opt**) coordinates for **1–3**, ordered by uranium oxidation state.

**2-Opt**	**Hartrees**	**eV**	**Kcal mol^{-1}**	**kJ mol^{-1}**
**Δ*E*$_{Pauli}$**	0.21	5.79	133.56	558.82
**Δ*E*$_{elstat}$**	–0.48	–13.09	–301.97	–1263.42
**Δ*E*$_{steric}$**	–0.27	–7.30	–168.40	–704.60
**Δ*E*$_{disp}$**	–0.01	–0.27	–6.12	–25.60
**Δ*E*$_{orb}$ (Total)**	–0.26	–7.01	–161.58	–676.06
Δ*E*$_{orb}$ (Kinetic)	–1.53	–41.66	–960.59	–4019.13
Δ*E*$_{orb}$ (Coulomb)	1.22	33.07	762.58	3190.64
**Δ*E*$_{int}$**	–0.54	–14.57	–336.10	–1406.26

**1-Opt**	**Hartrees**	**eV**	**Kcal mol^{-1}**	**kJ mol^{-1}**
**Δ*E*$_{Pauli}$**	0.27	7.44	171.49	717.51
**Δ*E*$_{elstat}$**	–0.77	–20.83	–480.28	–2009.50
**Δ*E*$_{steric}$**	–0.49	–13.39	–308.79	–1292.00
**Δ*E*$_{disp}$**	–0.01	–0.30	–6.89	–28.81
**Δ*E*$_{orb}$ (Total)**	–0.40	–10.93	–252.01	–1054.41
Δ*E*$_{orb}$ (Kinetic)	–1.39	–37.75	–870.60	–3642.61
Δ*E*$_{orb}$ (Coulomb)	0.96	26.25	605.34	2532.76
**Δ*E*$_{int}$**	–0.90	–24.62	–567.69	–2375.22

**3-Opt**	**Hartrees**	**eV**	**Kcal mol^{-1}**	**kJ mol^{-1}**
**Δ*E*$_{Pauli}$**	0.31	8.30	191.52	801.30
**Δ*E*$_{elstat}$**	–1.04	–28.26	–651.74	–2726.86
**Δ*E*$_{steric}$**	–0.73	–19.96	–460.22	–1925.56
**Δ*E*$_{disp}$**	–0.01	–0.32	–7.31	–30.59
**Δ*E*$_{orb}$ (Total)**	–0.59	–15.93	–367.33	–1536.92
Δ*E*$_{orb}$ (Kinetic)	–1.24	–33.77	–778.64	–3257.84
Δ*E*$_{orb}$ (Coulomb)	0.66	17.99	414.89	1735.90
**Δ*E*$_{int}$**	–1.33	–36.20	–834.86	–3493.07

Table S62. Energy decomposition and bonding analysis for symmetrized (**1-D8h**, **2-C2v**, **3-C2v**, **3-D8d**) coordinates for **1–3**, ordered by uranium oxidation state.

**2-C2v**	**Hartrees**	**eV**	**Kcal mol^{-1}**	**kJ mol^{-1}**
**Δ*E*$_{Pauli}$**	0.24	6.52	150.33	628.98
**Δ*E*$_{elstat}$**	–0.48	–13.12	–302.63	–1266.18
**Δ*E*$_{steric}$**	–0.24	–6.60	–152.29	–637.20
**Δ*E*$_{disp}$**	–0.01	–0.26	–6.09	–25.47
**Δ*E*$_{orb}$ (Total)**	–0.25	–6.88	–158.68	–663.93
Δ*E*$_{orb}$ (Kinetic)	–1.94	–52.76	–1216.74	–5090.83
Δ*E*$_{orb}$ (Coulomb)	1.62	44.18	1018.70	4262.26
**Δ*E*$_{int}$**	–0.51	–13.75	–317.06	–1326.60

**1-D8h**	**Hartrees**	**eV**	**Kcal mol^{-1}**	**kJ mol^{-1}**
**Δ*E*$_{Pauli}$**	0.26	7.07	163.09	682.36
**Δ*E*$_{elstat}$**	–0.76	–20.59	–474.76	–1986.39
**Δ*E*$_{steric}$**	–0.50	–13.52	–311.67	–1304.04
**Δ*E*$_{disp}$**	–0.01	–0.29	–6.67	–27.89
**Δ*E*$_{orb}$ (Total)**	–0.40	–10.96	–252.82	–1057.80
Δ*E*$_{orb}$ (Kinetic)	–1.35	–36.68	–845.79	–3538.78
Δ*E*$_{orb}$ (Coulomb)	0.91	24.68	569.21	2381.56
**Δ*E*$_{int}$**	–0.91	–24.77	–571.16	–2389.73

**3-C2v**	**Hartrees**	**eV**	**Kcal mol^{-1}**	**kJ mol^{-1}**
**Δ*E*$_{Pauli}$**	0.34	9.14	210.67	881.46
**Δ*E*$_{elstat}$**	–1.04	–28.39	–654.71	–2739.31
**Δ*E*$_{steric}$**	–0.71	–19.26	–444.04	–1857.84
**Δ*E*$_{disp}$**	–0.01	–0.32	–7.39	–30.93
**Δ*E*$_{orb}$ (Total)**	–0.58	–15.88	–366.32	–1532.66
Δ*E*$_{orb}$ (Kinetic)	–1.62	–43.96	–1013.75	–4241.53
Δ*E*$_{orb}$ (Coulomb)	0.94	25.54	589.00	2464.36
**Δ*E*$_{int}$**	–1.30	–35.46	–817.74	–3421.44

**3-D8d**	**Hartrees**	**eV**	**Kcal mol^{-1}**	**kJ mol^{-1}**
**Δ*E*$_{Pauli}$**	0.30	8.17	188.38	788.19
**Δ*E*$_{elstat}$**	–1.02	–27.84	–641.90	–2685.72
**Δ*E*$_{steric}$**	–0.72	–19.67	–453.52	–1897.53
**Δ*E*$_{disp}$**	–0.01	–0.31	–7.13	–29.82
**Δ*E*$_{orb}$ (Total)**	–0.57	–15.44	–356.12	–1490.01
Δ*E*$_{orb}$ (Kinetic)	–1.48	–40.37	–930.85	–3894.67
Δ*E*$_{orb}$ (Coulomb)	0.81	21.96	506.30	2118.36
**Δ*E*$_{int}$**	–1.30	–35.42	–816.77	–3417.36

Table S63. Energy decomposition and bonding analysis for H-atom optimized (**4**), and fully optimized (**4-Opt**) coordinates for **4**.

**4**	**Hartrees**	**eV**	**Kcal mol^{-1}**	**kJ mol^{-1}**
**Δ*E*Pauli**	0.31	8.53	196.60	822.58
**Δ*E*elstat**	–0.64	–17.43	–402.01	–1682.00
**Δ*E*steric**	–0.33	–8.91	–205.41	–859.42
**Δ*E*disp**	–0.04	–1.09	–25.16	–105.28
**Δ*E*orb (Total)**	–0.45	–12.16	–280.40	–1173.20
Δ*E*orb (Kinetic)	–2.60	–70.75	–1631.49	–6826.17
Δ*E*orb (Coulomb)	2.07	56.42	1301.00	5443.38
**Δ*E*int**	–0.81	–22.16	–510.97	–2137.90

**4-Opt**	**Hartrees**	**eV**	**Kcal mol^{-1}**	**kJ mol^{-1}**
**Δ*E*Pauli**	0.30	8.16	188.21	787.46
**Δ*E*elstat**	–0.64	–17.28	–398.60	–1667.73
**Δ*E*steric**	–0.34	–9.12	–210.39	–880.27
**Δ*E*disp**	–0.04	–1.08	–24.87	–104.04
**Δ*E*orb (Total)**	–0.44	–12.00	–276.73	–1157.82
Δ*E*orb (Kinetic)	–2.56	–69.74	–1608.14	–6728.47
Δ*E*orb (Coulomb)	2.04	55.58	1281.73	5362.76
**Δ*E*int**	–0.82	–22.20	–511.98	–2142.12

Table S64. Energy decomposition and bonding analysis for H-atom optimized (**5**), and fully optimized (**5-Opt**) coordinates for **5**.

**5**	**Hartrees**	**eV**	**Kcal mol^{-1}**	**kJ mol^{-1}**
**Δ*E*Pauli**	0.24	6.65	153.43	641.96
**Δ*E*elstat**	–0.42	–11.35	–261.78	–1095.27
**Δ*E*steric**	–0.17	–4.70	–108.34	–453.31
**Δ*E*disp**	–0.04	–0.95	–22.01	–92.08
**Δ*E*orb (Total)**	–0.27	–7.36	–169.73	–710.14
Δ*E*orb (Kinetic)	–2.85	–77.58	–1789.13	–7485.73
Δ*E*orb (Coulomb)	2.49	67.77	1562.93	6539.29
**Δ*E*int**	–0.48	–13.01	–300.08	–1255.53

**5-Opt**	**Hartrees**	**eV**	**Kcal mol^{-1}**	**kJ mol^{-1}**
**Δ*E*Pauli**	0.26	7.19	165.90	694.12
**Δ*E*elstat**	–0.43	–11.57	–266.86	–1116.54
**Δ*E*steric**	–0.16	–4.38	–100.96	–422.41
**Δ*E*disp**	–0.04	–1.04	–23.93	–100.11
**Δ*E*orb (Total)**	–0.28	–7.57	–174.64	–730.68
Δ*E*orb (Kinetic)	–2.80	–76.19	–1756.98	–7351.19
Δ*E*orb (Coulomb)	2.41	65.70	1515.06	6339.02
**Δ*E*int**	–0.48	–12.99	–299.52	–1253.20

Table S65. Energy decomposition and bonding analysis for H-atom optimized (**6**), and fully optimized (**6-Opt**) coordinates for **6**.

**6**	**Hartrees**	**eV**	**Kcal mol^{-1}**	**kJ mol^{-1}**
$\Delta \boldsymbol{E}_{\mathbf{Pauli}}$	0.33	9.05	208.58	872.72
$\Delta \boldsymbol{E}_{\mathbf{elstat}}$	–0.78	–21.34	–492.14	–2059.10
$\Delta \boldsymbol{E}_{\mathbf{steric}}$	–0.45	–12.30	–283.55	–1186.38
$\Delta \boldsymbol{E}_{\mathbf{disp}}$	–0.04	–1.07	–24.59	–102.87
$\Delta \boldsymbol{E}_{\mathbf{orb}}$ **(Total)**	–0.61	–16.55	–381.58	–1596.55
ΔE_{orb} (Kinetic)	–2.61	–70.93	–1635.70	–6843.76
ΔE_{orb} (Coulomb)	1.82	49.44	1140.07	4770.06
$\Delta \boldsymbol{E}_{\mathbf{int}}$	–1.10	–29.91	–689.72	–2885.79

**6-Opt**	**Hartrees**	**eV**	**Kcal mol^{-1}**	**kJ mol^{-1}**
$\Delta \boldsymbol{E}_{\mathbf{Pauli}}$	0.36	9.84	226.98	949.69
$\Delta \boldsymbol{E}_{\mathbf{elstat}}$	–0.79	–21.60	–498.12	–2084.12
$\Delta \boldsymbol{E}_{\mathbf{steric}}$	–0.43	–11.76	–271.14	–1134.43
$\Delta \boldsymbol{E}_{\mathbf{disp}}$	–0.04	–1.11	–25.57	–106.98
$\Delta \boldsymbol{E}_{\mathbf{orb}}$ **(Total)**	–0.63	–17.05	–393.23	–1645.26
ΔE_{orb} (Kinetic)	–2.68	–73.01	–1683.65	–7044.39
ΔE_{orb} (Coulomb)	1.88	51.12	1178.82	4932.20
$\Delta \boldsymbol{E}_{\mathbf{int}}$	–1.10	–29.92	–689.93	–2886.67

The large difference in total bonding energy between **6** and **6-Opt** derives almost entirely from the kinetic component of the ΔE_{orb} interaction. Structurally, the two coordinate sets don't differ by much except for the twist angle between the two COT†† rings, see **Figure 244**.

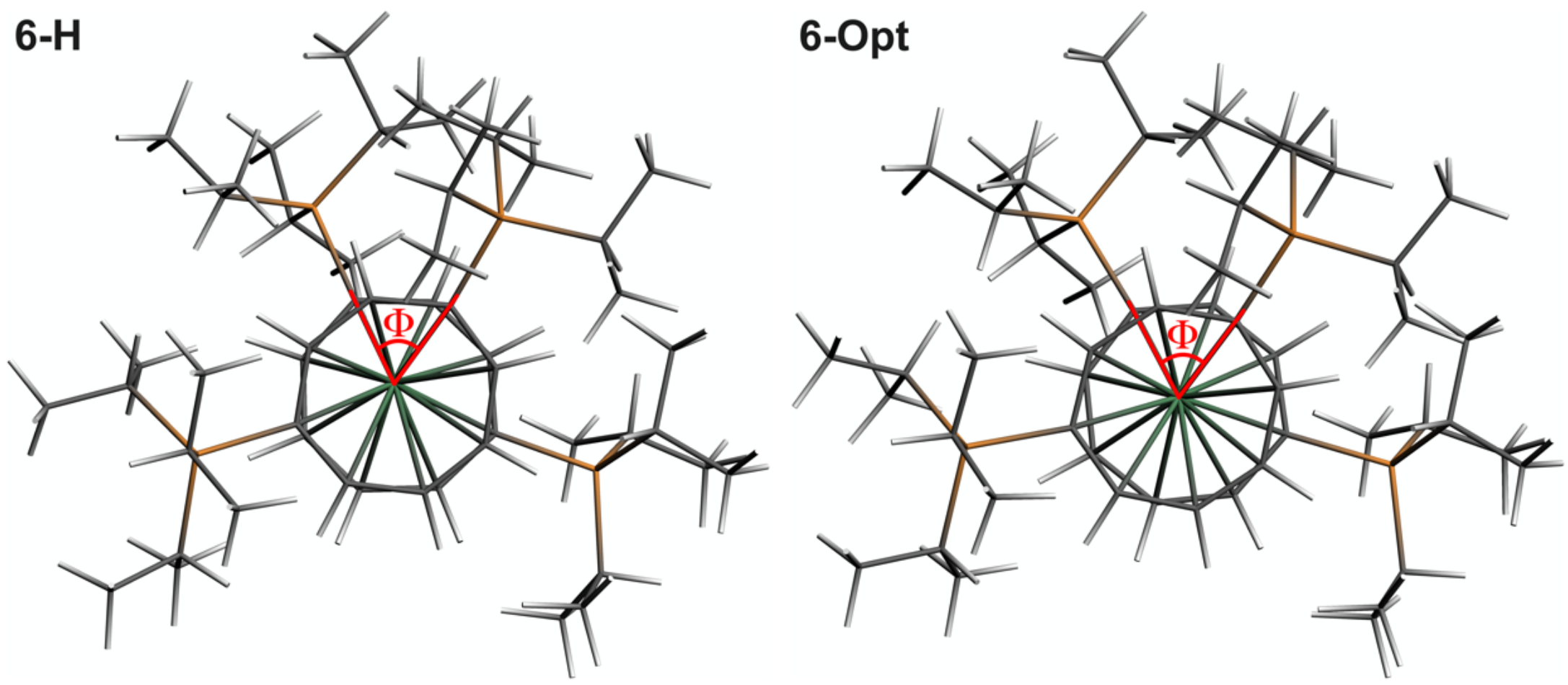

Figure 244. Top–down views of **6** (left) and **6-Opt** (right) showing their differing Φ twist angles between the C(Si*i*Pr$_3$) groups, which are 56.42° and 64.28°, respectively.

Table S66. Energy decomposition and bonding analysis for H-atom optimized coordinates for **4**–**6**, ordered by uranium oxidation state.

**5**	**Hartrees**	**eV**	**Kcal mol^{-1}**	**kJ mol^{-1}**
**ΔE_{Pauli}**	0.24	6.65	153.43	641.96
**ΔE_{elstat}**	–0.42	–11.35	–261.78	–1095.27
**ΔE_{steric}**	–0.17	–4.70	–108.34	–453.31
**ΔE_{disp}**	–0.04	–0.95	–22.01	–92.08
**ΔE_{orb} (Total)**	–0.27	–7.36	–169.73	–710.14
ΔE_{orb} (Kinetic)	–2.85	–77.58	–1789.13	–7485.73
ΔE_{orb} (Coulomb)	2.49	67.77	1562.93	6539.29
**ΔE_{int}**	–0.48	–13.01	–300.08	–1255.53

**4**	**Hartrees**	**eV**	**Kcal mol^{-1}**	**kJ mol^{-1}**
**ΔE_{Pauli}**	0.31	8.53	196.60	822.58
**ΔE_{elstat}**	–0.64	–17.43	–402.01	–1682.00
**ΔE_{steric}**	–0.33	–8.91	–205.41	–859.42
**ΔE_{disp}**	–0.04	–1.09	–25.16	–105.28
**ΔE_{orb} (Total)**	–0.45	–12.16	–280.40	–1173.20
ΔE_{orb} (Kinetic)	–2.60	–70.75	–1631.49	–6826.17
ΔE_{orb} (Coulomb)	2.07	56.42	1301.00	5443.38
**ΔE_{int}**	–0.81	–22.16	–510.97	–2137.90

**6**	**Hartrees**	**eV**	**Kcal mol^{-1}**	**kJ mol^{-1}**
**ΔE_{Pauli}**	0.33	9.05	208.58	872.72
**ΔE_{elstat}**	–0.78	–21.34	–492.14	–2059.10
**ΔE_{steric}**	–0.45	–12.30	–283.55	–1186.38
**ΔE_{disp}**	–0.04	–1.07	–24.59	–102.87
**ΔE_{orb} (Total)**	–0.61	–16.55	–381.58	–1596.55
ΔE_{orb} (Kinetic)	–2.61	–70.93	–1635.70	–6843.76
ΔE_{orb} (Coulomb)	1.82	49.44	1140.07	4770.06
**ΔE_{int}**	–1.10	–29.91	–689.72	–2885.79

Table S67. Energy decomposition and bonding analysis for fully optimized (**4-Opt**, **5-Opt**, **6-Opt**) coordinates for **4–6**, ordered by uranium oxidation state.

**5-Opt**	**Hartrees**	**eV**	**Kcal mol^{-1}**	**kJ mol^{-1}**
**ΔE_{Pauli}**	0.26	7.19	165.90	694.12
**ΔE_{elstat}**	–0.43	–11.57	–266.86	–1116.54
**ΔE_{steric}**	–0.16	–4.38	–100.96	–422.41
**ΔE_{disp}**	–0.04	–1.04	–23.93	–100.11
**ΔE_{orb} (Total)**	–0.28	–7.57	–174.64	–730.68
ΔE_{orb} (Kinetic)	–2.80	–76.19	–1756.98	–7351.19
ΔE_{orb} (Coulomb)	2.41	65.70	1515.06	6339.02
**ΔE_{int}**	–0.48	–12.99	–299.52	–1253.20

**4-Opt**	**Hartrees**	**eV**	**Kcal mol^{-1}**	**kJ mol^{-1}**
**ΔE_{Pauli}**	0.30	8.16	188.21	787.46
**ΔE_{elstat}**	–0.64	–17.28	–398.60	–1667.73
**ΔE_{steric}**	–0.34	–9.12	–210.39	–880.27
**ΔE_{disp}**	–0.04	–1.08	–24.87	–104.04
**ΔE_{orb} (Total)**	–0.44	–12.00	–276.73	–1157.82
ΔE_{orb} (Kinetic)	–2.56	–69.74	–1608.14	–6728.47
ΔE_{orb} (Coulomb)	2.04	55.58	1281.73	5362.76
**ΔE_{int}**	–0.82	–22.20	–511.98	–2142.12

**6-Opt**	**Hartrees**	**eV**	**Kcal mol^{-1}**	**kJ mol^{-1}**
**ΔE_{Pauli}**	0.36	9.84	226.98	949.69
**ΔE_{elstat}**	–0.79	–21.60	–498.12	–2084.12
**ΔE_{steric}**	–0.43	–11.76	–271.14	–1134.43
**ΔE_{disp}**	–0.04	–1.11	–25.57	–106.98
**ΔE_{orb} (Total)**	–0.63	–17.05	–393.23	–1645.26
ΔE_{orb} (Kinetic)	–2.68	–73.01	–1683.65	–7044.39
ΔE_{orb} (Coulomb)	1.88	51.12	1178.82	4932.20
**ΔE_{int}**	–1.10	–29.92	–689.93	–2886.67

*ETS-NOCV deformation density surfaces for H-atom optimized **1–3**.*

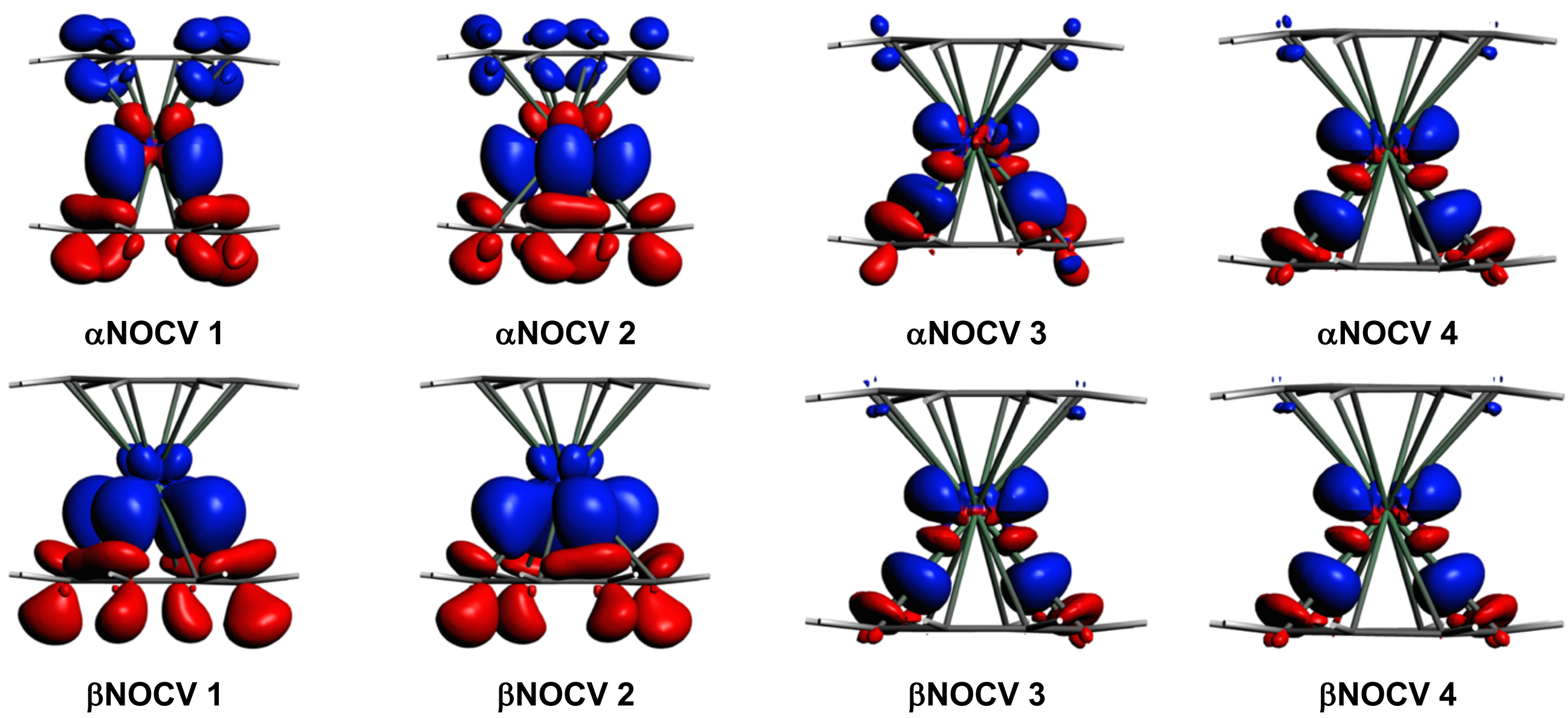

Figure S245. Selected NOCV orbital isosurfaces for **1**. Isosurface values 0.001 a.u. The charge flows from fragments to the full complex from red → blue (view rotated for best angle).

Table S68. NOCV orbital summary for **1** (energies in kJ mol^{-1}).

NOCV #	Eigenvalue	ΔE_{orb} (total)	ΔT_{orb} (kinetic)	ΔV_{orb} (potential)
**α1**	–0.47	–132.46	–1686.70	1554.23
**α2**	–0.45	–128.23	–818.34	690.12
**α3**	–0.22	–58.92	279.36	–338.28
**α4**	–0.21	–58.18	419.78	–477.96
**β1**	–0.35	–100.90	2255.87	–2356.77
**β2**	–0.34	–96.98	2150.75	–2247.72
**β3**	–0.20	–56.51	378.46	–434.97
**β4**	–0.20	–53.71	350.45	–404.16

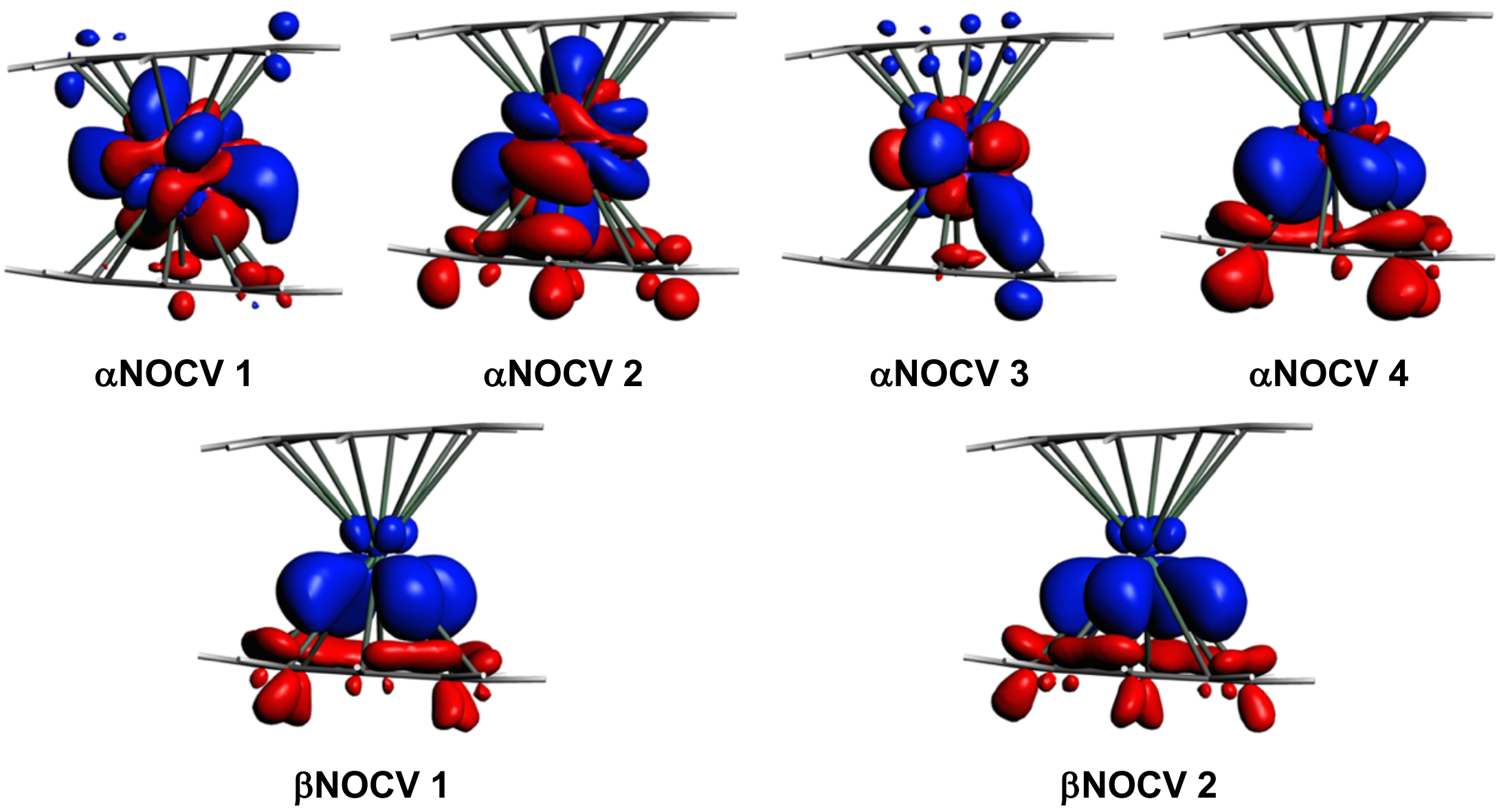

Figure S246. Selected NOCV orbital isosurfaces for **2**. Isosurface values 0.001 a.u. The charge flows from fragments to the full complex from red → blue (view rotated for best angle).

Table S69. NOCV orbital summary for **2** (energies in kJ mol^{-1}).

NOCV #	Eigenvalue	ΔE_{orb} (total)	ΔT_{orb} (kinetic)	ΔV_{orb} (potential)
**α1**	–0.73	–38.92	–2934.20	2895.28
**α2**	–0.57	–61.20	530.46	–591.66
**α3**	–0.51	–30.72	–3347.12	3316.40
**α4**	–0.33	–65.13	1747.72	–1812.85
**β1**	–0.27	–55.66	1088.28	–1143.94
**β2**	–0.26	–53.74	1030.89	–1084.62

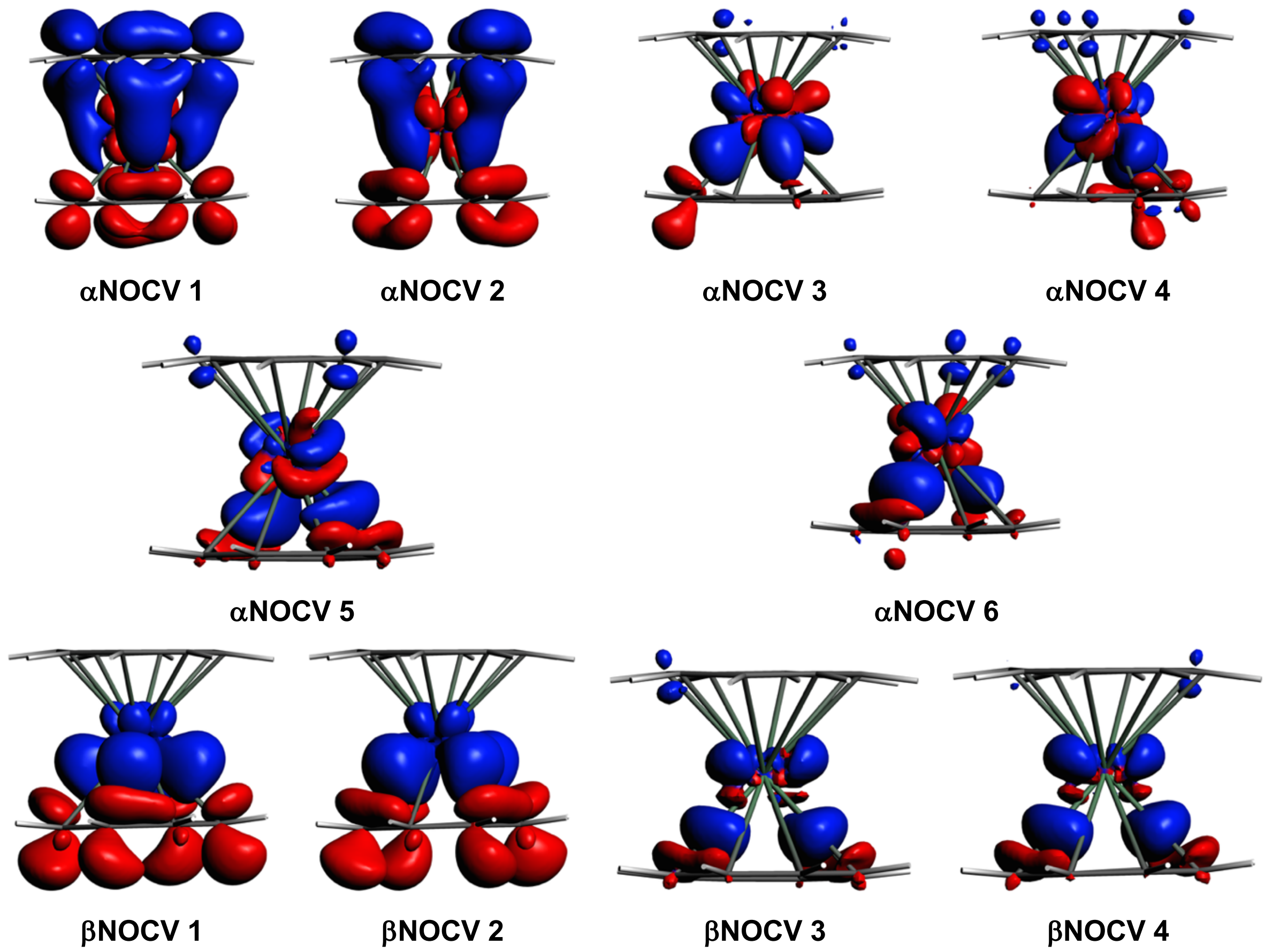

Figure S247. Selected NOCV orbital isosurfaces for **3**. Isosurface values 0.001 a.u. The charge flows from fragments to the full complex from red → blue (view rotated for best angle).

Table S70. NOCV orbital summary for **3** (energies in kJ mol^{-1}).

NOCV #	Eigenvalue	ΔE_{orb} (total)	ΔT_{orb} (kinetic)	ΔV_{orb} (potential)
α1	–0.63	–206.42	–7180.57	6974.15
α2	–0.62	–204.70	–6789.58	6584.88
α3	–0.31	–67.27	306.23	–373.50
α4	–0.30	–68.10	83.48	–151.57
α5	–0.22	–65.07	249.31	–314.38
α6	–0.20	–56.12	–161.27	105.15
β1	–0.45	–155.61	4147.10	–4302.71
β2	–0.45	–153.40	4052.02	–4205.42
β3	–0.23	–72.44	561.72	–634.15
β4	–0.22	–70.10	548.35	–618.45

Table S71. QTAIM localization and delocalization indices, and QTAIM charge and spin, for the U-atoms in complexes **1**–**6**.

Compound M(OS) f^n	Z(III) [A]	LI(A) [B]	N(A) [C]	%Loc(A) [D]	DI(A,A') [E]	%Deloc(A,A') [F]	*Q*(A)	QTAIM spin	∆[Z(III)–LI(A)]
**2** U(III) $5f^3$	89	88.33	90.14	97.99	1.81	2.01	1.86	2.91	0.67
**5** U(III) $5f^3$	89	88.34	90.12	98.02	1.78	1.98	1.88	2.91	0.66
**10** Ce(III) $4f^1$	54	54.68	56.17	97.35	1.49	2.65	1.83	1.02	0.32
**1** U(IV) $5f^2$	88	87.68	89.94	97.49	2.26	2.51	2.06	2.16	0.32
**4** U(IV) $5f^2$	88	87.66	89.91	97.50	2.25	2.50	2.09	2.15	0.34
**9** Ce(IV) $4f^0$	55	53.95	56.09	96.18	2.14	3.82	1.91	–	0.05
**3** U(V) $5f^1$	87	87.18	89.85	97.03	2.67	2.97	2.15	1.38	–0.13
**6** U(V) $5f^1$	87	87.26	89.86	97.11	2.60	2.89	2.14	1.54	–0.26

[A] Formal electron count in atom A for M(III). [B] Average number of electrons localized in atom A. [C] Average number of electrons in atom A. [D] Percentage of average number of electrons in atom a that are localized in atom A. [E] Average number of electrons in atom A that are delocalized between atom A and other atoms in the molecule [N(A)–LI(A)]. [F] Percentage of average number of electrons in atom A shared with other atoms of the molecule.

Table S72. The mean electron density (ρ), Laplacian of the electron density ($\nabla^2\rho$), energy density (H), delocalization index (δ), electronic kinetic energy density (G), and the potential energy density (V) at M–C bond critical points (BCPs) in complexes **1**–**6**.

**Compound M(OS) f^n**	**Metric** [A]	**ρ**	**$\nabla^2\rho$**	**H** [B]	**δ(M,C)**	**G**	**V**	$\left(G/\lvert V\rvert\right)_{BCP}$
**2** U(III) $5f^3$	M–C	0.040(1)	0.115(4)	–0.004(–)	0.218(6)	0.033(1)	–0.036(1)	0.895(5)
**5** U(III) $5f^3$	M–C	0.041(1)	0.114(4)	–0.004(–)	0.220(4)	0.033(1)	–0.037(1)	0.886(6)
**10** Ce(III) $4f^1$	M–C	0.036(–)	0.101(2)	–0.002(–)	0.185(1)	0.028(–)	–0.030(–)	0.920(2)
**1** U(IV) $5f^2$	M–C	0.048(–)	0.126(2)	–0.006(–)	0.272(10)	0.038(–)	–0.044(1)	0.860(3)
**4** U(IV) $5f^2$	M–C	0.048(2)	0.124(5)	–0.006(1)	0.272(10)	0.037(2)	–0.043(2)	0.857(7)
**9** Ce(IV) $4f^0$	M–C	0.042(1)	0.106(–)	–0.004(1)	0.262(6)	0.031(–)	–0.035(1)	0.878(8)
**3** U(V) $5f^1$	M–C	0.054(3)	0.129(5)	–0.008(1)	0.324(13)	0.041(2)	–0.049(3)	0.828(10)
**6** U(V) $5f^1$	M–C	0.052(1)	0.119(4)	–0.008(–)	0.322(4)	0.038(1)	–0.046(2)	0.827(5)

[A] σ is the standard deviation, values all rounded to three significant figures or fewer. Where this results in the standard deviation being more than one order of magnitude smaller than the value, "–" is given instead. [B] Note $H = G + V$.

Table S73. The mean electron density (ρ), Laplacian of the electron density ($\nabla^2\rho$), energy density (H), delocalization index (δ), ellipticity (ε), electronic kinetic energy density (G), and the potential energy density (V) at $C–C_{COT}$ bond critical points (BCPs) in complexes **1**–**6**.

**Compound M(OS) f^n**	**Metric** [A]	**ρ**	**$\nabla^2\rho$**	**H** [B]	**δ(M,C)**	**ε**	**G**	**V**	$\left(G/\lvert V\rvert\right)_{BCP}$
**2** U(III) $5f^3$	$C–C_{COT}$	0.298(6)	–0.81(3)	–0.307(12)	1.328(13)	0.253(3)	0.104(4)	–0.411(15)	0.252(1)
**5** U(III) $5f^3$	$C–C_{COT}$	0.293(6)	–0.78(4)	–0.298(12)	1.329(10)	0.23(3)	0.102(3)	–0.400(14)	0.255(2)
**10** Ce(III) $4f^1$	$C–C_{COT}$	0.294(5)	–0.79(3)	–0.299(8)	1.337(7)	0.23(3)	0.102(2)	–0.401(10)	0.255(2)
**1** U(IV) $5f^2$	$C–C_{COT}$	0.31(2)	–0.88(13)	–0.33(4)	1.32(3)	0.240(3)	0.111(13)	–0.44(6)	0.251(3)
**4** U(IV) $5f^2$	$C–C_{COT}$	0.296(3)	–0.795(19)	–0.303(5)	1.322(13)	0.22(2)	0.104(1)	–0.408(6)	0.256(2)
**9** Ce(IV) $4f^0$	$C–C_{COT}$	0.295(5)	–0.79(3)	–0.300(8)	1.327(11)	0.21(24	0.102(2)	–0.402(9)	0.254(3)
**3** U(V) $5f^1$	$C–C_{COT}$	0.306(11)	–0.86(6)	–0.32(2)	1.31(2)	0.218(4)	0.107(6)	–0.43(3)	0.250(2)
**6** U(V) $5f^1$	$C–C_{COT}$	0.296(5)	–0.80(3)	–0.303(9)	1.31(2)	0.19(3)	0.103(2)	–0.41(1)	0.253(2)

[A] σ is the standard deviation, values all rounded to three significant figures or fewer. Where this results in the standard deviation being more than one order of magnitude smaller than the value, "–" is given instead. [B] Note $H = G + V$.

S10. Computational methodology – CASSCF & RASSCF

General considerations

All simulations were performed using version of 23.02 of the OpenMolcas software suite[55], employing all electron Atomic Natural Orbital (ANO) basis sets of approximate polarised triple-zeta quality for Ce and U (polarising h-functions removed for ease of subsequent analysis), and polarised double-zeta quality for C and H[56-59]. Scalar relativistic effects were captured via the 2nd order Douglas-Kroll-Hess Hamiltonian and spin-orbit coupling (SOC) was incorporated via the Restricted Active Space State Interactions (RASSI) approach. All simulations were performed at the experimentally determined structures of the parent compounds, with H-atom positions optimised at the DFT level of theory. All simulations were performed in the absence of spatial symmetry.

Active spaces

Simulations entailed electronic structure optimisations performed at the state-averaged Restricted Active Space Self-Consistent Field (RASSCF) level of theory followed by simulations of larger state averages at the RAS Configuration Interaction (RASCI) level of theory. All Restricted active spaces (RAS) were characterised as follows: RAS1 – frontier δ-bonding π-orbitals; RAS2 – metal 4*f*/5*f* orbitals (7 orbitals). RAS3 – π-orbitals, 5*d*/6*d* (note: the composition of RAS3 was not maintained throughout the simulations due to near-zero occupation of RAS3 orbitals). A maximum of two holes (electrons) was allowed in RAS1 (RAS3). This RAS can be characterised as RAS(4, 7, 9; *n*, 2, 2), where *n* is dependent on chemical element and oxidations state: Ce(IV): n = 8; Ce(III)/U(V): n = 9; U(IV): n = 10; U(III): n = 11. Selected additional simulations were performed as the complete Active Space SCF (CASSCF) level of theory, incorporating CAS(n,11) active spaces where the CAS was defined by the orbitals comprising RAS1 and RAS2 in RASSCF simulations. Selected simulations employed supersymmetry specifications in attempts to stabilise appropriate active space orbital composition. Dynamical correlation was recovered using multiconfigurational 2nd order perturbation theory, suppressing intruder states via an imaginary level shift of *i*0.2 a.u.

State averaging

RASSCF simulations were performed on the highest spin-state of each system. State-averaging was performed to ensure that all possible $4f^n$/$5f^n$ configurations were equally represented. In the case of the $4f^0$ Ce(IV) compound, both ground state and 28-state averaged (allowing for single excitation from each delta-bonding π-orbitals into each 4*f* orbital) simulations were performed. The same protocol was employed for selected CAS simulations.

Table S74. Summary of state-averages employed in the RASSCF simulations of the high-spin state of each system.

System	RASSCF state average
Ce(III)	7SA (doublet)
Ce(IV)	GS/28SA (singlet)
**2**: U(III)	35SA (quartet)
**1**: U(IV)	21SA (triplet)
**3**: U(V)	7SA (doublet)

The RASSCF state-averaged orbitals were then used as the basis for RASCI simulations of a larger number of excited states of both high and intermediate spin, subsequently used in the simulation of UV-vis spectra.

Electronic structure

Table S75. SOF state-averaged RASSCF π_δ Pseudonatural Occupations of **1–3**, [$Ce^{IV}(COT)_2$], and [$Ce^{III}(COT)_2$]$^-$.

System	π_δ (metal *d*-character)	π_δ (metal *f*-character)
**2**: U(III) – 35SA	1.96, 1.96	1.96, 1.96
**1**: U(IV) – 21SA	1.96, 1.96	1.95, 1.95
**3**: U(V) – 7SA	1.97, 1.97	1.88, 1.88
Ce(III) – 7SA	1.96, 1.96 [B]	1.96, 1.96
Ce(IV) [A] – GS	1.96, 1.95	1.78, 1.82

[A] Results from CAS(8,11) simulation. [B] Supersymmetry employed.

In **Table S75**, deviation of the occupation from 2 in the π_δ-orbital with metal *f*-character reflects the multiconfigurational character exhibited in cerocene, [Ce^{IV}(COT)$_2$], where there is commensurate occupation of the formally antibonding metal-dominated f_δ-orbital (see **Table S76**).

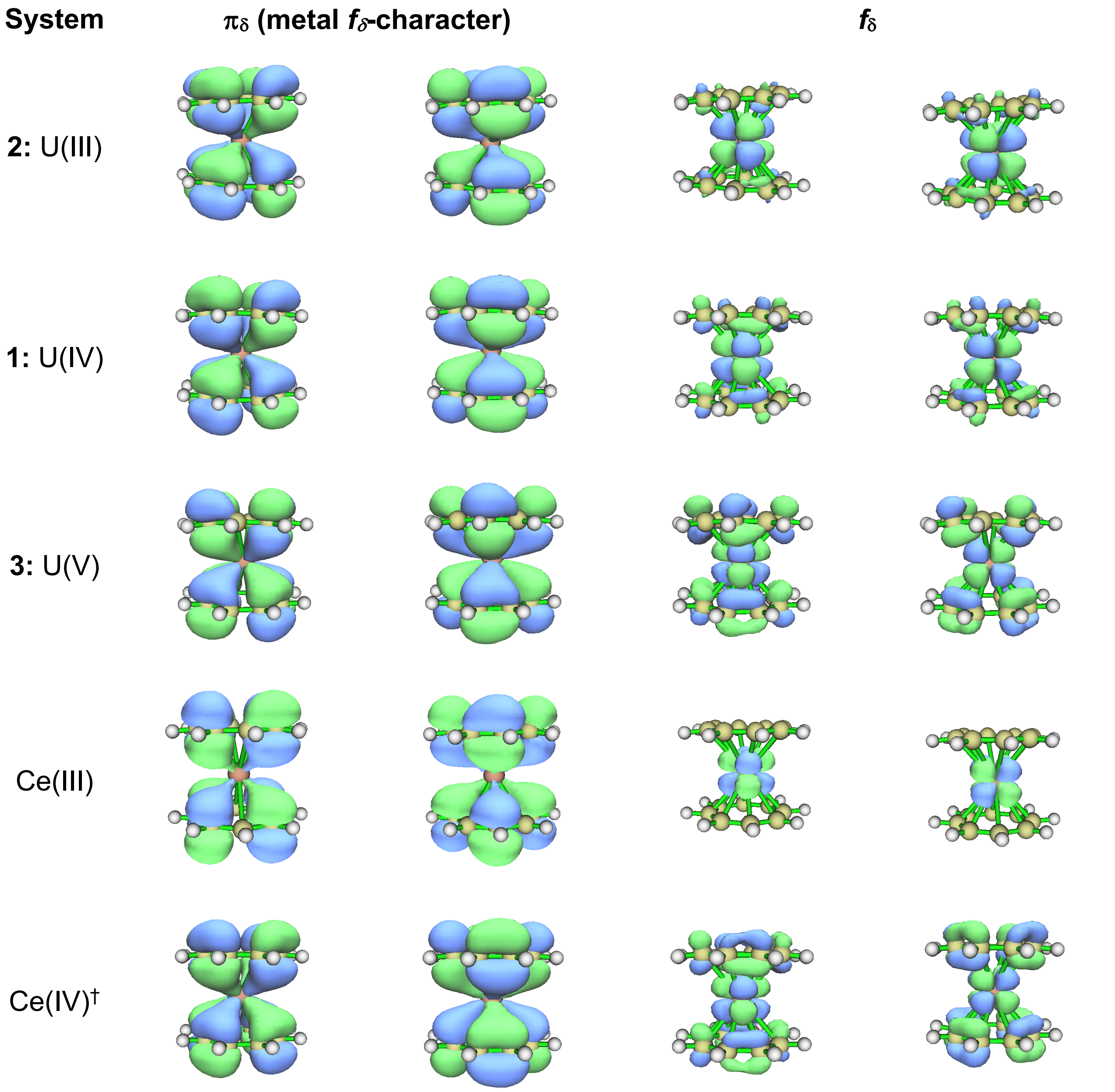

Figure S248. SA-RASSCF pseudonatural orbitals with metal f_δ character of systems studied. *Supersymmetry employed. †Results from ground state CAS(8,11) simulation

Table S76. SOF state-averaged RASSCF π_δ Natural Occupations (from state-averaged calculations) of **1–3**, [Ce^{IV}(COT)$_2$], and [Ce^{III}(COT)$_2$]$^-$. *f*-orbitals occupations are for the strongly occupied orbitals and the metal-dominated f_δ orbitals (latter in bold).

System	π_δ (metal *d*-character)	π_δ (metal *f*-character)	*f*
**2:** U(III)	1.96, 1.96	1.96, 1.96	0.97, 0.97, 0.93, **0.03, 0.03**
**1:** U(IV)	1.96, 1.96	1.95, 1.95	0.97, 0.96, **0.04, 0.04**
**3:** U(V)	1.97, 1.97	1.90, 1.91	1.00, **0.08, 0.08**
Ce(III) [A]	1.96, 1.96	1.96, 1.96	1.00, **0.00, 0.00**
Ce(IV)	1.98, 1.98	1.78, 1.82	**0.21, 0.17**
Ce(IV) [B]	1.96, 1.95	1.78, 1.82	**0.19, 0.15**

[A] Supersymmetry employed. [B] Results from ground state CAS(8,11) simulation.

EPR g-values

Table S77. RASCI-PT2-calculated *g*-values. $g_\perp$ = averaged g_x and g_y values.

System	g_x		g_y		$g_\perp$		g_z	
	Expt.	*AS*	*Expt.*	*AS*	*Expt.*	*AS*	*Expt.*	*AS*
**1:** U(III) [A]	< 0.4	0.01	< 0.4	0.02	< 0.4	0.02	3.34	3.42
**3:** U(V)	2.83	2.86	2.83	2.42	2.83	2.64	1.06	1.12
Ce(III) [B]	2.35	2.33	2.25	2.29	2.3	2.31	1.09	1.11

[A] Experimental data from complex **5**, calculated data from model [U^{III}(COT)$_2$]$^-$. [B] Experimental data from complex **10**, calculated data from model [Ce^{III}(COT)$_2$]$^-$.

Table S78. Method dependence of **3:** U(V) EPR *g*-values.

Method	g_x	g_y	g_z
Expt.	2.83	2.83	1.06
RASCI-PT2(4,7,9,9,2,2): doublets and quartets	2.86	2.42	1.11
CASPT2(9,11): doublets only	2.87	2.78	1.15

Active space orbitals

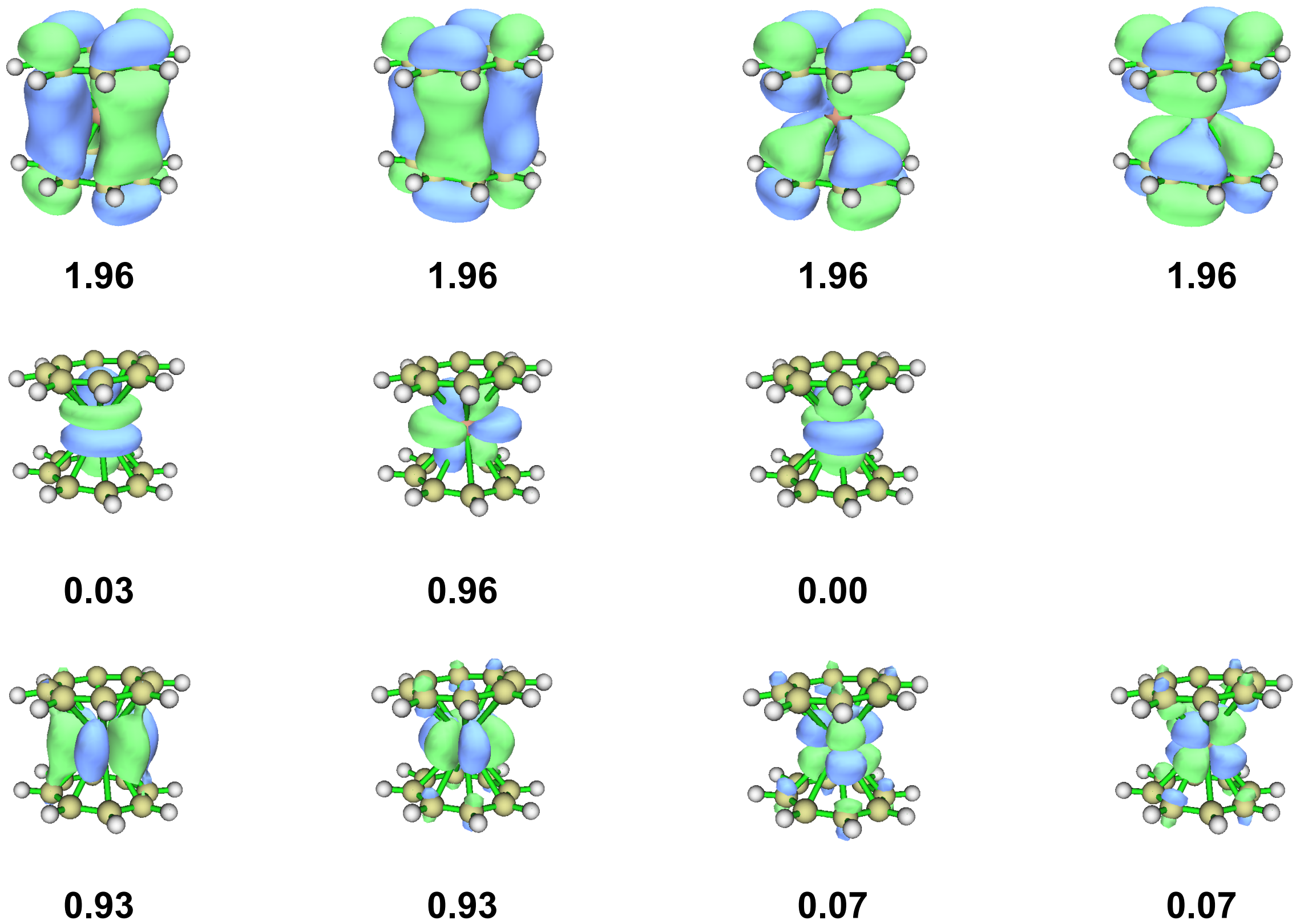

Figure S249. Natural Orbitals of **2:** U(III), obtained as the ground state of a SA-RASSCF simulation.

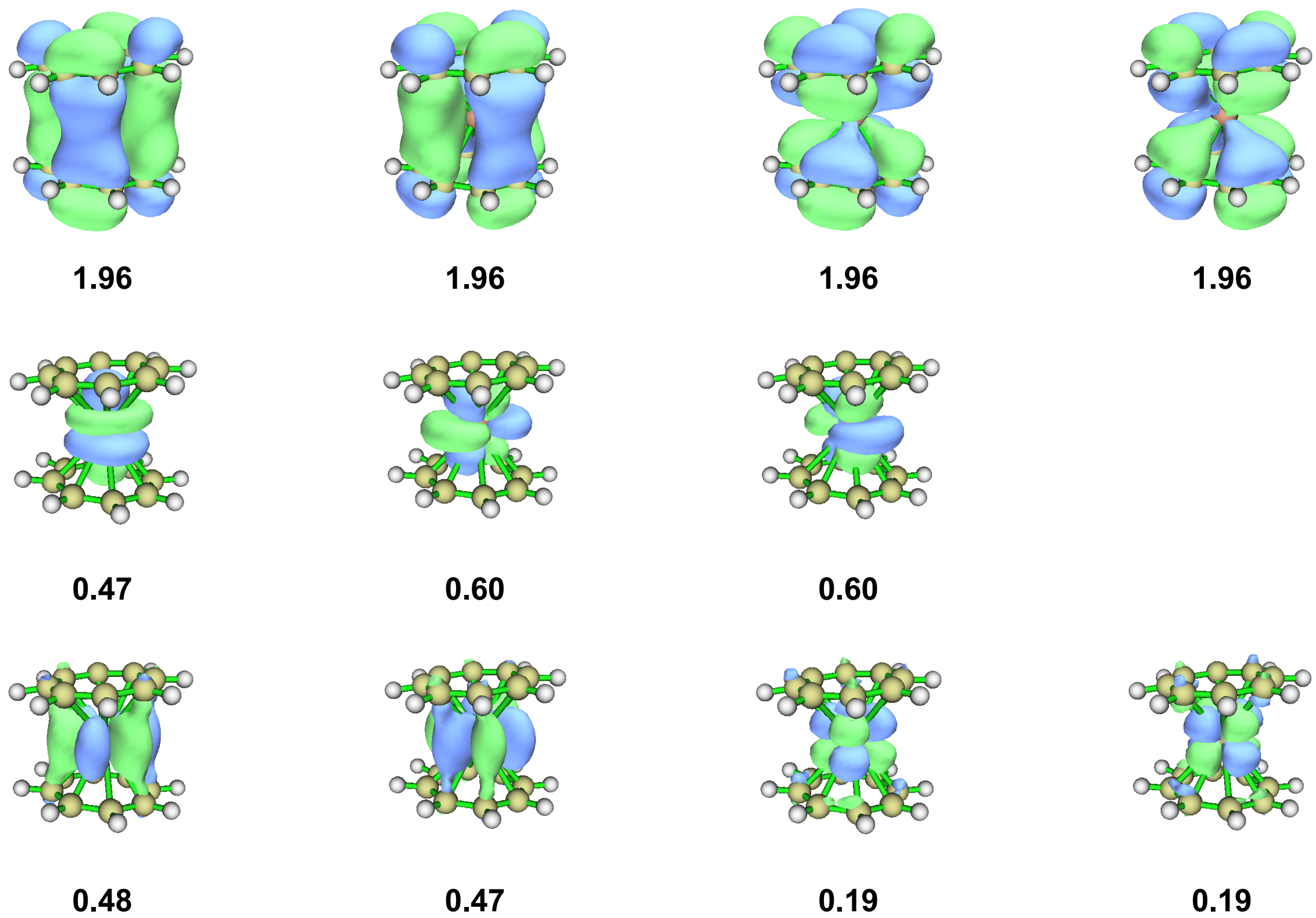

Figure S250. Spin-Orbit Natural Orbitals (SONOs) of **2:** U(III), obtained as the ground state of a SOC RASSI simulation.

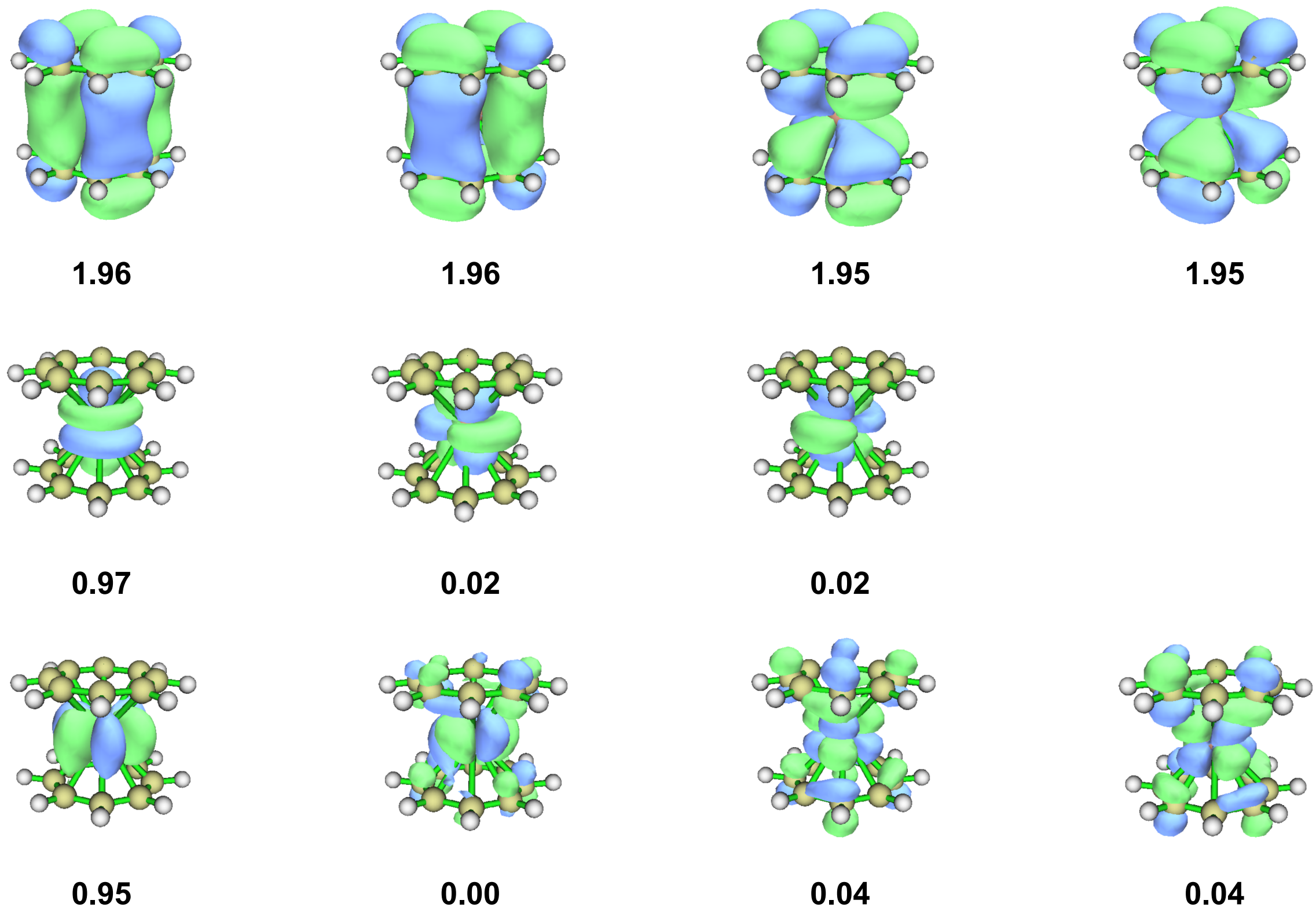

Figure S251. Natural Orbitals of **1:** U(IV), obtained as the ground state of a SA-RASSCF simulation

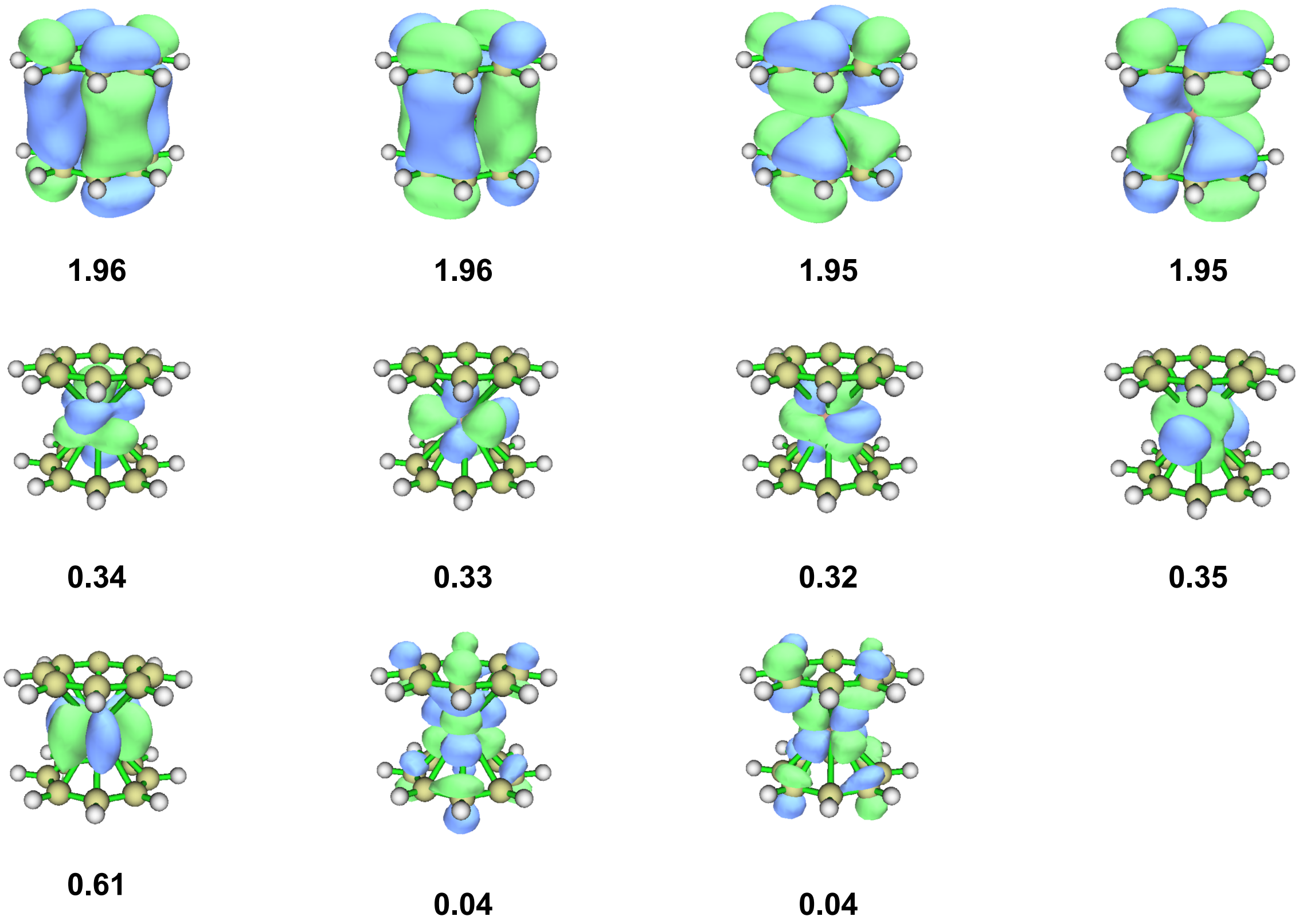

Figure S252. Spin-Orbit Natural Orbitals (SONOs) of **1:** U(IV), obtained as the ground state of a SOC RASSI simulation.

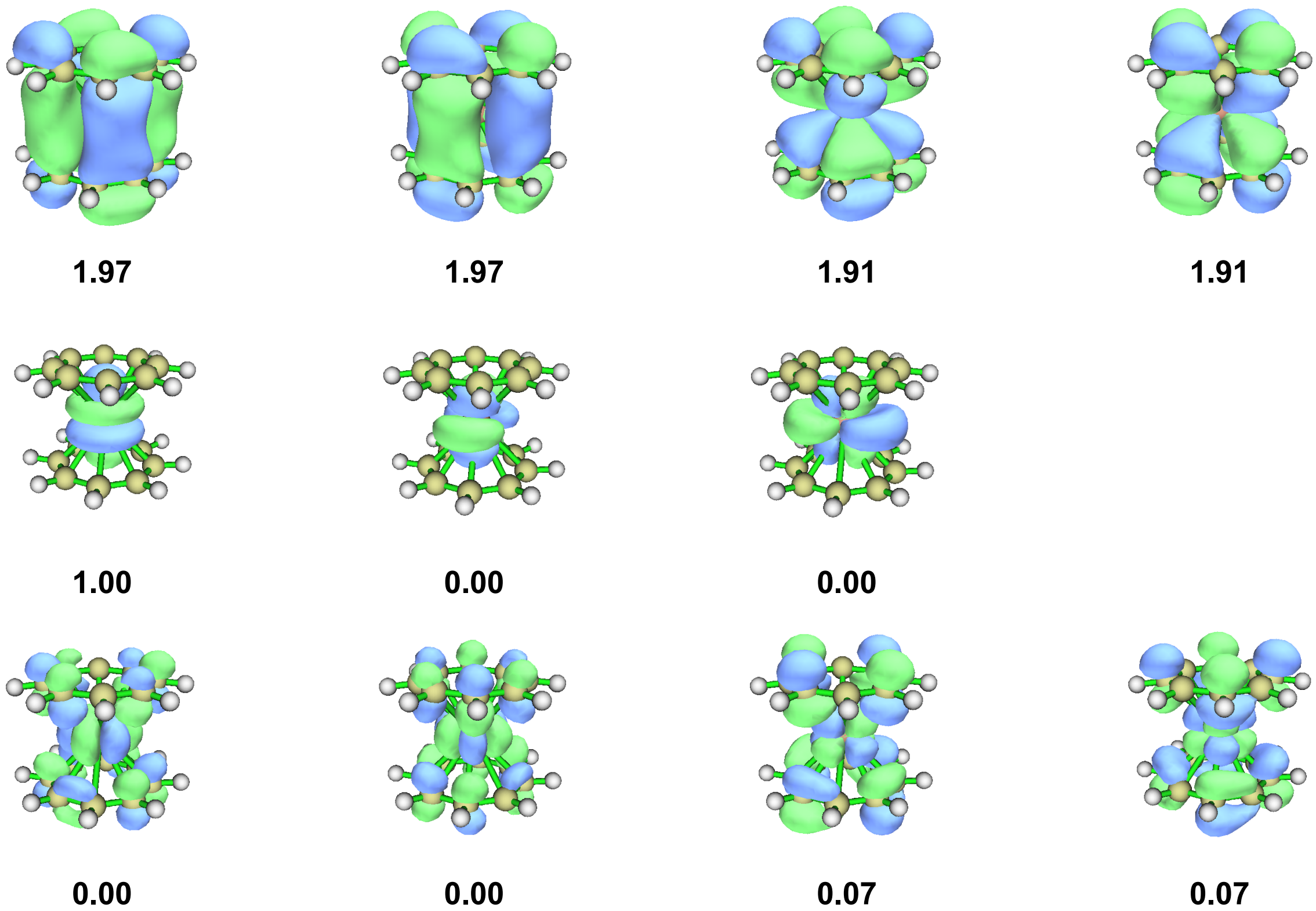

Figure S253. Natural Orbitals of **3:** U(V), obtained as the ground state of a SA-RASSCF simulation.

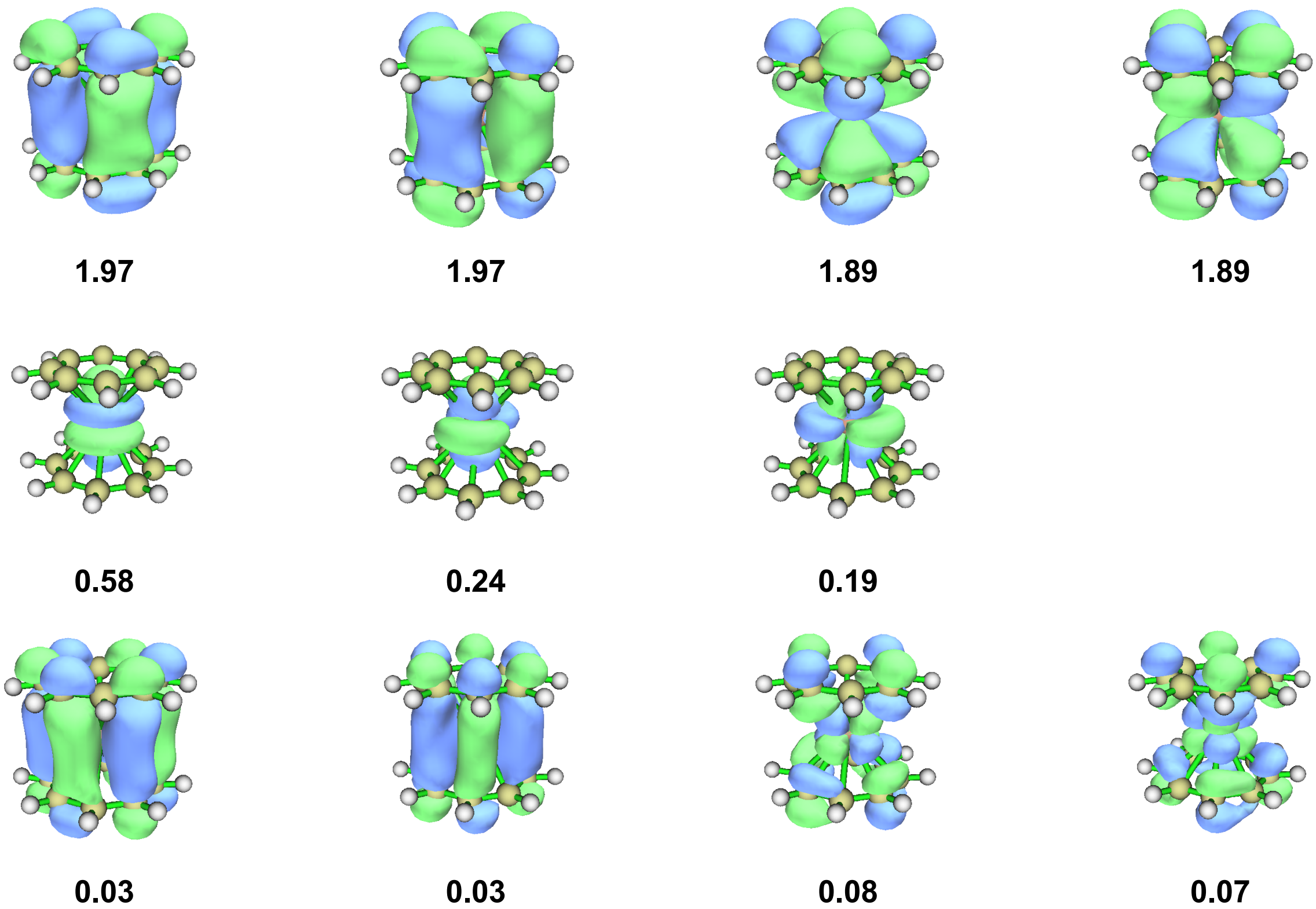

Figure S254. Spin-Orbit Natural Orbitals (SONOs) of **3:** U(V), obtained as the ground state of a SOC RASSI simulation.

Table S79. Integrated QTAIM parameters for **1**–**3** derived from the ground states of SA-RASSCF simulations.

**Compound M(OS) f^n**	**Z(III)** [A]	**LI(A)** [B]	**N(A)** [C]	**%Loc(A)** [D]	**DI(A,A′)** [E]	**%Deloc(A,A′)** [F]	***Q*(A)**	**QTAIM spin**	**Δ[Z(III)–LI(A)]**
**2** U(III) 5f^3	89	88.52	89.94	98.42	2.84	3.16	2.06	–	0.48
**1** U(IV) 5f^2	88	87.94	89.59	98.15	3.30	3.69	2.41	–	0.06
**3** U(V) 5f^1	87	87.49	89.46	97.79	3.95	4.42	2.54	–	–0.49

[A] Formal electron count in atom A for M(III). [B] Average number of electrons localized in atom A. [C] Average number of electrons in atom A. [D] Percentage of average number of electrons in atom a that are localized in atom A. [E] Average number of electrons in atom A that are delocalized between atom A and other atoms in the molecule [N(A)–LI(A)]. [F] Percentage of average number of electrons in atom A shared with other atoms of the molecule.

Table S80. Topological QTAIM parameters for **1**–**3**, as well as the delocalisation index, δ(M,C), derived from the ground states of SA-RASSCF simulations.

**Compound M(OS) f^n**	**Metric** [A]	**ρ**	**$\nabla^2\rho$**	**H** [B]	**δ(M,C)**	**G**	**V**	$\left(G/\lvert V\rvert\right)_{BCP}$
**2** U(III) 5f^3	M–C	0.038(6)	0.125(7)	–0.003(2)	0.171(4)	0.034(6)	–0.037(8)	0.918()
**1** U(IV) 5f^2	M–C	0.048(4)	0.135(6)	–0.007(1)	0.199(7)	0.041(0)	–0.048(2)	0.854(1)
**3** U(V) 5f^1	M–C	0.054(8)	0.130(2)	–0.108(5)	0.240(4)	0.043(4)	–0.054(2)	0.796(2)

[A] σ is the standard deviation, values all rounded to three significant figures or fewer. Where this results in the standard deviation being more than one order of magnitude smaller than the value, "–" is given instead. [B] Note $H = G + V$.

Table S81. % SCPA orbital compositions for **1**–**3**, averaged over C-atoms and electron spins, derived from the ground states of SA-RASSCF simulations.

**Compound M(OS) f^n**	**COT C**	**Metal (5*f*)**	**COT C**	**Metal (6*d*)**
**2** U(III) 5f^3	96.16	3.45	87.01	12.78
**1** U(IV) 5f^2	88.60	11.2	80.15	19.6
**3** U(V) 5f^1	71.18	28.74	73.99	25.69

S11. References

1 Izod, K., Liddle, S. T. & Clegg, W. A Convenient Route to Lanthanide Triiodide THF Solvates. Crystal Structures of $LnI_3(THF)_4$ [Ln = Pr] and $LnI_3(THF)_{3.5}$ [Ln = Nd, Gd, Y]. *Inorg. Chem.* **43**, 214-218 (2004).

2 Patel, D. *et al.* Comments on reactions of oxide derivatives of uranium with hexachloropropene to give UCl_4. *New J. Chem.* **39**, 7559-7562 (2015).

3 Lambert, J. B., Lin, L. & Keinan, S. The $C_5SiMe_7^+$ cation: pyramidal, bicyclic, or cyclohexadienyl? *Org. Biomol. Chem.* **1**, 2559-2565 (2003).

4 Summerscales, O. T., Cloke, F. G. N., Hitchcock, P. B., Green, J. C. & Hazari, N. Reductive Cyclotrimerization of Carbon Monoxide to the Deltate Dianion by an Organometallic Uranium Complex. *Science* **311**, 829-831 (2006).

5 Münzfeld, L. *et al.* Introduction of plumbole to f-element chemistry. *Chem. Sci.* **13**, 945-954 (2022).

6 Streitwieser, A., Jr. & Mueller-Westerhoff, U. Bis(cyclooctatetraenyl)uranium (Uranocene). A New Class of Sandwich Complexes That Utilize Atomic f Orbitals. *J. Am. Chem. Soc.* **90**, 7364-7364 (1968).

7 Rigaku Oxford Diffraction, (2023), CrysAlisPro Software system, Rigaku Corporation, Wroclaw, Poland. (Rigaku Oxford Diffraction, 2023).

8 Dolomanov, O. V., Bourhis, L. J., Gildea, R. J., Howard, J. A. K. & Puschmann, H. OLEX2: a complete structure solution, refinement and analysis program. *J. Appl. Crystallogr.* **42**, 339-341 (2009).

9 Sheldrick, G. M. Crystal structure refinement with SHELXL. *Acta Crystallogr., Sect. C* **71**, 3-8 (2015).

10 Sheldrick, G. M. A short history of SHELX. *Acta Crystallogr., Sect. A* **64**, 112-122 (2008).

11 Persistence of Vision Raytracer (Version 3.6) (Persistence of Vision Pty. Ltd., 2004).

12 GNU Image Manipulation Program (GIMP) (Version 2.10.38) (The GIMP Team, 2024).

13 Inkscape: Open Source Scalable Vector Graphics Editor. https://inkscape.org/.

14 Clegg, W. *et al. Crystal Structure Analysis*. 2nd edn, (Oxford University Press, 2009).

15 Kabova, E. A. *et al.* SDPD-SX: combining a single crystal X-ray diffraction setup with advanced powder data structure determination for use in early stage drug discovery. *CrystEngComm* **24**, 4337-4340 (2022).

16 Coelho, A. An indexing algorithm independent of peak position extraction for X-ray powder diffraction patterns. *J. Appl. Crystallogr.* **50**, 1323-1330 (2017).

17 Petříček, V., Dušek, M. & Palatinus, L. Crystallographic Computing System JANA2006: General features. *Z. Kristallogr. - Cryst. Mater.* **229**, 345-352 (2014).

18 Eisenberg, D. C., Streitwieser, A., Jr. & Kot, W. K. Electron transfer in organouranium and transuranium systems. *Inorg. Chem.* **29**, 10-14 (1990).

19 Edelstein, N., Lamar, G. N., Mares, F. & Streitwieser, A. Proton magnetic resonance shifts in (bis(cyclooctatetraenyl)uranium(IV). *Chem. Phys. Lett.* **8**, 399-402 (1971).

20 Kuppuswamy, S. *et al.* One-Electron Oxidation Chemistry and Subsequent Reactivity of Diiron Imido Complexes. *Inorg. Chem.* **53**, 5429-5437 (2014).

21 Aranzaes, J. R., Daniel, M.-C. & Astruc, D. Metallocenes as references for the determination of redox potentials by cyclic voltammetry — Permethylated iron and cobalt sandwich complexes, inhibition by polyamine dendrimers, and the role of hydroxy-containing ferrocenes. *Can. J. Chem.* **84**, 288-299 (2006).

22 Goodwin, C. A. P. *et al.* Isolation and electronic structures of derivatized manganocene, ferrocene and cobaltocene anions. *Nat. Chem.* **13**, 243-248 (2021).

23 Singh, S. K., Cramer, C. J. & Gagliardi, L. Correlating Electronic Structure and Magnetic Anisotropy in Actinide Complexes $[An(COT)_2]$, $An^{III/IV}$ = U, Np, and Pu. *Inorg. Chem.* **59**, 6815-6825 (2020).

24 Gendron, F., Pritchard, B., Bolvin, H. & Autschbach, J. Single-ion 4f element magnetism: an ab-initio look at $Ln(COT)_2^-$. *Dalton Trans.* **44**, 19886-19900 (2015).

25 Solis-Céspedes, E. & Páez-Hernández, D. Modeling the electronic states and magnetic properties derived from the f^1 configuration in lanthanocene and actinocene compounds. *Dalton Trans.* **46**, 4834-4843 (2017).

26 Stoll, S. & Schweiger, A. EasySpin, a comprehensive software package for spectral simulation and analysis in EPR. *J. Magn. Reson.* **178**, 42-55 (2006).

27 MATLAB v. R2025b (The Mathworks, Inc., Massachusetts).

28 Abragam, A. & Bleaney, B. *Electron Paramagnetic Resonance of Transition Ions*. (Clarendon P., 1970).

29 Gourier, D., Caurant, D., Arliguie, T. & Ephritikhine, M. EPR and Angle-Selected ENDOR Study of 5f-Ligand Interactions in the $[U(\eta^7\text{-}C_7H_7)_2]^-$ Anion, an f^1 Analogue of Uranocene. *J. Am. Chem. Soc.* **120**, 6084-6092 (1998).

30 Neese, F. Software Update: The ORCA Program System—Version 6.0. *WIREs Comput. Mol. Sci.* **15**, e70019 (2025).

31 Perdew, J. P., Ernzerhof, M. & Burke, K. Rationale for mixing exact exchange with density functional approximations. *J. Chem. Phys.* **105**, 9982-9985 (1996).

32 Adamo, C. & Barone, V. Toward reliable density functional methods without adjustable parameters: The PBE0 model. *J. Chem. Phys.* **110**, 6158-6170 (1999).

33 Grimme, S., Ehrlich, S. & Goerigk, L. Effect of the damping function in dispersion corrected density functional theory. *J. Comput. Chem.* **32**, 1456-1465 (2011).

34 Neese, F., Wennmohs, F., Hansen, A. & Becker, U. Efficient, approximate and parallel Hartree–Fock and hybrid DFT calculations. A 'chain-of-spheres' algorithm for the Hartree–Fock exchange. *Chem. Phys.* **356**, 98-109 (2009).

35 Izsak, R. & Neese, F. An overlap fitted chain of spheres exchange method. *J. Chem. Phys.* **135**, 144105 (2011).

36 Peng, D. & Reiher, M. Local relativistic exact decoupling. *J. Chem. Phys.* **136**, 244108 (2012).

37 Pollak, P. & Weigend, F. Segmented Contracted Error-Consistent Basis Sets of Double- and Triple-ζ Valence Quality for One- and Two-Component Relativistic All-Electron Calculations. *J. Chem. Theory Comput.* **13**, 3696-3705 (2017).

38 Franzke, Y. J., Treß, R., Pazdera, T. M. & Weigend, F. Error-consistent segmented contracted all-electron relativistic basis sets of double- and triple-zeta quality for NMR shielding constants. *Phys. Chem. Chem. Phys.* **21**, 16658-16664 (2019).

39 Pritchard, B. P., Altarawy, D., Didier, B., Gibson, T. D. & Windus, T. L. New Basis Set Exchange: An Open, Up-to-Date Resource for the Molecular Sciences Community. *J. Chem. Inf. Model.* **59**, 4814-4820 (2019).

40 Schuchardt, K. L. *et al.* Basis Set Exchange: A Community Database for Computational Sciences. *J. Chem. Inf. Model.* **47**, 1045-1052 (2007).

41 Feng, R. & Peterson, K. A. Correlation consistent basis sets for actinides. II. The atoms Ac and Np-Lr. *J. Chem. Phys.* **147**, 084108 (2017).

42 Stoychev, G. L., Auer, A. A. & Neese, F. Automatic Generation of Auxiliary Basis Sets. *J. Chem. Theory Comput.* **13**, 554-562 (2017).

43 Baerends, E. J. *et al.* The Amsterdam Modeling Suite. *J. Chem. Phys.* **162**, 162501 (2025).

44 Michalak, A., DeKock, R. L. & Ziegler, T. Bond Multiplicity in Transition-Metal Complexes: Applications of Two-Electron Valence Indices. *The Journal of Physical Chemistry A* **112**, 7256-7263 (2008).

45 Nalewajski, R. F., Mrozek, J. & Michalak, A. Two-electron valence indices from the Kohn-Sham orbitals. *Int. J. Quantum Chem.* **61**, 589-601 (1997).

46 Mitoraj, M. P., Michalak, A. & Ziegler, T. A Combined Charge and Energy Decomposition Scheme for Bond Analysis. *J. Chem. Theory Comput.* **5**, 962-975 (2009).

47 Sagan, F. & Mitoraj, M. P. Kinetic and Potential Energy Contributions to a Chemical Bond from the Charge and Energy Decomposition Scheme of Extended Transition State Natural Orbitals for Chemical Valence. *J. Phys. Chem. A* **123**, 4616-4622 (2019).

48 Caldeweyher, E. *et al.* A generally applicable atomic-charge dependent London dispersion correction. *J. Chem. Phys.* **150** (2019).

49 van Lenthe, E. & Baerends, E. J. Optimized Slater-type basis sets for the elements 1-118. *J. Comput. Chem.* **24**, 1142-1156 (2003).

50 van Lenthe, E., Baerends, E. J. & Snijders, J. G. Relativistic regular two-component Hamiltonians. *J. Chem. Phys.* **99**, 4597-4610 (1993).

51 van Lenthe, E., Baerends, E. J. & Snijders, J. G. Relativistic total energy using regular approximations. *J. Chem. Phys.* **101**, 9783-9792 (1994).

52 AIMAll v. Version 19.10.12 (TK Gristmill Software, Overland Park KS, USA, 2019).

53 Glendening, E. D., Landis, C. R. & Weinhold, F. NBO 7.0: New vistas in localized and delocalized chemical bonding theory. *J. Comput. Chem.* **40**, 2234-2241 (2019).

54 Chemcraft - graphical software for visualization of quantum chemistry computations v. 1.8, build 770 (2025).

55 Li Manni, G. *et al.* The OpenMolcas Web: A Community-Driven Approach to Advancing Computational Chemistry. *J. Chem. Theory Comput.* **19**, 6933-6991 (2023).

56 Widmark, P.-O., Malmqvist, P.-Å. & Roos, B. O. Density matrix averaged atomic natural orbital (ANO) basis sets for correlated molecular wave functions. *Theor. Chim. Acta* **77**, 291-306 (1990).

57 Roos, B. O., Lindh, R., Malmqvist, P.-Å., Veryazov, V. & Widmark, P.-O. Main Group Atoms and Dimers Studied with a New Relativistic ANO Basis Set. *J. Phys. Chem. A* **108**, 2851-2858 (2004).

58 Roos, B. O. *et al.* New Relativistic Atomic Natural Orbital Basis Sets for Lanthanide Atoms with Applications to the Ce Diatom and LuF_3. *J. Phys. Chem. A* **112**, 11431-11435 (2008).

59 Roos, B. O., Lindh, R., Malmqvist, P.-Å., Veryazov, V. & Widmark, P.-O. New relativistic ANO basis sets for actinide atoms. *Chem. Phys. Lett.* **409**, 295-299 (2005).
