# Supplementary material for: Isolation of uranocenium, the bis-[8]annulene uranium cation
Source: Nat Commun. 2026 Oct 23;17:10914. doi: 10.1038/s41467-026-77066-2 (PMC13694272; doi:10.1038/s41467-026-77066-2)
Supplement: Supplementary file 2 [file 41467_2026_77066_MOESM2_ESM.pdf]

nature portfolio

Peer Review File

Isolation of Uranocenium, the bis-[8]annulene Uranium Cation

Corresponding Author: Dr Conrad Goodwin

This file contains all reviewer reports in order by version, followed by all author rebuttals in order by version.

Version 0:

Reviewer comments:

Reviewer #1

(Remarks to the Author)
The authors provide a well written manuscript reporting uranocenium cations of classical [U(COT)2]+ and substituted [U(COT††)2]+. Experiments were performed at high standards and described and interpreted with a high degree of detail. After a long hunt in the actinide coordination chemistry community, it is indeed an impactful contribution that the uranocene/uranocenium couple is now fully characterized. The authors even went to full length of elaborating side reactions of the oxidized species with PF6 electrolyte ions through detailed control reactions and give very detailed comments on their data interpretation in a solid supplementary information file. I consider this work suitable for publication in Nature Communications and recommend to accept the manuscript for publication with minor changes in response to comments listed below.

1) Considering that oxidation and reduction waves of 4 different compounds are reported in the main text, it may be beneficial to include a table reporting the half wave potentials.
In that context, the authors list the literature value of uranocene oxidation at -0.13V vs Fc+/0 in dichloromethane. This appears to be considerably milder than the ca -0.33V of the uranocene oxidation in DFB (or -0.28V in THF) that the authors measure. I therefore consulted the cited reference #23 (J. Am. Chem. Soc. 1978), finding that it only reports electrochemistry in benzonitrile solvent but mentions in a footnote that data in THF, DCM, and MeCN gave "qualitatively similar" results. Reference #24 (J. Organomet. Chem. 1980) then actually lists values in THF, DCM, MeCN, and benzonitrile, but with all data referenced vs. SCE reference. Which conversion factor did the authors use to translate the literature values into potentials vs. Fc+/0 and how did they determine half-wave potentials given that Table 1 only reports oxidation peak potentials. Maybe it is worth revisiting this value of -0.13V the authors currently state, to make sure the reported numbers are trustworthy. I do belief the new data reported by the authors of this manuscript is accurate and I am doubting that solvent effects between DCM of the literature values and DFB or THF of the new work would be so severe to explain a half wave potential shift of 200mV.

2) On Page 4 line 85, the authors state that complexes 3 and 6 “catalyse” the polymerization of THF. I would like to ask the authors to reconsider the statement. Probably the complexes are only initiating the THF polymerization and are not continuously catalysing it?

3) On page 4 line 102, the authors mention previous works that showed irreversible redox waves for 1 and 3 when using TBAPF6, but the reference to the mentioned works is not given there.

4) On page 9 lines 223 – 225 the authors characterize the U(V) complexes 3 and 6 as 5f1 centres, yet in the electronic structure calculations it is elaborated that the HOMOs are involved in delta bonding, and hence show some considerable mixing with ligand orbitals in their character. In that context, could the authors comment how such delta bonding is expected to contribute deviations from theoretically expected EPR signals of a uranocenium f1 system without delta bonding (without orbital mixing)?

5) On a related note to comment 4, Figure S170 of the supplementary information clearly visualizes delta bonding for the HOMO (alpha-MO 101), whereas for the HOMO of compound 6 (alpha-MO 277) there seems to be no visual orbital overlap of a delta bond between the uranium centre and the ligand. Yet on page 10, line 257 of the main text the authors state the HOMO of compound 6 contributes considerably (>40%) to delta-bonding. Could the authors explain why delta bonding

interactions are numerically described quite similar for complexes 3 and 6 (5f contributions of 42.7% and 43.0% for 3) while the graphical representations of alpha-MOs in the supplementary information gives a very different impression?

Further minor comments regarding the supplementary information file are:

i) In Figure S2, it seems strange that there are unproportionally tiny C-H stretches compared to all other (closely related) compounds of the study. Could the authors revisit, whether the data set is of appropriate quality?

ii) For completeness and as a courtesy to future readers, who may seek to find trends across the nice series of reported complexes, it may be useful to also include an IR spectrum of complex 1 to complete the series of data on all compounds in this study.

iii) Also, for the solution phase UV-Vis-NIR spectroscopy data, a complete and comparable series is reported for complexes 4, 5, and 6, but for the unsubstituted uranocene the analogous data set for complex 1 is missing and only data on 2, and 3 are reported. In order to allow readers to draw conclusions easily from data sets that nicely match conditions, it may be appreciated to also report a comparable UV-Vis-NIR data set for 1 at analogous ranges and concentrations.

iv) Comparing Figures S41 and S43, it seems the reported t1/2 value is almost identical for complexes 3 (21.4 min) and 6 (24.8 min). Yet the straight portion of the graph, on which the linear regression was performed, is ca. 2.7 times as longer for 6 for than for complex 3. Do the authors have a hypothesis, why it was possible to monitor this very similar reaction so much longer for complex 6, despite the quite comparable reaction rates?

v) According to Figure S135 and the quite reversible reduction feature at -3.16 V, it appears the authors should be able to synthesize and possibly isolate a doubly reduced complex with a U(II) centre. It is by no means requested to include such efforts into the manuscript presented here, which is already complete in its outline. Yet, I did not want to leave the opportunity uncommented that the authors may want to look into this possibility for future work.

Reviewer #2

(Remarks to the Author)
The manuscript by Deaking et al. reports the synthesis and initial characterization of the quite rare pentavalent uranocenium cation (5f1 configuration). This species is of high interest to the organometallic chemistry community since it completes a classical sandwich series with an actinide in the +3, +4 and +5 oxidation state. This is long sought in testing the variation in 5f versus 6d bonding and the community will be quite excited to see it finally reported. The topic is well suited for publication un the prestigious Nature Communications and I recommend it with the following comments and suggestions.

Here, the authors used several approaches to probe the 5f1 configuration of uranocenium, as well as family members with U(III) and U(IV), such as EPR, magnetic susceptibility, voltammetry and electronic structure calculations. All data is consistent with the U(V) assignment and the axial (substituted uranocenium 3) or near-axial (6) g-tensor with g values in the range of ~2.8 (g⊥) and ~1.1 (g‖) support the notorious electronic ground state originating from the j=5/2, mj=±1/2 free ion level. The EPR data is quantitatively reproduced by spin-orbit state wavefunction calculations. To this end, I think it would be great to throw a comparison, or at least mention the pentavalent U(C7H7)2- complex made by Didier Gourier in ’98 (J. Am. Chem. Soc. 1998, 120, 24, 6084–6092). The g-factors for the uranocenium are aligned with those for U(C7H7)2- (2.365 and 1.244), both complexes exhibit f -f mixing due to SOC, and both likely show similar bonding within the delta symmetry.

Using a variety of calculations (MO analyses, QTAIM and energy decomposition analyses), the authors show that f-bonding in uranocenium is larger than in U(IV) and U(III) analogues despite there being almost no changes in geometrical parameters. I noted plenty of additional calculations reported in the computational details section (with data present in the SI document) but these are not actually indented anywhere in the manuscript: ETS-NOCV and NLMO analyses, MO diagrams with plenty of DFT functionals such as r2SCAN and variants, etc. Should these be indented in the main manuscript or removed?
A comment concerns the sentence on line 229. Can the authors cross-check if what they mean is that uranocene has 2 electrons in the metal φ-orbitals in a configuration calculated with DFT? Otherwise, it is likely that the two electrons of the U(IV) uranocene are in fσ/fπ or fφ/fπ (or combination of these), at least in the spin-free formalism. With SOC it could be a bigger mess, because electron correlation deems the valence configurations of uranocene near-degenerate and the two unpaired electrons become smeared across the 5f orbitals.

Other comments: 1) Cross-check Figur3. A subfigure 3d is referred to in the manuscript but such figure does not exist or the label is missing (I think 3d refers to the two EPR curves at bottom of Figure 3c) . 2) A 2 subscript is missing in the chemical formula on line 202. 3) On line 209, I suggest changing “pure |mj|>1/2 KD” to “pure KD with |mj|>1/2” to avoid someone trying to understand what is KD that gets multiplied into the 1/2 factor.

Dr. Dumitru-Claudiu Sergentu (UAIC)

Reviewer #3

(Remarks to the Author)
This manuscript describes the synthesis and structural characterization of a pair of uranocenes in three oxidation states; the oxidized U(V) cation is of particular interest. The introduction covers the background and rationale for the work very well and

the collection, presentation and analysis of the data is very professional. Indeed, I find the science to be of high-quality and carefully completed. However, in my opinion the impact of the manuscript in its current form doesn't quite reach the requisite level of Nature Communications, as explained below.

The authors have provided a large amount of data but have not convinced this reader that their results are groundbreaking. Clearly it was initially challenging to obtain the structural information for the uranocenium species reported; the use of the Ph3C[B(C6F5)4] reagent in oDFB solvent is a nice route to generate the target species, and it is thus surprising that the final products are, in fact, quite stable in a fairly wide range of solvents and in the solid-state. More importantly, the careful assessment of the source of the previously reported instability of the in situ generated uranocenium cations illustrated that the use of PF6-based electrolytes was responsible for the decomposition of the complex - this is quite nice to see (note that this been observed in other transition-metal systems such as Fe(III) and Mn(III)), and raises several other questions: Does NaSbF6 also F-transfer - this is a common route to avoiding fluoride transfer from these reagents? Are perchlorate salts (often used as electrolytes) also sources of decomposition via a different route? Do other oxidizing agents in oDFB still generate complex mixtures of products? Why (or is) oDFB a uniquely important solvent to facilitate the oxidation reaction?

It is also interesting that there is apparently increased covalency in the U(V) species rather than in the U(III) species, which is perhaps counterintuitive (the manuscript should be examined by a heavy-metal/relativistic computational expert if it hasn't already been). It would be worth investigating this point computationally in a more general sense in other actinide species. As well, given the apparent stability of the uranocenium species, illustrating some of the potentially unique reactivity of this U(V) species (even in limited broad survey form), and a further discussion of under what conditions it decomposes would increase the impact of this manuscript.

As is, however, the manuscript is not of sufficient broad scope to warrant publication in Nature Communications in my view and could be published in a more specialized chemistry journal such as RSC Chemical Science. I would encourage the authors to include a survey of some reactivity of the title uranocenium species and compare it with the parent uranocene; this addition would, in my view, elevate this exciting work into Nature Communications.

Version 1:

Reviewer comments:

Reviewer #1

(Remarks to the Author)
The authors provide a comprehensive and convincing response letter that addresses all comments adequately. The manuscript is now even more clear and convincing than it was in its original version and I have no further comments or concerns. Accordingly, I recommend to accept the revised version for publication.

Reviewer #2

(Remarks to the Author)
Deakin et al. have revised their manuscript on the synthesis and electronic structure of uranocenium in response to the comments and remarks from me and the other two reviewers. I am overall pleased with their responses, and I reiterate that this is a timely manuscript reporting a formal U(V) f1 complex of significant interest to the wider public working with the f-elements. Two aspects are, in my opinion, still not fully resolved and I recommend considering them in a minor revision.

a) It is now nice to see a comparison with the formal U(V) complex by Gourier, U(C7H7)2^-, but I find this comparison not complete. For instance, despite being formally 5f1, this complex is generally accepted as a U(III) complex due to large bonding involving the 5f_delta orbitals. See for instance: J. Am. Chem. Soc. 1997, 119, 9021; Chem. Sci., 2018,9, 6292; Chem. Commun., 2021,57, 9562. In this context, should the uranocenium also be regarded a U(III)? This kind of comparison would also complete comment 4 from reviewer 1.

b) I had initially a comment on the two electrons in the ground state of uranocene being distributed both in the 5f_phi orbitals. The authors did clarify that this is indeed an assignment from the perspective of DFT calculations and provided two prior citations in support. DFT clearly gives the wrong assignment, since high level calculations, MCSCF and CASPT2//CASSCF (some which were performed by authors here), provide the (f_pi^1)(f_phi^1) distribution of the 2 electrons: J. Phys. Chem. A, 113, 8737 (2009); Liu, Dolg & Fulde, J. Chem. Phys., 107, 3584 (1997).
I find it necessary to at least mention here that these DFT ground state configurations are in disagreement with data provided with more advanced theoretical treatments.

DCS, UAIC.

Reviewer #3

(Remarks to the Author)
The authors have suitably addressed the points of the referees in this revision and I thank them for including substantial

additional data and some commentary regarding reactivity. I am happy to recommend publication in Nature Communications.

Open Access This Peer Review File is licensed under a Creative Commons Attribution 4.0 International License, which permits use, sharing, adaptation, distribution and reproduction in any medium or format, as long as you give appropriate credit to the original author(s) and the source, provide a link to the Creative Commons license, and indicate if changes were made.
In cases where reviewers are anonymous, credit should be given to 'Anonymous Referee' and the source.
The images or other third party material in this Peer Review File are included in the article's Creative Commons license, unless indicated otherwise in a credit line to the material. If material is not included in the article's Creative Commons license and your intended use is not permitted by statutory regulation or exceeds the permitted use, you will need to obtain permission directly from the copyright holder.
To view a copy of this license, visit https://creativecommons.org/licenses/by/4.0/

Response to reviewer comments on NCOMMS-26-019450-T
"Uranocenium – The *bis*-[8]annulene Uranium Cation"

Reviewer 1

Comment:

The authors provide a well written manuscript reporting uranocenium cations of classical [U(COT)2]+ and substituted [U(COT††)2]+. Experiments were performed at high standards and described and interpreted with a high degree of detail. After a long hunt in the actinide coordination chemistry community, it is indeed an impactful contribution that the uranocene/uranocenium couple is now fully characterized. The authors even went to full length of elaborating side reactions of the oxidized species with PF6 electrolyte ions through detailed control reactions and give very detailed comments on their data interpretation in a solid supplementary information file. I consider this work suitable for publication in Nature Communications and recommend to accept the manuscript for publication with minor changes in response to comments listed below.

Response:

We thank the reviewer for their time and for the warm appraisal of the work. Below we provide our responses to their queries, which have been addressed in full.

Comment:

1) Considering that oxidation and reduction waves of 4 different compounds are reported in the main text, it may be beneficial to include a table reporting the half wave potentials.

Response:

We have added a table to the main text that gives the U(IV/III) and U(V/IV) couples for **1**, **4**, **3**, and **6** as well as the relevant E_{pa}, E_{pc}, ΔE, and I_{pa}/I_{pc} values (all at 200 mV/s) so the reader is able to fully interpret the results.

Comment:

In that context, the authors list the literature value of uranocene oxidation at -0.13V vs Fc+/0 in dichloromethane. This appears to be considerably milder than the ca -0.33V of the

uranocene oxidation in DFB (or -0.28V in THF) that the authors measure. I therefore consulted the cited reference #23 (J. Am. Chem. Soc. 1978), finding that it only reports electrochemistry in benzonitrile solvent but mentions in a footnote that data in THF, DCM, and MeCN gave "qualitatively similar" results. Reference #24 (J. Organomet. Chem. 1980) then actually lists values in THF, DCM, MeCN, and benzonitrile, but with all data referenced vs. SCE reference. Which conversion factor did the authors use to translate the literature values into potentials vs. Fc+/0 and how did they determine half-wave potentials given that Table 1 only reports oxidation peak potentials. Maybe it is worth revisiting this value of -0.13V the authors currently state, to make sure the reported numbers are trustworthy. I do belief the new data reported by the authors of this manuscript is accurate and I am doubting that solvent effects between DCM of the literature values and DFB or THF of the new work would be so severe to explain a half wave potential shift of 200mV.

Response:

We really appreciate the detective work by the reviewer here as we were not sufficiently clear in the text for this part. The value we gave was for the oxidation wave, not for a redox couple. The text has been amended to clarify this. Furthermore, we have now been more explicit about how we arrived at –0.13 V vs Fc by also reporting the original value vs SCE, and giving the reference used for the correction factor. Geiger and Connelly reported Fc as being at +0.46 V vs SCE in DCM using the same electrolyte as the uranocene references given. The text now reads:

> An irreversible anodic wave (E_{pa} value only) for **1** has been reported at 0.33 V *vs* SCE in dichloromethane[23,24], or –0.13 V when referenced to $[Fc]^{+/0}$ (ref.[33]).

Comment:

2) On Page 4 line 85, the authors state that complexes 3 and 6 “catalyse” the polymerization of THF. I would like to ask the authors to reconsider the statement. Probably the complexes are only initiating the THF polymerization and are not continuously catalysing it?

Response:

The reviewer is absolutely correct – the process is clearly not catalytic as the U(IV) precursor complexes are recovered during the reaction, not the U(V) reactive species that initiate the polymerization. The cause of this incorrect terminology is because the process appears to be a living polymerization, whereby the addition of more THF to the as-generated poly-THF

puck results in further polymer being generated on top. However, at this point, all of the U(V) has been consumed (as determined by UV-Vis-NIR spectroscopy, given in the *Supplementary Information*, Figures now referenced in the main text) and so it is not a catalyst. We have amended the text to state "living-chain polymerization", and have added additional discussion in line with an additional comment from the reviewer which is given below (*minor comment iv*).

Comment:

3) On page 4 line 102, the authors mention previous works that showed irreversible redox waves for 1 and 3 when using TBAPF6, but the reference to the mentioned works is not given there.

Response:

These references were given in the previous sentence, and have now been added after the phrase "as in previous works", for clarity.

Comment:

4) On page 9 lines 223 – 225 the authors characterize the U(V) complexes 3 and 6 as 5f1 centres, yet in the electronic structure calculations it is elaborated that the HOMOs are involved in delta bonding, and hence show some considerable mixing with ligand orbitals in their character. In that context, could the authors comment how such delta bonding is expected to contribute deviations from theoretically expected EPR signals of a uranocenium f1 system without delta bonding (without orbital mixing)?

Response:

Previous work by Gourier et al. (*J. Am. Chem. Soc.* 1998, **120**, 6084-6092) on the bis-(cycloheptatrienyl)-uranium anion $[U(\eta^7\text{-}C_7H_7)_2]^-$, analogous to this work's bis-COT uranocenium, found that the main effect of delta-bonding is to push the $|m_l| = 2$ ($5f_{delta}$) orbitals to much higher energy and hence these states having little effect on the ground state Kramers doublet (KD) and consequently its effective g-values. This leaves mixing with states deriving from $|m_l| = 0,1,3$ to dominate. They also described this in terms of Russel Saunders (RS) terms, with mixing of $|J,m_J\rangle = |5/2,1/2\rangle$ with $|J,m_J\rangle = |5/2,5/2\rangle$ dominating deviations from the pure $|J,m_J\rangle = |5/2,1/2\rangle$ g-values, with $|J,m_J\rangle = |5/2,3/2\rangle$ at much higher energy.

Either way, both these mechanisms lead to a decrease of $|g_{perp}|$ from 18/7 or to highly rhombic g-values (depending on admixtures).

However, this can also be a result of simple crystal field-induced mixing between RS states, hence is not diagnostic of delta-bonding. The work by Solis-Céspedes et al. (*Dalton Trans.*, 2017, **46**, 4834) also considers, via CASSCF on a calculated structure of $[U^{V}(COT)_2]^+$ (which deviates significantly from the actual structure found in our current manuscript), the effect of mixing of LMCT states into the ground KD and, although the direct cause-and-effect on the *g*-values is not clear, they also calculated a reduction in $|g_{perp}|$ from 18/7.

In summary, there are potential multiple and competing effects: the similar relative strengths of spin-orbit coupling, crystal field effects and admixtures of ligand orbitals in the ground state wavefunction makes it difficult to isolate the effect that each individual interaction has on *g*-values. We are currently working on this, and in particular the observed $|g_{perp}| > 18/7$ for **3** and **6**. The paragraph discussing U(V) *g*-values has been modified (text highlighted in yellow) in the following way in the main manuscript:

> The spectra of **3** and **6** are consistent with well-isolated Kramers doublet (KD) ground states. Under the assumption of Russell-Saunders (RS) coupling, the U(V) ion gives a $^2F_{5/2}$ ground multiplet, which is split into a series of KDs by the ligand field. In D_{8h} symmetry, these KDs are pure $|m_J|$ states (although mixing with the $^2F_{7/2}$ excited state is allowed). The experimental effective *g*-values for **3** and **6** are reasonably close to those expected for the pure $|J, m_J\rangle = |5/2, \pm1/2\rangle$ KD ($g_{x,y}$ = 2.57 and g_z = 0.86). Similar *g*-values, but with lower $g_{x,y}$, have been reported by Gourier and co-workers for the related *bis*-(cycloheptatrienyl)-uranium anion $[U^{V}(\eta^7\text{-}C_7H_7)_2]^-$ ($g_{x,y}$ = 2.37 and g_z = 1.24) which they also assign as dominated by the $|J, m_J\rangle = |5/2, \pm1/2\rangle$ KD (ref. [43]). They concluded that deviations were primarily due to mixing with $|J, m_J\rangle = |5/2, \pm5/2\rangle$. They argued that the effect of the delta-bonding is to push the $|m_l| = 2$ ($5f_\delta$) orbitals to much higher energy and hence these states have little effect on the ground state KD. This leaves mixing with states deriving from $|m_l|$ = 0,1,3 to dominate; this can also be described in terms of mixing of $|J,m_J\rangle = |5/2, \pm1/2\rangle$ with $|J,m_J\rangle = |5/2, \pm5/2\rangle$ dominating deviations from the pure $|5/2, \pm1/2\rangle$ *g*-values, with $|J,m_J\rangle = |5/2, \pm3/2\rangle$ at much higher energy. Either way, modelling shows that this leads to a decrease of $|g_{x,y}|$ from 18/7 or to highly rhombic *g*-values (depending on admixtures allowed under the assumed symmetry)[25,43]. CASSCF calculations by Solis-Céspedes

et al.[25] on a calculated structure of $[U^{V}(COT)_2]^+$ consider the effect LMCT states and, although the direct cause-and-effect on the *g*-values is not clear, they also calculated a reduction in $|g_{perp}|$ from 18/7. We note that the calculated geometry in their manuscript deviates from our experimental structure by having (i) co-planar COT ligands, whereas both **3** and **6** are slightly bent, though we cannot discount the influence of crystal packing on the bent structure of either experimental structure; (ii) markedly longer U···$COT_{centroid}$ distances (1.927 or 1.964 Å, using GGA-PBE or B3LYP functionals, respectively) than in **3**; (iii) somewhat longer U–C distances, though the precision on our SCXRD measurements means the difference does not reach statistical significance at the 3σ-level in all cases. Interestingly, similar *g*-values are found for the valence isoelectronic $4f^1$ complexes $[Li(TMEDA)_2][Ce^{III}(COT^{††})_2]$ (see *Supporting Information* **Table S25**; g_x = 2.35, g_y = 2.25, g_z = 1.09) and $[Li(THF)_4][Ce^{III}(COT)_2]$ (g_x = 2.270, g_y = 2.274, g_z = 1.123)[44]. This can be explained by a second-order admixture with the $|J, m_J\rangle = |7/2, \pm 1/2\rangle$ KD of the $^2F_{7/2}$ manifold (see *Supporting Information*). Hence, there are potential competing effects in the observed *g*-values. The models above do not rationalise our finding of $|g_{x,y}| > 18/7$. Our active space calculations, based on the actual structure reported here, give $|g_{x,y}|$ = 2.83 (2.64) and $|g_z|$ = 1.15 (1.12) at the CAS(RAS)SCF level, in good agreement with experiment. Previous calculations on $[U^{V}(COT)_2]^+$ in D_{8h} symmetry gave lower values of $|g_{x,y}|$ = 2.43 and $|g_z|$ = 1.11, albeit with a smaller active space that captured the metal–ligand interaction less precisely[25].

Comment:

5) On a related note to comment 4, Figure S170 of the supplementary information clearly visualizes delta bonding for the HOMO (alpha-MO 101), whereas for the HOMO of compound 6 (alpha-MO 277) there seems to be no visual orbital overlap of a delta bond between the uranium centre and the ligand. Yet on page 10, line 257 of the main text the authors state the HOMO of compound 6 contributes considerably (>40%) to delta-bonding. Could the authors explain why delta bonding interactions are numerically described quite similar for complexes 3 and 6 (5f contributions of 42.7% and 43.0% for 3) while the graphical representations of alpha-MOs in the supplementary information gives a very different impression?

Response:

This is because molecular orbitals derived from DFT methods tend to smear electron density over large parts of the molecule, even when a chemist would quite like to partition them neatly. This is problematic when considering that each MO contains just one electron, and that the images have all been plotted at the same isovalue (0.05 e / Å^3). The issue when comparing **3** to **6** is that the four Si*i*Pr$_3$ groups in **6** result in a slightly greater amount of electron density (from the one electron MO) smeared over a greater number of atoms. Simply plotting the HOMO for **6** at a more forgiving isovalue (0.04 e / Å^3), as shown below, returns an image that is qualitatively the same as that seen with **3**:

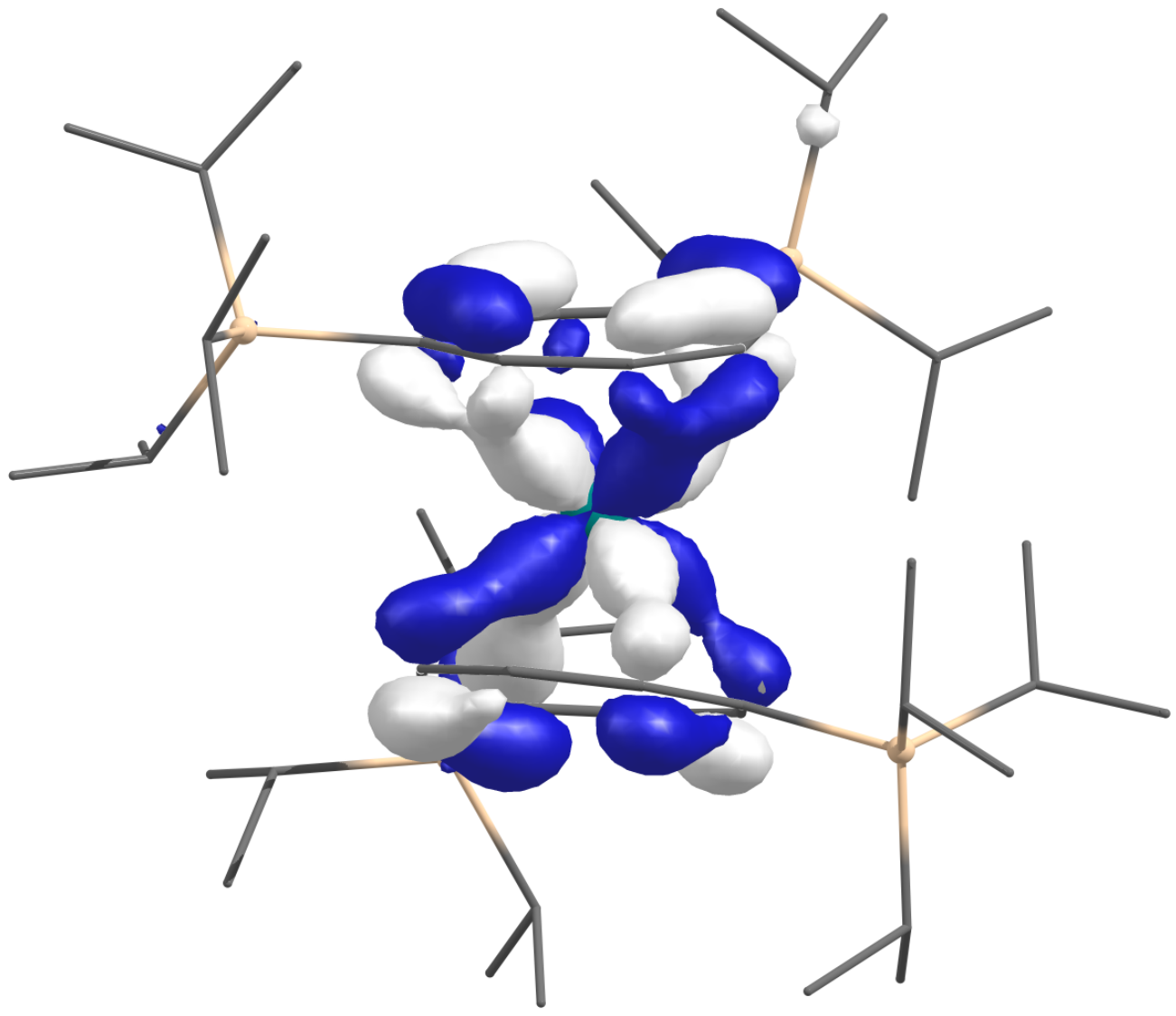

Essentially, while the metal percentage contribution is comparable between **3** and **6**, the ligand part in **6** is smeared a bit more over the larger number of extra atoms – each bearing a very small amount of the total one electron.

We hope this satisfies the reviewer, and we have added a note at the start of the DFT section in the *Supplementary Information* which says:

> In the following sections, MO isosurfaces are all given at the same isovalue (0.05 e Å^{-3}). This makes direct comparison between the unsubstituted (**1**–**3**) and substituted (**4**–**6**) complexes challenging due to the diffuse nature of Kohn-Sham MOs. As such, we also present tables giving the composition of relevant MOs to provide a clearer picture of the metal *vs* ligand involvement.

Comment:

Further minor comments regarding the supplementary information file are:

i) In Figure S2, it seems strange that there are unproportionally tiny C-H stretches compared to all other (closely related) compounds of the study. Could the authors revisit, whether the data set is of appropriate quality?

Response:

We agree with the reviewer and re-measured the spectrum of **3**, as well as that of **2**, as the C–H stretches in the spectrum of **3** are even weaker than those of complex **2**, which the reviewer mentioned. We found these weak stretches to be typical of both complexes, as well as that of **1**. The Figure below shows the original (black) and remeasured (red) data for **2**:

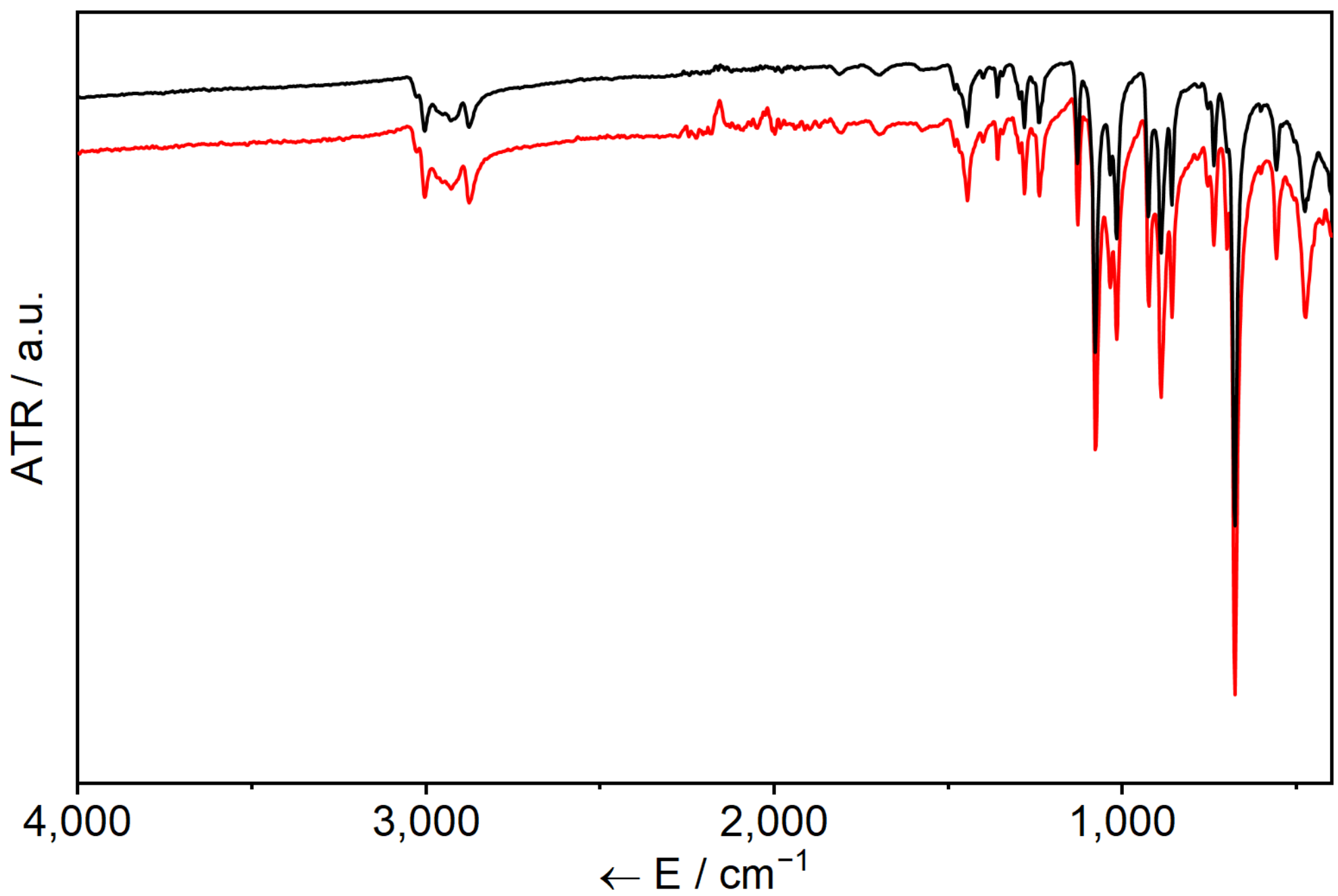

These data suggest that the modest C–H intensity we see in **2** arises mostly from the [Li(12-crown-4)(THF)] unit, and not the $[U^{III}(COT)_2]^-$ anion. Now that we have also included the spectrum of **1** (in answer to the comment below), we can be more confident of this conclusion, as the C–H stretches in **1** are quite a bit weaker than in **2**, both of which are stronger than in **3**.

Prior IR and Raman analyses on $[M(COT)_2]$ complexes have shown that the C–H stretches are indeed very weak, see: *J. Organomet. Chem*. **217**, 169-177 (1981) and *J. Chem. Phys*. **120**, 2708-2718 (2004).

Comment:

ii) For completeness and as a courtesy to future readers, who may seek to find trends across the nice series of reported complexes, it may be useful to also include an IR spectrum of complex 1 to complete the series of data on all compounds in this study.

Response:

This has been measured and included as a new Figure.

Comment:

iii) Also, for the solution phase UV-Vis-NIR spectroscopy data, a complete and comparable series is reported for complexes 4, 5, and 6, but for the unsubstituted uranocene the analogous data set for complex 1 is missing and only data on 2, and 3 are reported. In order to allow readers to draw conclusions easily from data sets that nicely match conditions, it may be appreciated to also report a comparable UV-Vis-NIR data set for 1 at analogous ranges and concentrations.

Response:

This solution data was originally given for **1** in the solid-state UV-Vis-NIR section which included comparison between the solution and solid-state spectra for each complex, but we appreciate this was not obvious. The solution data has now been given separately for **1** as a new Figure.

Comment:

iv) Comparing Figures S41 and S43, it seems the reported t1/2 value is almost identical for complexes 3 (21.4 min) and 6 (24.8 min). Yet the straight portion of the graph, on which the linear regression was performed, is ca. 2.7 times as longer for 6 for than for complex 3. Do the authors have a hypothesis, why it was possible to monitor this very similar reaction so much longer for complex 6, despite the quite comparable reaction rates?

Response:

We thank the reviewer for this insightful question, we had not considered this. The answer is that in the region analysed for **3** (710 nm), there is absorbance from the product complex **1** – therefore, as the decomposition progresses, the difference in absorbance at this

wavelength decreases to insignificance as the concentration of **3** (the starting material) decreases, while simultaneously absorbance from **1** (the product) increases, see the spectra below.

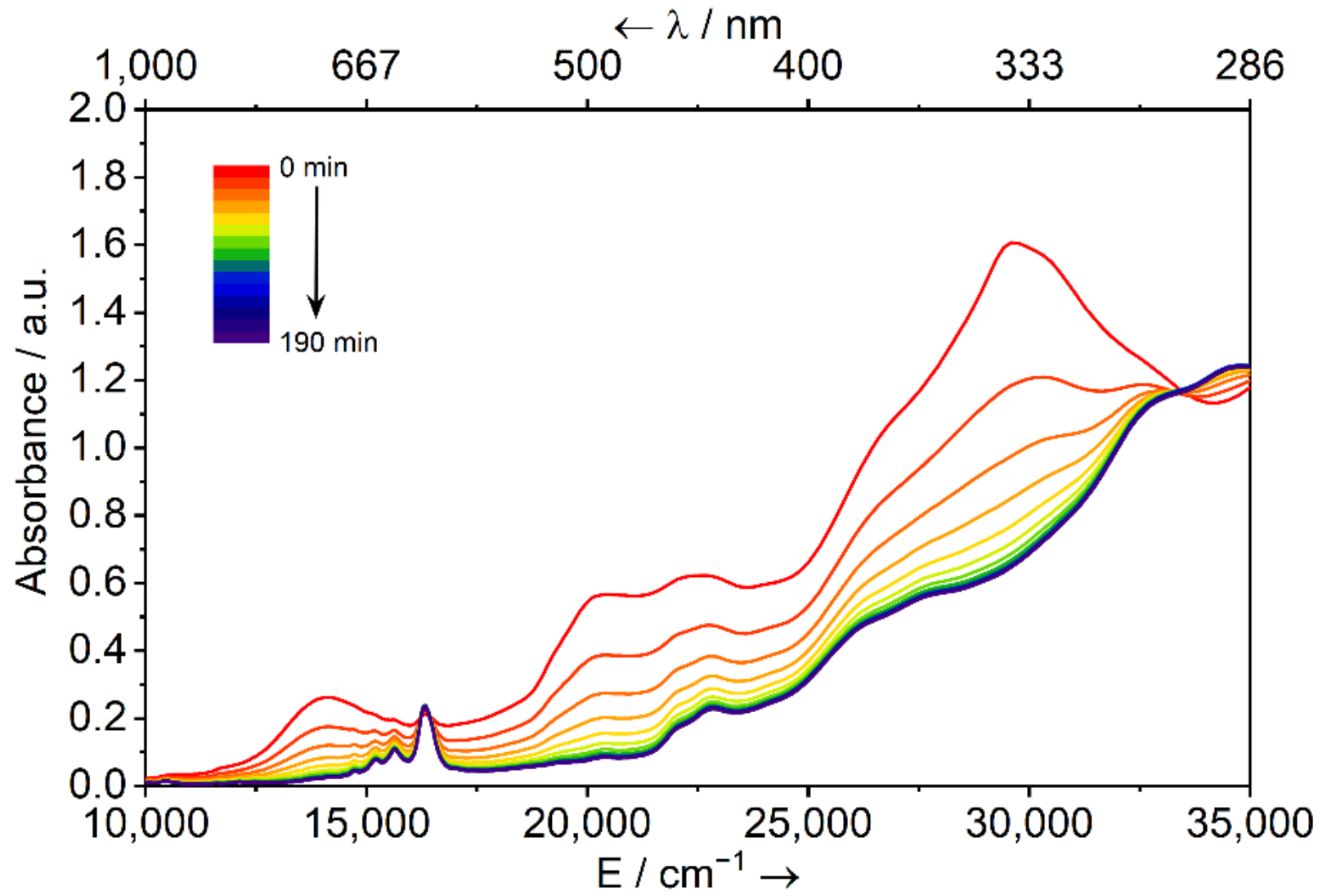

Conversely, in the monitored region for **6** (784 nm), there is essentially no absorbance from **4** (see below).

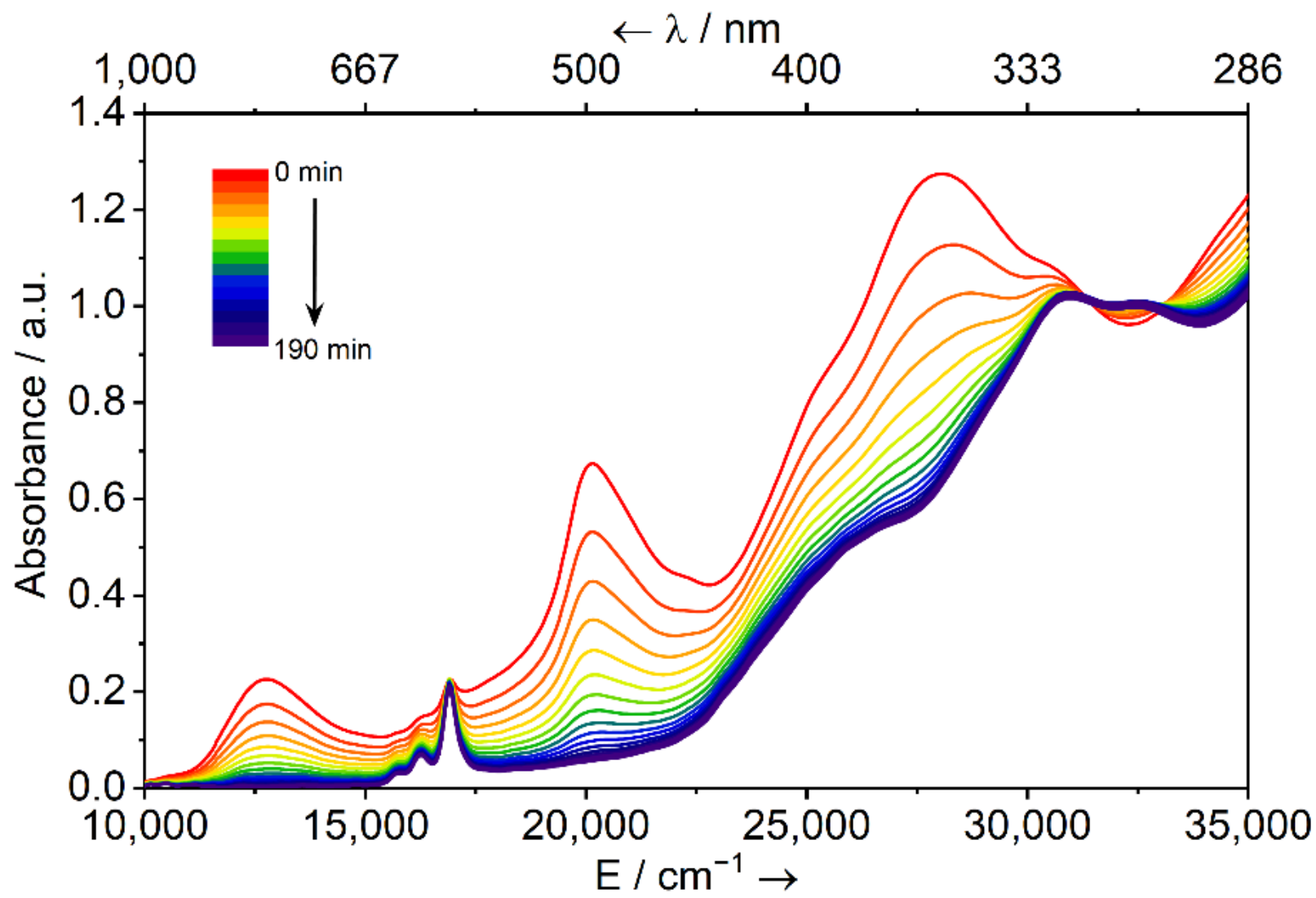

This means that the kinetic data is observing the decrease in concentration of **6**, whereas for **3** there is a convolution of the decreasing concentration of **3** and increasing concentration of **1**. The absorptivity of **1** is small at the observed wavelength, but not zero. During the early measurements, this is not an issue, but towards the end (when the data deviates from the straight line), the problem becomes significant.

However, with the kinetic data in mind, we have expanded the discussion in the main text by a small amount to now read:

> When exposed to THF in *o*DFB solution (1:5 w/w), both complex **3** and **6** initiate the living-chain polymerisation of THF following 1st order reaction kinetics ($t_{1/2}$ = 21.4 and 24.8 minutes, respectively). In both cases we find direct conversion of the U(V) complexes into their respective U(IV) precursors (**1** and **4**) without intermediates observed by UV-Vis-NIR spectroscopy, evidenced by the presence of isospestic points in both. The lifetime of complexes **3** and **6** in neat THF was too short for analysis, but kinetic data gathered in 2-MeTHF revealed longer lifetimes ($t_{1/2}$ = 115.5 and 187.3 minutes, respectively) than in the dilute THF/*o*DFB solutions (see *Supplementary Information*).

Comment:

v) According to Figure S135 and the quite reversible reduction feature at -3.16 V, it appears the authors should be able to synthesize and possibly isolate a doubly reduced complex with a U(II) centre. It is by no means requested to include such efforts into the manuscript presented here, which is already complete in its outline. Yet, I did not want to leave the opportunity uncommented that the authors may want to look into this possibility for future work.

Response:

The data in this Figure (and the ones after it) are from scans where [nBu$_4$N][PF$_6$] was used as the electrolyte.

The first reduction event, when scanning from positive values (denoted as $E_{pc}(2)$ in the Figure labelling), is at approximately the same potential (–2.66 V) as the value for the reductive wave observed for **1** (*i.e.* [U(COT)$_2$]$^{0/–}$) when [nBu$_4$N][B(C$_6$F$_5$)$_4$] was used as the electrolyte (–2.69 V) – both in THF solvent. We therefore believe that the redox pair the reviewer refers to ($E_{pc}(1)$ = –3.28 V / $E_{pa}(1)$ = –3.04 V) is due to a product that forms once the U(III) complex generated by the wave at –2.66 V reacts with the PF$_6$ electrolyte. For example, we postulate that [U^{III}(COT)$_2$]$^–$ could react with [PF$_6$] anions by fluoride abstraction and loss of COT rings either as anions or neutral species. The isolation and characterisation of these products is beyond the scope of this study, but it accounts for the diminished intensity on event $E_{pa}(2)$ (the re-oxidation of [U^{III}(COT)$_2$]$^–$) relative to the reduction of

$[U^{IV}(COT)_2]$, $E_{pc}(2)$, as some of this U(III) material is decomposed during the course of the scan. Additional context has been added to the Figure caption:

> The pair $E_{pc}(1)$ and $E_{pa}(1)$ likely arise from a product derived the reaction between $[U^{III}(COT)_2]^-$, generated at $E_{pc}(2)$, and the and the PF_6-electrolyte. Comparison with the scans where $[n Bu_4N][B(C_6F_5)_4]$ was used as the electrolyte show the generation of $[U^{III}(COT)_2]^-$ at a nearly-identical E_{pc} value (–2.69 V), without the additional cathodically shifted features shown here.

Reviewer 2

Comment:

The manuscript by Deaking et al. reports the synthesis and initial characterization of the quite rare pentavalent uranocenium cation (5f1 configuration). This species is of high interest to the organometallic chemistry community since it completes a classical sandwich series with an actinide in the +3, +4 and +5 oxidation state. This is long sought in testing the variation in 5f versus 6d bonding and the community will be quite excited to see it finally reported. The topic is well suited for publication un the prestigious Nature Communications and I recommend it with the following comments and suggestions.

Response:

We thank the reviewer for their careful review of the work which has provided key points of clarification.

Comment:

Here, the authors used several approaches to probe the 5f1 configuration of uranocenium, as well as family members with U(III) and U(IV), such as EPR, magnetic susceptibility, voltammetry and electronic structure calculations. All data is consistent with the U(V) assignment and the axial (substituted uranocenium 3) or near-axial (6) g-tensor with g values in the range of ~2.8 (g⊥) and ~1.1 (g‖) support the notorious electronic ground state originating from the j=5/2, mj=±1/2 free ion level. The EPR data is quantitatively reproduced by spin-orbit state wavefunction calculations. To this end, I think it would be great to throw a comparison, or at least mention the pentavalent U(C7H7)2- complex made by Didier

Gourier in ’98 (J. Am. Chem. Soc. 1998, 120, 24, 6084–6092). The g-factors for the uranocenium are aligned with those for U(C7H7)2- (2.365 and 1.244), both complexes exhibit f□-f□ mixing due to SOC, and both likely show similar bonding within the delta symmetry.

Response:

We thank the reviewer for pointing this out: this was an oversight in the original manuscript. We have added the reference and an explicit comparison with Gourier’s bis-(cycloheptatrienyl)-uranium anion $[U(\eta^7\text{-}C_7H_7)_2]^-$. They also describe the ground-state of $[U(\eta^7\text{-}C_7H_7)_2]^-$ as primarily $|J, m_J\rangle = |5/2, \pm 1/2\rangle$ KD, although their g_{perp} value is lower than ours. The discussion of the EPR in the main text has been changed extensively, as described above (see response to Reviewer 1).

Comment:

Using a variety of calculations (MO analyses, QTAIM and energy decomposition analyses), the authors show that f-bonding in uranocenium is larger than in U(IV) and U(III) analogues despite there being almost no changes in geometrical parameters. I noted plenty of additional calculations reported in the computational details section (with data present in the SI document) but these are not actually indented anywhere in the manuscript: ETS-NOCV and NLMO analyses, MO diagrams with plenty of DFT functionals such as r2SCAN and variants, etc. Should these be indented in the main manuscript or removed?

Response:

We thank the reviewers for raising this. We performed these calculations for completeness and to provide internal validation of our data, and believe that the community should have access to everything that is relevant to the work. When provided without context, this is perhaps less useful. To address these points, we have added the following passages into the main text at relevant positions:

> Additional DFT functionals were explored with complexes **1** and **3** spanning GGA, meta-GGA, and hybrid(meta)-GGA functionals and a range of included Hartree-Fock exchange (HFX) – see *Supplementary Information*. Analysis of the MO compositions and energies in **1** and **3** showed that separation in energy between the 5*f* and 6*d*-derived δ-bonding MOs was similar across all functionals except when very large values of HFX were included. The most pertinent difference in all cases is how the

energy of the non-bonding 5*f* electron(s) in **1** and **3** changed relative to the δ-bonding MOs. Functionals with greater (or any) Hartree-Fock exchange (HFX) included generally reported the MO which comprises the non-bonding 5*f* electron(s) as close to, or below, the energy of the occupied δ-bonding orbitals, whereas those less, or no HFX included gave this MO as above the δ-bonding orbitals. We proceed with analysis based on orbitals derived from calculations at the PBE0 level has been successful with other actinide systems, and note that while orbital energies have a functional dependence, orbital compositions are similar across all methods.

Analysis of the MO compositions within the natural localised molecular orbital (NLMO) framework revealed qualitatively similar results to the Kohn-Sham orbital analysis above. The uranium contribution to the 5*f* and 6*d*-derived δ-symmetry orbitals increases markedly from U(III) **2**, where no bonding interaction is recovered, to U(IV) **1** [35% U (100% 5*f*) and 18% U (99% 6*d*, 1% 5*f*)], and U(V) **3** [46% U (89% 5*f*, 11% 6*d*) and 41% U (81% 5*f*, 19% 6*d*)]. The results for **4**–**6** are similar (see *Supplementary Information*).

ETS-NOCV data for **3** confirms the energetic results above do not arise simply from internal polarisation within the fragments. In the case of the U(III) complex **2**, polarisation of the non-bonding 5*f* electrons is clearly visible in the NOCV α-channel. Conversely, with U(IV) **1**, and U(V) **3**, there is substantial rearrangement from the ligand fragment into δ-symmetry bonding orbitals which reflects the stronger covalent contribution to bonding in these complexes.

Comment:

A comment concerns the sentence on line 229. Can the authors cross-check if what they mean is that uranocene has 2 electrons in the metal φ-orbitals in a configuration calculated with DFT? Otherwise, it is likely that the two electrons of the U(IV) uranocene are in fσ/fπ or fφ/fπ (or combination of these), at least in the spin-free formalism. With SOC it could be a bigger mess, because electron correlation deems the valence configurations of uranocene near-degenerate and the two unpaired electrons become smeared across the 5f orbitals.

Response:

The reviewer is right to ask for clarity here. The original statement was qualitatively correct at the DFT-level of theory, per the two cited references from the LANL+LBNL collaborations, but not once other factors omitted by DFT are considered. The sentence has been re-worded to now say this outright:

> Prior calculations on uranocene at the density functional theory (DFT) level describe the two unpaired valence 5*f* electrons as residing in metal-localised ϕ-symmetry (e_{3u}) non-bonding orbitals[12,13].

Comment:

Other comments: 1) Cross-check Figur3. A subfigure 3d is referred to in the manuscript but such figure does not exist or the label is missing (I think 3d refers to the two EPR curves at bottom of Figure 3c) .

Response:

We thank the reviewer for spotting this oversight, and they are correct that this was in error. The caption has been re-labelled.

Comment:

2) A 2 subscript is missing in the chemical formula on line 202.

Response:

This has been corrected.

Comment:

3) On line 209, I suggest changing "pure |mj|>1/2 KD" to "pure KD with |mj|>1/2" to avoid someone trying to understand what is KD that gets multiplied into the 1/2 factor.

Response:

We agree this could be made more clear, and have actioned this suggestion.

Reviewer 3

Comment:

This manuscript describes the synthesis and structural characterization of a pair of uranocenes in three oxidation states; the oxidized U(V) cation is of particular interest. The introduction covers the background and rationale for the work very well and the collection, presentation and analysis of the data is very professional. Indeed, I find the science to be of high-quality and carefully completed. However, in my opinion the impact of the manuscript in its current form doesn't quite reach the requisite level of Nature Communications, as explained below.

Response:

We thank the reviewer for their assessment of the work which has prompted us to explore the chemical properties and reactivity of the molecules in this work in greater depth, and politely disagree with their stance on the fit of the work for the journal.

Comment:

The authors have provided a large amount of data but have not convinced this reader that their results are groundbreaking. Clearly it was initially challenging to obtain the structural information for the uranocenium species reported; the use of the Ph3C[B(C6F5)4] reagent in oDFB solvent is a nice route to generate the target species, and it is thus surprising that the final products are, in fact, quite stable in a fairly wide range of solvents and in the solid-state.

Response:

We thank the reviewer for their thought-provoking comment here. It has prompted us to explore in greater detail whether alternative oxidising agents, or alternative reaction solvents, can be used to generate the uranocenium cation complexes **3** and **6**.

With that said, we note that stability is a relative term. The reaction was performed in such a way that decomposition routes cannot be reached under the conditions used – the complexes are sensitive air, moisture, and the ethereal solvents noted in the text. The notion

that the products are stable across a wide range of solvents is incorrect. We listed just three low-polarity, weakly nucleophilic, aprotic solvents in which both **3** and **6** are stable at room temperature (bromobenzene, Et_2O, and *ortho*-difluorobenzene). In response to comments by other reviewers, we have added additional text regarding the solvent-dependent decomposition of **3** and **6** into the main text (see other comments and additions). We hope this is helpful to the reviewer.

Comment:

More importantly, the careful assessment of the source of the previously reported instability of the in situ generated uranocenium cations illustrated that the use of PF6-based electrolytes was responsible for the decomposition of the complex - this is quite nice to see (note that this been observed in other transition-metal systems such as Fe(III) and Mn(III))

Response:

We agree that it has been useful to reconcile the previously reported electrochemistry of uranocene with experimental data showing that $[PF_6]$ activation occurs.

Comment:

and raises several other questions: Does NaSbF6 also F-transfer - this is a common route to avoiding fluoride transfer from these reagents?

Response:

It is not clear what the reviewer is asking for. NaSbF6 is neither an oxidant, nor an electrolyte, and $NaPF_6$ (the PF_6 analogue) was not reacted with any of our complexes. However, inspired by this comment, we have examined the following:

1) Oxidation of **1** with $Ag^{I}[SbF_6]$, which generated a silver mirror (presumably silver metal), and black solids that were insoluble in *o*DFB. NMR spectroscopy showed neutral C_8H_8 as the only identifiable product.
2) Anion exchange between **6** and $[nBu_4N][SbF_6]$ – in analogy to our $[PF_6]$ exchange reaction. The reaction generated black solids which did not dissolve in solvents it did not decompose in (insoluble in *o*DFB and d_5-bromobenzene, appeared to polymerise THF). ^1H NMR spectroscopy of the solution phase showed neutral $COT^{††}$ and the $[nBu_4N]$ cation in approximately 1:1 ratio, and the ^{19}F NMR spectrum showed

$[B(C_6F_5)_4]$ along with a weak signal for $[SbF_6]$. The presence of neutral COT[††] suggests a degree of fluoride abstraction occurs, but the insolubility of the products precludes further analysis.

The results of both of these reactions (and others below) are shown in the *Supplementary Information*, and mentioned in the main text. We have collated the main text entries in response to this, and several other related comments in a response below. We also report the SC-XRD structure of $[nBu_4N][SbF_6]$ for the first time.

Comment:

Are perchlorate salts (often used as electrolytes) also sources of decomposition via a different route?

Response:

The study in Ref. 23 of the original submission (Chambers and Pagni) mentions "There is some evidence that either $(n\text{-Bu})_4N^+ClO_4^-$ or a trace impurity in it participates in the chemical reactions in this system as witnessed by a large and variable reduction wave observed in CVs in the presence of this electrolyte". Given the poor solubility of perchlorate salts in solvents in which **3** and **6** are stable, and the documented reactivity of both **3** and **6** with both $[PF_6]$ and $[SbF_6]$ anions, we prefer to keep the manuscript focused on the work already presented.

Comment:

Do other oxidizing agents in oDFB still generate complex mixtures of products?

Response:

In the reported work, $[Ph_3C][B(C_6F_5)_4]$ does not generate a complex mixture of products and is the only oxidant we originally reported using because it was the first oxidant we explored. Its use was informed by electrochemical measurements, and the focus of the work is on the unusual electronic structure of the products (**3** and **6**), rather than their synthesis alone.

However, it is a somewhat uncommon oxidant due to the formation of a highly soluble byproduct and the proclivity of $[Ph_3C]$ for hydride abstraction reactions. We believe the

reviewer asks a question that other readers may wish answered. As such, we have surveyed the reaction between **1** and several other oxidants:

1) $[Fc][B(C_6F_5)_4]$ (generated **3**).
2) $Ag^I[B(C_6F_5)_4]$ (^1H NMR spectroscopy showed **1** and an unidentified COT-containing product, and no resonances for **3**).
3) $Ag^I[B\{C_6H_3\text{-}3,5\text{-}(CF_3)_2\}_4]$ (^1H NMR spectroscopy showed no traces of **1** and an unidentified COT-containing product. Resonances for **3** were not observed).
4) $Ag^I[SbF_6]$ (^1H NMR spectroscopy showed **1** was consumed, neutral COT was formed, and no other products could be identified from the reaction).

Given that cations of ferrocene, [Fc], and decamethylferrocene, [Fc*], are both widely used as components of outer-sphere non-aqueous one electron oxidants, we have also explored the reactivity of **3** with neutral [Fc] and [Fc*] to see if ligand scrambling and/or electron transfer occurs. As expected from electrochemical measurements, no reaction occurs with [Fc], but [Fc*] is oxidised to give $[Fc][B(C_6F_5)_4]$ and neutral **1**. We have collated the main text entries in response to this, and several other related comments in a response below

Comment:

Why (or is) oDFB a uniquely important solvent to facilitate the oxidation reaction?

Response:

*o*DFB is a solvent that solubilises the reactants and the products, without being reactive towards either, and has a useful range of temperatures at which it remains a liquid. Therefore, it possesses all the requisite properties for the reaction to work.

We have described some common solvents that the products are reactive towards (cyclic ethers), and which therefore would be unsuitable for the reaction. Other common solvents (hydrocarbons such as alkenes and arenes) are not sufficiently polar to solubilise the products, and prior electrochemical studies (Refs 23 and 24) suggested nitriles would likely be unsuitable. Exploration of their use is beyond the scope of this manuscript.

In response to query, we have also examined the oxidation of **1** with $[Ph_3C][B(C_6F_5)_4]$ in both fluorobenzene and d_5-bromobenzene. Both produce **3** (and Gomberg's dimer) as the sole products, as was seen when *o*DFB was used.

Comment:

It is also interesting that there is apparently increased covalency in the U(V) species rather than in the U(III) species, which is perhaps counterintuitive (the manuscript should be examined by a heavy-metal/relativistic computational expert if it hasn't already been). It would be worth investigating this point computationally in a more general sense in other actinide species.

Response:

It is a well-established core concept that metal–ligand covalency is greater with higher oxidation state metals, both in the *f*-block, and with transition metals. This is because increased charge at the metal centre is able to better polarised ligand electron density into the internuclear region. For this reason, we disagree with the reviewer that our findings are counterintuitive.

However, we do wish to highlight that the impact of the much greater covalency in **3**, over that in U(IV) **1** or U(III) **2**, on the molecular structure and redox chemistry was unexpected.

Comment:

As well, given the apparent stability of the uranocenium species, illustrating some of the potentially unique reactivity of this U(V) species (even in limited broad survey form), and a further discussion of under what conditions it decomposes would increase the impact of this manuscript.

Response:

This comment is well-aligned with comments from the other reviewers, and so we have sought to address these together by expanding the passage which details the reactions of **3** and **6** with THF and 2-MeTHF. Please see the other response, and the additional main text content. We do not wish to further expand on this, as the manuscript is focused on the synthesis and unexpected physical properties of these molecules.

Comment:

As is, however, the manuscript is not of sufficient broad scope to warrant publication in Nature Communications in my view and could be published in a more specialized chemistry journal such as RSC Chemical Science. I would encourage the authors to include a survey of some reactivity of the title uranocenium species and compare it with the parent uranocene; this addition would, in my view, elevate this exciting work into Nature Communications.

Response:

We thank the reviewer for their work assessing the manuscript, and politely disagree with their assessment. We have described the synthesis, electrochemistry, spectroscopy (EPR, optical), and electronic structure (from multi-pronged *ab initio* theory calculations) of the first uranocenium complexes, which have been conspicuously absent for over 50 years. We have also demonstrated their reactivity and (in)stability towards some common solvents and provided an experimentally-backed rationale for why these molecules haven't been discovered previously, and corrected the peer-reviewed literature surrounding the electrochemistry of uranocene itself. The manuscript is already comprehensive and detailed.

However, the reviewer raised thought-provoking questions which led us to conduct additional experiments which have been added to the *Supplementary Information*, and comments have been added to the main text where relevant in the passages given below:

Following the section describing the solvent stability of **3** and **6**:

> The oxidation of **1** with $[Ph_3C][B(C_6F_5)_4]$ was also explored in fluorobenzene and d_5-bromobenzene, which gave **3** as the sole uranium-containing product on both occasions, suggesting *o*DFB is not unique as a suitable solvent and others may be appropriate provided they are unreactive towards the formal U(V) centre.

Following the electrochemical discussion:

> The electrochemical data suggest that other oxidants may be suitable for the oxidation of **1**, provided the anionic component is unreactive towards the $[U^{V}(COT)_2]^+$ cation. The oxidants $[Fc][B(C_6F_5)_4]$ and $Ag^{I}[B(C_6F_5)_4]$ both feature the same anion as in **3**, and so the nature of the oxidising component can be examined in isolation. While **1** is smoothly oxidised by $[Fc][B(C_6F_5)_4]$ to give **3**, and indeed **3** is unreactive to [Fc]

as shown in a separate experiment, the reaction with $Ag^I[B(C_6F_5)_4]$ gave neutral COT as the sole identifiable product. Alternative Ag(I) oxidants such as $Ag^I[B\{C_6H_3\text{-}3,5\text{-}(CF_3)_2\}_4]$ and $Ag^I[SbF_6]$ gave similar results (see *Supplementary Information*).

Additions to the section discussing PF_6 reactivity:

However, multinuclear NMR spectroscopy of the solution above the insoluble products over the course of 144 hours showed the growth of signals for PF_3, PF_5, and POF_3 (**Figures S88** and **S89**) as seen with **6**.

Defluorination of $[PF_6]$ is a known degradation pathway for this anion in the presence of Lewis-acids or redox active metal centres[35-38]. Given the propensity for fluoride abstraction from $[PF_6]$ by both **3** and **6**, we explored the anion-exchange reaction of **6** using $[n\text{Bu}_4\text{N}][SbF_6]$ as Sb(V) is more Lewis-acidic than P(V), and therefore perhaps more resistant to defluorination. Complex **6** was chosen due to the greater solubility imparted by the $COT^{\dagger\dagger}$ ligands to aid in the isolation of any crystalline products, as seen with **7**. When d_5-bromobenzene was added to a mixture of $[n\text{Bu}_4\text{N}][SbF_6]$ and **6**, a dark coloured insoluble material quickly formed. ^1H NMR spectroscopy of the solution revealed the presence of neutral $COT^{\dagger\dagger}$, indicating a redox process had occurred, and none of the resonances associated with **6** remained. After 24 hours, the relative integrals of neutral $COT^{\dagger\dagger}$ to the $[n\text{Bu}_4\text{N}]$ cation reached approximately 1:1. The $^{19}F\{^1H\}$ NMR spectrum showed a weak signal corresponding to $[SbF_6]$, but quadrupolar broadening from ^{121}Sb ($I = {}^5/_2$) and ^{123}Sb ($I = {}^7/_2$) precluded the identification of any other Sb-containing products. Given that **6** is stable indefinitely in the solvent the reaction was performed in, these data suggest that the $[U^V(COT^{\dagger\dagger})_2]^+$ cation is unstable in the presence of $[SbF_6]$, but the reaction pathway is unknown at this stage.

We hope that the reviewer agrees with us that additions proposed by themselves, and the other reviewers, have improved the manuscript, and make it suitable for publication in *Nature Communications*.

Response to reviewer comments on NCOMMS-26-019450-B
"Uranocenium – The *bis*-[8]annulene Uranium Cation"

Reviewer 1

Comment:

The authors provide a comprehensive and convincing response letter that addresses all comments adequately. The manuscript is now even more clear and convincing than it was in its original version and I have no further comments or concerns. Accordingly, I recommend to accept the revised version for publication.

Response:

We thank the reviewer for their comments which have improved the article.

Reviewer 2

Comment:

Deakin et al. have revised their manuscript on the synthesis and electronic structure of uranocenium in response to the comments and remarks from me and the other two reviewers. I am overall pleased with their responses, and I reiterate that this is a timely manuscript reporting a formal U(V) f1 complex of significant interest to the wider public working with the f-elements. Two aspects are, in my opinion, still not fully resolved and I recommend considering them in a minor revision.

Response:

We are thankful for the time reviewer 2 has spent shoring-up the theoretical and spectroscopic insights in the article. We have worked hard to address all their points and to strike a balance between the language used by theoreticians, and concepts that are more meaningful to synthetic chemists. We think that with their guidance, this balance has been struck, and the article is better for this.

Comment:

a) It is now nice to see a comparison with the formal U(V) complex by Gourier, U(C7H7)2^-, but I find this comparison not complete. For instance, despite being formally 5f1, this complex is generally accepted as a U(III) complex due to large bonding involving the 5f_delta orbitals. See for instance: J. Am. Chem. Soc. 1997, 119, 9021; Chem. Sci., 2018,9, 6292; Chem. Commun., 2021,57, 9562. In this context, should the uranocenium also be regarded a U(III)? This kind of comparison would also complete comment 4 from reviewer 1.

Response:

The reviewer cites three articles: (1) https://doi.org/10.1021/ja971149m; (2) https://doi.org/10.1039/c7sc05373h; and (3) https://doi.org/10.1039/d1cc03414f.

Article (1) by Bursten and Li arrives at the conclusion that the $[U(C_7H_7)_2]^-$ anion is best viewed as a U(III) complex by noting that the two sets of δ-symmetry bonding orbitals are doubly-occupied (holding four electrons total) and are roughly 50% U-character, and so they have partitioned the four electrons between the U-atom, and the ligands – arriving at U(III). This is conceptually simple and is very similar to the orbital description we find for $[U(COT)_2]^+$.

However, we feel this argument does not mesh well with spectroscopic data that support a description of the U-centre in $[U(COT)_2]^+$ as distinct from the unambiguous U(III) in $[U(COT)_2]^-$. Two sentences in Article (2) capture the issue elegantly: "*We note that the assigned formal oxidation state and the actual metal charge may be different*" and "*In any case, the assigned formal metal oxidation state, being either III or V, has no bearing on the actual electron (spin) density in U(C7H7)2−*". We think the distinction here is semantic and depends on how the reader wishes to interpret the complexes between: (i) formal oxidation states derived from partitioning electrons from electronegativity arguments (*i.e.* 2 x COT^{2-}, $[B(C_6F_5)_4]^-$, therefore U^V); (ii) experimental data (EPR spectroscopy and SQUID magnetometry show the uranocenium cations are entirely distinct from the genuine U^{III} anion complexes) – one could term this the spectroscopic oxidation state; and (iii) theoretical methods which partition electron density in nuanced ways. Throughout the text we have, therefore, chosen to carefully describe the formal oxidation state of each complex. We have amended the section below just above the electronic structure calculations section (additions in yellow):

Taken together, these experimental data describe complexes **3** and **6** as containing formal U(V) $5f^1$ metal centres, despite the presence of two strongly reducing dianions around a highly oxidising U(V) centre. Some authors have argued the U-centre in $[U(\eta^7\text{-}C_7H_7)_2]^-$ is best described as U(III) based on computational studies and partitioning pairs of bonding electrons in mixed ligand and $5f_\delta$ orbitals[47], whereas EPR spectroscopy and magnetic studies have been interpreted in terms of a formal U(V) oxidation state in $[U^V(\eta^7\text{-}C_7H_7)_2]^-$ with extensive $5f_\delta$ mixing[43]. Others highlight the nuance between assigning formal oxidation state and descriptions of electron density or metal charge derived from spectroscopic and theoretical analyses[48,49]. The same criteria used to assign $[U^V(\eta^7\text{-}C_7H_7)_2]^-$ as containing a U(III) centre also apply to complex **3** which has very high uranium content in the bonding δ-orbitals. However, magnetic, spectroscopic, and various computational analyses comparing the unambiguous U(III) complex **2** to formal U(V) complex **3** show they are entirely distinct. It is for this reason that we describe the formal oxidation state of the metal in all cases herein, and defer to further spectroscopic investigations into the new actinocenium class of molecules.

Comment:

b) I had initially a comment on the two electrons in the ground state of uranocene being distributed both in the 5f_phi orbitals. The authors did clarify that this is indeed an assignment from the perspective of DFT calculations and provided two prior citations in support. DFT clearly gives the wrong assignment, since high level calculations, MCSCF and CASPT2//CASSCF (some which were performed by authors here), provide the (f_pi^1)(f_phi^1) distribution of the 2 electrons: J. Phys. Chem. A, 113, 8737 (2009); Liu, Dolg & Fulde, J. Chem. Phys., 107, 3584 (1997).

I find it necessary to at least mention here that these DFT ground state configurations are in disagreement with data provided with more advanced theoretical treatments.

DCS, UAIC.

Response:

We agree with the reviewer that it would be unhelpful to the community to only mention the corroborating DFT-centric description of the electronic structure without more explicitly

referencing the higher-level methods that provide an alternative picture. We have added the following sentences into the starting paragraph of the electronic structure section (new in yellow):

> Prior calculations on uranocene at the density functional theory (DFT) level describe the two unpaired valence 5*f* electrons as residing in metal-localised $5f_{\phi}$-symmetry (*i.e.* $5f_{\phi}{}^{2}$, e_{3u} under D_{8h} symmetry) non-bonding molecular orbitals (MOs)[12,13]. This contrasts with calculations performed at higher levels of theory, which give a $5f_{\pi}{}^{1}5f_{\phi}{}^{1}$ configuration[47-49]. Nevertheless, topological analyses of the electron density *via* the quantum theory of atoms in molecules (QTAIM) approach in complex **1** reveals qualitatively similar results using wavefunctions derived from DFT calculations[50], and from CASSCF calculations[47].

Reviewer 3

Comment:

The authors have suitably addressed the points of the referees in this revision and I thank them for including substantial additional data and some commentary regarding reactivity. I am happy to recommend publication in Nature Communications.

Response:

We thank the reviewer for suggesting several thought-provoking synthetic avenues to explore which we feel have broadened the insights we have now presented, and we are grateful for their time spent on the article.
